# A Velvet Transcription Factor Specifically Activates Mating through a Novel Mating-Responsive Protein in the Human Fungal Pathogen Cryptococcus deneoformans

**DOI:** 10.1128/spectrum.02653-21

**Published:** 2022-04-26

**Authors:** Huimin Liu, Xiaoxia Yao, Weixin Ke, Hao Ding, Guang-Jun He, Shuang Ma, Yan Peng, Xinping Xu, Guojian Liao, Xiuyun Tian, Linqi Wang

**Affiliations:** a School of Life Sciences, Division of Life Sciences and Medicine, University of Science and Technology of China (USTC), Hefei, China; b State Key Laboratory of Mycology, Institute of Microbiology, Chinese Academy of Sciences, Beijing, China; c University of Chinese Academy of Sciences, Beijing, China; d Jiangxi Institute of Respiratory Disease, The First Affiliated Hospital of Nanchang University, Jiangxi, China; e College of Pharmaceutical Sciences, Medical Research Institute, Southwest University, Chongqing, China; South China Sea Institute of Oceanology, Chinese Academy of Sciences

**Keywords:** *Cryptococcus*, sexual reproduction, mating, cell-cell fusion, velvet protein family

## Abstract

Sexual reproduction facilitates infection by the production of both a lineage advantage and infectious sexual spores in the ubiquitous human fungal pathogen Cryptococcus deneoformans. However, the regulatory determinants specific for initiating mating remain poorly understood. Here, we identified a velvet family regulator, Cva1, that strongly promotes sexual reproduction in C. deneoformans. This regulation was determined to be specific, based on a comprehensive phenotypic analysis of *cva1*Δ under 26 distinct *in vitro* and *in vivo* growth conditions. We further revealed that Cva1 plays a critical role in the initiation of early mating events, including sexual cell-cell fusion, but is not important for the late sexual development stages or meiosis. Thus, Cva1 specifically contributes to mating activation. Importantly, a novel mating-responsive protein, Cfs1, serves as the key target of Cva1 during mating, since its absence nearly blocks cell-cell fusion in C. deneoformans and its sister species C. neoformans. Together, our findings provide insight into how C. deneoformans ensures the regulatory specificity of mating.

**IMPORTANCE** The human fungal pathogen C. deneoformans is a model organism for studying fungal sexual reproduction, which is considered to be important to infection. However, the specific regulatory determinants for activation of sexual reproduction remain poorly understood. In this study, by combining transcriptomic and comprehensive phenotypic analysis, we identified a velvet family regulator Cva1 that specifically and critically elicits early mating events, including sexual cell-cell fusion. Significantly, Cva1 induces mating through the novel mating-responsive protein Cfs1, which is essential for cell-cell fusion in C. deneoformans and its sister species C. neoformans. Considering that Cva1 and Cfs1 are highly conserved in species belonging to Cryptococcaeceae, they may play conserved and specific roles in the initiation of sexual reproduction in this important fungal clade, which includes multiple human fungal pathogens.

## INTRODUCTION

**FIG 1:**
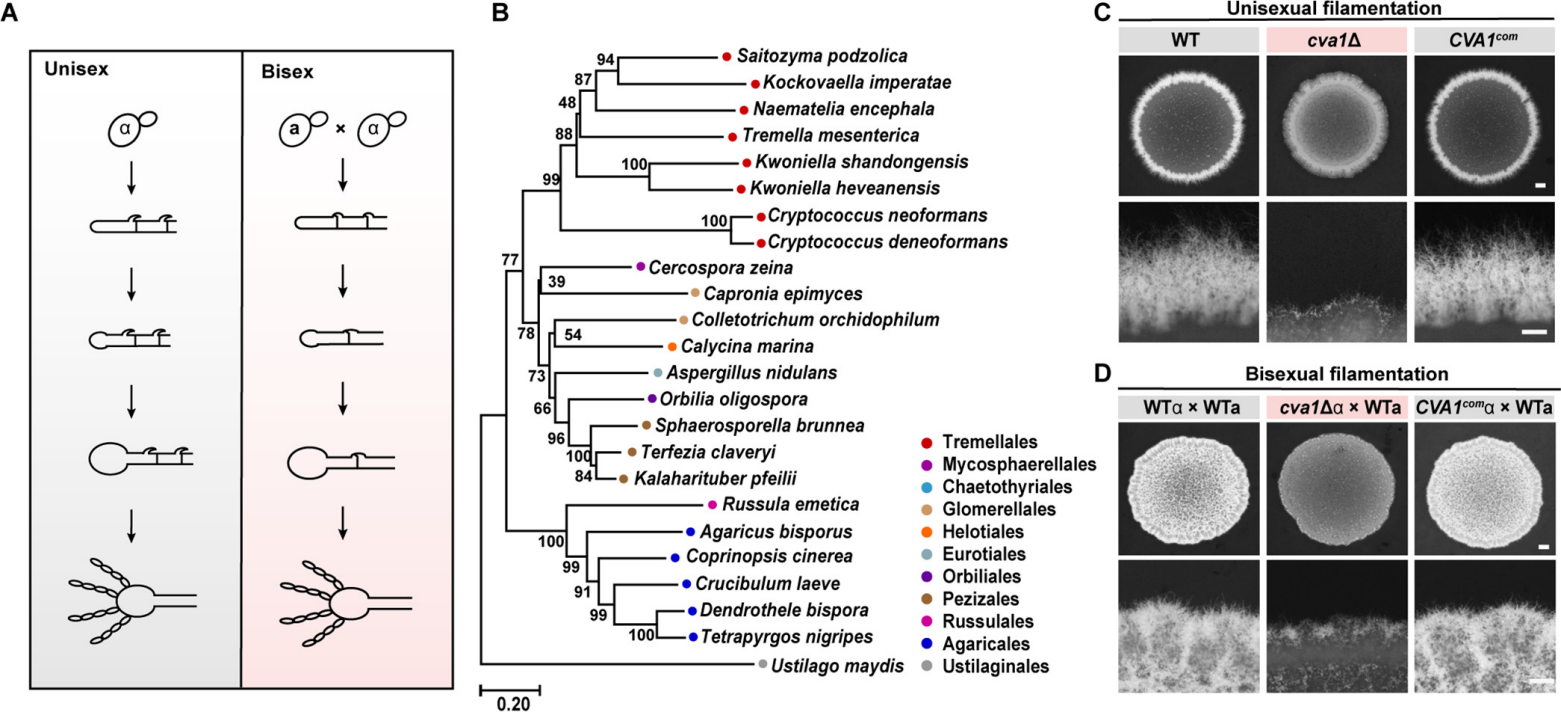
Cva1 is conserved in evolutionarily divergent fungi and is required for hyphal development induced by mating cue in C. deneoformans. (A) Diagram depicting unisexual and bisexual cycles in C. deneoformans. (B) Phylogenetic tree of CNA05460 homologues. Protein sequences were aligned using the neighbor-joining method with the MEGA program, version 7.0.26. (C) The hyphal morphology of wild-type (WT) and *cva1*Δ strains at the colony level during unisexual reproduction. All patches were spotted on V8 medium and incubated in the dark at 25°C for 7 days. Bars, 1 mm (top panel) and 400 μm (bottom panel). (D) Colony morphology of the cross between α isolates (wild-type or *cva1*Δ) and wild-type **a** strain on V8 agar at 25°C in the dark for 3 days. Bars, 1 mm (top panel) and 400 μm (bottom panel).

Sexual reproduction is a unique feature of eukaryotes, including fungi (1–5). A key model organism for investigating fungal sexual reproduction is the human pathogen Cryptococcus deneoformans (6–10), which can cause severe fungal pneumonia and meningitis (11–16). This pathogen has two opposite mating types, *MAT*α and *MAT***a** (1, 5, 17). It can undergo two defined sexual cycles: unisexual reproduction, which occurs mostly in the *MAT*α cell type, and α-**a** bisexual reproduction (1, 7, 9, 18–20). The sequential events that characterize these two sexual cycles are similar (9, 19) (Fig. 1A). Once the mating cue is available, mating response genes are induced synchronously, further enabling the initiation of the morphological transition from yeasts to hyphae (1, 7, 10, 19, 21, 22). The tips of some of the aerial hyphae subsequently differentiate into sexual structures known as basidia, where meiosis takes place (7, 19, 20, 23). Successful spatiotemporal coordination of basidial maturation and meiotic progression leads to the formation of four chains of basidiospores (9, 19, 23–26).

Several lines of evidence have demonstrated the importance of sexual reproduction to infections at different levels in C. deneoformans. For instance, sex-created genetic diversity and ploidy variation can facilitate the emergence of hypervirulent or drug-resistant progenies (1, 7, 9, 19, 27). In addition, sexual reproduction offers an exclusive route to the production of infectious spores (basidiospores), since asexual sporulation does not occur in this species (28–32). Given the significance of sexual reproduction in C. deneoformans, the underlying genetic basis has been extensively investigated, and multiple signaling pathways and genes involved in sexual reproduction have been explored (6–8, 19, 33–35). However, most of these pathways and genes appear to function pleiotropically rather than specifically contributing to sexual development, the regulatory determinants specific for sexual activation in C. deneoformans remain poorly understood.

Here, we identified in C. deneoformans a velvet regulator that we named Cva1, which specifically activates sexual reproduction. Transcriptomic and phenotypic analysis indicated that Cva1 is dispensable for late sexual differentiation stages or meiosis but exerts a critical function on the initiation of early mating events, including cell-cell fusion (sexual syngamy). Furthermore, we identified a novel cell mating-responsive protein, Cfs1, as the key target of Cva1. Cfs1 is strongly induced in response to mating cues and plays a vital role in sexual syngamy in C. deneoformans and its sister species C. neoformans. These findings contribute to our understanding of the underlying mechanisms for the regulatory specificity of sexual reproduction in different Cryptococcus pathogens.

## RESULTS

### Velvet regulator Cva1 is required for sexual filamentation but not mating-independent filamentation evoked by glucosamine.

To investigate determinants important for sexual reproduction, we focused on a velvet family gene (CNA05460) that displayed a highly dynamic expression throughout the unisexual cycle, according to publicly available transcriptomic data (23) (Fig. S1A). This result was further confirmed by examining the expression of mCherry-tagged CNA05460 in response to mating stimulation, which showed evident fluorescent signals in the nuclei of a large number of cells cultured on mating-inducing medium (Fig. S1B). The velvet protein produced by CNA05460 is highly conserved among species belonging to the order Tremellales and shares a remarkable similarity with VelB from Aspergillus nidulans (34% coverage, 48% identity) (Fig. 1B), which is known to play pleiotropic roles in various biological processes (36, 37).

To examine the impact of gene CNA05460 on sexual development, a deletion mutant was generated in C. deneoformans strain XL280, which can undergo robust unisexual and bisexual reproduction (18, 38). As shown in Fig. S1C, the resulting mutant showed dramatic defects in self-filamentation (also known as unisexual filamentation) when cultured alone on V8 medium (mating-inducing conditions) (39, 40). Even after extended incubation on V8 medium, only sparse aerial hyphae were detected. Reintroduction of the wild-type CNA05460 gene complemented this hyphal defect (Fig. 1C). To examine whether CNA05460 is also required for bisexual filamentation, we performed a unilateral mating assay. It was found that filamentation produced via mating between CNA05460Δ α and wild-type **a** was substantially impaired compared with that generated via crosses of wild-type α with wild-type **a** or of complemented strain α with wild-type **a** (Fig. 1D).

**FIG 2:**
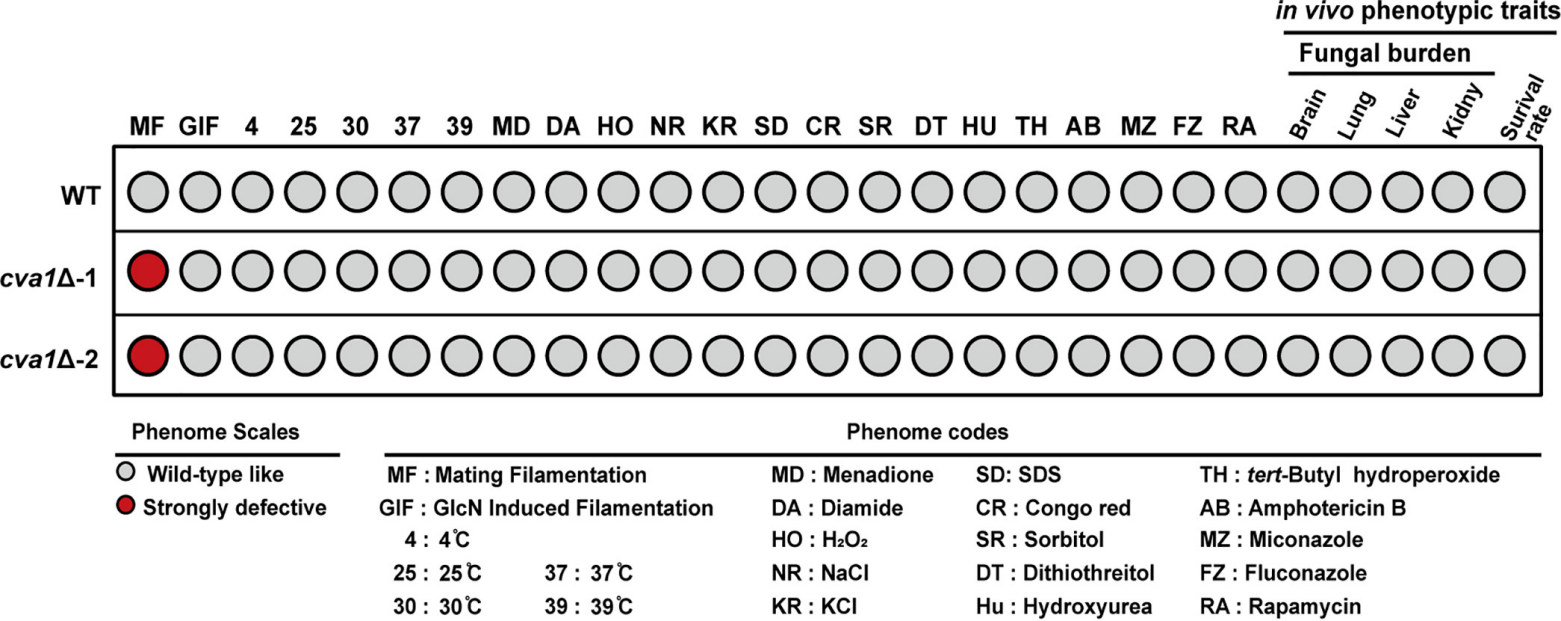
Cva1 is specific for sexual reproduction in C. deneoformans. Phenotypic scores based on qualitative or semiquantitative measurements of mutants lacking Cva1 under distinct *in vitro* and *in vivo* growth conditions. The meanings of the colors and abbreviations are noted in the legend below the figure.

In C. deneoformans, filamentation can be stimulated by the presence of either mating signal or glucosamine (GlcN), the monomer of cell wall chitosan (41, 42). Interestingly, unlike its importance in mating signal-stimulated filamentation, deletion of CNA05460 did not lead to a detectable defect of GlcN-induced filamentation (Fig. 2; Fig. S2A). Because GlcN-induced filamentation has been demonstrated to be independent of the mating process (41, 42), these results suggest that CNA05460 plays a specific role in sexual development. Given this important role of CNA05460 in the sexual development of C. deneoformans, we named the gene *CVA1* (Cryptococcus velvet sex activator 1).

### Comprehensive phenotypic analysis suggests that *CVA1* is specific for the control of sexual reproduction.

The fact that *CVA1* promotes sexual filamentation but not the mating-independent hyphal differentiation stimulated by GlcN suggests that it may serve as a key regulator specific to sexual reproduction. To test this idea, we assessed the effect of *CVA1* on other physiological functions by monitoring the change in growth caused by depletion of Cva1 under a variety of growth conditions. These conditions included high temperature, high osmotic strength, high salt concentrations, presence of oxidants, presence of chemicals that destabilize the cell membrane or cell wall, presence of chemicals that induce ER stress, and presence of antifungal agents. As shown in Fig. 2 and Fig. S2B, disruption of *CVA1* did not lead to detectable defects in growth under any of these conditions.

We performed further investigations to determine whether Cva1 plays a role in the virulence of C. deneoformans. To achieve this, C57 BL/6 mice were infected with wild-type or *cva1*Δ cells via the intravenous route. Fungal burdens in the lungs, brains, livers, and kidneys of infected mice were evaluated 14 days after infection. The absence of Cva1 did not result in a significant change in the fungal burden in any organs tested in this study, suggesting that Cva1 is independent of cryptococcal survival during infection (Fig. 2; Fig. S3A). Consistent with this idea, no significant differences were observed in body weights or mortality rates of animals infected by wild-type or *cva1*Δ fungi (Fig. S3B and C). Taken together, these data indicate that Cva1, which is essential for sexual reproduction, does not appear to function under the aforementioned *in vivo* and *in vitro* growth conditions, supporting the specific role of Cva1 in mating.

### Cva1 is dispensable for late sexual differentiation events and meiosis in C. deneoformans.

**FIG 3:**
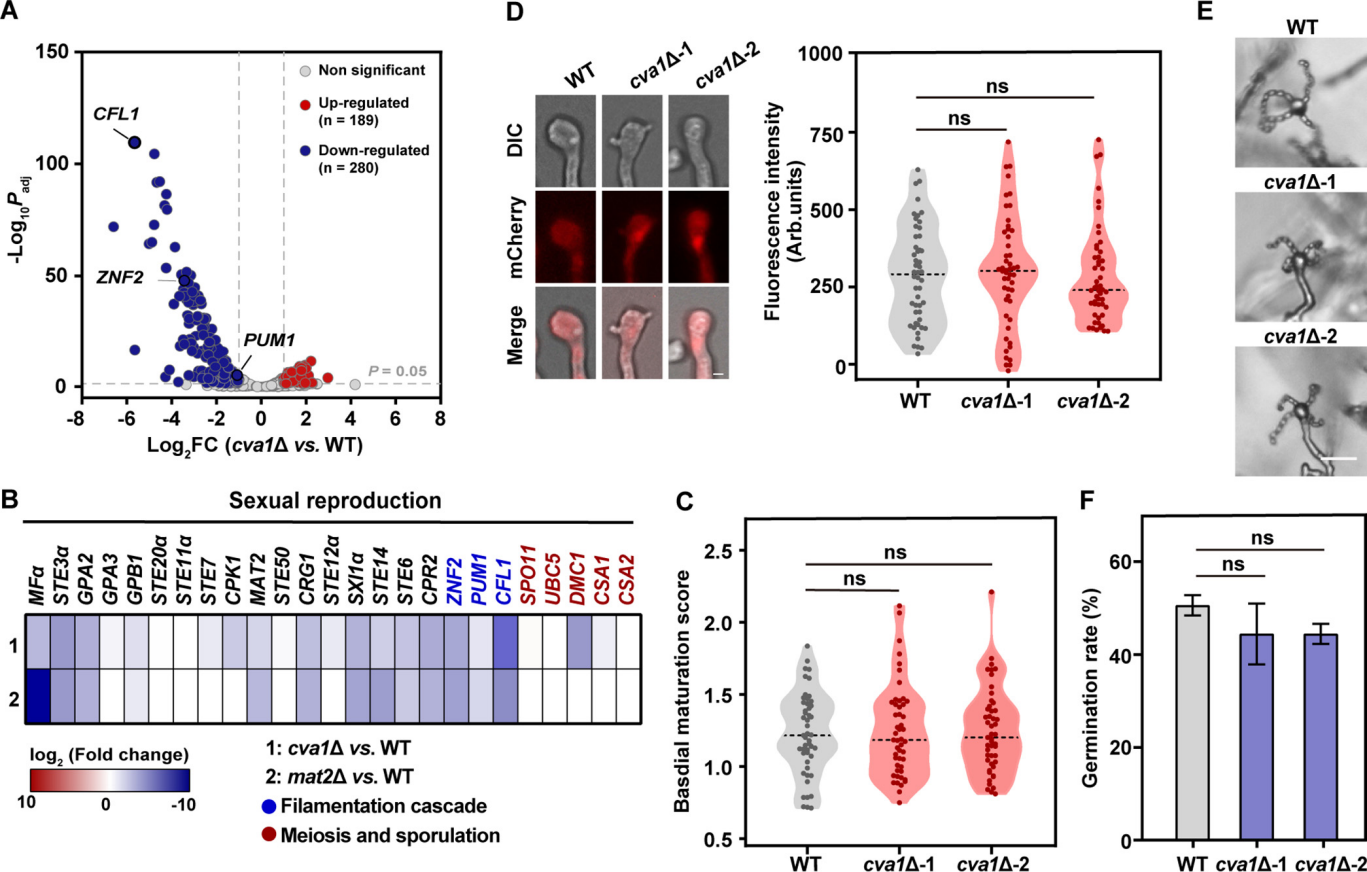
Cva1 mediates sexual activation but is dispensable for late sexual phases. (A) Volcano diagram of the differentially expressed genes (DEGs) in comparison between wild-type and *cva1*Δ strains in the XL280α background. (B) The expression levels of genes under mating-inducing conditions at 24 h. The legend color bar represents log_2_ relative expression values. Identification of the DEGs between *mat2*Δ and the wild-type XL280α strain was based on publicly available transcriptomic data (35). (C) Violin plot analysis shows the basidial maturation score distribution of wild-type and *cva1*Δ strains. Basidia were photographed at 7 days (*n* = 50). ns, not significant (two-tailed Student’s *t* test). (D) The localization and expression of Dmc1-mCherry in basidia during unisexual reproduction of *cva1*Δ compared with the wild-type strain. The images were taken at 7 days (*n* = 50). ns, not significant (two-tailed Student’s *t* test). DIC, differential inference contrast Bar, 5 μm. (E) Sporulation phenotypes of different strains during unisexual mating. Bar, 20 μm. (F) Germination rate of basidiospores from the wild-type strain and deletion mutants. The data are presented as the means ± SD (*n* = 2). ns, not significant (two-tailed Student’s *t* test).

We next sought to investigate the mechanism underlying the regulatory function of Cva1 during unisexual development by conducting high-coverage strand-specific RNA sequencing (RNA-seq) analysis targeting wild-type and *cva1*Δ strains. The RNA-seq experiments were performed using cryptococcal cells incubated for 24 h after sexual induction, when a majority of mating-responsive genes have been shown to display dynamic expression, according to our previous study (23). Here, our analysis revealed 6,905 genes predicted to encode proteins, which covered 99.2% of the protein-coding genes in the XL280 genome. Among these genes, 469 differentially expressed genes (DEGs) were identified in response to Cva1 absence, including 189 genes downregulated and 280 genes upregulated by Cva1 (|Log_2_(fold change)| > 1.0, *P*_adj_ < 0.05) (Fig. 3A; Table S1).

Among these DEGs, we noticed that deletion of *CVA1* caused a dramatic decrease in the expression of genes responsible for filamentation, including the filamentation master regulator *ZNF2*, as well as its key downstream targets *CFL1* and *PUM1* (21, 35, 43) (Fig. 3A and B). This finding is consistent with our phenotypic data showing that Cva1 is required for sexual filamentation.

In contrast, Cva1 appears not to notably affect the expression of the majority of genes involved in the late sexual stages, including basidial maturation, meiosis, and sporulation (Fig. 3B). These data further support the dispensability of any roles for Cva1 in late sexual events. To test this idea, we conducted quantitative phenotypic analyses to examine the abilities of the *cva1*Δ strain to undergo basidial maturation, meiosis, sporulation, and spore germination. We found that the absence of Cva1 did not cause significant changes in basidial development, as revealed by the results of a basidial maturation score assay, which we developed to quantitatively evaluate basidial maturation (23) (Fig. 3C). In addition, we assessed the impact of Cva1 on meiotic activity with mCherry-tagged Dmc1, a meiosis-specific recombinase, serving as a molecular indicator for meiosis (18, 43). In this set of experiments, a nearly identical level of fluorescence from Dmc1-mCherry was observed in the basidia from the wild-type strain and the *cva1*Δ strain, suggesting that Cva1 does not influence meiosis (Fig. 3D). Furthermore, we found that the *cva1*Δ strain undergoes sporulation normally, and no detectable difference in the germination rate of basidiospores was observed between the mutant and wild-type strains (Fig. 3E and F). Together, these results demonstrate that Cva1 is not critical for the late stages of sexual development in C. deneoformans.

### Cva1 plays an important role in early mating events in C. deneoformans.

**FIG 4:**
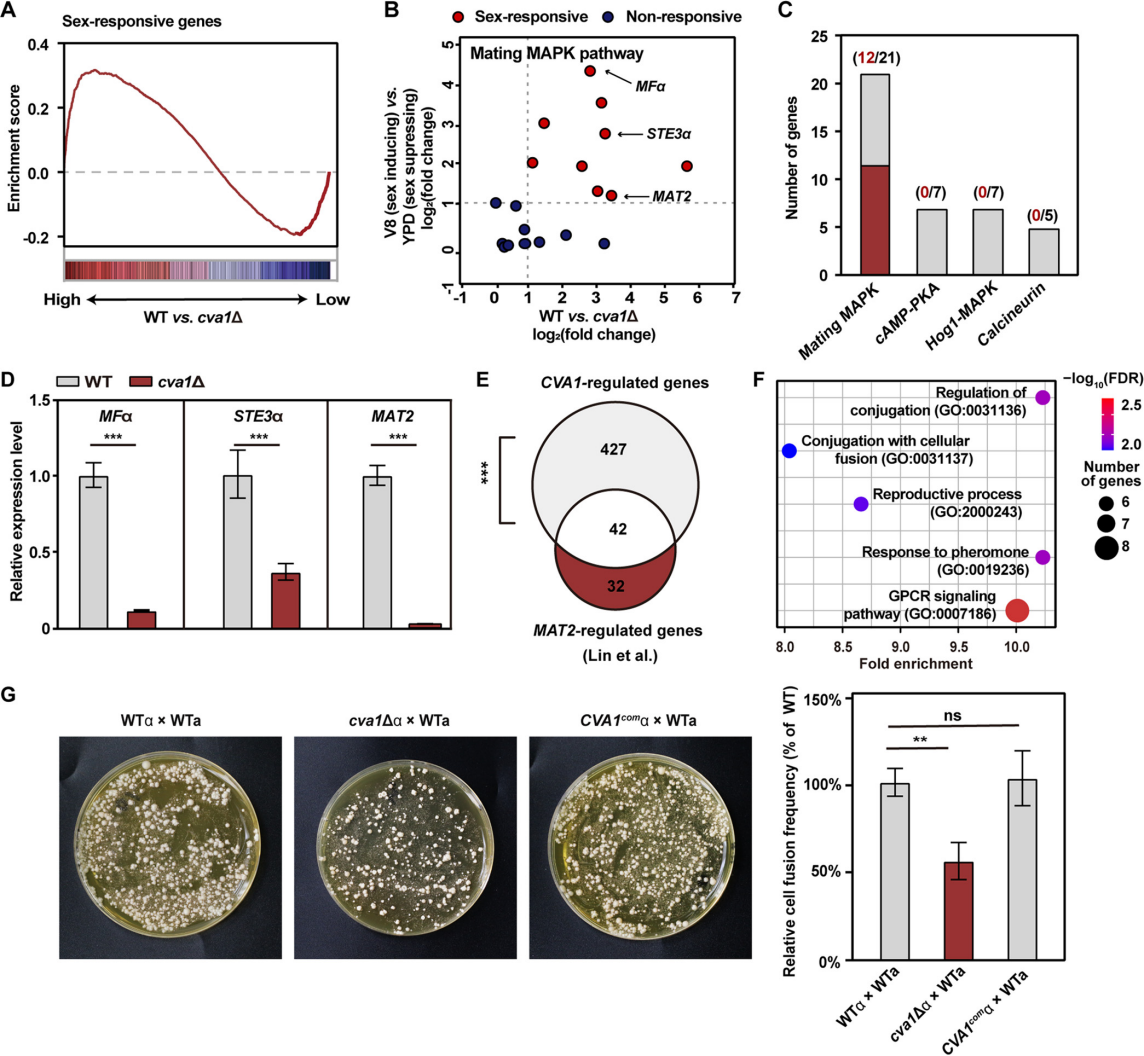
Cva1 specifically induces genes involved in early mating events. (A) A significant enrichment of sex-responsive genes in the *CVA1* regulon based on gene set enrichment analysis (ES = 0.28, *P < *0.01). ES, enrichment score. Sex-responsive genes of C. deneoformans were identified in a previous study (6). (B) Many mating cue-activated genes in the mating mitogen-activated protein kinase (MAPK) pathway are activated by Cva1. The arrows indicate three genes encoding the pheromone Mfα, pheromone receptor Ste3α, and key regulator Mat2. (C) Many genes activated by Cva1 belong to the mating MAPK cascade but not to other pathways that are not specific for sexual activation. (D) Results of a quantitative real-time PCR (qRT-PCR) assay showing the relative mRNA levels of the genes encoding pheromone, its receptor, and Mat2 in the *cva1*Δ mutant during sexual reproduction compared to wild-type. The data represent the means ± SD (*n* = 4). ***, *P < *0.001; two-tailed Student’s *t* test. (E) Comparison of Cva1-regulated and Mat2-regulated genes under mating-inducing conditions. Transcriptome data of the *mat2*Δ mutant were obtained from previous research (35). (F) Gene Ontology (GO) terms associated with mating of C. deneoformans were significantly enriched in the *cva1*Δ mutant. The vertical axis indicates GO terms. (G) Cell fusion products of the indicated crosses for 15 h on V8 medium at 25°C in the dark in C. deneoformans (left). Unilateral cell fusion frequency of the indicated strains. The data represent the means ± SD (*n* = 3), ND, not detected. **, *P < *0.01; ns, not significant (two-tailed Student’s *t* test) (right).

Gene Set Enrichment Analysis showed significant enrichment of early sex-responsive genes among the Cva1 regulon (*P < *0.01, permutation test) (Fig. 4A), suggesting its importance in early mating events. These early sex-responsive genes include multiple elements of the mating mitogen-activated protein kinase (MAPK) pathway, which represents the core signaling cascade that initiates mating in C. deneoformans and other fungi (19, 44–47) (Fig. 4B). In contrast, we did not detect the enrichment of genes from other well-known signaling cascades that are not dedicated to mating in C. deneoformans (48–50) (Fig. 4C).

We further investigated the function of Cva1 on the induction of mating MAPK genes using quantitative real-time PCR (qRT-PCR) analysis, which indicated a significantly reduced expression of the key mating MAPK genes in the absence of Cva1 (Fig. 4D). These genes are those coding for the pheromone *MF*α, the pheromone receptor *STE3*α, and the mating master regulator *MAT2*. The latter gene, in particular, has been shown to be essential for α-**a** cell-cell fusion (35). The tight regulatory relationship between Mat2 and Cva1 was further confirmed by the identification of a significant overlap between targets regulated by these two regulators (Fig. 4E). Furthermore, Gene Ontology (GO) analysis of the Cva1-regulated genes revealed GO terms related to sexual cell-cell fusion, such as conjugation with cellular fusion (GO:0031137) (Fig. 4F).

To experimentally corroborate the involvement of Cva1 in α-**a** cell-cell fusion, we conducted unilateral mating assays. As shown in Fig. 4G, the efficiency of cell-cell fusion between *cva1*Δ α and wild-type **a** strains was significantly lower than that between wild-type α and wild-type **a** strains. As expected, this defect was able to be rescued by complementation of *CVA1* in the *cva1*Δ strain (Fig. 4G).

### Cfs1 is a key target of Cva1 and governs cell-cell fusion.

**FIG 5:**
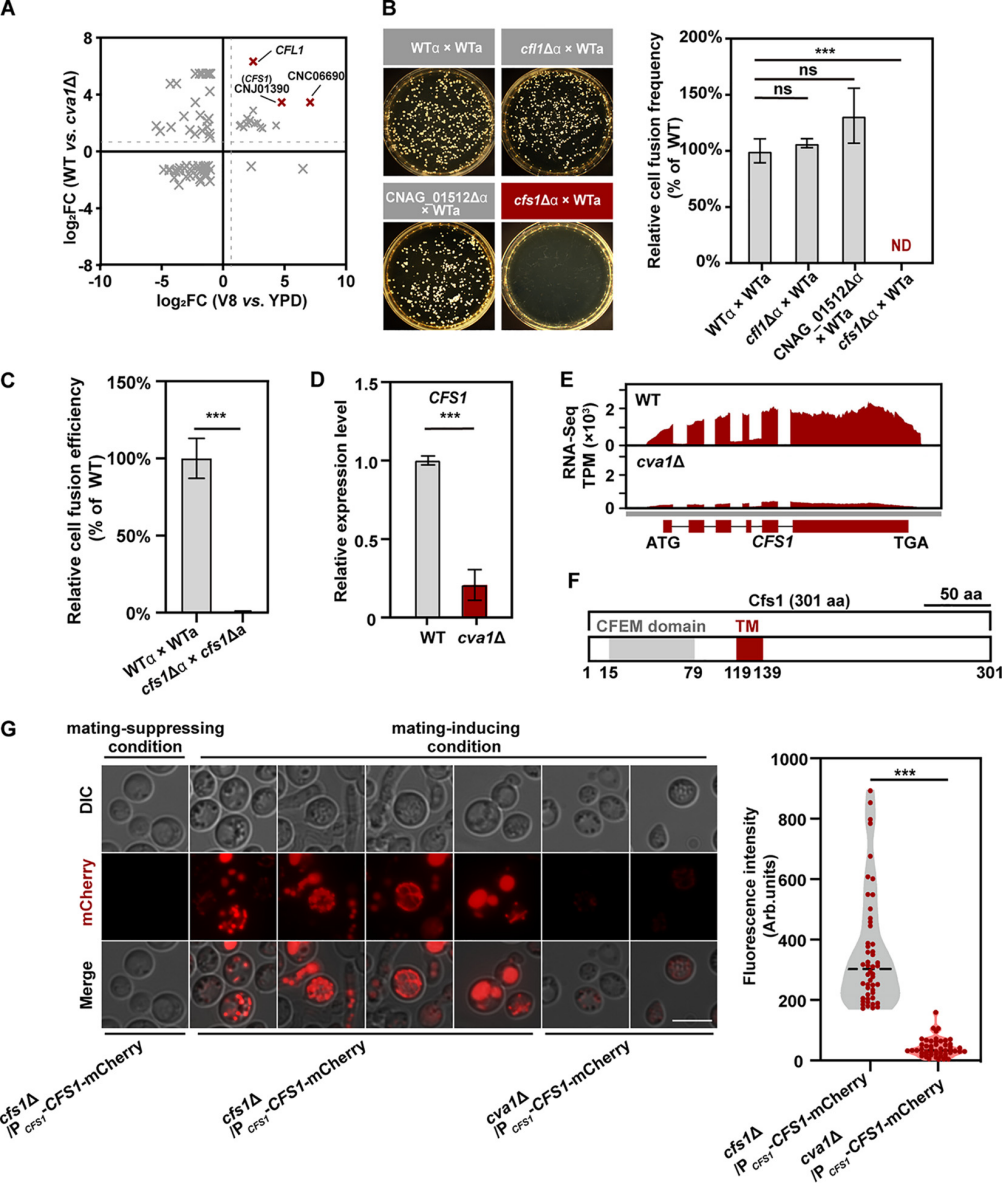
Cfs1, as the key target of Cva1, governs sexual syngamy in C. deneoformans and C. neoformans. (A) The surface/secretory protein-coding genes regulated by mating cue are differentially expressed in *cva1*Δ compared with the wild-type strain (log_2_FC (V8 *versus* YPD) > 1). (B) Cell fusion products of the indicated cross for 48 h on V8 at 25°C in the dark in C. neoformans (left). Unilateral cell fusion frequency of the indicated strains. The data represent the means ± SD (*n* = 3), ND, not detected. ns, not significant (two-tailed Student’s *t* test) (right). (C) Bilateral cell fusion frequency of the indicated strains. Cell fusion products of the indicated cross for 15 h on V8 at 25°C in the dark in C. deneoformans. The data represent the means ± SD (*n* = 3). ***, *P < *0.001; two-tailed Student’s *t* test. (D) qRT-PCR showing the relative levels of *CFS1* mRNA in the *cva1*Δ strain during sexual reproduction compared to wild-type. The data represent the means ± SD (*n* = 3). ***, *P < *0.001 (two-tailed Student’s *t* test). (E) Cva1 controls the transcript levels of *CFS1*, as visualized by the Integrated Genome Browser based on two independent RNA sequence reads. (F) Domain organization of Cfs1. TM, transmembrane; aa, amino acids; CFEM, common in fungal extracellular membrane proteins. (G) Localization and expression of Cfs1-mCherry in different strains under mating-suppressing or mating-inducing conditions. Bar, 5 μm (left). Violin plot analysis shows expression of Cfs1-mCherry in different strains under mating-inducing condition (right). ***, *P < *0.001 (two-tailed Student’s *t* test).

Multiple studies have shown that certain secretory/surface proteins play critical roles in sexual cell-cell fusion in various fungi (51–53). Thus, we hypothesized that there may be cell secretory/surface proteins that are responsible for Cva1-mediated sexual syngamy. To test this idea, we focused on the Cva1-induced genes predicted to encode secretory/surface proteins that were significantly induced in response to mating cues. Our analysis explored 16 genes, with the top three hits identified to be *CFL1*, CNJ01390, and CNC06690 (Fig. 5A). Among these genes, *CFL1* was previously shown to be dispensable for sexual syngamy in C. deneoformans (21). This result was also confirmed by unilateral mating experiments using the *cfl1*Δ strain from a gene deletion library constructed in C. neoformans H99 by the Madhani laboratory (54). In these mating experiments, fungi lacking *CFL1* were found to mate normally (Fig. 5B). Similarly, a strain from the same library in which the homolog of CNC06690 (CNAG_01512) was deleted did not demonstrate a detectable defect in cell fusion efficiency (Fig. 5B).

In contrast to *CFL1* and CNC06690, we observed a complete blocking of mating between wild-type **a** and α strain of C. neoformans in which the orthologue of CNJ01390 was deleted (Fig. 5B). Likewise, CNJ01390 is also essential for α-**a** mating in C. deneoformans, since its absence in XL280 background nearly abolished cell-cell fusion, as revealed by bilateral mating assays (Fig. 5C). Given its key role in sexual cell fusion (Fig. 5C), we named CNJ01390 *CFS1* (Cryptococcus cell-cell fusion secreted-like protein 1). To further confirm the regulation of *CFS1* by Cva1 during sexual reproduction, we performed a qRT-PCR analysis. We found that the level of transcription of *CFS1* was significantly attenuated in the absence of Cva1, in line with our transcriptomic data (Fig. 5D and E). Notably, previous transcriptomic analysis targeting the *mat2*Δ mutant have suggested that the absence of Mat2 likewise reduced the expression of *CFS1* (35). Moreover, the promoter of *CFS1* includes the binding motif of Mat2 reported previously (55). These data suggest that *CFS1* is a key target controlled by the Cva1-Mat2 regulatory circuit during α-**a** bisexual mating.

A bioinformatics analysis using the program TOPCONS led to the prediction that Cfs1 is a transmembrane protein. Pfam domain prediction further revealed that Cfs1 contains a domain called “common in fungal extracellular membrane proteins” (CFEM), which is unique to fungi and is strictly associated with secretory/surface proteins (56) (Fig. 5F). Notably, Cfs1 does not share evident similarity with any well-characterized CFEM proteins, which may reflect the functional distinctiveness of Cfs1.

To examine the temporospatial characteristics of expression of Cfs1 during sexual syngamy, we generated a construct in which the fluorescent protein mCherry is fused with Cfs1 and that is under the control of the native promoter of *CFS1*. In agreement with the previous transcriptomic results showing that *CFS1* was highly induced during sexual reproduction, abundant Cfs1-mCherry was detected upon sex induction, while no detectable fluorescence signal was observed when cultured under mating-suppressing condition (Fig. 5G). Imaging Cfs1-mCherry in XL280α cells cultured on mating-inducing medium revealed that Cfs1 displayed different patterns in its subcellular localizations. Upon mating induction, Cfs1 was localized in vesicles in the majority of Cfs1 expressing yeast and hyphal populations (Fig. 5G). These vesicles are morphologically similar in appearance to known secretory vesicles described in other fungi (57, 58). In addition, Cfs1-mCherry could also be detected around the surface or in vacuoles of some yeast and hyphal cells (Fig. 5G). Furthermore, the absence of Cva1 led to substantially weakened expression of Cfs1-mCherry, in agreement with our qRT-PCR results (Fig. 5D and G). These data collectively demonstrate that the mating-responsive protein Cfs1 is required for Cva1-mediated sexual syngamy.

## DISCUSSION

In C. deneoformans, sexual reproduction is thought to facilitate its infection through multiple routes (27, 30, 32, 59–62). In addition to its involvement in pathogenicity, sexual reproduction also enhances the flexibility and resilience of C. deneoformans in competitive environmental niches (1, 7, 9, 19, 60, 63, 64). For instance, the hyphae produced in the process of sexual reproduction have been documented to assist in the prevention of engulfment by soil amoeba, a natural predator of C. deneoformans (7, 60). Furthermore, sexual sporulation and filamentation confer an ecological benefit by promoting foraging for nutrients and mating partners (63, 64). Sexual reproduction therefore serves as a key adaptation strategy that contributes to fitness in diverse natural niches and in interactions with different hosts. It may therefore not be surprising that C. deneoformans evolved complicated regulatory systems that ensure the proper sexual activation in response to diverse extracellular and physiological stimuli.

Research over the last 30 years has explored multiple signaling pathways and many genes that are involved in sexual reproduction (7, 10, 35, 65–71). However, most of these pathways and genes are not specific for mating regulation but play pleotropic roles in orchestrating a variety of biological processes (65–68, 71). The regulatory specificity of sexual reproduction in C. deneoformans remains poorly understood.

Our data demonstrated that Cva1 specifically activates sexual reproduction. Transcriptomic analysis also indicated a key role for this protein in the synchronous induction of the early mating genes, including the core elements of the mating-MAPK cascade, which serves as the central signaling pathway in both reproduction modes (19, 20, 22). In contrast, Cva1 does not affect the expression of the genes belonging to other well-known pathways that do not contribute to the regulatory specificity of sexual reproduction (48–50). These data suggest that Cva1 serves as a mating-specific activator in C. deneoformans.

The domain prediction demonstrated that Cva1 contains a velvet domain and shares obvious similarities with fungal VelB orthologues. These proteins typically act as pleiotropic regulators involved in multiple functions, such as in the case of the regulation of asexual development and secondary metabolism by VelB of A. nidulans (36, 72). Another example is the product of the *BcVELB* gene from the basidiomycete Botrytis cinerea. This protein has been found to control not only sexual reproduction but also the oxidative stress response and to also affect interactions with a host during infection (73). Unlike these homologues, Cva1 exerts a specific regulation toward mating.

Interestingly, bioinformatics analysis showed that there are six putative velvet protein-coding genes in the genome of C. deneoformans; other fungal species, including A. nidulans and B. cinerea, typically have three or four such genes (74–77). The redundancy of velvet members in C. deneoformans may be related to the “specialist” role of Cva1 in sexual reproduction, which is distinct from its homologues that tend to act as “generalists” that coordinate various biological processes.

Further analysis of the targets of Cva1 indicated that it does not function in the coordination of the multistaged sexual cycle but instead appears to be dedicated to the induction of the early mating events, including sexual syngamy, a hallmark of mating. Interestingly, we identified that the mating-responsive protein Cfs1 is an important target of Cva1. The absence of this protein can nearly abolish sexual syngamy in C. deneoformans and C. neoformans. Cfs1 harbors a CFEM domain that is unique to fungal species (56). In fungi, proteins containing CFEM domain are associated with diverse functions (78–80). However, Cfs1, to our knowledge, represents a previously unknown CFEM protein that is solely dedicated to fungal mating. While Cfs1 has a CFEM domain, the lack of overall similarity of Cfs1 with other CFEM proteins may explain the uniqueness of its functions.

Previous studies have shown that the CFEM domain is involved in Fe^3+^ heme acquisition (81–83). This raises the possibility that iron homeostasis may play a role during Cfs1-mediated sexual syngamy in C. deneoformans. However, our data indicated that supplementation with hemin, which is ferric heme coordinated to chloride, cannot restore the defect of cell fusion caused by the absence of Cfs1 (80) (Fig. S4), suggesting that it may control sexual syngamy independently of heme-iron acquisition. In this regard, the detailed mechanism of action underlying Cfs1-mediated sexual syngamy warrants further investigation, especially considering that it is highly conserved in Cryptococcaeceae, which include several important human fungal pathogens (Fig. S5).

### Compliance and ethics statement.

The mouse experiments were performed according to the guidelines of Regulation of the Institute of Microbiology, Chinese Academy of Sciences of Research Ethics Committee and approved by Regulation of the Institute of Microbiology, Chinese Academy of Sciences of Research Ethics Committee (permit SQIMCAS2020147).

## MATERIALS AND METHODS

### Strains and culture conditions.

The strains described in this study are listed in Table S2. Deletion strains of H99 were obtained from the Fungal Genetics Stock Center. All strains were stored in sample storage tubes containing 20% glycerin. Yeast cells were grown on solid YPD medium (1% yeast extract, 2% peptone, 2% glucose, 2% agar) at 30°C in the dark. Unisexual and bisexual mating assays were performed on V8 juice agar (0.5 g/L KH_2_PO_4_, 5% V8 juice, and 4% Bacto agar, pH 7.0 for C. deneoformans and pH 5.0 for C. neoformans) at 25°C in the dark as described previously (6, 53). For the GlcN-induced filamentation assay, 2% galactose was added to the YPGlcN base medium (1% yeast extract, 2% peptone, 2% GlcN and 2% Bacto agar), and the cells were incubated at 25°C in the dark for 3 days, as described previously (41, 42).

### Gene disruption and complementation.

Gene disruption was performed as previously described (84). Briefly, 5′ and the 3′ homologous arms of the target gene were amplified by PCR and fused with a G418 or nourseothricin (NAT) resistance marker to generate a gene deletion cassette. Then, the deletion cassette was introduced into C. deneoformans strains through the TRACE method (85). All mutants were confirmed by PCR. To generate a strain in which the *cva1*Δ mutation was complemented with *CVA1*, a plasmid containing the coding region of *CVA1* under the control of its native promoter was constructed and linearized. The linearized plasmid was then introduced into the indicated strains by electroporation. The primers used in this study are listed in Table S3.

### Analysis of susceptibility to chemicals and antifungal agents.

Susceptibility analyses to the presence of antifungal drugs and other stressors were performed as previously described (86). Each strain was incubated at 30°C in YPD medium for 12 h and diluted to an optical density at 600 nm (OD_600_) of 1.0 in distilled water (dH_2_O), whereupon 5-fold dilutions were created for subsequent use. To analyze growth phenotypes at distinct temperatures, the cells were spotted on solid YPD medium and monitored at a range of temperatures (4, 25, 30, 37, and 39°C). To analyze susceptibility to stress, the prepared cells were spotted on YP or YPD medium containing one of the following chemicals: 0.015% hydrogen peroxide, 100 mM hydroxyurea, 3 mM *tert*-Butyl hydroperoxide, 3 mM diamide, and 30 μM menadione to induce oxidative stress; 2 M KCl and 2 M NaCl to induce salt stress; 2 M sorbitol to induce osmotic stress; 0.05% SDS and 3% Congo red to induce cell membrane/cell wall stress; 15 mM dithiothreitol to induce endoplasmic reticulum stress; and 0.75 μg/mL amphotericin B, 10 μg/mL 5-flucytosine, 20 μg/mL fluconazole, and 250 mg/mL rapamycin to induce antifungal stress.

### Virulence assays.

Wild-type and *cva1*Δ strains were grown in YPD liquid medium for 12 h at 30°C. The cells were then resuspended in phosphate-buffered saline (PBS) at a final concentration of 2 × 10^6^ CFU mL^−1^. Groups of five 7-week-old female C57 BL/6 mice were infected with 2 × 10^6^ cells (in 50 μL) of the C. deneoformans strain for survival tests using the well-established intravenous infection models as described previously (38, 87). To examine the fungal burden in the wild-type and *cva1*Δ strains, the lungs, brains, livers, and kidneys from three infected mice were dissected 14 days postinfection. The tissue suspensions were diluted, plated onto YPD plates, and incubated for 3 days at 30°C in the dark.

### Filamentation, basidial maturation score, sporulation, and basidiospore germination assays.

All strains were grown on YPD solid medium for 12 h at 30°C in the dark and then resuspended in sterile water. Filamentation assays was performed as previously described (8). Briefly, *MAT*α cells were spotted onto V8 agar alone and incubated in the dark at 25°C for the observation of unisexual hyphae. For bisexual hyphae, equal numbers (OD_600_ = 1.0) of congenic α and **a** cells were mixed, spotted onto V8 agar, and incubated at 25°C in the dark for 3 days.

Basidial maturation score assays were conducted as previously described (23). Briefly, cells on the edge of mating patches were harvested in formalin and dropped onto a glass slide for examination via a Zeiss Imager A2-M2 imaging system with AxioCam MRm camera software Zen 2011 (Carl Zeiss Microscopy). Fifty hyphae, with or without basidia, were randomly chosen from each sample for calculation of the basidial maturation score. Unisexual mating phenotypes were examined microscopically for chains of basidiospores as described previously. To evaluate basidiospore germination, basidiospores were dissected on YPD medium using a SporePlay dissection microscope (Singer Instruments), and the dissected spores were incubated at 30°C for 3 to 5 days until colonies were formed, as previously described (88).

### Cell-cell fusion assay.

Cell-cell fusion assays were performed as previously described (53). Briefly, strains for each fusion pair were cultured on YPD agar for 12 h at 30°C. The cells were suspended and washed twice with sterile water. Then, equal numbers (OD_600_ = 1.0) of congenic α and **a** cells were mixed, spotted onto V8 juice agar, and incubated at 25°C in the dark for 15 h for C. deneoformans bisexual reproduction or 48 h for C. neoformans bisexual reproduction. The cells were then removed and plated on YPD agar supplemented with both NAT and G418 or both hygromycin and G418 for 3 to 5 days.

### Microscopy and fluorescence.

To examine the expression and localization of Cfs1 under mating-suppressing or mating-inducing condition, cells from different strains harboring P*_CFS1_*-*CFS1*-*mCherry* were cultured on YPD or V8 agar for 24 h. To investigate the effect of Cva1 on the expression of Dmc1, the indicated strains harboring P*_DMC1_-DMC1-mCherry* were grown on V8 juice agar for 7 days. Localization of Cva1-mCherry during unisexual reproduction was observed on V8 agar 24 h after mating induction. The images were captured with a Zeiss Axioplan two imaging system with the AxioCam MRm camera software Zen 2011 (Carl Zeiss Microscopy).

### RNA extraction and qRT-PCR.

RNA extraction and qRT-PCR were performed as previously described (89). Briefly, the wild-type and the *cva1*Δ strains of C. deneoformans were cultured in YPD liquid medium at 30°C for 16 h. Then, the cells were washed with water and spotted onto V8 juice medium (pH 7.0) for 24 h for the isolation of total RNA. Total RNA was extracted using Ultrapure RNA kit (Kangweishiji, CW0581S) according to the manufacturer’s instructions. The Fastquant reverse transcription (RT) kit (Tiangen KR106-02, with gDNase) and Power SYBR qPCR premix reagents (KAPA) were used for reverse transcription and quantitative real-time PCR, respectively. Two biological replicates and two technical replicates were performed for each sample. Gene expression levels were normalized to the expression of the endogenous reference gene *TEF1* and were determined using the comparative *C_t_* (threshold cycle) method. The primers used for qRT-PCR are listed in Table S3.

### RNA-seq and data analysis.

RNA-seq was performed as described previously (6). Briefly, the VAHTS mRNA-seq version 2 library prep kit (Vazyme Biotech Co., Ltd., Nanjing, China) was used to generate transcriptome libraries following the manufacturer’s protocols. The samples were clustered and sequenced using VAHRS RNA adapters set 1/set 2 and Illumina Hiseq 4000 platform, respectively. StringTie version 1.3.3 was used to measure the gene expression level in transcripts per million (TPM), and DEseq2 version 1.16.1 Bioconductor package was used to assess the differential expression of genes. On average, over 6 million filtered and aligned reads were generated for each sample, which covered 99.2% of the protein-coding genes in the XL280 genome. The significant differential expression of genes regulated by Cva1 was defined based on the fold change criterion (|log_2_(fold change)| > 1). GraphPad Prism 8.0.1 was used to generate the heat maps. The gene list was uploaded into the PANTHER database to generate the enriched GO terms.

### Data availability.

The transcriptome sequencing data have been deposited in the Gene Expression Omnibus under the accession number GSE196267.
