# Supplementary material for: A Velvet Transcription Factor Specifically Activates Mating through a Novel Mating-Responsive Protein in the Human Fungal Pathogen Cryptococcus deneoformans
Source: Microbiol Spectr. 2022 Apr 26;10(3):e02653-21. doi: 10.1128/spectrum.02653-21 (PMC9241590; doi:10.1128/spectrum.02653-21)
Supplement: SUPPLEMENTAL FILE 1 — Supplemental material. Download spectrum.02653-21-s001.pdf, PDF file, 3.0 MB [file spectrum.02653-21-s001.pdf]

**Supplementary figure legends**

**Figures S1-S5**

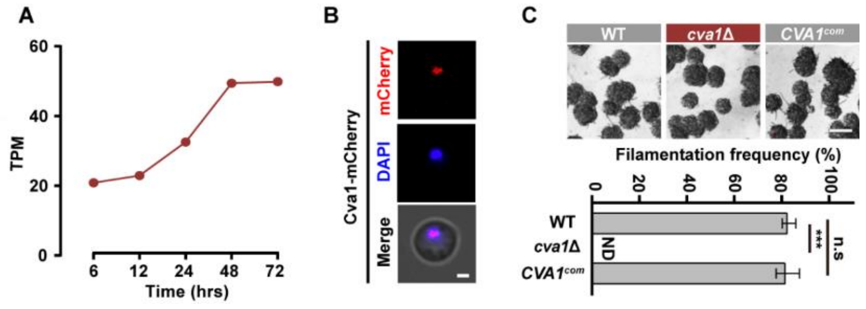


**Figure S1. CNA05460 displays a highly dynamic expression during the unisexual cycle and controls unisexual filamentation in *C. deneoformans*.** (A) Time course of expression of CNA05460 mRNA expression, as transcripts per million (TPM), under mating-inducing condition. (B) Expression and localization of Cva1-mCherry during unisexual reproduction. DAPI, 4′,6-diamidino-2-phenylindole. Scale bar, 1 μm. (C) Quantitative evaluation of filamentation frequency of different XL280 α strains. The filamentous mini-colony morphology in various mutants for 22 h at 25°C after induction of mating in the dark. Data are presented as the mean ± SD (n = 3). ND, not detected. ns, not significant (two-tailed Student's *t*-test). Scale bar, 100 μm.

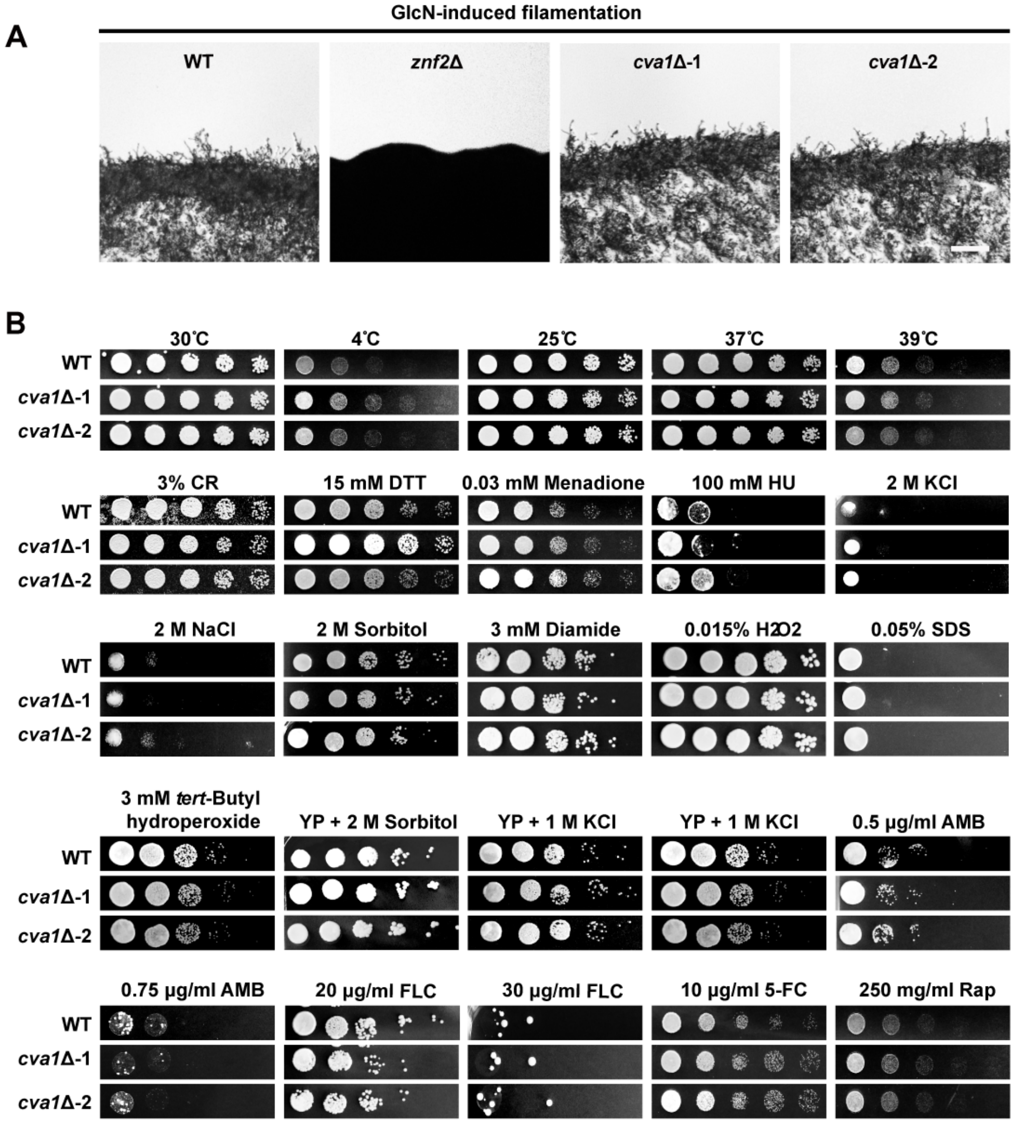


**Figure S2. The absence of Cva1 does not cause remarkable changes in *C. deneoformans* during GlcN-induced filamentation or under various stress conditions.** (A) Images of wild-type and the *cva1*Δ strains at the colony level when cultured on GlcN agar for 3 days at 25°C in the dark. Scale bar, 100 μm. (B) Strains were grown overnight on YPD agar at 30°C in the dark, then spotted onto YPD or YP agar containing the following reagents or antifungal drugs: sorbitol, NaCl, KCl, hydrogen peroxide, *tert*-Butyl hydroperoxide, menadione, diamide, hydroxyurea (HU),

SDS, Congo red (CR), DTT, amphotericin B (AMB), fluconazole (FLC), 5-flucytosine (5-FC), or rapamycin (Rap).

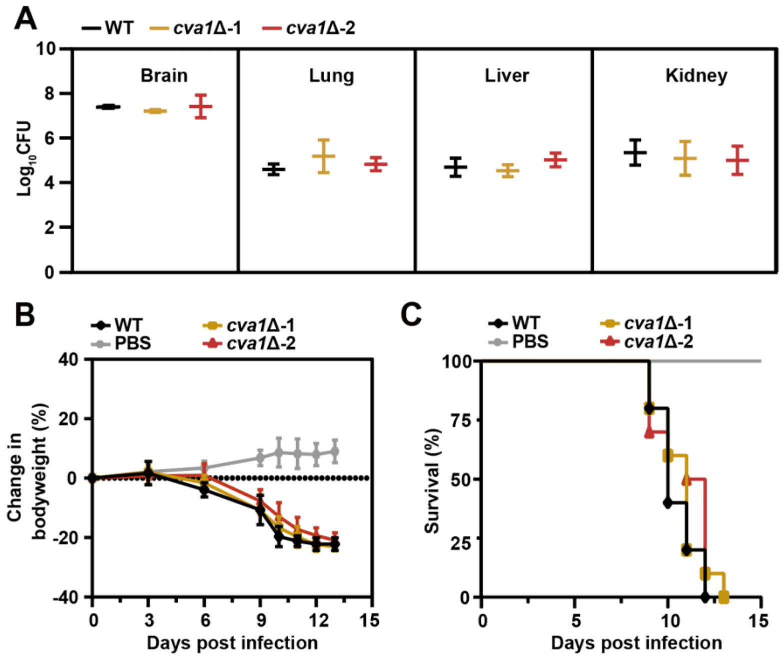


**Figure S3. Cva1 is dispensable for pathogenicity in *C. deneoformans*.** (A) Fungal burden in the lungs, brains, livers and kidneys of mice infected with wild-type or mutant fungi was evaluated 14 days after infection. (B) Body weight changes of mice infected through intravenous injection were monitored at different time points. (C) Survival rates of infected mice were monitored for 13 days.

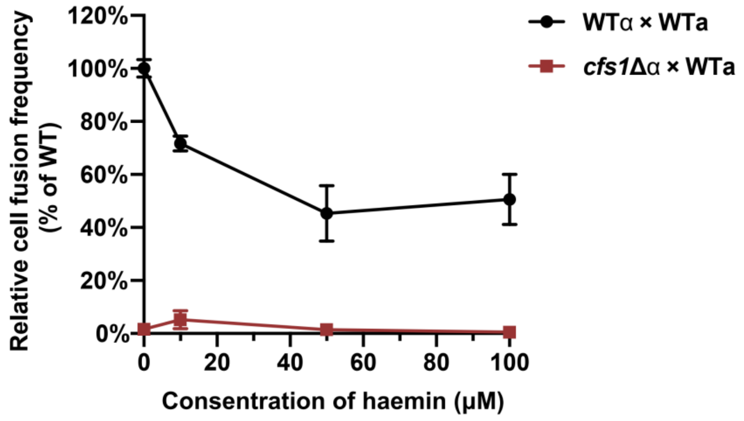


**Figure S4. The effect of haemin on bisexual cell fusion in the presence or absence of Cfs1.** Unilateral cell fusion frequency of the indicated strains for 15h on V8 medium containing haemin at different concentrations at 25°C in the dark. Data shown are from two independent experiments.

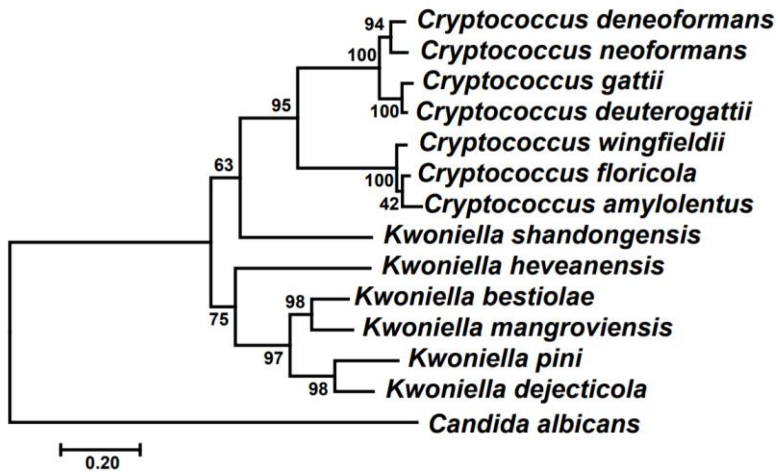


**Figure S5. Cfs1 is conserved in the species belonging to Cryptococcaeceae.** The protein sequences were aligned using MEGA version 7.0 by the neighbor-joining method.

## Table S1: Genes expressed in the *cva1* Δ mutant during unisexual mating

| Gene ID | Homolog in JEC2 | *cva1* Δ(V8) vs WT(V8) | $P_{adj}$ | annotation |
|---|---|---|---|---|
| CNXL_000010 | CNA00010 | 0 | NA | annotation |
| CNXL_000020 | CNA00020 | 0.509905703 | 0.113628393 | hypothetical protein |
| CNXL_000030 | CNA00030 | -0.248780292 | 0.632998683 | hypothetical protein |
| CNXL_000040 | CNA00040 | 0.251216041 | 0.654994028 | cation transporter |
| CNXL_000050 | CNA00050 | -0.039586659 | 0.963266917 | calcium-binding protein |
| CNXL_000060 | CNA00060 | -0.270533417 | 0.623549135 | hypothetical protein |
| CNXL_000070 | CNA00070 | 0.293127118 | 0.480684646 | methionine-tRNA ligase |
| CNXL_000080 | | -0.131389572 | 0.864719008 | prohibitin-2 |
| CNXL_000090 | CNA00080 | 1.212140501 | 0.001928327 | hypothetical protein |
| CNXL_000100 | CNA00100 | -0.310638828 | 0.50345637 | hypothetical protein |
| CNXL_000110 | | 0.304342983 | 0.437062442 | hypothetical protein |
| CNXL_000120 | CNA00110 | 0.210088454 | 0.671905355 | U6 snRNA-associated Sm-like protein LSm5 |
| CNXL_000130 | CNA00120 | -0.129444912 | 0.805451003 | hypothetical protein |
| CNXL_000140 | CNA00130 | -0.455493114 | 0.327311741 | hypothetical protein |
| CNXL_000150 | CNA00140 | -0.432866931 | 0.303744123 | hypothetical protein |
| CNXL_000160 | CNA00150 | 0.023990986 | 0.981240834 | S-adenosylmethionine decarboxylase proenzyme |
| CNXL_000170 | CNA00160 | 0.397915204 | 0.312497815 | cytochrome c heme-lyase |
| CNXL_000180 | CNA00170 | -0.135617283 | 0.79546523 | hypothetical protein |
| CNXL_000190 | CNA00180 | -0.67182561 | 0.063702758 | hypothetical protein |
| CNXL_000200 | CNA00190 | -0.109121172 | 0.861760083 | vacuolar calcium exchanger |
| CNXL_000210 | CNA00200 | 0.239110851 | 0.637704309 | kynurenine aminotransferase |
| CNXL_000220 | CNA00210 | 0.246599293 | 0.637704309 | transcriptional activator |
| CNXL_000230 | CNA00220 | 0.44923742 | 0.647364118 | high-affinity nicotinic acid transporter |
| CNXL_000240 | | 0.258866531 | 0.683642142 | D-3-phosphoglycerate dehydrogenase |
| CNXL_000250 | CNA00230 | 0.165313771 | 0.814730279 | D-3-phosphoglycerate dehydrogenase |
| CNXL_000260 | CNA00240 | 0.668348959 | 0.096975702 | hypothetical protein |
| CNXL_000270 | CNA00250 | -0.186204276 | 0.670494322 | 2 |
| CNXL_000280 | CNA00260 | -2.280425438 | 4.19782E-14 | large subunit ribosomal protein L9 |
| CNXL_000290 | | 0.977742358 | 0.002053592 | hypothetical protein |
| CNXL_000300 | CNA00270 | 1.056208772 | 0.001080531 | hypothetical protein |
| CNXL_000310 | CNA00280 | 0.246146307 | 0.584015643 | phosphatidylinositol |
| CNXL_000320 | CNA00290 | -0.454167216 | 0.290975028 | alcohol dehydrogenase |
| CNXL_000330 | CNA00300 | -0.491851032 | 0.225918409 | hypothetical protein |
| CNXL_000340 | CNA00310 | 0.80316025 | 0.008581942 | lanosterol 14-alpha-demethylase |
| CNXL_000350 | CNA00320 | 0.824253797 | 0.005117686 | hypothetical protein |
| CNXL_000360 | CNA00330 | 0.575738644 | 0.120635533 | hypothetical protein |
| CNXL_000370 | CNA00340 | 0.060001164 | 0.939400455 | ribosome assembly protein 1 |
| CNXL_000380 | CNA00350 | 0.086682327 | 0.889191243 | hypothetical protein |
| CNXL_000390 | CNA00360 | -0.173948975 | 0.746537956 | 26S proteasome regulatory subunit N8 |
| CNXL_000400 | CNA00370 | 0.148332258 | 0.802348605 | pyruvate dehydrogenase kinase |
| CNXL_000410 | CNA00380 | 0.29824892 | 0.460815133 | sugar transporter |
| CNXL_000420 | CNA00390 | -0.114931673 | 0.889001755 | hypothetical protein |
| CNXL_000430 | | 0.630348885 | 0.031829912 | hypothetical protein |
| CNXL_000440 | CNA00410 | 1.783824274 | 0.123590227 | hypothetical protein |
| CNXL_000450 | CNA00420 | 0.689543085 | 0.065918831 | hypothetical protein |
| CNXL_000460 | CNA00430 | 0.409880366 | 0.542233393 | hypothetical protein |
| CNXL_000470 | CNA00450 | 0.471434812 | 0.18112051 | hypothetical protein |
| CNXL_000480 | CNA00460 | 0.447324055 | 0.295034424 | hypothetical protein |
| CNXL_000490 | CNA00470 | 0.261644253 | 0.586181879 | hypothetical protein |
| CNXL_000500 | CNA00480 | 0.030927963 | 0.964414864 | fructose-1 |
| CNXL_000510 | | -0.074473832 | 0.924607253 | T-complex protein 1 subunit epsilon |
| CNXL_000520 | CNA00490 | 0.07836514 | 0.925523772 | hypothetical protein |
| CNXL_000530 | CNA00510 | -0.18734098 | 0.781467115 | chaperone regulator |
| CNXL_000540 | CNA00520 | -0.186609831 | 0.70913058 | citrate synthase |
| CNXL_000550 | CNA00540 | 0.043922153 | 0.957098418 | transcription elongation regulator 1 |
| CNXL_000560 | CNA00550 | 0.036857408 | 0.965966884 | histone H3 |

| | | | | |
|---|---|---|---|---|
| CNXL_000570 | CNA00560 | -0.466348615 | 0.260431503 | COPII-coated vesicle protein |
| CNXL_000580 | CNA00570 | -0.308057702 | 0.433191867 | ran-binding protein 1 |
| CNXL_000590 | CNA00580 | -0.16075956 | 0.771763303 | membrane protein |
| CNXL_000600 | CNA00590 | -0.272606163 | 0.634728039 | sec61 translocation complex protein |
| CNXL_000610 | CNA00600 | 0.110265321 | 0.965004754 | specific RNA polymerase II transcription factor |
| CNXL_000620 | CNA00610 | -0.241282683 | 0.700912881 | hypothetical protein |
| CNXL_000630 | CNA00620 | 0.349306998 | 0.431497115 | hypothetical protein |
| CNXL_000640 | CNA00630 | 0.022196883 | 0.977457668 | protein disulfide-isomerase |
| CNXL_000650 | CNA00640 | 0.889019127 | 0.020908076 | nuclear mRNA splicing protein |
| CNXL_000660 | CNA00650 | 1.277859555 | 0.000504047 | protein PNS1 |
| CNXL_000670 | CNA00660 | 0.746497658 | 0.061154988 | hypothetical protein |
| CNXL_000680 | CNA00670 | -0.057788523 | 0.929378909 | A/G-specific adenine glycosylase |
| CNXL_000690 | CNA00680 | -0.30070329 | 0.43363696 | solute carrier family 26 |
| CNXL_000700 | CNA00690 | -0.631664361 | 0.028632479 | vacuolar protein |
| CNXL_000710 | CNA00700 | -0.086854748 | 0.92224659 | hypothetical protein |
| CNXL_000720 | CNA00710 | -0.079861949 | 0.892423276 | hypothetical protein |
| CNXL_000730 | CNA00720 | 0.269610466 | 0.641029652 | pre-mRNA-processing factor 6 |
| CNXL_000740 | CNA00730 | 0.039059786 | 0.958962395 | cytosolic Fe-S cluster assembly factor CFD1 |
| CNXL_000750 | CNA00740 | -0.353929474 | 0.43363696 | hypothetical protein |
| CNXL_000760 | CNA00750 | -0.130126676 | 0.879531297 | glutamine-tRNA ligase |
| CNXL_000770 | CNA00760 | -0.273218379 | 0.58959601 | histone chaperone ASF1 |
| CNXL_000780 | | -0.36638994 | 0.654601802 | hypothetical protein |
| CNXL_000790 | CNA00770 | 0.712909958 | 0.012197829 | hypothetical protein |
| CNXL_000800 | CNA00780 | 0.163487176 | 0.766412687 | hypothetical protein |
| CNXL_000810 | CNA00790 | -0.072186836 | 0.919196787 | prohibitin PHB1 |
| CNXL_000820 | CNA00800 | 0.233104171 | 0.615328522 | exosome complex component RRP4 |
| CNXL_000830 | CNA00820 | 2.358342273 | 0.061215984 | hypothetical protein |
| CNXL_000840 | CNA00830 | -0.101510776 | 0.847311574 | hypothetical protein |
| CNXL_000850 | CNA00840 | 0.956698372 | 0.024690962 | mitochondrial protein |
| CNXL_000860 | CNA00850 | -0.253661019 | 0.592413305 | hypothetical protein |
| CNXL_000870 | CNA00860 | -0.301344923 | 0.593178899 | NAD-dependent epimerase/dehydratase |
| CNXL_000880 | CNA00870 | 0.127408512 | 0.874890451 | hypothetical protein |
| CNXL_000890 | CNA00880 | 0.061921196 | 0.928564114 | hypothetical protein |
| CNXL_000900 | CNA00890 | -0.024920161 | 0.983499434 | myo-inositol transporter |
| CNXL_000910 | CNA00900 | -1.118997711 | 0.000681137 | palmitoyl-protein thioesterase |
| CNXL_000920 | CNA00910 | 0.290372694 | 0.482812185 | minichromosome maintenance protein 3 |
| CNXL_000930 | CNA00920 | -0.251541314 | 0.634728039 | stress-induced-phosphoprotein 1 |
| CNXL_000940 | CNA00930 | 0.629172742 | 0.032666864 | hypothetical protein |
| CNXL_000950 | CNA00940 | 0.339515401 | 0.357542656 | mRNA surveillance protein pelota |
| CNXL_000960 | CNA00950 | -0.477018479 | 0.242541001 | hypothetical protein |
| CNXL_000970 | CNA00960 | 0.505828492 | 0.421588467 | elongation factor 3 |
| CNXL_000980 | CNA00970 | -0.978591099 | 0.00232079 | pyridoxal reductase |
| CNXL_000990 | CNA00980 | 0.65578233 | 0.064192122 | two-component-like sensor kinase |
| CNXL_001000 | CNA00990 | 0.010443232 | 0.990777629 | hypothetical protein |
| CNXL_001010 | CNA01000 | 0.109217313 | 0.865015851 | RuvB-like helicase 1 |
| CNXL_001020 | CNA01010 | -1.558485599 | 0.036409011 | large subunit ribosomal protein L34 |
| CNXL_001030 | CNA01020 | -0.019588616 | 0.981851244 | hypothetical protein |
| CNXL_001040 | CNA01030 | -0.320543524 | 0.722427823 | splicing factor 3A subunit 1 |
| CNXL_001050 | CNA01040 | 0.537002026 | 0.173210496 | Set1/Ash2 histone methyltransferase complex subunit |
| CNXL_001060 | CNA01050 | -0.289740441 | 0.473529666 | hypothetical protein |
| CNXL_001070 | CNA01060 | -0.264062863 | 0.512043294 | chlorophyll synthesis pathway protein BchC |
| CNXL_001080 | CNA01070 | 0.091901222 | 0.882149483 | small subunit ribosomal protein S3 |
| CNXL_001090 | CNA01080 | -0.022633804 | 0.982102702 | C-14 sterol reductase |
| CNXL_001100 | CNA01090 | -0.015048821 | 0.988873975 | hypothetical protein |
| CNXL_001110 | | -0.111202275 | 0.871700589 | hypothetical protein |
| CNXL_001120 | CNA01100 | 0.437838493 | 0.203714539 | hypothetical protein |
| CNXL_001130 | | 0.285635139 | 0.640550491 | glycerol-3-phosphate dehydrogenase |
| CNXL_001140 | CNA01120 | -3.284331013 | 1.1526E-32 | hypothetical protein |

| | | | | |
|---|---|---|---|---|
| CNXL_001150 | CNA01130 | 0.650198396 | 0.072401784 | alpha-amylase |
| CNXL_001160 | CNA01140 | -0.201020841 | 0.710705655 | hypothetical protein |
| CNXL_001170 | CNA01150 | -2.569041749 | 2.16305E-31 | capsule structure designer protein |
| CNXL_001180 | CNA01160 | -0.372246514 | 0.336943807 | regulator of G-protein signaling |
| CNXL_001190 | CNA01170 | 0.143727788 | 0.799344206 | 2-deoxy-D-gluconate 3-dehydrogenase |
| CNXL_001200 | CNA01180 | 0.527526192 | 0.143054301 | hypothetical protein |
| CNXL_001210 | | -0.029248714 | 0.965966884 | hypothetical protein |
| CNXL_001220 | CNA01200 | -0.368556659 | 0.365658793 | alcohol dehydrogenase |
| CNXL_001230 | | 0.156944202 | 0.806051382 | hypothetical protein |
| CNXL_001240 | CNA01210 | 0.300877502 | 0.500910612 | hypothetical protein |
| CNXL_001250 | CNA01220 | 0.251252623 | 0.599348746 | hypothetical protein |
| CNXL_001260 | CNA01230 | 0.396419879 | 0.25888418 | cyclophilin |
| CNXL_001270 | CNA01250 | -0.011298221 | 0.990008547 | ubiquitin-activating enzyme E1 |
| CNXL_001280 | CNA01260 | 1.16593164 | 0.000431951 | ubiquinone biosynthesis methyltransferase |
| CNXL_001290 | CNA01270 | -0.288897724 | 0.532490033 | hypothetical protein |
| CNXL_001300 | | -0.13356863 | 0.868398587 | U1 small nuclear ribonucleoprotein 70kDa |
| CNXL_001310 | | 0.101712926 | 0.91726998 | hypothetical protein |
| CNXL_001320 | CNA01290 | -0.1828187 | 0.754127945 | hypothetical protein |
| CNXL_001330 | CNA01310 | -0.571239528 | 0.185202544 | hypothetical protein |
| CNXL_001340 | CNA01320 | 0.017589402 | 0.986881191 | hypothetical protein |
| CNXL_001350 | CNA01330 | -0.825017526 | 0.014736206 | hypothetical protein |
| CNXL_001360 | CNA01340 | 0.143227701 | 0.8469385 | actin-2 |
| CNXL_001370 | CNA01350 | -0.0614512 | 0.925523772 | 5'/3'-nucleotidase SurE |
| CNXL_001380 | CNA01360 | -0.155808545 | 0.78976708 | pre-mRNA-processing-splicing factor 8 |
| CNXL_001390 | CNA01370 | 0.39959589 | 0.227069466 | hypothetical protein |
| CNXL_001400 | CNA01380 | -0.202031981 | 0.645032535 | NADH dehydrogenase |
| CNXL_001410 | | -0.568081127 | 0.293493438 | peptidase |
| CNXL_001420 | CNA01400 | -0.00097691 | 0.998007755 | hypothetical protein |
| CNXL_001430 | CNA01410 | -0.422978882 | 0.61517823 | hypothetical protein |
| CNXL_001440 | CNA01420 | 0.795015349 | 0.003196773 | hypothetical protein |
| CNXL_001450 | CNA01440 | 0.769439034 | 0.065805314 | oxidoreductase |
| CNXL_001460 | CNA01450 | -0.721461264 | 0.019899342 | hypothetical protein |
| CNXL_001470 | CNA01460 | -0.422466156 | 0.193686503 | zinc finger transcription factor |
| CNXL_001480 | CNA01470 | -0.894193891 | 0.000570846 | hypothetical protein |
| CNXL_001490 | CNA01480 | -0.283120128 | 0.510006134 | glycosyltransferase |
| CNXL_001500 | CNA01500 | -0.280504104 | 0.437062442 | hypothetical protein |
| CNXL_001510 | CNA01510 | 0.358478247 | 0.319872213 | hypothetical protein |
| CNXL_001520 | CNA01520 | 0.847929013 | 0.005798111 | hypothetical protein |
| CNXL_001530 | CNA01530 | 0.124681459 | 0.810224824 | hypothetical protein |
| CNXL_001540 | CNA01540 | 0.086688018 | 0.891760995 | methylthioadenosine phosphorylase |
| CNXL_001550 | CNA01550 | -0.017854858 | 0.985173026 | ATP-dependent RNA helicase DBP8 |
| CNXL_001560 | CNA01580 | -0.086907967 | 0.879531297 | hypothetical protein |
| CNXL_001570 | CNA01590 | 0.431296868 | 0.328633829 | hypothetical protein |
| CNXL_001580 | CNA01600 | -0.476411381 | 0.534266327 | peroxin-2 |
| CNXL_001590 | CNA01610 | 0.152744642 | 0.781467115 | kinesin |
| CNXL_001600 | CNA01620 | -0.345089978 | 0.541691065 | hypothetical protein |
| CNXL_001610 | | -0.948879231 | 0.217267964 | hypothetical protein |
| CNXL_001620 | CNA01630 | -0.69799818 | 0.137357068 | hypothetical protein |
| CNXL_001630 | CNA01640 | -0.023898063 | 0.972727243 | hypothetical protein |
| CNXL_001640 | CNA01650 | -1.631813468 | 0.001233389 | glutamate carboxypeptidase |
| CNXL_001650 | CNA01660 | -0.35421534 | 0.491312112 | hypothetical protein |
| CNXL_001660 | CNA01680 | -3.14393155 | 1.92136E-50 | DNA repair protein REV1 |
| CNXL_001670 | CNA01690 | 0.96056428 | 0.001095672 | hypothetical protein |
| CNXL_001680 | CNA01700 | 0.384324448 | 0.346301684 | ubiquitin carboxyl-terminal hydrolase L3 |
| CNXL_001690 | CNA01710 | 0.216730707 | 0.634728039 | hypothetical protein |
| CNXL_001700 | CNA01720 | -0.219611292 | 0.626594366 | hypothetical protein |
| CNXL_001710 | CNA01730 | -0.011471675 | 0.990008547 | hypothetical protein |
| CNXL_001720 | CNA01740 | -0.22746682 | 0.606699873 | pre-mRNA cleavage complex 2 protein Pcf11 |

| | | | | |
|---|---|---|---|---|
| CNXL_001730 | CNA01750 | -0.053866544 | 0.942099307 | hypothetical protein |
| CNXL_001740 | CNA01760 | 0.179043806 | 0.730212038 | mitotic spindle organizing protein 1 |
| CNXL_001750 | CNA01770 | -0.021440327 | 0.982102702 | ubiquitin carboxyl-terminal hydrolase |
| CNXL_001760 | CNA01780 | 0.160238758 | 0.779355929 | endoplasmic reticulum protein |
| CNXL_001770 | CNA01790 | 0.467847254 | 0.170587304 | hypothetical protein |
| CNXL_001780 | CNA01800 | -0.318711234 | 0.566468698 | hypothetical protein |
| CNXL_001790 | | -1.188237636 | 0.000635796 | etoposide-induced protein |
| CNXL_001800 | CNA01820 | -0.416719914 | 0.28126755 | hypothetical protein |
| CNXL_001810 | CNA01830 | 0.21353045 | 0.613465435 | gata type zinc finger protein |
| CNXL_001820 | CNA01840 | 0.082177385 | 0.885449489 | hypothetical protein |
| CNXL_001830 | CNA01850 | 0.023113142 | 0.975380213 | exosome complex protein LRP1 |
| CNXL_001840 | CNA01860 | 0.883764024 | 0.004361028 | hydroxyacid-oxoacid transhydrogenase |
| CNXL_001850 | CNA01870 | 0.153310587 | 0.773509113 | hypothetical protein |
| CNXL_001860 | CNA01880 | 1.335547247 | 0.000535754 | AP-1 complex subunit gamma-1 |
| CNXL_001870 | CNA01890 | 0.595728855 | 0.149676606 | hypothetical protein |
| CNXL_001880 | | 0.516437687 | 0.298641736 | hypothetical protein |
| CNXL_001890 | CNA01900 | 0.175417645 | 0.797485767 | hypothetical protein |
| CNXL_001900 | CNA01910 | -0.423338161 | 0.413608864 | hypothetical protein |
| CNXL_001910 | CNA01920 | 0.108873496 | 0.862655799 | hypothetical protein |
| CNXL_001920 | CNA01930 | -0.044523769 | 0.952399638 | cactin |
| CNXL_001930 | CNA01940 | 0.486600336 | 0.148131392 | hypothetical protein |
| CNXL_001940 | CNA01950 | 0.019525624 | 0.981240834 | hypothetical protein |
| CNXL_001950 | CNA01960 | -0.365289998 | 0.332332744 | hypothetical protein |
| CNXL_001960 | CNA01990 | -0.108446901 | 0.841354437 | hypothetical protein |
| CNXL_001970 | CNA02000 | -0.396009967 | 0.556162827 | hypothetical protein |
| CNXL_001980 | CNA02010 | -1.172945881 | 0.112661106 | hypothetical protein |
| CNXL_001990 | | 0.211073237 | 0.784304932 | hypothetical protein |
| CNXL_002000 | CNA02020 | -0.117746854 | 0.870051341 | hypothetical protein |
| CNXL_002010 | CNA02030 | -0.16490086 | 0.785269877 | hypothetical protein |
| CNXL_002020 | CNA02040 | -0.431673091 | 0.224117965 | ubiquitin carboxyl-terminal hydrolase 25/28 |
| CNXL_002030 | CNA02050 | -0.666757537 | 0.071408618 | ferric reductase |
| CNXL_002040 | | 0.210146161 | 0.737538678 | cystinosin |
| CNXL_002050 | CNA02060 | 0.005252482 | 0.995735232 | hypothetical protein |
| CNXL_002060 | | -0.231878865 | 0.749039348 | hypothetical protein |
| CNXL_002070 | CNA02080 | 0.240863267 | 0.681029948 | hypothetical protein |
| CNXL_002080 | CNA02090 | 0.159155488 | 0.781894338 | hypothetical protein |
| CNXL_002090 | CNA02100 | 0.156304131 | 0.764330585 | vesicle-associated membrane protein 7 |
| CNXL_002100 | | 0.448067568 | 0.167054658 | 26s proteasome lid component |
| CNXL_002110 | CNA02120 | -0.082209525 | 0.900330972 | hypothetical protein |
| CNXL_002120 | CNA02130 | 0.151161578 | 0.814480545 | hypothetical protein |
| CNXL_002130 | CNA02140 | 1.298097935 | 4.92277E-06 | protein SPT2 |
| CNXL_002140 | CNA02150 | 0.71539828 | 0.021196786 | hypothetical protein |
| CNXL_002150 | | -0.042045445 | 0.962194201 | hypothetical protein |
| CNXL_002160 | CNA02170 | -0.1436325 | 0.777403833 | hypothetical protein |
| CNXL_002170 | CNA02180 | 0.090997051 | 0.880060691 | hypothetical protein |
| CNXL_002180 | | 0.508025811 | 0.516659115 | cytoplasmic protein |
| CNXL_002190 | | 0.366286425 | 0.580509402 | hypothetical protein |
| CNXL_002200 | CNA02210 | 0.001363085 | 0.997135738 | hypothetical protein |
| CNXL_002210 | CNA02220 | -0.059860969 | 0.918689896 | hypothetical protein |
| CNXL_002220 | CNA02230 | -0.00360574 | 0.996158838 | large subunit ribosomal protein L30e |
| CNXL_002230 | CNA02240 | -0.202198531 | 0.757965387 | chaperone protein |
| CNXL_002240 | CNA02250 | -0.03238658 | 0.965101267 | tRNA-dihydrouridine synthase 4 |
| CNXL_002250 | CNA02260 | -0.167621666 | 0.771763303 | low-affinity ammonium transporter |
| CNXL_002260 | CNA02270 | 0.169374804 | 0.77300142 | 8-amino-7-oxononanoate synthase |
| CNXL_002270 | CNA02290 | -0.305945529 | 0.417866535 | 3-isopropylmalate dehydratase |
| CNXL_002280 | CNA02300 | 0.041728095 | 0.961474415 | isoleucine-tRNA ligase |
| CNXL_002290 | CNA02310 | -0.331000663 | 0.598241347 | hypothetical protein |
| CNXL_002300 | CNA02330 | -0.493503818 | 0.242392789 | hypothetical protein |

| | | | | |
|---|---|---|---|---|
| CNXL_002310 | | -0.663238319 | 0.233174199 | protein AIR1/2 |
| CNXL_002320 | CNA02340 | 0.095646073 | 0.890150525 | hypothetical protein |
| CNXL_002330 | CNA02350 | -0.263313568 | 0.527852637 | hypothetical protein |
| CNXL_002340 | CNA02360 | -0.161414531 | 0.848021859 | ras guanyl-nucleotide exchange factor |
| CNXL_002350 | CNA02370 | -0.02032375 | 0.981240834 | hypothetical protein |
| CNXL_002360 | CNA02380 | 0.060447708 | 0.95265881 | saccharopine dehydrogenase |
| CNXL_002370 | CNA02390 | -0.647133312 | 0.208087633 | ESCRT-II complex subunit VPS36 |
| CNXL_002380 | CNA02400 | -1.31181922 | 1.46558E-06 | hypothetical protein |
| CNXL_002390 | | 0.356109156 | 0.388278926 | hypothetical protein |
| CNXL_002400 | CNA02410 | 0.072849244 | 0.933810298 | hypothetical protein |
| CNXL_002410 | CNA02420 | 0.939259922 | 0.005692302 | transcription elongation factor B |
| CNXL_002420 | CNA02450 | -0.466617426 | 0.456456998 | hypothetical protein |
| CNXL_002430 | CNA02460 | -0.486377259 | 0.121868248 | aspartate-semialdehyde dehydrogenase |
| CNXL_002440 | CNA02470 | -0.044557728 | 0.958917101 | kinesin family member 21A |
| CNXL_002450 | | -0.131959788 | 0.840398313 | Fungal gamma tubulin complex family protein |
| CNXL_002460 | | -0.46191628 | 0.444875077 | hypothetical protein |
| CNXL_002470 | CNA02490 | -0.005016539 | 0.995735232 | hypothetical protein |
| CNXL_002480 | CNA02520 | -3.3134893 | 2.79915E-44 | transformer-2 protein |
| CNXL_002490 | | 0.034653174 | 0.960513489 | hypothetical protein |
| CNXL_002500 | CNA02540 | 0.294188709 | 0.521743214 | serine/threonine/tyrosine-interacting protein |
| CNXL_002510 | CNA02550 | 0.111151062 | 0.846112002 | nuclease I |
| CNXL_002520 | | 0.187447554 | 0.746237137 | isopentenyl-diphosphate delta-isomerase |
| CNXL_002530 | CNA02560 | 0.025725779 | 0.984593329 | hypothetical protein |
| CNXL_002540 | CNA02570 | -0.284650126 | 0.532490033 | hypothetical protein |
| CNXL_002550 | CNA02580 | 0.147967105 | 0.747332879 | acetolactate synthase |
| CNXL_002560 | CNA02590 | -0.306155022 | 0.405021185 | sorbitol dehydrogenase |
| CNXL_002570 | CNA02600 | -0.164930852 | 0.755040408 | mitochondrial protein |
| CNXL_002580 | CNA02610 | -0.172205457 | 0.771763303 | INO80 complex subunit C |
| CNXL_002590 | | -0.039323977 | 0.976006716 | hypothetical protein |
| CNXL_002600 | | 0.882344295 | 0.095784948 | Unknown |
| CNXL_002610 | | -0.336960137 | 0.460815133 | hypothetical protein |
| CNXL_002620 | CNA02630 | 0.158568603 | 0.761797019 | hypothetical protein |
| CNXL_002630 | CNA02640 | 0.357126838 | 0.354037215 | hypothetical protein |
| CNXL_002640 | | -0.084392905 | 0.891600898 | hypothetical protein |
| CNXL_002650 | CNA02650 | 0.109950614 | 0.862977858 | hypothetical protein |
| CNXL_002660 | CNA02660 | 0.166446042 | 0.750884762 | methionine aminopeptidase |
| CNXL_002670 | CNA02670 | 0.051137049 | 0.936783812 | hypothetical protein |
| CNXL_002680 | CNA02680 | 0.208582357 | 0.678131584 | hypothetical protein |
| CNXL_002690 | CNA02690 | 0.006630396 | 0.992176872 | hypothetical protein |
| CNXL_002700 | CNA02700 | -0.328975976 | 0.391858761 | V-type proton ATPase proteolipid subunit 2 |
| CNXL_002710 | CNA02710 | -0.214629015 | 0.676394583 | T-complex protein 1 subunit eta |
| CNXL_002720 | | -2.555882966 | 1.44217E-28 | hypothetical protein |
| CNXL_002730 | CNA02730 | 0.532536055 | 0.098237999 | efflux protein EncT |
| CNXL_002740 | CNA02740 | -0.042028634 | 0.951846328 | hypothetical protein |
| CNXL_002750 | CNA02750 | -0.105847635 | 0.842339591 | hypothetical protein |
| CNXL_002760 | CNA02760 | 0.368402997 | 0.350570336 | cytoplasmic protein |
| CNXL_002770 | CNA02770 | -0.129117775 | 0.865225322 | oligoribonuclease |
| CNXL_002780 | CNA02780 | -0.434935567 | 0.232845005 | hypothetical protein |
| CNXL_002790 | CNA02790 | -0.061821134 | 0.953095191 | aminopeptidase |
| CNXL_002800 | CNA02800 | -0.145810247 | 0.779355929 | hypothetical protein |
| CNXL_002810 | CNA02810 | -0.231608057 | 0.616413141 | zinc finger protein |
| CNXL_002820 | CNA02820 | -0.291978745 | 0.619100215 | G-protein signaling GTPase |
| CNXL_002830 | | -0.511968488 | 0.453868281 | splicing factor 45 |
| CNXL_002840 | CNA02830 | 0.45651686 | 0.187505217 | hypothetical protein |
| CNXL_002850 | | -0.255443505 | 0.592413305 | heat shock protein |
| CNXL_002860 | | 0.159938849 | 0.814730279 | hypothetical protein |
| CNXL_002870 | CNA02850 | -0.035212324 | 0.965101267 | hypothetical protein |
| CNXL_002880 | CNA02860 | 0.157630251 | 0.811287636 | DNA repair protein RAD5 |

| | | | | |
|---|---|---|---|---|
| CNXL_002890 | CNA02870 | 0.511980331 | 0.276076329 | gamma-tubulin complex component 2 |
| CNXL_002900 | | 0.160890693 | 0.84689626 | hypothetical protein |
| CNXL_002910 | CNA02880 | -0.044731517 | 0.957843273 | hypothetical protein |
| CNXL_002920 | | -0.382952656 | 0.504534893 | high-affinity nicotinic acid transporter |
| CNXL_002930 | CNA02890 | 0.223946779 | 0.638378602 | hypothetical protein |
| CNXL_002940 | | -0.135690877 | 0.826907872 | chaperone activator |
| CNXL_002950 | CNA02900 | -0.163481269 | 0.747332879 | hypothetical protein |
| CNXL_002960 | | -0.022303799 | 0.977303234 | hypothetical protein |
| CNXL_002970 | | 0.110300912 | 0.899804184 | hypothetical protein |
| CNXL_002980 | CNA02920 | -0.05314507 | 0.931449101 | hypothetical protein |
| CNXL_002990 | CNA02930 | 0.170583591 | 0.798976008 | membrane protein |
| CNXL_003000 | CNA02940 | -0.107997547 | 0.841905236 | origin recognition complex subunit 5 |
| CNXL_003010 | | 0.250720625 | 0.626703863 | 3-hydroxyisobutyryl-CoA hydrolase |
| CNXL_003020 | CNA02960 | -0.116318444 | 0.85183218 | synaptobrevin |
| CNXL_003030 | | 0.649689334 | 0.061154988 | hypothetical protein |
| CNXL_003040 | | 0.77306964 | 0.039924281 | hypothetical protein |
| CNXL_003050 | CNA02980 | 1.689694961 | 1.78941E-07 | hypothetical protein |
| CNXL_003060 | CNA02990 | 1.033062771 | 0.001774278 | HHE domain-containing protein |
| CNXL_003070 | CNA03000 | -0.131255989 | 0.814480545 | hypothetical protein |
| CNXL_003080 | CNA03010 | 0.514765317 | 0.462504391 | hypothetical protein |
| CNXL_003090 | CNA03020 | 0.373954787 | 0.41607574 | hypothetical protein |
| CNXL_003100 | CNA03030 | 0.26832236 | 0.509453385 | hypothetical protein |
| CNXL_003110 | CNA03040 | -0.462629687 | 0.219542282 | arp2/3 complex 16 kda subunit |
| CNXL_003120 | CNA03050 | -0.068210906 | 0.944263762 | hypothetical protein |
| CNXL_003130 | | -0.047386311 | 0.969179648 | anaphase-promoting complex subunit 3 |
| CNXL_003140 | CNA03060 | 0.32989358 | 0.525959757 | hypothetical protein |
| CNXL_003150 | | 0.360158177 | 0.432050869 | hypothetical protein |
| CNXL_003160 | | 0.741394833 | 0.725978857 | hypothetical protein |
| CNXL_003170 | CNA03070 | 0.021606989 | 0.981567052 | hypothetical protein |
| CNXL_003180 | CNA03090 | 0.48881097 | 0.12383618 | hypothetical protein |
| CNXL_003190 | | 0.121373206 | 0.885831771 | hypothetical protein |
| CNXL_003200 | CNA03100 | -0.008944082 | 0.992176872 | DASH complex subunit DAD2 |
| CNXL_003210 | CNA03110 | 0.002581813 | 0.996472611 | DNA excision repair protein ERCC-5 |
| CNXL_003220 | CNA03120 | -0.537778584 | 0.234185908 | NADH dehydrogenase |
| CNXL_003230 | CNA03130 | -0.068821981 | 0.906691628 | transcription factor TFIIIB component b" |
| CNXL_003240 | CNA03140 | 0.042092183 | 0.948286148 | anon-23da protein |
| CNXL_003250 | CNA03150 | -0.292635653 | 0.514510361 | hypothetical protein |
| CNXL_003260 | CNA03160 | -0.244440014 | 0.534266327 | hypothetical protein |
| CNXL_003270 | CNA03170 | -0.271461834 | 0.606726524 | hsp75-like protein |
| CNXL_003280 | CNA03180 | -0.586619084 | 0.092378562 | hypothetical protein |
| CNXL_003290 | CNA03190 | -0.348881161 | 0.421287685 | hypothetical protein |
| CNXL_003300 | CNA03200 | -0.138503832 | 0.859615039 | hypothetical protein |
| CNXL_003310 | CNA03210 | 0.09910171 | 0.86171371 | 26S proteasome non-ATPase regulatory subunit 9 |
| CNXL_003320 | CNA03220 | 0.122197739 | 0.885370637 | 3' |
| CNXL_003330 | CNA03240 | -0.428112604 | 0.533571143 | hypothetical protein |
| CNXL_003340 | CNA03250 | -0.682807033 | 0.219114292 | hypothetical protein |
| CNXL_003350 | | -0.077167894 | 0.916084262 | hypothetical protein |
| CNXL_003360 | CNA03280 | -0.14921009 | 0.841282635 | hypothetical protein |
| CNXL_003370 | CNA03290 | 0.951541459 | 0.002267039 | saga complex histone acetyltransferase |
| CNXL_003380 | | -0.598399668 | 0.754836997 | hypothetical protein |
| CNXL_003390 | CNA03300 | 0.478484485 | 0.735456246 | hypothetical protein |
| CNXL_003400 | CNA03310 | 0.372285037 | 0.336719089 | glucan 1 |
| CNXL_003410 | CNA03320 | 0.243225144 | 0.55313399 | chromatin structure-remodeling complex protein RSC7 |
| CNXL_003420 | CNA03330 | -0.152284815 | 0.756597645 | methylglutaconyl-CoA hydratase |
| CNXL_003430 | CNA03340 | 0.234677935 | 0.669966613 | hypothetical protein |
| CNXL_003440 | CNA03350 | 0.634511493 | 0.051375479 | hypothetical protein |
| CNXL_003450 | CNA03360 | 0.202275674 | 0.700697818 | vacuolar-sorting protein 53 long isoform |
| CNXL_003460 | CNA03370 | -2.915133076 | 1.7749E-23 | dynactin 5 |

| | | | | |
|---|---|---|---|---|
| CNXL_003470 | | -0.377519592 | 0.416220794 | hypothetical protein |
| CNXL_003480 | CNA03380 | -0.291469395 | 0.586181879 | hypothetical protein |
| CNXL_003490 | CNA03390 | 0.548001239 | 0.120433754 | lysophospholipid acyltransferase |
| CNXL_003500 | CNA03400 | 0.049089015 | 0.95086529 | tRNA threonylcarbamoyladenosine biosynthesis protein |
| CNXL_003510 | CNA03410 | 0.288693735 | 0.576230883 | two-component-like sensor kinase |
| CNXL_003520 | CNA03420 | -0.030791041 | 0.965966884 | hypothetical protein |
| CNXL_003530 | CNA03430 | 0.51463637 | 0.149035091 | hypothetical protein |
| CNXL_003540 | CNA03450 | -1.26299717 | 0.000635796 | nuclear protein SNF4 |
| CNXL_003550 | CNA03460 | -0.091509015 | 0.90492405 | hypothetical protein |
| CNXL_003560 | CNA03470 | -0.155764593 | 0.781247582 | tRNA dimethylallyltransferase |
| CNXL_003570 | CNA03480 | -0.012383165 | 0.987958739 | ribonuclease HI |
| CNXL_003580 | CNA03490 | 0.095707627 | 0.866290947 | survival factor 1 |
| CNXL_003590 | CNA03500 | 0.115102418 | 0.83412415 | hypothetical protein |
| CNXL_003600 | CNA03520 | 0.251221133 | 0.634728039 | vacuolar protein 8 |
| CNXL_003610 | | -0.185864 | 0.742187954 | A1 cistron-splicing factor AAR2 |
| CNXL_003620 | | 0.184053518 | 0.818704891 | hypothetical protein |
| CNXL_003630 | CNA03530 | -0.013761408 | 0.986546741 | hypothetical protein |
| CNXL_003640 | CNA03550 | -0.088648011 | 0.894815557 | succinate dehydrogenase |
| CNXL_003650 | CNA03560 | -0.362280255 | 0.416154363 | hypothetical protein |
| CNXL_003660 | | 0.710014841 | 0.037474908 | DEAH box polypeptide 36 |
| CNXL_003670 | CNA03580 | 0.353731833 | 0.383307552 | hypothetical protein |
| CNXL_003680 | CNA03590 | 0.133507347 | 0.851659386 | hypothetical protein |
| CNXL_003690 | CNA03600 | -0.468749419 | 0.184858591 | hypothetical protein |
| CNXL_003700 | | NA | NA | DNA topoisomerase II |
| CNXL_003710 | | NA | NA | hypothetical protein |
| CNXL_003720 | | NA | NA | hypothetical protein |
| CNXL_003730 | CNE03000 | 2.637144045 | NA | hypothetical protein |
| CNXL_003740 | CNE02980 | 0.594227896 | 0.87678601 | hypothetical protein |
| CNXL_003750 | | 1.197739973 | 0.227278474 | hypothetical protein |
| CNXL_003760 | | NA | NA | hypothetical protein |
| CNXL_003770 | CNA03690 | 0 | NA | hypothetical protein |
| CNXL_003780 | | 0.189273405 | 0.918848255 | hypothetical protein |
| CNXL_003790 | | 0.805074553 | 0.450585467 | hypothetical protein |
| CNXL_003800 | CNA03720 | 0.124794177 | 0.81842519 | hypothetical protein |
| CNXL_003810 | | -1.35965921 | 0.00412885 | aminophospholipid translocase |
| CNXL_003820 | | -1.222632978 | 0.119421369 | RAD51-like protein 2 |
| CNXL_003830 | CNA03730 | -0.176167568 | 0.719248355 | hypothetical protein |
| CNXL_003840 | CNA03740 | -0.630333768 | 0.153214191 | 20S proteasome subunit beta 3 |
| CNXL_003850 | CNA03750 | 0.075198877 | 0.92224659 | translation initiation factor 4G |
| CNXL_003860 | CNA03760 | -0.421425936 | 0.397621992 | hypothetical protein |
| CNXL_003870 | CNA03770 | -0.329305425 | 0.462811802 | 1-phosphatidylinositol-4-phosphate 5-kinase |
| CNXL_003880 | CNA03780 | -0.261150556 | 0.665128131 | mitochondrial protein |
| CNXL_003890 | CNA03790 | -0.236517284 | 0.638320737 | WD-repeat protein JIP5 |
| CNXL_003900 | | -0.089602628 | 0.891261332 | myotubularin |
| CNXL_003910 | | -0.530905509 | 0.483451258 | hypothetical protein |
| CNXL_003920 | | -0.276788406 | 0.735456246 | hypothetical protein |
| CNXL_003930 | CNA03810 | 0.909127524 | 0.001651572 | hypothetical protein |
| CNXL_003940 | CNA03820 | -0.624910874 | 0.09437679 | 1 |
| CNXL_003950 | CNA03830 | 0.048439111 | 0.949084374 | diphthamide biosynthesis protein 3 |
| CNXL_003960 | CNA03840 | 0.387864018 | 0.276952897 | structure-specific endonuclease subunit SLX1 |
| CNXL_003970 | CNA03850 | 0.001705631 | 0.996833113 | protein kinase A catalytic subunit |
| CNXL_003980 | CNA03860 | 0.169609916 | 0.765840893 | 2-oxoisovalerate dehydrogenase E1 component |
| CNXL_003990 | CNA03870 | 0.034950015 | 0.966210833 | hypothetical protein |
| CNXL_004000 | CNA03880 | 0.488871866 | 0.124108583 | transformer-2-beta isoform 3 |
| CNXL_004010 | CNA03890 | -0.069962039 | 0.922663867 | secondary thiamine-phosphate synthase enzyme |
| CNXL_004020 | CNA03900 | -0.04000426 | 0.949084374 | phosphatidylinositol glycan |
| CNXL_004030 | CNA03910 | -0.149169879 | 0.819555276 | translation initiation factor 4G |
| CNXL_004040 | CNA03930 | -0.237963446 | 0.690261873 | mitochondrial protein |

| | | | | |
|---|---|---|---|---|
| CNXL_004050 | CNA03940 | -0.280703302 | 0.660204343 | RING zinc finger protein |
| CNXL_004060 | CNA03950 | -0.193388385 | 0.787454471 | ste/ste20/ysk protein kinase |
| CNXL_004070 | CNA03960 | -3.463731009 | 2.14297E-44 | hypothetical protein |
| CNXL_004080 | CNA03970 | 0.109826073 | 0.857663092 | glyoxal oxidase |
| CNXL_004090 | CNA03990 | 0.163702135 | 0.756596395 | serine/threonine-protein phosphatase catalytic subunit |
| CNXL_004100 | CNA04000 | -0.391880516 | 0.311813532 | hypothetical protein |
| CNXL_004110 | CNA04010 | -0.234487501 | 0.676372743 | hypothetical protein |
| CNXL_004120 | | 0.094985987 | 0.913749948 | nucleoporin p58/p45 |
| CNXL_004130 | CNA04020 | -0.725772171 | 0.016891408 | hypothetical protein |
| CNXL_004140 | CNA04030 | -0.050802066 | 0.94868362 | putative oxidoreductase |
| CNXL_004150 | CNA04040 | 0.2807664 | 0.566540169 | cyclin-dependent kinase of PITSLRE subfamily |
| CNXL_004160 | CNA04050 | -0.078515531 | 0.885370637 | f-box/wd-repeat protein lin-23 |
| CNXL_004170 | CNA04060 | -0.506710146 | 0.131612861 | elongation factor 1-gamma |
| CNXL_004180 | | -0.103320074 | 0.933786064 | S-adenosylmethionine synthase |
| CNXL_004190 | | -0.149308348 | 0.794943165 | DNA polymerase beta subunit |
| CNXL_004200 | | 0.222738363 | 0.686488755 | dolichyl-phosphate mannosyltransferase polypeptide 3 |
| CNXL_004210 | CNA04080 | -0.003003661 | 0.996472611 | charged multivesicular body protein 3 |
| CNXL_004220 | CNA04090 | -1.159587372 | 0.000527628 | hypothetical protein |
| CNXL_004230 | CNA04100 | 0.491169446 | 0.190282816 | kinetochore protein Spc24 |
| CNXL_004240 | | 0.070094822 | 0.95265881 | choline-phosphate cytidylyltransferase |
| CNXL_004250 | CNA04110 | -0.0627324 | 0.936783812 | hypothetical protein |
| CNXL_004260 | CNA04120 | -0.255039727 | 0.560670633 | UBA/TS-N domain-containing protein |
| CNXL_004270 | CNA04130 | -0.752815455 | 0.077637592 | type 2C protein Phosphatase |
| CNXL_004280 | CNA04140 | -0.614111214 | 0.227278474 | hypothetical protein |
| CNXL_004290 | | -0.132988465 | 0.874890451 | tRNA-dihydrouridine synthase 1 |
| CNXL_004300 | CNA04150 | 0.392505754 | 0.312160498 | hypothetical protein |
| CNXL_004310 | CNA04160 | -0.247162863 | 0.553008373 | Atypical/ABC1/ABC1-C protein kinase |
| CNXL_004320 | CNA04170 | 0.223659329 | 0.634728039 | hypothetical protein |
| CNXL_004330 | CNA04180 | 0.738022337 | 0.057616112 | arginine N-methyltransferase 2 |
| CNXL_004340 | CNA04190 | -0.574580011 | 0.074787003 | hypothetical protein |
| CNXL_004350 | CNA04200 | 0.089285345 | 0.908000623 | palmitoyltransferase AKR1 |
| CNXL_004360 | CNA04210 | 0.265363133 | 0.603532035 | anaphase-promoting complex subunit 11 |
| CNXL_004370 | CNA04220 | 0.125419332 | 0.830440343 | hypothetical protein |
| CNXL_004380 | CNA04230 | 0.393508615 | 0.321499513 | hypothetical protein |
| CNXL_004390 | CNA04240 | -0.417684299 | 0.248071372 | cyclin subunit of mediator subcomplex |
| CNXL_004400 | CNA04250 | 0.353352553 | 0.319892928 | inosine-5'-monophosphate dehydrogenase |
| CNXL_004410 | CNA04260 | -0.063373043 | 0.93433919 | cyclin |
| CNXL_004420 | CNA04270 | -0.251631224 | 0.592954724 | hypothetical protein |
| CNXL_004430 | CNA04280 | 0.096337659 | 0.864611433 | nonhistone chromosomal protein |
| CNXL_004440 | CNA04290 | 0.122913596 | 0.819099921 | T-complex protein 1 subunit beta |
| CNXL_004450 | CNA04300 | -0.192380969 | 0.735456246 | V-type H+-transporting ATPase subunit AC39 |
| CNXL_004460 | CNA04320 | -0.23693984 | 0.615722567 | RNA-binding protein NOB1 |
| CNXL_004470 | CNA04330 | -0.189346558 | 0.703985737 | cytoplasmic protein |
| CNXL_004480 | CNA04340 | -0.844316991 | 0.001375111 | isovaleryl-CoA dehydrogenase |
| CNXL_004490 | CNA04350 | -0.168269953 | 0.755421754 | mitochondrial protein |
| CNXL_004500 | CNA04360 | -0.266382258 | 0.6500583 | ubiquitin carboxyl-terminal hydrolase Ubp16 |
| CNXL_004510 | CNA04370 | 0.299647365 | 0.407642256 | hypothetical protein |
| CNXL_004520 | CNA04380 | 0.01394586 | 0.987085598 | glutamine synthetase |
| CNXL_004530 | CNA04390 | 0.226627999 | 0.725978857 | hypothetical protein |
| CNXL_004540 | CNA04400 | -0.264083797 | 0.714106807 | hypothetical protein |
| CNXL_004550 | CNA04410 | 0.055534815 | 0.934072032 | hypothetical protein |
| CNXL_004560 | CNA04420 | -0.022296246 | 0.977343303 | protein kinase C substrate 80K-H |
| CNXL_004570 | | -1.449385089 | 0.633324298 | electron-transferring-flavoprotein dehydrogenase |
| CNXL_004580 | CNA04430 | 0.013257429 | 0.987076518 | hypothetical protein |
| CNXL_004590 | | 1.197176005 | 0.034014041 | pre-mRNA-splicing factor RSE1 |
| CNXL_004600 | CNA04460 | 0.821913713 | 0.005522962 | hypothetical protein |
| CNXL_004610 | CNA04470 | 0.68721054 | 0.046107065 | hypothetical protein |
| CNXL_004620 | CNA04480 | 0.35194584 | 0.38102639 | hypothetical protein |

| | | | | |
|---|---|---|---|---|
| CNXL_004630 | CNA04490 | 0.490718643 | 0.151256368 | hypothetical protein |
| CNXL_004640 | CNA04500 | 0.408808353 | 0.28631663 | hypothetical protein |
| CNXL_004650 | CNA04510 | 0.311180553 | 0.432050869 | endoplasmic reticulum protein |
| CNXL_004660 | CNA04520 | 0.063991218 | 0.932840267 | charged multivesicular body protein 1 |
| CNXL_004670 | CNA04540 | -0.734192614 | 0.067840292 | mitochondrial protein |
| CNXL_004680 | CNA04550 | -0.175298812 | 0.776535714 | hypothetical protein |
| CNXL_004690 | CNA04560 | 0.503501753 | 0.238503403 | ER oligosaccharyl transferase complex subunit |
| CNXL_004700 | CNA04570 | -1.398614555 | 4.78031E-08 | hypothetical protein |
| CNXL_004710 | | -1.28599521 | 0.197652607 | WSC domain-containing protein |
| CNXL_004720 | CNA04590 | 0.294997383 | 0.493812577 | hypothetical protein |
| CNXL_004730 | CNA04600 | -0.259168587 | 0.581889939 | hypothetical protein |
| CNXL_004740 | CNA04610 | 0.210191597 | 0.634323515 | nucleolin |
| CNXL_004750 | CNA04620 | 1.055657553 | 0.000176784 | isocitrate dehydrogenase |
| CNXL_004760 | CNA04630 | 1.084023295 | 0.023311074 | hypothetical protein |
| CNXL_004770 | CNA04640 | 0.411550168 | 0.312497815 | hypothetical protein |
| CNXL_004780 | CNA04650 | -0.027920221 | 0.965966884 | 26S proteasome regulatory subunit N10 |
| CNXL_004790 | CNA04660 | 0.201928881 | 0.669854065 | actin |
| CNXL_004800 | CNA04670 | 1.112378534 | 0.358178392 | 2-oxoisovalerate dehydrogenase E2 component |
| CNXL_004810 | | -0.021474833 | 0.982102702 | hypothetical protein |
| CNXL_004820 | | 0.03268774 | 0.980972278 | hypothetical protein |
| CNXL_004830 | CNA04690 | 0.12522917 | 0.81842519 | hypothetical protein |
| CNXL_004840 | | -0.353887833 | 0.716771972 | hypothetical protein |
| CNXL_004850 | CNA04700 | 0.360844614 | 0.388278926 | hypothetical protein |
| CNXL_004860 | CNA04710 | -0.087697865 | 0.884028948 | acetyl-CoA acyltransferase |
| CNXL_004870 | | 0.258121471 | 0.742187954 | hypothetical protein |
| CNXL_004880 | CNA04720 | -0.862202808 | 0.016626382 | hypothetical protein |
| CNXL_004890 | CNA04730 | 0.131250225 | 0.814480545 | hypothetical protein |
| CNXL_004900 | CNA04740 | -0.500128732 | 0.227069466 | WD-repeat protein 21A |
| CNXL_004910 | CNA04750 | 0.134667865 | 0.838837582 | hypothetical protein |
| CNXL_004920 | CNA04760 | 0.773930043 | 0.01514281 | hypothetical protein |
| CNXL_004930 | CNA04770 | -0.865009133 | 0.002843529 | hypothetical protein |
| CNXL_004940 | CNA04780 | -0.77581839 | 0.003237825 | cell division cycle protein 14 |
| CNXL_004950 | CNA04790 | 3.148731978 | NA | solute carrier family 25 |
| CNXL_004960 | CNA04800 | 0.271822655 | 0.628160086 | hypothetical protein |
| CNXL_004970 | CNA04810 | 0.221462173 | 0.80500309 | hypothetical protein |
| CNXL_004980 | CNA04830 | 0.215383301 | 0.661992658 | hypothetical protein |
| CNXL_004990 | CNA04840 | -0.917141455 | 0.357542656 | protein phosphatase |
| CNXL_005000 | | 0.005603238 | 0.995728975 | hypothetical protein |
| CNXL_005010 | CNA04860 | -0.159396782 | 0.742187954 | large subunit ribosomal protein L35 |
| CNXL_005020 | CNA04870 | 0.047435643 | 0.951498478 | hypothetical protein |
| CNXL_005030 | | 0.390770698 | 0.431820392 | peptidyl-prolyl cis-trans isomerase-like 4 |
| CNXL_005040 | CNA04890 | -0.043387363 | 0.962926966 | hypothetical protein |
| CNXL_005050 | | -0.221335607 | 0.865225322 | hypothetical protein |
| CNXL_005060 | | -0.27640862 | 0.791528992 | hypothetical protein |
| CNXL_005070 | CNA04910 | -0.492318491 | 0.305084524 | hypothetical protein |
| CNXL_005080 | CNA04920 | -0.430637748 | 0.200265575 | membrane-associated retromer complex subunit |
| CNXL_005090 | CNA04930 | -0.081433894 | 0.911754274 | translation initiation factor 3 subunit M |
| CNXL_005100 | | 0.309862421 | 0.789047334 | prefoldin subunit 2 |
| CNXL_005110 | CNA04940 | 0.109556736 | 0.852076639 | hypothetical protein |
| CNXL_005120 | CNA04950 | -0.325279436 | 0.544571109 | mitochondrial carrier protein |
| CNXL_005130 | CNA04960 | -0.273500971 | 0.735456246 | nucleolar protein 53 |
| CNXL_005140 | CNA04970 | 1.021578551 | 0.062398851 | hypothetical protein |
| CNXL_005150 | CNA04980 | -0.083211494 | 0.919554697 | hypothetical protein |
| CNXL_005160 | CNA04990 | 0.120593646 | 0.813598552 | peroxisomal signal |
| CNXL_005170 | CNA05000 | -0.247952268 | 0.690752287 | hypothetical protein |
| CNXL_005180 | CNA05020 | -0.553119093 | 0.084536517 | oxidoreductase |
| CNXL_005190 | CNA05030 | 0.302378995 | 0.586181879 | hypothetical protein |
| CNXL_005200 | CNA05040 | 0.998138286 | 0.010088182 | hypothetical protein |

| | | | | |
|---|---|---|---|---|
| CNXL_005210 | CNA05050 | 0.131172919 | 0.817237481 | C2 domain-containing protein |
| CNXL_005220 | CNA05070 | -0.174208766 | 0.700912881 | hypothetical protein |
| CNXL_005230 | CNA05080 | 0.163970416 | 0.843283188 | dynein light intermediate chain 1 |
| CNXL_005240 | CNA05090 | -0.374216813 | 0.337023834 | hypothetical protein |
| CNXL_005250 | CNA05100 | 0.122605874 | 0.830826702 | translation initiation factor eIF-2B subunit epsilon |
| CNXL_005260 | CNA05110 | -0.102988389 | 0.846287172 | WD-repeat protein 23 |
| CNXL_005270 | CNA05120 | 0.342063743 | 0.36810585 | sulfite transporter |
| CNXL_005280 | CNA05130 | 0.468267818 | 0.208728595 | hypothetical protein |
| CNXL_005290 | | 0.505010014 | 0.260596728 | p-type ATPase sodium pump |
| CNXL_005300 | CNA05140 | 0.17991397 | 0.72158924 | hypothetical protein |
| CNXL_005310 | CNA05150 | 0.186408849 | 0.683247689 | hypothetical protein |
| CNXL_005320 | CNA05160 | -0.16366943 | 0.746765994 | hypothetical protein |
| CNXL_005330 | CNA05170 | -0.035629319 | 0.965101267 | hypothetical protein |
| CNXL_005340 | CNA05180 | -0.041271847 | 0.95086529 | large subunit ribosomal protein L16 |
| CNXL_005350 | CNA05200 | -0.849574403 | 0.016212628 | hypothetical protein |
| CNXL_005360 | | -0.681775366 | 0.13633492 | carnitine O-acetyltransferase |
| CNXL_005370 | CNA05210 | -0.284481196 | 0.499530076 | hypothetical protein |
| CNXL_005380 | CNA05220 | -0.128139298 | 0.872448157 | hypothetical protein |
| CNXL_005390 | CNA05240 | 0.143004043 | 0.855909646 | membrane transporter |
| CNXL_005400 | CNA05250 | 0.552588721 | 0.081873342 | pantothenate transporter |
| CNXL_005410 | CNA05260 | 0.343044637 | 0.509453385 | dimethylaniline monooxygenase |
| CNXL_005420 | CNA05270 | 0.051852172 | 0.936783812 | salicylate hydroxylase |
| CNXL_005430 | | -0.146054834 | 0.856294543 | hypothetical protein |
| CNXL_005440 | CNA05280 | -0.417688924 | 0.586181879 | hypothetical protein |
| CNXL_005450 | CNA05290 | -0.23053174 | 0.637704309 | hypothetical protein |
| CNXL_005460 | CNA05300 | -0.185326281 | 0.704832822 | cohesin loading factor subunit SCC2 |
| CNXL_005470 | CNA05310 | 0.023736885 | 0.972638293 | putative chitin synthase |
| CNXL_005480 | CNA05320 | 0.141615578 | 0.812703117 | hypothetical protein |
| CNXL_005490 | | -0.10721114 | 0.912477825 | hypothetical protein |
| CNXL_005500 | | -0.079078044 | 0.936507583 | hypothetical protein |
| CNXL_005510 | CNA05330 | 0.003618853 | 0.996158838 | hypothetical protein |
| CNXL_005520 | | 0.02900477 | 0.984103354 | poly |
| CNXL_005530 | | 0.262180278 | 0.771763303 | hypothetical protein |
| CNXL_005540 | | 1.287364315 | 0.000890328 | hypothetical protein |
| CNXL_005550 | | -0.207131043 | 0.822421189 | hypothetical protein |
| CNXL_005560 | | -0.151829799 | 0.813394551 | hypothetical protein |
| CNXL_005570 | CNA05360 | 1.306878055 | 7.57076E-05 | hypothetical protein |
| CNXL_005580 | CNA05370 | 0.59688431 | 0.05908683 | calcium activated cation channel protein |
| CNXL_005590 | CNA05380 | 0.178902158 | 0.760551033 | inositol phosphorylsphingolipid-phospholipase C |
| CNXL_005600 | CNA05390 | -0.650003939 | 0.05915075 | hypothetical protein |
| CNXL_005610 | CNA05400 | -0.007221041 | 0.992176872 | casein kinase I |
| CNXL_005620 | CNA05410 | -0.34870853 | 0.430213834 | U6 snRNA-associated Sm-like protein LSm8 |
| CNXL_005630 | | 0.134759701 | 0.805451003 | hypothetical protein |
| CNXL_005640 | CNA05420 | 0.25914871 | 0.560646266 | hypothetical protein |
| CNXL_005650 | CNA05430 | -0.00441334 | 0.995735232 | hypothetical protein |
| CNXL_005660 | CNA05440 | 0.136194218 | 0.813601278 | histone acetyltransferase type B catalytic subunit |
| CNXL_005670 | | 1.189577006 | 0.000348843 | hypothetical protein |
| CNXL_005680 | CNA05460 | -1.904800006 | 7.45636E-10 | hypothetical protein |
| CNXL_005690 | CNA05470 | 0.103564638 | 0.8610985 | hypothetical protein |
| CNXL_005700 | CNA05480 | 0.843103838 | 0.002917755 | dynamin GTPase |
| CNXL_005710 | CNA05490 | -0.101201795 | 0.856469734 | DNA ligase 1 |
| CNXL_005720 | | 0.850724571 | 0.006384724 | WD40 repeat protein |
| CNXL_005730 | CNA05500 | -0.219235217 | 0.662943154 | hypothetical protein |
| CNXL_005740 | CNA05510 | 0.223774424 | 0.592954724 | GTP cyclohydrolase II |
| CNXL_005750 | | NA | NA | camp dependent protein kinase regulatory subunit |
| CNXL_005760 | CNA05540 | 0 | NA | hypothetical protein |
| CNXL_005770 | CNA05560 | 0.299713822 | 0.532274144 | hypothetical protein |
| CNXL_005780 | CNA05570 | 0.257642557 | 0.552790986 | hypothetical protein |

| | | | | |
|---|---|---|---|---|
| CNXL_005790 | CNA05580 | -0.17440149 | 0.730212038 | DNA excision repair protein ERCC-2 |
| CNXL_005800 | CNA05590 | 0.176707693 | 0.725978857 | NADH dehydrogenase |
| CNXL_005810 | | 1.490533406 | 2.92983E-05 | amino acid/metabolite permease |
| CNXL_005820 | | 1.673230608 | 7.78804E-06 | hypothetical protein |
| CNXL_005830 | CNA05610 | 0.607525497 | 0.063742019 | hypothetical protein |
| CNXL_005840 | CNA05620 | 0.390533823 | 0.500319755 | mitochondrial protein |
| CNXL_005850 | | 0.302009272 | 0.681796222 | hypothetical protein |
| CNXL_005860 | CNA05630 | -0.12357588 | 0.820247927 | hypothetical protein |
| CNXL_005870 | CNA05650 | 0.383952392 | 0.360562897 | translation initiation factor IF-2 |
| CNXL_005880 | CNA05660 | 0.465267028 | 0.162514284 | saccharopepsin |
| CNXL_005890 | CNA05670 | -0.060737016 | 0.933394957 | vacuolar transporter chaperone 1 |
| CNXL_005900 | CNA05680 | 0.32443571 | 0.334783364 | hypothetical protein |
| CNXL_005910 | CNA05690 | 0.059402717 | 0.949084374 | profilin |
| CNXL_005920 | CNA05700 | -0.17337832 | 0.764293155 | hypothetical protein |
| CNXL_005930 | CNA05710 | -0.625208679 | 0.170587304 | hypothetical protein |
| CNXL_005940 | CNA05720 | 0.253768172 | 0.630279293 | hypothetical protein |
| CNXL_005950 | | 0.045278584 | 0.952791117 | hypothetical protein |
| CNXL_005960 | CNA05730 | -0.209411404 | 0.741662589 | 5-formyltetrahydrofolate cyclo-ligase |
| CNXL_005970 | | 0.388080155 | 0.75185116 | para-aminobenzoate synthetase |
| CNXL_005980 | CNA05740 | 0.077610865 | 0.920697736 | hypothetical protein |
| CNXL_005990 | CNA05750 | -0.212063027 | 0.779355929 | dolichol-phosphate mannosyltransferase |
| CNXL_006000 | CNA05760 | -0.015020423 | 0.986620478 | hypothetical protein |
| CNXL_006010 | | 0.746564872 | 0.012690294 | lipid particle protein |
| CNXL_006020 | CNA05790 | -0.698697545 | 0.012024131 | hypothetical protein |
| CNXL_006030 | CNA05800 | 0.01406984 | 0.985595171 | hypothetical protein |
| CNXL_006040 | CNA05810 | 0.328064947 | 0.406732739 | hypothetical protein |
| CNXL_006050 | CNA05820 | -0.158843501 | 0.755572574 | nicotinamide mononucleotide permease |
| CNXL_006060 | CNA05830 | -0.550653008 | 0.110174855 | anion transporter |
| CNXL_006070 | CNA05840 | -0.54262423 | 0.049073907 | capsule-associated protein |
| CNXL_006080 | CNA05850 | -0.376613404 | 0.261879704 | putative glycosyl hydrolase |
| CNXL_006090 | CNA05860 | -0.174374386 | 0.749039348 | translation initiation factor 3 subunit I |
| CNXL_006100 | CNA05870 | -0.524667762 | 0.161311453 | pre-rRNA-processing protein PNO1 |
| CNXL_006110 | CNA05880 | 0.7975609 | 0.01440355 | hypothetical protein |
| CNXL_006120 | CNA05890 | -0.321641745 | 0.527852637 | cytoplasmic protein |
| CNXL_006130 | CNA05900 | -0.080407723 | 0.893955723 | transcription initiation factor TFIID subunit 7 |
| CNXL_006140 | CNA05910 | -0.116546521 | 0.846287172 | hypothetical protein |
| CNXL_006150 | CNA05920 | -0.213334109 | 0.638320737 | hypothetical protein |
| CNXL_006160 | | 0.356123793 | 0.554133845 | hypothetical protein |
| CNXL_006170 | CNA05930 | -0.552319871 | 0.164687762 | hypothetical protein |
| CNXL_006180 | CNA05940 | 0.026542061 | 0.973463477 | WD repeat and SOF domain-containing protein 1 |
| CNXL_006190 | CNA05950 | 0.141309093 | 0.803882252 | DNA excision repair protein ERCC-3 |
| CNXL_006200 | CNA05960 | -0.246046398 | 0.632998683 | cytosine deaminase |
| CNXL_006210 | CNA05970 | -0.019944855 | 0.990008547 | hypothetical protein |
| CNXL_006220 | | -0.092279382 | 0.902229878 | hypothetical protein |
| CNXL_006230 | | -0.165278847 | 0.792671606 | hypothetical protein |
| CNXL_006240 | CNA05990 | 0.124702219 | 0.838178569 | hypothetical protein |
| CNXL_006250 | CNA06000 | -0.675888474 | 0.061114824 | tubulin folding cofactor C |
| CNXL_006260 | CNA06010 | -0.552230462 | 0.401258094 | carbamoyl-phosphate synthase |
| CNXL_006270 | CNA06020 | -0.131708591 | 0.83412486 | DNA-directed RNA polymerase III subunit RPC5 |
| CNXL_006280 | CNA06030 | -0.188393883 | 0.700912881 | acetylornithine aminotransferase |
| CNXL_006290 | CNA06040 | 0.247385089 | 0.606726524 | fungus-specific glucosylceramide |
| CNXL_006300 | CNA06050 | 0.196060378 | 0.706531725 | hypothetical protein |
| CNXL_006310 | CNA06060 | 0.869157438 | 0.004885889 | ARF guanyl-nucleotide exchange factor |
| CNXL_006320 | CNA06070 | 0.32538659 | 0.681029948 | hypothetical protein |
| CNXL_006330 | CNA06080 | -0.056233185 | 0.939255348 | specific transcriptional repressor |
| CNXL_006340 | | 0.102542103 | 0.925793142 | hypothetical protein |
| CNXL_006350 | CNA06090 | 0.240705764 | 0.622869303 | Unknown |
| CNXL_006360 | | -0.079625777 | 0.909205841 | guanyl-nucleotide exchange factor |

| | | | | |
|---|---|---|---|---|
| CNXL_006370 | CNA06110 | -0.031287328 | 0.965101267 | Unknown |
| CNXL_006380 | CNA06120 | -0.132505296 | 0.797709591 | hypothetical protein |
| CNXL_006390 | CNA06130 | -0.289050236 | 0.523358959 | hypothetical protein |
| CNXL_006400 | CNA06140 | -0.140131642 | 0.795111334 | hypothetical protein |
| CNXL_006410 | CNA06150 | -0.124159412 | 0.817518655 | hypothetical protein |
| CNXL_006420 | CNA06160 | -0.598004427 | 0.156637331 | cytoplasmic protein |
| CNXL_006430 | CNA06170 | 0.20054458 | 0.672636096 | CDC7 protein kinase |
| CNXL_006440 | CNA06180 | 0.895417244 | 0.001546193 | cystathionine beta-synthase |
| CNXL_006450 | CNA06190 | -0.108055721 | 0.879713683 | GTPase |
| CNXL_006460 | CNA06200 | -0.036624799 | 0.956622745 | OHCU decarboxylase |
| CNXL_006470 | CNA06210 | -0.241797204 | 0.624278162 | small subunit ribosomal protein S4-A |
| CNXL_006480 | CNA06220 | -1.115935225 | 0.000258781 | transcription elongation factor SPT5 |
| CNXL_006490 | CNA06230 | -0.424412221 | 0.332119185 | hypothetical protein |
| CNXL_006500 | CNA06240 | -0.200622722 | 0.796334467 | hypothetical protein |
| CNXL_006510 | | -0.138649193 | 0.869656381 | hypothetical protein |
| CNXL_006520 | CNA06260 | -0.327764459 | 0.45681383 | 2 |
| CNXL_006530 | CNA06270 | -0.406214543 | 0.241458451 | hypothetical protein |
| CNXL_006540 | CNA06280 | 0.759129576 | 0.03627015 | hypothetical protein |
| CNXL_006550 | CNA06290 | -0.344866827 | 0.483067762 | esterase/lipase |
| CNXL_006560 | CNA06300 | -0.253981849 | 0.591488032 | tryptophan synthase |
| CNXL_006570 | CNA06310 | -0.217121351 | 0.667369865 | hypothetical protein |
| CNXL_006580 | CNA06320 | -0.86600021 | 0.002138362 | hypothetical protein |
| CNXL_006590 | CNA06330 | -3.190982766 | 7.14433E-22 | hypothetical protein |
| CNXL_006600 | CNA06340 | 1.616837739 | 0.016212628 | hypothetical protein |
| CNXL_006610 | CNA06350 | -0.0709135 | 0.906291044 | sulfiredoxin |
| CNXL_006620 | CNA06360 | -0.229122534 | 0.568883048 | large subunit acidic ribosomal protein P1 |
| CNXL_006630 | | 0.525700362 | 0.616439959 | hypothetical protein |
| CNXL_006640 | CNA06370 | -0.270661134 | 0.71157929 | hypothetical protein |
| CNXL_006650 | CNA06380 | -0.074149311 | 0.939255348 | hypothetical protein |
| CNXL_006660 | CNA06390 | -0.105965452 | 0.900330972 | TFIIH basal transcription factor complex TTD-A subunit |
| CNXL_006670 | CNA06400 | 0.002009006 | 0.997553627 | histone deacetylase HOS3 |
| CNXL_006680 | CNA06410 | 0.195624661 | 0.688968135 | hypothetical protein |
| CNXL_006690 | CNA06420 | -0.248711013 | 0.71410991 | carboxymethylenebutenolidase |
| CNXL_006700 | | -0.312296445 | 0.690752287 | hypothetical protein |
| CNXL_006710 | CNA06430 | 0.110476717 | 0.846287172 | hypothetical protein |
| CNXL_006720 | CNA06440 | -0.173285987 | 0.78519633 | hypothetical protein |
| CNXL_006730 | CNA06450 | -0.179236774 | 0.779355929 | nucleoporin nsp1 |
| CNXL_006740 | CNA06460 | 0.184916107 | 0.678131584 | hypothetical protein |
| CNXL_006750 | CNA06480 | -0.863636812 | 0.019801897 | hypothetical protein |
| CNXL_006760 | CNA06470 | -0.323506594 | 0.441275494 | hypothetical protein |
| CNXL_006770 | CNA06490 | 0.568061022 | 0.107353814 | hypothetical protein |
| CNXL_006780 | CNA06500 | -0.130168086 | 0.78773562 | cytoplasmic protein |
| CNXL_006790 | CNA06510 | 0.191973238 | 0.718337881 | small subunit ribosomal protein S11 |
| CNXL_006800 | CNA06520 | 0.155815078 | 0.819555276 | cytoplasmic protein |
| CNXL_006810 | CNA06530 | -0.184539507 | 0.73464097 | hypothetical protein |
| CNXL_006820 | CNA06540 | 0.3748212 | 0.305163836 | carnitine/acyl carnitine carrier |
| CNXL_006830 | CNA06550 | -0.491295904 | 0.388422979 | ESCRT-I complex subunit VPS28 |
| CNXL_006840 | | 0.251580446 | 0.701325477 | E3 ubiquitin-protein ligase UHRF1 |
| CNXL_006850 | CNA06560 | 0.018918006 | 0.981567052 | hypothetical protein |
| CNXL_006860 | CNA06570 | 0.480251737 | 0.105763986 | urease accessory protein |
| CNXL_006870 | CNA06580 | 0.08573757 | 0.885449489 | hypothetical protein |
| CNXL_006880 | CNA06590 | 0.524663371 | 0.176678435 | kinetochore protein Nuf2 |
| CNXL_006890 | | -0.232315763 | 0.719248355 | condensin complex subunit 3 |
| CNXL_006900 | | -0.274188712 | 0.540948602 | Unknown |
| CNXL_006910 | CNA06610 | -0.134869548 | 0.812003184 | Unknown |
| CNXL_006920 | | -0.369910042 | 0.404910404 | kinesin |
| CNXL_006930 | CNA06620 | -0.076159696 | 0.918375149 | Unknown |
| CNXL_006940 | CNA06630 | 0.656629279 | 0.03987196 | CMGC/CLK protein kinase |

| | | | | |
|---|---|---|---|---|
| CNXL_006950 | CNA06640 | 0.015843334 | 0.987076518 | ribulose-phosphate 3-epimerase |
| CNXL_006960 | CNA06650 | 1.006414335 | 0.000666369 | hypothetical protein |
| CNXL_006970 | CNA06660 | -0.261260413 | 0.623472961 | hypothetical protein |
| CNXL_006980 | | -0.304384131 | 0.488583556 | hypothetical protein |
| CNXL_006990 | CNA06670 | 0.439326434 | 0.197592932 | hypothetical protein |
| CNXL_007000 | CNA06680 | 0.129074253 | 0.825693641 | beta-1 |
| CNXL_007010 | CNA06690 | -0.094061428 | 0.879531297 | hypothetical protein |
| CNXL_007020 | CNA06700 | -0.058518091 | 0.939400455 | hypothetical protein |
| CNXL_007030 | CNA06710 | -0.523965073 | 0.163523664 | FAD dependent oxidoreductase |
| CNXL_007040 | CNA06730 | -0.032626826 | 0.963072147 | hypothetical protein |
| CNXL_007050 | CNA06740 | 0.327211654 | 0.514510361 | hypothetical protein |
| CNXL_007060 | CNA06750 | 0.289685091 | 0.462811802 | hypothetical protein |
| CNXL_007070 | CNA06760 | 0.717588216 | 0.023709223 | hypothetical protein |
| CNXL_007080 | CNA06770 | 0.187200954 | 0.703985737 | UDP-glucose epimerase |
| CNXL_007090 | CNA06780 | -0.303491022 | 0.777403833 | hypothetical protein |
| CNXL_007100 | CNA06790 | 0.053086181 | 0.934642219 | hypothetical protein |
| CNXL_007110 | CNA06800 | -0.010664953 | 0.990008547 | hypothetical protein |
| CNXL_007120 | CNA06810 | 0.315490262 | 0.436229174 | hypothetical protein |
| CNXL_007130 | CNA06820 | -0.076230589 | 0.902229878 | hypothetical protein |
| CNXL_007140 | CNA06830 | -0.34635625 | 0.43363696 | hypothetical protein |
| CNXL_007150 | CNA06840 | 0.063835595 | 0.918756814 | hypothetical protein |
| CNXL_007160 | CNA06850 | -0.067706706 | 0.923885152 | RuvB-like helicase 2 |
| CNXL_007170 | CNA06860 | 0.03323343 | 0.962706186 | RNA-binding protein rnp24 |
| CNXL_007180 | CNA06870 | 0.432146048 | 0.230241346 | protein SYM1 |
| CNXL_007190 | CNA06880 | -0.068097771 | 0.933276008 | glutamine synthetase |
| CNXL_007200 | CNA06890 | -0.10599385 | 0.848021859 | uracil-DNA glycosylase |
| CNXL_007210 | CNA06900 | 0.826475874 | 0.004678464 | pre-mRNA-splicing factor SLT11 |
| CNXL_007220 | CNA06910 | 0.275503825 | 0.570605968 | hypothetical protein |
| CNXL_007230 | CNA06920 | -0.10639636 | 0.860561823 | hypothetical protein |
| CNXL_007240 | CNA06930 | -1.608671382 | 0.000337919 | hypothetical protein |
| CNXL_007250 | | -0.153049356 | 0.783630658 | hypothetical protein |
| CNXL_007260 | CNA06950 | 0.187241611 | 0.664461114 | hypothetical protein |
| CNXL_007270 | CNA06960 | 0.448888411 | 0.242541001 | cytochrome c |
| CNXL_007280 | CNA06970 | -0.336290321 | 0.567249149 | hypothetical protein |
| CNXL_007290 | CNA06980 | -0.669170921 | 0.205413742 | hypothetical protein |
| CNXL_007300 | CNA06990 | -0.754872636 | 0.040601652 | hypothetical protein |
| CNXL_007310 | CNA07000 | -0.181613293 | 0.770559628 | DNA repair protein RAD51 |
| CNXL_007320 | CNA07010 | -0.154127999 | 0.779355929 | alpha-1 |
| CNXL_007330 | CNA07020 | 0.25134429 | 0.72030696 | large subunit ribosomal protein L27 |
| CNXL_007340 | CNA07030 | -0.26922513 | 0.617084227 | hypothetical protein |
| CNXL_007350 | CNA07040 | -0.188224628 | 0.68904326 | ATP-dependent RNA helicase prh1 |
| CNXL_007360 | CNA07050 | 0.466505909 | 0.247895546 | hypothetical protein |
| CNXL_007370 | CNA07060 | -0.535361887 | 0.114620357 | hypothetical protein |
| CNXL_007380 | CNA07070 | -0.757793008 | 0.122306443 | mitochondrial protein with role in iron accumulation |
| CNXL_007390 | CNA07080 | 0.587168409 | 0.09592004 | dityrosine transporter |
| CNXL_007400 | CNA07090 | -1.044578454 | 1.84876E-05 | hypothetical protein |
| CNXL_007410 | CNA07100 | -0.632659854 | 0.050992608 | ATP-binding cassette transporter |
| CNXL_007420 | CNA07110 | -0.039337732 | 0.958917101 | hypothetical protein |
| CNXL_007430 | CNA07120 | -0.208508619 | 0.653017727 | hypothetical protein |
| CNXL_007440 | CNA07130 | -0.377033248 | 0.355793208 | dihydroorotase |
| CNXL_007450 | CNA07140 | 0.284622268 | 0.49418561 | aldehyde dehydrogenase family 7 member A1 |
| CNXL_007460 | CNA07150 | 0.050974333 | 0.954207791 | exocyst protein |
| CNXL_007470 | CNA07170 | -0.074688838 | 0.95613742 | hypothetical protein |
| CNXL_007480 | CNA07180 | -0.013569451 | 0.988873975 | hypothetical protein |
| CNXL_007490 | CNA07190 | -0.259036837 | 0.628160086 | hypothetical protein |
| CNXL_007500 | CNA07200 | -0.074462576 | 0.903834486 | swi/snf chromatin-remodeling complex subunit |
| CNXL_007510 | CNA07210 | -0.799312876 | 0.027422024 | ATP-dependent RNA helicase SUB2 |
| CNXL_007520 | CNA07220 | -0.2138427 | 0.700630908 | serine palmitoyltransferase |

| | | | | |
|---|---|---|---|---|
| CNXL_007530 | CNA07230 | 0.34407811 | 0.454587377 | imidazoleglycerol phosphate synthase |
| CNXL_007540 | CNA07240 | 0.608812171 | 0.031829912 | alpha 1 |
| CNXL_007550 | CNA07260 | 0.104206032 | 0.84269935 | serine/threonine-protein kinase |
| CNXL_007560 | | 0.736022618 | 0.05245218 | succinyl-CoA synthetase beta subunit |
| CNXL_007570 | | 0.436237063 | 0.58502688 | hypothetical protein |
| CNXL_007580 | NA07280\|CNA072[illegible] | 0.152856949 | 0.789076022 | hypothetical protein |
| CNXL_007590 | | 0.191177146 | 0.902044607 | hypothetical protein |
| CNXL_007600 | CNA07300 | 1.226031574 | 1.66633E-05 | hypothetical protein |
| CNXL_007610 | CNA07310 | -0.156844707 | 0.808695753 | hypothetical protein |
| CNXL_007620 | CNA07320 | -0.380838243 | 0.393218452 | transducin family protein |
| CNXL_007630 | CNA07330 | -0.439301054 | 0.174335736 | hypothetical protein |
| CNXL_007640 | CNA07340 | 0.055408944 | 0.949641665 | hypothetical protein |
| CNXL_007650 | CNA07350 | -0.015088533 | 0.98818763 | hypothetical protein |
| CNXL_007660 | CNA07360 | -0.226996322 | 0.669854065 | hypothetical protein |
| CNXL_007670 | CNA07370 | -2.635118689 | 6.7998E-17 | ubiquitin carboxyl-terminal hydrolase |
| CNXL_007680 | CNA07380 | 0.140036957 | 0.920669589 | regulatory protein ral2 |
| CNXL_007690 | CNA07390 | -0.593007424 | 0.261955879 | hypothetical protein |
| CNXL_007700 | | -0.086489686 | 0.891760995 | methylenetetrahydrofolate reductase |
| CNXL_007710 | CNA07400 | -0.275205759 | 0.662390198 | coiled-coil domain-containing protein 12 |
| CNXL_007720 | CNA07410 | 0.132212922 | 0.819835748 | diphthamide biosynthesis protein |
| CNXL_007730 | CNA07420 | -0.417053779 | 0.414012366 | hypothetical protein |
| CNXL_007740 | CNA07430 | -1.964778502 | 7.25317E-17 | ATP-dependent RNA helicase DBP4 |
| CNXL_007750 | CNA07450 | 0.489929903 | 0.196906673 | hypothetical protein |
| CNXL_007760 | | -1.991799423 | 2.58654E-14 | hypothetical protein |
| CNXL_007770 | CNA07460 | 0.006975122 | 0.993259314 | hypothetical protein |
| CNXL_007780 | CNA07470 | -0.219167317 | 0.683642142 | hypothetical protein |
| CNXL_007790 | CNA07480 | -0.439660855 | 0.2824512 | hypothetical protein |
| CNXL_007800 | CNA07490 | -0.289433407 | 0.430945378 | DNA mismatch repair protein MSH2 |
| CNXL_007810 | CNA07500 | -0.176326784 | 0.78495956 | large subunit ribosomal protein L29 |
| CNXL_007820 | CNA07510 | 0.466944444 | 0.285965334 | putative UV excision repair protein |
| CNXL_007830 | CNA07520 | -0.226035863 | 0.636323797 | hypothetical protein |
| CNXL_007840 | CNA07530 | -0.434024812 | 0.45163285 | phosphoglycerate dehydrogenase |
| CNXL_007850 | CNA07540 | -0.966971173 | 0.000110022 | U3 small nucleolar RNA-associated protein 15 |
| CNXL_007860 | CNA07550 | -0.746494599 | 0.011562656 | immunoreactive mannoprotein |
| CNXL_007870 | CNA07560 | -0.446479045 | 0.346301684 | CCR4-NOT transcriptional complex subunit CAF120 |
| CNXL_007880 | CNA07570 | -0.161444922 | 0.742187954 | hypothetical protein |
| CNXL_007890 | CNA07580 | -1.263469525 | 0.00600962 | large subunit ribosomal protein L27e |
| CNXL_007900 | CNA07590 | 0.036575068 | 0.960513489 | hypothetical protein |
| CNXL_007910 | CNA07600 | -0.733470018 | 0.077441507 | U6 snRNA-associated Sm-like protein LSm3 |
| CNXL_007920 | | 0.518161657 | 0.205950327 | STE/STE20/Fray protein kinase |
| CNXL_007930 | CNA07610 | -0.396000104 | 0.293624485 | hypothetical protein |
| CNXL_007940 | CNA07620 | -0.32106353 | 0.335274247 | hypothetical protein |
| CNXL_007950 | CNA07630 | 0.014677038 | 0.988855811 | ATP-dependent RNA helicase eIF4A |
| CNXL_007960 | CNA07640 | -0.166265591 | 0.776955714 | hypothetical protein |
| CNXL_007970 | CNA07650 | -0.403295464 | 0.248605145 | hypothetical protein |
| CNXL_007980 | CNA07660 | 0.14803587 | 0.798976008 | NADH dehydrogenase |
| CNXL_007990 | CNA07670 | -0.59759736 | 0.124762015 | PAP2 domain-containing protein |
| CNXL_008000 | CNA07680 | 0.388702748 | 0.237871488 | hypothetical protein |
| CNXL_008010 | CNA07690 | -0.044238442 | 0.974020242 | hypothetical protein |
| CNXL_008020 | CNA07700 | 0.060067227 | 0.92313127 | hypothetical protein |
| CNXL_008030 | CNA07710 | -0.192881996 | 0.689993365 | ATP-binding cassette transporter |
| CNXL_008040 | | 0.204561921 | 0.749362254 | DNA-directed RNA polymerase I |
| CNXL_008050 | CNA07720 | -5.649700559 | 8.4455E-110 | hypothetical protein |
| CNXL_008060 | CNA07730 | -0.381172805 | 0.264396338 | hyphal flocculin |
| CNXL_008070 | CNA07740 | -0.470602104 | 0.251404171 | hypothetical protein |
| CNXL_008080 | | -1.886892584 | 6.44332E-10 | hypothetical protein |
| CNXL_008090 | | 0.468065438 | 0.208746699 | hypothetical protein |
| CNXL_008100 | CNA07770 | -2.482559594 | 8.76522E-16 | hypothetical protein |

| | | | | |
|---|---|---|---|---|
| CNXL_008110 | CNA07780 | -0.700801491 | 0.14789324 | cellulase |
| CNXL_008120 | | 0.804510755 | 0.0117379 | nicotinamidase |
| CNXL_008130 | CNA07790 | 0.168586129 | 0.804701377 | hypothetical protein |
| CNXL_008140 | CNA07800 | 0.257561117 | 0.569003608 | calcineurin binding protein |
| CNXL_008150 | CNA07810 | 0.19021146 | 0.716440724 | hypothetical protein |
| CNXL_008160 | CNA07820 | 0.057705674 | 0.943772344 | Gtr1/RagA G protein Gtr1 |
| CNXL_008170 | CNA07830 | 0.075480113 | 0.889191243 | hypothetical protein |
| CNXL_008180 | CNA07840 | 0.488598009 | 0.177772838 | hypothetical protein |
| CNXL_008190 | CNA07850 | -1.040833716 | 3.89492E-05 | hypothetical protein |
| CNXL_008200 | CNA07860 | -0.610492554 | 0.123440865 | myosin regulatory light chain cdc4 |
| CNXL_008210 | CNA07870 | 0.301369421 | 0.593883843 | hypothetical protein |
| CNXL_008220 | CNA07880 | -0.022309351 | 0.985173026 | polynucleotide kinase 3'-phosphatase |
| CNXL_008230 | CNA07890 | -0.463111832 | 0.279637638 | hypothetical protein |
| CNXL_008240 | | -0.768238196 | 0.541921648 | hypothetical protein |
| CNXL_008250 | CNA07900 | 0.334025586 | 0.594692631 | hypothetical protein |
| CNXL_008260 | | 0.184140789 | 0.78205771 | hypothetical protein |
| CNXL_008270 | CNA07920 | 0.265955342 | 0.495140189 | hypothetical protein |
| CNXL_008280 | CNA07930 | 0.326307984 | 0.387277366 | siderophore iron transporter |
| CNXL_008290 | CNA07940 | 0.08720797 | 0.909451569 | microtubule binding protein |
| CNXL_008300 | CNA07950 | -0.242385166 | 0.664461114 | hypothetical protein |
| CNXL_008310 | CNA07960 | -0.282411946 | 0.556872599 | hypothetical protein |
| CNXL_008320 | CNA07970 | -0.052661452 | 0.944879244 | small subunit ribosomal protein S30 |
| CNXL_008330 | CNA07980 | -0.191498246 | 0.662983357 | prephenate dehydratase |
| CNXL_008340 | CNA07990 | -0.242615027 | 0.623549135 | large subunit ribosomal protein L34e |
| CNXL_008350 | CNA08000 | 0.24976139 | 0.566184472 | ribosome assembly protein SQT1 |
| CNXL_008360 | CNA08010 | 0.021921765 | 0.981240834 | cadmium ion transporter |
| CNXL_008370 | | 0.659036432 | 0.067063478 | ubiquitin-conjugating enzyme E2 2 |
| CNXL_008380 | CNA08040 | 0.11146547 | 0.874890451 | hypothetical protein |
| CNXL_008390 | CNA08050 | 0.172192225 | 0.732349499 | solute carrier family 25 |
| CNXL_008400 | CNA08060 | 0.172237355 | 0.729199291 | dihydroxyacetone kinase |
| CNXL_008410 | CNA08070 | -0.366601132 | 0.526368704 | ribose 5-phosphate isomerase |
| CNXL_008420 | CNA08080 | 0.098719472 | 0.851880548 | Zn2-Cys6 zinc-finger transcription factor |
| CNXL_008430 | CNA08090 | -0.350247244 | 0.422478883 | E3 ubiquitin ligase complex SCF subunit sconC |
| CNXL_008440 | CNA08100 | 0.066200846 | 0.918701468 | hypothetical protein |
| CNXL_008450 | CNA08110 | 0.560617447 | 0.062809268 | nucleoside-diphosphate-sugar epimerase |
| CNXL_008460 | | 0.638201642 | 0.033808576 | D-lactate dehydrogenase |
| CNXL_008470 | CNA08120 | 1.585018343 | 9.43932E-08 | hypothetical protein |
| CNXL_008480 | CNA08130 | 0.01953973 | 0.983931833 | phosphatidylserine decarboxylase |
| CNXL_008490 | CNA08140 | -0.041125624 | 0.962850612 | 2-hydroxyacid dehydrogenase |
| CNXL_008500 | CNA08150 | -0.355544304 | 0.638378602 | hypothetical protein |
| CNXL_008510 | CNA08160 | -0.465800248 | 0.511541134 | hypothetical protein |
| CNXL_008520 | CNA08170 | -0.272718645 | 0.670362199 | mitochondrial protein |
| CNXL_008530 | CNA08180 | -0.067512711 | 0.926035744 | hypothetical protein |
| CNXL_008540 | CNA08190 | -0.017712639 | 0.988662258 | hypothetical protein |
| CNXL_008550 | CNA08200 | -0.643422113 | 0.31818404 | hypothetical protein |
| CNXL_008560 | CNA08210 | 0.319204737 | 0.615328522 | salicylate hydroxylase |
| CNXL_008570 | CNA08220 | -0.13937751 | 0.816572532 | hypothetical protein |
| CNXL_008580 | CNA08230 | -0.451134844 | 0.437094312 | hypothetical protein |
| CNXL_008590 | CNA08240 | 0.606970765 | 0.069009964 | hypothetical protein |
| CNXL_008600 | CNA08250 | 2.083399523 | 0.014384482 | hypothetical protein |
| CNXL_008610 | CNA08260 | -1.110706846 | 0.113628393 | hypothetical protein |
| CNXL_008620 | | 1.313842359 | 0.001356716 | plant-inducible protein |
| CNXL_008630 | CNA08270 | 0.156096631 | 0.814220979 | hypothetical protein |
| CNXL_008640 | CNA08280 | -0.050057916 | 0.948173304 | hypothetical protein |
| CNXL_008650 | CNA08290 | -0.854273882 | 0.00465933 | exosome complex component RRP43 |
| CNXL_008660 | CNA08310 | 0.202801298 | 0.799344206 | C-8 sterol isomerase |
| CNXL_008670 | CNA08320 | 0.320843685 | 0.527938546 | ligase |
| CNXL_008680 | | 0.212087446 | 0.680080961 | siderophore-iron transporter Str3 |

| | | | | |
|---|---|---|---|---|
| CNXL_008690 | CNA08340 | 0.164672463 | 0.811154336 | hypothetical protein |
| CNXL_008700 | CNA08350 | -0.542574594 | 0.893955723 | hypothetical protein |
| CNXL_008710 | CNA08360 | 0 | NA | UDP-glucose 4-epimerase |
| CNXL_008720 | | NA | NA | hypothetical protein |
| CNXL_008730 | CNB00010 | 0 | NA | hypothetical protein |
| CNXL_008740 | CNB00020 | 1.063038967 | 0.003552024 | hypothetical protein |
| CNXL_008750 | CNB00030 | 0.289831983 | 0.498081161 | hypothetical protein |
| CNXL_008760 | CNB00040 | -0.256991335 | 0.614333627 | uroporphyrinogen decarboxylase |
| CNXL_008770 | CNB00050 | -0.658305581 | 0.062444654 | hypothetical protein |
| CNXL_008780 | CNB00060 | 0.33934123 | 0.469848742 | hypothetical protein |
| CNXL_008790 | | -3.617326572 | 7.50058E-19 | hypothetical protein |
| CNXL_008800 | CNB00070 | -0.233679763 | 0.631694508 | hypothetical protein |
| CNXL_008810 | CNB00080 | -0.630702489 | 0.146723052 | hypothetical protein |
| CNXL_008820 | CNB00090 | 0.217000228 | 0.700912881 | hypothetical protein |
| CNXL_008830 | CNB00100 | 1.08680261 | 0.001721315 | hypothetical protein |
| CNXL_008840 | CNB00110 | -0.03946128 | 0.958454641 | hypothetical protein |
| CNXL_008850 | CNB00120 | 0.000645247 | 0.998287442 | hypothetical protein |
| CNXL_008860 | | -1.526499937 | 3.55761E-06 | hypothetical protein |
| CNXL_008870 | CNB00130 | -1.513066361 | 6.56548E-06 | hypothetical protein |
| CNXL_008880 | | -0.10375439 | 0.937834503 | hypothetical protein |
| CNXL_008890 | | 0.238920728 | 0.684784071 | hypothetical protein |
| CNXL_008900 | CNB00140 | 0.019132151 | 0.987160302 | hypothetical protein |
| CNXL_008910 | | 0.159053042 | 0.779687006 | hypothetical protein |
| CNXL_008920 | | 0.665680437 | 0.081975892 | hypothetical protein |
| CNXL_008930 | | 0.140008644 | 0.787846251 | hypothetical protein |
| CNXL_008940 | CNB00170 | 0.253738909 | 0.546370656 | hypothetical protein |
| CNXL_008950 | | 0.825519234 | 0.007312416 | hypothetical protein |
| CNXL_008960 | CNB00190 | -0.629784848 | 0.132308784 | hypothetical protein |
| CNXL_008970 | CNB00200 | 0.12175401 | 0.814220979 | hypothetical protein |
| CNXL_008980 | CNB00210 | -0.034159868 | 0.965966884 | hypothetical protein |
| CNXL_008990 | CNB00220 | 0.222458434 | 0.662151848 | serine-threonine kinase receptor-associated protein |
| CNXL_009000 | CNB00230 | 0.049384177 | 0.948087035 | fructosyl amino acid oxidase |
| CNXL_009010 | CNB00240 | -0.364457913 | 0.525959757 | membrane protein |
| CNXL_009020 | CNB00250 | -0.396246856 | 0.459805517 | hypothetical protein |
| CNXL_009030 | | -1.42790091 | 0.017707947 | tRNA |
| CNXL_009040 | CNB00260 | -0.326387286 | 0.478094003 | hypothetical protein |
| CNXL_009050 | CNB00270 | -0.286875121 | 0.657725818 | copii vesicle coat protein |
| CNXL_009060 | CNB00290 | 1.237027578 | 2.04358E-05 | nucleoporin SEH1 |
| CNXL_009070 | CNB00300 | 0.522261307 | 0.071401102 | hypothetical protein |
| CNXL_009080 | | -0.013750505 | 0.991792083 | hypothetical protein |
| CNXL_009090 | CNB00310 | 0.059951464 | 0.936086163 | hypothetical protein |
| CNXL_009100 | CNB00320 | 0.735693972 | 0.406713802 | hypothetical protein |
| CNXL_009110 | CNB00330 | -0.16275319 | 0.735456246 | hypothetical protein |
| CNXL_009120 | CNB00340 | -0.347296272 | 0.463712384 | F-type H+-transporting ATPase subunit delta |
| CNXL_009130 | CNB00360 | -0.262362643 | 0.521158873 | hypothetical protein |
| CNXL_009140 | CNB00370 | 0.240422532 | 0.61372863 | hypothetical protein |
| CNXL_009150 | CNB00380 | -0.236423366 | 0.667369865 | hypothetical protein |
| CNXL_009160 | CNB00390 | -0.016970715 | 0.984913368 | serine-tRNA ligase |
| CNXL_009170 | CNB00400 | 0.084981229 | 0.897991632 | gata transcription factor |
| CNXL_009180 | CNB00410 | -0.059209419 | 0.927441884 | siderophore-iron transporter Str1 |
| CNXL_009190 | | -0.378587367 | 0.764121818 | hypothetical protein |
| CNXL_009200 | CNB00420 | 1.226603614 | 0.000367053 | hypothetical protein |
| CNXL_009210 | CNB00430 | 0.282502235 | 0.574330347 | dehydrogenase |
| CNXL_009220 | | 1.1209117 | 0.000571402 | efflux protein |
| CNXL_009230 | | 0.591511031 | 0.058935438 | hypothetical protein |
| CNXL_009240 | CNB00450 | 0.005574828 | 0.994987997 | hypothetical protein |
| CNXL_009250 | CNB00460 | -0.496312833 | 0.78519633 | pre-rRNA-processing protein ESF2 |
| CNXL_009260 | | -0.566612927 | 0.78519633 | hypothetical protein |

| | | | | |
|---|---|---|---|---|
| CNXL_009270 | CNB00470 | -0.788348748 | 0.290975028 | hypothetical protein |
| CNXL_009280 | CNB00480 | -0.418693093 | 0.244955281 | hypothetical protein |
| CNXL_009290 | CNB00490 | -0.141152645 | 0.787481637 | threonine-tRNA ligase |
| CNXL_009300 | CNB00500 | 0.104110744 | 0.858106401 | RNA polymerase-associated protein CTR9 |
| CNXL_009310 | CNB00510 | 0.077574609 | 0.911130835 | nuclear cap-binding protein subunit 1 |
| CNXL_009320 | CNB00520 | -0.472659983 | 0.232022599 | hypothetical protein |
| CNXL_009330 | CNB00530 | 0.490137012 | 0.216917103 | hypothetical protein |
| CNXL_009340 | CNB00540 | -0.28085899 | 0.612746451 | hypothetical protein |
| CNXL_009350 | CNB00550 | 0.028555364 | 0.965101267 | hypothetical protein |
| CNXL_009360 | CNB00560 | -0.080765155 | 0.884028948 | histone H2A |
| CNXL_009370 | CNB00570 | -0.122073955 | 0.803947851 | histone H2B |
| CNXL_009380 | CNB00580 | -0.082747209 | 0.920669589 | histone H3 |
| CNXL_009390 | CNB00590 | -0.637092334 | 0.108552208 | hypothetical protein |
| CNXL_009400 | CNB00600 | 0.184039817 | 0.703985737 | hypothetical protein |
| CNXL_009410 | CNB00610 | -0.337113711 | 0.48260506 | hypothetical protein |
| CNXL_009420 | CNB00620 | -0.341106706 | 0.505486204 | hypothetical protein |
| CNXL_009430 | CNB00630 | -0.349391326 | 0.570755994 | hypothetical protein |
| CNXL_009440 | CNB00640 | -0.035008446 | 0.965004754 | hypothetical protein |
| CNXL_009450 | CNB00650 | 0.315035242 | 0.432050869 | hypothetical protein |
| CNXL_009460 | CNB00670 | 0.113241053 | 0.868893618 | hypothetical protein |
| CNXL_009470 | CNB00680 | -0.257095811 | 0.649391055 | hypothetical protein |
| CNXL_009480 | CNB00690 | 0.463944625 | 0.279897398 | DNA-directed RNA polymerase I |
| CNXL_009490 | CNB00700 | 0.267723285 | 0.548869305 | 19s proteasome regulatory particle base assembly chaperone |
| CNXL_009500 | CNB00710 | 0.228440658 | 0.753699936 | hypothetical protein |
| CNXL_009510 | CNB00720 | 0.258099533 | 0.527938546 | hypothetical protein |
| CNXL_009520 | CNB00730 | -0.0846512 | 0.944879244 | similar to glycogen synthase kinase |
| CNXL_009530 | CNB00740 | -1.233162926 | 4.22628E-05 | hypothetical protein |
| CNXL_009540 | CNB00750 | -0.095583693 | 0.885831771 | kinesin |
| CNXL_009550 | CNB00770 | 0.005144423 | 0.995735232 | hypothetical protein |
| CNXL_009560 | CNB00780 | -0.117431254 | 0.891760995 | putative chitin synthase regulator |
| CNXL_009570 | CNB00790 | -0.315746746 | 0.62364132 | hypothetical protein |
| CNXL_009580 | CNB00800 | -0.012525267 | 0.987759522 | hypothetical protein |
| CNXL_009590 | CNB00810 | -0.016171229 | 0.987423727 | hypothetical protein |
| CNXL_009600 | CNB00820 | -0.572558826 | 0.170587304 | hypothetical protein |
| CNXL_009610 | CNB00830 | 0.11567467 | 0.857197728 | mbp1/swi4-like transcription factor |
| CNXL_009620 | | 0.234215073 | 0.620329837 | hypothetical protein |
| CNXL_009630 | | -0.081124341 | 0.892724552 | hypothetical protein |
| CNXL_009640 | CNB00850 | -0.218267942 | 0.638378602 | hypothetical protein |
| CNXL_009650 | CNB00860 | -0.088174023 | 0.90384925 | hypothetical protein |
| CNXL_009660 | | -0.174743897 | 0.748179373 | acyl-CoA-dependent ceramide synthase |
| CNXL_009670 | | -0.772284704 | 0.05908683 | hypothetical protein |
| CNXL_009680 | CNB00890 | 0.024112506 | 0.979990369 | hypothetical protein |
| CNXL_009690 | CNB00900 | -0.434901644 | 0.35484081 | endoplasmic reticulum vesicle protein 25 |
| CNXL_009700 | CNB00910 | 0.601192067 | 0.081449041 | cysteine-tRNA ligase |
| CNXL_009710 | CNB00920 | 0.170712686 | 0.779355929 | hypothetical protein |
| CNXL_009720 | CNB00930 | 0.190772936 | 0.742187954 | hypothetical protein |
| CNXL_009730 | CNB00940 | 0.151752441 | 0.811154336 | hypothetical protein |
| CNXL_009740 | CNB00950 | -0.70711943 | 0.124179444 | hypothetical protein |
| CNXL_009750 | CNB00960 | 0.588385688 | 0.112325572 | dCMP deaminase |
| CNXL_009760 | | -1.28357933 | 7.14535E-08 | hypothetical protein |
| CNXL_009770 | CNB00970 | -1.221139787 | 0.000710884 | hypothetical protein |
| CNXL_009780 | CNB00980 | 0.40973412 | 0.314390734 | hypothetical protein |
| CNXL_009790 | CNB00990 | 0.261317114 | 0.549016425 | hypothetical protein |
| CNXL_009800 | CNB01000 | 0.033968634 | 0.965966884 | hypothetical protein |
| CNXL_009810 | CNB01010 | 0.189726514 | 0.719248355 | hypothetical protein |
| CNXL_009820 | CNB01020 | -0.424750599 | 0.248114375 | ubiquilin |
| CNXL_009830 | CNB01030 | -2.330684691 | 2.63261E-18 | mandelate racemase/muconate lactonizing enzyme |
| CNXL_009840 | CNB01040 | -0.142207009 | 0.814220979 | putative pheromone transporter |

| | | | | |
|---|---|---|---|---|
| CNXL_009850 | CNB01050 | -0.125632422 | 0.80500309 | hypothetical protein |
| CNXL_009860 | CNB01060 | -0.057011458 | 0.939400455 | U3 small nucleolar RNA-associated protein 5 |
| CNXL_009870 | CNB01070 | 0.196814724 | 0.678724355 | ATP-dependent RNA helicase HAS1 |
| CNXL_009880 | CNB01080 | -0.21659687 | 0.754070285 | mRNA 3'-end-processing protein YTH1 |
| CNXL_009890 | CNB01090 | -0.512145342 | 0.18027341 | hypothetical protein |
| CNXL_009900 | CNB01100 | -0.016183565 | 0.990008547 | hypothetical protein |
| CNXL_009910 | CNB01110 | 0.377255589 | 0.402210452 | hypothetical protein |
| CNXL_009920 | CNB01120 | 0.770591574 | 0.02554521 | hypothetical protein |
| CNXL_009930 | CNB01130 | 0.424183496 | 0.277600586 | hypothetical protein |
| CNXL_009940 | CNB01140 | -0.382492928 | 0.348883302 | hypothetical protein |
| CNXL_009950 | CNB01150 | -0.222032737 | 0.677970373 | hypothetical protein |
| CNXL_009960 | CNB01160 | 0.209828064 | 0.746537956 | threonine synthase |
| CNXL_009970 | CNB01170 | 0.225053986 | 0.618868301 | hypothetical protein |
| CNXL_009980 | CNB01190 | -0.705583458 | 0.488222343 | phosphodiesterase |
| CNXL_009990 | CNB01200 | 0.109878717 | 0.85039879 | hypothetical protein |
| CNXL_010000 | CNB01210 | -0.033394115 | 0.970404228 | NADPH2:quinone reductase |
| CNXL_010010 | CNB01220 | -0.437097045 | 0.365333703 | hypothetical protein |
| CNXL_010020 | CNB01230 | 0.11689982 | 0.819551095 | hypothetical protein |
| CNXL_010030 | CNB01240 | -0.409787985 | 0.393343978 | cyclophilin A |
| CNXL_010040 | | 0.051799975 | 0.949641665 | ram signaling network cell polarity protein |
| CNXL_010050 | CNB01250 | -0.139883262 | 0.808366697 | hypothetical protein |
| CNXL_010060 | CNB01260 | -0.110018005 | 0.855455189 | hypothetical protein |
| CNXL_010070 | CNB01270 | 0.034704206 | 0.965101267 | pescadillo |
| CNXL_010080 | CNB01280 | -0.180564807 | 0.761797019 | hypothetical protein |
| CNXL_010090 | CNB01290 | 0.303361365 | 0.445982809 | actin cross-linking protein |
| CNXL_010100 | CNB01300 | 0.354008069 | 0.417588807 | cyclophilin A |
| CNXL_010110 | CNB01310 | -0.237495185 | 0.596226424 | hypothetical protein |
| CNXL_010120 | CNB01320 | -2.208793543 | 0.014472633 | NADH dehydrogenase |
| CNXL_010130 | CNB01330 | 0.781452579 | 0.019855885 | hypothetical protein |
| CNXL_010140 | CNB01340 | 0.572397499 | 0.172738218 | hypothetical protein |
| CNXL_010150 | CNB01350 | 0.068978225 | 0.92988625 | hypothetical protein |
| CNXL_010160 | CNB01360 | -0.325006461 | 0.544657787 | hypothetical protein |
| CNXL_010170 | | -3.218604384 | 8.31691E-29 | DNA-directed RNA polymerase I subunit RPA49 |
| CNXL_010180 | CNB01370 | -0.825668849 | 0.005277971 | hypothetical protein |
| CNXL_010190 | CNB01380 | 0.760383718 | 0.057184294 | DNA-directed RNA polymerase I and III subunit RPAC2 |
| CNXL_010200 | CNB01390 | 0.60312687 | 0.090247305 | ATP-dependent DNA helicase |
| CNXL_010210 | CNB01400 | 0.483727712 | 0.195436707 | heterokaryon incompatibility protein HET-C |
| CNXL_010220 | CNB01410 | 0.044647852 | 0.956449152 | hypothetical protein |
| CNXL_010230 | | 0.117862149 | 0.865225322 | hypothetical protein |
| CNXL_010240 | CNB01420 | -0.360347236 | 0.313087832 | hypothetical protein |
| CNXL_010250 | | 0.294126597 | 0.683247689 | hypothetical protein |
| CNXL_010260 | | 0.292699331 | 0.593883843 | hypothetical protein |
| CNXL_010270 | CNB01440 | -0.139839369 | 0.798191388 | hypothetical protein |
| CNXL_010280 | CNB01450 | -0.570644969 | 0.143124598 | capsule related protein |
| CNXL_010290 | CNB01460 | 0.066182486 | 0.92070335 | NET1-associated nuclear protein 1 |
| CNXL_010300 | CNB01470 | -0.326517542 | 0.573586726 | hypothetical protein |
| CNXL_010310 | CNB01480 | -0.011603116 | 0.988662258 | hypothetical protein |
| CNXL_010320 | CNB01500 | 0.115745141 | 0.877313027 | UDP-glucose:glycoprotein glucosyltransferase |
| CNXL_010330 | CNB01510 | -0.844286963 | 0.084687783 | hypothetical protein |
| CNXL_010340 | CNB01520 | 0.041941097 | 0.95086529 | hypothetical protein |
| CNXL_010350 | CNB01530 | 0.064201132 | 0.937834503 | syntaxin 7 |
| CNXL_010360 | | -0.065124483 | 0.9274899 | dynactin 6 |
| CNXL_010370 | CNB01550 | -0.533463876 | 0.203820014 | hypothetical protein |
| CNXL_010380 | CNB01560 | -1.429076109 | 2.48089E-05 | ATP-dependent DNA helicase |
| CNXL_010390 | | 0.393246826 | 0.613291613 | hypothetical protein |
| CNXL_010400 | CNB01580 | -0.729680086 | 0.098243449 | hypothetical protein |
| CNXL_010410 | | -0.080573707 | 0.926407741 | hypothetical protein |
| CNXL_010420 | CNB01590 | 0.313727181 | 0.551883314 | hypothetical protein |

| | | | | |
|---|---|---|---|---|
| CNXL_010430 | CNB01600 | 0.650485816 | 0.045818153 | DNA replication regulator DPB11 |
| CNXL_010440 | | 0.204566522 | 0.761312549 | lipoate-protein ligase A |
| CNXL_010450 | CNB01610 | 0.749353481 | 0.024632712 | hypothetical protein |
| CNXL_010460 | CNB01620 | 0.309356077 | 0.51244137 | hypothetical protein |
| CNXL_010470 | CNB01630 | 0.31404137 | 0.383471707 | L-lactate dehydrogenase |
| CNXL_010480 | CNB01640 | 0.32838249 | 0.445982809 | putative nickel transporter |
| CNXL_010490 | CNB01650 | 0.134924348 | 0.807491917 | ATP-dependent RNA helicase DDX35 |
| CNXL_010500 | CNB01660 | 1.279123349 | 0.000236599 | acyl-CoA dehydrogenase |
| CNXL_010510 | CNB01670 | 0.262549985 | 0.573586726 | hypothetical protein |
| CNXL_010520 | CNB01680 | -0.585021953 | 0.072794568 | hypothetical protein |
| CNXL_010530 | CNB01690 | -0.522829338 | 0.152050804 | hypothetical protein |
| CNXL_010540 | CNB01700 | 0.068472791 | 0.965004754 | serine/threonine-protein kinase |
| CNXL_010550 | CNB01710 | 0.105314951 | 0.853018168 | hypothetical protein |
| CNXL_010560 | CNB01720 | -0.191808756 | 0.76831241 | BET3 family protein |
| CNXL_010570 | CNB01730 | -0.047850995 | 0.946116137 | serine/threonine-protein phosphatase PP-Z1 |
| CNXL_010580 | CNB01740 | -0.137919032 | 0.818704891 | oxoglutarate dehydrogenase |
| CNXL_010590 | CNB01750 | 0.772502529 | 0.012698458 | pumilio domain-containing protein |
| CNXL_010600 | CNB01760 | 1.01874683 | 0.001160289 | D-lactate dehydrogenase |
| CNXL_010610 | CNB01770 | 0.209164744 | 0.675457822 | hypothetical protein |
| CNXL_010620 | | 1.652016587 | 8.71596E-09 | hypothetical protein |
| CNXL_010630 | | 0.216207151 | 0.733041323 | hypothetical protein |
| CNXL_010640 | NB01780\|CNB017 | 0.188187271 | 0.671905355 | Unknown |
| CNXL_010650 | CNB01800 | 0.08642104 | 0.874670769 | Unknown |
| CNXL_010660 | CNB01810 | 0.117857912 | 0.833643586 | fk506-binding protein |
| CNXL_010670 | CNB01820 | 0.565809194 | 0.068722808 | short-chain dehydrogenase/reductase SDR |
| CNXL_010680 | CNB01830 | 0.35843506 | 0.404910404 | ATP-binding cassette |
| CNXL_010690 | | 0.385509323 | 0.361761993 | hypothetical protein |
| CNXL_010700 | CNB01850 | 0.204904546 | 0.681669987 | Unknown |
| CNXL_010710 | | 0.66640502 | 0.057135304 | hypothetical protein |
| CNXL_010720 | | 0.952472245 | 0.013896463 | hypothetical protein |
| CNXL_010730 | | 0.141034538 | 0.841905236 | Unknown |
| CNXL_010740 | CNB01870 | 0.559826042 | 0.287017678 | Unknown |
| CNXL_010750 | CNB01880 | -0.098444342 | 0.86171371 | zf-C3HC4 type zinc finger protein |
| CNXL_010760 | CNB01890 | -0.071432981 | 0.918689896 | valine-tRNA ligase |
| CNXL_010770 | CNB01900 | 0.114653302 | 0.881726613 | AP-2 complex subunit mu-1 |
| CNXL_010780 | CNB01910 | -0.054962524 | 0.948275601 | hypothetical protein |
| CNXL_010790 | CNB01920 | 0.006465039 | 0.993094074 | large subunit ribosomal protein L11 |
| CNXL_010800 | | 0.033926722 | 0.971783504 | iron permease |
| CNXL_010810 | CNB01940 | 0.604420728 | 0.213413887 | probable sugar phosphate/phosphate translocator |
| CNXL_010820 | CNB01950 | -0.182699125 | 0.746765994 | hypothetical protein |
| CNXL_010830 | CNB01960 | -0.113874435 | 0.853287691 | hormone-sensitive lipase |
| CNXL_010840 | CNB01970 | -0.472319843 | 0.143932072 | E3 ubiquitin-protein ligase UBR7 |
| CNXL_010850 | CNB01980 | 0.31861783 | 0.49958231 | hypothetical protein |
| CNXL_010860 | CNB01990 | -0.326162698 | 0.452146814 | hypothetical protein |
| CNXL_010870 | CNB02000 | -0.181875581 | 0.803833694 | 3-phosphoshikimate 1-carboxyvinyltransferase |
| CNXL_010880 | | -1.448670673 | 1.92549E-07 | tubulin-folding cofactor B |
| CNXL_010890 | CNB02020 | 1.080232625 | 0.001744662 | hypothetical protein |
| CNXL_010900 | CNB02030 | 0.089915888 | 0.870049852 | hypothetical protein |
| CNXL_010910 | CNB02040 | -0.415998502 | 0.312497815 | serine/threonine-protein phosphatase PP1 |
| CNXL_010920 | NB02050\|CNB020 | -0.381803693 | 0.276952897 | efflux protein |
| CNXL_010930 | CNB02070 | -0.404170303 | 0.447817937 | Unknown |
| CNXL_010940 | CNB02080 | -0.273147929 | 0.616439959 | hypothetical protein |
| CNXL_010950 | CNB02090 | 0.206311759 | 0.716771972 | oligosaccharidyl-lipid flippase |
| CNXL_010960 | CNB02100 | 0.120810361 | 0.847311574 | hypothetical protein |
| CNXL_010970 | CNB02110 | 0.04268513 | 0.95086529 | efflux protein EncT |
| CNXL_010980 | CNB02120 | -0.154496653 | 0.788599237 | microtubule motor protein |
| CNXL_010990 | CNB02130 | -0.036523145 | 0.978217372 | hypothetical protein |
| CNXL_011000 | | 0.007251947 | 0.996158838 | pr4/barwin domain protein |

| | | | | |
|---|---|---|---|---|
| CNXL_011010 | | 0.076781292 | 0.936507583 | Unknown |
| CNXL_011020 | CNB02140 | 0.243699637 | 0.680746566 | Unknown |
| CNXL_011030 | CNB02150 | -0.142982829 | 0.783220222 | mitochondrial intermediate peptidase 2 |
| CNXL_011040 | CNB02160 | 0.270987784 | 0.797182044 | Unknown |
| CNXL_011050 | CNB02170 | 0.372954879 | 0.35683476 | Unknown |
| CNXL_011060 | CNB02180 | -0.017195939 | 0.986381348 | Putative transporter of the major facilitator superfamily |
| CNXL_011070 | CNB02190 | 0.15321123 | 0.78519633 | D-tyrosyl-tRNA |
| CNXL_011080 | CNB02200 | -0.06853969 | 0.91726998 | 26S proteasome regulatory subunit N12 |
| CNXL_011090 | CNB02210 | 0.064900754 | 0.927745648 | RAN protein binding protein |
| CNXL_011100 | CNB02220 | -0.363336287 | 0.346750302 | nuclear protein |
| CNXL_011110 | CNB02230 | 0.011506489 | 0.988641003 | ribosomal RNA methyltransferase Nop2 |
| CNXL_011120 | CNB02240 | 0.05116869 | 0.936783812 | dTDP-4-dehydrorhamnose reductase |
| CNXL_011130 | CNB02250 | -0.162153057 | 0.779355929 | hypothetical protein |
| CNXL_011140 | CNB02260 | 1.193173191 | 0.000915689 | hypothetical protein |
| CNXL_011150 | CNB02270 | 0.153582832 | 0.840398313 | hypothetical protein |
| CNXL_011160 | CNB02280 | -0.075668327 | 0.914338536 | peroxin-5 |
| CNXL_011170 | CNB02290 | 0.830770087 | 0.00176548 | DNA-directed RNA polymerase II subunit RPB11 |
| CNXL_011180 | CNB02300 | 0.140619754 | 0.812703117 | hypothetical protein |
| CNXL_011190 | CNB02310 | -1.224056447 | 0.000473348 | integral membrane protein |
| CNXL_011200 | CNB02320 | 0.013556591 | 0.988641003 | hypothetical protein |
| CNXL_011210 | CNB02330 | -0.425678352 | 0.451211894 | hypothetical protein |
| CNXL_011220 | CNB02340 | -1.507879436 | 9.43932E-08 | beta-1 |
| CNXL_011230 | CNB02350 | 0.572793766 | 0.095737732 | hypothetical protein |
| CNXL_011240 | CNB02360 | -0.09003659 | 0.868129828 | pantetheine-phosphate adenylyltransferase |
| CNXL_011250 | | 0.651344448 | 0.365333703 | large subunit ribosomal protein L10-like |
| CNXL_011260 | CNB02370 | -0.633376854 | 0.865225322 | hypothetical protein |
| CNXL_011270 | CNB02380 | -0.035787538 | 0.959808031 | hypothetical protein |
| CNXL_011280 | CNB02390 | -0.189292168 | 0.779355929 | staphylococcal nuclease domain-containing protein 1 |
| CNXL_011290 | CNB02400 | 0.173299558 | 0.911130835 | glutamine amidotransferase |
| CNXL_011300 | CNB02410 | 0.337320631 | 0.529965977 | hypothetical protein |
| CNXL_011310 | CNB02420 | -0.172647107 | 0.721253882 | hypothetical protein |
| CNXL_011320 | CNB02430 | -0.212136158 | 0.699223686 | large subunit ribosomal protein L27Ae |
| CNXL_011330 | CNB02440 | 0.237637111 | 0.716440724 | large subunit ribosomal protein L19 |
| CNXL_011340 | CNB02450 | 0.187797523 | 0.806721847 | hypothetical protein |
| CNXL_011350 | CNB02460 | 0.219147584 | 0.645131211 | hypothetical protein |
| CNXL_011360 | CNB02470 | -0.353134759 | 0.538910254 | calcium channel protein |
| CNXL_011370 | CNB02480 | -0.16319223 | 0.776125901 | hypothetical protein |
| CNXL_011380 | CNB02490 | 0.78942537 | 0.388091044 | dolichol-phosphate mannosyltransferase |
| CNXL_011390 | | -0.477636754 | 0.35648622 | short-chain dehydrogenase/reductase SDR |
| CNXL_011400 | CNB02510 | 0.10745375 | 0.875938406 | hypothetical protein |
| CNXL_011410 | CNB02520 | -1.330752087 | NA | hypothetical protein |
| CNXL_011420 | CND05880 | 0.494273082 | 0.38937973 | Unknown |
| CNXL_011430 | CNB02530 | -0.430823134 | 0.32189769 | Unknown |
| CNXL_011440 | CNB02540 | 0.942910144 | 0.012339766 | hypothetical protein |
| CNXL_011450 | | 0.790227555 | 0.015027277 | Unknown |
| CNXL_011460 | CNB02550 | 0.271504137 | 0.603329126 | Unknown |
| CNXL_011470 | CNB02560 | -0.332562659 | 0.479536106 | Rad50-interacting protein 1 homolog |
| CNXL_011480 | CNB02570 | -0.37714092 | 0.372306093 | mRNA 3'-end-processing protein RNA14 |
| CNXL_011490 | | -1.271508914 | 0.015219211 | AdoMet-dependent rRNA methyltransferase SPB1 |
| CNXL_011500 | | -0.408179362 | 0.313918019 | Unknown |
| CNXL_011510 | | 0.055640505 | 0.950831293 | IQ domain-containing GTPase activating protein |
| CNXL_011520 | CNB02600 | -0.458957879 | 0.443600568 | Unknown |
| CNXL_011530 | CNB02610 | 0.232348891 | 0.630229087 | integral membrane protein |
| CNXL_011540 | CNB02620 | -0.399494145 | 0.312160498 | trehalose-6-phophate phosphatase |
| CNXL_011550 | | -0.5716022 | 0.486787083 | ankyrin repeat-containing protein |
| CNXL_011560 | CNB02640 | -0.141324503 | 0.802313068 | Unknown |
| CNXL_011570 | CNB02650 | -0.448873212 | 0.260431503 | cohesin complex subunit psm1 |
| CNXL_011580 | CNB02660 | 0.579669741 | 0.081975892 | hypothetical protein |

| | | | | |
|---|---|---|---|---|
| CNXL_011590 | | 0.143872258 | 0.833396996 | hexokinase |
| CNXL_011600 | CNB02670 | 0.992253396 | 0.003439034 | hypothetical protein |
| CNXL_011610 | CNB02680 | 0.108264356 | 0.857811508 | DNA binding protein Ncp1 |
| CNXL_011620 | CNB02690 | 0.279484001 | 0.498897935 | high-affinity glucose transporter |
| CNXL_011630 | CNB02700 | 0.062357836 | 0.918701468 | hypothetical protein |
| CNXL_011640 | CNB02710 | 0.377757771 | 0.427699195 | hypothetical protein |
| CNXL_011650 | CNB02720 | 0.467910321 | 0.186852071 | hypothetical protein |
| CNXL_011660 | | 0.254017855 | 0.916894829 | hypothetical protein |
| CNXL_011670 | CNB02730 | 0.528000559 | 0.13443672 | hypothetical protein |
| CNXL_011680 | CNB02740 | -0.225692473 | 0.787481637 | hypothetical protein |
| CNXL_011690 | CNB02750 | -0.247485092 | 0.607065672 | large subunit ribosomal protein L4 |
| CNXL_011700 | CNB02760 | -0.631568011 | 0.126171266 | small subunit ribosomal protein S16 |
| CNXL_011710 | CNB02770 | 0.724437455 | 0.071289176 | hypothetical protein |
| CNXL_011720 | | 0.530978768 | 0.227067905 | hypothetical protein |
| CNXL_011730 | | 0.426499376 | 0.544868122 | Unknown |
| CNXL_011740 | CNB02790 | 0.342574332 | 0.320878752 | hypothetical protein |
| CNXL_011750 | | 0.280704518 | 0.63756045 | putative trehalase |
| CNXL_011760 | CNB02810 | -0.629219077 | 0.015554314 | Unknown |
| CNXL_011770 | CNB02820 | -0.269988876 | 0.583718777 | tubulin alpha-1A chain |
| CNXL_011780 | CNB02830 | -0.172505431 | 0.733468304 | hypothetical protein |
| CNXL_011790 | CNB02840 | -0.126343504 | 0.814220979 | mRNA turnover protein 4 |
| CNXL_011800 | CNB02850 | -0.356742626 | 0.510006134 | hypothetical protein |
| CNXL_011810 | CNB02860 | 0.030304177 | 0.976545574 | CAMKK/ELM protein kinase |
| CNXL_011820 | CNB02870 | 0.087918198 | 0.884028948 | tRNA adenylyltransferase |
| CNXL_011830 | CNB02880 | 1.237890807 | 0.000150476 | hypothetical protein |
| CNXL_011840 | CNB02890 | 0.093884788 | 0.891600898 | endoplasmic reticulum protein |
| CNXL_011850 | CNB02900 | 0.34809127 | 0.382997689 | N-glycosylase/DNA lyase |
| CNXL_011860 | | -0.728151358 | 0.742187954 | NAK protein kinase |
| CNXL_011870 | | 0.19378278 | 0.741662589 | Unknown |
| CNXL_011880 | | 0.931645883 | 0.802982005 | hypothetical protein |
| CNXL_011890 | | 0.171437661 | NA | Unknown |
| CNXL_011900 | CNA03700 | 0.074112102 | NA | Unknown |
| CNXL_011910 | | -2.364417376 | NA | Unknown |
| CNXL_011920 | CNB02940 | -1.440226686 | NA | Unknown |
| CNXL_011930 | CNB02980 | 0.558046217 | 0.146688128 | Unknown |
| CNXL_011940 | CNB02990 | 0.576666972 | 0.083255499 | calcium/proton exchanger |
| CNXL_011950 | | 0.689915511 | 0.024690962 | acyltransferase |
| CNXL_011960 | | 0.298278304 | 0.658936841 | E3 ubiquitin-protein ligase CCNP1IP1 |
| CNXL_011970 | CNB03000 | 0.186192745 | 0.652704639 | Unknown |
| CNXL_011980 | | 0.046010937 | 0.955507961 | aromatic amino acid aminotransferase I |
| CNXL_011990 | | 0.100934165 | 0.926523266 | Unknown |
| CNXL_012000 | CNB03010 | 0.220473205 | 0.634323515 | Unknown |
| CNXL_012010 | | -0.861215334 | 0.008634381 | large subunit ribosomal protein L13 |
| CNXL_012020 | CNB03020 | -0.908954246 | 0.001201403 | Unknown |
| CNXL_012030 | CNB03030 | 0.104070624 | 0.874890451 | AGC/YANK protein kinase |
| CNXL_012040 | CNB03040 | 0.260031749 | 0.540113713 | histidinol-phosphate transaminase |
| CNXL_012050 | CNB03050 | -0.086810293 | 0.879531297 | replication factor A3 |
| CNXL_012060 | CNB03060 | -0.369529315 | 0.443530973 | small nuclear ribonucleoprotein Sm D2 |
| CNXL_012070 | CNB03070 | -0.566276134 | 0.063005266 | hypothetical protein |
| CNXL_012080 | CNB03080 | -0.177404735 | 0.787846251 | 20S proteasome subunit beta 1 |
| CNXL_012090 | CNB03090 | -0.278732535 | 0.615328522 | cell growth-regulating nucleolar protein |
| CNXL_012100 | CNB03100 | 0.190655483 | 0.703818084 | two-component system response regulator |
| CNXL_012110 | | -0.192271982 | 0.78519633 | sterol 24-C-methyltransferase |
| CNXL_012120 | CNB03110 | -0.138721525 | 0.78519633 | Unknown |
| CNXL_012130 | CNB03120 | 0.098267276 | 0.889757615 | nucleolar protein 14 |
| CNXL_012140 | CNB03130 | -0.412670879 | 0.630279293 | phosphatidylinositol 3-kinase |
| CNXL_012150 | CNB03140 | 0.050787676 | 0.936783812 | monolysocardiolipin acyltransferase |
| CNXL_012160 | CNB03150 | 1.254281723 | 0.000382843 | ubiquitin ligase complex ring-box protein |

| | | | | |
|---|---|---|---|---|
| CNXL_012170 | | -3.446152406 | 6.53643E-43 | solute carrier family 25 |
| CNXL_012180 | CNB03160 | 0.063873994 | 0.918756814 | hypothetical protein |
| CNXL_012190 | CNB03170 | -0.091852412 | 0.884028948 | histone acetyltransferase ESA1 |
| CNXL_012200 | CNB03180 | 0.326843872 | 0.445982809 | hypothetical protein |
| CNXL_012210 | CNB03190 | -0.131957034 | 0.816572532 | aromatic amino acid aminotransferase I |
| CNXL_012220 | | -3.039653284 | 2.73942E-37 | L-carnitine dehydratase/bile acid-inducible protein F |
| CNXL_012230 | CNB03200 | -1.330752087 | NA | hypothetical protein |
| CNXL_012240 | CNB03210 | -0.061075911 | 0.932765996 | hypothetical protein |
| CNXL_012250 | | -0.320697847 | 0.697785609 | alpha 1 |
| CNXL_012260 | | 0.157359091 | 0.814730279 | hypothetical protein |
| CNXL_012270 | CNB03230 | -2.103157672 | 2.42249E-11 | Unknown |
| CNXL_012280 | | 0.228339936 | 0.6409676 | C4-hydroxylase |
| CNXL_012290 | CNB03240 | 0.185503731 | 0.703985737 | DASH complex subunit DAD3 |
| CNXL_012300 | CNB03250 | 0.781701026 | 0.004070183 | hypothetical protein |
| CNXL_012310 | NB03260\|CNB032' | 0.337337062 | 0.331567493 | protein-L-isoaspartate O-methyltransferase |
| CNXL_012320 | CNB03280 | -0.312978251 | 0.483451258 | hypothetical protein |
| CNXL_012330 | CNB03290 | 0.298480819 | 0.544657787 | CCR4-NOT transcription complex subunit 4 |
| CNXL_012340 | CNB03300 | -0.401836596 | 0.258852606 | pre-mRNA-splicing factor CWC26 |
| CNXL_012350 | | 0.366981347 | 0.636723305 | hypothetical protein |
| CNXL_012360 | CNB03330 | -0.092234241 | 0.902044607 | hypothetical protein |
| CNXL_012370 | CNB03340 | 0.050325107 | 0.944879244 | NAK protein kinase |
| CNXL_012380 | CNB03350 | 0.092573961 | 0.907984995 | nonselective cation channel protein |
| CNXL_012390 | CNB03360 | -0.235067282 | 0.633920399 | multidrug resistance transporter |
| CNXL_012400 | CNB03370 | 0.755225829 | 0.048775287 | vacuolar membrane protein |
| CNXL_012410 | CNB03380 | -0.875958968 | 0.003852158 | hypothetical protein |
| CNXL_012420 | CNB03390 | -0.426163795 | 0.233087998 | glutathione transferase |
| CNXL_012430 | CNB03400 | -0.152129078 | 0.793711563 | nuclear protein |
| CNXL_012440 | CNB03420 | -0.127572223 | 0.871477499 | hypothetical protein |
| CNXL_012450 | CNB03430 | 0.121372891 | 0.817058875 | general transcription factor 3C polypeptide 5 |
| CNXL_012460 | CNB03440 | -0.553634541 | 0.120932459 | small copII coat GTPase |
| CNXL_012470 | | 0.117660511 | 0.909451569 | lysine decarboxylase |
| CNXL_012480 | CNB03460 | -2.202381284 | 1.86639E-26 | Unknown |
| CNXL_012490 | CNB03470 | -2.139630735 | 1.59003E-18 | hypothetical protein |
| CNXL_012500 | CNB03490 | -0.250508885 | 0.563181769 | hypothetical protein |
| CNXL_012510 | CNB03500 | 0.058450061 | 0.920669589 | transcriptional regulator Medusa |
| CNXL_012520 | CNB03510 | 0.067934572 | 0.918006109 | cytochrome c oxidase subunit 5a |
| CNXL_012530 | CNB03520 | -0.417129505 | 0.21653299 | hypothetical protein |
| CNXL_012540 | CNB03530 | 0.555999121 | 0.105763986 | protein arginine N-methyltransferase 1 |
| CNXL_012550 | CNB03540 | 1.135628502 | 0.000360004 | hypothetical protein |
| CNXL_012560 | CNB03550 | -0.249601147 | 0.634148092 | cytoplasmic protein |
| CNXL_012570 | | -0.024695532 | 0.986620478 | solute carrier family 32 |
| CNXL_012580 | CNB03570 | 0.234074697 | 0.634323515 | hypothetical protein |
| CNXL_012590 | | 0.404584615 | 0.35455944 | hypothetical protein |
| CNXL_012600 | CNB03580 | -0.216891453 | 0.733117842 | hypothetical protein |
| CNXL_012610 | CNB03590 | -0.318850738 | 0.527018595 | hypothetical protein |
| CNXL_012620 | CNB03600 | 0.394338741 | 0.314390734 | delta8-fatty-acid desaturase |
| CNXL_012630 | CNB03620 | -0.15520146 | 0.799344206 | phosphotyrosine protein phosphatase |
| CNXL_012640 | CNB03630 | 1.286799396 | 0.237718322 | ubiquitin carboxyl-terminal hydrolase |
| CNXL_012650 | CNB03640 | -0.379072575 | 0.597848423 | hypothetical protein |
| CNXL_012660 | CNB03650 | 0.532757958 | 0.08509133 | oxidoreductase |
| CNXL_012670 | NB03660\|CNB036' | 0.641339109 | 0.02332575 | galactose-1-phosphate uridylyltransferase |
| CNXL_012680 | CNB03680 | -0.480930047 | 0.233640619 | Unknown |
| CNXL_012690 | CNB03690 | 0.72838932 | 0.027559659 | hypothetical protein |
| CNXL_012700 | CNB03700 | 0.294533524 | 0.570755994 | SCF-associated factor 1 |
| CNXL_012710 | CNB03710 | 1.166973685 | 0.000112502 | DNA polymerase zeta subunit |
| CNXL_012720 | CNB03720 | 0.038883584 | 0.955507961 | hypothetical protein |
| CNXL_012730 | CNB03730 | -0.316000589 | 0.493046043 | hypothetical protein |
| CNXL_012740 | CNB03740 | 0.041928526 | 0.954513975 | hypothetical protein |

| | | | | |
|---|---|---|---|---|
| CNXL_012750 | CNB03750 | -0.364183112 | 0.477835242 | cytoplasmic protein |
| CNXL_012760 | | 0.250812217 | 0.632776905 | hypothetical protein |
| CNXL_012770 | CNB03760 | -0.019912575 | 0.981569994 | hypothetical protein |
| CNXL_012780 | CNB03770 | 0.554925604 | 0.196920936 | hypothetical protein |
| CNXL_012790 | CNB03780 | 0.046905499 | 0.95265881 | hypothetical protein |
| CNXL_012800 | CNB03790 | -0.246762091 | 0.540948602 | protein lysine methyltransferase SET5 |
| CNXL_012810 | | -0.517586251 | 0.137569503 | hsp60-like protein |
| CNXL_012820 | CNB03810 | -0.013130916 | 0.990703091 | chaperonin GroES |
| CNXL_012830 | CNB03820 | -0.108678322 | 0.857811508 | MAP kinase phosphatase |
| CNXL_012840 | | 0.322208075 | 0.53966149 | Zn2-Cys6 zinc-finger transcription factor |
| CNXL_012850 | CNB03850 | -0.230688226 | 0.632998683 | Unknown |
| CNXL_012860 | CNB03860 | 0.11983388 | 0.881169328 | protein BCP1 |
| CNXL_012870 | CNB03870 | -0.276331384 | 0.606726524 | phosphoadenosine phosphosulfate reductase |
| CNXL_012880 | CNB03880 | 0.148381023 | 0.793332959 | hypoxia up-regulated 1 |
| CNXL_012890 | CNB03890 | 0.078763723 | 0.919145053 | f-actin-capping protein subunit beta |
| CNXL_012900 | CNB03900 | -0.349880753 | 0.402220139 | magnesium transporter |
| CNXL_012910 | CNB03920 | -0.435722513 | 0.337036418 | transcriptional regulatory protein |
| CNXL_012920 | CNB03930 | 0.109735672 | 0.875696601 | hypothetical protein |
| CNXL_012930 | CNB03940 | -0.024426378 | 0.97925254 | 26S protease regulatory subunit 6B |
| CNXL_012940 | | -0.197774328 | 0.799344206 | hypothetical protein |
| CNXL_012950 | CNB03950 | -2.302539927 | 2.38757E-13 | hypothetical protein |
| CNXL_012960 | CNB03960 | -0.309363923 | 0.523679914 | LIM domain containing protein |
| CNXL_012970 | CNB03970 | -0.228621768 | 0.656993267 | WD-repeat protein |
| CNXL_012980 | CNB03980 | 0.900142961 | 0.015092651 | hypothetical protein |
| CNXL_012990 | CNB04010 | -0.764194978 | 0.056639519 | myo-inositol transporter |
| CNXL_013000 | CNB04020 | 0.147043578 | 0.874337207 | hydrolase |
| CNXL_013010 | CNB04030 | -0.557640667 | 0.083397364 | membrane protein |
| CNXL_013020 | CNB04040 | -0.396602542 | 0.399675058 | hypothetical protein |
| CNXL_013030 | CNB04050 | 0.150177539 | 0.756597645 | hypothetical protein |
| CNXL_013040 | CNB04060 | -0.155582672 | 0.804909036 | glucose-6-phosphate isomerase |
| CNXL_013050 | CNB04070 | -0.437452861 | 0.322555045 | nuclear pore complex protein |
| CNXL_013060 | CNB04080 | 0.182859858 | 0.787481637 | ram signaling network protein |
| CNXL_013070 | CNB04100 | -0.091401863 | 0.889001755 | acetate non-utilizing protein 9 |
| CNXL_013080 | CNB04110 | 0.254935504 | 0.619100215 | hypothetical protein |
| CNXL_013090 | CNB04120 | -0.005209757 | 0.996374193 | hypothetical protein |
| CNXL_013100 | CNB04130 | 0.191568797 | 0.789040329 | crossover junction endonuclease MUS81 |
| CNXL_013110 | CNB04140 | 0.241189764 | 0.676934482 | nit protein 1 |
| CNXL_013120 | CNB04150 | -0.084932568 | 0.884028948 | mitochondrial inner membrane protease subunit 2 |
| CNXL_013130 | CNB04160 | 0.009252724 | 0.991690032 | methionine aminopeptidase |
| CNXL_013140 | CNB04170 | -0.271043774 | 0.57573283 | hypothetical protein |
| CNXL_013150 | CNB04180 | -0.030887979 | 0.97076745 | adaptor protein complex AP-1 |
| CNXL_013160 | | 0.224466017 | 0.646850757 | putative sugar transporter |
| CNXL_013170 | CNB04190 | -0.039202057 | 0.95265881 | hypothetical protein |
| CNXL_013180 | CNB04200 | 0.074585852 | 0.918848255 | mitochondrial import receptor subunit TOM40 |
| CNXL_013190 | | 0.173392952 | 0.77767171 | cell cycle checkpoint protein |
| CNXL_013200 | CNB04220 | 0.16978645 | 0.764177425 | transmembrane protein |
| CNXL_013210 | CNB04240 | 0.116470332 | 0.817433984 | similar to Neprilysin |
| CNXL_013220 | | 1.074543424 | 0.000455937 | NAD |
| CNXL_013230 | CNB04250 | -3.309978698 | 2.32341E-39 | hypothetical protein |
| CNXL_013240 | CNB04260 | 0.262967466 | 0.592736437 | hypothetical protein |
| CNXL_013250 | CNB04270 | -0.180858338 | 0.784651013 | 5-aminolevulinic acid synthase |
| CNXL_013260 | CNB04280 | -0.244254594 | 0.603329126 | elongator complex protein 2 |
| CNXL_013270 | CNB04290 | -0.330203507 | 0.498298574 | 20S proteasome subunit alpha 2 |
| CNXL_013280 | CNB04300 | -0.138270464 | 0.802958215 | THO complex subunit 2 |
| CNXL_013290 | CNB04310 | -0.595105941 | 0.048447072 | membrane protein |
| CNXL_013300 | CNB04320 | -0.264550395 | 0.746347427 | chaperone regulator |
| CNXL_013310 | CNB04330 | 0.255021087 | 0.626273098 | hypothetical protein |
| CNXL_013320 | NB04340\|CNB043 | 0.252914462 | 0.550631543 | galactokinase |

| | | | | |
|---|---|---|---|---|
| CNXL_013330 | CNB04360 | -0.355104928 | 0.432050869 | Unknown |
| CNXL_013340 | CNB04370 | -0.258419994 | 0.597047638 | 4-nitrophenyl phosphatase |
| CNXL_013350 | CNB04380 | -0.50479477 | 0.163394194 | transcription elongation factor SPT6 |
| CNXL_013360 | CNB04400 | 0.119636593 | 0.820111382 | hypothetical protein |
| CNXL_013370 | CNB04410 | 0.071713637 | 0.918375149 | hypothetical protein |
| CNXL_013380 | CNB04420 | -0.144982951 | 0.799344206 | Sua5/YciO/YrdC/YwlC family protein |
| CNXL_013390 | CNB04430 | 0.01413582 | 0.986620478 | ribosomal RNA-processing protein 17 |
| CNXL_013400 | CNB04440 | 0.269889753 | 0.597383333 | L-methionine transporter |
| CNXL_013410 | CNB04450 | -0.048243881 | 0.949084374 | replication factor C subunit 3/5 |
| CNXL_013420 | CNB04460 | 0.673211016 | 0.083435528 | 4-nitrophenyl phosphatase |
| CNXL_013430 | CNB04470 | 0.554088598 | 0.155884641 | hypothetical protein |
| CNXL_013440 | CNB04480 | -0.381276274 | 0.326972642 | hypothetical protein |
| CNXL_013450 | CNB04500 | -0.104037814 | 0.88622696 | Gly-Xaa carboxypeptidase |
| CNXL_013460 | CNB04510 | -0.846304272 | 0.015762174 | hypothetical protein |
| CNXL_013470 | CNB04520 | 0.199174468 | 0.767511661 | minichromosome maintenance protein 6 |
| CNXL_013480 | CNB04530 | -0.257962349 | 0.637704309 | tyrosine phosphatase |
| CNXL_013490 | CNB04540 | -0.484331386 | 0.131296263 | zinc knuckle family protein |
| CNXL_013500 | CNB04550 | -0.21916387 | 0.741426667 | ribosome recycling factor |
| CNXL_013510 | CNB04560 | 0.798184879 | 0.014800468 | hypothetical protein |
| CNXL_013520 | CNB04570 | -0.172849178 | 0.750060175 | f-actin-capping protein subunit alpha |
| CNXL_013530 | CNB04580 | 0.009181422 | 0.990777629 | nuclear poly |
| CNXL_013540 | CNB04590 | 0.175196982 | 0.742187954 | F-type H+-transporting ATPase subunit D |
| CNXL_013550 | CNB04600 | -0.329373555 | 0.615520877 | hypothetical protein |
| CNXL_013560 | CNB04610 | 0.007294678 | 0.994114935 | Unknown |
| CNXL_013570 | CNB04620 | -0.407500623 | 0.243026936 | hydantoinase |
| CNXL_013580 | CNB04630 | 0.380490783 | 0.350443288 | hypothetical protein |
| CNXL_013590 | CNB04640 | -0.247917643 | 0.600660852 | Unknown |
| CNXL_013600 | CNB04650 | 0.492627327 | 0.141858814 | ribosome biogenesis protein ERB1 |
| CNXL_013610 | CNB04660 | -0.196544068 | 0.87678601 | hypothetical protein |
| CNXL_013620 | CNB04670 | -0.116698054 | 0.863067672 | hypothetical protein |
| CNXL_013630 | CNB04680 | 0.246189319 | 0.631542919 | hypothetical protein |
| CNXL_013640 | | -3.7001638 | 0.007904957 | ATP |
| CNXL_013650 | CNB04690 | -0.185519444 | 0.738006012 | hypothetical protein |
| CNXL_013660 | CNB04700 | -0.151395901 | 0.776125901 | palmitoyltransferase PFA4 |
| CNXL_013670 | CNB04710 | 0.263226586 | 0.634728039 | hypothetical protein |
| CNXL_013680 | | 0.497787121 | 0.150894977 | oxidoreductase |
| CNXL_013690 | CNB04720 | 0.025805408 | 0.972638293 | hypothetical protein |
| CNXL_013700 | CNB04730 | -0.111636573 | 0.843913486 | glutathione-dependent oxidoreductase |
| CNXL_013710 | CNB04740 | 0.474208316 | 0.176625829 | nam9 protein |
| CNXL_013720 | CNB04750 | -0.00277307 | 0.996472611 | elongation factor 1 alpha-like protein |
| CNXL_013730 | CNB04760 | 0.02473135 | 0.981240834 | solute carrier family 38 |
| CNXL_013740 | | 0.412385131 | 0.643890318 | hypothetical protein |
| CNXL_013750 | CNB04770 | 0.877734611 | 0.005375317 | Unknown |
| CNXL_013760 | CNB04780 | 0.501740791 | 0.152901938 | integral membrane protein |
| CNXL_013770 | CNB04790 | -0.484245749 | 0.232022599 | GTP-binding protein 1 |
| CNXL_013780 | CNB04800 | -0.504122953 | 0.170587304 | microtubule binding protein |
| CNXL_013790 | | 0.332719508 | 0.55161956 | leucine carboxyl methyltransferase 1 |
| CNXL_013800 | CNB04810 | -0.074672779 | 0.90260794 | hypothetical protein |
| CNXL_013810 | | -0.801463101 | 0.787846251 | guanosine-diphosphatase |
| CNXL_013820 | CNB04820 | -0.00734243 | 0.992176872 | hypothetical protein |
| CNXL_013830 | CNB04830 | 0.552170854 | 0.202621105 | mads-box transcription factor |
| CNXL_013840 | CNB04840 | -0.480534067 | 0.432050869 | hypothetical protein |
| CNXL_013850 | CNB04850 | -0.032638169 | 0.965101267 | cytochrome c oxidase assembly protein subunit 11 |
| CNXL_013860 | CNB04860 | -0.099841016 | 0.858924359 | 20S proteasome subunit alpha 1 |
| CNXL_013870 | CNB04870 | -1.076163458 | 7.32658E-06 | hypothetical protein |
| CNXL_013880 | NB04880\|CNB048[illegible] | -0.0129319 | 0.986747477 | pumilio 2 |
| CNXL_013890 | | 0.007866947 | 0.996158838 | Unknown |
| CNXL_013900 | CNB04900 | -3.479011113 | 9.9769E-22 | hypothetical protein |

| | | | | |
|---|---|---|---|---|
| CNXL_013910 | | -0.012181677 | 0.99143423 | septin ring protein |
| CNXL_013920 | CNB04910 | 0.097827647 | 0.856469734 | hypothetical protein |
| CNXL_013930 | CNB04920 | -0.016078508 | 0.986381348 | aminopeptidase 2 |
| CNXL_013940 | CNB04930 | -0.293781204 | 0.50345637 | GTP cyclohydrolase I |
| CNXL_013950 | CNB04940 | 0.409164585 | 0.372306093 | large subunit ribosomal protein L37a |
| CNXL_013960 | CNB04950 | 0.596197368 | 0.068722808 | hypothetical protein |
| CNXL_013970 | CNB04960 | 0.31925865 | 0.474675516 | potassium channel protein |
| CNXL_013980 | | -1.462076112 | 0.059246477 | 26S proteasome regulatory subunit N9 |
| CNXL_013990 | | -0.917354752 | 0.322555045 | Unknown |
| CNXL_014000 | CNB04980 | 0.427904399 | 0.417866535 | Unknown |
| CNXL_014010 | CNB04990 | 0.231332905 | 0.606726524 | hypothetical protein |
| CNXL_014020 | CNB05000 | -1.123534376 | 0.00081585 | methionine-R-sulfoxide reductase |
| CNXL_014030 | | 0.198889718 | 0.701325477 | hypothetical protein |
| CNXL_014040 | CNB05030 | -0.221473086 | 0.592514661 | hypothetical protein |
| CNXL_014050 | CNB05040 | -0.578201407 | 0.230808262 | large subunit ribosomal protein L24 |
| CNXL_014060 | CNB05050 | -0.054305526 | 0.939255348 | translation initiation factor |
| CNXL_014070 | CNB05060 | 0.979268287 | 0.003311461 | hypothetical protein |
| CNXL_014080 | CNB05080 | 0.973529387 | 0.000963619 | myo-inositol transporter |
| CNXL_014090 | CNB05090 | -0.004760128 | 0.995735232 | transaldolase |
| CNXL_014100 | CNB05100 | -4.309582972 | 1.20504E-81 | chromosome transmission fidelity protein 1 |
| CNXL_014110 | CNB05110 | 0.328503705 | 0.35683476 | hypothetical protein |
| CNXL_014120 | CNB05120 | -2.291635031 | 1.28806E-22 | RNA binding protein |
| CNXL_014130 | | 0.215453924 | 0.638378602 | cytochrome P450 |
| CNXL_014140 | CNB05130 | 0.003564481 | 0.996158838 | hypothetical protein |
| CNXL_014150 | CNB05140 | -0.125381627 | 0.807491917 | ribosomal RNA large subunit methyltransferase J |
| CNXL_014160 | CNB05150 | -0.031468286 | 0.965465882 | AFG3 family protein |
| CNXL_014170 | CNB05160 | 0.064896596 | 0.93619311 | alpha 1 |
| CNXL_014180 | CNB05180 | -0.868074993 | 0.006867703 | hypothetical protein |
| CNXL_014190 | CNB05190 | -0.169824557 | 0.760634427 | hypothetical protein |
| CNXL_014200 | CNB05200 | 0.475614192 | 0.232845005 | hypothetical protein |
| CNXL_014210 | | 0.117332883 | 0.911130835 | MFS quinate transporter QutD |
| CNXL_014220 | CNB05230 | 0.209035075 | 0.669296216 | Unknown |
| CNXL_014230 | CNB05240 | -0.46029933 | 0.39417374 | AGC/RSK protein kinase |
| CNXL_014240 | | 0.341004799 | 0.570755994 | hypothetical protein |
| CNXL_014250 | CNB05260 | 1.168104204 | 0.001095672 | Unknown |
| CNXL_014260 | CNB05270 | -0.052816666 | 0.937054176 | hypothetical protein |
| CNXL_014270 | CNB05280 | -0.12815617 | 0.857811508 | U3 small nucleolar RNA-associated protein 6 |
| CNXL_014280 | CNB05290 | -0.208721699 | 0.720877413 | hypothetical protein |
| CNXL_014290 | CNB05310 | -0.18926199 | 0.730404763 | hypothetical protein |
| CNXL_014300 | CNB05320 | -0.157868211 | 0.778681483 | hypothetical protein |
| CNXL_014310 | CNB05330 | -0.510077072 | 0.316888126 | 53 kda brg1-associated factor b |
| CNXL_014320 | CNB05340 | -0.076374492 | 0.90257926 | serine/threonine protein phosphatase 5 phosphatase |
| CNXL_014330 | CNB05350 | -0.17180439 | 0.78809694 | small nuclear ribonucleoprotein D3 |
| CNXL_014340 | CNB05360 | -0.648168071 | 0.049176815 | ATP-dependent RNA helicase A |
| CNXL_014350 | CNB05370 | 0.168444972 | 0.771763303 | minichromosome maintenance protein 5 |
| CNXL_014360 | CNB05380 | 0.36818288 | 0.306455537 | hypothetical protein |
| CNXL_014370 | CNB05390 | -0.525386586 | 0.260424002 | translation initiation factor SUI1 |
| CNXL_014380 | CNB05400 | -0.153838215 | 0.8124229 | cell cycle checkpoint protein |
| CNXL_014390 | CNB05410 | -1.103707782 | 2.48089E-05 | hypothetical protein |
| CNXL_014400 | CNB05430 | -0.234164128 | 0.75185116 | glutaredoxin |
| CNXL_014410 | CNB05440 | 0.214474834 | 0.879531297 | Unknown |
| CNXL_014420 | | -1.484034675 | 0.105307289 | Unknown |
| CNXL_014430 | | -0.298806884 | 0.776955714 | Unknown |
| CNXL_014440 | CNB05450 | -0.354681897 | 0.421861586 | Unknown |
| CNXL_014450 | CNB05460 | 0.604796141 | 0.139632831 | ribosome biogenesis protein ENP2 |
| CNXL_014460 | CNB05480 | -0.834579487 | 0.016891408 | selenoprotein W |
| CNXL_014470 | CNB05490 | 0.188626998 | 0.804701377 | hypothetical protein |
| CNXL_014480 | CNB05500 | -0.085368593 | 0.88147344 | UDP-N-acetylglucosamine transferase subunit ALG13 |

| | | | | |
|---|---|---|---|---|
| CNXL_014490 | | 1.556800549 | 0.112976658 | endoribonuclease L-PSP |
| CNXL_014500 | CNB05510 | -0.334030684 | 0.43911626 | haloacid dehalogenase |
| CNXL_014510 | CNB05520 | 0.004383273 | 0.995735232 | large subunit ribosomal protein L28e |
| CNXL_014520 | CNB05530 | 1.057405427 | 0.002180243 | U4/U6 small nuclear ribonucleoprotein PRP31 |
| CNXL_014530 | CNB05540 | 0.145412486 | 0.792018301 | exonuclease |
| CNXL_014540 | CNB05550 | 0.095518879 | 0.889001755 | 20S proteasome subunit alpha 6 |
| CNXL_014550 | CNB05560 | -0.414722835 | 0.381569079 | 60S ribosome subunit biogenesis protein nip7 |
| CNXL_014560 | CNB05570 | -0.296092195 | 0.455207658 | hypothetical protein |
| CNXL_014570 | CNB05580 | -0.115138047 | 0.85924574 | coatomer beta' subunit |
| CNXL_014580 | CNB05600 | -0.176329238 | 0.753998183 | U3 small nucleolar RNA-associated protein MPP10 |
| CNXL_014590 | CNB05610 | -0.172769698 | 0.771763303 | oligosaccharyltransferase complex subunit gamma |
| CNXL_014600 | CNB05620 | 0.156932253 | 0.779355929 | HAT1-interacting factor 1 |
| CNXL_014610 | CNB05630 | 0.203197574 | 0.721006731 | 3-hydroxyanthranilate 3 |
| CNXL_014620 | CNB05640 | -0.185322243 | 0.804454639 | hypothetical protein |
| CNXL_014630 | CNB05650 | -0.517643563 | 0.191011984 | proline-tRNA ligase |
| CNXL_014640 | CNB05660 | 1.054433172 | 0.000448401 | dolichol kinase |
| CNXL_014650 | CNB05670 | -1.464293272 | 8.15326E-09 | oxidoreductase |
| CNXL_014660 | | -0.173041316 | 0.847923235 | hypothetical protein |
| CNXL_014670 | | -0.224583615 | 0.714520393 | hypothetical protein |
| CNXL_014680 | CNB05680 | 0.068297241 | 0.918171803 | hypothetical protein |
| CNXL_014690 | CNB05690 | 0.314419675 | 0.460815133 | coatomer zeta subunit |
| CNXL_014700 | CNB05700 | -0.406639894 | 0.450491582 | activating transcription factor |
| CNXL_014710 | NB05710\|CNB057: | -0.052206229 | 0.935317161 | hypothetical protein |
| CNXL_014720 | CNB05730 | -0.753809529 | 0.090454478 | Unknown |
| CNXL_014730 | CNB05740 | -0.36359889 | 0.461869386 | hypothetical protein |
| CNXL_014740 | CNB05750 | -0.457380396 | 0.226593506 | alpha-L-rhamnosidase |
| CNXL_014750 | CNB05760 | -0.157669308 | 0.793711563 | racemase |
| CNXL_014760 | CNB05770 | 0.657098122 | 0.046232519 | glucose 1-dehydrogenase |
| CNXL_014770 | | 0.709415701 | 0.102152715 | ATP-binding cassette transporter |
| CNXL_014780 | CNC01250 | 0.389258593 | 0.403907252 | Unknown |
| CNXL_014790 | | 0.689610824 | 0.415892664 | Unknown |
| CNXL_014800 | | 0.180187931 | NA | Unknown |
| CNXL_014810 | CNC00020 | -0.143428668 | 0.814220979 | hypothetical protein |
| CNXL_014820 | | 0.363140015 | 0.690050337 | hypothetical protein |
| CNXL_014830 | CNC00030 | -0.011809574 | 0.990008547 | Unknown |
| CNXL_014840 | CNC00040 | 0.173322486 | 0.725978857 | hypothetical protein |
| CNXL_014850 | | 0.216619906 | 0.654774176 | hypothetical protein |
| CNXL_014860 | | -0.069839004 | 0.962706186 | hypothetical protein |
| CNXL_014870 | CNC00050 | -1.341831917 | 1.72762E-06 | hypothetical protein |
| CNXL_014880 | CNC00060 | -0.163754112 | 0.799344206 | putative chitin synthase |
| CNXL_014890 | CNC00080 | 1.051969267 | 0.000232355 | sugar transporter |
| CNXL_014900 | CNC00090 | 0.943863639 | 0.002985831 | DNA ligase D |
| CNXL_014910 | CNC00100 | -0.258075598 | 0.690272467 | cupin domain-containing protein |
| CNXL_014920 | CNC00110 | -0.355063655 | 0.342703959 | ATP-dependent RNA helicase MRH4 |
| CNXL_014930 | CNC00130 | -0.102798375 | 0.886480005 | fatty acid elongase |
| CNXL_014940 | | -0.88404185 | 0.086776919 | type I phosphodiesterase/nucleotide pyrophosphatase |
| CNXL_014950 | | -0.503700532 | 0.604159997 | hypothetical protein |
| CNXL_014960 | CNC00140 | -0.302244655 | 0.648836492 | hypothetical protein |
| CNXL_014970 | | -0.450803774 | 0.233279208 | DNA polymerase delta subunit 3 |
| CNXL_014980 | CNC00160 | 0.255679261 | 0.519652797 | Unknown |
| CNXL_014990 | | -0.120501752 | 0.890053972 | enolase |
| CNXL_015000 | | -0.005622357 | 0.996158838 | Unknown |
| CNXL_015010 | CNC00180 | 0.159560564 | 0.82996137 | Unknown |
| CNXL_015020 | CNC00190 | 0.465196354 | 0.146597183 | compass component swd2 |
| CNXL_015030 | CNC00200 | 0.196610733 | 0.779355929 | hydrolase |
| CNXL_015040 | CNC00210 | 0.458783134 | 0.202621105 | hypothetical protein |
| CNXL_015050 | CNC00220 | -0.887617036 | 0.009405958 | hydroxymethylglutaryl-CoA lyase |
| CNXL_015060 | CNC00230 | 0.120714926 | 0.809974479 | CTP synthase |

| | | | | |
|---|---|---|---|---|
| CNXL_015070 | CNC00240 | -0.067436057 | 0.929657464 | ER-derived vesicles protein ERV14 |
| CNXL_015080 | CNC00250 | -0.437239062 | 0.299696006 | nucleolar protein 16 |
| CNXL_015090 | CNC00260 | -0.084414148 | 0.918171803 | hypothetical protein |
| CNXL_015100 | CNC00270 | -0.850816364 | 0.006867703 | hypothetical protein |
| CNXL_015110 | | -0.805392996 | 0.276076329 | multiple drug resistance protein |
| CNXL_015120 | CNC00290 | 1.176810147 | 0.000160622 | Unknown |
| CNXL_015130 | CNC00300 | 0.431157733 | 0.189523416 | multiple drug resistance protein |
| CNXL_015140 | CNC00310 | 1.303587675 | 0.000309854 | hypothetical protein |
| CNXL_015150 | CNC00320 | 0.379558657 | 0.423141864 | hypothetical protein |
| CNXL_015160 | | -1.461019702 | 2.44911E-07 | hypothetical protein |
| CNXL_015170 | CNC00330 | -0.222135165 | 0.660412993 | hypothetical protein |
| CNXL_015180 | CNC00340 | 0.852227392 | 0.002481424 | bud site selection protein 20 |
| CNXL_015190 | CNC00350 | -0.139935216 | 0.779355929 | hypothetical protein |
| CNXL_015200 | CNC00360 | -0.119885089 | 0.842469317 | large subunit ribosomal protein L23 |
| CNXL_015210 | CNC00370 | 0.528346137 | 0.21968773 | PP2Cc protein phosphatase |
| CNXL_015220 | | 0.12291821 | 0.910818522 | polyamine transporter |
| CNXL_015230 | CNC00380 | -0.011466335 | 0.990777629 | Unknown |
| CNXL_015240 | CNC00390 | -0.15031999 | 0.771763303 | hypothetical protein |
| CNXL_015250 | CNC00400 | -0.459625674 | 0.258951716 | hypothetical protein |
| CNXL_015260 | CNC00410 | -1.226188798 | 0.00027121 | gluconokinase |
| CNXL_015270 | CNC00420 | -0.469076811 | 0.271362906 | hypothetical protein |
| CNXL_015280 | CNC00430 | 0.654051669 | 0.051721235 | hypothetical protein |
| CNXL_015290 | CNC00440 | -0.516038468 | 0.50585751 | hypothetical protein |
| CNXL_015300 | CNC00450 | -0.079891632 | 0.927387051 | hypothetical protein |
| CNXL_015310 | CNC00460 | 0.231529601 | 0.637704309 | Unknown |
| CNXL_015320 | CNC00470 | 0.349111863 | 0.337535369 | DNA damage-inducible protein 1 |
| CNXL_015330 | CNC00480 | -0.204464953 | 0.883693135 | hypothetical protein |
| CNXL_015340 | CNC00490 | 0.56962869 | 0.113446245 | hypothetical protein |
| CNXL_015350 | CNC00500 | -0.156269343 | 0.809305305 | potassium ion transporter |
| CNXL_015360 | | 0.012238608 | 0.995735232 | RNA exonuclease 1 |
| CNXL_015370 | CNC00510 | 0.071284002 | 0.939255348 | Unknown |
| CNXL_015380 | CNC00520 | -0.125410023 | 0.838178569 | hypothetical protein |
| CNXL_015390 | | -0.038543286 | 0.972638293 | dynactin 2 |
| CNXL_015400 | | -1.725654829 | 0.063702758 | Unknown |
| CNXL_015410 | CNC00550 | 0.221743076 | 0.689993365 | hypothetical protein |
| CNXL_015420 | CNC00560 | -0.540154746 | 0.369739382 | protein FRA10AC1 |
| CNXL_015430 | | 0.233316029 | 0.677906547 | hypothetical protein |
| CNXL_015440 | CNC00570 | -0.024718317 | 0.975204851 | hypothetical protein |
| CNXL_015450 | | 0.239278938 | 0.652704639 | hypothetical protein |
| CNXL_015460 | CNC00590 | 0.184464376 | 0.733041323 | Unknown |
| CNXL_015470 | CNC00600 | 0.16875989 | 0.761797019 | V-type H+-transporting ATPase subunit D |
| CNXL_015480 | CNC00620 | -0.262463072 | 0.665556981 | AGC protein kinase |
| CNXL_015490 | CNC00630 | 0.43640367 | 0.321761273 | hypothetical protein |
| CNXL_015500 | CNC00640 | -3.902821333 | 1.36937E-37 | hypothetical protein |
| CNXL_015510 | CNC00650 | -0.251578758 | 0.561580907 | hypothetical protein |
| CNXL_015520 | CNC00660 | 0.44623643 | 0.151765096 | hypothetical protein |
| CNXL_015530 | CNC00670 | 0.296008367 | 0.534813127 | long-chain acyl-CoA synthetase |
| CNXL_015540 | CNC00680 | 0.319675662 | 0.462811802 | nuclear protein |
| CNXL_015550 | CNC00690 | 0.61745851 | 0.040167747 | transcription initiation factor TFIIF subunit alpha |
| CNXL_015560 | | -0.032853533 | 0.965101267 | chaperone regulator |
| CNXL_015570 | | 0.4156074 | 0.570755994 | Unknown |
| CNXL_015580 | | 0.934386632 | 0.007878419 | hypothetical protein |
| CNXL_015590 | CNC00720 | 0.98026751 | 0.001795837 | dolichyldiphosphatase |
| CNXL_015600 | CNC00730 | 0.051066786 | 0.936783812 | OPT family small oligopeptide transporter |
| CNXL_015610 | | 0.469583311 | NA | quorum sensing-like molecule |
| CNXL_015620 | CNC00760 | -3.55820075 | 8.55106E-51 | Unknown |
| CNXL_015630 | CNC00780 | -0.498545982 | 0.160833876 | pr4/barwin domain protein |
| CNXL_015640 | CNC00790 | -0.308999524 | 0.499530076 | histone deacetylase 6/10 |

| | | | | |
|---|---|---|---|---|
| CNXL_015650 | CNC00800 | -0.349962627 | 0.49332489 | nucleolar protein 12 |
| CNXL_015660 | CNC00810 | -0.188349822 | 0.787846251 | biotin-[acetyl-CoA-carboxylase] ligase |
| CNXL_015670 | CNC00820 | -0.346881231 | 0.38882813 | gamma-tubulin complex component 3 |
| CNXL_015680 | CNC00830 | 0.093379298 | 0.866675918 | hypothetical protein |
| CNXL_015690 | CNC00840 | 0.260766877 | 0.581889939 | T-complex protein 1 subunit alpha |
| CNXL_015700 | | 0.495853589 | 0.458970882 | hypothetical protein |
| CNXL_015710 | | 0.807104417 | 0.032155316 | hypothetical protein |
| CNXL_015720 | CNC00850 | 1.077389155 | 0.00083476 | hypothetical protein |
| CNXL_015730 | CNC00860 | 0.348556557 | 0.450895285 | hypothetical protein |
| CNXL_015740 | CNC00870 | -0.836911368 | 0.034218166 | Unknown |
| CNXL_015750 | CNC00880 | -0.438659347 | 0.341422478 | tyrosine phosphatase |
| CNXL_015760 | CNC00890 | -0.76699743 | 0.085687476 | hypothetical protein |
| CNXL_015770 | CNC00900 | 0.291845898 | 0.519652797 | hypothetical protein |
| CNXL_015780 | CNC00910 | 0.46107102 | 0.119559959 | ATP-binding cassette transporter |
| CNXL_015790 | CNC00920 | 0.10866936 | 0.828543224 | hypothetical protein |
| CNXL_015800 | | -0.03816437 | 0.967295199 | glutamate dehydrogenase |
| CNXL_015810 | | 0.168825887 | 0.797685526 | Unknown |
| CNXL_015820 | CNC00940 | -0.320582409 | 0.531504526 | hypothetical protein |
| CNXL_015830 | CNC00950 | -0.49015161 | 0.26081757 | vacuolar membrane-associated protein IML1 |
| CNXL_015840 | CNC00960 | 0.262598796 | 0.539612629 | hypothetical protein |
| CNXL_015850 | CNC00970 | 1.398811284 | 8.6303E-06 | vacuolar-sorting protein SNF7 |
| CNXL_015860 | CNC00980 | 1.614771944 | 6.02535E-06 | hydrolase |
| CNXL_015870 | CNC00990 | -0.044536996 | 0.944879244 | hypothetical protein |
| CNXL_015880 | CNC01000 | 0.399236692 | 0.315522035 | F-type H+-transporting ATPase subunit B |
| CNXL_015890 | CNC01030 | -0.323061852 | 0.509453385 | RNA polymerase-associated protein LEO1 |
| CNXL_015900 | CNC01040 | -0.422390839 | 0.353330493 | large subunit ribosomal protein L32 |
| CNXL_015910 | CNC01050 | -1.947827956 | 1.6998E-16 | hypothetical protein |
| CNXL_015920 | CNC01060 | 0.021364957 | 0.982102702 | protein-S-isoprenylcysteine O-methyltransferase |
| CNXL_015930 | CNC01070 | -0.423455218 | 0.249559817 | ribosome biogenesis protein SSF1/2 |
| CNXL_015940 | CNC01080 | 0.089851197 | 0.902229878 | glycine dehydrogenase |
| CNXL_015950 | CNC01090 | -0.001501236 | 0.997459233 | hypothetical protein |
| CNXL_015960 | | -0.16152407 | 0.837479784 | hypothetical protein |
| CNXL_015970 | CNC01110 | 0.07249936 | 0.903834486 | hypothetical protein |
| CNXL_015980 | | -0.416742835 | 0.623050693 | template-activating factor I |
| CNXL_015990 | CNC01130 | -0.519999105 | 0.179780745 | hypothetical protein |
| CNXL_016000 | CNC01140 | -0.173588645 | 0.814220979 | ribosome assembly protein RRB1 |
| CNXL_016010 | CNC01150 | -0.536383006 | 0.113351667 | lipase |
| CNXL_016020 | CNC01160 | -0.245726338 | 0.595655121 | IQ domain-containing calmodulin-binding protein |
| CNXL_016030 | CNC01170 | 0.825621338 | 0.010542889 | hypothetical protein |
| CNXL_016040 | CNC01180 | 1.218066743 | 0.000682682 | TIGR01458 family HAD hydrolase |
| CNXL_016050 | CNC01190 | 0.393849642 | 0.862678265 | rossman fold oxidoreductase |
| CNXL_016060 | CNC01200 | 0.408350798 | 0.282976705 | hypothetical protein |
| CNXL_016070 | CNC01210 | -0.118346773 | 0.846829842 | thioredoxin |
| CNXL_016080 | CNC01220 | 0.152567756 | 0.78519633 | nuclear GTP-binding protein |
| CNXL_016090 | CNC01230 | 0.480889196 | 0.152336867 | cystathionine gamma-synthase |
| CNXL_016100 | CNC01240 | -0.361057099 | 0.462919345 | hypothetical protein |
| CNXL_016110 | | -0.220514348 | 0.678123328 | infection related protein of unknown function |
| CNXL_016120 | CNC01260 | -0.860126938 | 0.001630427 | Unknown |
| CNXL_016130 | CNC01270 | -0.201986523 | 0.703985737 | CAMK/CAMKL protein kinase |
| CNXL_016140 | CNC01280 | 0.02173821 | 0.981733632 | hypothetical protein |
| CNXL_016150 | CNC01290 | 1.088611857 | 0.001928327 | putative ubiquitin carboxyl-terminal hydrolase |
| CNXL_016160 | CNC01300 | 0.014870412 | 0.986880752 | aldo-keto reductase |
| CNXL_016170 | CNC01310 | 0.002889825 | 0.996374193 | hypothetical protein |
| CNXL_016180 | CNC01320 | -0.354483042 | 0.393761434 | bZip transcription factor |
| CNXL_016190 | CNC01330 | -0.271808581 | 0.621585258 | hypothetical protein |
| CNXL_016200 | CNC01340 | 0.334016758 | 0.476292329 | hypothetical protein |
| CNXL_016210 | CNC01350 | 0.512834669 | 0.137302949 | hypothetical protein |
| CNXL_016220 | CNC01360 | 0.351928706 | 0.452964114 | hypothetical protein |

| | | | | |
|---|---|---|---|---|
| CNXL_016230 | CNC01370 | -0.014602566 | 0.987076518 | CTD kinase subunit beta |
| CNXL_016240 | | 0.102132169 | 0.879816975 | tRNA pseudouridine |
| CNXL_016250 | CNC01380 | 0.276136706 | 0.615722567 | hypothetical protein |
| CNXL_016260 | CNC01390 | 0.220216674 | 0.691220257 | hypothetical protein |
| CNXL_016270 | CNC01400 | -0.117123775 | 0.885449489 | predicted saga histone acetyltransferase complex component |
| CNXL_016280 | CNC01410 | -0.277431967 | 0.535382959 | amino oxidase |
| CNXL_016290 | CNC01420 | -0.276303402 | 0.564032914 | small subunit ribosomal protein S20 |
| CNXL_016300 | CNC01430 | -0.126266302 | 0.90384925 | multifunctional methyltransferase subunit TRM112 |
| CNXL_016310 | CNC01440 | -0.150486049 | 0.86171371 | polyadenylation factor subunit 2 |
| CNXL_016320 | CNC01450 | 0.629096236 | 0.08672496 | mRNA cap guanine-N7 methyltransferase |
| CNXL_016330 | | 0.225900353 | 0.661992658 | triacylglycerol lipase |
| CNXL_016340 | CNC01470 | -0.189390811 | 0.734629931 | anaphase-promoting complex subunit 5 |
| CNXL_016350 | NC01480\|CNI0366 | 0.459886692 | 0.146597183 | large subunit GTPase 1 |
| CNXL_016360 | CNC01490 | 0.194868466 | 0.73717204 | hypothetical protein |
| CNXL_016370 | CNC01500 | 0.045777452 | 0.948286148 | peptidyl-prolyl cis-trans isomerase-like 1 |
| CNXL_016380 | CNC01510 | -0.174606381 | 0.771763303 | COPII-coated vesicle component Erv46 |
| CNXL_016390 | CNC01520 | -0.412604569 | 0.332119185 | mitotic spindle assembly checkpoint protein MAD2 |
| CNXL_016400 | | 0.231057929 | 0.671115959 | elongation factor G |
| CNXL_016410 | CNC01530 | -0.043255572 | 0.957169694 | Unknown |
| CNXL_016420 | | 0.296657472 | 0.683642142 | hypothetical protein |
| CNXL_016430 | CNC01540 | -0.203964686 | 0.637722771 | Unknown |
| CNXL_016440 | CNC01550 | 0.243007835 | 0.551883314 | DNA polymerase phi subunit |
| CNXL_016450 | | 0.063205721 | 0.927504878 | DNA mismatch repair protein MSH3 |
| CNXL_016460 | CNC01570 | 0.049783488 | 0.936783812 | hypothetical protein |
| CNXL_016470 | CNC01580 | 0.409352497 | 0.533593687 | protein of unknown function |
| CNXL_016480 | CNC01590 | 0.430992745 | 0.259411381 | hypothetical protein |
| CNXL_016490 | CNC01600 | 0.042320881 | 0.953095191 | rab escort protein |
| CNXL_016500 | NC01610\|CNL056 | 0.18125978 | 0.666536582 | translocation protein SEC66 |
| CNXL_016510 | CNC01620 | -0.238819316 | 0.616763577 | Unknown |
| CNXL_016520 | CNC01630 | 0.03361892 | 0.970515325 | hypothetical protein |
| CNXL_016530 | CNC01640 | -0.113169977 | 0.879770554 | large subunit ribosomal protein L7 |
| CNXL_016540 | NC01650\|CNC016 | 0.708478289 | 0.043740344 | glicosidase |
| CNXL_016550 | CNC01670 | -1.001885115 | 0.000562565 | Unknown |
| CNXL_016560 | CNC01680 | -0.225056261 | 0.687195736 | putative capsule structure designer protein |
| CNXL_016570 | CNC01690 | -0.785705775 | 0.265153342 | dynamin GTPase |
| CNXL_016580 | CNC01700 | 0.035252451 | 0.956213883 | hypothetical protein |
| CNXL_016590 | CNC01710 | -0.369915333 | 0.526048587 | fumarate hydratase |
| CNXL_016600 | | -0.204894492 | 0.710705655 | hypothetical protein |
| CNXL_016610 | CNC01720 | 0.028128717 | 0.981240834 | Unknown |
| CNXL_016620 | CNC01730 | -0.107522302 | 0.889158863 | hypothetical protein |
| CNXL_016630 | CNC01740 | -0.533212749 | 0.312497815 | translation initiation factor eif-2b subunit alpha |
| CNXL_016640 | | -0.441205805 | 0.324707325 | hypothetical protein |
| CNXL_016650 | CNC01750 | -0.690192298 | 0.039954962 | dynactin 4 |
| CNXL_016660 | CNC01760 | -0.213010412 | 0.77138893 | CMGC/CDK/CDC2 protein kinase |
| CNXL_016670 | CNC01770 | 0.000486653 | 0.99867251 | tRNA-splicing endonuclease subunit Sen34 |
| CNXL_016680 | CNC01780 | -0.173797292 | 0.825383092 | U6 snRNA-associated Sm-like protein LSm2 |
| CNXL_016690 | CNC01790 | -2.795924162 | 1.19019E-07 | 4-hydroxybenzoate polyprenyl transferase |
| CNXL_016700 | CNC01800 | -0.205713614 | 0.78519633 | hypothetical protein |
| CNXL_016710 | CNC01810 | 0.092255707 | 0.928118121 | metallo-beta-lactamase |
| CNXL_016720 | CNC01820 | -0.242476183 | 0.624571241 | hypothetical protein |
| CNXL_016730 | CNC01830 | -1.215155933 | 0.000415078 | acetyl/propionyl CoA carboxylase |
| CNXL_016740 | CNC01840 | -0.198968535 | 0.680057336 | Ras family protein |
| CNXL_016750 | CNC01850 | -0.475711357 | 0.251973685 | hypothetical protein |
| CNXL_016760 | CNC01860 | 0.012815031 | 0.987759522 | MFS transporter |
| CNXL_016770 | CNC01870 | 0.123574474 | 0.814220979 | tagatose-bisphosphate aldolase |
| CNXL_016780 | CNC01880 | -0.034348386 | 0.965966884 | tRNA |
| CNXL_016790 | CNC01890 | -0.194139774 | 0.696516885 | ER to Golgi transporter Yif1 |
| CNXL_016800 | CNC01900 | 0.014537034 | 0.986620478 | Na+/H+ antiporter |

| | | | | |
|---|---|---|---|---|
| CNXL_016810 | | -0.06123281 | 0.919196787 | small subunit ribosomal protein S15 |
| CNXL_016820 | | 0.001857954 | 0.996833113 | Unknown |
| CNXL_016830 | CNC01920 | -0.648103485 | 0.041154937 | Unknown |
| CNXL_016840 | CNC01930 | 0.558231002 | 0.051735477 | cytosine permease |
| CNXL_016850 | CNC01940 | 0.005943771 | 0.995428247 | mitochondrial outer membrane 72K protein |
| CNXL_016860 | | -0.61383293 | 0.099077349 | putative monosaccharide transporter |
| CNXL_016870 | CNC01950 | 0.788114853 | 0.018223382 | Unknown |
| CNXL_016880 | CNC01960 | 0.158261085 | 0.740130561 | hypothetical protein |
| CNXL_016890 | CNC01970 | -0.110615182 | 0.840027363 | Atypical/RIO/RIO2 protein kinase |
| CNXL_016900 | | 0.650047469 | 0.055459171 | ATP-dependent metalloprotease |
| CNXL_016910 | CNC01990 | 1.603298375 | 1.33149E-06 | hypothetical protein |
| CNXL_016920 | | 0.484075833 | 0.170528344 | MFS transporter |
| CNXL_016930 | CNC02000 | 0.385133044 | 0.371037534 | hypothetical protein |
| CNXL_016940 | CNC02020 | -0.240835719 | 0.592736437 | rub1/nedd8 activating enzyme |
| CNXL_016950 | CNC02030 | -0.0590451 | 0.942888075 | hypothetical protein |
| CNXL_016960 | CNC02040 | -0.347875472 | 0.363121872 | hypothetical protein |
| CNXL_016970 | CNC02050 | -0.164291234 | 0.749315941 | chaperone DnaJ |
| CNXL_016980 | CNC02060 | 0.005834244 | 0.995735232 | hypothetical protein |
| CNXL_016990 | CNC02070 | -0.573531136 | 0.055015121 | histone deacetylase 1/2 |
| CNXL_017000 | | -0.116927565 | 0.842469317 | importin-alpha export receptor |
| CNXL_017010 | CNC02100 | -0.475525814 | 0.334301792 | integral membrane protein |
| CNXL_017020 | CNC02110 | 0.186673596 | 0.733117842 | integral membrane protein |
| CNXL_017030 | CNC02120 | -0.670587376 | 0.018623644 | hypothetical protein |
| CNXL_017040 | | 0.223376393 | 0.742187954 | serine/threonine protein kinase |
| CNXL_017050 | CNC02130 | -0.431607534 | 0.308584752 | hypothetical protein |
| CNXL_017060 | CNC02140 | -0.262430162 | 0.689917421 | regulator of G protein signaling |
| CNXL_017070 | CNC02150 | -0.571938822 | 0.044032389 | hypothetical protein |
| CNXL_017080 | | 0.860510047 | 0.425599277 | translation initiation factor 5 |
| CNXL_017090 | CNC02160 | 0.506679434 | 0.107353814 | hypothetical protein |
| CNXL_017100 | CNC02170 | -0.096619755 | 0.894317988 | glycerol-3-phosphate O-acyltransferase |
| CNXL_017110 | | 0.481695067 | 0.112263079 | hypothetical protein |
| CNXL_017120 | CNC02180 | -0.240562089 | 0.824755136 | hypothetical protein |
| CNXL_017130 | CNC02190 | -0.462128098 | 0.258626534 | sulfonate dioxygenase |
| CNXL_017140 | CNC02200 | -0.372709457 | 0.400883927 | ribosome biogenesis protein BMS1 |
| CNXL_017150 | CNC02210 | -0.481894139 | 0.272623867 | hypothetical protein |
| CNXL_017160 | CNC02220 | 0.267086736 | 0.529724345 | cell differentiation protein rcd1 |
| CNXL_017170 | CNC02230 | -0.290250973 | 0.656993267 | hypothetical protein |
| CNXL_017180 | CNC02240 | -0.298362582 | 0.641029652 | hypothetical protein |
| CNXL_017190 | CNC02250 | -0.158397939 | 0.749189982 | predicted escrt-I complex protein |
| CNXL_017200 | CNC02260 | 0.461702685 | 0.202085011 | porphobilinogen deaminase |
| CNXL_017210 | CNC02270 | 0.13093298 | 0.830440343 | vacuolar protein sorting-associated protein vps13 |
| CNXL_017220 | | 0.501749243 | 0.497362585 | hypothetical protein |
| CNXL_017230 | | -1.100183545 | 4.6492E-05 | Unknown |
| CNXL_017240 | | -2.968555131 | 5.93251E-45 | Unknown |
| CNXL_017250 | CNC02310 | -0.599945977 | 0.041808753 | hypothetical protein |
| CNXL_017260 | CNC02320 | -0.580485298 | 0.078446193 | FACT complex subunit SPT16 |
| CNXL_017270 | CNC02330 | 0.377333126 | 0.3912428 | hsp71-like protein |
| CNXL_017280 | CNC02340 | -0.517924813 | 0.230812707 | hypothetical protein |
| CNXL_017290 | CNC02350 | -0.897384779 | 0.038945749 | ATP-dependent RNA helicase DHX37/DHR1 |
| CNXL_017300 | | 0.806853432 | 0.483451258 | map kinase kinase |
| CNXL_017310 | CNC02360 | 0.415519553 | 0.261955879 | Unknown |
| CNXL_017320 | CNC02370 | 0.39451962 | 0.392781441 | hypothetical protein |
| CNXL_017330 | CNC02380 | -0.214689915 | 0.671115959 | nuclear protein localization protein 4 |
| CNXL_017340 | CNC02390 | -0.002050499 | 0.996739329 | pre-mRNA-processing factor 19 |
| CNXL_017350 | CNC02400 | 0.777744424 | 0.01117304 | pre-mRNA-splicing factor ATP-dependent RNA helicase |
| CNXL_017360 | | -0.026675567 | 0.976612116 | hypothetical protein |
| CNXL_017370 | CNC02410 | -1.167093835 | 0.000499424 | DASH complex subunit DAD4 |
| CNXL_017380 | CNC02420 | -0.588112226 | 0.208728595 | C-4 methyl sterol oxidase |

| | | | | |
|---|---|---|---|---|
| CNXL_017390 | CNC02430 | -0.557497163 | 0.152901938 | hypothetical protein |
| CNXL_017400 | CNC02440 | -1.141885584 | 1.41696E-05 | hypothetical protein |
| CNXL_017410 | | 0.474310435 | 0.294783143 | septin |
| CNXL_017420 | CNC02450 | 0.21677439 | 0.650714942 | hypothetical protein |
| CNXL_017430 | CNC02460 | 1.292907631 | 0.000127098 | aquaporin water channel |
| CNXL_017440 | CNC02470 | 0.621557432 | 0.088235406 | hypothetical protein |
| CNXL_017450 | CNC02480 | 0.682170984 | 0.017977728 | phosphatase |
| CNXL_017460 | CNC02490 | -0.235453171 | 0.647485945 | glycerol-3-phosphate dehydrogenase |
| CNXL_017470 | CNC02500 | -0.031073924 | 0.970515325 | E3 ubiquitin-protein ligase RNF14 |
| CNXL_017480 | | -0.249287135 | 0.634728039 | DNA mismatch repair protein PMS2 |
| CNXL_017490 | CNC02510 | -0.171830741 | 0.714943724 | Unknown |
| CNXL_017500 | | -0.292265804 | 0.487397795 | beta-glucosidase |
| CNXL_017510 | CNC02520 | 0.619471204 | 0.047437309 | hypothetical protein |
| CNXL_017520 | CNC02530 | 0.990323365 | 0.001503564 | hsp72-like protein |
| CNXL_017530 | CNC02540 | 0.016418615 | 0.983980369 | hypothetical protein |
| CNXL_017540 | CNC02550 | 1.444405883 | 9.99373E-05 | solute carrier family 25 |
| CNXL_017550 | CNC02560 | 0.093804631 | 0.879531297 | hypothetical protein |
| CNXL_017560 | CNC02570 | -0.503309852 | 0.208087633 | hypothetical protein |
| CNXL_017570 | CNC02580 | -0.278435295 | 0.592750411 | hypothetical protein |
| CNXL_017580 | CNC02590 | -0.43070179 | 0.267600275 | hypothetical protein |
| CNXL_017590 | CNC02600 | 0.622375527 | 0.168782041 | polygalacturonase |
| CNXL_017600 | | 0.228942781 | 0.638378602 | vacuolar membrane protein |
| CNXL_017610 | CNC02610 | 0.074584088 | 0.918171803 | hypothetical protein |
| CNXL_017620 | CNC02620 | 0.280300231 | 0.45163285 | hypothetical protein |
| CNXL_017630 | CNC02630 | 0.304158586 | 0.417944835 | hypothetical protein |
| CNXL_017640 | CNC02640 | -0.186917148 | 0.725038374 | hypothetical protein |
| CNXL_017650 | CNC02650 | -0.078074489 | 0.906691628 | cytoplasmic protein |
| CNXL_017660 | | -0.071644087 | 0.918756814 | cytoplasmic protein |
| CNXL_017670 | | -0.147974041 | 0.838021224 | hypothetical protein |
| CNXL_017680 | CNC02670 | -1.017986691 | 0.00083476 | Unknown |
| CNXL_017690 | CNC02680 | -0.292702414 | 0.519073971 | ferric reductase |
| CNXL_017700 | CNC02690 | -0.395287061 | 0.403618844 | hypothetical protein |
| CNXL_017710 | CNC02700 | -0.299745957 | 0.567944024 | solute carrier family 25 |
| CNXL_017720 | CNC02710 | -0.381329888 | 0.358178392 | MRS7 family protein |
| CNXL_017730 | CNC02720 | -0.431187368 | 0.394732969 | hypothetical protein |
| CNXL_017740 | CNC02730 | -0.078661759 | 0.901676457 | hypothetical protein |
| CNXL_017750 | CNC02740 | -0.250579737 | 0.664771357 | beclin 1 |
| CNXL_017760 | CNC02750 | 0.090830175 | 0.885370637 | hypothetical protein |
| CNXL_017770 | CNC02760 | 0.389670204 | 0.30897417 | 2 |
| CNXL_017780 | CNC02770 | 0.66325678 | 0.048176746 | glyoxylate reductase |
| CNXL_017790 | CNC02780 | 0.0381029 | 0.957169694 | hypothetical protein |
| CNXL_017800 | CNC02790 | -0.185614738 | 0.679255446 | hypothetical protein |
| CNXL_017810 | | NA | NA | cytoplasmic protein |
| CNXL_017820 | | NA | NA | Unknown |
| CNXL_017830 | | 2.637144045 | NA | Unknown |
| CNXL_017840 | | -0.436946821 | 0.899721549 | Unknown |
| CNXL_017850 | | 0.34893653 | 0.508466155 | Unknown |
| CNXL_017860 | CNH01470 | 0 | NA | Unknown |
| CNXL_017870 | | NA | NA | Unknown |
| CNXL_017880 | | 0.028072506 | NA | Unknown |
| CNXL_017890 | | 0.542439828 | 0.827557138 | Unknown |
| CNXL_017900 | CNC02800 | 0.02137634 | 0.981240834 | Unknown |
| CNXL_017910 | | -0.080464174 | 0.901676457 | tRNA |
| CNXL_017920 | CNC02810 | 0.15061998 | 0.789326936 | hypothetical protein |
| CNXL_017930 | CNC02820 | -0.301292048 | 0.461869386 | upstream activation factor subunit UAF30 |
| CNXL_017940 | CNC02830 | -0.3224665 | 0.443639403 | ribosome production factor 1 |
| CNXL_017950 | | -0.415463475 | 0.485459548 | ATP-dependent DNA helicase PIF1 |
| CNXL_017960 | CNC02840 | -0.434307216 | 0.246329242 | Unknown |

| | | | | |
|---|---|---|---|---|
| CNXL_017970 | CNC02850 | -0.029661903 | 0.965966884 | hypothetical protein |
| CNXL_017980 | CNC02860 | -0.276690368 | 0.613465435 | 2-hydroxyacid dehydrogenase |
| CNXL_017990 | CNC02870 | -0.632078913 | 0.021447877 | hypothetical protein |
| CNXL_018000 | CNC02880 | -0.436600206 | 0.254129014 | hypothetical protein |
| CNXL_018010 | CNC02890 | -0.065832356 | 0.92249377 | hypothetical protein |
| CNXL_018020 | | 0.020830454 | 0.978188468 | phosphatase activator |
| CNXL_018030 | | 0.306625695 | 0.746347427 | ubiquinol-cytochrome c reductase subunit 8 |
| CNXL_018040 | CNC02920 | -0.096520278 | 0.879531297 | hypothetical protein |
| CNXL_018050 | | 0.286644941 | 0.529724345 | Fe-S cluster assembly protein DRE2 |
| CNXL_018060 | CNC02950 | -2.836880252 | 1.09077E-15 | Unknown |
| CNXL_018070 | CNC02960 | -0.42579805 | 0.297121219 | hypothetical protein |
| CNXL_018080 | CNC02970 | 0.029608034 | 0.966210833 | ATP-dependent helicase |
| CNXL_018090 | CNC02980 | -0.317467749 | 0.576230883 | solute carrier family 25 |
| CNXL_018100 | CNC02990 | -0.51004461 | 0.368990556 | U4/U6 small nuclear ribonucleoprotein PRP3 |
| CNXL_018110 | CNC03000 | 0.214781541 | 0.663234353 | DNA-directed RNA polymerase I subunit RPA12 |
| CNXL_018120 | CNC03010 | -0.15855626 | 0.779355929 | hypothetical protein |
| CNXL_018130 | CNC03030 | -0.372758514 | 0.271524617 | large subunit ribosomal protein L24e |
| CNXL_018140 | CNC03040 | -0.205609863 | 0.724256813 | hypothetical protein |
| CNXL_018150 | | -0.396488615 | 0.857436363 | hypothetical protein |
| CNXL_018160 | CNC03050 | -0.198032351 | 0.715801343 | hypothetical protein |
| CNXL_018170 | CNC03060 | -0.034572497 | 0.965101267 | signal recognition particle receptor subunit alpha |
| CNXL_018180 | CNC03070 | -0.200935492 | 0.687195736 | hypothetical protein |
| CNXL_018190 | CNC03080 | 0.112820344 | 0.820111382 | large subunit ribosomal protein L1 |
| CNXL_018200 | CNC03090 | 0.500579346 | 0.261894145 | pyruvate kinase |
| CNXL_018210 | CNC03100 | 0.056517735 | 0.936410342 | hypothetical protein |
| CNXL_018220 | CNC03110 | 0.690371216 | 0.013896463 | hypothetical protein |
| CNXL_018230 | CNC03120 | 0.255155008 | 0.564998733 | myo-inositol-1 |
| CNXL_018240 | | -0.175832187 | 0.810224824 | transmembrane protein |
| CNXL_018250 | CNC03130 | 0.064093492 | 0.920669589 | hypothetical protein |
| CNXL_018260 | | -0.307839874 | 0.548869305 | transcription initiation factor TFIIH subunit 1 |
| CNXL_018270 | CNC03150 | -0.188819203 | 0.742187954 | hypothetical protein |
| CNXL_018280 | CNC03160 | 0.548563444 | 0.11106406 | compass component swd1 |
| CNXL_018290 | CNC03170 | 0.177310123 | 0.723258981 | chaperone regulator |
| CNXL_018300 | CNC03180 | -0.165354942 | 0.743116933 | hypothetical protein |
| CNXL_018310 | CNC03190 | 0.036514248 | 0.965101267 | ATP-dependent RNA helicase DDX51/DBP6 |
| CNXL_018320 | CNC03200 | -0.067948927 | 0.912929878 | hypothetical protein |
| CNXL_018330 | CNC03210 | 0.557309623 | 0.085860731 | translation initiation factor 4E |
| CNXL_018340 | CNC03220 | -0.224941519 | 0.58400495 | hypothetical protein |
| CNXL_018350 | CNC03230 | -0.888513382 | 0.087121891 | hypothetical protein |
| CNXL_018360 | CNC03240 | 0.098004616 | 0.865015851 | long-chain acyl-CoA synthetase |
| CNXL_018370 | CNC03250 | 0.106381584 | 0.8442367 | vacuolar protein sorting-associated protein VPS35 |
| CNXL_018380 | CNC03260 | -1.631424162 | 7.86357E-13 | peptidyl-prolyl cis-trans isomerase |
| CNXL_018390 | | 0.793557637 | 0.060659334 | tubulin beta chain |
| CNXL_018400 | CNC03270 | -0.556839028 | 0.21296764 | gata family transcription factor |
| CNXL_018410 | CNC03280 | -0.439625194 | 0.481606389 | hypothetical protein |
| CNXL_018420 | CNC03290 | -0.196350856 | 0.776955714 | elongation factor Ts |
| CNXL_018430 | CNC03300 | -0.426147608 | 0.211279804 | tetracycline efflux protein |
| CNXL_018440 | CNC03310 | 1.39987282 | 1.23162E-05 | protein kinase C |
| CNXL_018450 | CNC03320 | 0.871401458 | 0.022368761 | flavoprotein |
| CNXL_018460 | CNC03330 | -0.093751391 | 0.869733568 | hypothetical protein |
| CNXL_018470 | CNC03340 | -0.608975224 | 0.060852917 | hypothetical protein |
| CNXL_018480 | CNC03350 | 0.786440533 | 0.483451258 | two-component-like sensor kinase |
| CNXL_018490 | CNC03360 | -0.004308571 | 0.995735232 | hypothetical protein |
| CNXL_018500 | CNC03370 | 0.189634947 | 0.681547537 | CCR4-NOT transcription complex subunit 2 |
| CNXL_018510 | CNC03380 | -0.220805086 | 0.615722567 | splicing factor 3B subunit 5 |
| CNXL_018520 | CNC03390 | 0.018745116 | 0.983499434 | heparinase II/III family protein |
| CNXL_018530 | CNC03400 | -0.261851411 | 0.671115959 | hypothetical protein |
| CNXL_018540 | CNC03410 | 0.066197034 | 0.924825499 | hypothetical protein |

| | | | | |
|---|---|---|---|---|
| CNXL_018550 | CNC03420 | -0.069556759 | 0.901127763 | Unknown |
| CNXL_018560 | CNC03430 | 0.056589618 | 0.935544021 | elongation factor 1-beta |
| CNXL_018570 | CNC03440 | -0.286110294 | 0.534447681 | alpha-1 |
| CNXL_018580 | | 0.436295565 | 0.162514284 | hypothetical protein |
| CNXL_018590 | CNC03460 | 0.813354756 | 0.002051794 | aryl-alcohol dehydrogenase |
| CNXL_018600 | CNC03480 | -0.060988663 | 0.91726998 | NAD binding dehydrogenase |
| CNXL_018610 | | -0.050262491 | 0.953095191 | nuclear GTP-binding protein |
| CNXL_018620 | CNC03500 | 0.190234764 | 0.678207179 | leucine repeat containing protein |
| CNXL_018630 | CNC03510 | 0.418085605 | 0.258458107 | aldose reductase |
| CNXL_018640 | CNC03520 | -0.948005262 | 0.168782041 | hypothetical protein |
| CNXL_018650 | CNC03530 | -0.087209672 | 0.883532908 | hypothetical protein |
| CNXL_018660 | CNC03540 | 0.021826813 | 0.97922031 | 20S proteasome subunit beta 2 |
| CNXL_018670 | | -0.102771181 | 0.9003865 | cytoplasmic protein |
| CNXL_018680 | CNC03550 | -0.186541538 | 0.871700589 | similar to Avo2 |
| CNXL_018690 | | 0.195033626 | 0.837168643 | peroxin-13 |
| CNXL_018700 | CNC03560 | -0.345615982 | 0.502368015 | hypothetical protein |
| CNXL_018710 | CNC03570 | 0.038760238 | 0.964076726 | sorting nexin-41 |
| CNXL_018720 | CNC03580 | -0.504368526 | 0.27463373 | AMP deaminase |
| CNXL_018730 | CNC03590 | -0.091910691 | 0.917841237 | monosaccharide transporter |
| CNXL_018740 | CNC03600 | 0.062820226 | 0.944263762 | Unknown |
| CNXL_018750 | | 0.256842629 | 0.839081694 | Unknown |
| CNXL_018760 | CNC03610 | -0.257773857 | 0.557382058 | hypothetical protein |
| CNXL_018770 | CNC03630 | 0.001760631 | 0.996833113 | T-complex protein 1 subunit theta |
| CNXL_018780 | | 0.553259537 | 0.851528333 | hypothetical protein |
| CNXL_018790 | | 0.626703716 | 0.233640619 | Unknown |
| CNXL_018800 | CNC03640 | 0.390173177 | 0.294579563 | Unknown |
| CNXL_018810 | | -0.164031195 | 0.802958215 | hypothetical protein |
| CNXL_018820 | CNC03660 | -0.848201015 | 0.068247699 | hypothetical protein |
| CNXL_018830 | CNC03670 | -0.493187268 | 0.258951716 | mitochondrial intermediate peptidase 1 |
| CNXL_018840 | CNC03680 | -0.109473639 | 0.859615039 | dicer |
| CNXL_018850 | | 0.02123053 | 0.983499434 | dicer |
| CNXL_018860 | CNC03700 | 0.567416467 | 0.053786546 | chaperone protein DNAJ |
| CNXL_018870 | CNC03710 | -0.379803756 | 0.496574337 | UTP-glucose-1-phosphate uridylyltransferase |
| CNXL_018880 | CNC03720 | 0.20646676 | 0.692708511 | histone-lysine N-methyltransferase SUV420H |
| CNXL_018890 | CNC03730 | 1.363831822 | 0.000451446 | hypothetical protein |
| CNXL_018900 | CNC03740 | 0.350758769 | 0.467310008 | short-chain dehydrogenase |
| CNXL_018910 | CNC03750 | 0.924497287 | 0.006867703 | short-chain dehydrogenase |
| CNXL_018920 | CNC03760 | 0.013992758 | 0.986381348 | endoplasmic reticulum protein |
| CNXL_018930 | CNC03770 | 0.090790014 | 0.898815058 | small subunit ribosomal protein S12e |
| CNXL_018940 | CNC03780 | 0.09445246 | 0.901645572 | hypothetical protein |
| CNXL_018950 | CNC03790 | -0.345032462 | 0.392734784 | geranylgeranyltransferase-I beta subunit |
| CNXL_018960 | | -1.169288335 | 0.545996243 | hypothetical protein |
| CNXL_018970 | CNC03810 | -0.913114343 | 0.000213456 | Unknown |
| CNXL_018980 | CNC03820 | 0.21698472 | 0.706539593 | hypothetical protein |
| CNXL_018990 | CNC03830 | -0.062858063 | 0.920669589 | hypothetical protein |
| CNXL_019000 | CNC03840 | -0.099740734 | 0.857811508 | inorganic phosphate transporter pho88 |
| CNXL_019010 | CNC03850 | -0.264619366 | 0.582388984 | F-type H+-transporting ATPase subunit G |
| CNXL_019020 | CNC03860 | 0.0164979 | 0.984913368 | phenylalanine-tRNA ligase |
| CNXL_019030 | CNC03870 | -0.028556601 | 0.968420633 | peroxisomal ATP-binding cassette transporter |
| CNXL_019040 | CNC03880 | -0.277732602 | 0.700912881 | 3-hydroxyisobutyrate dehydrogenase |
| CNXL_019050 | CNC03890 | 0.415726605 | 0.28631663 | hypothetical protein |
| CNXL_019060 | | 1.102232518 | 0.002045423 | transcription initiation factor TFIIE subunit alpha |
| CNXL_019070 | CNC03900 | 0.081887487 | 0.920669589 | hypothetical protein |
| CNXL_019080 | | 0.632921365 | 0.057747856 | hypothetical protein |
| CNXL_019090 | CNC03920 | -0.146962032 | 0.814730279 | transcription initiation factor TFIID subunit 11 |
| CNXL_019100 | | -0.128898754 | 0.819461283 | DNA repair and recombination protein RAD54B |
| CNXL_019110 | | -2.258827929 | NA | hypothetical protein |
| CNXL_019120 | CNC03930 | 0.131451765 | 0.794943165 | Unknown |

| | | | | |
|---|---|---|---|---|
| CNXL_019130 | | -0.243516682 | 0.878054651 | hypothetical protein |
| CNXL_019140 | CNC03940 | -0.43001987 | 0.260431503 | Unknown |
| CNXL_019150 | CNC03950 | -0.735676725 | 0.002780834 | exosome complex exonuclease |
| CNXL_019160 | CNC03960 | -0.061407051 | 0.930990489 | hypothetical protein |
| CNXL_019170 | CNC03970 | -0.385900918 | 0.368365274 | inorganic phosphate transporter |
| CNXL_019180 | CNC03980 | 0.042698821 | 0.952560219 | nicotinate-nucleotide pyrophosphorylase |
| CNXL_019190 | CNC03990 | 0.471912587 | 0.127357665 | kynureninase |
| CNXL_019200 | CNC04000 | -0.100222998 | 0.865015851 | dihydrodipicolinate synthetase |
| CNXL_019210 | CNC04010 | 0.01232936 | 0.990703091 | cytoplasmic protein |
| CNXL_019220 | CNC04020 | 0.036639591 | 0.965101267 | hypothetical protein |
| CNXL_019230 | CNC04030 | 0.150624079 | 0.814220979 | methyltransferase |
| CNXL_019240 | CNC04040 | -0.111902073 | 0.854840132 | hypothetical protein |
| CNXL_019250 | CNC04050 | 0.139351158 | 0.833345826 | dihydropteroate synthase |
| CNXL_019260 | CNC04060 | 0.356953085 | 0.495174856 | dephospho-CoA kinase |
| CNXL_019270 | CNC04070 | -0.033825416 | 0.965966884 | hypothetical protein |
| CNXL_019280 | CNC04080 | -0.447044791 | 0.252243073 | nitrogen permease regulator 2 |
| CNXL_019290 | CNC04100 | -0.16222118 | 0.787454471 | galactinol synthase |
| CNXL_019300 | CNC04110 | -0.261064118 | 0.633915651 | hypothetical protein |
| CNXL_019310 | CNC04120 | 0.334372766 | 0.428799713 | mitochondrial chaperone BCS1 |
| CNXL_019320 | CNC04130 | -0.437947451 | 0.286742114 | kynurenine 3-monooxygenase |
| CNXL_019330 | CNC04140 | -0.507344986 | 0.217998625 | dihydroorotate dehydrogenase |
| CNXL_019340 | CNC04150 | 0.516191935 | 0.134021059 | phosphoribosylglycinamide formyltransferase |
| CNXL_019350 | CNC04160 | 0.460114181 | 0.17318254 | 3-deoxy-7-phosphoheptulonate synthase |
| CNXL_019360 | CNC04170 | 0.1088835 | 0.867654918 | putative secreted protein |
| CNXL_019370 | CNC04180 | -0.612000527 | 0.069285289 | hypothetical protein |
| CNXL_019380 | CNC04190 | -0.285676824 | 0.455991866 | dihydroxyacetone kinase |
| CNXL_019390 | | -0.234031582 | 0.745175905 | hypothetical protein |
| CNXL_019400 | CNC04200 | 0.084551725 | 0.877322829 | Unknown |
| CNXL_019410 | CNC04210 | 0.401931513 | 0.434256079 | thioredoxin |
| CNXL_019420 | | 0.272178375 | 0.628160086 | inositol/phosphatidylinositol kinase |
| CNXL_019430 | CNC04220 | -0.191511467 | 0.779355929 | hypothetical protein |
| CNXL_019440 | CNC04230 | -0.540699664 | 0.157355754 | hypothetical protein |
| CNXL_019450 | CNC04240 | -0.209838911 | 0.706539593 | beta-type carbonic anhydrase |
| CNXL_019460 | | 0.836513687 | 0.011634122 | solute carrier family 30 |
| CNXL_019470 | CNC04270 | -0.334806006 | 0.608495206 | hypothetical protein |
| CNXL_019480 | CNC04280 | -0.465853337 | 0.281836596 | tRNA-specific adenosine deaminase 3 |
| CNXL_019490 | CNC04300 | 0.058705427 | 0.934642219 | pumilio-family mRNA binding protein |
| CNXL_019500 | | 0.155989183 | 0.789040329 | ornithine carbamoyltransferase |
| CNXL_019510 | CNC04310 | -0.069632256 | 0.91248949 | Unknown |
| CNXL_019520 | CNC04320 | 1.082638871 | 0.00040763 | glycerol-3-phosphate dehydrogenase |
| CNXL_019530 | CNC04330 | -0.082601798 | 0.889001755 | glycerol-3-phosphate dehydrogenase |
| CNXL_019540 | CNC04340 | 0.06315842 | 0.919196787 | nuclear pore complex protein Nup93 |
| CNXL_019550 | CNC04350 | 0.023211951 | 0.977925118 | rab family GTPase |
| CNXL_019560 | CNC04360 | 0.536958331 | 0.165890121 | glycine cleavage system T protein |
| CNXL_019570 | CNC04370 | -0.34349116 | 0.546233067 | vacuolar membrane protein |
| CNXL_019580 | CNC04380 | -0.062228339 | 0.936783812 | serine/threonine protein kinase |
| CNXL_019590 | CNC04390 | 0.671029024 | 0.04771905 | hypothetical protein |
| CNXL_019600 | CNC04400 | -0.714359568 | 0.068054632 | hypothetical protein |
| CNXL_019610 | CNC04410 | -0.109310913 | 0.880060691 | bloom syndrome protein |
| CNXL_019620 | | 0.389516581 | 0.456456998 | hypothetical protein |
| CNXL_019630 | CNC04420 | -0.03214574 | 0.965101267 | hypothetical protein |
| CNXL_019640 | CNC04430 | -0.451230672 | 0.404910404 | argininosuccinate lyase |
| CNXL_019650 | CNC04440 | -0.056695766 | 0.928647164 | amino-acid acetyltransferase |
| CNXL_019660 | | 0.365728069 | 0.497967352 | ubiquitin-like protein |
| CNXL_019670 | CNC04460 | -1.147320814 | 0.000535754 | hypothetical protein |
| CNXL_019680 | CNC04470 | -1.092894966 | 0.000112224 | protein arginine N-methyltransferase 5 |
| CNXL_019690 | CNC04480 | 0.616510447 | 0.092987507 | C-24 |
| CNXL_019700 | CNC04490 | -0.072247824 | 0.912595206 | endopeptidase |

| | | | | |
|---|---|---|---|---|
| CNXL_019710 | CNC04500 | 0.379212816 | 0.35683476 | NADH dehydrogenase |
| CNXL_019720 | CNC04510 | 0.173105224 | 0.697146261 | sterol 3-beta-glucosyltransferase |
| CNXL_019730 | CNC04520 | 0.14200742 | 0.813394551 | hypothetical protein |
| CNXL_019740 | CNC04530 | -0.050067448 | 0.956370213 | ribonuclease P/MRP protein subunit POP5 |
| CNXL_019750 | CNC04540 | 0.021343334 | 0.981240834 | hypothetical protein |
| CNXL_019760 | | 0.040064092 | 0.96580389 | small subunit ribosomal protein S17 |
| CNXL_019770 | CNC04560 | 0.411458437 | 0.526319198 | hypothetical protein |
| CNXL_019780 | CNC04570 | 0.421686046 | 0.309128838 | hypothetical protein |
| CNXL_019790 | CNC04580 | -0.175779218 | 0.755239836 | cytochrome P450 monooxygenase pc-2 |
| CNXL_019800 | CNC04590 | 0.983045479 | 0.006151884 | hypothetical protein |
| CNXL_019810 | CNC04600 | 0.026435837 | 0.981851244 | hypothetical protein |
| CNXL_019820 | CNC04610 | -0.17379101 | 0.767511661 | hypothetical protein |
| CNXL_019830 | CNC04620 | 0.208043218 | 0.700697818 | hypothetical protein |
| CNXL_019840 | CNC04630 | -1.150296347 | 0.008451635 | endoplasmic reticulum protein |
| CNXL_019850 | CNC04640 | -0.832702173 | 0.000961171 | thymidylate kinase |
| CNXL_019860 | CNC04660 | -0.253955776 | 0.81790448 | hypothetical protein |
| CNXL_019870 | CNC04670 | 0.93038873 | 0.000112916 | glutathione transferase |
| CNXL_019880 | CNC04680 | 0.007143894 | 0.992176872 | glucan endo-1 |
| CNXL_019890 | CNC04690 | -0.203273247 | 0.802348605 | threonine aldolase |
| CNXL_019900 | CNC04700 | -0.526984967 | 0.181933117 | 4-aminobutyrate transaminase |
| CNXL_019910 | CNC04710 | 0.276440458 | 0.517631308 | amidophosphoribosyltransferase |
| CNXL_019920 | CNC04720 | -0.250794079 | 0.632998683 | peroxiredoxin Q/BCP |
| CNXL_019930 | CNC04730 | -0.357523135 | 0.37369301 | oligosaccharyltransferase complex subunit delta |
| CNXL_019940 | | -0.424183592 | 0.495942837 | potassium ion transporter |
| CNXL_019950 | CNC04740 | -0.125519705 | 0.848021859 | Unknown |
| CNXL_019960 | CNC04750 | -0.186248092 | 0.662061256 | hypothetical protein |
| CNXL_019970 | CNC04760 | 0.137577321 | 0.798976008 | adenylosuccinate synthetase |
| CNXL_019980 | CNC04770 | -0.293693143 | 0.415823204 | NADH kinase |
| CNXL_019990 | CNC04790 | -0.204289748 | 0.810224824 | endo-1 |
| CNXL_020000 | CNC04800 | 0.334992028 | 0.589709705 | histidinol-phosphatase |
| CNXL_020010 | | 1.342635226 | 0.420919628 | aprataxin |
| CNXL_020020 | | 1.996232506 | 3.76758E-09 | Unknown |
| CNXL_020030 | | NA | NA | hypothetical protein |
| CNXL_020040 | | 1.484927388 | NA | Unknown |
| CNXL_020050 | CNC04810 | -0.020435519 | 0.982102702 | Unknown |
| CNXL_020060 | CNC04820 | 0.04890739 | 0.936783812 | pantothenate kinase |
| CNXL_020070 | CNC04830 | -0.544707633 | 0.323308793 | phosphatidylinositol phospholipase C |
| CNXL_020080 | | -0.689047158 | 0.30897417 | tryptophan 2 |
| CNXL_020090 | CNC04840 | -0.339268723 | 0.397048245 | hypothetical protein |
| CNXL_020100 | CNC04850 | 0.129015725 | 0.835972626 | folylpolyglutamate synthetase |
| CNXL_020110 | | -3.012123974 | 3.55611E-05 | GPI mannosyltransferase 2 |
| CNXL_020120 | CNC04860 | 0.002922396 | 0.996472611 | Unknown |
| CNXL_020130 | CNC04870 | -0.806457163 | 0.024632712 | signal peptidase I |
| CNXL_020140 | CNC04880 | 0.050350856 | 0.944879244 | hypothetical protein |
| CNXL_020150 | CNC04890 | 0.311681164 | 0.38167154 | hypothetical protein |
| CNXL_020160 | CNC04900 | 1.508844964 | 1.69051E-05 | ornithine decarboxylase |
| CNXL_020170 | CNC04910 | 0.059756516 | 0.932840267 | hypothetical protein |
| CNXL_020180 | CNC04920 | -0.106302783 | 0.865068963 | hypothetical protein |
| CNXL_020190 | CNC04930 | 0.084063175 | 0.878054651 | chloride channel protein |
| CNXL_020200 | CNC04940 | -0.245528191 | 0.634625988 | GTP-binding protein YchF |
| CNXL_020210 | | -0.708157672 | 0.137569503 | cytidine deaminase |
| CNXL_020220 | CNC04960 | -0.440992692 | 0.189297101 | hypothetical protein |
| CNXL_020230 | CNC04970 | -0.407508356 | 0.495174856 | ras-like gtp-binding protein |
| CNXL_020240 | CNC04980 | -0.583451174 | 0.093042002 | DNA replication complex GINS protein PSF2 |
| CNXL_020250 | CNC04990 | 0.030562355 | 0.968757042 | capsule-associated protein |
| CNXL_020260 | CNC05000 | -0.12089879 | 0.833396996 | 20S proteasome subunit beta 4 |
| CNXL_020270 | CNC05010 | -0.528604309 | 0.308584752 | Sec7 domain-containing protein |
| CNXL_020280 | CNC05020 | -0.19842102 | 0.833396996 | gamma-glutamyltransferase |

| | | | | |
|---|---|---|---|---|
| CNXL_020290 | CNC05030 | -0.155568158 | 0.872355538 | hypothetical protein |
| CNXL_020300 | CNC05040 | 0.380089722 | 0.326972642 | hypothetical protein |
| CNXL_020310 | CNC05050 | 1.025419986 | 0.000670786 | endoplasmic reticulum protein |
| CNXL_020320 | CNC05060 | 0.706757554 | 0.016151708 | phosphatidylinositol glycan |
| CNXL_020330 | | -0.264657494 | 0.7013463 | hypothetical protein |
| CNXL_020340 | CNC05070 | 0.287396362 | 0.452146814 | hypothetical protein |
| CNXL_020350 | CNC05090 | -0.208905941 | 0.639965483 | dynamin GTPase |
| CNXL_020360 | CNC05100 | -0.069227693 | 0.921294394 | inositol hexaphosphate kinase 1 |
| CNXL_020370 | | 2.969141094 | 0.000127098 | RNA-binding protein 8A |
| CNXL_020380 | CNC05110 | -1.103582737 | 0.000328158 | hypothetical protein |
| CNXL_020390 | CNC05120 | -0.155538616 | 0.773509113 | hypothetical protein |
| CNXL_020400 | CNC05130 | 0.192826947 | 0.827557138 | U6 snRNA-associated Sm-like protein LSm4 |
| CNXL_020410 | CNC05140 | 0.759919243 | 0.006384724 | hypothetical protein |
| CNXL_020420 | | 0.418857662 | 0.264527288 | alcohol dehydrogenase |
| CNXL_020430 | CNC05150 | 0.190595213 | 0.732889168 | hypothetical protein |
| CNXL_020440 | CNC05160 | 0.071653462 | 0.916084262 | hypothetical protein |
| CNXL_020450 | CNC05170 | -0.225774494 | 0.634625988 | ubiquitin-conjugating enzyme |
| CNXL_020460 | | -0.162355364 | 0.763757096 | tRNA |
| CNXL_020470 | CNC05200 | 0.421600598 | 0.233522268 | hypothetical protein |
| CNXL_020480 | CNC05210 | -0.30982848 | 0.553726767 | hypothetical protein |
| CNXL_020490 | CNC05220 | -0.06224091 | 0.927929791 | hypothetical protein |
| CNXL_020500 | CNC05230 | 0.247044353 | 0.667605477 | hypothetical protein |
| CNXL_020510 | CNC05240 | 0.009189177 | 0.992176872 | hypothetical protein |
| CNXL_020520 | CNC05250 | -0.446937367 | 0.25879631 | hypothetical protein |
| CNXL_020530 | CNC05260 | -0.337895513 | 0.417866535 | serine threonine protein kinase |
| CNXL_020540 | CNC05270 | -0.306574753 | 0.60949078 | F-type H+-transporting ATPase subunit F |
| CNXL_020550 | CNC05280 | -0.335376176 | 0.523679914 | hypothetical protein |
| CNXL_020560 | CNC05290 | -0.045167137 | 0.954346886 | acetyl-CoA C-acetyltransferase |
| CNXL_020570 | CNC05300 | 0.280854456 | 0.498081161 | hydrolase |
| CNXL_020580 | CNC05310 | -0.108812206 | 0.856460878 | hypothetical protein |
| CNXL_020590 | CNC05320 | 0.027682992 | 0.970190057 | hypothetical protein |
| CNXL_020600 | CNC05330 | 0.382993297 | 0.247252358 | large subunit ribosomal protein L14 |
| CNXL_020610 | CNC05340 | -0.137492956 | 0.779355929 | histone H1/5 |
| CNXL_020620 | | 0.229058287 | 0.625674534 | d-arabinitol 2-dehydrogenase |
| CNXL_020630 | CNC05350 | -0.227043935 | 0.64162027 | hypothetical protein |
| CNXL_020640 | CNC05360 | -0.087040652 | 0.86860514 | protein disulfide-isomerase |
| CNXL_020650 | CNC05370 | 0.151983055 | 0.781361786 | large subunit ribosomal protein L5e |
| CNXL_020660 | CNC05390 | -0.831437339 | 0.08214898 | transcription initiation factor TFIID subunit 1 |
| CNXL_020670 | CNC05400 | 0.367256454 | 0.546233067 | virulence related protein of unknown function |
| CNXL_020680 | CNC05410 | 0.181426752 | 0.777634355 | hypothetical protein |
| CNXL_020690 | CNC05420 | 0.350832989 | 0.358799682 | hypothetical protein |
| CNXL_020700 | CNC05430 | -0.095170778 | 0.944263762 | quinone oxidoreductase |
| CNXL_020710 | CNC05440 | 0.078144102 | 0.890147007 | Unknown |
| CNXL_020720 | CNC05450 | -0.426888945 | 0.496574337 | hypothetical protein |
| CNXL_020730 | CNC05460 | 0.013537515 | 0.987967132 | malonic semialdehyde reductase |
| CNXL_020740 | CNC05470 | -0.149152405 | 0.778362394 | CCR4-NOT transcription complex subunit 1 |
| CNXL_020750 | CNC05480 | -0.065890306 | 0.910345571 | translocation protein SEC63 |
| CNXL_020760 | CNC05490 | -0.16512226 | 0.762884467 | cytochrome c oxidase subunit 6a |
| CNXL_020770 | CNC05500 | -0.454268097 | 0.213349287 | peroxin-3 |
| CNXL_020780 | CNC05510 | 0.569927648 | 0.044654589 | calcium/proton exchanger |
| CNXL_020790 | CNC05530 | 1.129021586 | 0.000670496 | hypothetical protein |
| CNXL_020800 | CNC05540 | 0.104868503 | 0.874872772 | cytoplasmic protein |
| CNXL_020810 | CNC05550 | 0.598482938 | 0.044774395 | phosphate-repressible vacuolar acid phosphatase |
| CNXL_020820 | CNC05560 | 0.004570517 | 0.995735232 | hypothetical protein |
| CNXL_020830 | CNC05570 | 0.083125417 | 0.907858192 | nucleolar protein 15 |
| CNXL_020840 | CNC05580 | 0.084451172 | 0.889396164 | SCYL protein kinase |
| CNXL_020850 | CNC05590 | 0.019356798 | 0.982102702 | peptide chain release factor 1 |
| CNXL_020860 | CNC05600 | 0.03524195 | 0.965465882 | thioredoxin |

| | | | | |
|---|---|---|---|---|
| CNXL_020870 | CNC05610 | 0.060242422 | 0.934642219 | hypothetical protein |
| CNXL_020880 | | 0.362485087 | 0.3985333 | hypothetical protein |
| CNXL_020890 | CNC05640 | -0.261573758 | 0.65287141 | tuberin |
| CNXL_020900 | CNC05650 | 0.185844453 | 0.716889472 | hypothetical protein |
| CNXL_020910 | CNC05660 | 0.019693411 | 0.981614017 | transcription factor IWS1 |
| CNXL_020920 | CNC05670 | -0.067062949 | 0.935508486 | farnesyl-diphosphate farnesyltransferase |
| CNXL_020930 | CNC05680 | -0.328852535 | 0.670362199 | xaa-Pro aminopeptidase |
| CNXL_020940 | CNC05690 | 0.168254825 | 0.814730279 | hypothetical protein |
| CNXL_020950 | CNC05700 | 0.085111324 | 0.918701468 | ferroxidase |
| CNXL_020960 | CNC05710 | -0.050706045 | 0.949841766 | iron permease |
| CNXL_020970 | CNC05730 | -0.322891546 | 0.434588143 | RNA exonuclease 4 |
| CNXL_020980 | CNC05740 | -0.378018386 | 0.414496592 | CMGC/DYRK/PRP4 protein kinase |
| CNXL_020990 | CNC05750 | -0.501311244 | 0.200048803 | anaphase-promoting complex subunit 8 |
| CNXL_021000 | | -0.006239939 | 0.994325965 | hypothetical protein |
| CNXL_021010 | CNC05760 | 1.462230431 | 0.35231862 | hypothetical protein |
| CNXL_021020 | CNC05770 | 0.484427134 | 0.397567134 | carboxypeptidase |
| CNXL_021030 | CNC05780 | 0.507586317 | 0.186130848 | hypothetical protein |
| CNXL_021040 | CNC05790 | 0.934759933 | 0.002032879 | phosphatidylinositol-specific phospholipase C |
| CNXL_021050 | | 0.785018668 | 0.02932986 | thioesterase |
| CNXL_021060 | CNC05800 | -0.23661327 | 0.633023767 | Unknown |
| CNXL_021070 | CNC05810 | -0.494497778 | 0.252374871 | mapk signaling pathway adaptor protein |
| CNXL_021080 | CNC05820 | -0.311122113 | 0.544657787 | transcriptional repressor NF-X1 |
| CNXL_021090 | CNC05830 | 0.090367046 | 0.866174286 | tRNA-splicing endonuclease subunit Sen2 |
| CNXL_021100 | CNC05840 | 0.507653562 | 0.168034195 | voltage-dependent anion channel protein 2 |
| CNXL_021110 | CNC05850 | -0.054678046 | 0.941658589 | hypothetical protein |
| CNXL_021120 | CNC05860 | 0.191759164 | 0.701847486 | riboflavin kinase |
| CNXL_021130 | CNC05870 | 0.492386494 | 0.095784948 | metal resistance protein ycf1 |
| CNXL_021140 | CNC05880 | -0.083846058 | 0.933346134 | hypothetical protein |
| CNXL_021150 | CNC05890 | -0.20378223 | 0.789040329 | hypothetical protein |
| CNXL_021160 | CNC05900 | -0.156560966 | 0.792789868 | membrane dipeptidase |
| CNXL_021170 | CNC05910 | -0.494254135 | 0.235460968 | paired amphipathic helix protein Sin3a |
| CNXL_021180 | CNC05920 | -0.258735432 | 0.557606572 | U3 small nucleolar RNA-associated protein 13 |
| CNXL_021190 | CNC05930 | -0.165587026 | 0.797485767 | hypothetical protein |
| CNXL_021200 | CNC05940 | -0.029073387 | 0.966608981 | sarcosine oxidase |
| CNXL_021210 | CNC05950 | -0.128361463 | 0.807491917 | nuclear mRNA splicing protein |
| CNXL_021220 | | 0.680127875 | 0.499968632 | ADP-ribose pyrophosphatase |
| CNXL_021230 | CNC05960 | -0.180503712 | 0.71868682 | hypothetical protein |
| CNXL_021240 | CNC05970 | 0.379034062 | 0.372044973 | acetyltransferase |
| CNXL_021250 | CNC05980 | -0.810983619 | 0.003053889 | PX domain-containing protein |
| CNXL_021260 | CNC05990 | 0.200075927 | 0.633398513 | nuclear protein |
| CNXL_021270 | CNC06000 | -0.399314164 | 0.453868281 | cofilin |
| CNXL_021280 | CNC06010 | 0.069121761 | 0.926407741 | actin cross-linking protein |
| CNXL_021290 | CNC06020 | 0.65676959 | 0.035994459 | solute carrier family 39 |
| CNXL_021300 | CNC06030 | 0.193987579 | 0.674850767 | hypothetical protein |
| CNXL_021310 | CNC06060 | 0.662908029 | 0.048176746 | RNA-binding protein with serine-rich domain 1 |
| CNXL_021320 | CNC06070 | -0.046075281 | 0.962194201 | flavoprotein oxygenase |
| CNXL_021330 | CNC06080 | 0.449557237 | 0.210324483 | protein STU1 |
| CNXL_021340 | CNC06090 | 0.46338524 | 0.30897417 | nuclear protein |
| CNXL_021350 | CNC06100 | -0.100935975 | 0.846421979 | hypothetical protein |
| CNXL_021360 | CNC06110 | -0.113002607 | 0.884028948 | small subunit ribosomal protein S19e |
| CNXL_021370 | CNC06120 | 1.002150508 | 0.000223039 | multiple RNA-binding domain-containing protein 1 |
| CNXL_021380 | CNC06140 | 0.32675961 | 0.428799713 | hypothetical protein |
| CNXL_021390 | CNC06150 | -0.020732382 | 0.985173026 | chromatin structure-remodeling complex subunit SFH1 |
| CNXL_021400 | CNC06160 | -0.315009029 | 0.541560974 | riboflavin synthase |
| CNXL_021410 | CNC06170 | -0.107996332 | 0.930990489 | cytoplasmic tRNA 2-thiolation protein 2 |
| CNXL_021420 | CNC06180 | 0.559364167 | 0.130833608 | hypothetical protein |
| CNXL_021430 | CNC06190 | -0.112395393 | 0.865062469 | hypothetical protein |
| CNXL_021440 | CNC06200 | -0.322077747 | 0.603532035 | hypothetical protein |

| | | | | |
|---|---|---|---|---|
| CNXL_021450 | CNC06210 | -0.120733886 | 0.830440343 | putative ER membrane protein |
| CNXL_021460 | CNC06220 | -0.799150347 | 0.132308784 | enoyl-CoA hydratase/isomerase |
| CNXL_021470 | | -0.758999437 | NA | glycerate-and formate-dehydrogenase |
| CNXL_021480 | | NA | NA | Unknown |
| CNXL_021490 | CNC06240 | 0.383258086 | 0.311813532 | Unknown |
| CNXL_021500 | | 0.401744154 | 0.634625988 | WD-repeat protein |
| CNXL_021510 | CNC06250 | -0.545131781 | 0.12793218 | hypothetical protein |
| CNXL_021520 | CNC06260 | 1.339293849 | 0.000112224 | hypothetical protein |
| CNXL_021530 | CNC06280 | 0.327684883 | 0.370509035 | chlorophyll synthesis pathway protein BchC |
| CNXL_021540 | CNC06290 | 0.084278416 | 0.906691628 | calmodulin |
| CNXL_021550 | | 0.137121391 | 0.866730187 | cytoplasmic protein |
| CNXL_021560 | CNC06300 | 0.22011748 | 0.659118995 | hypothetical protein |
| CNXL_021570 | CNC06310 | -0.826945579 | 0.02395945 | sorting nexin-4 |
| CNXL_021580 | CNC06320 | -0.671267764 | 0.087042518 | CORD and CS domain-containing protein |
| CNXL_021590 | CNC06330 | 0.413261673 | 0.358272717 | blocked early in transport 1 |
| CNXL_021600 | CNC06340 | -0.311148701 | 0.508841455 | gata family transcription factor |
| CNXL_021610 | CNC06350 | -0.0788754 | 0.893955723 | pre-rRNA-processing protein TSR3 |
| CNXL_021620 | CNC06360 | 0.03420568 | 0.962640129 | splicing factor 3A subunit 3 |
| CNXL_021630 | CNC06370 | -0.145600224 | 0.794943165 | ATP-dependent RNA helicase FAL1 |
| CNXL_021640 | CNC06380 | 0.016977546 | 0.987336046 | WD-repeat protein |
| CNXL_021650 | CNC06390 | -0.721341235 | 0.110539145 | S-adenosylmethionine-dependent methyltransferase |
| CNXL_021660 | CNC06400 | -0.042564568 | 0.95086529 | hypothetical protein |
| CNXL_021670 | CNC06410 | 0.418485389 | 0.276952897 | histidyl-tRNA synthetase |
| CNXL_021680 | CNC06420 | -0.446767445 | 0.30555532 | AP-3 complex subunit beta |
| CNXL_021690 | | -0.436333261 | 0.27463373 | taurine catabolism dioxygenase TauD |
| CNXL_021700 | CNC06430 | -1.211153931 | 1.88383E-06 | hypothetical protein |
| CNXL_021710 | CNC06440 | -0.204111202 | 0.702849493 | dehydrogenase |
| CNXL_021720 | CNC06450 | -0.496904384 | 0.249559817 | myo-inositol-1-phosphate synthase |
| CNXL_021730 | CNC06460 | -0.038182461 | 0.96275474 | hypothetical protein |
| CNXL_021740 | CNC06470 | -0.606930654 | 0.091426576 | virulence associated ATP-dependent mRNA helicase |
| CNXL_021750 | CNC06480 | -0.129349969 | 0.794943165 | myosin heavy chain |
| CNXL_021760 | | 1.327816038 | 6.15138E-05 | amino acid permease |
| CNXL_021770 | CNC06490 | -0.509371501 | 0.200496092 | hypothetical protein |
| CNXL_021780 | CNC06500 | -0.148475007 | 0.820111382 | rho GTPase activator |
| CNXL_021790 | CNC06510 | 0.161843566 | 0.809974479 | phosphatidylinositol phosphate phosphatase |
| CNXL_021800 | CNC06520 | -0.721401096 | 0.015979048 | DNA helicase II/ATP-dependent DNA helicase PcrA |
| CNXL_021810 | CNC06530 | -0.700567416 | 0.044310031 | transcription factor |
| CNXL_021820 | CNC06540 | -0.506583268 | 0.153256606 | solute carrier family 25 |
| CNXL_021830 | CNC06550 | -4.235250843 | 1.1638E-86 | hypothetical protein |
| CNXL_021840 | CNC06560 | -0.364348989 | 0.499968632 | hypothetical protein |
| CNXL_021850 | CNC06570 | -0.51913329 | 0.281476137 | cleavage and polyadenylation specificity factor subunit 4 |
| CNXL_021860 | CNC06590 | 0.140550765 | 0.771763303 | hypothetical protein |
| CNXL_021870 | CNC06600 | -0.107243728 | 0.889001755 | mitogen-activated protein kinase |
| CNXL_021880 | CNC06610 | 1.180396642 | 1.9828E-05 | phenylalanine-tRNA ligase |
| CNXL_021890 | CNC06620 | 0.845341363 | 0.012822477 | metallo-beta-lactamase |
| CNXL_021900 | CNC06630 | 0.663382499 | 0.070628776 | histone-arginine methyltransferase CARM1 |
| CNXL_021910 | CNC06640 | 0.162886558 | 0.794331499 | endonuclease/exonuclease/phosphatase |
| CNXL_021920 | CNC06650 | 0.64218112 | 0.051379578 | phosphoglycerate mutase |
| CNXL_021930 | CNC06660 | 0.108963983 | 0.859615039 | mitochondrial metalloendopeptidase OMA1 |
| CNXL_021940 | CNC06670 | -0.64181718 | 0.051609433 | V-type H+-transporting ATPase subunit C |
| CNXL_021950 | | -0.263144793 | 0.602349674 | xylitol dehydrogenase |
| CNXL_021960 | CNC06680 | 0.398671038 | 0.372306093 | hypothetical protein |
| CNXL_021970 | CNC06690 | -2.676022922 | 1.12708E-37 | hypothetical protein |
| CNXL_021980 | | -0.299620226 | 0.529472069 | hypothetical protein |
| CNXL_021990 | | 0.651309601 | 0.75185116 | Unknown |
| CNXL_022000 | CNC06870 | 0.720211438 | 0.118110154 | Unknown |
| CNXL_022010 | CNC06710 | -0.081717292 | 0.911130835 | Unknown |
| CNXL_022020 | CNC06720 | 0.159756738 | 0.816572532 | hypothetical protein |

| | | | | |
|---|---|---|---|---|
| CNXL_022030 | CNC06730 | -0.221580177 | 0.703985737 | hypothetical protein |
| CNXL_022040 | CNC06740 | 0.125335414 | 0.858651348 | hypothetical protein |
| CNXL_022050 | CNC06750 | -0.81488302 | 0.110250219 | protein CGI121 |
| CNXL_022060 | | -0.098453897 | 0.885449489 | hypothetical protein |
| CNXL_022070 | CNC06760 | 0.371934455 | 0.45163285 | hypothetical protein |
| CNXL_022080 | CNC06770 | 0.763133386 | 0.163322941 | hypothetical protein |
| CNXL_022090 | CNC06780 | 0.107149784 | 0.854327858 | Unknown |
| CNXL_022100 | CNC06790 | -0.066307939 | 0.932840267 | major facilitator superfamily transporter |
| CNXL_022110 | CNC06800 | 1.019394425 | 0.057679889 | hypothetical protein |
| CNXL_022120 | CNC06810 | 0.339989438 | 0.616861062 | taurine dioxygenase |
| CNXL_022130 | CNC06820 | -0.25376299 | 0.748609863 | Unknown |
| CNXL_022140 | CNC06830 | -0.083918941 | 0.927235611 | arylsulfatase |
| CNXL_022150 | CNC06840 | -0.026016629 | 0.977965004 | integral membrane protein |
| CNXL_022160 | CNC06850 | -0.037380826 | 0.95901729 | protein phosphatase 5 |
| CNXL_022170 | | 0.217792027 | 0.886997541 | myo-inositol 2-dehydrogenase |
| CNXL_022180 | | -0.112616705 | 0.843098307 | hypothetical protein |
| CNXL_022190 | CNC06860 | -1.269937432 | NA | hypothetical protein |
| CNXL_022200 | CNC06870 | -0.473806021 | 0.621994485 | Unknown |
| CNXL_022210 | | 1.189277791 | 6.63496E-06 | Unknown |
| CNXL_022220 | CNC06880 | 0.499030032 | 0.133115992 | hypothetical protein |
| CNXL_022230 | | 0.223366447 | 0.76831241 | hypothetical protein |
| CNXL_022240 | CNC06890 | -0.04790402 | 0.951846328 | Unknown |
| CNXL_022250 | CNC06900 | -0.325622032 | 0.519652797 | hypothetical protein |
| CNXL_022260 | CNC06920 | -0.98444807 | 0.762405378 | hypothetical protein |
| CNXL_022270 | CNC06910 | 0.88665355 | 0.004341948 | maltose O-acetyltransferase |
| CNXL_022280 | CNC06940 | -0.149402394 | 0.770559628 | Unknown |
| CNXL_022290 | CNC06950 | 0.600276168 | 0.106221376 | large subunit ribosomal protein L15-A |
| CNXL_022300 | CNC06960 | 0.154879044 | 0.779355929 | D-lactate dehydrogenase |
| CNXL_022310 | CNC06970 | -0.149337131 | 0.78519633 | hypothetical protein |
| CNXL_022320 | CNC06980 | -0.078832486 | 0.908809603 | solute carrier family 25 |
| CNXL_022330 | CNC06990 | -0.162486767 | 0.742187954 | hypothetical protein |
| CNXL_022340 | CNC07000 | 0.301270087 | 0.597306649 | large subunit ribosomal protein L12 |
| CNXL_022350 | CNC07010 | 0.39466883 | 0.388278926 | hypothetical protein |
| CNXL_022360 | CNC07020 | -0.720975034 | 0.02517951 | monooxygenase |
| CNXL_022370 | CNC07030 | -0.012593743 | 0.987964663 | serine palmitoyltransferase |
| CNXL_022380 | CNC07040 | -0.18626833 | 0.707988087 | hypothetical protein |
| CNXL_022390 | CNC07050 | -1.827103862 | 1.15058E-11 | sphingolipid C9-methyltransferase |
| CNXL_022400 | | 0.265397741 | 0.635142308 | hypothetical protein |
| CNXL_022410 | CNC07070 | 0.528120933 | 0.090552036 | hypothetical protein |
| CNXL_022420 | CNC07080 | 0.459922985 | 0.280284344 | endoplasmic reticulum protein |
| CNXL_022430 | CNC07090 | -0.154547112 | 0.779355929 | hypothetical protein |
| CNXL_022440 | | -0.880865417 | 0.237718322 | NADH dehydrogenase |
| CNXL_022450 | | 0.026609078 | 0.983980369 | Unknown |
| CNXL_022460 | CNC07100 | 0.083850139 | 0.920854816 | Unknown |
| CNXL_022470 | CNC07110 | -0.542469679 | 0.249506414 | Unknown |
| CNXL_022480 | CNC07120 | -0.353796142 | 0.567369556 | CDP-diacylglycerol-serine O-phosphatidyltransferase |
| CNXL_022490 | CNC07130 | 0.014342302 | 0.987964663 | hypothetical protein |
| CNXL_022500 | CNC07140 | 0.117236973 | 0.862678265 | solute carrier family 35 |
| CNXL_022510 | CNC07150 | 0.48641852 | 0.350443288 | hypothetical protein |
| CNXL_022520 | CNC07160 | -2.46849039 | 3.05674E-31 | 5' flap endonuclease |
| CNXL_022530 | CNC07170 | 0.244232063 | 0.641901571 | flavohemoglobin |
| CNXL_022540 | CNC07180 | -0.233575565 | 0.660204343 | hypothetical protein |
| CNXL_022550 | | -0.218462426 | 0.756597645 | hypothetical protein |
| CNXL_022560 | | -0.738284552 | 0.779355929 | hypothetical protein |
| CNXL_022570 | CND00030 | 0.650642248 | 0.058979422 | Unknown |
| CNXL_022580 | CND00040 | 1.543675205 | 1.16596E-06 | putative O-acetyltransferase |
| CNXL_022590 | CND00070 | -0.210095942 | 0.700912881 | transketolase |
| CNXL_022600 | | 0.352221161 | 0.482118261 | myo-inositol transporter |

| | | | | |
|---|---|---|---|---|
| CNXL_022610 | | 0.645950111 | 0.029044252 | Unknown |
| CNXL_022620 | CND00090 | -0.859121079 | 0.003196301 | Unknown |
| CNXL_022630 | | 0.418338052 | 0.195548343 | ATP-binding cassette transporter |
| CNXL_022640 | CND00100 | 0.262849229 | 0.634728039 | hypothetical protein |
| CNXL_022650 | CND00110 | 0.0126676 | 0.988641003 | hypothetical protein |
| CNXL_022660 | CND00120 | -0.197519163 | 0.746537956 | ATP-dependent DNA helicase |
| CNXL_022670 | CND00130 | -0.460673604 | 0.39417374 | hypothetical protein |
| CNXL_022680 | CND00140 | 0.410558431 | 0.30897417 | multisite-specific tRNA |
| CNXL_022690 | CND00150 | -0.257380215 | 0.539585872 | rab family protein |
| CNXL_022700 | CND00160 | -0.120590212 | 0.838178569 | ferric reductase |
| CNXL_022710 | | 0.513904394 | 0.592750411 | adenylate kinase |
| CNXL_022720 | CND00180 | -0.996994487 | 0.00018715 | hypothetical protein |
| CNXL_022730 | | -1.284100734 | 0.376373253 | glutamate dehydrogenase |
| CNXL_022740 | CND00190 | -0.062117988 | 0.927235611 | Unknown |
| CNXL_022750 | CND00200 | -0.391341685 | 0.398556028 | hypothetical protein |
| CNXL_022760 | | 0.783302901 | 0.179780745 | Zn2-Cys6 zinc-finger transcription factor |
| CNXL_022770 | CND00210 | 0.477571044 | 0.253643137 | Unknown |
| CNXL_022780 | CND00220 | -0.127498997 | 0.928118121 | exocyst complex component protein |
| CNXL_022790 | CND00240 | -0.154184391 | 0.755572574 | hypothetical protein |
| CNXL_022800 | CND00250 | -1.444092691 | 0.006227782 | adenosylhomocysteinase |
| CNXL_022810 | CND00260 | 0.292388962 | 0.495140189 | hypothetical protein |
| CNXL_022820 | CND00280 | 0.005106397 | 0.995735232 | calcineurin b |
| CNXL_022830 | CND00290 | -0.631296754 | 0.112183936 | hypothetical protein |
| CNXL_022840 | CND00300 | 0.266403651 | 0.510243991 | hypothetical protein |
| CNXL_022850 | CND00310 | 0.157147964 | 0.797632564 | ATP-binding cassette |
| CNXL_022860 | CND00320 | 0.071201666 | 0.915477258 | hypothetical protein |
| CNXL_022870 | CND00340 | -0.242299885 | 0.55888162 | hypothetical protein |
| CNXL_022880 | CND00350 | -0.009020902 | 0.992176872 | hypothetical protein |
| CNXL_022890 | CND00370 | 0.952886768 | 0.001034672 | zinc ion transporter |
| CNXL_022900 | CND00380 | 0.64688971 | 0.057747856 | transcription factor |
| CNXL_022910 | CND00390 | 1.449054019 | 3.67299E-05 | putative glucosidase |
| CNXL_022920 | CND00400 | -0.389740152 | 0.500910612 | multidrug efflux pump |
| CNXL_022930 | CND00410 | -0.495251126 | 0.502368015 | hypothetical protein |
| CNXL_022940 | CND00420 | 0.632170885 | 0.098322722 | hypothetical protein |
| CNXL_022950 | CND00430 | 0.296624344 | 0.53679353 | hypothetical protein |
| CNXL_022960 | CND00440 | -0.349804478 | 0.377508109 | hypothetical protein |
| CNXL_022970 | CND00450 | 1.703221629 | 2.7694E-07 | aflatoxin efflux pump AFLT |
| CNXL_022980 | CND00460 | -0.557556531 | 0.161051858 | MFS transporter |
| CNXL_022990 | CND00470 | 0.3185164 | 0.485277158 | nucleolar pre-ribosomal-associated protein 1 |
| CNXL_023000 | CND00480 | -0.499830042 | 0.142026987 | Unknown |
| CNXL_023010 | CND00490 | -4.209516469 | 7.53872E-80 | phosphopantothenoylcysteine decarboxylase |
| CNXL_023020 | CND00500 | 0.015097073 | 0.9898378 | hypothetical protein |
| CNXL_023030 | CND00510 | -0.485652034 | 0.332119185 | phytanoyl-CoA dioxygenase |
| CNXL_023040 | CND00530 | 0.010461635 | 0.98960551 | amino acid transporter |
| CNXL_023050 | CND00550 | -0.10125671 | 0.885449489 | putative urea transporter |
| CNXL_023060 | CND00560 | -0.686402981 | 0.149837648 | putative beta-glucan synthase |
| CNXL_023070 | CND00570 | 0.023519767 | 0.981240834 | hypothetical protein |
| CNXL_023080 | CND00580 | 0.178030316 | 0.784612376 | transcription factor |
| CNXL_023090 | CND00600 | -0.255511499 | 0.700912881 | alkylbase DNA N-glycosylase |
| CNXL_023100 | CND00610 | 0.867747908 | 0.003155359 | carboxypeptidase D |
| CNXL_023110 | CND00620 | 0.259397844 | 0.61372863 | YjeF family protein |
| CNXL_023120 | CND00630 | -0.335875009 | 0.632998683 | glutathione transferase |
| CNXL_023130 | CND00640 | 0.36392517 | 0.516492158 | hypothetical protein |
| CNXL_023140 | CND00650 | -3.335902768 | 6.89405E-52 | membrane protein |
| CNXL_023150 | CND00660 | 0.749831645 | 0.016626382 | hypothetical protein |
| CNXL_023160 | CND00670 | 0.352057818 | 0.421670869 | alpha-1 |
| CNXL_023170 | CND00680 | -0.277142061 | 0.572463279 | guanyl nucleotide binding protein |
| CNXL_023180 | CND00690 | -0.202033388 | 0.690266807 | peptidase |

| | | | | |
|---|---|---|---|---|
| CNXL_023190 | CND00700 | -0.522958395 | 0.170587304 | hypothetical protein |
| CNXL_023200 | CND00710 | 0.126797085 | 0.855051728 | argininosuccinate synthase |
| CNXL_023210 | CND00720 | 0.703388134 | 0.012902916 | protein transporter |
| CNXL_023220 | CND00730 | 0.631493779 | 0.071408618 | hypothetical protein |
| CNXL_023230 | CND00740 | -0.514270389 | 0.253992342 | hypothetical protein |
| CNXL_023240 | CND00750 | -0.039868391 | 0.960513489 | allantoinase |
| CNXL_023250 | CND00760 | -0.382473999 | 0.488394981 | DNA-binding protein |
| CNXL_023260 | CND00770 | -0.512628353 | 0.231393839 | lipid particle protein |
| CNXL_023270 | CND00780 | -0.353383927 | 0.53679353 | hypothetical protein |
| CNXL_023280 | CND00790 | -0.895233047 | 0.009708037 | hypothetical protein |
| CNXL_023290 | CND00800 | -0.18550299 | 0.71725693 | glucan 1 |
| CNXL_023300 | CND00810 | 0.465282827 | 0.529627838 | exosome complex exonuclease |
| CNXL_023310 | CND00820 | 0 | NA | Unknown |
| CNXL_023320 | | NA | NA | Unknown |
| CNXL_023330 | CNN02020 | 2.52745236 | NA | Unknown |
| CNXL_023340 | | NA | NA | Unknown |
| CNXL_023350 | | -0.171923633 | 0.784304932 | Unknown |
| CNXL_023360 | | 1.271876086 | 2.4441E-07 | Unknown |
| CNXL_023370 | | 2.441343764 | NA | Unknown |
| CNXL_023380 | | NA | NA | Unknown |
| CNXL_023390 | CND00870 | 1.484927388 | NA | Unknown |
| CNXL_023400 | CND00900 | 0.369346134 | 0.316654495 | Unknown |
| CNXL_023410 | ND00910\|CND009 | 1.322357848 | 3.11232E-05 | ATP-dependent RNA helicase DHX8/PRP22 |
| CNXL_023420 | | -0.300416822 | 0.796875929 | Unknown |
| CNXL_023430 | CND00940 | 0.175526295 | 0.717376522 | hypothetical protein |
| CNXL_023440 | CND00950 | 0.199291116 | 0.679691843 | transketolase |
| CNXL_023450 | CND00960 | 0.590405014 | 0.08989414 | aromatic-L-amino-acid decarboxylase |
| CNXL_023460 | CND00970 | 0.65090838 | 0.183277443 | hypothetical protein |
| CNXL_023470 | CND00980 | 0.247485223 | 0.740314378 | hypothetical protein |
| CNXL_023480 | CND00990 | 0.339063827 | 0.406574476 | hypothetical protein |
| CNXL_023490 | CND01000 | -0.113851753 | 0.848021859 | phosphatidate cytidylyltransferase |
| CNXL_023500 | CND01010 | 0.403834967 | 0.237021921 | vacuolar fusion protein MON1 |
| CNXL_023510 | CND01020 | -1.344704939 | 0.017719875 | hypothetical protein |
| CNXL_023520 | CND01030 | -0.795011197 | 0.134439284 | hypothetical protein |
| CNXL_023530 | CND01040 | -0.427360683 | 0.510006134 | beta-lactamase |
| CNXL_023540 | CND01050 | -0.227544891 | 0.62092322 | hypothetical protein |
| CNXL_023550 | CND01060 | -0.430305806 | 0.249559817 | carbamoyl-phosphate synthase |
| CNXL_023560 | CND01070 | -0.073593182 | 0.91248949 | VHS domain-containing protein |
| CNXL_023570 | CND01080 | 0.52236284 | 0.105214931 | NADH dehydrogenase |
| CNXL_023580 | CND01090 | 1.474996471 | 6.47706E-07 | copper uptake transporter |
| CNXL_023590 | CND01100 | 0.237613623 | 0.63756045 | hypothetical protein |
| CNXL_023600 | CND01110 | -0.267152522 | 0.651490615 | hypothetical protein |
| CNXL_023610 | CND01120 | 2.147681121 | 0.068615652 | hypothetical protein |
| CNXL_023620 | CND01130 | 0.301293496 | 0.550631543 | glucose and ribitol dehydrogenase |
| CNXL_023630 | CND01140 | 0.00292815 | 0.996472611 | hypothetical protein |
| CNXL_023640 | CND01150 | 0.186368155 | 0.734939207 | urm1 activating enzyme |
| CNXL_023650 | CND01160 | -0.433692487 | 0.260424002 | prefoldin subunit |
| CNXL_023660 | CND01170 | -0.27066528 | 0.622869303 | importin subunit beta-1 |
| CNXL_023670 | CND01180 | 0.025049296 | 0.970515325 | ubiquitin carboxyl-terminal hydrolase |
| CNXL_023680 | CND01190 | 0.137493006 | 0.814220979 | F-type H+-transporting ATPase subunit H |
| CNXL_023690 | CND01200 | 0.042407135 | 0.944879244 | flap endonuclease 1 |
| CNXL_023700 | CND01210 | 0.110145151 | 0.855058004 | homocitrate synthase |
| CNXL_023710 | CND01220 | -0.079298038 | 0.902148461 | ubiquinone biosynthesis monooxygenase Coq7 |
| CNXL_023720 | CND01230 | 1.341622107 | 0.163523664 | protein mago nashi |
| CNXL_023730 | CND01240 | 0.022455386 | 0.978794024 | hypothetical protein |
| CNXL_023740 | CND01250 | -0.118160518 | 0.879531297 | O-mannosyltransferase |
| CNXL_023750 | CND01260 | -0.132881376 | 0.865068963 | hypothetical protein |
| CNXL_023760 | CND01270 | 0.448733973 | 0.25218212 | hypothetical protein |

| | | | | |
|---|---|---|---|---|
| CNXL_023770 | CND01280 | -0.441887092 | 0.315028758 | hypothetical protein |
| CNXL_023780 | CND01290 | 0.069889527 | 0.947835086 | nuclear pore complex protein Nup155 |
| CNXL_023790 | CND01310 | 0.808730787 | 0.003553157 | hypothetical protein |
| CNXL_023800 | CND01320 | -0.353733052 | 0.384367526 | hypothetical protein |
| CNXL_023810 | CND01330 | -2.564470788 | 4.75759E-27 | electron transporter |
| CNXL_023820 | CND01340 | 0.129565502 | 0.802877744 | hypothetical protein |
| CNXL_023830 | CND01350 | 0.266832957 | 0.602187556 | glutaredoxin |
| CNXL_023840 | CND01360 | 1.036684859 | 0.000838723 | hypothetical protein |
| CNXL_023850 | CND01370 | 0.407850813 | 0.392734784 | C2 domain-containing protein |
| CNXL_023860 | CND01380 | -0.788203333 | 0.004661011 | hypothetical protein |
| CNXL_023870 | CND01400 | 0.520603198 | 0.136259099 | hypothetical protein |
| CNXL_023880 | | 0.185400455 | 0.78519633 | hypothetical protein |
| CNXL_023890 | CND01430 | -0.202924724 | 0.765840893 | hypothetical protein |
| CNXL_023900 | CND01440 | -0.135723989 | 0.786945286 | chromatin binding protein |
| CNXL_023910 | CND01450 | -0.405908786 | 0.527852637 | hypothetical protein |
| CNXL_023920 | CND01460 | -0.141861072 | 0.833643586 | cytoplasmic protein |
| CNXL_023930 | CND01470 | -0.033938212 | 0.969527869 | vacuolar membrane protein |
| CNXL_023940 | CND01480 | -0.059674467 | 0.921544337 | E3 ubiquitin-protein ligase SHPRH |
| CNXL_023950 | CND01490 | 0.006726596 | 0.992176872 | histone-lysine N-methyltransferase Su |
| CNXL_023960 | CND01500 | -0.915422151 | 0.003480956 | copper/zinc superoxide dismutase |
| CNXL_023970 | CND01510 | -0.348970179 | 0.494360023 | hypothetical protein |
| CNXL_023980 | CND01520 | 0.657136626 | 0.08995737 | ATP phosphoribosyltransferase |
| CNXL_023990 | CND01530 | 0.089016419 | 0.885370637 | hypothetical protein |
| CNXL_024000 | CND01540 | -0.687998517 | 0.118180237 | cohesin complex subunit SCC1 |
| CNXL_024010 | CND01550 | 0.044244154 | 0.957098418 | translocation protein SEC72 |
| CNXL_024020 | CND01560 | -0.494591421 | 0.256216847 | hypothetical protein |
| CNXL_024030 | CND01570 | 0.443397313 | 0.299696006 | gamma-glutamyltransferase |
| CNXL_024040 | CND01580 | 0.057575901 | 0.944879244 | succinate-semialdehyde dehydrogenase |
| CNXL_024050 | CND01590 | 0.907964935 | 0.002295505 | hypothetical protein |
| CNXL_024060 | CND01600 | 0.24746213 | 0.768164316 | impact family protein |
| CNXL_024070 | CND01620 | 1.208735743 | 0.000828366 | hypothetical protein |
| CNXL_024080 | CND01630 | 0.036753295 | 0.964414864 | hypothetical protein |
| CNXL_024090 | CND01650 | 0.054520323 | 0.948286148 | hypothetical protein |
| CNXL_024100 | CND01660 | 0.018843541 | 0.982102702 | exosome complex component CSL4 |
| CNXL_024110 | CND01670 | 0.389498183 | 0.377185962 | 20S proteasome subunit alpha 4 |
| CNXL_024120 | CND01680 | 0.340545385 | 0.417866535 | geranylgeranyl transferase type-2 subunit beta |
| CNXL_024130 | CND01690 | -0.085246908 | 0.916084262 | DNA mismatch repair protein MLH3 |
| CNXL_024140 | CND01700 | -0.223596314 | 0.633393243 | hypothetical protein |
| CNXL_024150 | CND01720 | -0.557149231 | 0.179438445 | superkiller protein 3 |
| CNXL_024160 | CND01730 | 0.940603892 | 0.003971452 | carboxypeptidase D |
| CNXL_024170 | CND01740 | 0.413667238 | 0.320327175 | hypothetical protein |
| CNXL_024180 | CND01750 | 0.139201043 | 0.794331499 | dihydroceramidase |
| CNXL_024190 | CND01760 | 0.628363591 | 0.039954962 | hypothetical protein |
| CNXL_024200 | CND01770 | 1.3779665 | 3.44376E-05 | N |
| CNXL_024210 | | -0.181983961 | 0.717347987 | hypothetical protein |
| CNXL_024220 | CND01790 | -0.261186247 | 0.554514544 | hypothetical protein |
| CNXL_024230 | CND01800 | -0.012090197 | 0.990008547 | H/ACA ribonucleoprotein complex subunit 3 |
| CNXL_024240 | CND01810 | -0.07160608 | 0.911130835 | hypothetical protein |
| CNXL_024250 | CND01820 | 1.195340547 | 0.29684976 | dynactin 1 |
| CNXL_024260 | CND01830 | -0.187231124 | 0.891467418 | hypothetical protein |
| CNXL_024270 | CND01840 | 0.00165197 | 0.997135738 | Unknown |
| CNXL_024280 | | -0.045699812 | 0.966962785 | nuclear pore complex protein Nup160 |
| CNXL_024290 | CND01860 | 0.345175026 | 0.365237105 | hypothetical protein |
| CNXL_024300 | | 0.493353817 | 0.176771646 | phospholipid transporter |
| CNXL_024310 | CND01880 | 0.240005703 | 0.603329126 | hypothetical protein |
| CNXL_024320 | CND01890 | 0.133612831 | 0.81842519 | COP9 signalosome complex subunit 2 |
| CNXL_024330 | CND01900 | -0.194340927 | 0.705198732 | nitric oxide synthase-interacting protein |
| CNXL_024340 | CND01910 | 0.305137866 | 0.46380108 | vacuolar membrane protein |

| | | | | |
|---|---|---|---|---|
| CNXL_024350 | CND01920 | 0.489167954 | 0.157787273 | hypothetical protein |
| CNXL_024360 | CND01930 | -0.400743569 | 0.308102113 | serine/threonine protein kinase |
| CNXL_024370 | CND01940 | -0.076953726 | 0.905178976 | CAMK/CAMKL/PASK protein kinase |
| CNXL_024380 | CND01950 | 0.771246937 | 0.006907933 | WD-repeat protein mip1 |
| CNXL_024390 | CND01960 | 0.355167572 | 0.467252392 | hypothetical protein |
| CNXL_024400 | CND01980 | -1.217575967 | 4.92891E-06 | hypothetical protein |
| CNXL_024410 | CND01990 | 0.01408162 | 0.986620478 | hypothetical protein |
| CNXL_024420 | CND02000 | 0.343417909 | 0.417866535 | hypothetical protein |
| CNXL_024430 | CND02010 | -0.166638545 | 0.789040329 | hypothetical protein |
| CNXL_024440 | CND02020 | -0.06497217 | 0.934677332 | hypothetical protein |
| CNXL_024450 | CND02030 | -0.014622572 | 0.987967132 | neutral amino acid transporter |
| CNXL_024460 | CND02040 | 0.895645254 | 0.060144397 | methylmalonate-semialdehyde dehydrogenase |
| CNXL_024470 | CND02050 | -1.533518339 | 0.033808576 | 4-aminobutyrate aminotransferase |
| CNXL_024480 | CND02060 | -0.092769896 | 0.911130835 | hypothetical protein |
| CNXL_024490 | CND02070 | 0.177326295 | 0.889001755 | aldehyde dehydrogenase |
| CNXL_024500 | CND02080 | -1.133360968 | 0.529627838 | 2 |
| CNXL_024510 | CND02090 | 0.201938515 | 0.823403666 | glycolate oxidase |
| CNXL_024520 | CND02100 | -0.020965502 | 0.982102702 | hypothetical protein |
| CNXL_024530 | CND02110 | 0.29295552 | 0.49418561 | hypothetical protein |
| CNXL_024540 | CND02120 | -0.341552541 | 0.489809679 | 26s proteasome regulatory subunit |
| CNXL_024550 | | 0.009865628 | 0.992370477 | ubiquitin conjugating enzyme |
| CNXL_024560 | CND02130 | 0.188961924 | 0.779355929 | hypothetical protein |
| CNXL_024570 | CND02140 | 0.282827003 | 0.452146814 | hypothetical protein |
| CNXL_024580 | CND02150 | 0.040400202 | 0.965101267 | hypothetical protein |
| CNXL_024590 | CND02160 | -0.109795849 | 0.861693974 | hypothetical protein |
| CNXL_024600 | CND02170 | -0.015980838 | 0.985173026 | hypothetical protein |
| CNXL_024610 | CND02180 | -0.184489173 | 0.737538678 | U5 small nuclear ribonucleoprotein component |
| CNXL_024620 | CND02190 | -0.142796753 | 0.810102246 | hypothetical protein |
| CNXL_024630 | CND02200 | 0.134393701 | 0.803947851 | hypothetical protein |
| CNXL_024640 | CND02210 | 0.243814085 | 0.646500974 | pre-mRNA-splicing factor CWC25 |
| CNXL_024650 | CND02220 | -0.032669654 | 0.972727243 | BEM46 family protein |
| CNXL_024660 | ND02230\|CND022 | 0.324282444 | 0.366302025 | hypothetical protein |
| CNXL_024670 | CND02250 | -0.225373245 | 0.633915651 | Unknown |
| CNXL_024680 | CND02260 | -0.622501879 | 0.055459171 | hypothetical protein |
| CNXL_024690 | CND02270 | -0.187093295 | 0.746237137 | hypothetical protein |
| CNXL_024700 | CND02280 | 1.843120852 | 0.038107022 | hypothetical protein |
| CNXL_024710 | CND02290 | 0.005828674 | 0.994610173 | oxidoreductase |
| CNXL_024720 | CND02300 | -0.063702487 | 0.934072032 | splicing factor 3A subunit 2 |
| CNXL_024730 | CND02310 | -1.713567892 | 1.3855E-05 | ribosomal RNA-processing protein 8 |
| CNXL_024740 | CND02320 | -0.039386896 | 0.954207791 | hypothetical protein |
| CNXL_024750 | CND02330 | -0.014378402 | 0.987160302 | vacuolar-ATPase subunit |
| CNXL_024760 | CND02340 | -0.390245394 | 0.414496592 | phosphatidylinositol glycan |
| CNXL_024770 | CND02350 | -0.07576988 | 0.907893004 | allantoicase |
| CNXL_024780 | CND02360 | 0.398335835 | 0.286857977 | hypothetical protein |
| CNXL_024790 | CND02370 | -0.223739973 | 0.615520877 | co-chaperone |
| CNXL_024800 | CND02380 | 0.919579286 | 0.014417471 | translation initiation factor 3 subunit K |
| CNXL_024810 | | 0.021143619 | 0.982795272 | xenobiotic reductase |
| CNXL_024820 | CND02390 | -0.011887472 | 0.990703091 | hypothetical protein |
| CNXL_024830 | CND02400 | 0.256956232 | 0.628160086 | hypothetical protein |
| CNXL_024840 | | 0.372516373 | 0.450895285 | hypothetical protein |
| CNXL_024850 | CND02410 | -0.115208663 | 0.839374462 | Unknown |
| CNXL_024860 | CND02420 | -0.004006914 | 0.995928861 | beta-ketoacyl reductase |
| CNXL_024870 | CND02430 | 0.643169796 | 0.027448551 | elongation factor 3 |
| CNXL_024880 | CND02440 | 0.40002429 | 0.288029878 | AAT family amino acid transporter |
| CNXL_024890 | CND02450 | -0.238033876 | 0.578320964 | hypothetical protein |
| CNXL_024900 | CND02460 | -1.930813088 | 7.01363E-16 | hypothetical protein |
| CNXL_024910 | | -1.385510592 | 8.97273E-05 | hypothetical protein |
| CNXL_024920 | CND02470 | 0.323175005 | 0.544657787 | hypothetical protein |

| | | | | |
|---|---|---|---|---|
| CNXL_024930 | | -0.101875788 | 0.91272593 | DRAP deaminase |
| CNXL_024940 | CND02480 | 0.2977497 | 0.427104346 | hypothetical protein |
| CNXL_024950 | CND02490 | -0.39112609 | 0.353576993 | hypothetical protein |
| CNXL_024960 | CND02500 | 0.109781652 | 0.868398587 | hypothetical protein |
| CNXL_024970 | CND02510 | 0.740480805 | 0.031096848 | hypothetical protein |
| CNXL_024980 | CND02520 | -0.057661013 | 0.931289001 | hydroxyacylglutathione hydrolase |
| CNXL_024990 | CND02530 | -0.339528539 | 0.531504526 | lanosterol synthase |
| CNXL_025000 | CND02540 | -2.040861717 | 1.09077E-15 | hypothetical protein |
| CNXL_025010 | CND02550 | -0.18022805 | 0.773509113 | hypothetical protein |
| CNXL_025020 | CND02560 | 0.16916723 | 0.837479784 | nucleolar protein 6 |
| CNXL_025030 | CND02570 | -1.205449809 | 0.022739274 | Unknown |
| CNXL_025040 | CND02580 | -0.035090699 | 0.9653396 | mitochondrial protein |
| CNXL_025050 | CND02590 | -0.514346278 | 0.425879761 | hypothetical protein |
| CNXL_025060 | CND02600 | -0.289245689 | 0.638320737 | nuclear distribution protein PAC1 |
| CNXL_025070 | CND02610 | -0.453040624 | 0.35484081 | delta |
| CNXL_025080 | CND02620 | 0.215170827 | 0.592954724 | hypothetical protein |
| CNXL_025090 | CND02630 | 0.263199105 | 0.535369833 | aconitate hydratase |
| CNXL_025100 | | 0.821971067 | 0.038240381 | putative mitochondrial cytochrome c peroxidase |
| CNXL_025110 | CND02650 | -0.25815103 | 0.604085424 | Unknown |
| CNXL_025120 | CND02660 | 0.070026071 | 0.920669589 | ATP-dependent RNA helicase DRS1 |
| CNXL_025130 | CND02670 | 0.291885312 | 0.527852637 | exosome complex component RRP40 |
| CNXL_025140 | CND02680 | -0.165592911 | 0.789040329 | endoplasmic reticulum protein |
| CNXL_025150 | CND02690 | -0.520096614 | 0.160833876 | hypothetical protein |
| CNXL_025160 | CND02700 | -0.680774497 | 0.353760275 | replication protein |
| CNXL_025170 | CND02710 | -0.688419111 | 0.048602556 | hypothetical protein |
| CNXL_025180 | CND02720 | 0.496961847 | 0.18112051 | U3 small nucleolar RNA-associated protein 22 |
| CNXL_025190 | CND02730 | -0.065077668 | 0.918701468 | ARP2/3 complex 20 kDa subunit |
| CNXL_025200 | CND02740 | -1.409948034 | 1.77978E-07 | peptidyl-prolyl cis-trans isomerase |
| CNXL_025210 | CND02750 | 0.465282155 | 0.114264467 | hypothetical protein |
| CNXL_025220 | CND02770 | -0.006644891 | 0.992209833 | omega-6 fatty acid desaturase |
| CNXL_025230 | CND02780 | -0.173486316 | 0.7126331 | ubiquitin-conjugating enzyme E2 D/E |
| CNXL_025240 | CND02790 | -0.185355538 | 0.712119576 | small subunit ribosomal protein S6e |
| CNXL_025250 | | 0.172242438 | 0.822421189 | small subunit ribosomal protein S13e |
| CNXL_025260 | CND02800 | 0.765779918 | 0.003530596 | hypothetical protein |
| CNXL_025270 | CND02810 | 0.63736035 | 0.1057341 | glycerol kinase |
| CNXL_025280 | CND02820 | -0.124492299 | 0.833643586 | capsular related protein |
| CNXL_025290 | CND02830 | 0.379330867 | 0.270697928 | hypothetical protein |
| CNXL_025300 | CND02840 | 0.778100479 | 0.008740283 | hypothetical protein |
| CNXL_025310 | CND02860 | -0.216391975 | 0.637983721 | hypothetical protein |
| CNXL_025320 | CND02870 | 0.176952649 | 0.710014173 | U3 small nucleolar RNA-associated protein 21 |
| CNXL_025330 | CND02880 | -0.301667346 | 0.473663995 | pre-mRNA-splicing factor SLU7 |
| CNXL_025340 | CND02890 | -0.986972953 | 0.010542889 | branchpoint-bridging protein |
| CNXL_025350 | CND02900 | 0.388554299 | 0.311813532 | DNA repair and recombination protein |
| CNXL_025360 | CND02910 | -0.066068735 | 0.935017691 | glutamine-fructose-6-phosphate transaminase |
| CNXL_025370 | CND02920 | 0.171473084 | 0.748609863 | D-erythro-sphingosine kinase |
| CNXL_025380 | CND02930 | -0.078136896 | 0.902044607 | urease accessory protein |
| CNXL_025390 | CND02940 | -0.054933643 | 0.926407741 | protein of unknown function |
| CNXL_025400 | CND02950 | -0.373735023 | 0.487397795 | H/ACA ribonucleoprotein complex subunit 4 |
| CNXL_025410 | CND02960 | -0.149580742 | 0.766858347 | aspartyl aminopeptidase |
| CNXL_025420 | CND02970 | -0.308987774 | 0.539585872 | small subunit ribosomal protein S17 |
| CNXL_025430 | CND02980 | 0.15101682 | 0.798267462 | hypothetical protein |
| CNXL_025440 | CND02990 | -0.770806001 | 0.004816702 | parallel beta-helix repeat protein |
| CNXL_025450 | CND03010 | 0.210861023 | 0.662908358 | Lag1 family transcription factor |
| CNXL_025460 | | -0.197225457 | 0.739013485 | hypothetical protein |
| CNXL_025470 | CND03020 | 0.161837881 | 0.810102246 | 3-keto sterol reductase |
| CNXL_025480 | | -0.358123739 | 0.523679914 | 3-keto sterol reductase |
| CNXL_025490 | CND03030 | -0.550220409 | 0.389877867 | hypothetical protein |
| CNXL_025500 | CND03040 | -0.036247386 | 0.964076726 | hypothetical protein |

| | | | | |
|---|---|---|---|---|
| CNXL_025510 | CND03050 | -0.403546464 | 0.39417374 | hypothetical protein |
| CNXL_025520 | CND03060 | -0.029049953 | 0.965101267 | hypothetical protein |
| CNXL_025530 | CND03070 | -0.006351416 | 0.992176872 | small subunit ribosomal protein S27Ae |
| CNXL_025540 | CND03080 | 0.051200415 | 0.948286148 | cytoplasmic protein |
| CNXL_025550 | CND03090 | 0.650161641 | 0.039924281 | bar-SH3 domain protein |
| CNXL_025560 | CND03100 | -0.615131598 | 0.060383523 | hypothetical protein |
| CNXL_025570 | CND03110 | -0.216806303 | 0.665556981 | hsp70-like protein |
| CNXL_025580 | CND03120 | -0.043015894 | 0.95265881 | hypothetical protein |
| CNXL_025590 | CND03130 | -0.614183989 | 0.071240987 | ribosome production factor 2 |
| CNXL_025600 | CND03140 | -0.533772327 | 0.129163389 | peroxin-19 |
| CNXL_025610 | CND03150 | 0.076563718 | 0.902044607 | DNA-directed RNA polymerase I subunit RPA1 |
| CNXL_025620 | CND03160 | 0.16069498 | 0.765354566 | vesicle transporter SEC22 |
| CNXL_025630 | | 0.178349213 | 0.771763303 | long-chain fatty acid transporter |
| CNXL_025640 | | -1.408609583 | 0.047255368 | hypothetical protein |
| CNXL_025650 | CND03170 | -1.064848663 | 0.000140314 | Unknown |
| CNXL_025660 | CND03190 | 0.029940141 | 0.969179648 | hypothetical protein |
| CNXL_025670 | CND03200 | 0.329538965 | 0.443697582 | cation-transporting ATPase 13A3/4/5 |
| CNXL_025680 | CND03210 | -0.492451491 | 0.188739467 | tyrosyl-DNA phosphodiesterase 1 |
| CNXL_025690 | CND03220 | -0.137766319 | 0.813525402 | ran GTPase-activating protein 1 |
| CNXL_025700 | CND03230 | -0.547222339 | 0.259411381 | small nuclear ribonucleoprotein F |
| CNXL_025710 | CND03240 | 0.430370375 | 0.338239663 | nuclear pore complex protein Nup62 |
| CNXL_025720 | CND03250 | 0.00096606 | 0.998007755 | pre-mRNA-splicing factor 38A |
| CNXL_025730 | CND03260 | -0.118397882 | 0.890150525 | hypothetical protein |
| CNXL_025740 | CND03270 | 0.139316824 | 0.813710183 | hypothetical protein |
| CNXL_025750 | CND03280 | -0.706865006 | 0.033430524 | dual specificity phosphatase 12 |
| CNXL_025760 | CND03290 | -0.422199108 | 0.38906085 | ATP synthase F1 |
| CNXL_025770 | CND03300 | 0.611461039 | 0.089107701 | CCAAT-binding transcription factor |
| CNXL_025780 | CND03310 | 0.14682064 | 0.770005211 | HAD hydrolase |
| CNXL_025790 | CND03320 | -0.317923403 | 0.485492767 | hypothetical protein |
| CNXL_025800 | CND03330 | -0.015919528 | 0.985173026 | Ca |
| CNXL_025810 | | 0.996184156 | 0.000802569 | 1-phosphatidylinositol-3-phosphate 5-kinase |
| CNXL_025820 | CND03340 | 0.069538124 | 0.919196787 | hypothetical protein |
| CNXL_025830 | CND03360 | 0.942694316 | 0.002832908 | hypothetical protein |
| CNXL_025840 | CND03370 | 0.5320974 | 0.107144034 | hypothetical protein |
| CNXL_025850 | CND03380 | -0.224736044 | 0.75185116 | hypothetical protein |
| CNXL_025860 | | -0.11675045 | 0.840322617 | alpha 1 |
| CNXL_025870 | CND03390 | -0.003662795 | 0.996158838 | hypothetical protein |
| CNXL_025880 | CND03400 | -0.228859127 | 0.626496712 | 6-phosphogluconolactonase |
| CNXL_025890 | | 0.987760606 | 0.000106528 | protein MPE1 |
| CNXL_025900 | CND03420 | 0.109813065 | 0.881830184 | hypothetical protein |
| CNXL_025910 | CND03430 | 0.096477702 | 0.883172612 | mitochondrial FAD-linked sulfhydryl oxidase ERV1 |
| CNXL_025920 | CND03440 | -0.071635903 | 0.914458963 | di-trans |
| CNXL_025930 | CND03450 | -2.881026877 | 9.55078E-39 | hypothetical protein |
| CNXL_025940 | CND03460 | -0.147037609 | 0.771324374 | hypothetical protein |
| CNXL_025950 | CND03470 | -0.352764591 | 0.662151848 | large subunit ribosomal protein L18-A |
| CNXL_025960 | | -0.475248112 | 0.315823773 | hypothetical protein |
| CNXL_025970 | CND03480 | -0.045250489 | 0.964414864 | hypothetical protein |
| CNXL_025980 | CND03490 | -0.127879251 | 0.830440343 | L-mandelate dehydrogenase |
| CNXL_025990 | CND03500 | -0.137921641 | 0.806543943 | chitin-deacetylase |
| CNXL_026000 | CND03510 | -0.443990577 | 0.269459866 | agmatinase |
| CNXL_026010 | CND03520 | -0.820376627 | 0.004250969 | putative calcium-transporting ATPase |
| CNXL_026020 | CND03530 | 0.793307245 | 0.022422038 | hypothetical protein |
| CNXL_026030 | CND03540 | -0.122701658 | 0.808723333 | spore wall assembly-associated protein |
| CNXL_026040 | CND03550 | -0.088763376 | 0.885831771 | DNA-directed RNA polymerase II subunit RPB2 |
| CNXL_026050 | | 0.446990215 | 0.271524617 | rRNA-processing protein EBP2 |
| CNXL_026060 | CND03570 | 0.218209115 | 0.650597819 | hypothetical protein |
| CNXL_026070 | CND03580 | 0.111222175 | 0.81842519 | arginine biosynthesis ArgJ |
| CNXL_026080 | CND03590 | 0.213405452 | 0.63888365 | chitin deacetylase |

| | | | | |
|---|---|---|---|---|
| CNXL_026090 | CND03600 | -0.328827001 | 0.492041786 | enzyme regulator |
| CNXL_026100 | CND03610 | -0.712159758 | 0.095121736 | CCAAT -binding transcription factor |
| CNXL_026110 | CND03620 | 0.566679655 | 0.099263361 | histone-lysine N-methyltransferase |
| CNXL_026120 | CND03630 | -0.377233228 | 0.311813532 | hypothetical protein |
| CNXL_026130 | CND03640 | 0.178049617 | 0.770559628 | hypothetical protein |
| CNXL_026140 | | 0.081948197 | 0.902229878 | hypothetical protein |
| CNXL_026150 | CND03650 | 0.238406273 | 0.615328522 | hypothetical protein |
| CNXL_026160 | CND03660 | -0.375254512 | 0.445982809 | vacuole morphology and inheritance protein 14 |
| CNXL_026170 | CND03670 | -0.276409043 | 0.623549135 | hypothetical protein |
| CNXL_026180 | CND03680 | -0.163725839 | 0.733041323 | tRNA ligase |
| CNXL_026190 | CND03690 | -0.362334532 | 0.334301792 | e3 ubiquitin-protein ligase |
| CNXL_026200 | CND03700 | 0.062364805 | 0.920254084 | thiosulfate/3-mercaptopyruvate sulfurtransferase |
| CNXL_026210 | CND03710 | -0.305501389 | 0.643890318 | hypothetical protein |
| CNXL_026220 | CND03720 | -1.875738116 | 1.13787E-15 | protein-tyrosine-phosphatase |
| CNXL_026230 | CND03730 | -0.225931885 | 0.626170102 | hypothetical protein |
| CNXL_026240 | CND03740 | 0.218907499 | 0.615328522 | hypothetical protein |
| CNXL_026250 | CND03750 | -0.659831134 | 0.063702758 | aldo-keto reductase |
| CNXL_026260 | CND03760 | -0.03017457 | 0.968722319 | hypothetical protein |
| CNXL_026270 | CND03770 | 0.183642611 | 0.664461114 | MATE family multidrug resistance protein |
| CNXL_026280 | CNE03210 | 1.182745331 | NA | hypothetical protein |
| CNXL_026290 | | NA | NA | Unknown |
| CNXL_026300 | CND03780 | 0.092314906 | 0.887770615 | Unknown |
| CNXL_026310 | CND03790 | -0.062914261 | 0.932840267 | essential nuclear protein 1 |
| CNXL_026320 | CND03800 | -0.088882939 | 0.881752079 | hypothetical protein |
| CNXL_026330 | CND03810 | -0.25427365 | 0.675638659 | hypothetical protein |
| CNXL_026340 | CND03820 | 0.24089957 | 0.595850448 | transcription factor binding protein |
| CNXL_026350 | CND03830 | -1.321975458 | 5.53674E-07 | myosin I binding protein |
| CNXL_026360 | CND03840 | 0.188279028 | 0.717347987 | G-protein beta subunit |
| CNXL_026370 | CND03850 | -0.231044706 | 0.616956521 | vacuolar transporter chaperone 4 |
| CNXL_026380 | | 0.525307416 | 0.176678435 | homoisocitrate dehydrogenase |
| CNXL_026390 | CND03860 | -0.12404078 | 0.861760083 | charged multivesicular body protein 6 |
| CNXL_026400 | CND03880 | -0.103660113 | 0.893955723 | ATP-dependent protease La |
| CNXL_026410 | CND03890 | 0.174310772 | 0.732619483 | hypothetical protein |
| CNXL_026420 | CND03900 | -0.04453632 | 0.949084374 | asparagine-tRNA ligase |
| CNXL_026430 | CND03910 | 0.147528145 | 0.782173947 | hypothetical protein |
| CNXL_026440 | CND03920 | -1.254333423 | 0.000168645 | pre-mRNA-splicing factor ISY1 |
| CNXL_026450 | CND03930 | 0.114664327 | 0.827849849 | hypothetical protein |
| CNXL_026460 | CND03940 | 0.009655675 | 0.990777629 | cellular nucleic acid-binding protein |
| CNXL_026470 | CND03950 | 0.01055684 | 0.990008547 | coatomer subunit gamma |
| CNXL_026480 | CND03960 | 0.000177004 | 0.999474019 | hypothetical protein |
| CNXL_026490 | CND03970 | -1.277101009 | 4.27275E-07 | hypothetical protein |
| CNXL_026500 | CND03980 | -0.573318237 | 0.10562276 | hypothetical protein |
| CNXL_026510 | CND04010 | 0.067662724 | 0.920669589 | aminophospholipid translocase |
| CNXL_026520 | | 0.450432466 | 0.30897417 | ubiquitin-conjugating enzyme E2 S |
| CNXL_026530 | CND04020 | 0.181993236 | 0.699872164 | Unknown |
| CNXL_026540 | CND04030 | -0.163107352 | 0.746347427 | hypothetical protein |
| CNXL_026550 | CND04040 | 0.096179572 | 0.879531297 | beta-1 |
| CNXL_026560 | CND04050 | -0.319951366 | 0.687195736 | ATPase |
| CNXL_026570 | CND04060 | 0.375201786 | 0.376662948 | Aur protein kinase |
| CNXL_026580 | CND04070 | -0.036920645 | 0.955507961 | hypothetical protein |
| CNXL_026590 | CND04080 | -0.174518868 | 0.802549726 | NADH-ubiquinone oxidoreductase 51 kDa subunit |
| CNXL_026600 | CND04090 | 0.37350315 | 0.361761993 | solute carrier family 25 |
| CNXL_026610 | CND04100 | 0.020340363 | 0.985595171 | hypothetical protein |
| CNXL_026620 | CND04110 | -1.294904908 | 0.000104772 | hypothetical protein |
| CNXL_026630 | CND04120 | -0.281956889 | 0.664165041 | hypothetical protein |
| CNXL_026640 | CND04130 | -0.361002953 | 0.414496592 | pol II transcription elongation factor |
| CNXL_026650 | CND04140 | 0.373995767 | 0.372306093 | hypothetical protein |
| CNXL_026660 | CND04150 | 0.653097778 | 0.039924281 | inositol-pentakisphosphate 2-kinase |

| | | | | |
|---|---|---|---|---|
| CNXL_026670 | CND04160 | -0.320767853 | 0.534266327 | acyl carrier protein |
| CNXL_026680 | | 0.162373416 | 0.761797019 | hypothetical protein |
| CNXL_026690 | CND04180 | -0.130715904 | 0.838048912 | hypothetical protein |
| CNXL_026700 | CND04190 | -1.873206466 | 2.73837E-13 | lysophospholipase NTE1 |
| CNXL_026710 | CND04200 | -0.137741673 | 0.796855977 | hypothetical protein |
| CNXL_026720 | CND04210 | 0.505420872 | 0.134036805 | small subunit ribosomal protein S21e |
| CNXL_026730 | CND04220 | 0.372037684 | 0.335274247 | hypothetical protein |
| CNXL_026740 | | -0.299702613 | 0.498081161 | hypothetical protein |
| CNXL_026750 | CND04240 | -0.321656232 | 0.495837743 | hypothetical protein |
| CNXL_026760 | CND04250 | -0.489088675 | 0.113559841 | polynucleotide 5'-hydroxyl-kinase GRC3 |
| CNXL_026770 | CND04260 | 0.392151117 | 0.334284541 | methionine-tRNA ligase |
| CNXL_026780 | CND04270 | 0.560487368 | 0.175295136 | chromodomain-helicase-DNA-binding protein 3 |
| CNXL_026790 | CND04280 | 0.143453916 | 0.81842519 | endoplasmic reticulum protein |
| CNXL_026800 | CND04290 | 0.013371226 | 0.988855811 | protein NRD1 |
| CNXL_026810 | CND04300 | 0.027882262 | 0.975380213 | arf/Sar family protein |
| CNXL_026820 | CND04310 | -0.076070807 | 0.893955723 | nucleolar protein |
| CNXL_026830 | CND04320 | -0.630919345 | 0.051496226 | casein kinase I |
| CNXL_026840 | CND04330 | 0.133357418 | 0.809176467 | proteasome maturation protein |
| CNXL_026850 | CND04340 | -0.048659434 | 0.937680326 | hypothetical protein |
| CNXL_026860 | CND04350 | -0.334925953 | 0.442107452 | vacuolar protein |
| CNXL_026870 | CND04360 | -0.553938187 | 0.264480588 | protein transporter |
| CNXL_026880 | CND04370 | 0.101398587 | 0.879713683 | replication protein |
| CNXL_026890 | CND04380 | -0.011020479 | 0.990008547 | hypothetical protein |
| CNXL_026900 | CND04390 | -0.090550506 | 0.886480005 | sodium/hydrogen exchanger 3 |
| CNXL_026910 | CND04400 | -0.078114706 | 0.911754274 | hypothetical protein |
| CNXL_026920 | CND04410 | 0.222516311 | 0.602461418 | large subunit ribosomal protein L17 |
| CNXL_026930 | CND04420 | -0.244359864 | 0.637983721 | acyl carrier protein |
| CNXL_026940 | CND04430 | -0.392090029 | 0.233441904 | hypothetical protein |
| CNXL_026950 | | -0.344616278 | 0.525549979 | ubiquinol-cytochrome c reductase subunit 7 |
| CNXL_026960 | CND04450 | -0.095310745 | 0.919196787 | hypothetical protein |
| CNXL_026970 | CND04460 | -0.272425692 | 0.58338982 | DASH complex subunit SPC19 |
| CNXL_026980 | CND04470 | 0.34830217 | 0.353691088 | hypothetical protein |
| CNXL_026990 | CND04480 | -0.077748474 | 0.893955723 | hypothetical protein |
| CNXL_027000 | CND04490 | -0.300435662 | 0.803882252 | small subunit ribosomal protein S24e |
| CNXL_027010 | CND04500 | -0.292363442 | 0.606485752 | Haspin protein kinase |
| CNXL_027020 | CND04510 | -1.070434727 | 0.007838513 | hypothetical protein |
| CNXL_027030 | | -0.242305486 | 0.65082026 | hypothetical protein |
| CNXL_027040 | CND04520 | 0.066446525 | 0.942099307 | hypothetical protein |
| CNXL_027050 | CND04530 | 0.419467901 | 0.245125122 | WD-repeat protein |
| CNXL_027060 | CND04540 | 0.204789689 | 0.666917003 | hypothetical protein |
| CNXL_027070 | CND04550 | -0.172413739 | 0.77424887 | cytoplasmic protein |
| CNXL_027080 | CND04560 | 1.175498031 | 0.000829225 | ATPase |
| CNXL_027090 | CND04570 | 0.203349839 | 0.746537956 | mannose-6-phosphate isomerase |
| CNXL_027100 | CND04580 | 0.195951186 | 0.746537956 | hypothetical protein |
| CNXL_027110 | CND04590 | 0.505850727 | 0.127361287 | hypothetical protein |
| CNXL_027120 | CND04600 | 0.055324721 | 0.931289001 | hypothetical protein |
| CNXL_027130 | CND04610 | 0.887487578 | 0.001498768 | small subunit ribosomal protein S2 |
| CNXL_027140 | CND04620 | -0.205669583 | 0.701325477 | hypothetical protein |
| CNXL_027150 | CND04630 | 2.129873978 | 0.01174828 | co-chaperone |
| CNXL_027160 | CND04640 | -1.164277946 | 0.000164778 | cyanate hydratase |
| CNXL_027170 | | -0.426848167 | 0.410672957 | hypothetical protein |
| CNXL_027180 | CND04660 | 0.233425871 | 0.586181879 | DNA-directed RNA polymerase I |
| CNXL_027190 | CND04670 | 0.319244414 | 0.440656673 | putative ubiquitin ligase |
| CNXL_027200 | CND04680 | -0.181852694 | 0.718553782 | hypothetical protein |
| CNXL_027210 | | 0.051277599 | 0.950413156 | hypothetical protein |
| CNXL_027220 | | -0.159060078 | 0.776125901 | hypothetical protein |
| CNXL_027230 | CND04690 | -0.165458679 | 0.78519633 | hypothetical protein |
| CNXL_027240 | CND04700 | -0.040981299 | 0.954207791 | hypothetical protein |

| | | | | |
|---|---|---|---|---|
| CNXL_027250 | CND04710 | 0.00832983 | 0.992176872 | thioredoxin |
| CNXL_027260 | CND04730 | -0.54723727 | 0.375781065 | hypothetical protein |
| CNXL_027270 | CND04740 | -0.234406175 | 0.779355929 | hypothetical protein |
| CNXL_027280 | CND04750 | -0.0047948 | 0.995428247 | hypothetical protein |
| CNXL_027290 | CND04760 | -0.578444741 | 0.17432549 | DNA-directed RNA polymerase II subunit RPB7 |
| CNXL_027300 | CND04770 | -0.137592809 | 0.784279547 | pumilio domain-containing protein c |
| CNXL_027310 | CND04780 | 0.290974804 | 0.492825703 | importin alpha subunit |
| CNXL_027320 | CND04790 | -0.246447071 | 0.608487348 | cell cycle control protein cwf19 |
| CNXL_027330 | CND04800 | -1.182614909 | 0.000937518 | XPA-binding protein 1 |
| CNXL_027340 | CND04810 | 0.151516633 | 0.809974479 | guanylate kinase |
| CNXL_027350 | CND04840 | -0.349839115 | 0.359326943 | hypothetical protein |
| CNXL_027360 | CND04850 | -0.203363356 | 0.767914053 | regulator of G protein signaling |
| CNXL_027370 | CND04860 | -1.491764955 | 1.49696E-09 | hypothetical protein |
| CNXL_027380 | CND04870 | -2.169685559 | 2.8185E-06 | septin ring protein |
| CNXL_027390 | CND04880 | 0.262379247 | 0.617084227 | delayed-type hypersensitivity antigen |
| CNXL_027400 | CND04890 | -0.452090344 | 0.238702905 | actin-binding protein homolog |
| CNXL_027410 | CND04900 | -0.16145726 | 0.773509113 | stress response protein NST1 |
| CNXL_027420 | CND04910 | -1.443422329 | 1.08467E-08 | pab-dependent poly |
| CNXL_027430 | CND04920 | -0.719364174 | 0.023480155 | STE24 endopeptidase |
| CNXL_027440 | CND04930 | 0.979944135 | 0.002661542 | hypothetical protein |
| CNXL_027450 | CND04940 | 0.584617664 | 0.062208681 | phosphatidylserine decarboxylase |
| CNXL_027460 | CND04950 | 0.451235631 | 0.258951716 | hypothetical protein |
| CNXL_027470 | | 0.263759187 | 0.487397795 | aspartyl-tRNA |
| CNXL_027480 | CND04960 | 0.386768646 | 0.394293269 | high-affinity nicotinic acid transporter |
| CNXL_027490 | CND04970 | -0.168455209 | 0.776535714 | Unknown |
| CNXL_027500 | CND04980 | 0.616973765 | 0.047289945 | hypothetical protein |
| CNXL_027510 | CND04990 | 1.102506312 | 0.001214534 | phosphatidylinositol glycan |
| CNXL_027520 | CND05000 | 0.5660732 | 0.157818222 | hypothetical protein |
| CNXL_027530 | CND05010 | -0.514557148 | 0.541840271 | hypothetical protein |
| CNXL_027540 | CND05020 | -0.708435704 | 0.040745404 | hypothetical protein |
| CNXL_027550 | CND05030 | 0.02406656 | 0.978139473 | adenine phosphoribosyltransferase |
| CNXL_027560 | CND05040 | 0.202001929 | 0.735456246 | cell division cycle protein 37 |
| CNXL_027570 | CND05050 | -0.085763906 | 0.919952257 | hypothetical protein |
| CNXL_027580 | | 0.173601798 | 0.771239252 | hypothetical protein |
| CNXL_027590 | | 0.050579102 | 0.964845459 | hypothetical protein |
| CNXL_027600 | CND05060 | -0.417262112 | 0.356885743 | hypothetical protein |
| CNXL_027610 | CND05070 | -0.077252945 | 0.911130835 | ribose-5-phosphate isomerase |
| CNXL_027620 | CND05080 | -0.148997268 | 0.798090976 | protein SUS1 |
| CNXL_027630 | CND05090 | -0.165963177 | 0.747332879 | hypothetical protein |
| CNXL_027640 | CND05100 | 0.066365804 | 0.927441884 | ATP-dependent RNA helicase DOB1 |
| CNXL_027650 | | 0.256474775 | 0.740096919 | hypothetical protein |
| CNXL_027660 | | -0.058809339 | 0.939400455 | Unknown |
| CNXL_027670 | CND05120 | -0.394275634 | 0.381569079 | Unknown |
| CNXL_027680 | CND05130 | -1.361128984 | 0.071408618 | 3-deoxy-7-phosphoheptulonate synthase |
| CNXL_027690 | | 0.332249213 | 0.500319755 | hypothetical protein |
| CNXL_027700 | CND05140 | 0.42183308 | 0.262925589 | Unknown |
| CNXL_027710 | CND05150 | -0.044885584 | 0.958917101 | actin binding protein |
| CNXL_027720 | CND05160 | -0.748458831 | 0.044126274 | eclair-PA |
| CNXL_027730 | CND05170 | -0.256843456 | 0.594692631 | hsp71-like protein |
| CNXL_027740 | CND05180 | -0.360536987 | 0.421670869 | hypothetical protein |
| CNXL_027750 | CND05200 | -0.220490208 | 0.73987454 | hypothetical protein |
| CNXL_027760 | CND05210 | -0.020006381 | 0.985595171 | PH and SEC7 domain-containing protein |
| CNXL_027770 | | -0.598419412 | 0.216968611 | scaffold-type e3 ligase |
| CNXL_027780 | CND05230 | -0.146517917 | 0.807491917 | Unknown |
| CNXL_027790 | CND05240 | -0.229801742 | 0.615722567 | phosphatidate phosphatase LPIN |
| CNXL_027800 | CND05250 | -0.223463224 | 0.62364132 | putative polyadenylated mRNA-binding protein |
| CNXL_027810 | CND05260 | 0.197618029 | 0.714520393 | hypothetical protein |
| CNXL_027820 | CND05270 | 0.173210675 | 0.757739987 | cytoplasmic protein |

| | | | | |
|---|---|---|---|---|
| CNXL_027830 | CND05280 | 0.979872962 | 0.002570666 | hypothetical protein |
| CNXL_027840 | CND05290 | 0.30441901 | 0.474675516 | hypothetical protein |
| CNXL_027850 | | 0.734774704 | 0.053355967 | hypothetical protein |
| CNXL_027860 | CND05310 | 0.653395535 | 0.070979277 | Unknown |
| CNXL_027870 | CND05320 | -0.398812995 | 0.308102113 | hypothetical protein |
| CNXL_027880 | CND05330 | -0.582537194 | 0.044950679 | hypothetical protein |
| CNXL_027890 | CND05340 | 1.005651089 | 0.004408514 | hypothetical protein |
| CNXL_027900 | CND05350 | 0.027392163 | 0.970515325 | endoplasmic reticulum protein |
| CNXL_027910 | CND05360 | 0.273105408 | 0.510006134 | hypothetical protein |
| CNXL_027920 | CND05370 | 0.509453604 | 0.453722961 | hypothetical protein |
| CNXL_027930 | CND05390 | 0.205918805 | 0.648500229 | hypothetical protein |
| CNXL_027940 | CND05400 | -0.218982764 | 0.623094586 | vacuolar protein sorting 26 |
| CNXL_027950 | CND05410 | 0.267307343 | 0.570476424 | translation initiation factor 5A |
| CNXL_027960 | CND05430 | -0.020298485 | 0.985173026 | hypothetical protein |
| CNXL_027970 | CND05440 | 0.072855949 | 0.919196787 | hypothetical protein |
| CNXL_027980 | CND05450 | 0.106739484 | 0.867958111 | hypothetical protein |
| CNXL_027990 | CND05480 | -0.658287302 | 0.103546355 | ribosome assembly protein 4 |
| CNXL_028000 | CND05490 | -0.072850764 | 0.91272593 | hypothetical protein |
| CNXL_028010 | CND05500 | 0.251996766 | 0.608495206 | serine-tRNA ligase |
| CNXL_028020 | CND05510 | 0.013777717 | 0.986747477 | hypothetical protein |
| CNXL_028030 | CND05520 | -0.44402801 | 0.406574476 | ribosomal RNA assembly protein |
| CNXL_028040 | CND05530 | 0.083000703 | 0.906238287 | transcription factor |
| CNXL_028050 | CND05540 | 0.844702377 | 0.02488041 | U4/U6 small nuclear ribonucleoprotein PRP4 |
| CNXL_028060 | CND05550 | 0.205616776 | 0.679276465 | hypothetical protein |
| CNXL_028070 | CND05560 | 0.066360869 | 0.916191462 | hypothetical protein |
| CNXL_028080 | CND05570 | -0.168327426 | 0.814480545 | cysteine desulfurase IscS |
| CNXL_028090 | CND05580 | 0.141075266 | 0.824564918 | hypothetical protein |
| CNXL_028100 | CND05590 | 0.121512613 | 0.830676885 | mRNA polymerase II subunit |
| CNXL_028110 | CND05600 | 0.943674715 | 0.006918635 | autophagy-related protein |
| CNXL_028120 | CND05620 | -0.080221739 | 0.916191462 | heat shock protein |
| CNXL_028130 | CND05630 | 0.417753086 | 0.261605186 | hypothetical protein |
| CNXL_028140 | CND05640 | -0.440107652 | 0.331351174 | pantoate-beta-alanine ligase |
| CNXL_028150 | | -0.67536966 | 0.112183936 | putative nucleolar gtp-binding protein |
| CNXL_028160 | CND05650 | -0.232490012 | 0.663234353 | Unknown |
| CNXL_028170 | CND05660 | 0.241469234 | 0.557606572 | mat3 pheromone repeat protein |
| CNXL_028180 | CND05670 | -0.274128772 | 0.492041786 | serine/threonine-protein kinase |
| CNXL_028190 | | -4.661041262 | 5.97947E-92 | btp/pz domain protein |
| CNXL_028200 | | -5.002839628 | 1.51817E-64 | Unknown |
| CNXL_028210 | CND00010 | -0.298981833 | 0.534266327 | Unknown |
| CNXL_028220 | CND05730 | -0.208508878 | 0.663234353 | Unknown |
| CNXL_028230 | CND05740 | -0.304763207 | 0.413169928 | Unknown |
| CNXL_028240 | 680\|CND05750\|CN | -2.81142539 | 3.14286E-41 | Unknown |
| CNXL_028250 | ND05760\|CND057 | -0.031266 | 0.963420993 | mating-type pheromone alpha |
| CNXL_028260 | CND05780 | -0.096777689 | 0.863513463 | Unknown |
| CNXL_028270 | CND05790 | -0.043445999 | 0.95265881 | myosin class v heavy chain |
| CNXL_028280 | | -0.616411843 | 0.346662055 | ste/ste20/paka protein kinase |
| CNXL_028290 | CND05800 | -3.246942798 | 4.73305E-47 | Unknown |
| CNXL_028300 | CND05810 | -0.882672473 | 0.001159796 | a-factor pheromone receptor |
| CNXL_028310 | CND05820 | -0.612777593 | 0.078954247 | ste-like transcription factor |
| CNXL_028320 | CND05830 | 0.242522476 | 0.57573283 | DNA-directed mRNA polymerase |
| CNXL_028330 | CND05840 | -0.196906336 | 0.638320737 | armadillo/beta-catenin repeat protein |
| CNXL_028340 | CND05850 | -0.121630452 | 0.830440343 | putative dihydrolipoyl dehydrogenase |
| CNXL_028350 | CND05860 | -0.393420369 | 0.496574337 | cid1-family polyA polymerase |
| CNXL_028360 | | -0.063285846 | 0.934642219 | hypothetical protein |
| CNXL_028370 | | -0.329051565 | 0.55313399 | Unknown |
| CNXL_028380 | | -0.906976704 | 0.078923725 | Unknown |
| CNXL_028390 | CND05880 | 0.930572386 | NA | Unknown |
| CNXL_028400 | CNB02520 | 4.075200997 | NA | Unknown |

| | | | | |
|---|---|---|---|---|
| CNXL_028410 | CND05900 | -0.104572569 | 0.86171371 | Unknown |
| CNXL_028420 | | 0.963427442 | 0.017977728 | protein of unknown function |
| CNXL_028430 | CND05910 | -0.092430559 | 0.874351584 | Unknown |
| CNXL_028440 | CND05920 | -0.055739184 | 0.934219437 | large subunit ribosomal protein L22e |
| CNXL_028450 | CND05940 | 0.131570408 | 0.801583128 | phospholipase D1 |
| CNXL_028460 | CND05950 | -3.028068672 | 1.40855E-38 | O-glucosyltransferase |
| CNXL_028470 | CND05960 | 0.074283882 | 0.920669589 | alpha cell-type homeodomain transcription factor |
| CNXL_028480 | CND05970 | 0.004128753 | 0.996158838 | putative fatty alcohol oxidase in the mating-type locus |
| CNXL_028490 | CND05980 | -0.337615291 | 0.445982809 | hypothetical protein |
| CNXL_028500 | CND05990 | 0.283839378 | 0.606726524 | uric acid xanthine permease |
| CNXL_028510 | | -0.193921553 | 0.701325477 | Zn2-Cys6 transcription factor |
| CNXL_028520 | CND06010 | -0.171463761 | 0.747332879 | Unknown |
| CNXL_028530 | | 0.612116094 | 0.056873107 | DNA helicase INO80 |
| CNXL_028540 | CND06030 | 0.279093469 | 0.524368764 | hypothetical protein |
| CNXL_028550 | CND06040 | -0.968467471 | 0.013983372 | ferric reductase transmembrane component |
| CNXL_028560 | CND06050 | 1.142616289 | 0.552790986 | hypothetical protein |
| CNXL_028570 | CND06060 | -0.563469125 | 0.296205552 | hypothetical protein |
| CNXL_028580 | CND06070 | -0.290483941 | 0.602461418 | hypothetical protein |
| CNXL_028590 | CND06080 | 0.100750596 | 0.914338536 | hypothetical protein |
| CNXL_028600 | ND06090\|CND061( | 0.276628937 | 0.483451258 | hypothetical protein |
| CNXL_028610 | CND06110 | -0.677849614 | 0.040667013 | Unknown |
| CNXL_028620 | CND06120 | -0.531327403 | 0.249615143 | squalene monooxygenase |
| CNXL_028630 | CND06130 | -0.269205888 | 0.615520877 | histidinol dehydrogenase |
| CNXL_028640 | CND06140 | -2.317785618 | NA | putative beta-glucan synthase |
| CNXL_028650 | CND06150 | 0.159882896 | 0.773095399 | Unknown |
| CNXL_028660 | CND06160 | -0.895419354 | 0.00086093 | protein-O-mannosyltransferase |
| CNXL_028670 | CND06170 | -0.87496687 | 0.009434938 | putative beta-glucan synthase |
| CNXL_028680 | CND06180 | 0.021265381 | 0.981240834 | oxidoreductase |
| CNXL_028690 | CND06190 | 0.131672391 | 0.847923235 | PH domain-containing protein |
| CNXL_028700 | CND06200 | -0.116569949 | 0.861349479 | mitochondrial RNA helicase |
| CNXL_028710 | CND06220 | -0.19587215 | 0.636752734 | methylene-fatty-acyl-phospholipid synthase |
| CNXL_028720 | CND06230 | -0.271472652 | 0.614293431 | elongation factor 2 |
| CNXL_028730 | | 0.312192724 | 0.560646266 | AAA-peroxin |
| CNXL_028740 | | 0.217320344 | 0.800552138 | pantothenate transporter |
| CNXL_028750 | CND06250 | -0.293935301 | 0.519652797 | Unknown |
| CNXL_028760 | CND06260 | 0.324021731 | 0.482812185 | STE/STE11/CDC15 protein kinase |
| CNXL_028770 | | 0.580498106 | 0.227069466 | hypothetical protein |
| CNXL_028780 | CND06280 | -0.213099755 | 0.641029652 | hypothetical protein |
| CNXL_028790 | CND06290 | -0.239540624 | 0.685425894 | small subunit ribosomal protein S28 |
| CNXL_028800 | CND06300 | -0.496048749 | 0.26395447 | saccharopine dehydrogenase |
| CNXL_028810 | CND06310 | 0.142503109 | 0.884028948 | aspartate-tRNA |
| CNXL_028820 | CND06330 | -0.012510494 | 0.988670326 | transcriptional coactivator HFI1/ADA1 |
| CNXL_028830 | CND06350 | -3.028274136 | 1.62974E-18 | hypothetical protein |
| CNXL_028840 | | -5.635225274 | 3.67715E-17 | Unknown |
| CNXL_028850 | | -0.426895392 | 0.776232213 | hypothetical protein |
| CNXL_028860 | CND06360 | -0.130301874 | 0.879713683 | Unknown |
| CNXL_028870 | CND06370 | 0.047216251 | 0.95265881 | Unknown |
| CNXL_028880 | CND06390 | -0.420286139 | 0.253430843 | hypothetical protein |
| CNXL_028890 | CND06400 | -0.257452742 | 0.638899901 | nuclear protein |
| CNXL_028900 | CND06410 | 0.806987965 | 0.008148747 | putative TFIID and saga complex component |
| CNXL_028910 | | 1.014787834 | 0.013529045 | vacuolar protein |
| CNXL_028920 | | 0.083340151 | 0.964414864 | Unknown |
| CNXL_028930 | | -0.365195431 | 0.443600568 | Unknown |
| CNXL_028940 | | 0.111444062 | 0.861981252 | Unknown |
| CNXL_028950 | NE00020\|CNE052( | 0 | NA | Unknown |
| CNXL_028960 | NE00040\|CNE0525 | 4.181166125 | 0.108539336 | HPP family protein |
| CNXL_028970 | NE00050\|CNE0524 | 0.978902335 | 0.037278381 | phosphopyruvate hydratase |
| CNXL_028980 | NE00070\|CNE0522 | 0.022189255 | 0.995735232 | hypothetical protein |

| | | | | |
|---|---|---|---|---|
| CNXL_028990 | CNE00080 | 0 | NA | translation machinery-associated protein 16 |
| CNXL_029000 | | -0.508084281 | 0.335282372 | hypothetical protein |
| CNXL_029010 | CNE00110 | 0.470749721 | 0.175271548 | Unknown |
| CNXL_029020 | | 0.324677277 | 0.69874241 | Zn2-Cys6 zinc-finger transcription factor |
| CNXL_029030 | CNE00120 | 0.822744102 | 0.004325636 | Unknown |
| CNXL_029040 | NE00130\|CNE001 | 0.437448818 | 0.141169725 | sugar transporter |
| CNXL_029050 | CNE00150 | 1.099205398 | 0.000336086 | Unknown |
| CNXL_029060 | CNE00160 | 0.415777728 | 0.294515322 | hypothetical protein |
| CNXL_029070 | CNE00170 | 0.475903192 | 0.13288731 | hypothetical protein |
| CNXL_029080 | CNE00190 | -0.156821278 | 0.793711563 | cobalamin synthesis protein |
| CNXL_029090 | CNE00200 | -0.268342546 | 0.540113713 | nuclear pore complex protein Nup205 |
| CNXL_029100 | CNE00210 | 0.058765312 | 0.921544337 | transferase |
| CNXL_029110 | CNE00220 | -4.276758487 | 4.24586E-05 | inorganic pyrophosphatase |
| CNXL_029120 | CNE00230 | 1.014861293 | 0.000886974 | DNA repair protein Swi5/Sae3 |
| CNXL_029130 | CNE00240 | -0.333298426 | 0.354037215 | hypothetical protein |
| CNXL_029140 | CNE00250 | -0.240265262 | 0.628625129 | fructosamine kinase |
| CNXL_029150 | CNE00260 | -0.627517072 | 0.061588564 | cyclin-dependent protein kinase inhibitor |
| CNXL_029160 | NE00270\|CNE002 | -0.41522315 | 0.232022599 | hypothetical protein |
| CNXL_029170 | CNC05520 | 0.154905884 | 0.911130835 | AAT family amino acid transporter |
| CNXL_029180 | CNE00290 | -0.206234363 | 0.776535714 | hypothetical protein |
| CNXL_029190 | CNE00300 | -0.073853796 | 0.939627791 | putative TFIID and saga complex component |
| CNXL_029200 | CNE00310 | -0.266253504 | 0.749050005 | hypothetical protein |
| CNXL_029210 | CNE00320 | 0.029705187 | 0.970488713 | tRNA 2'-phosphotransferase |
| CNXL_029220 | CNE00330 | -0.193814373 | 0.765354566 | hypothetical protein |
| CNXL_029230 | CNE00340 | 0.207843884 | 0.703985737 | D-amino-acid oxidase |
| CNXL_029240 | | 0.347320979 | 0.534447681 | Unknown |
| CNXL_029250 | CNE00350 | -2.101598422 | 1.04479E-10 | Unknown |
| CNXL_029260 | CNE00360 | -0.308769306 | 0.524381096 | mitogen activated protein kinase |
| CNXL_029270 | CNE00370 | -0.83177633 | 0.042705195 | hypothetical protein |
| CNXL_029280 | CNE00380 | -1.978245564 | 4.63373E-12 | hypothetical protein |
| CNXL_029290 | CNE00390 | 0.066618909 | 0.925141393 | hypothetical protein |
| CNXL_029300 | CNE00400 | -1.788145433 | 9.43932E-08 | multidrug transporter |
| CNXL_029310 | CNE00410 | -0.411958874 | 0.394293269 | hypothetical protein |
| CNXL_029320 | CNE00420 | 0.1904569 | 0.746237137 | hypothetical protein |
| CNXL_029330 | | -1.201063921 | 0.000623829 | hypothetical protein |
| CNXL_029340 | | -0.840077945 | 0.028997881 | hypothetical protein |
| CNXL_029350 | CNE00440 | 0.035298568 | 0.963190895 | Unknown |
| CNXL_029360 | | -0.534977034 | 0.548869305 | mitochondrial carrier protein |
| CNXL_029370 | CNE00450 | -0.316403217 | 0.613703345 | hypothetical protein |
| CNXL_029380 | CNE00460 | 0.132388982 | 0.809305305 | DNA-directed RNA polymerase III subunit RPC4 |
| CNXL_029390 | CNE00470 | -0.020459742 | 0.982102702 | mitochondria fission 1 protein |
| CNXL_029400 | CNE00480 | 0.262361924 | 0.65287141 | hypothetical protein |
| CNXL_029410 | CNE00490 | -0.103211331 | 0.87555787 | hypothetical protein |
| CNXL_029420 | CNE00500 | 0.121804986 | 0.842469317 | hypothetical protein |
| CNXL_029430 | CNE00510 | -0.292449408 | 0.474482847 | COP9 signalosome complex subunit 1 |
| CNXL_029440 | CNE00520 | -1.729634784 | 4.32459E-13 | DNA repair protein RAD16 |
| CNXL_029450 | CNE00530 | -0.490996128 | 0.194911786 | CMGC/MAPK protein kinase |
| CNXL_029460 | | -1.356905342 | 0.05908683 | hypothetical protein |
| CNXL_029470 | CNE00540 | -1.162793294 | 0.001095047 | hypothetical protein |
| CNXL_029480 | CNE00550 | -0.276966405 | 0.647485945 | hypothetical protein |
| CNXL_029490 | CNE00560 | -0.001864861 | 0.996833113 | translation initiation factor 3 subunit H |
| CNXL_029500 | CNE00570 | -0.22810937 | 0.701325477 | 3 |
| CNXL_029510 | | -0.21823743 | 0.64885828 | cardiolipin-specific phospholipase |
| CNXL_029520 | CNE00580 | 0.36717586 | 0.445982809 | hypothetical protein |
| CNXL_029530 | CNE00590 | -0.17739756 | 0.766858347 | glutathione peroxidase |
| CNXL_029540 | CNE00600 | 0.259171545 | 0.584869453 | ATP-dependent RNA helicase DBP3 |
| CNXL_029550 | CNE00610 | -0.085419004 | 0.88268871 | hypothetical protein |
| CNXL_029560 | | -2.250052787 | NA | calnexin |

| | | | | |
|---|---|---|---|---|
| CNXL_029570 | CNE00620 | 0.079561461 | 0.902044607 | hypothetical protein |
| CNXL_029580 | CNE00630 | -0.555067171 | 0.083397364 | hypothetical protein |
| CNXL_029590 | CNE00640 | 0.309512721 | 0.434147477 | thaumatin family protein |
| CNXL_029600 | CNE00650 | -0.186484703 | 0.78519633 | hypothetical protein |
| CNXL_029610 | CNE00660 | -0.10485691 | 0.912281765 | cytoplasmic protein |
| CNXL_029620 | CNE00670 | 0.120156723 | 0.842469317 | carboxypeptidase D |
| CNXL_029630 | CNE00680 | 0.071709011 | 0.917445028 | hypothetical protein |
| CNXL_029640 | CNE00690 | -0.727367013 | 0.096551642 | peptidyl-prolyl cis-trans isomerase-like 3 |
| CNXL_029650 | CNE00700 | 0.090196003 | 0.8877677 | nuclear protein |
| CNXL_029660 | CNE00710 | -0.334362498 | 0.393252659 | DNA repair protein MRE11 |
| CNXL_029670 | CNE00720 | -1.459420056 | 6.47706E-07 | alcohol dehydrogenase |
| CNXL_029680 | CNE00730 | -0.022919547 | 0.982102702 | methionyl-tRNA formyltransferase |
| CNXL_029690 | CNE00740 | -0.253685073 | 0.542938863 | 3-hydroxy acyl-CoA dehydratase |
| CNXL_029700 | CNE00750 | 0.097102025 | 0.856294543 | translation initiation factor 2 subunit 2 |
| CNXL_029710 | CNE00760 | -0.031724734 | 0.970515325 | ATP synthase F1 |
| CNXL_029720 | CNE00770 | 0.372263099 | 0.427104346 | peptidyl-prolyl cis-trans isomerase |
| CNXL_029730 | CNE00780 | -0.291357553 | 0.660663885 | plasma membrane protein |
| CNXL_029740 | CNE00790 | -0.053151596 | 0.944263762 | hypothetical protein |
| CNXL_029750 | CNE00800 | 0.017153886 | 0.986615879 | translation machinery-associated protein 22 |
| CNXL_029760 | CNE00810 | 0.162667089 | 0.779355929 | vacuolar protein |
| CNXL_029770 | CNE00820 | -0.520227929 | 0.123223552 | cell cycle checkpoint control protein RAD9A |
| CNXL_029780 | CNE00830 | 0.341978843 | 0.5305986 | glycerol dehydrogenase |
| CNXL_029790 | CNE00840 | 0.04288293 | 0.962706186 | hypothetical protein |
| CNXL_029800 | CNE00850 | -0.003148095 | 0.996472611 | putative transcription factor |
| CNXL_029810 | CNE00860 | 0.284760247 | 0.70913058 | flavin-containing monooxygenase |
| CNXL_029820 | CNE00870 | -0.004163928 | 0.995735232 | NAD-binding Rossmann fold oxidoreductase |
| CNXL_029830 | CNE00880 | -0.142468247 | 0.806888133 | endoplasmic reticulum protein |
| CNXL_029840 | | 0.13936683 | 0.814730279 | endosomal P24A protein |
| CNXL_029850 | CNE00890 | 0.291876866 | 0.484800737 | hypothetical protein |
| CNXL_029860 | CNE00900 | 0.212400636 | 0.688968135 | phosphoric monoester hydrolase |
| CNXL_029870 | CNE00910 | 0.150476779 | 0.789040329 | cytoplasmic protein |
| CNXL_029880 | CNE00920 | 0.302357117 | 0.487463273 | AP-3 complex subunit delta-1 |
| CNXL_029890 | | 0.312387878 | 0.503339903 | DNA repair/transcription protein MET18/MMS19 |
| CNXL_029900 | CNE00930 | 0.500668113 | 0.179703559 | Unknown |
| CNXL_029910 | CNE00940 | 0.896254688 | 0.001651572 | hypothetical protein |
| CNXL_029920 | | 0.61342776 | 0.064192122 | allantoate permease |
| CNXL_029930 | CNE00950 | 0.232766799 | 0.566457057 | Unknown |
| CNXL_029940 | CNE00960 | -0.049901338 | 0.981240834 | 5-oxoprolinase |
| CNXL_029950 | CNE00970 | -0.07351441 | 0.927441884 | hypothetical protein |
| CNXL_029960 | | -0.388898132 | 0.586215516 | hypothetical protein |
| CNXL_029970 | CNE00980 | -1.797085029 | 1.16954E-08 | Unknown |
| CNXL_029980 | CNE00990 | -0.54291504 | 0.226593506 | coproporphyrinogen-III oxidase |
| CNXL_029990 | CNE01000 | -0.288203089 | 0.544657787 | Atypical/ABC1/ABC1-A protein kinase |
| CNXL_030000 | CNE01010 | -0.369412471 | 0.41106632 | GTPase activating protein |
| CNXL_030010 | CNE01020 | -0.626910939 | 0.028838358 | importin beta-2 subunit |
| CNXL_030020 | CNE01030 | 0.815849079 | 0.021247224 | translation initiation factor 3 subunit C |
| CNXL_030030 | CNE01050 | -0.185165869 | 0.777814879 | choline transporter |
| CNXL_030040 | CNE01060 | -0.063839727 | 0.919822815 | hypothetical protein |
| CNXL_030050 | CNE01070 | 0.081393369 | 0.906291044 | hypothetical protein |
| CNXL_030060 | CNE01080 | 0.695087248 | 0.03627015 | hypothetical protein |
| CNXL_030070 | CNE01090 | 0.408316954 | 0.234477661 | endopolyphosphatase |
| CNXL_030080 | CNE01100 | -0.377857278 | 0.488916159 | U3 small nucleolar ribonucleoprotein IMP4 |
| CNXL_030090 | CNE01110 | 0.310148834 | 0.516378636 | long-chain fatty acid CoA ligase |
| CNXL_030100 | CNE01120 | -0.094592552 | 0.885268793 | hypothetical protein |
| CNXL_030110 | CNE01130 | 0.394848621 | 0.264827301 | response regulator receiver protein |
| CNXL_030120 | CNE01140 | 0.401130995 | 0.331688924 | phosphoacetylglucosamine mutase |
| CNXL_030130 | CNE01150 | -0.105008379 | 0.855058004 | hypothetical protein |
| CNXL_030140 | CNE01160 | 0.189189682 | 0.75185116 | NADH dehydrogenase |

| | | | | |
|---|---|---|---|---|
| CNXL_030150 | | 0.686887248 | 0.539585872 | hypothetical protein |
| CNXL_030160 | CNE01170 | -0.740365767 | 0.050467753 | hypothetical protein |
| CNXL_030170 | CNE01180 | 0.122487602 | 0.848988082 | cation-transporting ATPase |
| CNXL_030180 | | 0.716517174 | 0.057424997 | hypothetical protein |
| CNXL_030190 | | 0.584408176 | 0.227019254 | hypothetical protein |
| CNXL_030200 | CNE01200 | -0.522368207 | 0.123440865 | Unknown |
| CNXL_030210 | NE01210\|CNE012 | 0.348908779 | 0.368884101 | rRNA biogenesis protein RRP5 |
| CNXL_030220 | CNE01230 | -0.463510495 | 0.164354975 | Unknown |
| CNXL_030230 | CNE01240 | -0.075547271 | 0.902229878 | putative copper ion transporter |
| CNXL_030240 | CNE01250 | -0.850681255 | 0.144421956 | thiol oxidase |
| CNXL_030250 | CNE01260 | 0.360523256 | 0.462832736 | hypothetical protein |
| CNXL_030260 | CNE01270 | 0.145477992 | 0.781247582 | TatD DNase |
| CNXL_030270 | CNE01280 | -0.044063382 | 0.949958757 | ABC transporter ABCC.6 |
| CNXL_030280 | CNE01300 | 1.599293681 | 2.81104E-08 | GC-rich sequence DNA-binding factor |
| CNXL_030290 | CNE01310 | -0.084865271 | 0.893873062 | hypothetical protein |
| CNXL_030300 | CNE01320 | -0.129556136 | 0.874670769 | pre-mRNA-splicing factor SYF1 |
| CNXL_030310 | CNE01330 | -0.231511289 | 0.746765994 | hypothetical protein |
| CNXL_030320 | CNE01340 | -0.461664694 | 0.318004271 | hypothetical protein |
| CNXL_030330 | CNE01350 | 0.479366781 | 0.158608203 | hypothetical protein |
| CNXL_030340 | CNE01360 | -0.063384163 | 0.924136135 | hypothetical protein |
| CNXL_030350 | CNE01370 | 0.396690982 | 0.315126975 | hypothetical protein |
| CNXL_030360 | CNE01380 | -0.093828892 | 0.90564498 | hypothetical protein |
| CNXL_030370 | CNE01390 | -0.50140089 | 0.365472431 | hypothetical protein |
| CNXL_030380 | CNE01400 | 0.820147351 | 0.040601652 | asparagine-tRNA ligase |
| CNXL_030390 | | -0.038125258 | 0.972638293 | lipase/esterase |
| CNXL_030400 | CNE01410 | -0.675987616 | 0.111375561 | hypothetical protein |
| CNXL_030410 | | 0.039444818 | 0.972296773 | annexin XIV |
| CNXL_030420 | CNE01430 | 0.149243091 | 0.781580785 | hypothetical protein |
| CNXL_030430 | CNE01440 | 0.174914596 | 0.727102527 | hypothetical protein |
| CNXL_030440 | | 0.674455578 | 0.081165364 | rRNA-processing protein CGR1 |
| CNXL_030450 | CNE01450 | -0.009551199 | 0.991141666 | hypothetical protein |
| CNXL_030460 | CNE01460 | -0.117516171 | 0.869733568 | hypothetical protein |
| CNXL_030470 | CNE01470 | 0.099295549 | 0.91647607 | protein of unknown function |
| CNXL_030480 | CNE01480 | 0.868808463 | 0.010553176 | hypothetical protein |
| CNXL_030490 | | 1.039638336 | 0.003956119 | phospholipase D |
| CNXL_030500 | CNE01490 | 0.582634098 | 0.187369695 | Unknown |
| CNXL_030510 | CNE01500 | 0.385315852 | 0.39417374 | cell division control protein 45 |
| CNXL_030520 | CNE01510 | 0.079199116 | 0.892724552 | hypothetical protein |
| CNXL_030530 | | 0.031525529 | 0.965101267 | nuclear segregation protein Bfr1 |
| CNXL_030540 | CNE01530 | 0.232098058 | 0.643890318 | NADH dehydrogenase |
| CNXL_030550 | CNE01540 | -0.050939752 | 0.944879244 | hypothetical protein |
| CNXL_030560 | CNE01550 | 0.078518353 | 0.903282886 | G patch domain-containing protein 1 |
| CNXL_030570 | CNE01560 | 0.254876381 | 0.553726767 | hypothetical protein |
| CNXL_030580 | CNE01570 | 1.176674948 | 0.000435335 | glutathione oxidoreductase |
| CNXL_030590 | CNE01580 | 0.451392008 | 0.304500885 | hypothetical protein |
| CNXL_030600 | CNE01590 | 0.115564542 | 0.860094261 | hypothetical protein |
| CNXL_030610 | CNE01600 | 0.030151819 | 0.969527869 | hypothetical protein |
| CNXL_030620 | CNE01610 | 0.084074297 | 0.898899892 | ubiquitin carboxyl-terminal hydrolase 9/13 |
| CNXL_030630 | CNE01620 | -0.946330138 | 0.003214347 | hypothetical protein |
| CNXL_030640 | CNE01630 | -1.984822678 | 2.12202E-13 | hypothetical protein |
| CNXL_030650 | | -0.056736206 | 0.946605516 | AGC protein kinase |
| CNXL_030660 | CNE01640 | 0.336022262 | 0.393252659 | hypothetical protein |
| CNXL_030670 | | -0.290870984 | 0.677906547 | hypothetical protein |
| CNXL_030680 | CNE01650 | -0.718847306 | 0.17290643 | hypothetical protein |
| CNXL_030690 | | -0.469997742 | 0.489809679 | DNA primase small subunit |
| CNXL_030700 | CNE01660 | 0.079613 | 0.906691628 | Unknown |
| CNXL_030710 | CNE01670 | 0.150528354 | 0.771763303 | trimethylguanosine synthase |
| CNXL_030720 | CNE01680 | -0.878653763 | 0.450585467 | ribosome biogenesis protein BRX1 |

| | | | | |
|---|---|---|---|---|
| CNXL_030730 | CNE01690 | -0.518065414 | 0.689555844 | hypothetical protein |
| CNXL_030740 | CNE01700 | -0.326056436 | 0.524381096 | hypothetical protein |
| CNXL_030750 | CNE01710 | 0.002258234 | 0.996472611 | hypothetical protein |
| CNXL_030760 | CNE01720 | 0.388762377 | 0.303744123 | H/ACA ribonucleoprotein complex subunit 2 |
| CNXL_030770 | CNE01730 | 0.218469655 | 0.635378645 | aldehyde dehydrogenase |
| CNXL_030780 | CNE01740 | 0.034162293 | 0.970876289 | hypothetical protein |
| CNXL_030790 | CNE01760 | -0.155316485 | 0.802313068 | polyphosphoinositide phosphatase |
| CNXL_030800 | CNE01770 | 0.510658284 | 0.190652363 | hypothetical protein |
| CNXL_030810 | CNE01780 | 0.130199845 | 0.818585852 | ATP-binding protein |
| CNXL_030820 | CNE01790 | -0.011724016 | 0.990777629 | enoyl reductase |
| CNXL_030830 | CNE01810 | -0.118201035 | 0.855305199 | coiled-coil domain-containing protein 130 |
| CNXL_030840 | CNE01820 | -0.171883794 | 0.746237137 | hypothetical protein |
| CNXL_030850 | CNE01830 | 0.034802128 | 0.969527869 | GTP-binding protein ypt3 |
| CNXL_030860 | CNE01840 | -0.458576007 | 0.38033895 | 4-aminobutyrate aminotransferase |
| CNXL_030870 | CNE01850 | 0.023580194 | 0.977521185 | amidase |
| CNXL_030880 | CNE01860 | 0.621307037 | 0.067840292 | hypothetical protein |
| CNXL_030890 | CNE01870 | 0.074091653 | 0.916084262 | SCR1 protein |
| CNXL_030900 | CNE01880 | -0.318886555 | 0.628160086 | hypothetical protein |
| CNXL_030910 | CNE01900 | 0.327591035 | 0.492659883 | MFS transporter |
| CNXL_030920 | CNE01910 | -0.145491378 | 0.781580785 | hypothetical protein |
| CNXL_030930 | CNE01920 | 0.103996118 | 0.884028948 | small subunit ribosomal protein S25e |
| CNXL_030940 | CNE01930 | -0.09942262 | 0.899804184 | MRP family ATP-binding protein |
| CNXL_030950 | CNE01940 | -0.078858671 | 0.935317161 | serine/threonine-protein kinase |
| CNXL_030960 | CNE01950 | -0.057137995 | 0.935317161 | phosphatidylinositol glycan |
| CNXL_030970 | CNE01960 | -0.291468116 | 0.615328522 | solute carrier family 35 |
| CNXL_030980 | CNE01970 | 0.039410164 | 0.965101267 | large subunit ribosomal protein L2 |
| CNXL_030990 | CNE01980 | 0.02517143 | 0.97704705 | betaine lipid synthase |
| CNXL_031000 | CNE01990 | -2.046068443 | 5.11078E-13 | hypothetical protein |
| CNXL_031010 | CNE02000 | 0.397189185 | 0.37135203 | endochitinase |
| CNXL_031020 | CNE02010 | 0.016057113 | 0.988556874 | hypothetical protein |
| CNXL_031030 | | -0.359888041 | 0.477835242 | hypothetical protein |
| CNXL_031040 | CNE02020 | 0.327499635 | 0.481511569 | hypothetical protein |
| CNXL_031050 | | 0.401226122 | 0.638320737 | hypothetical protein |
| CNXL_031060 | CNE02030 | 0.140296134 | 0.801947013 | hypothetical protein |
| CNXL_031070 | | -0.066359695 | 0.986880752 | polycomb protein EED |
| CNXL_031080 | CNE02040 | -1.318258016 | 2.04871E-05 | Unknown |
| CNXL_031090 | CNE02050 | -0.197256677 | 0.708742909 | uracil phosphoribosyltransferase |
| CNXL_031100 | CNE02060 | -0.182472206 | 0.809974479 | hypothetical protein |
| CNXL_031110 | CNE02070 | -0.330540351 | 0.452146814 | hypothetical protein |
| CNXL_031120 | CNE02080 | 0.140219399 | 0.814220979 | cyclin binding protein |
| CNXL_031130 | | -1.118703832 | 0.116400267 | hypothetical protein |
| CNXL_031140 | CNE02090 | -0.203793676 | 0.647175553 | hypothetical protein |
| CNXL_031150 | CNE02100 | 0.071499657 | 0.916084262 | cellular nucleic acid-binding protein |
| CNXL_031160 | CNE02110 | -0.633072572 | 0.098170709 | uracil phosphoribosyltransferase |
| CNXL_031170 | CNE02120 | 0.601781167 | 0.097567152 | hypothetical protein |
| CNXL_031180 | | -0.175134455 | 0.809453126 | hypothetical protein |
| CNXL_031190 | CNE02140 | 0.169339497 | 0.799344206 | hypothetical protein |
| CNXL_031200 | CNE02150 | 0.468854296 | 0.227892542 | hypothetical protein |
| CNXL_031210 | CNE02160 | -0.072160799 | 0.897988379 | hypothetical protein |
| CNXL_031220 | CNE02170 | -0.173785513 | 0.710705655 | small subunit ribosomal protein S9 |
| CNXL_031230 | | -0.109404587 | 0.913424974 | large subunit ribosomal protein L21e |
| CNXL_031240 | CNE02180 | 0.011803394 | 0.991351411 | Unknown |
| CNXL_031250 | CNE02190 | -0.456363383 | 0.193620149 | telomerase reverse transcriptase |
| CNXL_031260 | CNE02200 | -0.525135336 | 0.098322722 | pre-rRNA-processing protein TSR1 |
| CNXL_031270 | CNE02210 | 0.151456751 | 0.779355929 | xaa-Pro dipeptidase |
| CNXL_031280 | CNE02220 | -0.012724679 | 0.990777629 | V-type proton ATPase catalytic subunit A |
| CNXL_031290 | CNE02230 | -0.035159008 | 0.965966884 | mitochondrial protein |
| CNXL_031300 | CNE02240 | -0.578129907 | 0.062066354 | hypothetical protein |

| | | | | |
|---|---|---|---|---|
| CNXL_031310 | CNE02250 | -1.5731521 | 1.51464E-07 | hypothetical protein |
| CNXL_031320 | | 0.610828642 | 0.161387861 | hypothetical protein |
| CNXL_031330 | CNE02260 | 0.740961079 | 0.006714456 | hypothetical protein |
| CNXL_031340 | CNE02280 | -0.354603422 | 0.535382959 | hypothetical protein |
| CNXL_031350 | CNE02290 | -0.323683242 | 0.677486529 | hypothetical protein |
| CNXL_031360 | CNE02300 | -0.546476263 | 0.261879704 | protein arginine N-methyltransferase 3 |
| CNXL_031370 | CNE02310 | -0.499985795 | 0.118329883 | hypothetical protein |
| CNXL_031380 | | -0.017621906 | 0.992176872 | ubiquinol-cytochrome c reductase |
| CNXL_031390 | CNE02330 | 0.011331954 | 0.990703091 | Unknown |
| CNXL_031400 | CNE02340 | 0.639068996 | 0.061843613 | hypothetical protein |
| CNXL_031410 | CNE02350 | 0.194877829 | 0.721006731 | patatin-like phospholipase domain-containing protein |
| CNXL_031420 | CNE02360 | -0.131025092 | 0.811669553 | hypothetical protein |
| CNXL_031430 | CNE02370 | -0.063772448 | 0.939255348 | casein kinase II subunit beta |
| CNXL_031440 | CNE02380 | -0.158069637 | 0.776535714 | FK506-binding protein 2 |
| CNXL_031450 | CNE02390 | 0.074366347 | 0.927441884 | hypothetical protein |
| CNXL_031460 | CNE02400 | -0.045679249 | 0.948286148 | L-serine ammonia-lyase |
| CNXL_031470 | CNE02410 | -0.356362381 | 0.557292226 | molybdopterin binding domain-containing protein |
| CNXL_031480 | CNE02420 | 0.129447711 | 0.833643586 | hypothetical protein |
| CNXL_031490 | CNE02430 | 0.01914076 | 0.982102702 | mitochondrial distribution and morphology protein 34 |
| CNXL_031500 | | -2.662439265 | 4.88148E-08 | hypothetical protein |
| CNXL_031510 | CNE02440 | -0.124486752 | 0.816572532 | hypothetical protein |
| CNXL_031520 | CNE02450 | 0.368376201 | 0.346301684 | lupus La protein |
| CNXL_031530 | CNE02460 | -0.284176099 | 0.553056866 | hypothetical protein |
| CNXL_031540 | CNE02470 | 0.383242581 | 0.334533777 | hypothetical protein |
| CNXL_031550 | | 0.132459836 | 0.865225322 | niemann-Pick C1 protein |
| CNXL_031560 | CNE02480 | 0.131757058 | 0.868016272 | hypothetical protein |
| CNXL_031570 | CNE02490 | -0.060944001 | 0.942099307 | ATP-binding protein |
| CNXL_031580 | CNE02500 | -0.036150065 | 0.961771284 | phosphotransferase enzyme family protein |
| CNXL_031590 | CNE02510 | -0.166055665 | 0.78495956 | phosphoribosylaminoimidazole carboxylase |
| CNXL_031600 | CNE02520 | -0.157014297 | 0.802982005 | hypothetical protein |
| CNXL_031610 | | -0.419562908 | 0.350443288 | copper chaperone |
| CNXL_031620 | | -1.05719679 | 0.246642755 | DASH complex subunit DAM1 |
| CNXL_031630 | CNE02530 | -0.12862762 | 0.837479784 | Unknown |
| CNXL_031640 | CNE02550 | 0.041641497 | 0.958291218 | Unknown |
| CNXL_031650 | CNE02560 | 0.021793456 | 0.981240834 | parafibromin |
| CNXL_031660 | CNE02570 | -0.417639848 | 0.253643137 | rab family protein |
| CNXL_031670 | CNE02580 | -0.079785122 | 0.924607253 | solute carrier family 25 |
| CNXL_031680 | CNE02590 | -0.319123965 | 0.462811802 | hypothetical protein |
| CNXL_031690 | | 0.387895624 | 0.679138812 | cytoplasmic protein |
| CNXL_031700 | | 0.221135351 | 0.768488673 | Unknown |
| CNXL_031710 | CNE02610 | -0.312962665 | 0.391858761 | Unknown |
| CNXL_031720 | CNE02620 | -0.186343951 | 0.702418636 | nucleoside diphosphate kinase |
| CNXL_031730 | CNE02630 | 0.291204507 | 0.485492767 | 2-oxoisovalerate dehydrogenase E1 component |
| CNXL_031740 | CNE02650 | 0.792060975 | 0.006077564 | glucoamylase |
| CNXL_031750 | CNE02660 | 0.231397496 | 0.687407679 | hypothetical protein |
| CNXL_031760 | CNE02670 | 0.105637662 | 0.884028948 | hypothetical protein |
| CNXL_031770 | CNE02680 | -0.106124963 | 0.863513463 | kinetochore protein NDC80 |
| CNXL_031780 | CNE02690 | -0.137989713 | 0.842339591 | putative chitin synthase regulator |
| CNXL_031790 | CNE02700 | -1.360730461 | 6.61933E-07 | large subunit ribosomal protein L3 |
| CNXL_031800 | CNE02710 | 0.276210191 | 0.572479135 | hypothetical protein |
| CNXL_031810 | CNE02730 | 0.339334927 | 0.429445024 | triacylglycerol lipase |
| CNXL_031820 | CNE02720 | 0.147274711 | 0.801741833 | monosaccharide transporter |
| CNXL_031830 | CNE02740 | -0.122377998 | 0.874890451 | hypothetical protein |
| CNXL_031840 | CNE02750 | 0.339564058 | 0.365784664 | homoserine O-acetyltransferase |
| CNXL_031850 | CNE02770 | -0.032623489 | 0.965966884 | autophagy-like protein 18 Atg18 |
| CNXL_031860 | CNE02780 | 1.012477171 | 0.000535754 | cell cycle arrest protein BUB3 |
| CNXL_031870 | CNE02790 | -0.421255165 | 0.453650498 | hypothetical protein |
| CNXL_031880 | CNE02800 | -0.402894715 | 0.402210452 | preprotein translocase subunit YidC |

| | | | | |
|---|---|---|---|---|
| CNXL_031890 | NE02810\|CNE0282 | -0.194482551 | 0.671115959 | NADH-quinone oxidoreductase subunit B 2 |
| CNXL_031900 | CNE02830 | 1.138154973 | 0.000210233 | Unknown |
| CNXL_031910 | CNE02840 | 0.754318974 | 0.044849477 | hypothetical protein |
| CNXL_031920 | CNE02850 | -0.402558182 | 0.300430135 | ATP-binding cassette transporter |
| CNXL_031930 | CNE02860 | 0.136666218 | 0.787846251 | hypothetical protein |
| CNXL_031940 | CNE02870 | 0.00256843 | 0.996472611 | xap5-domain-containing protein |
| CNXL_031950 | CNE02880 | 0.461218942 | 0.193620149 | tRNA-dihydrouridine synthase 2 |
| CNXL_031960 | | 0.445647002 | 0.339325252 | hypothetical protein |
| CNXL_031970 | CNE02890 | -0.295850126 | 0.428821207 | Unknown |
| CNXL_031980 | CNE02900 | 0.419297616 | 0.200582774 | GTP-binding nuclear protein spi1 |
| CNXL_031990 | CNE02910 | 0.320039365 | 0.453868281 | BNR/Asp-box repeat family protein |
| CNXL_032000 | | 0.112833607 | 0.877322829 | quinate permease |
| CNXL_032010 | | NA | NA | Unknown |
| CNXL_032020 | | 0.095057595 | NA | Unknown |
| CNXL_032030 | | -2.364417376 | NA | Unknown |
| CNXL_032040 | CNE02980 | 1.724232775 | NA | Unknown |
| CNXL_032050 | | NA | NA | Unknown |
| CNXL_032060 | | 0.351240531 | 0.948286148 | Unknown |
| CNXL_032070 | | -0.347209078 | NA | Unknown |
| CNXL_032080 | | 1.484927388 | NA | Unknown |
| CNXL_032090 | | -1.330752087 | NA | Unknown |
| CNXL_032100 | CNN02020 | -0.424690855 | NA | Unknown |
| CNXL_032110 | | 1.484927388 | NA | Unknown |
| CNXL_032120 | | NA | NA | Unknown |
| CNXL_032130 | CNE03010 | 0.363880898 | 0.319480227 | Unknown |
| CNXL_032140 | CNE03020 | 0.048529898 | 0.939255348 | cytoplasmic protein |
| CNXL_032150 | CNE03030 | 0.081652176 | 0.903252684 | 26S protease regulatory subunit 4 |
| CNXL_032160 | CNE03040 | 0.312130605 | 0.430596525 | ubiquitin-conjugating enzyme E2 G2 |
| CNXL_032170 | CNE03050 | 0.198513819 | 0.696473798 | actin cortical patch component |
| CNXL_032180 | | -0.12403577 | 0.906149032 | type 2a-like serine/threonine-protein phosphatase |
| CNXL_032190 | CNE03060 | -0.156689534 | 0.731942196 | hypothetical protein |
| CNXL_032200 | CNE03070 | -0.505621418 | 0.125429289 | large subunit ribosomal protein L6e |
| CNXL_032210 | CNE03080 | 0.238223617 | 0.626204081 | serine/threonine-protein kinase ATR |
| CNXL_032220 | CNE03090 | 0.307358295 | 0.484524029 | RNA polymerase II-associated factor 1 |
| CNXL_032230 | CNE03100 | 1.448141309 | 0.113351667 | cytosolic Fe-S cluster assembly factor NBP35 |
| CNXL_032240 | CNE03110 | -0.750122699 | 0.007855444 | phosphoketolase |
| CNXL_032250 | | 0.081855344 | 0.903282886 | protein farnesyltransferase alpha subunit |
| CNXL_032260 | CNE03120 | 0.092962233 | 0.885449489 | hypothetical protein |
| CNXL_032270 | | 0.043530264 | 0.964414864 | hypothetical protein |
| CNXL_032280 | CNE03130 | 0.443396724 | 0.217965571 | hypothetical protein |
| CNXL_032290 | CNE03140 | 0.671453232 | 0.014226241 | hypothetical protein |
| CNXL_032300 | CNE03150 | -0.747706764 | 0.017199183 | hypothetical protein |
| CNXL_032310 | CNE03160 | 0.690924793 | 0.028184441 | glucan 1 |
| CNXL_032320 | CNE03170 | -0.150059169 | 0.794943165 | hypothetical protein |
| CNXL_032330 | CNE03180 | -0.043315231 | 0.963190895 | signal sequence binding protein |
| CNXL_032340 | CNE03190 | -0.121898528 | 0.849436887 | phosphopantothenate-cysteine ligase |
| CNXL_032350 | CNE03200 | -0.342798865 | 0.323308793 | zinc ion transporter |
| CNXL_032360 | | -2.269731231 | NA | hypothetical protein |
| CNXL_032370 | CNE03210 | -0.942856932 | NA | Unknown |
| CNXL_032380 | CNE03220 | 0.565394144 | 0.067802501 | Unknown |
| CNXL_032390 | | -0.128664405 | 0.874670769 | hypothetical protein |
| CNXL_032400 | CNE03240 | -1.575217353 | 1.8666E-09 | Unknown |
| CNXL_032410 | CNE03250 | 0.045873571 | 0.949958757 | putative chitin synthase |
| CNXL_032420 | CNE03260 | -0.097932613 | 0.906875136 | hypothetical protein |
| CNXL_032430 | CNE03270 | -0.228075435 | 0.69536267 | CCAAT -binding transcription factor |
| CNXL_032440 | CNE03280 | 0.215853739 | 0.634728039 | ubiquitin conjugating enzyme |
| CNXL_032450 | CNE03290 | -0.733612953 | 0.03134473 | epsin |
| CNXL_032460 | CNE03300 | 0.328223212 | 0.534813127 | translation initiation factor 3 subunit A |

| | | | | |
|---|---|---|---|---|
| CNXL_032470 | CNE03310 | 0.774991408 | 0.010312142 | hypothetical protein |
| CNXL_032480 | CNE03320 | -0.278502649 | 0.532843849 | ribonuclease Z |
| CNXL_032490 | CNE03330 | -0.845132625 | 0.003855762 | nucleolar protein 56 |
| CNXL_032500 | CNE03340 | -0.520821621 | 0.164821847 | ribonucleoside-diphosphate reductase large subunit |
| CNXL_032510 | | 0.528730306 | 0.8610985 | hypothetical protein |
| CNXL_032520 | CNE03350 | 0.787267329 | 0.078650246 | hypothetical protein |
| CNXL_032530 | CNE03360 | -0.113514717 | 0.901676457 | pH-response regulator protein palC |
| CNXL_032540 | CNE03370 | -0.237886521 | 0.61372863 | endonuclease G |
| CNXL_032550 | CNE03380 | -0.326967564 | 0.500750138 | hypothetical protein |
| CNXL_032560 | CNE03390 | -0.066314753 | 0.949641665 | adenylylsulfate kinase |
| CNXL_032570 | CNE03400 | -0.669848961 | 0.058091765 | hypothetical protein |
| CNXL_032580 | CNE03410 | -0.197869546 | 0.771763303 | hypothetical protein |
| CNXL_032590 | CNE03420 | 0.159569395 | 0.782173947 | mitochondrial inner membrane protease ATP23 |
| CNXL_032600 | CNE03430 | -1.161386945 | 4.95107E-05 | hypothetical protein |
| CNXL_032610 | CNE03440 | -0.482217609 | 0.552790986 | septin |
| CNXL_032620 | CNE03450 | -0.980343328 | 0.051851032 | origin recognition complex subunit 1 |
| CNXL_032630 | CNE03460 | 0.149803588 | 0.814220979 | AGC/NDR protein kinase |
| CNXL_032640 | | -0.114090451 | 0.919952257 | kinetochore protein Spc25 |
| CNXL_032650 | | 0.016835801 | 0.988641003 | hypothetical protein |
| CNXL_032660 | CNE03470 | -0.066136467 | 0.936507583 | hypothetical protein |
| CNXL_032670 | CNE03480 | -0.507797775 | 0.139492286 | hypothetical protein |
| CNXL_032680 | CNE03490 | -0.032144601 | 0.965966884 | alpha-amylase |
| CNXL_032690 | CNE03500 | 0.037247348 | 0.965966884 | hypothetical protein |
| CNXL_032700 | CNE03510 | 0.336133556 | 0.52420732 | terbinafine resistance locus protein |
| CNXL_032710 | CNE03520 | 0.283487409 | 0.469904784 | myosin regulatory light chain |
| CNXL_032720 | | -4.246664263 | 1.0611E-53 | nuclear protein |
| CNXL_032730 | CNE03530 | 0.131675876 | 0.803577282 | hypothetical protein |
| CNXL_032740 | CNE03540 | 2.41783686 | 0.056145687 | anaphase-promoting complex subunit 1 |
| CNXL_032750 | CNE03550 | -0.184538287 | 0.689555844 | D-lactaldehyde dehydrogenase |
| CNXL_032760 | CNE03560 | 0.017222821 | 0.986381348 | Unknown |
| CNXL_032770 | CNE03570 | -0.337068095 | 0.499737894 | dihydrokaempferol 4-reductase |
| CNXL_032780 | CNE03590 | 0.173248248 | 0.742187954 | phosphate transporter |
| CNXL_032790 | CNE03600 | 0.142535724 | 0.806904085 | hemolysin |
| CNXL_032800 | CNE03610 | 0.090063288 | 0.892724552 | hypothetical protein |
| CNXL_032810 | CNE03620 | -0.12823661 | 0.809974479 | hypothetical protein |
| CNXL_032820 | CNE03630 | -0.269109326 | 0.529911907 | 5'-3' exoribonuclease 1 |
| CNXL_032830 | CNE03640 | 0.122087167 | 0.865068963 | signal recognition particle protein SRP54 |
| CNXL_032840 | CNE03650 | -0.33636231 | 0.495942837 | transporter particle component |
| CNXL_032850 | CNE03660 | 0.185291285 | 0.761026029 | ubiquitin-protein ligase |
| CNXL_032860 | CNE03670 | 1.087071734 | 0.003502057 | DNA polymerase eta subunit |
| CNXL_032870 | CNE03700 | 0.550817196 | 0.156465695 | hypothetical protein |
| CNXL_032880 | CNE03710 | -0.105578015 | 0.902969744 | glucoamylase |
| CNXL_032890 | CNE03720 | -0.26676818 | 0.535382959 | vacuolar protein sorting-associated protein 27 |
| CNXL_032900 | CNE03730 | 0.483584397 | 0.202621105 | DNA-directed RNA polymerase II subunit RPB1 |
| CNXL_032910 | CNE03740 | -0.127016466 | 0.81790448 | cytoplasmic protein |
| CNXL_032920 | CNE03750 | -0.782131835 | 0.5737945 | hypothetical protein |
| CNXL_032930 | CNE03760 | -0.173205848 | 0.768388475 | hypothetical protein |
| CNXL_032940 | CNE03770 | -0.236838905 | 0.638378602 | U3 small nucleolar RNA-associated protein 24 |
| CNXL_032950 | CNE03780 | 0.117352129 | 0.893628894 | cortical actin cytoskeleton protein asp1 |
| CNXL_032960 | | -0.105280543 | 0.948457186 | hypothetical protein |
| CNXL_032970 | CNE03790 | -0.361885024 | 0.384549545 | Unknown |
| CNXL_032980 | CNE03800 | -0.00388227 | 0.996158838 | voltage-gated chloride channel protein |
| CNXL_032990 | CNE03810 | -0.195119847 | 0.752736444 | hypothetical protein |
| CNXL_033000 | CNE03820 | -0.578005548 | 0.384549545 | hypothetical protein |
| CNXL_033010 | CNE03830 | -0.095610985 | 0.873249995 | anaphase-promoting complex subunit 6 |
| CNXL_033020 | CNE03840 | -0.460899178 | 0.372306093 | general transcriptional repressor |
| CNXL_033030 | | -0.398096599 | 0.32259991 | hypothetical protein |
| CNXL_033040 | CNE03860 | 0.158779475 | 0.817410012 | amidohydrolase |

| | | | | |
|---|---|---|---|---|
| CNXL_033050 | CNE03870 | 0.031696802 | 0.96580389 | mRNA methyltransferase |
| CNXL_033060 | CNE03880 | -0.016547667 | 0.985173026 | hypothetical protein |
| CNXL_033070 | CNE03890 | -0.077021707 | 0.904038129 | ubiquitin-conjugating enzyme E2 35 |
| CNXL_033080 | CNE03900 | 0.645475359 | 0.107812691 | cytochrome c peroxidase |
| CNXL_033090 | CNE03910 | -0.083778573 | 0.9003865 | hypothetical protein |
| CNXL_033100 | CNE03920 | -0.147500649 | 0.757739987 | hypothetical protein |
| CNXL_033110 | | 0.044162396 | 0.954346886 | large subunit ribosomal protein L1-A |
| CNXL_033120 | | 0.236818881 | 0.729415108 | hypothetical protein |
| CNXL_033130 | CNE03960 | -0.006844809 | 0.992176872 | hypothetical protein |
| CNXL_033140 | CNE03970 | 0.204500893 | 0.689987618 | NADH dehydrogenase |
| CNXL_033150 | CNE03980 | -0.286166805 | 0.576086926 | oxysterol-binding protein |
| CNXL_033160 | CNE03990 | -0.229511956 | 0.647364118 | DNA replication ATP-dependent helicase Dna2 |
| CNXL_033170 | CNE04000 | -0.172854148 | 0.748690308 | hypothetical protein |
| CNXL_033180 | CNE04010 | 0.503789298 | 0.194911786 | small subunit ribosomal protein S6 |
| CNXL_033190 | CNE04020 | 0.055285951 | 0.936783812 | protein transporter SEC20 |
| CNXL_033200 | CNE04030 | 0.927223117 | 0.0039568 | rsc chromatin remodeling complex subunit |
| CNXL_033210 | CNE04040 | 0.181064822 | 0.801741833 | 6-phosphogluconolactonase |
| CNXL_033220 | CNE04050 | 0.264168915 | 0.615328522 | hypothetical protein |
| CNXL_033230 | NE04060\|CNM004 | 0.365596653 | 0.302711288 | iron-sulfur cluster assembly protein |
| CNXL_033240 | | -0.266410619 | 0.634728039 | Unknown |
| CNXL_033250 | CNE04080 | 1.707049686 | 0.117352703 | Unknown |
| CNXL_033260 | CNE04090 | -0.231988182 | 0.576230883 | hypothetical protein |
| CNXL_033270 | CNE04100 | 0.148058408 | 0.810102246 | translation initiation factor 3 subunit J |
| CNXL_033280 | CNE04110 | 0.154293007 | 0.782518595 | cyclin-dependent protein kinase regulator |
| CNXL_033290 | CNE04120 | -1.181828032 | 2.49409E-05 | hypothetical protein |
| CNXL_033300 | CNE04130 | 0.628328408 | 0.126171266 | hypothetical protein |
| CNXL_033310 | CNE04140 | -0.015324423 | 0.990777629 | hypothetical protein |
| CNXL_033320 | CNE04150 | -0.081771312 | 0.903834486 | methionyl-tRNA formyltransferase |
| CNXL_033330 | CNE04160 | 0.005835116 | 0.994731938 | cytoplasmic tRNA 2-thiolation protein 1 |
| CNXL_033340 | CNE04170 | 0.405983546 | 0.336457352 | hypothetical protein |
| CNXL_033350 | CNE04180 | 0.198724406 | 0.723317464 | TIGR01456 family HAD hydrolase |
| CNXL_033360 | CNE04190 | 1.711763338 | 8.34069E-10 | hypothetical protein |
| CNXL_033370 | CNE04200 | 0.015048058 | 0.986747477 | hypothetical protein |
| CNXL_033380 | CNE04210 | -0.269650019 | 0.593029492 | NAD-binding Rossmann fold oxidoreductase |
| CNXL_033390 | CNE04220 | 0.022705685 | 0.975873016 | arf/Sar family protein |
| CNXL_033400 | CNE04230 | -0.342748228 | 0.540948602 | nonhistone chromosomal protein |
| CNXL_033410 | CNE04240 | 0.110021935 | 0.852076639 | hypothetical protein |
| CNXL_033420 | CNE04250 | -0.327686336 | 0.44895554 | clathrin assembly protein |
| CNXL_033430 | CNE04260 | -0.042000477 | 0.956449152 | ubiquitin-conjugating enzyme E2 W |
| CNXL_033440 | CNE04270 | 0.041128113 | 0.953095191 | protein Clp1 |
| CNXL_033450 | CNE04280 | 0.096725115 | 0.890147007 | peptide alpha-N-acetyltransferase |
| CNXL_033460 | CNE04290 | 0.026476906 | 0.97922031 | type I inositol-1 |
| CNXL_033470 | CNE04300 | 1.151419685 | 0.00059673 | GTPase activating protein |
| CNXL_033480 | CNE04310 | -0.015867454 | 0.987014445 | hypothetical protein |
| CNXL_033490 | | 0.132859679 | 0.885449489 | hypothetical protein |
| CNXL_033500 | CNE04330 | -0.089124583 | 0.884028948 | methylated-DNA-protein-cysteine methyltransferase |
| CNXL_033510 | CNE04340 | 0.412681655 | 0.462811802 | hypothetical protein |
| CNXL_033520 | CNE04350 | -0.133719883 | 0.837479784 | hypothetical protein |
| CNXL_033530 | CNE04360 | -0.462551524 | 0.188907193 | hypothetical protein |
| CNXL_033540 | CNE04370 | -0.415039418 | 0.271362906 | fatty acid synthase complex protein |
| CNXL_033550 | CNE04380 | 0.409417698 | 0.354001758 | fatty acid synthase beta subunit |
| CNXL_033560 | CNE04390 | -0.03009616 | 0.981240834 | hypothetical protein |
| CNXL_033570 | CNE04400 | -1.325423057 | 1.30012E-05 | peroxin-16 |
| CNXL_033580 | CNE04410 | 0.057771616 | 0.932840267 | cyclin |
| CNXL_033590 | | -0.549001362 | 0.253865879 | arp2/3 complex 34 kda subunit |
| CNXL_033600 | | -0.618792868 | 0.35455944 | hypothetical protein |
| CNXL_033610 | CNE04430 | -0.001324391 | 0.997686984 | Unknown |
| CNXL_033620 | CNE04440 | 0.010046551 | 0.990777629 | protein EFR3 |

| | | | | |
|---|---|---|---|---|
| CNXL_033630 | CNE04450 | -0.48976279 | 0.173210496 | nucleosome assembly protein 1-like 1 |
| CNXL_033640 | CNE04460 | 0.39265836 | 0.301417944 | hypothetical protein |
| CNXL_033650 | CNE04470 | -0.189743805 | 0.717991689 | hypothetical protein |
| CNXL_033660 | CNE04480 | -0.937185493 | 0.002001095 | hypothetical protein |
| CNXL_033670 | CNE04490 | -0.034236025 | 0.964076726 | acyl-CoA-dependent ceramide synthase |
| CNXL_033680 | CNE04500 | 0.679419606 | 0.029042446 | acyl-CoA-dependent ceramide synthase |
| CNXL_033690 | CNE04510 | -0.291332119 | 0.522860075 | histone deacetylase |
| CNXL_033700 | | -0.023189311 | 0.986226596 | farnesyl pyrophosphate synthetase |
| CNXL_033710 | CNE04520 | 1.421886964 | 8.20017E-05 | Unknown |
| CNXL_033720 | CNE04530 | 0.403532553 | 0.345963301 | hypothetical protein |
| CNXL_033730 | CNE04540 | -0.012198167 | 0.990777629 | siderochrome-iron transporter |
| CNXL_033740 | CNE04550 | 0.063906553 | 0.926879789 | DASH complex subunit DAD1 |
| CNXL_033750 | CNE04560 | 0.387919119 | 0.43363696 | mannosyl-oligosaccharide alpha-1 |
| CNXL_033760 | CNE04580 | 0.07307904 | 0.911130835 | hypothetical protein |
| CNXL_033770 | CNE04590 | -0.446945422 | 0.264480588 | cytoplasmic protein |
| CNXL_033780 | | -0.459617537 | 0.586181879 | hydrolase |
| CNXL_033790 | CNE04600 | -0.119877123 | 0.815757652 | elongator complex protein 5 |
| CNXL_033800 | CNE04610 | 0.216989538 | 0.73673899 | leukotriene A-4 hydrolase/aminopeptidase |
| CNXL_033810 | CNE04620 | 0.017383478 | 0.985803294 | hypothetical protein |
| CNXL_033820 | CNE04630 | 0.757867744 | 0.010969003 | mitochondrial processing peptidase |
| CNXL_033830 | CNE04640 | 0.997224641 | 0.00086093 | DNA mismatch repair protein MLH1 |
| CNXL_033840 | CNE04650 | 0.050252512 | 0.946605516 | hypothetical protein |
| CNXL_033850 | CNE04660 | 1.268479857 | 0.197592932 | putative scaffold protein |
| CNXL_033860 | CNE04670 | 1.247637803 | 2.76946E-05 | hypothetical protein |
| CNXL_033870 | CNE04680 | -0.256880212 | 0.756597645 | hypothetical protein |
| CNXL_033880 | CNE04690 | -0.16181786 | 0.746765994 | stromal membrane-associated protein |
| CNXL_033890 | | 0.117335878 | 0.925141393 | vacuolar protein |
| CNXL_033900 | CNE04700 | -0.751579033 | 0.110223992 | hypothetical protein |
| CNXL_033910 | CNE04710 | -0.133741487 | 0.809974479 | hypothetical protein |
| CNXL_033920 | CNE04720 | 0.491012676 | 0.270697928 | TATA-box-binding protein-associated factor |
| CNXL_033930 | CNE04730 | -0.344659029 | 0.490315559 | hypothetical protein |
| CNXL_033940 | CNE04740 | 0.585268826 | 0.063702758 | glycoside hydrolase family 2 |
| CNXL_033950 | CNE04750 | -0.912469086 | 0.17318254 | Unknown |
| CNXL_033960 | CNE04760 | -0.0068479 | 0.992981679 | hypothetical protein |
| CNXL_033970 | CNE04770 | 0.038990266 | 0.965465882 | CCR4-NOT complex subunit CAF16 |
| CNXL_033980 | CNE04780 | -0.035240243 | 0.965465882 | urease accessory protein |
| CNXL_033990 | CNE04790 | 0.888236846 | 0.006807024 | hypothetical protein |
| CNXL_034000 | CNE04800 | -0.210066532 | 0.678131584 | hypothetical protein |
| CNXL_034010 | CNE04810 | -0.503146664 | 0.61517823 | hypothetical protein |
| CNXL_034020 | CNE04820 | 0.274029227 | 0.540001756 | hypothetical protein |
| CNXL_034030 | CNE04830 | -0.055680703 | 0.964414864 | dimeric dihydrodiol dehydrogenase |
| CNXL_034040 | CNE04840 | 0.175665896 | 0.789827438 | kinesin family member 20/23 |
| CNXL_034050 | CNE04860 | 0.587130554 | 0.049978018 | MFS multidrug transporter |
| CNXL_034060 | CNE04870 | -0.350129225 | 0.510006134 | proline oxidase |
| CNXL_034070 | CNE04890 | 0.222678449 | 0.61372863 | proline oxidase |
| CNXL_034080 | CNE04900 | -0.039385868 | 0.963420993 | poly |
| CNXL_034090 | CNE04910 | 0.385017552 | 0.273415774 | acetoacetate-CoA ligase |
| CNXL_034100 | | 0.311614461 | 0.544657787 | hypothetical protein |
| CNXL_034110 | | 0.028134446 | 0.965465882 | hypothetical protein |
| CNXL_034120 | CNE04930 | 1.264528964 | 9.34812E-05 | Unknown |
| CNXL_034130 | CNE04940 | -1.187074207 | 9.36446E-06 | Unknown |
| CNXL_034140 | | 0.776790628 | 0.388091044 | hypothetical protein |
| CNXL_034150 | CNE04950 | 0.110311707 | 0.930990489 | Unknown |
| CNXL_034160 | CNE04960 | 0.070052718 | 0.930994697 | hypothetical protein |
| CNXL_034170 | CNE04970 | -0.12876226 | 0.820111382 | integral membrane protein |
| CNXL_034180 | CNE04980 | -0.382247257 | 0.423141864 | hypothetical protein |
| CNXL_034190 | CNE04990 | -0.531487687 | 0.146688128 | large subunit ribosomal protein L22 |
| CNXL_034200 | CNE05000 | 0.580300513 | 0.063015081 | putative sugar transporter |

| | | | | |
|---|---|---|---|---|
| CNXL_034210 | CNE05010 | 0.319181383 | 0.555395578 | triose-phosphate isomerase |
| CNXL_034220 | | -0.392767142 | 0.432050869 | hypothetical protein |
| CNXL_034230 | CNE05030 | -0.109734206 | 0.885370637 | Unknown |
| CNXL_034240 | | -0.434396086 | 0.837168643 | nuclear export factor |
| CNXL_034250 | | 0.292952326 | 0.643890318 | Unknown |
| CNXL_034260 | CNE05040 | -0.190243802 | 0.716771972 | Unknown |
| CNXL_034270 | CNE05050 | 0.323099012 | 0.523679914 | glyoxal oxidase |
| CNXL_034280 | CNE05060 | -0.267723277 | 0.554068099 | Wiskott-Aldrich syndrome protein |
| CNXL_034290 | CNE05070 | -0.684115598 | 0.081569132 | CMGC/SRPK protein kinase |
| CNXL_034300 | CNE05080 | -0.410260401 | 0.305872929 | hypothetical protein |
| CNXL_034310 | CNE05090 | -0.173963132 | 0.731955533 | hypothetical protein |
| CNXL_034320 | CNE05100 | -0.722554296 | 0.085666549 | DNA-directed RNA polymerase III subunit RPC3 |
| CNXL_034330 | CNE05110 | 0.13795549 | 0.84689626 | hypothetical protein |
| CNXL_034340 | CNE05120 | -0.452701053 | 0.308605515 | hypothetical protein |
| CNXL_034350 | CNE05130 | 0.018904644 | 0.984913368 | DNA-directed RNA polymerase II subunit RPB3 |
| CNXL_034360 | CNE05140 | 0.4183236 | 0.308626777 | hypothetical protein |
| CNXL_034370 | | 0.1828945 | 0.810224824 | SWR1-complex protein 4 |
| CNXL_034380 | CNE05150 | 0.147646732 | 0.825693641 | Unknown |
| CNXL_034390 | CNE05160 | 0.465716871 | 0.213413887 | DEAD-box ATP-dependent RNA helicase 26 |
| CNXL_034400 | CNE05170 | -0.107791475 | 0.84689626 | hypothetical protein |
| CNXL_034410 | CNE05180 | 0.503693015 | 0.165493103 | beta-glucosidase |
| CNXL_034420 | | 0.131592356 | 0.842469317 | sugar transporter |
| CNXL_034430 | | -0.252093734 | 0.682914878 | hypothetical protein |
| CNXL_034440 | NE00070\|CNE0522 | -0.950931835 | NA | Unknown |
| CNXL_034450 | | 1.935408927 | NA | translation machinery-associated protein 16 |
| CNXL_034460 | NE00040\|CNE0525 | 1.898863009 | 0.420406127 | Unknown |
| CNXL_034470 | NE00020\|CNE0526 | 0 | NA | phosphopyruvate hydratase |
| CNXL_034480 | CNE05290 | 0 | NA | HPP family protein |
| CNXL_034490 | CNE05300 | 0 | NA | hypothetical protein |
| CNXL_034500 | CNE05310 | 0 | NA | hypothetical protein |
| CNXL_034510 | CNE05330 | 0 | NA | 5-oxoprolinase |
| CNXL_034520 | CNE05340 | 0 | NA | HpcH/HpaI aldolase/citrate lyase |
| CNXL_034530 | CNE05350 | 0 | NA | allantoate permease |
| CNXL_034540 | CNE05360 | 0 | NA | alpha-ketoglutarate-dependent taurine dioxygenase |
| CNXL_034550 | | NA | NA | Unknown |
| CNXL_034560 | CNF00010 | -0.053707474 | 0.963420993 | Unknown |
| CNXL_034570 | CNF00020 | -1.099784631 | 0.000187618 | Unknown |
| CNXL_034580 | | -0.258524146 | 0.621585258 | MFS transporter |
| CNXL_034590 | CNF00040 | -0.004691583 | 0.995735232 | dynein light chain roadblock-type |
| CNXL_034600 | CNF00050 | -0.629171066 | 0.314390734 | large subunit ribosomal protein L7/L12 |
| CNXL_034610 | CNF00070 | -0.385177686 | 0.28631663 | phosphoglycerate dehydrogenase |
| CNXL_034620 | CNF00080 | 0.11861516 | 0.825693641 | glutamate-tRNA ligase |
| CNXL_034630 | CNF00090 | -0.16964459 | 0.743024901 | proteasome activator subunit 4 |
| CNXL_034640 | CNF00100 | -0.255814543 | 0.598241347 | nucleolar protein 58 |
| CNXL_034650 | | -0.476498059 | 0.810260352 | ribonuclease Z |
| CNXL_034660 | | -1.86093521 | 0.002644676 | Unknown |
| CNXL_034670 | CNF00120 | 0.015581199 | 0.985595171 | Unknown |
| CNXL_034680 | | -1.166158703 | 0.17859659 | serine carboxypeptidase |
| CNXL_034690 | CNF00130 | 0.72433233 | 0.010506901 | hypothetical protein |
| CNXL_034700 | CNF00140 | -0.326938458 | 0.46380108 | Unknown |
| CNXL_034710 | CNF00150 | -1.248559253 | 0.01539502 | ste/ste20/paka protein kinase |
| CNXL_034720 | CNF00160 | -0.918139244 | 0.010312142 | O-sialoglycoprotein endopeptidase |
| CNXL_034730 | | -0.008233795 | 0.995735232 | Unknown |
| CNXL_034740 | CNF00170 | 0.353386481 | 0.488037843 | hypothetical protein |
| CNXL_034750 | CNF00180 | -0.272239592 | 0.748357146 | hypothetical protein |
| CNXL_034760 | CNF00190 | -0.234823963 | 0.714943777 | poly |
| CNXL_034770 | | -0.690645894 | 0.50023141 | hypothetical protein |
| CNXL_034780 | | 0.255453646 | 0.825693641 | hypothetical protein |

| | | | | |
|---|---|---|---|---|
| CNXL_034790 | CNF00200 | 0.235588148 | 0.678207179 | Unknown |
| CNXL_034800 | CNF00210 | -0.124560495 | 0.830440343 | hypothetical protein |
| CNXL_034810 | CNF00220 | 0.141744671 | 0.932840267 | putative copper ion transporter |
| CNXL_034820 | CNF00230 | -2.878580615 | 3.10962E-30 | hypothetical protein |
| CNXL_034830 | CNM02090 | 0.254116347 | 0.884028948 | zinc finger family protein |
| CNXL_034840 | | -0.530600055 | 0.75185116 | Unknown |
| CNXL_034850 | CNF00240 | 0.307859491 | 0.864880565 | Unknown |
| CNXL_034860 | CNF00250 | 0.276713751 | 0.654994028 | hypothetical protein |
| CNXL_034870 | CNF00260 | -0.263037634 | 0.677367265 | Unknown |
| CNXL_034880 | CNF00270 | 0.180570395 | 0.771239252 | pre-mRNA-splicing factor CWC22 |
| CNXL_034890 | CNF00280 | -0.730561737 | 0.092504567 | hypothetical protein |
| CNXL_034900 | CNF00290 | -0.028946439 | 0.972638293 | hypothetical protein |
| CNXL_034910 | CNF00300 | 0.022872946 | 0.983499434 | nucleoside phosphatase |
| CNXL_034920 | CNF00310 | -0.108051594 | 0.897217254 | hypothetical protein |
| CNXL_034930 | CNF00320 | -0.504551312 | 0.190652363 | high-affinity methionine permease |
| CNXL_034940 | CNF00330 | 0.579121863 | 0.030337262 | nicotinate |
| CNXL_034950 | CNF00340 | -0.658849886 | 0.10562276 | gamma-aminobutyric acid transporter |
| CNXL_034960 | CNF00350 | 0.150790084 | 0.770532174 | topoisomerase 1-associated factor 1 |
| CNXL_034970 | CNF00360 | 0.404882894 | 0.423141864 | Unknown |
| CNXL_034980 | | 0.431344309 | 0.597848423 | UMF1 family MFS transporter |
| CNXL_034990 | | 1.263026565 | 5.93592E-05 | Unknown |
| CNXL_035000 | CNF00370 | -0.337919623 | 0.366888453 | Unknown |
| CNXL_035010 | CNF00380 | 0.718895666 | 0.133565651 | hypothetical protein |
| CNXL_035020 | CNF00390 | -0.087464363 | 0.884028948 | hypothetical protein |
| CNXL_035030 | CNF00400 | -0.370608847 | 0.405796614 | DNA-directed RNA polymerase III subunit RPC1 |
| CNXL_035040 | CNF00410 | -0.477887503 | 0.25037723 | UMP-CMP kinase |
| CNXL_035050 | CNF00420 | -0.906445294 | 0.002078997 | translin domain protein |
| CNXL_035060 | CNF00430 | -0.891662351 | 0.004632716 | hypothetical protein |
| CNXL_035070 | CNF00440 | 0.391996503 | 0.253865879 | peptidyl-prolyl cis-trans isomerase D |
| CNXL_035080 | CNF00450 | 0.330832445 | 0.331351174 | protein BTN1 |
| CNXL_035090 | CNF00470 | 0.488258287 | 0.308473689 | hypothetical protein |
| CNXL_035100 | CNF00480 | -0.30875458 | 0.625150196 | hypothetical protein |
| CNXL_035110 | CNF00490 | 0.115765886 | 0.856084571 | hypothetical protein |
| CNXL_035120 | CNF00500 | -0.575586479 | 0.068372808 | U2 small nuclear ribonucleoprotein A' |
| CNXL_035130 | CNF00510 | -0.920213421 | 0.015979048 | septin ring protein |
| CNXL_035140 | | -0.167460215 | 0.810102246 | hypothetical protein |
| CNXL_035150 | | 0.536019908 | 0.091985509 | hypothetical protein |
| CNXL_035160 | CNF00550 | 0.122455149 | 0.861981252 | 2-acylglycerol O-acyltransferase 2 |
| CNXL_035170 | CNF00560 | 0.200378822 | 0.766858347 | tRNA-specific adenosine deaminase 2 |
| CNXL_035180 | CNF00570 | -0.374837291 | 0.415298045 | hypothetical protein |
| CNXL_035190 | CNF00580 | -0.182579613 | 0.771763303 | ATP synthase subunit beta |
| CNXL_035200 | | 0.352717248 | 0.616861062 | hypothetical protein |
| CNXL_035210 | CNF00590 | 0.844780216 | 0.016679695 | hypothetical protein |
| CNXL_035220 | CNF00600 | -0.667841474 | 0.023966976 | hypothetical protein |
| CNXL_035230 | CNF00610 | -0.825484504 | 0.008572222 | Unknown |
| CNXL_035240 | CNF00620 | -0.007776989 | 0.992176872 | alpha-glucosidase |
| CNXL_035250 | CNF00630 | -0.398581148 | 0.326055975 | hypothetical protein |
| CNXL_035260 | CNF00640 | -0.380479597 | 0.550659282 | cytochrome c1 |
| CNXL_035270 | CNF00650 | -0.151222686 | 0.796092658 | hypothetical protein |
| CNXL_035280 | | -0.269227767 | 0.626496712 | pyruvate carboxylase |
| CNXL_035290 | CNF00660 | -0.06571622 | 0.919196787 | hypothetical protein |
| CNXL_035300 | CNF00670 | -0.164196404 | 0.718774576 | transcription factor IIIB 90 kDa subunit |
| CNXL_035310 | CNF00680 | 0.701674319 | 0.063292368 | small subunit ribosomal protein S14 |
| CNXL_035320 | CNF00710 | 0.395544503 | 0.365237105 | hypothetical protein |
| CNXL_035330 | CNF00720 | 0.095606469 | 0.90461084 | AP-3 complex subunit sigma |
| CNXL_035340 | CNF00730 | -0.09568834 | 0.874670769 | hypothetical protein |
| CNXL_035350 | CNF00740 | -0.161923603 | 0.825383092 | glycine-tRNA ligase |
| CNXL_035360 | CNF00750 | -0.163275375 | 0.791528992 | pyrroline-5-carboxylate reductase |

| | | | | |
|---|---|---|---|---|
| CNXL_035370 | CNF00760 | 0.103852239 | 0.919952257 | hypothetical protein |
| CNXL_035380 | CNF00770 | 0.009204478 | 0.992176872 | hypothetical protein |
| CNXL_035390 | CNF00780 | -0.313796083 | 0.492996269 | ubiquitin-activating enzyme e1-like |
| CNXL_035400 | CNF00790 | -0.462862355 | 0.179645479 | protein CFT1 |
| CNXL_035410 | CNF00800 | -0.85236596 | 0.032580388 | dynein heavy chain 1 |
| CNXL_035420 | CNF00810 | -0.147308327 | 0.802877744 | hypothetical protein |
| CNXL_035430 | CNF00820 | 0.651610377 | 0.077611959 | phosphoglycerate mutase |
| CNXL_035440 | CNF00825 | -0.337235249 | 0.45976707 | TDG/mug DNA glycosylase |
| CNXL_035450 | CNF00830 | 0.175948514 | 0.747332879 | hypothetical protein |
| CNXL_035460 | CNF00850 | 0.256685746 | 0.496574337 | hypothetical protein |
| CNXL_035470 | CNF00860 | 0.041865645 | 0.955507961 | Unknown |
| CNXL_035480 | CNF00870 | 0.002749814 | 0.996374193 | pre-mRNA-splicing factor 18 |
| CNXL_035490 | CNF00880 | -1.231693337 | 9.80723E-05 | ubiquitin-conjugating enzyme |
| CNXL_035500 | CNF00890 | -1.055002485 | 0.002114604 | hypothetical protein |
| CNXL_035510 | CNF00900 | -0.314527361 | 0.535369833 | importin beta-4 subunit |
| CNXL_035520 | | -0.129680924 | 0.869120128 | hypothetical protein |
| CNXL_035530 | CNF00910 | -0.149294862 | 0.827849849 | CCAAT t-binding transcription factor |
| CNXL_035540 | CNF00920 | 0.161174757 | 0.75342527 | class e vacuolar protein-sorting machinery protein |
| CNXL_035550 | CNF00930 | 0.218791642 | 0.730539927 | alpha-1 |
| CNXL_035560 | | -0.412963256 | 0.918949484 | hypothetical protein |
| CNXL_035570 | CNF00940 | 0.314674981 | 0.425879761 | hypothetical protein |
| CNXL_035580 | CNF00950 | 0.129401214 | 0.849074517 | hypothetical protein |
| CNXL_035590 | CNF00960 | 0.499789392 | 0.475345003 | CDK-activating kinase assembly factor MAT1 |
| CNXL_035600 | CNF00970 | -0.229690573 | 0.771763303 | hypothetical protein |
| CNXL_035610 | CNF00980 | -0.991719441 | 0.000670496 | cytochrome c heme-lyase |
| CNXL_035620 | CNF00990 | -0.169082589 | 0.771763303 | hypothetical protein |
| CNXL_035630 | CNF01000 | 0.351451697 | 0.424157538 | hypothetical protein |
| CNXL_035640 | | 0.255029068 | 0.778681483 | endopeptidase |
| CNXL_035650 | | 0.040823502 | 0.964414864 | Unknown |
| CNXL_035660 | CNF01020 | 0.912405539 | 0.011361987 | Unknown |
| CNXL_035670 | CNF01030 | 1.172038533 | 0.000110022 | hypothetical protein |
| CNXL_035680 | CNF01040 | -0.687272157 | 0.529724345 | hypothetical protein |
| CNXL_035690 | CNF01050 | 0.306184406 | 0.660663885 | endopeptidase |
| CNXL_035700 | CNF01060 | 0.630564578 | 0.067001284 | Unknown |
| CNXL_035710 | CNF01070 | -3.222040688 | 4.06967E-31 | L-fucose transporter |
| CNXL_035720 | CNF01080 | 0.315196223 | 0.500910612 | putative plasma membrane fusion protein |
| CNXL_035730 | CNF01090 | -0.186383134 | 0.789500916 | 20S proteasome subunit beta 6 |
| CNXL_035740 | CNF01100 | 0.054608667 | 0.962194201 | hypothetical protein |
| CNXL_035750 | CNF01110 | 1.019732891 | 0.000562565 | hypothetical protein |
| CNXL_035760 | CNF01120 | -1.138869261 | 6.7285E-06 | DNA excision repair protein ERCC-1 |
| CNXL_035770 | CNF01130 | 0.033750882 | 0.965101267 | hypothetical protein |
| CNXL_035780 | CNF01140 | 0.212948287 | 0.690087697 | sterol O-acyltransferase |
| CNXL_035790 | CNF01150 | -0.675274375 | 0.173210496 | hypothetical protein |
| CNXL_035800 | CNF01160 | 0.000114163 | 0.999593276 | hypothetical protein |
| CNXL_035810 | CNF01170 | -0.242651524 | 0.637722771 | Unknown |
| CNXL_035820 | CNF01180 | -0.686572801 | 0.141210072 | hypothetical protein |
| CNXL_035830 | CNF01190 | -0.406557694 | 0.534447681 | hypothetical protein |
| CNXL_035840 | CNF01200 | -0.142755512 | 0.829317514 | hypothetical protein |
| CNXL_035850 | CNF01210 | -0.432348758 | 0.273759584 | heme oxygenase 2 |
| CNXL_035860 | CNF01220 | -0.451896172 | 0.164354975 | glycerol transporter |
| CNXL_035870 | CNF01230 | -0.904272131 | 0.039674704 | Unknown |
| CNXL_035880 | CNF01240 | -0.351830329 | 0.368884101 | hypothetical protein |
| CNXL_035890 | CNF01250 | -0.344784246 | 0.397276203 | ATP-dependent RNA helicase DBP2-A |
| CNXL_035900 | CNF01260 | -0.731820245 | 0.031829912 | splicing factor U2AF 65 kDa subunit |
| CNXL_035910 | CNF01270 | -0.583671506 | 0.132286922 | thioredoxin reductase |
| CNXL_035920 | CNF01280 | 0.38488596 | 0.236004557 | ubiquitin-conjugating enzyme E2 O |
| CNXL_035930 | CNF01290 | 0.294453451 | 0.53976803 | protein FAM32A |
| CNXL_035940 | CNF01300 | -0.228135668 | 0.676372743 | hypothetical protein |

| | | | | |
|---|---|---|---|---|
| CNXL_035950 | CNF01310 | 0.473306981 | 0.179438445 | translation initiation factor 4E |
| CNXL_035960 | CNF01320 | -0.798687217 | 0.078954247 | cytochrome P450 |
| CNXL_035970 | CNF01330 | 0.686251308 | 0.051200486 | hypothetical protein |
| CNXL_035980 | CNF01340 | -0.099397295 | 0.879688103 | hypothetical protein |
| CNXL_035990 | CNF01350 | -0.341816564 | 0.492021708 | cytochrome c oxidase subunit 6b |
| CNXL_036000 | CNF01360 | -1.503110884 | 0.003609283 | rho GTPase activating protein |
| CNXL_036010 | CNF01370 | 0.018517989 | 0.987958739 | Unknown |
| CNXL_036020 | CNF01380 | 0.093055657 | 0.916472973 | Unknown |
| CNXL_036030 | CNF01390 | -0.068550915 | 0.954346886 | Unknown |
| CNXL_036040 | CNF01400 | -0.131580837 | 0.883856201 | Unknown |
| CNXL_036050 | CNF01410 | 0.312604114 | 0.457691748 | Unknown |
| CNXL_036060 | CNF01420 | 0.027406921 | 0.982102702 | Unknown |
| CNXL_036070 | CNF01430 | 0.409364218 | 0.28631663 | Unknown |
| CNXL_036080 | CNF01440 | -0.416523012 | 0.372306093 | alpha 1 |
| CNXL_036090 | CNF01450 | 0.017006309 | 0.985173026 | wor1/pac2 family transcription factor |
| CNXL_036100 | CNF01460 | -1.762239122 | 3.67873E-13 | hypothetical protein |
| CNXL_036110 | CNF01470 | -3.240999567 | 2.14415E-38 | allantoate transporter |
| CNXL_036120 | CNF01480 | -0.713976323 | 0.015979497 | Unknown |
| CNXL_036130 | CNF01490 | -0.268087373 | 0.601456324 | endoribonuclease |
| CNXL_036140 | CNF01500 | -0.496439074 | 0.213426935 | Unknown |
| CNXL_036150 | CNF01510 | 0.009073415 | 0.991351411 | cytoplasmic protein |
| CNXL_036160 | CNF01520 | 0.253370943 | 0.520208668 | hypothetical protein |
| CNXL_036170 | CNF01530 | -0.428916992 | 0.262390935 | UDP-N-acetylglucosamine pyrophosphorylase |
| CNXL_036180 | CNF01540 | -0.644535212 | 0.105706348 | methionine-tRNA ligase |
| CNXL_036190 | CNF01550 | -0.858008241 | 0.008730468 | translation elongation factor Tu |
| CNXL_036200 | CNF01560 | 0.309819597 | 0.561171887 | minichromosome maintenance protein 7 |
| CNXL_036210 | CNF01570 | 0.266890658 | 0.683642142 | WD-repeat protein 68 |
| CNXL_036220 | CNF01580 | 0.21691362 | 0.734939207 | hypothetical protein |
| CNXL_036230 | | -0.23774398 | 0.885449489 | protein RAI1 |
| CNXL_036240 | CNF01590 | -0.555600085 | 0.112183936 | hypothetical protein |
| CNXL_036250 | CNF01610 | 0.079106803 | 0.918949484 | tryptophan aminotransferase |
| CNXL_036260 | | -0.004468377 | 0.995735232 | putative chitin synthase |
| CNXL_036270 | CNF01620 | -0.048481755 | 0.941187478 | Unknown |
| CNXL_036280 | CNF01630 | -0.071623624 | 0.936086163 | GDP-mannose transporter |
| CNXL_036290 | CNF01640 | 0.533517263 | 0.095784948 | hypothetical protein |
| CNXL_036300 | CNF01650 | -0.155681907 | 0.745210967 | putative beta-glucan synthase |
| CNXL_036310 | CNF01670 | -0.555685502 | 0.076166491 | small subunit ribosomal protein S10e |
| CNXL_036320 | CNF01680 | 0.106770396 | 0.878994922 | potassium:hydrogen antiporter |
| CNXL_036330 | CNF01690 | 0.323546626 | 0.500225679 | hypothetical protein |
| CNXL_036340 | CNF01700 | -0.17073465 | 0.787846251 | hypothetical protein |
| CNXL_036350 | CNF01710 | 0.422460081 | 0.323086046 | ferrochelatase |
| CNXL_036360 | CNF01720 | -0.02238744 | 0.981240834 | syntaxin 18 |
| CNXL_036370 | CNF01730 | -0.011985829 | 0.991351411 | hypothetical protein |
| CNXL_036380 | CNF01740 | 0.403821688 | 0.325787327 | hypothetical protein |
| CNXL_036390 | CNF01750 | -0.226969736 | 0.648500229 | hypothetical protein |
| CNXL_036400 | CNF01760 | -0.264633127 | 0.578501852 | nuclear pore complex protein Nup188 |
| CNXL_036410 | CNF01770 | -0.109285017 | 0.889757615 | exo-beta-1 |
| CNXL_036420 | CNF01780 | -0.114950059 | 0.847311574 | D-aspartate oxidase |
| CNXL_036430 | CNF01790 | -0.058742959 | 0.921547401 | hypothetical protein |
| CNXL_036440 | CNF01800 | -0.890114047 | 0.000199677 | large subunit ribosomal protein L33-b |
| CNXL_036450 | | 0.14582227 | 0.820111382 | chitin deactylase |
| CNXL_036460 | CNF01810 | -0.23248972 | 0.590497102 | signal recognition particle subunit SRP9 |
| CNXL_036470 | CNF01820 | -0.659023746 | 0.05908683 | 5'-3' exoribonuclease |
| CNXL_036480 | CNF01830 | -0.046709355 | 0.950280909 | neurofibromin 1 |
| CNXL_036490 | CNF01840 | -0.85028456 | 0.009965543 | mitogen-activated protein kinase organizer 1 |
| CNXL_036500 | CNF01850 | -0.024656884 | 0.977901553 | ram signaling network protein kinase activator |
| CNXL_036510 | CNF01860 | -0.09323045 | 0.874337207 | hypothetical protein |
| CNXL_036520 | CNF01870 | -0.092731064 | 0.915884007 | 20S proteasome subunit alpha 3 |

| | | | | |
|---|---|---|---|---|
| CNXL_036530 | CNF01880 | 0.062493862 | 0.93433919 | zds-like protein |
| CNXL_036540 | CNF01890 | -0.278297893 | 0.525549979 | OPT family small oligopeptide transporter |
| CNXL_036550 | CNF01900 | 0.11074118 | 0.833392807 | hypothetical protein |
| CNXL_036560 | CNF01910 | -0.252874736 | 0.62317995 | V-type ATPase |
| CNXL_036570 | | -1.299954688 | 7.97017E-06 | symplekin |
| CNXL_036580 | CNF01920 | 0.076944037 | 0.924136135 | hypothetical protein |
| CNXL_036590 | CNF01930 | -0.266714177 | 0.650953538 | Unknown |
| CNXL_036600 | CNF01940 | -0.098889505 | 0.869804835 | putative transcription factor |
| CNXL_036610 | CNF01950 | 0.184118432 | 0.678207179 | hypothetical protein |
| CNXL_036620 | CNF01960 | 0.003251422 | 0.996158838 | transcription initiation factor TFIIB |
| CNXL_036630 | CNF01970 | -0.637124124 | 0.236004557 | mortality factor 4-like protein 1 |
| CNXL_036640 | CNF01990 | 0.027487538 | 0.978689402 | dynein light chain LC8-type |
| CNXL_036650 | CNF02000 | -2.631456928 | 1.03581E-26 | hypothetical protein |
| CNXL_036660 | CNF02010 | 0.602587915 | 0.112183936 | WSC domain-containing protein |
| CNXL_036670 | CNF02020 | 0.202544825 | 0.700912881 | hypothetical protein |
| CNXL_036680 | CNF02030 | 0.023339438 | 0.981240834 | hypothetical protein |
| CNXL_036690 | CNF02040 | 1.125758916 | 0.000589504 | phosphatidylinositol glycan |
| CNXL_036700 | CNF02050 | -0.081244439 | 0.903621195 | hypothetical protein |
| CNXL_036710 | CNF02060 | -0.465044868 | 0.396149193 | F-box and WD-40 domain-containing protein MET30 |
| CNXL_036720 | | -3.353783818 | 0.093403887 | hypothetical protein |
| CNXL_036730 | CNF02070 | -0.416160354 | 0.236155468 | Unknown |
| CNXL_036740 | CNF02080 | -0.074200338 | 0.901676457 | serine/threonine-protein kinase TEL1 |
| CNXL_036750 | | 0.242981525 | 0.580839421 | proteasome component PRE2 |
| CNXL_036760 | CNF02100 | 0.294670945 | 0.482842388 | cytoplasmic protein |
| CNXL_036770 | CNF02110 | 0.046038243 | 0.946722381 | hypothetical protein |
| CNXL_036780 | CNF02120 | 0.085118536 | 0.883172612 | hypothetical protein |
| CNXL_036790 | CNF02130 | -0.153785512 | 0.77424887 | glycosyl hydrolase |
| CNXL_036800 | CNF02140 | -0.264260794 | 0.749050005 | ubiquitin-conjugating enzyme |
| CNXL_036810 | | 0.788603145 | 0.130047497 | small subunit ribosomal protein S5 |
| CNXL_036820 | CNF02150 | -0.061521397 | 0.923572134 | hypothetical protein |
| CNXL_036830 | CNF02160 | 0.289365891 | 0.463658548 | large subunit acidic ribosomal protein P2 |
| CNXL_036840 | | 1.331258888 | 0.193620149 | cyclin-dependent kinase regulatory subunit CKS1 |
| CNXL_036850 | CNF02180 | -0.180473185 | 0.719568426 | Unknown |
| CNXL_036860 | CNF02210 | 0.131491884 | 0.807491917 | acetyl-CoA carboxylase/biotin carboxylase |
| CNXL_036870 | CNF02220 | 0.777714264 | 0.020833973 | Unknown |
| CNXL_036880 | CNF02230 | -0.193420955 | 0.717347987 | mitochondrial protein |
| CNXL_036890 | CNF02240 | -0.148844537 | 0.813698475 | protein MAK16 |
| CNXL_036900 | | -0.174664667 | 0.742187954 | hypothetical protein |
| CNXL_036910 | CNF02250 | -0.09655015 | 0.873602909 | hypothetical protein |
| CNXL_036920 | CNF02260 | -0.727372215 | 0.228258666 | N-acetyl-gamma-glutamyl-phosphate reductase |
| CNXL_036930 | CNF02270 | -0.348701154 | 0.638378602 | kinesin microtuble motor protein |
| CNXL_036940 | CNF02280 | -0.077898991 | 0.884028948 | hypothetical protein |
| CNXL_036950 | CNF02290 | -0.173412771 | 0.864719008 | ATP synthase subunit alpha |
| CNXL_036960 | CNF02300 | 0.209549251 | 0.771763303 | hypothetical protein |
| CNXL_036970 | CNF02310 | 0.022088372 | 0.984913368 | NuA3 HAT complex component NTO1 |
| CNXL_036980 | | -0.930332757 | 0.131084493 | vacuolar protein sorting-associated protein VTA1 |
| CNXL_036990 | CNF02320 | -0.553166802 | 0.107353814 | Swi5-dependent recombination DNA repair protein 1 |
| CNXL_037000 | CNF02330 | -0.085842833 | 0.890150525 | cytoplasmic protein |
| CNXL_037010 | CNF02340 | 0.095156661 | 0.904401612 | 2-dehydropantoate 2-reductase |
| CNXL_037020 | CNF02350 | -0.137101209 | 0.848021859 | anaphase-promoting complex subunit 10 |
| CNXL_037030 | CNF02360 | -0.12810069 | 0.842469317 | putative site-2 protease |
| CNXL_037040 | CNF02370 | -1.624297122 | 8.0478E-12 | hypothetical protein |
| CNXL_037050 | CNF02380 | 0.241255984 | 0.668843965 | protein farnesyltransferase subunit beta |
| CNXL_037060 | | -0.762494706 | 0.521085092 | hypothetical protein |
| CNXL_037070 | CNF02390 | 0.250556781 | 0.623549135 | hypothetical protein |
| CNXL_037080 | | 0.395308201 | 0.482973679 | oligopeptide transporter 8 |
| CNXL_037090 | | -2.585347799 | 9.25444E-24 | hypothetical protein |
| CNXL_037100 | CNF02400 | -0.72445726 | 0.032186086 | hypothetical protein |

| | | | | |
|---|---|---|---|---|
| CNXL_037110 | CNF02410 | -0.188284487 | 0.787454471 | hypothetical protein |
| CNXL_037120 | | 1.462064136 | 0.113559841 | cytoplasmic protein |
| CNXL_037130 | CNF02420 | -0.378631771 | 0.372044973 | hypothetical protein |
| CNXL_037140 | CNF02430 | 0.115331616 | 0.850186907 | glyoxal oxidase |
| CNXL_037150 | CNF02440 | -6.591112735 | 4.16741E-72 | acyl-protein thioesterase 1 |
| CNXL_037160 | | 0.532779458 | 0.344867458 | hypothetical protein |
| CNXL_037170 | CNF02450 | -1.015060794 | 0.000179765 | hypothetical protein |
| CNXL_037180 | CNF02470 | -0.335768009 | 0.468867391 | hypothetical protein |
| CNXL_037190 | CNF02480 | 0.09733379 | 0.85183218 | hypothetical protein |
| CNXL_037200 | CNF02490 | -1.727734578 | 3.0985E-08 | ketol-acid reductoisomerase |
| CNXL_037210 | CNF02510 | 0.909119188 | 0.003842597 | hypothetical protein |
| CNXL_037220 | CNF02520 | -0.249980823 | 0.567887304 | alcohol dehydrogenase |
| CNXL_037230 | CNF02530 | -0.092677785 | 0.861349479 | alanine-tRNA ligase |
| CNXL_037240 | CNF02540 | 0.292918769 | 0.567594682 | multifunctional beta-oxidation protein |
| CNXL_037250 | CNF02550 | -0.135322366 | 0.866174286 | GTPase inhibitor |
| CNXL_037260 | CNF02560 | 0.412097877 | 0.276870355 | hypothetical protein |
| CNXL_037270 | CNF02570 | 0.061359319 | 0.935544021 | multidrug resistance protein fnx1 |
| CNXL_037280 | CNF02580 | -0.243308018 | 0.661992658 | hypothetical protein |
| CNXL_037290 | CNF02590 | 0.223654889 | 0.63756045 | hypothetical protein |
| CNXL_037300 | | -0.373283579 | 0.768292466 | charged multivesicular body protein 5 |
| CNXL_037310 | CNF02610 | -0.080441967 | 0.917445028 | hypothetical protein |
| CNXL_037320 | CNF02620 | -0.350587728 | 0.494759932 | hypothetical protein |
| CNXL_037330 | CNF02630 | -0.280025311 | 0.495183644 | elongator complex protein 1 |
| CNXL_037340 | CNF02640 | 0.196956992 | 0.706539593 | 6 |
| CNXL_037350 | CNF02650 | 0.377806417 | 0.388422979 | hypothetical protein |
| CNXL_037360 | CNF02660 | -0.03340056 | 0.965101267 | sorting nexin-3 |
| CNXL_037370 | CNF02670 | -0.071056232 | 0.924607253 | ubiquitin carboxyl-terminal hydrolase |
| CNXL_037380 | CNF02680 | -0.148197023 | 0.872477194 | cytoplasmic protein |
| CNXL_037390 | CNF02690 | 0.143616886 | 0.908809603 | hypothetical protein |
| CNXL_037400 | CNF02700 | 0.600635329 | 0.144402226 | hypothetical protein |
| CNXL_037410 | CNF02710 | -0.22271584 | 0.734939207 | ESCRT-II complex subunit VPS22 |
| CNXL_037420 | | -0.067299681 | 0.920669589 | rho GTPase-activating protein |
| CNXL_037430 | CNF02720 | -0.212132183 | 0.617998169 | hypothetical protein |
| CNXL_037440 | CNF02730 | -0.115806018 | 0.837168643 | tropomyosin |
| CNXL_037450 | | -1.159683864 | 0.067063478 | endoplasmic reticulum protein |
| CNXL_037460 | CNF02740 | 0.085931026 | 0.892423276 | hypothetical protein |
| CNXL_037470 | CNF02750 | 0.663192635 | 0.301460429 | hypothetical protein |
| CNXL_037480 | CNF02760 | -0.234894764 | 0.552854078 | hypothetical protein |
| CNXL_037490 | CNF02770 | 0.061670853 | 0.916084262 | ubiquitin-conjugating enzyme E2-16 kDa |
| CNXL_037500 | CNF02800 | 0.081092453 | 0.889001755 | glucosamine 6-phosphate N-acetyltransferase |
| CNXL_037510 | CNF02810 | -1.728096123 | 8.18575E-12 | CMGC/CK2 protein kinase |
| CNXL_037520 | CNF02820 | 0.137592284 | 0.807623732 | hypothetical protein |
| CNXL_037530 | | 0.193572016 | 0.701325477 | sphingosine-1-phosphate phosphatase |
| CNXL_037540 | CNF02840 | -0.148480311 | 0.770358678 | hypothetical protein |
| CNXL_037550 | CNF02850 | 0.154981804 | 0.776955714 | histone deacetylase |
| CNXL_037560 | CNF02860 | -0.083806264 | 0.894317988 | pre-mRNA-splicing factor |
| CNXL_037570 | | -0.072375009 | 0.907858192 | V-type ATPase |
| CNXL_037580 | CNM02440 | 0.447202678 | 0.260596728 | hypothetical protein |
| CNXL_037590 | CNF02890 | 0.356440265 | 0.527938546 | hypothetical protein |
| CNXL_037600 | | 0.401732956 | 0.419861756 | Unknown |
| CNXL_037610 | CNF02900 | 0.413269891 | 0.303744123 | Unknown |
| CNXL_037620 | CNF02910 | 1.926872162 | 0.051851032 | hypothetical protein |
| CNXL_037630 | CNF02920 | 1.078447212 | 0.000380759 | hypothetical protein |
| CNXL_037640 | CNF02930 | 0.304656694 | 0.474540273 | hypothetical protein |
| CNXL_037650 | CNF02940 | 0.164479669 | 0.747332879 | hypothetical protein |
| CNXL_037660 | CNF02950 | -0.134576795 | 0.78519633 | HAL protein kinase |
| CNXL_037670 | CNF02960 | -0.038823148 | 0.953095191 | pre-mRNA-splicing factor ini1 |
| CNXL_037680 | CNF02970 | 0.156883336 | 0.749039348 | membrane protein |

| | | | | |
|---|---|---|---|---|
| CNXL_037690 | CNF02980 | -0.362815505 | 0.315522035 | phospholipase |
| CNXL_037700 | CNF02990 | -0.40346886 | 0.36623284 | trimethyllysine dioxygenase |
| CNXL_037710 | CNF03000 | 0.0655439 | 0.914338536 | solute carrier family 35 |
| CNXL_037720 | CNF03020 | -0.110039462 | 0.940332853 | GPI inositol-deacylase |
| CNXL_037730 | | NA | NA | oxidation resistance protein 1 |
| CNXL_037740 | CNF03110 | 0 | NA | Unknown |
| CNXL_037750 | | NA | NA | Unknown |
| CNXL_037760 | | -1.330752087 | NA | Unknown |
| CNXL_037770 | | 0.63431984 | 0.06047424 | Unknown |
| CNXL_037780 | CNF03130 | 1.089323126 | 0.001195513 | hypothetical protein |
| CNXL_037790 | | NA | NA | hypothetical protein |
| CNXL_037800 | | NA | NA | Unknown |
| CNXL_037810 | CNF03170 | 1.198031908 | 4.28007E-07 | Unknown |
| CNXL_037820 | CNF03150 | 0.590987406 | 0.194555297 | hypothetical protein |
| CNXL_037830 | | 0.324059138 | 0.52947806 | Unknown |
| CNXL_037840 | CNF03160 | 0.698146736 | 0.023029677 | hypothetical protein |
| CNXL_037850 | CNF03180 | 1.313842792 | 1.04255E-05 | glyceraldehyde-3-phosphate dehydrogenase |
| CNXL_037860 | | 0.246000521 | 0.7126331 | phosphatase |
| CNXL_037870 | | -0.197731138 | 0.770559628 | hypothetical protein |
| CNXL_037880 | CNF03200 | 0.240326929 | 0.61517823 | TTK protein kinase |
| CNXL_037890 | | 0.672282217 | 0.126856702 | protein phosphatase PP2A regulatory subunit B |
| CNXL_037900 | CNF03210 | 0.010958656 | 0.990008547 | hypothetical protein |
| CNXL_037910 | | 0.60472241 | 0.065740795 | hydroxyisourate hydrolase |
| CNXL_037920 | CNF03220 | 0.655778529 | 0.017777565 | hypothetical protein |
| CNXL_037930 | CNF03230 | -0.040128112 | 0.962926966 | glucosamine-phosphate N-acetyltransferase |
| CNXL_037940 | CNF03240 | 0.537833414 | 0.106477482 | tuftelin-interacting protein 11 |
| CNXL_037950 | CNF03250 | -0.011307494 | 0.990008547 | hypothetical protein |
| CNXL_037960 | CNF03260 | -0.167950519 | 0.746537956 | cytoplasmic protein |
| CNXL_037970 | CNF03270 | -0.294020587 | 0.567249149 | mitofusin |
| CNXL_037980 | CNF03280 | 0.078943835 | 0.936507583 | hypothetical protein |
| CNXL_037990 | CNF03300 | 0.223286848 | 0.719067691 | Unknown |
| CNXL_038000 | CNF03310 | -0.118202561 | 0.847311574 | hypothetical protein |
| CNXL_038010 | CNF03320 | -0.288996465 | 0.607065672 | U2-associated protein SR140 |
| CNXL_038020 | CNF03330 | -0.091543981 | 0.903834486 | pre-rRNA-processing protein IPI3 |
| CNXL_038030 | | 0.233603424 | 0.901127763 | hypothetical protein |
| CNXL_038040 | CNF03360 | -0.704141212 | 0.137357068 | hypothetical protein |
| CNXL_038050 | CNF03370 | -0.611177634 | 0.083435528 | NADH dehydrogenase |
| CNXL_038060 | CNF03390 | 0.0718694 | 0.915837202 | hypothetical protein |
| CNXL_038070 | CNF03400 | -0.012267276 | 0.988873975 | hypothetical protein |
| CNXL_038080 | CNF03410 | -0.247244924 | 0.652998487 | COP9 signalosome complex subunit 6 |
| CNXL_038090 | CNF03420 | -0.312014976 | 0.569605611 | anthranilate synthase component I |
| CNXL_038100 | | 0.35426396 | 0.336796999 | putative chitin synthase regulator |
| CNXL_038110 | CNF03430 | 0.008726631 | 0.992176872 | hypothetical protein |
| CNXL_038120 | CNF03440 | -0.051552438 | 0.955330361 | hypothetical protein |
| CNXL_038130 | | -0.154032463 | 0.832551879 | hypothetical protein |
| CNXL_038140 | CNF03460 | 0.374187608 | 0.372306093 | hypothetical protein |
| CNXL_038150 | CNF03470 | 1.320769362 | 1.41696E-05 | hypothetical protein |
| CNXL_038160 | CNF03480 | 0.118288763 | 0.842111361 | formate dehydrogenase |
| CNXL_038170 | CNF03490 | 0.27935158 | 0.566535508 | TKL protein kinase |
| CNXL_038180 | | 0.295215541 | 0.521375775 | HIV Tat-specific factor 1 |
| CNXL_038190 | CNF03510 | 1.010657466 | 0.000997584 | hypothetical protein |
| CNXL_038200 | CNF03520 | 0.165056288 | 0.764533957 | mitochondrial protein |
| CNXL_038210 | CNF03530 | -0.080354376 | 0.885449489 | hypothetical protein |
| CNXL_038220 | CNF03540 | 0.353031168 | 0.551291645 | starch phosphorylase |
| CNXL_038230 | CNF03550 | 0.442282172 | 0.232925502 | hypothetical protein |
| CNXL_038240 | CNF03560 | -0.24200884 | 0.619100215 | sorting nexin MVP1 |
| CNXL_038250 | | 0.078583357 | 0.984913368 | ubiquinol-cytochrome c reductase subunit 6 |
| CNXL_038260 | CNF03580 | 0.31510267 | 0.440160428 | Unknown |

| | | | | |
|---|---|---|---|---|
| CNXL_038270 | CNF03590 | -0.438735323 | 0.312160498 | hypothetical protein |
| CNXL_038280 | CNF03610 | -0.134056954 | 0.841354437 | hypothetical protein |
| CNXL_038290 | | -0.057078475 | 0.953145249 | hypothetical protein |
| CNXL_038300 | CNF03620 | -0.46503964 | 0.142649555 | Unknown |
| CNXL_038310 | CNF03630 | -0.516477872 | 0.21653299 | beta-hexosaminidase |
| CNXL_038320 | CNF03640 | 0.895028827 | 0.009892771 | rhomboid family membrane protein |
| CNXL_038330 | CNF03650 | -0.206588512 | 0.705198732 | hypothetical protein |
| CNXL_038340 | CNF03660 | 0.125659098 | 0.867431426 | diphthine synthase |
| CNXL_038350 | CNF03670 | -0.201286403 | 0.789423788 | GPI-anchor transamidase |
| CNXL_038360 | | 0.224342547 | 0.650714942 | hypothetical protein |
| CNXL_038370 | CNF03680 | 0.349334882 | 0.488583556 | Unknown |
| CNXL_038380 | CNF03690 | -0.079193758 | 0.93471488 | mRNA polymerase-associated protein |
| CNXL_038390 | CNF03700 | 0.201200677 | 0.7126331 | hypothetical protein |
| CNXL_038400 | CNF03710 | -0.195322491 | 0.753537236 | membrane trafficking and ER morphology protein |
| CNXL_038410 | CNF03720 | -0.647742813 | 0.069009964 | methylenetetrahydrofolate dehydrogenase [NAD |
| CNXL_038420 | CNF03730 | -1.213232332 | 1.40474E-05 | C-22 sterol desaturase |
| CNXL_038430 | CNF03740 | 0.110131475 | 0.850544343 | ribonuclease III |
| CNXL_038440 | CNF03750 | -0.093066148 | 0.875743895 | Tor protein kinase |
| CNXL_038450 | CNF03760 | 0.126559921 | 0.830440343 | DNA-directed RNA polymerase III subunit RPC6 |
| CNXL_038460 | CNF03770 | -0.558774682 | 0.088587844 | cathepsin A |
| CNXL_038470 | CNF03780 | -0.425101955 | 0.214364087 | E3 ubiquitin-protein ligase MARCH6 |
| CNXL_038480 | CNF03800 | -0.061312481 | 0.943193944 | malate dehydrogenase |
| CNXL_038490 | CNF03810 | -1.066601635 | 0.002051794 | ubiquitin carboxyl-terminal hydrolase 22/27/51 |
| CNXL_038500 | CNF03820 | -0.313320104 | 0.498878677 | hypothetical protein |
| CNXL_038510 | CNF03830 | -0.413639395 | 0.51244137 | CCR4-NOT transcription complex subunit 3 |
| CNXL_038520 | CNF03840 | -0.052566783 | 0.933394957 | DNA polymerase epsilon subunit B |
| CNXL_038530 | CNF03860 | 0.75671844 | 0.012180411 | small subunit ribosomal protein S15 |
| CNXL_038540 | CNF03870 | -0.027358523 | 0.976443371 | Atypical/ABC1 protein kinase |
| CNXL_038550 | CNF03880 | -0.022362228 | 0.979695435 | myosin heavy chain |
| CNXL_038560 | CNF03890 | -0.434765319 | 0.341532505 | import inner membrane translocase subunit tim44 |
| CNXL_038570 | CNF03900 | 0.300535346 | 0.425328399 | Unknown |
| CNXL_038580 | CNF03910 | 0.065581907 | 0.923298629 | aldehyde dehydrogenase |
| CNXL_038590 | CNF03920 | -0.143102194 | 0.779355929 | pre-mRNA-processing factor 40 |
| CNXL_038600 | CNF03930 | 0.599386628 | 0.078923725 | hypothetical protein |
| CNXL_038610 | CNF03940 | -0.208946344 | 0.742187954 | UBX domain-containing protein 1 |
| CNXL_038620 | CNF03950 | 0.774648646 | 0.030574464 | hypothetical protein |
| CNXL_038630 | CNF03960 | 0.56774302 | 0.054428043 | inositol oxygenase |
| CNXL_038640 | CNF03970 | 0.237665899 | 0.562700958 | hypothetical protein |
| CNXL_038650 | CNF03990 | -0.558317669 | 0.17290643 | biotin synthase |
| CNXL_038660 | CNF04000 | -0.11116215 | 0.874351584 | mitochondrial Rho GTPase 1 |
| CNXL_038670 | | -1.409646926 | 0.001440555 | mitochondrial protein |
| CNXL_038680 | CNF04020 | 0.050678235 | 0.944879244 | hypothetical protein |
| CNXL_038690 | CNF04030 | 0.143760098 | 0.800552138 | hypothetical protein |
| CNXL_038700 | CNF04050 | -0.750452999 | 0.008654672 | hypothetical protein |
| CNXL_038710 | CNF04060 | -0.075228121 | 0.906286943 | glycerophosphodiesterase |
| CNXL_038720 | CNF04070 | 0.1027573 | 0.87555787 | endoplasmic reticulum protein |
| CNXL_038730 | CNF04080 | -0.599246237 | 0.098349258 | hypothetical protein |
| CNXL_038740 | CNF04090 | -1.053030628 | 0.001450786 | DNA-directed RNA polymerase I subunit RPA2 |
| CNXL_038750 | | 0.38982867 | 0.930990489 | MFS transporter |
| CNXL_038760 | CNF04110 | -0.637453834 | 0.264189715 | hypothetical protein |
| CNXL_038770 | CNF04120 | 0.467517827 | 0.179438445 | hypothetical protein |
| CNXL_038780 | CNF04130 | -0.357877601 | 0.417866535 | Unknown |
| CNXL_038790 | CNF04140 | -0.261914046 | 0.540948602 | DNA polymerase alpha subunit A |
| CNXL_038800 | CNF04150 | -0.302250549 | 0.452146814 | rho family GTPase |
| CNXL_038810 | CNF04160 | 0.251855079 | 0.720698078 | small subunit ribosomal protein S2 |
| CNXL_038820 | CNF04170 | -0.370144972 | 0.432050869 | hypothetical protein |
| CNXL_038830 | | 0.545399709 | 0.354219773 | di- and tripeptidase |
| CNXL_038840 | CNF04180 | 0.127868435 | 0.875142696 | Unknown |

| | | | | |
|---|---|---|---|---|
| CNXL_038850 | CNF04190 | 0.334605272 | 0.43363696 | amidohydrolase |
| CNXL_038860 | CNF04200 | -0.10433579 | 0.919952257 | vesicle-fusing ATPase |
| CNXL_038870 | | -0.076306986 | 0.965101267 | hypothetical protein |
| CNXL_038880 | CNF04210 | -0.018772432 | 0.983318676 | hypothetical protein |
| CNXL_038890 | CNF04220 | -0.832701272 | 0.217721523 | hypothetical protein |
| CNXL_038900 | CNF04230 | -0.376012091 | 0.755488326 | hypothetical protein |
| CNXL_038910 | CNF04240 | 0.603975569 | 0.062208681 | hypothetical protein |
| CNXL_038920 | CNF04250 | -0.366322424 | 0.292795277 | oxysterol binding protein |
| CNXL_038930 | CNF04270 | -0.002909363 | 0.996472611 | rhamnogalacturonan lyase |
| CNXL_038940 | CNF04280 | -0.09420018 | 0.868600207 | ubiquitin-conjugating enzyme |
| CNXL_038950 | | 1.018220121 | 0.035602799 | histone deacetylase complex protein |
| CNXL_038960 | CNF04290 | -0.23430529 | 0.732889168 | Unknown |
| CNXL_038970 | CNF04310 | -0.339044357 | 0.592736437 | hypothetical protein |
| CNXL_038980 | CNF04330 | 0.013073678 | 0.987759522 | cytoplasmic protein |
| CNXL_038990 | | 0.530011348 | 0.782173947 | pre-mRNA-splicing helicase BRR2 |
| CNXL_039000 | CNF04340 | -0.73049045 | 0.055617738 | Unknown |
| CNXL_039010 | CNF04350 | 0.494112288 | 0.189297101 | hypothetical protein |
| CNXL_039020 | | -0.36489339 | 0.510006134 | hypothetical protein |
| CNXL_039030 | CNF04390 | -1.177852804 | 0.000157781 | hypothetical protein |
| CNXL_039040 | | -0.103122041 | 0.885449489 | hypothetical protein |
| CNXL_039050 | CNF04400 | -0.637619119 | 0.014346309 | Unknown |
| CNXL_039060 | CNF04410 | 1.407628645 | 0.000468261 | hypothetical protein |
| CNXL_039070 | CNF04420 | 1.817439159 | 0.170587304 | hypothetical protein |
| CNXL_039080 | CNF04430 | 1.286205061 | 2.93714E-06 | cAMP-regulated gene 1 |
| CNXL_039090 | CNF04440 | -0.118260313 | 0.866875842 | secreted antiphagocytic protein |
| CNXL_039100 | CNF04450 | -0.439786701 | 0.385883789 | hypothetical protein |
| CNXL_039110 | CNF04470 | 0.310109639 | 0.431497115 | pyruvate dehydrogenase |
| CNXL_039120 | CNF04480 | 0.025486666 | 0.972638293 | hypothetical protein |
| CNXL_039130 | CNF04490 | 0.814197963 | 0.00331677 | membrane transporter |
| CNXL_039140 | CNF04500 | 0.645500598 | 0.095737732 | RAN protein kinase |
| CNXL_039150 | CNF04510 | 0.23276799 | 0.648883862 | hypothetical protein |
| CNXL_039160 | CNF04520 | 0.24572559 | 0.584869453 | hypothetical protein |
| CNXL_039170 | CNF04530 | -0.373617241 | 0.357567274 | hypothetical protein |
| CNXL_039180 | CNF04540 | -0.648510504 | 0.103886914 | clathrin-coated vesicle protein |
| CNXL_039190 | | 0.757609782 | 0.031003853 | translation initiation factor 3 subunit F |
| CNXL_039200 | | 1.344411592 | 0.213868556 | hypothetical protein |
| CNXL_039210 | | 0.674785908 | 0.404812566 | Unknown |
| CNXL_039220 | CNF04560 | -0.765444669 | 0.009648212 | Unknown |
| CNXL_039230 | CNF04570 | 0.418446522 | 0.318563249 | allantoate transporter |
| CNXL_039240 | CNF04580 | 0.687933749 | 0.102947308 | esterase |
| CNXL_039250 | CNF04590 | 0.064483597 | 0.928595731 | cysteine and glycine-rich protein |
| CNXL_039260 | CNF04600 | -0.378686617 | 0.357567274 | NADPH-ferrihemoprotein reductase |
| CNXL_039270 | CNF04610 | -0.979968463 | 0.040140554 | membrane protein |
| CNXL_039280 | CNF04620 | -0.017549633 | 0.985173026 | oxidoreductase |
| CNXL_039290 | CNF04630 | -0.131220405 | 0.819461283 | aromatic amino acid aminotransferase I |
| CNXL_039300 | CNF04640 | 0.258910772 | 0.635645504 | FAD dependent oxidoreductase |
| CNXL_039310 | CNF04650 | -0.095557846 | 0.881752079 | hypothetical protein |
| CNXL_039320 | CNF04660 | -0.177575765 | 0.74676769 | CAMK/CAMKL/AMPK protein kinase |
| CNXL_039330 | CNF04670 | -0.303854943 | 0.570605968 | carnitine O-acetyltransferase |
| CNXL_039340 | CNF04680 | 0.089717203 | 0.919952257 | nucleoporin family protein |
| CNXL_039350 | CNF04690 | 0.051989376 | 0.934678457 | mRNA-capping enzyme subunit beta |
| CNXL_039360 | CNF04700 | -0.215148044 | 0.724615229 | endoplasmic reticulum protein |
| CNXL_039370 | CNF04710 | -0.310247882 | 0.884028948 | U3 small nucleolar RNA-associated protein 19 |
| CNXL_039380 | NF04720\|CNF047[illegible] | 0.036672968 | 0.95265881 | hypothetical protein |
| CNXL_039390 | CNF04740 | -0.09716854 | 0.868600207 | Unknown |
| CNXL_039400 | CNF04750 | -0.150015593 | 0.835972626 | ethanolaminephosphotransferase |
| CNXL_039410 | CNF04760 | 0.739230948 | 0.046397979 | hypothetical protein |
| CNXL_039420 | CNF04770 | 0.138297162 | 0.799344206 | hypothetical protein |

| | | | | |
|---|---|---|---|---|
| CNXL_039430 | CNF04780 | 0.505518373 | 0.091914258 | dihydroxy-acid dehydratase |
| CNXL_039440 | CNF04790 | 0.003095365 | 0.996374193 | monocarboxylic acid transporter |
| CNXL_039450 | CNF04800 | 0.095101224 | 0.911130835 | monocarboxylic acid transporter |
| CNXL_039460 | CNF04810 | 0.701033389 | 0.184176331 | monocarboxylic acid transporter |
| CNXL_039470 | CNF04820 | -0.144183464 | 0.870087679 | monocarboxylic acid transporter |
| CNXL_039480 | CNF04830 | -1.275669494 | 1.57314E-05 | ribosome biogenesis protein UTP30 |
| CNXL_039490 | CNF04850 | 0.708004476 | 0.029494128 | hydroxymethylglutaryl-CoA reductase |
| CNXL_039500 | CNF04860 | 0.145384544 | 0.781361786 | Unknown |
| CNXL_039510 | CNF04870 | 0.427918614 | 0.293518315 | ATP-dependent permease |
| CNXL_039520 | CNF04880 | -0.618335408 | 0.13866828 | hypothetical protein |
| CNXL_039530 | CNF04890 | 0.519716113 | 0.298784347 | hypothetical protein |
| CNXL_039540 | CNF04900 | 0.141144079 | 0.797632564 | hypothetical protein |
| CNXL_039550 | CNF04910 | 0.39188779 | 0.567369556 | hypothetical protein |
| CNXL_039560 | | 0.69525989 | 0.273564602 | hypothetical protein |
| CNXL_039570 | | 0.795526988 | 0.219149239 | Unknown |
| CNXL_039580 | CNF04930 | 0.197837685 | 0.833643586 | Unknown |
| CNXL_039590 | CNF04940 | -0.811982317 | 0.420251154 | Unknown |
| CNXL_039600 | | -0.678580695 | 0.10562276 | Unknown |
| CNXL_039610 | CNG00010 | -0.320180121 | 0.425739759 | Unknown |
| CNXL_039620 | | 0.157932574 | 0.78519633 | nicotinamide mononucleotide permease |
| CNXL_039630 | CNG00020 | 0.246291328 | 0.566540169 | magnesium-dependent phosphatase-1 |
| CNXL_039640 | CNG00030 | 0.360111238 | 0.346301684 | hypothetical protein |
| CNXL_039650 | CNG00040 | -0.052746214 | 0.943586417 | phytase |
| CNXL_039660 | CNG00050 | 0.058116238 | 0.934072032 | Unknown |
| CNXL_039670 | CNG00060 | 1.164640839 | 0.010092763 | Unknown |
| CNXL_039680 | CNG00070 | 0.640466137 | 0.160213488 | UDP-galactopyranose mutase |
| CNXL_039690 | | 0.905890733 | 0.008730468 | hypothetical protein |
| CNXL_039700 | CNG00100 | -1.465468574 | 3.53205E-06 | hypothetical protein |
| CNXL_039710 | CNG00090 | 0.691296326 | 0.067525023 | Unknown |
| CNXL_039720 | CNG00110 | -0.276022899 | 0.589560207 | Unknown |
| CNXL_039730 | CNG00120 | -0.045696788 | 0.954505021 | ferric reductase |
| CNXL_039740 | CNG00130 | -0.122599394 | 0.854840132 | siderophore iron transporter MirB |
| CNXL_039750 | CNG00140 | 0.304081632 | 0.487397795 | phosphomethylpyrimidine kinase |
| CNXL_039760 | CNG00150 | -0.102079289 | 0.863067672 | RAB GDP-dissociation inhibitor |
| CNXL_039770 | CNG00160 | 0.069949858 | 0.921327484 | casein kinase II beta chain |
| CNXL_039780 | CNG00170 | -0.451342632 | 0.376887576 | adrenodoxin-type ferredoxin |
| CNXL_039790 | CNG00180 | -0.147139446 | 0.800552138 | L-aminoadipate-semialdehyde dehydrogenase |
| CNXL_039800 | CNG00190 | -0.013652678 | 0.988427236 | hypothetical protein |
| CNXL_039810 | CNG00210 | 0.326854517 | 0.481606389 | hypothetical protein |
| CNXL_039820 | CNG00230 | 0.025270077 | 0.976439859 | hypothetical protein |
| CNXL_039830 | CNG00240 | -0.009618284 | 0.991548886 | Prp8 binding protein |
| CNXL_039840 | CNG00250 | 0.37035867 | 0.39502065 | hypothetical protein |
| CNXL_039850 | CNG00270 | -0.024991073 | 0.975873016 | escrt-III-associated proteolytic activator of rim101 |
| CNXL_039860 | CNG00280 | -0.132680633 | 0.883172612 | 3-ketodihydrosphingosine reductase TSC10 |
| CNXL_039870 | CNG00290 | -0.086834567 | 0.931907099 | tRNA |
| CNXL_039880 | CNG00300 | -0.455514456 | 0.248429743 | hypothetical protein |
| CNXL_039890 | | -1.486971066 | 2.36231E-09 | large subunit ribosomal protein LP0 |
| CNXL_039900 | CNG00310 | -0.168492326 | 0.818704891 | cytochrome C assembly protein |
| CNXL_039910 | CNG00320 | 0.020036581 | 0.981240834 | alpha-L-rhamnosidase |
| CNXL_039920 | | 0.146059282 | 0.893955723 | acyl-CoA oxidase |
| CNXL_039930 | CNG00340 | 0.698141556 | 0.010305709 | Unknown |
| CNXL_039940 | CNG00350 | 1.777751943 | 6.7576E-07 | kynurenine 3-monooxygenase |
| CNXL_039950 | | 1.119379248 | 0.025871919 | opsin-1 |
| CNXL_039960 | CNG00360 | -0.052552418 | 0.939202685 | Unknown |
| CNXL_039970 | CNG00370 | 1.251043534 | 9.75772E-05 | serine/threonine kinase of the ram signaling pathway |
| CNXL_039980 | CNG00380 | 0.763768655 | 0.011065934 | hypothetical protein |
| CNXL_039990 | CNG00390 | 0.862011794 | 0.006579792 | plasma-membrane proton-efflux P-type ATPase |
| CNXL_040000 | CNG00400 | 1.154702372 | 0.002642296 | hypothetical protein |

| | | | | |
|---|---|---|---|---|
| CNXL_040010 | CNG00410 | -0.748726852 | 0.256378694 | aspartate-tRNA |
| CNXL_040020 | CNG00420 | 0.209593039 | 0.714520393 | D-amino-acid oxidase |
| CNXL_040030 | CNG00430 | -0.809867229 | 0.018540265 | hypothetical protein |
| CNXL_040040 | CNG00440 | -0.525055889 | 0.199822977 | hypothetical protein |
| CNXL_040050 | | 0.733444688 | 0.010373145 | allantoate permease |
| CNXL_040060 | CNG00450 | 0.392531489 | 0.36024513 | hypothetical protein |
| CNXL_040070 | CNG00460 | 0.220797177 | 0.660204343 | transcription initiation factor TFIIA small subunit |
| CNXL_040080 | CNG00470 | -0.273863474 | 0.626371103 | dimethylaniline monooxygenase |
| CNXL_040090 | CNG00480 | -0.091106671 | 0.869804835 | acylglycerone-phosphate reductase |
| CNXL_040100 | CNG00490 | 0.058925729 | 0.927441884 | coatomer protein complex |
| CNXL_040110 | CNG00500 | -0.082178451 | 0.897557388 | hypothetical protein |
| CNXL_040120 | | 0.092972981 | 0.969527869 | hypothetical protein |
| CNXL_040130 | CNG00510 | 0.258754537 | 0.678124378 | hypothetical protein |
| CNXL_040140 | CNG00520 | -1.036586022 | 0.000352124 | zinc finger HIT domain-containing protein 1 |
| CNXL_040150 | CNG00530 | -0.062101365 | 0.92070335 | hypothetical protein |
| CNXL_040160 | CNG00540 | -0.491296905 | 0.21599669 | hypothetical protein |
| CNXL_040170 | CNG00550 | 0.201305409 | 0.698369977 | hypothetical protein |
| CNXL_040180 | CNG00560 | 0.127440662 | 0.814480545 | arginase |
| CNXL_040190 | CNG00570 | 0.219834896 | 0.669593192 | hypothetical protein |
| CNXL_040200 | CNG00580 | -0.303940251 | 0.814220979 | aspartyl/glutamyl-tRNA |
| CNXL_040210 | CNG00590 | -0.495022295 | 0.206923398 | hypothetical protein |
| CNXL_040220 | CNG00600 | -1.578136767 | 3.56753E-12 | methylenetetrahydrofolate dehydrogenase |
| CNXL_040230 | CNG00610 | -0.18833801 | 0.781467115 | mannitol-1-phosphate dehydrogenase |
| CNXL_040240 | | 0.203071131 | 0.708742909 | 1-phosphatidylinositol 4-kinase |
| CNXL_040250 | CNG00620 | 0.044794862 | 0.954505021 | hypothetical protein |
| CNXL_040260 | CNG00630 | -0.934868596 | 0.004844914 | hypothetical protein |
| CNXL_040270 | CNG00640 | 0.129942243 | 0.838716167 | 3-beta hydroxysteroid dehydrogenase/isomerase |
| CNXL_040280 | CNG00650 | -0.181112351 | 0.761797019 | 3-beta hydroxysteroid dehydrogenase/isomerase |
| CNXL_040290 | CNG00660 | -0.292001396 | 0.461869386 | hypothetical protein |
| CNXL_040300 | CNG00670 | -0.177398177 | 0.77300142 | AP-2 complex subunit alpha |
| CNXL_040310 | CNG00680 | 0.032041226 | 0.981240834 | cytoplasmic protein |
| CNXL_040320 | CNA05340 | 1.857883414 | 8.46541E-07 | chromosome transmission fidelity protein 8 |
| CNXL_040330 | CNG00690 | 1.392405233 | 3.59346E-05 | Unknown |
| CNXL_040340 | CNG00700 | -0.507529248 | 0.078954247 | alpha |
| CNXL_040350 | | 0.313709314 | 0.515650743 | transmembrane receptor |
| CNXL_040360 | CNG00710 | 0.314749086 | 0.566713427 | solute carrier family 25 |
| CNXL_040370 | CNG00720 | -0.519155209 | 0.422333191 | hypothetical protein |
| CNXL_040380 | CNG00730 | 0.067802554 | 0.938635155 | DNA-directed RNA polymerase III subunit RPC10 |
| CNXL_040390 | | 1.333801455 | 0.30555532 | hypothetical protein |
| CNXL_040400 | | 0.651915792 | 0.580345903 | Unknown |
| CNXL_040410 | | 0.566197202 | 0.224254322 | Unknown |
| CNXL_040420 | CNG00750 | 1.099787738 | 0.001130193 | Unknown |
| CNXL_040430 | | 0.82498138 | 0.030569633 | NADH dehydrogenase |
| CNXL_040440 | | 0.519920045 | 0.427104346 | Unknown |
| CNXL_040450 | CNG00770 | -1.24326851 | 2.91978E-06 | Unknown |
| CNXL_040460 | CNG00780 | 0.195741264 | 0.749050005 | hypothetical protein |
| CNXL_040470 | CNG00790 | 0.115949481 | 0.828863599 | cytoplasmic protein |
| CNXL_040480 | CNG00800 | -0.042830572 | 0.956370213 | hypothetical protein |
| CNXL_040490 | CNG00810 | 0.253353746 | 0.623094586 | ribosomal RNA-processing protein 7 |
| CNXL_040500 | CNG00820 | 0.619573186 | 0.04832917 | hypothetical protein |
| CNXL_040510 | CNG00830 | -0.105951014 | 0.8610985 | hypothetical protein |
| CNXL_040520 | CNG00840 | -0.647365064 | 0.064718468 | large subunit ribosomal protein L36e |
| CNXL_040530 | CNG00850 | -0.207803796 | 0.664461114 | pyruvate dehydrogenase X component |
| CNXL_040540 | CNG00860 | -0.525708832 | 0.202621105 | hypothetical protein |
| CNXL_040550 | | -0.121511166 | 0.855752398 | mitochondrial-processing peptidase subunit beta |
| CNXL_040560 | CNG00890 | -0.696330762 | 0.02488041 | hypothetical protein |
| CNXL_040570 | CNG00900 | -0.299565894 | 0.489809679 | hypothetical protein |
| CNXL_040580 | CNG00910 | 0.602852447 | 0.052607176 | ATP-binding cassette transporter |

| | | | | |
|---|---|---|---|---|
| CNXL_040590 | CNG00930 | 0.117386247 | 0.890150525 | putative metal ion transporter |
| CNXL_040600 | CNG00940 | 0.482300452 | 0.149174594 | osmosensor |
| CNXL_040610 | CNG00950 | 0.804257512 | 0.015153011 | twin-arginine translocation pathway signal peptide |
| CNXL_040620 | CNG00960 | 0.080857721 | 0.899602567 | ferric reductase |
| CNXL_040630 | | 0.700743073 | 0.066164191 | BET3 family protein |
| CNXL_040640 | CNG00980 | 0.391450047 | 0.337819601 | hypothetical protein |
| CNXL_040650 | CNG00990 | -0.265760796 | 0.655081011 | hypothetical protein |
| CNXL_040660 | CNG01000 | 0.628920257 | 0.197695589 | 1-alkyl-2-acetylglycerophosphocholine esterase |
| CNXL_040670 | CNG01010 | -2.46759581 | 7.3948E-14 | hypothetical protein |
| CNXL_040680 | CNG01020 | -0.10120694 | 0.889001755 | hypothetical protein |
| CNXL_040690 | CNG01030 | 0.213215222 | 0.661992658 | hypothetical protein |
| CNXL_040700 | CNG01040 | -0.170521147 | 0.785269877 | RNA recognition domain-containing protein |
| CNXL_040710 | CNG01050 | -1.236586327 | 0.086136893 | hypothetical protein |
| CNXL_040720 | CNG01060 | -0.20838273 | 0.638378602 | DNAJ domain-containing protein |
| CNXL_040730 | CNG01070 | -0.091169274 | 0.902044607 | peptidyl-prolyl cis-trans isomerase B |
| CNXL_040740 | CNG01080 | -0.224118852 | 0.746537956 | polyamine transporter 1 |
| CNXL_040750 | CNG01090 | 0.298664787 | 0.48943204 | kinetochore protein Mis13/DSN1 |
| CNXL_040760 | CNG01100 | -0.905218579 | 0.001757212 | protein GET1 |
| CNXL_040770 | | -0.365064493 | 0.628160086 | thiol peroxidase |
| CNXL_040780 | CNG01110 | -0.493081025 | 0.175271548 | ribonuclease P protein component |
| CNXL_040790 | CNG01120 | -0.0456497 | 0.955507961 | Unknown |
| CNXL_040800 | CNG01130 | -0.126066109 | 0.854291768 | Unknown |
| CNXL_040810 | CNG01140 | -0.530830959 | 0.195861015 | NAD epimerase domain-containing protein |
| CNXL_040820 | CNG01150 | -0.491999297 | 0.184521912 | hypothetical protein |
| CNXL_040830 | CNG01160 | -0.264119873 | 0.536803217 | spermidine synthase |
| CNXL_040840 | CNG01170 | 0.236864946 | 0.793622194 | hypothetical protein |
| CNXL_040850 | CNG01190 | 0.010231364 | 0.993656238 | efflux protein EncT |
| CNXL_040860 | CNG01200 | -0.424681275 | 0.399675058 | lipoyl |
| CNXL_040870 | CNG01210 | 0.017293898 | 0.982477673 | glucan endo-1 |
| CNXL_040880 | | 0.442109063 | 0.706539593 | U3 small nucleolar RNA-associated protein 25 |
| CNXL_040890 | CNG01220 | 0.454639327 | 0.397048245 | Unknown |
| CNXL_040900 | | 0.01773732 | 0.983318676 | hypothetical protein |
| CNXL_040910 | CNG01240 | 0.647418551 | 0.010542889 | putative mRNA-dependent RNA polymerase |
| CNXL_040920 | | 0.523716736 | 0.225686862 | laccase |
| CNXL_040930 | CNG01260 | 0.449821134 | 0.264827301 | laccase |
| CNXL_040940 | | 0.503446786 | 0.332332744 | hypothetical protein |
| CNXL_040950 | | -1.940906379 | 6.59174E-18 | Unknown |
| CNXL_040960 | CNG01280 | 0.126479352 | 0.869733568 | hypothetical protein |
| CNXL_040970 | CNG01290 | 0.090382253 | 0.886480005 | phosphoglycerate dehydrogenase |
| CNXL_040980 | CNG01300 | 0.139427452 | 0.797485767 | T-complex protein 1 subunit delta |
| CNXL_040990 | CNG01310 | -0.376543839 | 0.354037215 | hypothetical protein |
| CNXL_041000 | CNG01320 | 0.051887972 | 0.939255348 | arginine-tRNA ligase |
| CNXL_041010 | | -0.271648262 | 0.510006134 | hypothetical protein |
| CNXL_041020 | CNG01330 | 1.226589076 | 0.174732924 | hypothetical protein |
| CNXL_041030 | CNG01340 | -1.160754853 | 0.063702758 | hypothetical protein |
| CNXL_041040 | CNG01350 | -0.787045212 | 0.073861425 | kinesin-like protein |
| CNXL_041050 | CNG01360 | 0.096993853 | 0.877313027 | AFG1 family mitochondrial ATPase |
| CNXL_041060 | CNG01370 | 0.034037113 | 0.965101267 | hypothetical protein |
| CNXL_041070 | CNG01380 | -0.206809402 | 0.696473798 | ATP-binding cassette transporter |
| CNXL_041080 | CNG01390 | 0.048012563 | 0.955507961 | DNA repair protein |
| CNXL_041090 | CNG01400 | 0.087084026 | 0.917496987 | translation initiation factor eIF-2B subunit delta |
| CNXL_041100 | CNG01410 | 0.154440451 | 0.809974479 | hypothetical protein |
| CNXL_041110 | CNG01420 | 0.296326848 | 0.496574337 | hypothetical protein |
| CNXL_041120 | CNG01430 | 0.009145469 | 0.992176872 | hypothetical protein |
| CNXL_041130 | CNG01440 | 0.17964244 | 0.787627196 | hypothetical protein |
| CNXL_041140 | CNG01450 | 0.179413954 | 0.776672985 | phosphatidylinositol glycan |
| CNXL_041150 | CNG01460 | 0.062458081 | 0.936783812 | small subunit ribosomal protein S21 |
| CNXL_041160 | CNG01470 | -0.148057443 | 0.776935181 | hypothetical protein |

| | | | | |
|---|---|---|---|---|
| CNXL_041170 | CNG01480 | 0.804586267 | 0.002951367 | hypothetical protein |
| CNXL_041180 | | 0.082064595 | 0.885449489 | hexose transporter |
| CNXL_041190 | CNG01490 | -0.368116155 | 0.417866535 | hypothetical protein |
| CNXL_041200 | CNG01500 | -0.093014717 | 0.881726613 | alanine transaminase |
| CNXL_041210 | CNG01510 | 0.055507205 | 0.935690014 | ribonucleoprotein-associated protein |
| CNXL_041220 | CNG01530 | 0.226972518 | 0.647175553 | solute carrier family 45 |
| CNXL_041230 | CNG01540 | 0.856038626 | 0.00655686 | endo alpha-1 |
| CNXL_041240 | CNG01550 | 0.305226802 | 0.475340486 | solute carrier family 2 |
| CNXL_041250 | CNG01560 | 0.096000018 | 0.877313027 | nuclear protein |
| CNXL_041260 | CNG01570 | 0.336422576 | 0.521085092 | amidohydrolase 2 |
| CNXL_041270 | CNG01580 | -0.051590072 | 0.972638293 | hypothetical protein |
| CNXL_041280 | CNG01590 | 0.208062713 | 0.717347987 | tricarboxylate transporter |
| CNXL_041290 | CNG01600 | -0.299181976 | 0.675638659 | aconitate hydratase |
| CNXL_041300 | CNG01610 | -0.303056428 | 0.531504526 | GDP-mannose transporter |
| CNXL_041310 | | -0.281551904 | 0.514179651 | hypothetical protein |
| CNXL_041320 | CNG01620 | -0.108232429 | 0.886997541 | hypothetical protein |
| CNXL_041330 | CNG01630 | -0.407565035 | 0.480560718 | transcriptional activator |
| CNXL_041340 | | 0.193449253 | 0.857954608 | calcineurin temperature suppressor |
| CNXL_041350 | CNG01640 | 0.018529312 | 0.982102702 | hypothetical protein |
| CNXL_041360 | CNG01650 | 0.249459259 | 0.602968931 | Gly-Xaa carboxypeptidase |
| CNXL_041370 | CNG01660 | -0.251965241 | 0.587056989 | ATP-dependent RNA helicase DDX41 |
| CNXL_041380 | CNG01670 | -0.379728942 | 0.43365669 | exportin-1 |
| CNXL_041390 | CNG01680 | -0.036455962 | 0.965465882 | hypothetical protein |
| CNXL_041400 | CNG01690 | -1.406107738 | 0.000130057 | hypothetical protein |
| CNXL_041410 | CNG01700 | -0.868305634 | 0.012537174 | hypothetical protein |
| CNXL_041420 | CNG01710 | -0.084942398 | 0.912929878 | hypothetical protein |
| CNXL_041430 | CNG01720 | 0.406276449 | 0.308324087 | alginate lyase |
| CNXL_041440 | CNG01730 | 0.169440408 | 0.779355929 | endochitinase |
| CNXL_041450 | CNG01740 | 0.219483169 | 0.612903776 | hypothetical protein |
| CNXL_041460 | CNG01750 | -0.17248046 | 0.781024995 | cytoplasmic protein |
| CNXL_041470 | CNG01760 | 1.618091526 | 0.070517297 | response regulator and transcription factor |
| CNXL_041480 | CNG01770 | 0.139282455 | 0.84818426 | hypothetical protein |
| CNXL_041490 | CNG01780 | -1.77737277 | 4.31689E-15 | putative compass/set1c complex subunit |
| CNXL_041500 | | -0.989165232 | 0.006327682 | hypothetical protein |
| CNXL_041510 | CNG01790 | -0.619543637 | 0.288244801 | Unknown |
| CNXL_041520 | CNG01800 | -0.204598388 | 0.790239393 | Unknown |
| CNXL_041530 | CNG01810 | 0.377176939 | 0.467310008 | mitochondrial protein |
| CNXL_041540 | CNG01820 | -0.767329726 | 0.024275521 | hypothetical protein |
| CNXL_041550 | CNG01830 | 0.35252934 | 0.478589474 | hypothetical protein |
| CNXL_041560 | CNG01850 | -0.372088441 | 0.323086046 | oxidoreductase |
| CNXL_041570 | CNG01860 | -0.110499862 | 0.942418612 | SIT4-associating protein/190 |
| CNXL_041580 | | 0.306288601 | 0.53981434 | zinc ion transporter |
| CNXL_041590 | | 0.464771019 | 0.219744161 | Unknown |
| CNXL_041600 | CNG01880 | 0.211888099 | 0.683642142 | Unknown |
| CNXL_041610 | CNG01890 | 0.388305056 | 0.383307552 | hypothetical protein |
| CNXL_041620 | CNG01900 | 0.063233497 | 0.926407741 | NAD+ diphosphatase |
| CNXL_041630 | CNG01910 | 0.046523737 | 0.944879244 | NifU-like protein c |
| CNXL_041640 | CNG01920 | -0.221758281 | 0.741426667 | hypothetical protein |
| CNXL_041650 | CNG01930 | 0.117666753 | 0.827926799 | acyl-CoA thioesterase II |
| CNXL_041660 | CNG01940 | 0.105538878 | 0.879555308 | DNA supercoiling protein |
| CNXL_041670 | CNG01950 | -0.284944911 | 0.511541134 | mitochondrial inner membrane protein |
| CNXL_041680 | CNG01960 | -0.367129994 | 0.29461261 | AAA family ATPase |
| CNXL_041690 | CNG01970 | 0.266441243 | 0.55161956 | alpha-ketoglutarate catabolism dioxygenase |
| CNXL_041700 | CNG01980 | -0.172673162 | 0.798267462 | nuclear protein |
| CNXL_041710 | CNG01990 | -1.401638577 | 4.92891E-06 | solute carrier family 25 |
| CNXL_041720 | CNG02000 | -0.690697402 | 0.550258326 | cyclin |
| CNXL_041730 | | 0.249023487 | 0.644443298 | hypothetical protein |
| CNXL_041740 | NG02020\|CNG020 | 0.329112625 | 0.391858761 | hypothetical protein |

| | | | | |
|---|---|---|---|---|
| CNXL_041750 | | 0.226426167 | 0.679255446 | Unknown |
| CNXL_041760 | CNG02040 | 0.267963052 | 0.636752734 | hypothetical protein |
| CNXL_041770 | CNG02050 | -0.399721852 | 0.435253961 | N-acetyltransferase 5 |
| CNXL_041780 | CNG02060 | 0.180064942 | 0.734939207 | hypothetical protein |
| CNXL_041790 | | -0.570354736 | 0.119973748 | hypothetical protein |
| CNXL_041800 | CNG02080 | 0.157406713 | 0.754261938 | cytoplasmic protein |
| CNXL_041810 | CNG02090 | -0.322168253 | 0.438321846 | succinyl-CoA synthetase alpha subunit |
| CNXL_041820 | | -0.094496087 | 0.881169328 | DNA replication complex GINS protein PSF1 |
| CNXL_041830 | CNG02100 | 0.439595882 | 0.202621105 | hypothetical protein |
| CNXL_041840 | CNG02110 | 0.111932373 | 0.869120128 | vacuolar protein-sorting protein BRO1 |
| CNXL_041850 | CNG02120 | 0.508385789 | 0.154689133 | HMG box factor |
| CNXL_041860 | CNG02130 | -0.102714273 | 0.923938002 | calcium-binding protein NCS-1 |
| CNXL_041870 | CNG02140 | -0.299678906 | 0.627774228 | Wee protein kinase |
| CNXL_041880 | CNG02150 | 0.07475828 | 0.903309304 | microtubule Associated protein |
| CNXL_041890 | CNG02160 | -3.434958767 | 4.68544E-48 | uridine kinase |
| CNXL_041900 | CNG02170 | 0.326224172 | 0.429445024 | C2-H2 zinc-finger transcription factor |
| CNXL_041910 | | -0.292894483 | 0.557606572 | hypothetical protein |
| CNXL_041920 | | -0.064546349 | 0.930990489 | hypothetical protein |
| CNXL_041930 | CNG02180 | 0.219280403 | 0.762884467 | hypothetical protein |
| CNXL_041940 | CNG02190 | -0.092700583 | 0.901676457 | hypothetical protein |
| CNXL_041950 | CNG02200 | -0.418127304 | 0.315126975 | hypothetical protein |
| CNXL_041960 | CNG02220 | 0.357752259 | 0.293682761 | hypothetical protein |
| CNXL_041970 | CNG02230 | -1.01286848 | 0.25401037 | phosphoglycerate kinase |
| CNXL_041980 | | 0.08539674 | 0.911130835 | hypothetical protein |
| CNXL_041990 | CNG02240 | 0.341016102 | 0.437062442 | hypothetical protein |
| CNXL_042000 | | 0.139543652 | 0.870189125 | two-component-like sensor kinase |
| CNXL_042010 | | 0.088731283 | 0.884028948 | Unknown |
| CNXL_042020 | CNG02250 | -0.455772802 | 0.24509521 | hypothetical protein |
| CNXL_042030 | CNG02260 | 0.648717596 | 0.030652868 | hypothetical protein |
| CNXL_042040 | CNG02270 | -0.225007757 | 0.730212038 | hypothetical protein |
| CNXL_042050 | CNG02280 | -0.123504101 | 0.842469317 | Unknown |
| CNXL_042060 | CNG02290 | 0.157267904 | 0.776672985 | Unknown |
| CNXL_042070 | CNG02300 | -0.141872652 | 0.840027363 | pre-mRNA-processing factor 17 |
| CNXL_042080 | CNG02310 | -0.088671416 | 0.889001755 | hypothetical protein |
| CNXL_042090 | | 1.183015333 | 3.6456E-06 | hypothetical protein |
| CNXL_042100 | 510\|CNG02330\|CN | 0.892478348 | 0.000243583 | hypothetical protein |
| CNXL_042110 | 500\|CNG02340\|CN | -1.001746548 | 0.332065103 | Unknown |
| CNXL_042120 | CNG02350 | 0.623793408 | 0.040140554 | hypothetical protein |
| CNXL_042130 | | 0.213518056 | 0.797632564 | putative translational repressor |
| CNXL_042140 | CNG02360 | 0.312533257 | 0.511551352 | hypothetical protein |
| CNXL_042150 | CNG02370 | -0.138960569 | 0.86171371 | hypothetical protein |
| CNXL_042160 | CNG02380 | -1.038620004 | 0.017217058 | tRNA |
| CNXL_042170 | CNG02390 | 0.878598249 | 0.106477482 | minichromosome maintenance protein 2 |
| CNXL_042180 | CNG02400 | 0.669414819 | 0.248605145 | flavonol synthase |
| CNXL_042190 | CNG02410 | -0.122229713 | 0.865225322 | biotin transporter |
| CNXL_042200 | CNG02420 | -0.265867133 | 0.620145083 | hypothetical protein |
| CNXL_042210 | CNG02430 | -0.359657375 | 0.456456998 | hypothetical protein |
| CNXL_042220 | CNG02440 | 0.076370453 | 0.898533825 | hypothetical protein |
| CNXL_042230 | CNG02460 | -0.4840041 | 0.112183936 | ribose-phosphate pyrophosphokinase |
| CNXL_042240 | CNG02470 | -0.172527199 | 0.77065988 | cytoplasmic protein |
| CNXL_042250 | CNG02480 | 0.176301489 | 0.773509113 | replication factor C subunit 2/4 |
| CNXL_042260 | CNG02490 | 0.485313116 | 0.19005168 | phosphatidylserine decarboxylase |
| CNXL_042270 | CNG02500 | 0.026865288 | 0.982795272 | RecQ-mediated genome instability protein 1 |
| CNXL_042280 | CNG02510 | -0.61053261 | 0.031096848 | PHD-finger protein |
| CNXL_042290 | | -2.4375115 | 0.027844163 | hypothetical protein |
| CNXL_042300 | CNG02520 | -0.736113839 | 0.015092651 | hypothetical protein |
| CNXL_042310 | CNG02530 | 0.223914137 | 0.675584168 | putative chitin synthase |
| CNXL_042320 | CNG02540 | -0.032032726 | 0.970488713 | ChAPs family protein |

| | | | | |
|---|---|---|---|---|
| CNXL_042330 | CNG02550 | 0.263872466 | 0.515010522 | hypothetical protein |
| CNXL_042340 | CNG02560 | -0.590824265 | 0.167425298 | hypothetical protein |
| CNXL_042350 | CNG02570 | 0.060219491 | 0.928118121 | UDP-glucuronic acid decarboxylase |
| CNXL_042360 | CNG02580 | -0.366148622 | 0.465784729 | vacuolar protein sorting-associated protein 9 |
| CNXL_042370 | CNG02590 | -0.18576247 | 0.752406534 | mannose-1-phosphate guanylyltransferase |
| CNXL_042380 | CNG02600 | 0.476063729 | 0.859615039 | phospholipid binding protein |
| CNXL_042390 | CNG02610 | -0.583098092 | 0.055666776 | hypothetical protein |
| CNXL_042400 | CNG02630 | -0.247220312 | 0.529621712 | AP-1 complex subunit mu-1 |
| CNXL_042410 | CNG02640 | -0.631405907 | 0.077637592 | GTP-binding protein |
| CNXL_042420 | CNG02650 | -1.021497599 | 0.166322519 | YTH domain family 2 |
| CNXL_042430 | CNG02660 | -0.222845436 | 0.656993267 | hypothetical protein |
| CNXL_042440 | CNG02670 | -0.803020806 | 0.003687758 | Unknown |
| CNXL_042450 | CNG02680 | -0.065585101 | 0.935317161 | hydroxymethylglutaryl-CoA synthase |
| CNXL_042460 | CNG02690 | 0.117951289 | 0.837168643 | nicotinamide N-methyltransferase |
| CNXL_042470 | | 0.506617296 | 0.634493899 | pre-mRNA-splicing factor CWC21 |
| CNXL_042480 | CNG02700 | -0.548456053 | 0.385883789 | Unknown |
| CNXL_042490 | CNG02710 | 0.255872715 | 0.553544499 | hypothetical protein |
| CNXL_042500 | CNG02720 | -1.244906814 | 1.14517E-05 | vesicle transport through interaction with t-SNAREs 1 |
| CNXL_042510 | CNG02730 | -0.485373616 | 0.492433582 | calcium binding protein 39 |
| CNXL_042520 | CNG02740 | -0.195905748 | 0.650209435 | hypothetical protein |
| CNXL_042530 | CNG02750 | -0.238340676 | 0.661992658 | small subunit ribosomal protein S27 |
| CNXL_042540 | CNG02760 | -0.152198775 | 0.804701377 | protein LTV1 |
| CNXL_042550 | CNG02770 | -0.175377855 | 0.796855977 | hypothetical protein |
| CNXL_042560 | CNG02780 | -0.082209922 | 0.887126434 | ribosomal RNA-processing protein 1 |
| CNXL_042570 | CNG02790 | -0.070619646 | 0.908000623 | coatomer beta subunit |
| CNXL_042580 | CNG02800 | -0.01444997 | 0.987076518 | hypothetical protein |
| CNXL_042590 | CNG02810 | 0.115752266 | 0.884028948 | putative histone acetyltransferase complex subunit |
| CNXL_042600 | CNG02820 | -0.428540052 | 0.212829513 | tyrosine specific protein phosphatase |
| CNXL_042610 | | 0.608125713 | 0.04571946 | hypothetical protein |
| CNXL_042620 | CNG02830 | -0.049866518 | 0.939400455 | hypothetical protein |
| CNXL_042630 | | 0.277161638 | 0.677367265 | pre-rRNA-processing protein |
| CNXL_042640 | CNG02840 | -0.306377895 | 0.544657787 | Unknown |
| CNXL_042650 | CNG02850 | -1.264606975 | 0.000176807 | hypothetical protein |
| CNXL_042660 | CNG02860 | 0.020122276 | 0.981240834 | STE/STE20/YSK protein kinase |
| CNXL_042670 | CNG02870 | 0.079936953 | 0.893873062 | methionine aminopeptidase |
| CNXL_042680 | CNG02880 | 0.273583154 | 0.558192148 | membrane protein |
| CNXL_042690 | CNG02890 | 0.137680923 | 0.825693641 | 28 kda golgi snare protein |
| CNXL_042700 | CNG02900 | -0.306808935 | 0.591192608 | hypothetical protein |
| CNXL_042710 | CNG02910 | -0.14024359 | 0.802877744 | hypothetical protein |
| CNXL_042720 | CNG02920 | -0.071584802 | 0.902044607 | rRNA biogenesis protein RRP36 |
| CNXL_042730 | CNG02930 | 0.840912877 | 0.0053065 | large subunit ribosomal protein L24e |
| CNXL_042740 | CNG02940 | -0.063228785 | 0.919145053 | hypothetical protein |
| CNXL_042750 | CNG02950 | -0.467860315 | 0.292245902 | glycine-rich RNA binding protein |
| CNXL_042760 | CNG02960 | 0.975215225 | 0.000352124 | dihydrodipicolinate synthetase |
| CNXL_042770 | | -0.015673653 | 0.992176872 | hypothetical protein |
| CNXL_042780 | CNG02980 | 0.018196624 | 0.983318676 | Unknown |
| CNXL_042790 | | 0.341519512 | 0.592954724 | Unknown |
| CNXL_042800 | | -0.729765885 | 0.031829912 | Unknown |
| CNXL_042810 | | 0.361874936 | 0.419388344 | Unknown |
| CNXL_042820 | | -0.99483444 | 0.440656673 | Unknown |
| CNXL_042830 | CNG03000 | 0.387087503 | 0.394293269 | Unknown |
| CNXL_042840 | CNG03010 | 0.508106029 | 0.279637638 | Unknown |
| CNXL_042850 | | 0.239836263 | 0.679138812 | inositol oxygenase |
| CNXL_042860 | CNG03020 | 0.022132991 | 0.981733632 | hypothetical protein |
| CNXL_042870 | CNG03030 | -0.541310812 | 0.372306093 | glutaryl-CoA dehydrogenase |
| CNXL_042880 | CNG03040 | -0.326954712 | 0.514351731 | solute carrier family 25 |
| CNXL_042890 | CNG03050 | -0.729714957 | 0.049310106 | transcription initiation factor TFIID subunit 2 |
| CNXL_042900 | CNG03060 | 0.111549817 | 0.857328617 | hypothetical protein |

| | | | | |
|---|---|---|---|---|
| CNXL_042910 | CNG03070 | 0.24729214 | 0.561684603 | pre-mRNA-splicing factor PRP46 |
| CNXL_042920 | | 0.139018709 | 0.83521771 | adenylosuccinate lyase |
| CNXL_042930 | CNG03080 | 0.371264578 | 0.312497815 | Unknown |
| CNXL_042940 | CNC01010 | 1.622530975 | 0.068765637 | aldehyde dehydrogenase |
| CNXL_042950 | CNG03090 | 0.017771121 | 0.983980369 | hypothetical protein |
| CNXL_042960 | CNG03100 | 0.233843444 | 0.572926427 | splicing factor 3B subunit 2 |
| CNXL_042970 | | -0.870952343 | 0.011733169 | malate dehydrogenase |
| CNXL_042980 | CNG03120 | -0.166408414 | 0.774581868 | hypothetical protein |
| CNXL_042990 | | -0.07145619 | 0.916084262 | hypothetical protein |
| CNXL_043000 | CNG03130 | -0.211254828 | 0.670734824 | Unknown |
| CNXL_043010 | CNG03140 | 0.009374164 | 0.991508881 | translation elongation factor Tu |
| CNXL_043020 | CNG03150 | -0.023731233 | 0.976496222 | myo-inositol-1 |
| CNXL_043030 | CNG03160 | 0.038990754 | 0.972638293 | zinc finger protein |
| CNXL_043040 | | 0.161540379 | 0.859882796 | large subunit ribosomal protein L6 |
| CNXL_043050 | CNG03180 | 0.099093864 | 0.881169328 | Unknown |
| CNXL_043060 | | -0.160544655 | 0.830440343 | AGC protein kinase |
| CNXL_043070 | | 0.606758166 | 0.331034626 | Unknown |
| CNXL_043080 | | NA | NA | Unknown |
| CNXL_043090 | | NA | NA | Unknown |
| CNXL_043100 | CNA03710 | 0 | NA | Unknown |
| CNXL_043110 | | 0.751023194 | 0.005798111 | Unknown |
| CNXL_043120 | | NA | NA | Unknown |
| CNXL_043130 | CNG03210 | 0 | NA | Unknown |
| CNXL_043140 | CNG03220 | -0.038866891 | 0.952330338 | Unknown |
| CNXL_043150 | CNG03230 | 0.415531065 | 0.318230668 | ADP-ribosylation factor |
| CNXL_043160 | CNG03240 | -0.086342577 | 0.885370637 | hypothetical protein |
| CNXL_043170 | CNG03250 | 0.091590421 | 0.870049852 | THO complex subunit 4 |
| CNXL_043180 | CNG03260 | -0.033104082 | 0.964414864 | rho GTPase activator |
| CNXL_043190 | | -0.195180852 | 0.701847486 | sterol 3beta-glucosyltransferase |
| CNXL_043200 | CNG03280 | 0.451915092 | 0.21296764 | Unknown |
| CNXL_043210 | CNG03290 | -0.249073549 | 0.654601802 | glucose-6-phosphate dehydrogenase |
| CNXL_043220 | CNG03300 | -0.068358621 | 0.911754274 | hypothetical protein |
| CNXL_043230 | CNG03310 | 0.105412252 | 0.849067839 | 2-nitropropane dioxygenase |
| CNXL_043240 | CNG03320 | 0.017936069 | 0.984913368 | solute carrier family 25 |
| CNXL_043250 | CNG03330 | -0.470938562 | 0.249418771 | hypothetical protein |
| CNXL_043260 | | 0.88052642 | 0.818776679 | mannosyl-oligosaccharide 1 |
| CNXL_043270 | | 0.400637898 | 0.570605968 | Unknown |
| CNXL_043280 | CNG03340 | -0.490354446 | 0.334783364 | hypothetical protein |
| CNXL_043290 | CNG03350 | -0.781655011 | 0.121068656 | dioxygenase subfamily protein |
| CNXL_043300 | CNG03360 | -0.360810908 | 0.393761434 | hypothetical protein |
| CNXL_043310 | CNG03370 | 0.005714267 | 0.994610173 | AMME syndrome candidate protein |
| CNXL_043320 | CNG03380 | -0.174778127 | 0.773509113 | THO complex subunit 1 |
| CNXL_043330 | CNG03390 | 0.426440984 | 0.206923398 | cell wall surface anchor protein |
| CNXL_043340 | CNG03400 | 1.877985343 | 4.35638E-09 | yippee-like |
| CNXL_043350 | CNG03420 | 0.824832274 | 0.004746854 | lactamase |
| CNXL_043360 | | 0.933892444 | 0.008687566 | Unknown |
| CNXL_043370 | | 0.425172342 | 0.292795277 | Unknown |
| CNXL_043380 | CNG03440 | 0.834767635 | 0.007241848 | Unknown |
| CNXL_043390 | CNG03450 | 0.211338255 | 0.675638659 | Unknown |
| CNXL_043400 | CNG03460 | 0.16360557 | 0.78519633 | homeobox transcriptional repressor |
| CNXL_043410 | CNG03480 | -0.429710285 | 0.181734454 | universal stress protein |
| CNXL_043420 | CNG03490 | -0.125397376 | 0.815653121 | hypothetical protein |
| CNXL_043430 | | -4.881469592 | 3.32862E-65 | malate dehydrogenase |
| CNXL_043440 | CNG03500 | -2.541323925 | 7.67531E-31 | hypothetical protein |
| CNXL_043450 | CNG03510 | -0.534483609 | 0.425633818 | hypothetical protein |
| CNXL_043460 | CNG03520 | -0.333508782 | 0.534813127 | RNA lariat debranching enzyme |
| CNXL_043470 | CNG03530 | 0.206199199 | 0.696516885 | large subunit ribosomal protein L29 |
| CNXL_043480 | CNG03540 | 0.247427444 | 0.633915651 | hypothetical protein |

| | | | | |
|---|---|---|---|---|
| CNXL_043490 | CNG03550 | -0.075224758 | 0.914331143 | hypothetical protein |
| CNXL_043500 | NG03560\|CNG035 | -0.379559622 | 0.427082991 | 26S protease regulatory subunit 7 |
| CNXL_043510 | CNG03580 | -0.246369532 | 0.732889168 | Unknown |
| CNXL_043520 | CNG03590 | -0.289675613 | 0.632776905 | hypothetical protein |
| CNXL_043530 | CNG03600 | 0.725579173 | 0.585297201 | tRNA |
| CNXL_043540 | CNG03610 | -0.002440094 | 0.996833113 | UV DNA damage endonuclease |
| CNXL_043550 | | -1.111345078 | 0.609589442 | forkhead domain-containing protein |
| CNXL_043560 | CNG03620 | 0.032899687 | 0.970404228 | hypothetical protein |
| CNXL_043570 | CNG03630 | -0.096646685 | 0.878994922 | SAGA-associated factor 73 |
| CNXL_043580 | CNG03640 | -0.150578861 | 0.806543943 | intersectin |
| CNXL_043590 | CNG03650 | -0.261780349 | 0.607055505 | endonuclease III |
| CNXL_043600 | | -0.716105073 | 0.024670051 | ATP-dependent RNA helicase ROK1 |
| CNXL_043610 | CNG03660 | -0.098135195 | 0.865015851 | hypothetical protein |
| CNXL_043620 | CNG03670 | 0.082614715 | 0.904401612 | DNA polymerase epsilon p12 subunit |
| CNXL_043630 | CNG03680 | -0.357900813 | 0.363022145 | adenylate cyclase |
| CNXL_043640 | CNG03690 | 0.342210744 | 0.37369301 | ubiquitin carboxyl-terminal hydrolase 4/11/15 |
| CNXL_043650 | CNG03710 | 0.096164696 | 0.865225322 | hypothetical protein |
| CNXL_043660 | CNG03720 | -0.092097482 | 0.863513463 | FAD dependent oxidoreductase |
| CNXL_043670 | | -0.437496356 | 0.46380108 | small subunit ribosomal protein S8e |
| CNXL_043680 | CNG03730 | -0.000818184 | 0.998007755 | hypothetical protein |
| CNXL_043690 | | 0.237289351 | 0.773704603 | orotidine monophosphate pyrophosphorylase |
| CNXL_043700 | CNG03750 | 0.369510353 | 0.453650498 | hypothetical protein |
| CNXL_043710 | | -1.276099376 | 0.111375561 | saccharopine dehydrogenase |
| CNXL_043720 | | 0.119954758 | 0.888190178 | Unknown |
| CNXL_043730 | CNG03760 | -0.139738387 | 0.814480545 | Unknown |
| CNXL_043740 | CNG03780 | -0.017901905 | 0.986531685 | tRNA-specific adenosine deaminase 1 |
| CNXL_043750 | CNG03790 | -0.02290993 | 0.981240834 | hypothetical protein |
| CNXL_043760 | CNG03800 | -0.234006341 | 0.602461418 | ccr4-not transcriptional regulatory complex component |
| CNXL_043770 | CNG03820 | 0.159418099 | 0.802549726 | DIL and ankyrin domain-containing protein |
| CNXL_043780 | CNG03830 | 0.1852838 | 0.697372377 | protoporphyrinogen oxidase |
| CNXL_043790 | CNG03840 | 0.086452046 | 0.950280909 | THO complex subunit 4 |
| CNXL_043800 | CNG03850 | 0.239424277 | 0.804701377 | low temperature-responsive protein |
| CNXL_043810 | CNG03860 | -0.249153588 | 0.700912881 | BUB protein kinase |
| CNXL_043820 | CNG03870 | 0.009503117 | 0.991508881 | nuclear protein |
| CNXL_043830 | | 0.774542162 | 0.244122766 | U4/U6.U5 tri-snRNP-associated protein 3 |
| CNXL_043840 | CNG03880 | -0.132246719 | 0.841905236 | hypothetical protein |
| CNXL_043850 | CNG03890 | -0.50765365 | 0.112661106 | hypothetical protein |
| CNXL_043860 | | 0.999433287 | 0.001945971 | hypothetical protein |
| CNXL_043870 | CNG03900 | 0.955260124 | 0.000304054 | hypothetical protein |
| CNXL_043880 | CNG03920 | -0.673945701 | 0.045907674 | hypothetical protein |
| CNXL_043890 | CNG03930 | 0.272968425 | 0.519060531 | endoplasmic oxidoreductin 1 |
| CNXL_043900 | CNG03940 | 0.15788791 | 0.773509113 | hypothetical protein |
| CNXL_043910 | | 0.583932391 | 0.279637638 | DNA damage-binding protein 1 |
| CNXL_043920 | CNG03950 | -1.089188969 | 0.000162376 | Unknown |
| CNXL_043930 | CNG03960 | -0.096309659 | 0.899194631 | hypothetical protein |
| CNXL_043940 | CNG03970 | -0.458483108 | 0.293580239 | serine/threonine-protein kinase |
| CNXL_043950 | | -2.239387219 | 2.04485E-07 | hypothetical protein |
| CNXL_043960 | CNG03990 | -0.0775648 | 0.917194913 | hypothetical protein |
| CNXL_043970 | CNG04000 | -0.674204698 | 0.151792461 | sulfite reductase |
| CNXL_043980 | CNG04010 | -0.915261839 | 0.050163907 | CAMK/CAMKL/Chk1 protein kinase |
| CNXL_043990 | CNG04020 | -0.30733244 | 0.512043294 | hypothetical protein |
| CNXL_044000 | | -0.020762595 | 0.995735232 | hypothetical protein |
| CNXL_044010 | CNG04030 | 0.048491402 | 0.965966884 | hypothetical protein |
| CNXL_044020 | CNG04040 | 0.425807548 | 0.35231862 | hypothetical protein |
| CNXL_044030 | CNG04050 | 0.458662005 | 0.289524368 | Unknown |
| CNXL_044040 | | -0.179033439 | 0.799344206 | vacuolar protein |
| CNXL_044050 | | 0.46967016 | 0.28004793 | hypothetical protein |
| CNXL_044060 | CNG04070 | 0.272989666 | 0.498081161 | Unknown |

| | | | | |
|---|---|---|---|---|
| CNXL_044070 | CNG04080 | -0.955624751 | 0.028845773 | DNA cross-link repair 1A protein |
| CNXL_044080 | CNG04090 | -0.3982826 | 0.290975028 | cytoplasmic protein |
| CNXL_044090 | CNG04100 | -0.081386057 | 0.914012027 | alpha-1 |
| CNXL_044100 | CNG04110 | 0.188419732 | 0.754214936 | hypothetical protein |
| CNXL_044110 | CNG04120 | 0.268236285 | 0.540001756 | hypothetical protein |
| CNXL_044120 | | 0.563900792 | 0.177355118 | ENTH domain-containing protein |
| CNXL_044130 | | -0.049604322 | 0.946988179 | Unknown |
| CNXL_044140 | | -0.118228857 | 0.890150525 | hypothetical protein |
| CNXL_044150 | CNG04130 | -0.131885015 | 0.842339591 | Unknown |
| CNXL_044160 | CNG04140 | -0.249078431 | 0.724414078 | hypothetical protein |
| CNXL_044170 | CNG04150 | 0.198009178 | 0.703985737 | calcium-binding protein |
| CNXL_044180 | CNG04160 | -0.510928007 | 0.391858761 | similar to cop9 signalosome subunit 4 |
| CNXL_044190 | CNG04170 | -0.066950032 | 0.93471488 | solute carrier family 25 |
| CNXL_044200 | CNG04180 | -0.10602015 | 0.893192733 | infection related ring finger protein |
| CNXL_044210 | CNG04190 | 0.52711917 | 0.159372565 | nuclear condensin complex protein |
| CNXL_044220 | CNG04200 | 0.468878245 | 0.292795277 | rhomboid-like protein |
| CNXL_044230 | | 1.107483193 | 0.205361326 | alpha-amylase |
| CNXL_044240 | CNG04210 | 0.469548279 | 0.179645479 | Unknown |
| CNXL_044250 | CNG04220 | 2.131228123 | 0.080505653 | peptide-methionine |
| CNXL_044260 | CNG04230 | 1.336652269 | 7.45229E-05 | hypothetical protein |
| CNXL_044270 | | 0.025282816 | 0.97242592 | hypothetical protein |
| CNXL_044280 | CNG04250 | -0.056731238 | 0.928697591 | Unknown |
| CNXL_044290 | CNG04260 | 0.784690232 | 0.026963105 | phosphatidylethanolamine N-methyltransferase |
| CNXL_044300 | CNG04270 | 0.311321414 | 0.454774776 | cullin subunit of scf-like ubiquitin ligase |
| CNXL_044310 | CNG04280 | 0.04340217 | 0.954286695 | CMGC/CDK protein kinase |
| CNXL_044320 | CNG04290 | -0.370648303 | 0.374737238 | hypothetical protein |
| CNXL_044330 | CNG04300 | 0.117404221 | 0.820111382 | hypothetical protein |
| CNXL_044340 | CNG04310 | 0.378467279 | 0.26603097 | 3-hydroxyacyl-CoA dehydrogenase |
| CNXL_044350 | | -0.311852961 | 0.770018577 | UDP-glucose sterol transferase |
| CNXL_044360 | CNG04320 | -0.030877478 | 0.969527869 | hypothetical protein |
| CNXL_044370 | CNG04330 | -0.039714865 | 0.962166617 | NAD-dependent histone deacetylase SIR2 |
| CNXL_044380 | CNG04340 | -0.213178764 | 0.690272467 | rho-type GTPase |
| CNXL_044390 | CNG04350 | -0.355941048 | 0.451926759 | transcription factor iiia |
| CNXL_044400 | CNG04360 | -0.111923568 | 0.82996137 | gamma-glutamyltransferase |
| CNXL_044410 | | 0.110758118 | 0.893955723 | small subunit ribosomal protein S23 |
| CNXL_044420 | CNG04370 | 0.193860297 | 0.695498721 | hypothetical protein |
| CNXL_044430 | CNG04380 | -0.060944142 | 0.920669589 | hypothetical protein |
| CNXL_044440 | CNG04390 | 0.3629572 | 0.396736518 | periodic tryptophan protein 2 |
| CNXL_044450 | | 0.143267931 | 0.841202784 | hypothetical protein |
| CNXL_044460 | CNG04410 | -0.148118266 | 0.838837582 | Unknown |
| CNXL_044470 | CNG04420 | -0.375607696 | 0.313087846 | hypothetical protein |
| CNXL_044480 | | 0.209555492 | 0.747332879 | alpha-1 |
| CNXL_044490 | CNG04430 | -0.156897248 | 0.830440343 | hypothetical protein |
| CNXL_044500 | CNG04440 | 0.106656971 | 0.85183218 | hypothetical protein |
| CNXL_044510 | CNG04450 | 0.053896281 | 0.941919857 | hypothetical protein |
| CNXL_044520 | CNG04460 | -0.331704412 | 0.540948602 | forkhead transcription factor |
| CNXL_044530 | CNG04470 | 1.053305886 | 0.002453981 | hypothetical protein |
| CNXL_044540 | CNG04480 | 0.864131817 | 0.024772018 | hypothetical protein |
| CNXL_044550 | | 0.262589264 | 0.598241347 | trehalose synthase |
| CNXL_044560 | | 0.78129629 | 0.519073971 | hypothetical protein |
| CNXL_044570 | CNG04530 | 0.171746785 | 0.72702678 | Unknown |
| CNXL_044580 | CNG04540 | 0.225172992 | 0.622163234 | hypothetical protein |
| CNXL_044590 | CNG04550 | -0.108927933 | 0.871700589 | ubiquitin carboxyl-terminal hydrolase 14 |
| CNXL_044600 | CNG04580 | 0.07550299 | 0.911130835 | hypothetical protein |
| CNXL_044610 | | 0.089576845 | 0.919729811 | hypothetical protein |
| CNXL_044620 | | 0.249583975 | 0.730212038 | hypothetical protein |
| CNXL_044630 | | 0.190889684 | 0.814480545 | Unknown |
| CNXL_044640 | CNG04600 | 0.032032542 | 0.972638293 | Unknown |

| | | | | |
|---|---|---|---|---|
| CNXL_044650 | CNG04610 | -0.235143301 | 0.671496054 | hypothetical protein |
| CNXL_044660 | CNG04620 | -0.14165242 | 0.872824892 | Cullin 3 |
| CNXL_044670 | CNG04630 | -0.836431773 | 0.010312142 | hypothetical protein |
| CNXL_044680 | CNG04640 | 0.027533119 | 0.977881714 | efflux protein EncT |
| CNXL_044690 | | -2.558141316 | 1.10398E-05 | Unknown |
| CNXL_044700 | | -0.978885769 | 0.022368761 | Unknown |
| CNXL_044710 | | 0.035941124 | 0.965101267 | Unknown |
| CNXL_044720 | CNG04650 | -0.057289553 | 0.92897568 | Unknown |
| CNXL_044730 | CNG04660 | -1.178621102 | 0.000122301 | cytoplasmic protein |
| CNXL_044740 | | -0.016464019 | 0.985173026 | putative chitin synthase |
| CNXL_044750 | CNG04670 | -0.216437163 | 0.770559628 | hypothetical protein |
| CNXL_044760 | CNA08350 | 1.035775963 | 0.770559628 | hypothetical protein |
| CNXL_044770 | CNG04690 | -0.29483872 | 0.667378962 | UDP-glucose 4-epimerase |
| CNXL_044780 | | -0.043287061 | 0.965966884 | Unknown |
| CNXL_044790 | CNG04710 | -0.146182513 | 0.874872772 | Unknown |
| CNXL_044800 | CNI00010 | 0.700478908 | 0.801947013 | Unknown |
| CNXL_044810 | CNI00020 | 0.297387949 | 0.572926427 | fungal specific transcription factor |
| CNXL_044820 | | 0.375610423 | 0.61517823 | phytanoyl-CoA dioxygenase |
| CNXL_044830 | CNI00030 | -0.044964944 | 0.982102702 | hypothetical protein |
| CNXL_044840 | | -0.146432671 | 0.868600207 | Unknown |
| CNXL_044850 | CNI00040 | -0.397652132 | 0.25607827 | Unknown |
| CNXL_044860 | CNI00050 | -0.493206354 | 0.271488322 | protein transporter SEC61 subunit alpha |
| CNXL_044870 | CNI00060 | -0.216508182 | 0.746537956 | tRNA-dihydrouridine synthase 3 |
| CNXL_044880 | CNI00070 | -0.004138624 | 0.996158838 | oxidoreductase |
| CNXL_044890 | CNI00080 | -0.033251221 | 0.965101267 | hypothetical protein |
| CNXL_044900 | CNI00090 | 0.001187776 | 0.997627947 | ATP-dependent bile acid transporter |
| CNXL_044910 | CNI00100 | -0.35371243 | 0.291884184 | geranylgeranyl diphosphate synthase |
| CNXL_044920 | CNI00110 | 0.185971047 | 0.823319274 | multidrug transporter |
| CNXL_044930 | CNI00120 | -0.008618659 | 0.994610173 | CTD kinase subunit gamma |
| CNXL_044940 | CNI00130 | -0.284076263 | 0.664771357 | hypothetical protein |
| CNXL_044950 | CNI00140 | -0.150228364 | 0.792018301 | cleavage stimulation factor subunit 2 |
| CNXL_044960 | CNI00150 | -0.195862519 | 0.781894338 | translocation protein SEC62 |
| CNXL_044970 | CNI00160 | -0.364325837 | 0.391858761 | mitochondrial protein required for respiration |
| CNXL_044980 | | -0.32498839 | 0.633023767 | ubiquitin-like modifier-activating enzyme ATG7 |
| CNXL_044990 | CNI00180 | -0.309699872 | 0.522210515 | hypothetical protein |
| CNXL_045000 | CNI00190 | -0.547866288 | 0.172797548 | nicotinamide mononucleotide permease |
| CNXL_045010 | | 0.20257377 | 0.733117842 | tartrate dehydrogenase |
| CNXL_045020 | CNI00210 | 0.006073171 | 0.996158838 | hypothetical protein |
| CNXL_045030 | CNI00230 | -0.310314551 | 0.430945378 | D-glycerate 3-kinase |
| CNXL_045040 | CNI00240 | 0.000700624 | 0.99817997 | translation initiation factor 2 subunit 1 |
| CNXL_045050 | CNI00250 | 0.155519639 | 0.77138893 | enoyl-CoA hydratase |
| CNXL_045060 | CNI00260 | -0.009678817 | 0.992176872 | DNA-binding protein |
| CNXL_045070 | CNI00270 | 0.021464093 | 0.988641003 | cytochrome c oxidase-assembly factor COX16 |
| CNXL_045080 | CNI00280 | 0.231195364 | 0.675638659 | hypothetical protein |
| CNXL_045090 | | -0.397984959 | 0.826575639 | hypothetical protein |
| CNXL_045100 | CNI00300 | -0.234277835 | 0.782678074 | hypothetical protein |
| CNXL_045110 | CNI00310 | -0.078302925 | 0.897988379 | glycosylphosphatidylinositol transamidase |
| CNXL_045120 | CNI00320 | -0.365681307 | 0.371037534 | zinc metalloprotease |
| CNXL_045130 | | 0.823473299 | 0.048602556 | glyceraldehyde-3-phosphate dehydrogenase |
| CNXL_045140 | CNI00350 | -0.187810281 | 0.7369155 | Unknown |
| CNXL_045150 | CNI00360 | -0.321261109 | 0.73464097 | hypothetical protein |
| CNXL_045160 | CNI00370 | -0.596864193 | 0.217175217 | oxidoreductase |
| CNXL_045170 | CNI00380 | -0.230867125 | 0.749050005 | D-aminoacylase |
| CNXL_045180 | CNI00390 | -0.165329144 | 0.774470967 | D-serine dehydratase |
| CNXL_045190 | CNI00400 | -0.283130151 | 0.615328522 | hypothetical protein |
| CNXL_045200 | CNI00410 | -0.007654711 | 0.992176872 | hypothetical protein |
| CNXL_045210 | CNI00420 | -0.611855658 | 0.113349233 | mitogen-activated protein kinase |
| CNXL_045220 | CNI00430 | -0.150035267 | 0.845033033 | DNA-directed RNA polymerase III subunit RPC2 |

| | | | | |
|---|---|---|---|---|
| CNXL_045230 | | 0.349663541 | 0.705578603 | hypothetical protein |
| CNXL_045240 | CNI00450 | -0.006436627 | 0.994987997 | Unknown |
| CNXL_045250 | CNI00460 | 0.171775612 | 0.746347427 | hypothetical protein |
| CNXL_045260 | CNI00470 | 0.911442202 | 0.443530973 | AAA-ATPase |
| CNXL_045270 | CNI00480 | 0.533862909 | 0.26013089 | hypothetical protein |
| CNXL_045280 | CNI00490 | 0.343424962 | 0.497967352 | hypothetical protein |
| CNXL_045290 | CNI00500 | -0.26694106 | 0.730212038 | hypothetical protein |
| CNXL_045300 | CNI00520 | -0.188803528 | 0.695498721 | hypothetical protein |
| CNXL_045310 | CNI00530 | -0.269634671 | 0.613272076 | guanine nucleotide-binding protein subunit alpha |
| CNXL_045320 | | 0.127495651 | 0.856186476 | hypothetical protein |
| CNXL_045330 | CNI00550 | 0.119644555 | 0.828543224 | hypothetical protein |
| CNXL_045340 | CNI00560 | -0.322483896 | 0.461869386 | hypothetical protein |
| CNXL_045350 | CNI00570 | -0.070426208 | 0.959808031 | phosphoribosylanthranilate isomerase |
| CNXL_045360 | CNI00580 | -0.114028848 | 0.866730187 | hypothetical protein |
| CNXL_045370 | CNI00600 | -0.38679229 | 0.383307552 | clathrin light chain |
| CNXL_045380 | CNI00610 | 0.428329608 | 0.24509521 | alpha-1 |
| CNXL_045390 | CNI00620 | -0.616181574 | 0.266724235 | hypothetical protein |
| CNXL_045400 | | 0.740582489 | 0.305872929 | rab GTPase activator |
| CNXL_045410 | CNI00630 | -0.217652156 | 0.742187954 | hypothetical protein |
| CNXL_045420 | CNI00640 | 0.094136088 | 0.890150525 | U1 small nuclear ribonucleoprotein C |
| CNXL_045430 | CNI00650 | -0.203527589 | 0.782518595 | ubiquitin carboxyl-terminal hydrolase 48 |
| CNXL_045440 | CNI00670 | -0.413130374 | 0.311813532 | hypothetical protein |
| CNXL_045450 | CNI00680 | 1.199591576 | 1.81051E-05 | tRNA pseudouridine |
| CNXL_045460 | CNI00690 | -0.115713051 | 0.861693974 | hypothetical protein |
| CNXL_045470 | CNI00700 | 0.218481213 | 0.696556358 | 3-ketoacyl-CoA reductase |
| CNXL_045480 | | -1.441239033 | 0.097342539 | transcription regulator |
| CNXL_045490 | CNI00710 | 0.458434925 | 0.19911361 | hypothetical protein |
| CNXL_045500 | CNI00720 | -0.584857708 | 0.085306326 | long-chain acyl-CoA synthetase |
| CNXL_045510 | CNI00730 | -1.923541243 | 1.88491E-09 | hypothetical protein |
| CNXL_045520 | CNI00740 | -0.208007136 | 0.798090976 | hypothetical protein |
| CNXL_045530 | CNI00770 | -1.941858809 | 6.71573E-10 | hypothetical protein |
| CNXL_045540 | CNI00780 | 0.536989236 | 0.428799713 | hypothetical protein |
| CNXL_045550 | CNI00790 | 0.010389322 | 0.990777629 | hypothetical protein |
| CNXL_045560 | CNI00800 | -0.237148515 | 0.645513161 | hypothetical protein |
| CNXL_045570 | CNI00810 | -1.342598207 | 0.002427313 | peptidase |
| CNXL_045580 | CNI00820 | 0.668248606 | 0.116009032 | hypothetical protein |
| CNXL_045590 | CNI00830 | -0.040653161 | 0.962926966 | Phosphoglycerate mutase-like superfamily protein |
| CNXL_045600 | CNI00840 | -0.47081077 | 0.271362906 | monocarboxylic acid transporter |
| CNXL_045610 | CNI00850 | 0.166505565 | 0.802877744 | hypothetical protein |
| CNXL_045620 | CNI00860 | 0.0806771 | 0.946605516 | membrane protein |
| CNXL_045630 | CNI00870 | -0.23195698 | 0.764121818 | FAD dependent oxidoreductase |
| CNXL_045640 | CNI00880 | -0.182517479 | 0.80500309 | haloacid dehalogenase |
| CNXL_045650 | CNI00890 | 0.533157566 | 0.244819978 | 4-aminobutyrate transaminase |
| CNXL_045660 | CNI00900 | -0.593019049 | 0.095827354 | tartrate dehydrogenase |
| CNXL_045670 | CNI00910 | 0.561670527 | 0.261738946 | succinate-semialdehyde dehydrogenase |
| CNXL_045680 | CNI00920 | -0.162202815 | 0.770342269 | hypothetical protein |
| CNXL_045690 | | 0.371456639 | 0.771763303 | small nuclear ribonucleoprotein |
| CNXL_045700 | CNI00930 | -0.114909839 | 0.892423276 | Unknown |
| CNXL_045710 | CNI00950 | -0.263833307 | 0.702776984 | Unknown |
| CNXL_045720 | CNI00960 | 0.594152272 | 0.087776413 | hypothetical protein |
| CNXL_045730 | CNI00970 | 0.386164682 | 0.281533526 | F-box protein 9 |
| CNXL_045740 | | 0.348957302 | 0.742187954 | ATP-dependent DNA helicase |
| CNXL_045750 | CNI00980 | 0.125694329 | 0.816159728 | Unknown |
| CNXL_045760 | CNI00990 | 1.946782337 | 2.87722E-10 | RNA polymerase II transcription factor |
| CNXL_045770 | CNI01000 | 0.496575248 | 0.208746699 | hypothetical protein |
| CNXL_045780 | | 0.698010821 | 0.068372808 | DNA helicase |
| CNXL_045790 | CNI01010 | 0.240924463 | 0.620718084 | hypothetical protein |
| CNXL_045800 | CNI01020 | 0.33016484 | 0.461869386 | hypothetical protein |

| | | | | |
|---|---|---|---|---|
| CNXL_045810 | CNI01030 | 0.322548491 | 0.475341202 | peroxisomal membrane protein 4 |
| CNXL_045820 | CNI01040 | -0.456338337 | 0.425879761 | hypothetical protein |
| CNXL_045830 | CNI01050 | 0.125321878 | 0.848267812 | inositol-phosphoryl ceramide synthase |
| CNXL_045840 | CNI01070 | -0.118519063 | 0.819555276 | small subunit ribosomal protein S18 |
| CNXL_045850 | CNI01080 | -0.606444525 | 0.067063478 | chromodomain-helicase-DNA-binding protein 1 |
| CNXL_045860 | CNI01090 | 0.028931144 | 0.965101267 | alpha-1 |
| CNXL_045870 | CNI01100 | 0.347126658 | 0.434588143 | large subunit ribosomal protein L19e |
| CNXL_045880 | | -0.248833941 | 0.889757615 | hypothetical protein |
| CNXL_045890 | CNI01120 | 0.030826901 | 0.963190895 | hypothetical protein |
| CNXL_045900 | CNI01130 | -0.087008481 | 0.879688103 | small subunit ribosomal protein S7e |
| CNXL_045910 | CNI01140 | 1.176953473 | 0.000367053 | splicing factor 3B subunit 1 |
| CNXL_045920 | CNI01160 | -0.69179975 | 0.027448551 | hypothetical protein |
| CNXL_045930 | CNI01170 | 0.589292451 | 0.079105 | polyadenylate-binding protein |
| CNXL_045940 | CNI01180 | -0.006874053 | 0.992176872 | thiamine pyrophosphokinase |
| CNXL_045950 | CNI01190 | 0.593734285 | 0.061142604 | V-type proton ATPase subunit B |
| CNXL_045960 | CNI01200 | 0.87406595 | 0.006301658 | hypothetical protein |
| CNXL_045970 | CNI01210 | -0.516185239 | 0.462811802 | nicotinamide riboside kinase |
| CNXL_045980 | CNI01220 | 0.110884007 | 0.869733568 | hypothetical protein |
| CNXL_045990 | CNI01230 | -0.328227561 | 0.784651013 | ferric reductase |
| CNXL_046000 | CNI01240 | -0.402357292 | 0.372044973 | hypothetical protein |
| CNXL_046010 | CNI01250 | 0.325964363 | 0.501729001 | CMGC/DYRK/DYRK2 protein kinase |
| CNXL_046020 | CNI01260 | 0.302217833 | 0.39931559 | hypothetical protein |
| CNXL_046030 | | 0.640337735 | 0.041796801 | e3 ubiquitin-protein ligase |
| CNXL_046040 | | 1.229114489 | 2.15584E-05 | Unknown |
| CNXL_046050 | | NA | NA | Unknown |
| CNXL_046060 | | -0.467299874 | 0.794943165 | Unknown |
| CNXL_046070 | | NA | NA | hypothetical protein |
| CNXL_046080 | | NA | NA | Unknown |
| CNXL_046090 | | NA | NA | Unknown |
| CNXL_046100 | | 0.116185702 | 0.855058004 | Unknown |
| CNXL_046110 | CND00850 | 0.084310016 | 0.903957128 | Unknown |
| CNXL_046120 | | -0.25688035 | 0.733041323 | Unknown |
| CNXL_046130 | | 0.036238704 | 0.981240834 | Unknown |
| CNXL_046140 | | 0.263422154 | 0.538584216 | Unknown |
| CNXL_046150 | CNI01300 | -0.990705395 | 0.000737566 | Unknown |
| CNXL_046160 | | -2.821110832 | 4.01887E-32 | alpha-ketoglutarate-dependent 2 |
| CNXL_046170 | CNI01310 | -0.316622912 | 0.396035434 | Unknown |
| CNXL_046180 | | -0.011878744 | 0.988364793 | major facilitator superfamily transporter |
| CNXL_046190 | CNI01350 | 0.258436371 | 0.55837865 | Unknown |
| CNXL_046200 | | 0.025662476 | 0.987759522 | multidrug resistance protein fnx1 |
| CNXL_046210 | CNI01360 | 1.307555867 | 0.00111032 | hypothetical protein |
| CNXL_046220 | CNI01370 | 0.196915193 | 0.824878804 | hypothetical protein |
| CNXL_046230 | | 0.53045255 | 0.380626134 | hypothetical protein |
| CNXL_046240 | CNI01380 | 0.364171757 | 0.411893985 | Unknown |
| CNXL_046250 | CNI01390 | -0.008425563 | 0.992370477 | endoplasmic reticulum protein |
| CNXL_046260 | CNI01400 | -0.44068403 | 0.411990901 | hypothetical protein |
| CNXL_046270 | CNI01410 | -0.061378593 | 0.934642219 | choline kinase |
| CNXL_046280 | CNI01420 | 0.505978747 | 0.177974568 | dynein intermediate chain |
| CNXL_046290 | CNI01430 | 0.613908083 | 0.063681258 | hypothetical protein |
| CNXL_046300 | CNI01440 | -0.094733905 | 0.899749891 | cyclin H |
| CNXL_046310 | CNI01450 | 0.349889931 | 0.420406127 | hypothetical protein |
| CNXL_046320 | CNI01460 | 0.303438695 | 0.519073971 | CCCH zinc finger protein |
| CNXL_046330 | CNI01470 | 0.612276376 | 0.071408618 | splicing factor U2AF 35 kDa subunit |
| CNXL_046340 | CNI01480 | -0.370820872 | 0.543720776 | alpha-soluble NSF attachment protein |
| CNXL_046350 | CNI01490 | 0.063110018 | 0.936086163 | small subunit ribosomal protein S10 |
| CNXL_046360 | CNI01500 | 0.750813067 | 0.010116904 | YggS family pyridoxal phosphate enzyme |
| CNXL_046370 | CNI01510 | 0.10662892 | 0.894815557 | specific RNA polymerase II transcription factor |
| CNXL_046380 | CNI01520 | -0.325104113 | 0.455991866 | hypothetical protein |

| | | | | |
|---|---|---|---|---|
| CNXL_046390 | CNI01530 | -0.196582799 | 0.757764875 | hypothetical protein |
| CNXL_046400 | | -0.34601413 | 0.508990542 | protein BFR2 |
| CNXL_046410 | CNI01550 | -0.032441778 | 0.963266917 | Unknown |
| CNXL_046420 | CNI01560 | 0.231939058 | 0.558192148 | RNA helicase |
| CNXL_046430 | CNI01570 | -0.193030982 | 0.724615229 | sterol-binding protein |
| CNXL_046440 | | 0.17131922 | 0.814220979 | DNA polymerase lambda subunit |
| CNXL_046450 | CNI01580 | 1.575310029 | 0.120404853 | Unknown |
| CNXL_046460 | | -0.279762347 | 0.717991689 | hypothetical protein |
| CNXL_046470 | CNI01590 | 0.728120219 | 0.019525849 | hypothetical protein |
| CNXL_046480 | CNI01600 | 0.454902735 | 0.24966051 | mitochondrial manganese superoxide dismutase |
| CNXL_046490 | CNI01610 | 0.962760839 | 0.019566102 | thioredoxin-like protein 4A |
| CNXL_046500 | | -0.035421019 | 0.990777629 | hypothetical protein |
| CNXL_046510 | CNI01630 | 0.189577321 | 0.764121818 | hypothetical protein |
| CNXL_046520 | CNI01640 | 0.177341374 | 0.810102246 | peptidyl-tRNA hydrolase |
| CNXL_046530 | CNI01650 | 0.195818933 | 0.702447191 | acetyltransferase |
| CNXL_046540 | CNI01660 | -0.009699449 | 0.991508881 | hypothetical protein |
| CNXL_046550 | CNI01670 | -0.360321316 | 0.496021199 | transcription initiation factor TFIIH subunit 2 |
| CNXL_046560 | CNI01680 | -0.162024358 | 0.764330585 | peptidase |
| CNXL_046570 | CNI01690 | -0.066064506 | 0.908809603 | hypothetical protein |
| CNXL_046580 | | -0.118025661 | 0.865068963 | NADH dehydrogenase |
| CNXL_046590 | CNI01730 | -0.638298165 | 0.092595672 | vacuolar protein |
| CNXL_046600 | CNI01740 | 0.365124111 | 0.351942351 | protein KRI1 |
| CNXL_046610 | CNI01540 | -0.646274883 | 0.098262608 | hexaprenyl-diphosphate synthase |
| CNXL_046620 | CNI01760 | -1.953059384 | 1.60991E-11 | Unknown |
| CNXL_046630 | CNI01770 | 0.008213269 | 0.992176872 | alginate lyase |
| CNXL_046640 | CNI01780 | 0.167215807 | 0.779355929 | DNA-directed RNA polymerase I |
| CNXL_046650 | CNI01790 | -0.571030957 | 0.148412605 | hypothetical protein |
| CNXL_046660 | CNI01800 | 0.573533504 | 0.191477648 | U3 small nucleolar RNA-associated protein 10 |
| CNXL_046670 | CNI01810 | 0.337702287 | 0.435206757 | hypothetical protein |
| CNXL_046680 | CNI01820 | 0.168348394 | 0.746765994 | CD2 antigen cytoplasmic tail-binding protein 2 |
| CNXL_046690 | | -0.106616235 | 0.862655799 | hypothetical protein |
| CNXL_046700 | CNI01830 | -0.540602307 | 0.106838284 | hypothetical protein |
| CNXL_046710 | CNI01850 | -0.194599146 | 0.749039348 | ribosome biogenesis protein MAK21 |
| CNXL_046720 | CNI01870 | -1.278401112 | 9.30627E-05 | hypothetical protein |
| CNXL_046730 | CNI01880 | -0.319795209 | 0.39417374 | hypothetical protein |
| CNXL_046740 | CNI01890 | 0.455609039 | 0.236684578 | ATP-dependent RNA helicase DBP5 |
| CNXL_046750 | CNI01900 | -0.358101665 | 0.63756045 | hypothetical protein |
| CNXL_046760 | CNI01910 | -0.407670613 | 0.370509035 | tRNA |
| CNXL_046770 | CNI01920 | -0.098428853 | 0.879688103 | hypothetical protein |
| CNXL_046780 | CNI01930 | -0.899864342 | 0.003415749 | mitochondrial inner membrane ABC transporter |
| CNXL_046790 | CNI01940 | -0.325329618 | 0.510006134 | hypothetical protein |
| CNXL_046800 | CNI01950 | -0.153843198 | 0.847311574 | TPR repeat-containing protein |
| CNXL_046810 | CNI01960 | 0.225854658 | 0.6500583 | hypothetical protein |
| CNXL_046820 | CNI01970 | 0.043035967 | 0.962194201 | exopolyphosphatase |
| CNXL_046830 | CNI01980 | 0.468743446 | 0.304899442 | hypothetical protein |
| CNXL_046840 | CNI01990 | 2.20624457 | 3.14781E-12 | putative zinc finger transcription factor |
| CNXL_046850 | CNI02010 | -0.055402107 | 0.927929791 | methylmalonate-semialdehyde dehydrogenase |
| CNXL_046860 | CNI02020 | -0.367656873 | 0.460815133 | hypothetical protein |
| CNXL_046870 | CNI02030 | -0.217557788 | 0.746537956 | chaperone |
| CNXL_046880 | CNI02040 | -0.522020336 | 0.089107701 | aspartate kinase |
| CNXL_046890 | CNI02050 | 0.320113821 | 0.339545068 | dihydrodipicolinate synthase |
| CNXL_046900 | CNI02060 | -0.661620847 | 0.027582937 | specific RNA polymerase II transcription factor |
| CNXL_046910 | CNI02070 | -0.425631589 | 0.26285894 | galactonate dehydratase |
| CNXL_046920 | CNI02080 | -0.268124992 | 0.554054079 | nicotinamide mononucleotide permease |
| CNXL_046930 | CNI02090 | 0.205064618 | 0.68904326 | hypothetical protein |
| CNXL_046940 | CNI02100 | -0.112013554 | 0.905943402 | recyclin-1 |
| CNXL_046950 | CNI02110 | 0.524165833 | 0.12340524 | hypothetical protein |
| CNXL_046960 | CNI02120 | 0.145821193 | 0.787846251 | exocyst complex component EXO84 |

| | | | | |
|---|---|---|---|---|
| CNXL_046970 | CNI02130 | 0.442248832 | 0.302177561 | cation diffusion facilitator 1 |
| CNXL_046980 | CNI02140 | 0.419910663 | 0.262705869 | single-stranded nucleic acid binding protein |
| CNXL_046990 | CNI02150 | -0.075693585 | 0.91647607 | phosphatidylinositol 4-kinase |
| CNXL_047000 | CNI02160 | -0.569096417 | 0.123440865 | hypothetical protein |
| CNXL_047010 | CNI02170 | 0.075248983 | 0.910379167 | hypothetical protein |
| CNXL_047020 | CNI02180 | -0.426898201 | 0.295911559 | large subunit ribosomal protein L31 |
| CNXL_047030 | | -0.881159137 | 0.154900292 | hypothetical protein |
| CNXL_047040 | CNI02200 | 0.019205512 | 0.984201326 | hypothetical protein |
| CNXL_047050 | CNI02210 | -0.129789657 | 0.824564918 | hypothetical protein |
| CNXL_047060 | CNI02220 | -0.248464591 | 0.61372863 | ubiquitin-conjugating enzyme E2 I |
| CNXL_047070 | CNI02230 | 0.702554225 | 0.030337262 | hypothetical protein |
| CNXL_047080 | CNI02240 | -0.380436616 | 0.595655121 | malate dehydrogenase |
| CNXL_047090 | CNI02250 | -0.15785116 | 0.769868694 | extradiol ring-cleavage dioxygenase |
| CNXL_047100 | CNI02260 | 0.169454657 | 0.806051382 | hypothetical protein |
| CNXL_047110 | CNI02270 | 1.667039581 | 1.87267E-06 | hypothetical protein |
| CNXL_047120 | CNI02280 | -0.42705305 | 0.331034626 | hypothetical protein |
| CNXL_047130 | CNI02290 | -0.344409128 | 0.375237211 | hypothetical protein |
| CNXL_047140 | CNI02300 | 0.08399484 | 0.932840267 | polysaccharide synthase |
| CNXL_047150 | CNI02310 | 0.217574888 | 0.703825663 | hypothetical protein |
| CNXL_047160 | CNI02320 | -0.049098177 | 0.963420993 | telomere length regulation protein |
| CNXL_047170 | | 0.367854178 | 0.331342508 | hypothetical protein |
| CNXL_047180 | CNI02340 | 0.544990171 | 0.140113104 | NAD+ kinase |
| CNXL_047190 | CNI02350 | 1.187593517 | 0.000890723 | hypothetical protein |
| CNXL_047200 | CNI02360 | 0.36008494 | 0.409160283 | NAD+ kinase |
| CNXL_047210 | CNI02370 | 0.393761739 | 0.393343978 | NADPH2 dehydrogenase |
| CNXL_047220 | CNI02380 | 0.116735381 | 0.853800228 | mannose-6-phosphate isomerase |
| CNXL_047230 | CNI02390 | 0.090551041 | 0.914338536 | charged multivesicular body protein 7 |
| CNXL_047240 | CNI02400 | 0.366945013 | 0.550258326 | hypothetical protein |
| CNXL_047250 | CNI02410 | 0.101701696 | 0.881811619 | hypothetical protein |
| CNXL_047260 | CNI02420 | -1.023475539 | 0.001382528 | 3-hydroxyacyl-CoA dehydrogenase |
| CNXL_047270 | CNI02440 | -0.212868588 | 0.655821512 | urate oxidase |
| CNXL_047280 | CNI02450 | -0.053961577 | 0.950999357 | vesicle transporter SFT2B |
| CNXL_047290 | CNI02460 | -0.168914815 | 0.748609863 | hypothetical protein |
| CNXL_047300 | CNI02470 | 0.3259651 | 0.39417374 | T-complex protein 1 subunit zeta |
| CNXL_047310 | CNI02480 | 0.428852581 | 0.457691748 | E3 ubiquitin-protein ligase BRE1 |
| CNXL_047320 | | 0.194589302 | 0.766858347 | hypothetical protein |
| CNXL_047330 | CNI02500 | -0.09768898 | 0.889001755 | hypothetical protein |
| CNXL_047340 | | 0.564964976 | 0.120932459 | hypothetical protein |
| CNXL_047350 | CNI02510 | -0.168064766 | 0.772848862 | hypothetical protein |
| CNXL_047360 | CNI02520 | -1.378969756 | 0.010808558 | nucleolar complex protein 3 |
| CNXL_047370 | | -1.001900283 | 0.503000429 | ribonuclease HII |
| CNXL_047380 | CNI02530 | 0.273018876 | 0.544657787 | Unknown |
| CNXL_047390 | CNI02540 | -0.2646997 | 0.539585872 | hypothetical protein |
| CNXL_047400 | CNI02550 | 0.4606724 | 0.170296094 | hypothetical protein |
| CNXL_047410 | CNI02560 | -0.011990614 | 0.990777629 | vacuolar protein sorting-associated protein |
| CNXL_047420 | CNI02570 | 0.87565098 | 0.003012304 | hypothetical protein |
| CNXL_047430 | | 0.558183999 | 0.208143452 | glycosyl-hydrolase |
| CNXL_047440 | CNI02590 | -0.080300049 | 0.889158863 | Unknown |
| CNXL_047450 | CNI02600 | -0.323792061 | 0.453096162 | hypothetical protein |
| CNXL_047460 | CNI02610 | 0.971084388 | 0.002754456 | hypothetical protein |
| CNXL_047470 | CNI02620 | -0.437438538 | 0.312160498 | Fe-S protein assembly co-chaperone HscB |
| CNXL_047480 | CNI02630 | -0.048388785 | 0.939255348 | hypothetical protein |
| CNXL_047490 | CNI02640 | -0.707885648 | 0.070095437 | DNA-directed RNA polymerase I |
| CNXL_047500 | CNI02650 | -0.493676202 | 0.213413887 | hypothetical protein |
| CNXL_047510 | CNI02660 | -0.002410291 | 0.996472611 | hypothetical protein |
| CNXL_047520 | CNI02670 | -0.112689456 | 0.885370637 | integral membrane protein |
| CNXL_047530 | CNI02680 | -0.107231033 | 0.842339591 | mitogen-activated protein |
| CNXL_047540 | CNI02690 | -0.178262015 | 0.789423788 | hypothetical protein |

| | | | | |
|---|---|---|---|---|
| CNXL_047550 | CNI02700 | -0.248367416 | 0.631371223 | hypothetical protein |
| CNXL_047560 | | -1.243556216 | 0.000961171 | hypothetical protein |
| CNXL_047570 | CNI02710 | 0.226376502 | 0.602461418 | Unknown |
| CNXL_047580 | CNI02720 | 0.286498813 | 0.517010563 | antiviral helicase SKI2 |
| CNXL_047590 | CNI02730 | -0.263799627 | 0.779355929 | pre-mRNA-splicing factor CWC2 |
| CNXL_047600 | CNI02740 | -0.319979441 | 0.498121256 | hypothetical protein |
| CNXL_047610 | CNI02750 | -0.428481822 | 0.372306093 | hypothetical protein |
| CNXL_047620 | CNI02760 | -0.289536544 | 0.572409295 | methylthioribose-1-phosphate isomerase |
| CNXL_047630 | CNI02770 | 0.612348302 | 0.083397364 | hypothetical protein |
| CNXL_047640 | CNM00430 | 0.492810066 | 0.241429802 | Unknown |
| CNXL_047650 | CNI02780 | 0.074112102 | NA | Unknown |
| CNXL_047660 | CNI02790 | -0.055088949 | 0.932840267 | hypothetical protein |
| CNXL_047670 | CNI02800 | -2.043806083 | 1.49781E-21 | vacuolar protein |
| CNXL_047680 | | -0.240259727 | 0.847311574 | tartrate transporter |
| CNXL_047690 | | -0.450019837 | 0.777403833 | hypothetical protein |
| CNXL_047700 | CNI02820 | -1.331517375 | 5.96771E-08 | Unknown |
| CNXL_047710 | | 0.255547775 | 0.808041494 | hypothetical protein |
| CNXL_047720 | CNI02830 | -0.109632532 | 0.861048266 | hypothetical protein |
| CNXL_047730 | CNI02840 | -0.942379669 | 0.171890332 | nuclear pore complex protein Nup107 |
| CNXL_047740 | CNI02850 | -0.136313074 | 0.82996137 | NEK protein kinase |
| CNXL_047750 | CNI02860 | -0.189704347 | 0.703818084 | ATP-dependent rRNA helicase RRP3 |
| CNXL_047760 | CNI02870 | 0.038666857 | 0.965101267 | hypothetical protein |
| CNXL_047770 | CNI02880 | 0.621527806 | 0.035013288 | hypothetical protein |
| CNXL_047780 | CNI02890 | 1.156486732 | 0.000256137 | oxysterol-binding protein |
| CNXL_047790 | CNI02900 | -0.909147279 | 0.212526095 | protein phosphatase methylesterase 1 |
| CNXL_047800 | CNI02910 | 0.632220164 | 0.42639861 | hypothetical protein |
| CNXL_047810 | CNI02920 | 0.109148098 | 0.842908869 | hypothetical protein |
| CNXL_047820 | CNI02930 | 0.178904724 | 0.735323021 | small nuclear ribonucleoprotein E |
| CNXL_047830 | CNI02940 | 0.146020697 | 0.865062469 | homoserine kinase |
| CNXL_047840 | CNI02950 | -0.200192852 | 0.70590796 | kinetochore protein Mis12/MTW1 |
| CNXL_047850 | CNI02960 | -0.043609014 | 0.953095191 | nucleosome assembly complex protein |
| CNXL_047860 | | -0.091148131 | 0.894815557 | ariadne-1 |
| CNXL_047870 | CNI02970 | 0.194960882 | 0.709467249 | Unknown |
| CNXL_047880 | CNI02980 | -0.218651336 | 0.742187954 | hypothetical protein |
| CNXL_047890 | CNI02990 | 0.890767165 | 0.013949228 | protein kinase A |
| CNXL_047900 | CNI03010 | -0.385784421 | 0.581095252 | hypothetical protein |
| CNXL_047910 | | 0.289217719 | 0.606726524 | hypothetical protein |
| CNXL_047920 | CNI03030 | 0.260098002 | 0.615328522 | hypothetical protein |
| CNXL_047930 | CNI03040 | 1.48816856 | 2.03656E-05 | smad nuclear-interacting protein 1 |
| CNXL_047940 | CNI03050 | -0.089136149 | 0.87784482 | hypothetical protein |
| CNXL_047950 | CNI03060 | -0.013557979 | 0.991351411 | histone H1/5 |
| CNXL_047960 | CNI03070 | -0.176201996 | 0.799344206 | holo-[acyl-carrier protein] synthase |
| CNXL_047970 | | -0.031821857 | 0.991133859 | WD-repeat protein |
| CNXL_047980 | CNI03080 | -0.155153154 | 0.746765994 | hypothetical protein |
| CNXL_047990 | CNI03090 | 0.313310636 | 0.616439959 | hypothetical protein |
| CNXL_048000 | CNI03100 | 0.260151355 | 0.623472961 | transcription factor C subunit 6 |
| CNXL_048010 | CNI03110 | -0.607350085 | 0.134780142 | hypothetical protein |
| CNXL_048020 | CNI03120 | -0.244747363 | 0.645909437 | AAA-peroxin |
| CNXL_048030 | CNI03130 | -0.353622905 | 0.397048245 | hypothetical protein |
| CNXL_048040 | | -0.009551616 | 0.995735232 | hypothetical protein |
| CNXL_048050 | CNI03140 | 0.034350223 | 0.964414864 | Unknown |
| CNXL_048060 | CNI03150 | 0.02930289 | 0.976006716 | similar to cop9 signalosome subunit 7 |
| CNXL_048070 | CNI03160 | -0.133000899 | 0.85816747 | hypothetical protein |
| CNXL_048080 | CNI03170 | -0.282057058 | 0.560646266 | lysine-tRNA ligase |
| CNXL_048090 | CNI03180 | -0.06891053 | 0.930010301 | large subunit ribosomal protein L15 |
| CNXL_048100 | CNI03190 | -0.257105612 | 0.615520877 | RNA-binding domain-containing protein |
| CNXL_048110 | CNI03200 | 0.242064784 | 0.576167679 | hypothetical protein |
| CNXL_048120 | CNI03210 | 0.064333854 | 0.928560253 | hypothetical protein |

| | | | | |
|---|---|---|---|---|
| CNXL_048130 | CNI03220 | 0.657992755 | 0.085306326 | hypothetical protein |
| CNXL_048140 | CNI03230 | -0.173273445 | 0.749315941 | hypothetical protein |
| CNXL_048150 | CNI03250 | -0.06727132 | 0.927185687 | hypothetical protein |
| CNXL_048160 | CNI03260 | 0.162474824 | 0.756597645 | GPI-anchored wall transfer protein 1 |
| CNXL_048170 | CNI03270 | -0.314165096 | 0.408746917 | O-methyltransferase |
| CNXL_048180 | CNI03280 | -0.205065533 | 0.683642142 | hypothetical protein |
| CNXL_048190 | CNI03290 | -0.04398785 | 0.960169622 | DNA topoisomerase 1 |
| CNXL_048200 | CNI03300 | -0.298793663 | 0.568146069 | SCYL protein kinase |
| CNXL_048210 | CNI03310 | -0.875172909 | 0.023352546 | phosphoribosylformylglycinamidine synthase |
| CNXL_048220 | CNI03320 | -0.001624615 | 0.996833113 | hypothetical protein |
| CNXL_048230 | CNI03330 | 0.902267034 | 0.004051186 | protein transporter SEC13 |
| CNXL_048240 | CNI03340 | 0.470401462 | 0.176747531 | O-methyltransferase |
| CNXL_048250 | CNI03350 | -0.113076037 | 0.890150525 | actin binding protein |
| CNXL_048260 | CNI03370 | -0.296635267 | 0.602461418 | CMGC/DYRK/YAK protein kinase |
| CNXL_048270 | CNI03380 | 0.362134407 | 0.510006134 | hypothetical protein |
| CNXL_048280 | CNI03390 | 0.14438656 | 0.836925435 | hypothetical protein |
| CNXL_048290 | CNI03400 | -0.279956658 | 0.57855354 | C2 domain-containing protein |
| CNXL_048300 | CNI03410 | -0.194347681 | 0.764121818 | hypothetical protein |
| CNXL_048310 | CNI03420 | 0.021617162 | 0.983237538 | cytosolic Fe-S cluster assembly factor NAR1 |
| CNXL_048320 | CNI03430 | 0.039828719 | 0.959619907 | hypothetical protein |
| CNXL_048330 | CNI03440 | 0.291678011 | 0.498081161 | transcription elongation factor SPT4 |
| CNXL_048340 | | 0.900307257 | 0.002464817 | bromodomain-containing protein 8 |
| CNXL_048350 | CNI03460 | 0.315109095 | 0.434283583 | hypothetical protein |
| CNXL_048360 | CNI03470 | 1.002250238 | 0.000293389 | homeobox protein cut-like |
| CNXL_048370 | CNI03480 | 0.492399081 | 0.115833705 | ataxin-3 |
| CNXL_048380 | CNI03490 | -0.447736375 | 0.169743402 | hypothetical protein |
| CNXL_048390 | CNI03510 | -0.451125675 | 0.238575211 | MFS transporter |
| CNXL_048400 | CNI03520 | -0.281859087 | 0.531504526 | Unknown |
| CNXL_048410 | CNI03530 | 0.201321008 | 0.771763303 | nuclear RNA export factor 1/2 |
| CNXL_048420 | CNI03540 | -0.931873467 | 0.098349258 | hypothetical protein |
| CNXL_048430 | CNI03560 | -0.562013858 | 0.306455537 | signal transducer |
| CNXL_048440 | | -0.022229332 | 0.986620478 | sulfate adenylyltransferase |
| CNXL_048450 | CNI03590 | -0.384675448 | 0.253171735 | hypothetical protein |
| CNXL_048460 | CNI03600 | -0.399756153 | 0.593301465 | phosphoenolpyruvate carboxykinase |
| CNXL_048470 | CNI03610 | 0.699689948 | 0.008467838 | hypothetical protein |
| CNXL_048480 | CNI03620 | 0.85856244 | 0.005074634 | lactoylglutathione lyase |
| CNXL_048490 | CNI03630 | 0.003934479 | 0.996158838 | ATP-dependent DNA helicase II subunit 1 |
| CNXL_048500 | CNI03640 | -0.303380033 | 0.547704491 | 6-phosphofructo-2-kinase/fructose-2 |
| CNXL_048510 | | 0.811011982 | 0.419557544 | protein SDA1 |
| CNXL_048520 | CNI03650 | -0.000932336 | 0.998287442 | hypothetical protein |
| CNXL_048530 | | 0.207488468 | 0.747814538 | hypothetical protein |
| CNXL_048540 | CNI03670 | 0.204292869 | 0.678606775 | Unknown |
| CNXL_048550 | CNI03680 | 0.496001211 | 0.259443105 | U4/U6.U5 tri-snRNP-associated protein 1 |
| CNXL_048560 | | -0.112263362 | 0.838837582 | hypothetical protein |
| CNXL_048570 | CNI03710 | 0.062967931 | 0.934876799 | Unknown |
| CNXL_048580 | CNI03720 | -0.579108197 | 0.330689507 | hydroxyethylthiazole kinase |
| CNXL_048590 | CNI03730 | 0.362900092 | 0.479536106 | hypothetical protein |
| CNXL_048600 | CNI03740 | -0.179811008 | 0.862678265 | hypothetical protein |
| CNXL_048610 | CNI03750 | 0.278061526 | 0.564998733 | hypothetical protein |
| CNXL_048620 | CNI03760 | 0.020361061 | 0.981851244 | hypothetical protein |
| CNXL_048630 | CNI03770 | -0.033673732 | 0.967512149 | hypothetical protein |
| CNXL_048640 | CNI03780 | -0.338099504 | 0.417866535 | pyridoxal phosphate phosphatase phospho2 |
| CNXL_048650 | CNI03790 | 0.061096994 | 0.931708838 | hypothetical protein |
| CNXL_048660 | CNI03800 | -0.034299943 | 0.980730226 | peroxisomal 2 |
| CNXL_048670 | CNI03810 | 0.022273669 | 0.982102702 | hypothetical protein |
| CNXL_048680 | CNI03820 | -0.275714033 | 0.573713031 | hypothetical protein |
| CNXL_048690 | CNI03830 | -0.076352969 | 0.907466014 | cytoplasmic protein |
| CNXL_048700 | CNI03840 | -1.344988098 | 4.21224E-06 | hypothetical protein |

| | | | | |
|---|---|---|---|---|
| CNXL_048710 | CNI03850 | 0.077650389 | 0.91248949 | guanine nucleotide exchange factor |
| CNXL_048720 | CNI03860 | -0.457240571 | 0.433846913 | hypothetical protein |
| CNXL_048730 | CNI03870 | 0.193585324 | 0.747569937 | endochitinase |
| CNXL_048740 | CNI03880 | 0.951208772 | 0.005720333 | hypothetical protein |
| CNXL_048750 | | 0.7346108 | 0.134439284 | ubiquitin thioesterase OTUB1 |
| CNXL_048760 | CNI03890 | 0.773043219 | 0.031679745 | Unknown |
| CNXL_048770 | CNI03900 | 0.06771874 | 0.946116137 | hypothetical protein |
| CNXL_048780 | CNI03910 | 0.150896176 | 0.802313068 | hypothetical protein |
| CNXL_048790 | CNI03920 | 0.17603613 | 0.809974479 | transmembrane protein |
| CNXL_048800 | CNI03930 | 0.284877808 | 0.680746566 | hypothetical protein |
| CNXL_048810 | CNI03940 | -0.394734891 | 0.35648622 | hypothetical protein |
| CNXL_048820 | CNI03950 | 0.142532497 | 0.801947013 | hypothetical protein |
| CNXL_048830 | CNI03960 | -0.034983844 | 0.965966884 | hypothetical protein |
| CNXL_048840 | CNI03970 | 0.003527395 | 0.996158838 | ADP-ribosylation factor |
| CNXL_048850 | CNI03980 | -0.321163485 | 0.55949398 | H/ACA ribonucleoprotein complex subunit 1 |
| CNXL_048860 | CNI03990 | -1.227482487 | 0.072401784 | translation initiation factor eIF-2B subunit gamma |
| CNXL_048870 | CNI04000 | -0.009895025 | 0.992176872 | hypothetical protein |
| CNXL_048880 | CNI04020 | 0.101671156 | 0.889757615 | E3 ubiquitin-protein ligase NRDP1 |
| CNXL_048890 | CNI04030 | 0.088548478 | 0.908809603 | hypothetical protein |
| CNXL_048900 | CNI04040 | 0.652615836 | 0.055622246 | ubiquinone biosynthesis monooxygenase COQ6 |
| CNXL_048910 | CNI04050 | 0.306704133 | 0.535957791 | hypothetical protein |
| CNXL_048920 | CNI04060 | -0.63175952 | 0.12598044 | YEATS domain-containing protein 4 |
| CNXL_048930 | CNI04070 | 0.284235182 | 0.580447333 | mitochondrial genome maintenance protein |
| CNXL_048940 | CNI04080 | -0.046023758 | 0.949084374 | exodeoxyribonuclease III |
| CNXL_048950 | CNI04090 | 0.791594826 | 0.008504453 | leucyl aminopeptidase |
| CNXL_048960 | CNI04100 | -0.220438744 | 0.757814488 | syntaxin 8 |
| CNXL_048970 | CNI04110 | -0.122923979 | 0.826907872 | hypothetical protein |
| CNXL_048980 | CNI04120 | 0.630136913 | 0.03885852 | CAMK/CAMK1 protein kinase |
| CNXL_048990 | CNI04130 | 0.370560748 | 0.444610739 | hypothetical protein |
| CNXL_049000 | | 1.346051929 | 1.41696E-05 | acyl-CoA thioesterase II |
| CNXL_049010 | CNI04150 | -0.49155659 | 0.445590137 | metalloendopeptidase |
| CNXL_049020 | CNI04160 | -0.226717459 | 0.663234353 | cytosine permease |
| CNXL_049030 | CNI04170 | -0.004388321 | 0.996158838 | hypothetical protein |
| CNXL_049040 | CNI04180 | -0.153735123 | 0.810102246 | DNA ligase 1 |
| CNXL_049050 | CNI04190 | -0.20177506 | 0.843283188 | hypothetical protein |
| CNXL_049060 | | 0.326650532 | 0.919952257 | hypothetical protein |
| CNXL_049070 | CNI04200 | 0.310391253 | 0.756597645 | Unknown |
| CNXL_049080 | | NA | NA | hypothetical protein |
| CNXL_049090 | | -0.651680879 | 0.056803616 | Unknown |
| CNXL_049100 | CNI04230 | -0.113591057 | 0.861760083 | putative cytosine-purine permease |
| CNXL_049110 | | 1.92828591 | 0.488456653 | pre-mRNA-splicing factor CWC24 |
| CNXL_049120 | CNI04240 | 0.234948392 | 0.771763303 | Unknown |
| CNXL_049130 | CNI04250 | 0.449543144 | 0.253992342 | Unknown |
| CNXL_049140 | CNI04260 | -0.221496172 | 0.667369865 | galactose dehydrogenase |
| CNXL_049150 | CNI04270 | 0.046004266 | 0.949084374 | hypothetical protein |
| CNXL_049160 | CNI04280 | -0.468638365 | 0.306455537 | RNA polymerase II subunit A domain phosphatase |
| CNXL_049170 | CNI04290 | -0.397310829 | 0.438321846 | rho guanyl-nucleotide exchange factor |
| CNXL_049180 | | 0.119697843 | 0.863513463 | CMGC/CDK/CRK7 protein kinase |
| CNXL_049190 | | 0.57565607 | 0.27463373 | hypothetical protein |
| CNXL_049200 | CNI04320 | -0.40861892 | 0.43363696 | Unknown |
| CNXL_049210 | CNI04330 | -0.716938792 | 0.375342854 | hypothetical protein |
| CNXL_049220 | CNI04340 | -0.533293271 | 0.182133927 | cell division control protein |
| CNXL_049230 | CNI04350 | 0.190170055 | 0.680057336 | small subunit ribosomal protein S0 |
| CNXL_049240 | CNI04360 | 0.875296904 | 0.001830484 | hypothetical protein |
| CNXL_049250 | CNI04370 | -0.09889136 | 0.872623756 | oxidoreductase |
| CNXL_049260 | CNI04380 | 0.877811824 | 0.009114985 | lectin |
| CNXL_049270 | | 0.546609033 | 0.766858347 | glutathione S-transferase |
| CNXL_049280 | CNI04400 | 0.750210637 | 0.012180411 | hypothetical protein |

| | | | | |
|---|---|---|---|---|
| CNXL_049290 | CNI04410 | 0.464601429 | 0.170296094 | pyruvate dehydrogenase kinase |
| CNXL_049300 | CNI04420 | 0.975349652 | 0.004388236 | hypothetical protein |
| CNXL_049310 | | 0.013020189 | 0.988641003 | hypothetical protein |
| CNXL_049320 | | 2.487613021 | 0.061495256 | Unknown |
| CNXL_049330 | | -1.440226686 | NA | hypothetical protein |
| CNXL_049340 | | 1.484927388 | NA | Unknown |
| CNXL_049350 | CNL06830 | 0.021128 | 0.983980369 | Unknown |
| CNXL_049360 | CNH03130 | -0.0850244 | 0.927441884 | transposase subfamily |
| CNXL_049370 | | -0.059819111 | 0.960513489 | oxidoreductase |
| CNXL_049380 | CNL06810 | 0.011833332 | 0.991141666 | Unknown |
| CNXL_049390 | CNL06800 | -0.060336296 | 0.938435008 | hypothetical protein |
| CNXL_049400 | CNL06790 | 0.135658625 | 0.825693641 | condensin complex subunit 2 |
| CNXL_049410 | CNL06780 | 1.292506971 | 0.000158673 | peroxin-12 |
| CNXL_049420 | CNL06770 | -0.328870187 | 0.582028001 | hypothetical protein |
| CNXL_049430 | CNL06760 | 0.256275248 | 0.587056989 | hypothetical protein |
| CNXL_049440 | CNL06750 | -1.025169996 | 0.002245653 | hypothetical protein |
| CNXL_049450 | CNL06740 | 0.054918665 | 0.964414864 | hypothetical protein |
| CNXL_049460 | CNL06730 | -0.068449625 | 0.938435008 | uroporphyrinogen-III C-methyltransferase |
| CNXL_049470 | CNL06720 | -0.377941609 | 0.422414274 | CBS domain-containing protein |
| CNXL_049480 | | 1.13555725 | 0.190357372 | hypothetical protein |
| CNXL_049490 | CNL06700 | 0.39787116 | 0.477835242 | Unknown |
| CNXL_049500 | CNL06690 | 0.112516499 | 0.907858192 | hypothetical protein |
| CNXL_049510 | CNL06680 | 0.21903331 | 0.676782389 | hypothetical protein |
| CNXL_049520 | CNL06670 | 0.332126403 | 0.457034932 | high-affinity nicotinic acid transporter |
| CNXL_049530 | CNL06660 | -0.275298087 | 0.664461114 | tubulin beta chain |
| CNXL_049540 | CNL06650 | -0.245638453 | 0.597680886 | hypothetical protein |
| CNXL_049550 | CNL06640 | 0.098037562 | 0.873324574 | hypothetical protein |
| CNXL_049560 | CNL06630 | -0.289188397 | 0.539585872 | 3-deoxy-7-phosphoheptulonate synthase |
| CNXL_049570 | CNL06620 | 1.009275955 | 0.001797137 | ADP-ribosylation factor 6 |
| CNXL_049580 | CNL06610 | -0.473922373 | 0.238702905 | hypothetical protein |
| CNXL_049590 | CNL06600 | 0.382978912 | 0.310343274 | hypothetical protein |
| CNXL_049600 | CNL06590 | 1.050092397 | 0.0292062 | oxidoreductase |
| CNXL_049610 | CNL06580 | -0.409718859 | 0.342591459 | Unknown |
| CNXL_049620 | CNL06570 | 0.594877467 | 0.08178911 | hypothetical protein |
| CNXL_049630 | CNL06560 | 0.116377618 | 0.848823288 | ubiquitin fusion degradation protein 1 |
| CNXL_049640 | | 0.065888281 | 0.918848255 | syntaxin 16 |
| CNXL_049650 | CNL06540 | -0.106074704 | 0.91726998 | hypothetical protein |
| CNXL_049660 | CNL06530 | -0.885533816 | 0.003498907 | Unknown |
| CNXL_049670 | CNL06520 | -3.848689779 | 4.35006E-63 | hypothetical protein |
| CNXL_049680 | CNL06510 | 0.180949224 | 0.771763303 | hypothetical protein |
| CNXL_049690 | CNL06500 | 0.26317407 | 0.647099831 | transcriptional regulator |
| CNXL_049700 | CNL06490 | -0.321944055 | 0.434588143 | amidohydrolase 3 |
| CNXL_049710 | CNL06480 | 0.170460383 | 0.793995856 | ABC transporter |
| CNXL_049720 | CNL06470 | -0.030319756 | 0.978689402 | hypothetical protein |
| CNXL_049730 | CNL06460 | -0.511463523 | 0.111496645 | hypothetical protein |
| CNXL_049740 | | -1.595370973 | 0.032580388 | UDP-glucose 6-dehydrogenase |
| CNXL_049750 | | -2.617058307 | 0.000464796 | Unknown |
| CNXL_049760 | | 0.229387472 | 0.906729846 | Unknown |
| CNXL_049770 | | 0.015133815 | 0.992176872 | Unknown |
| CNXL_049780 | | 0.01693701 | 0.992176872 | Unknown |
| CNXL_049790 | | -1.084421025 | 0.304723814 | Unknown |
| CNXL_049800 | CNL06440 | -0.241268062 | 0.576230883 | Unknown |
| CNXL_049810 | CNL06430 | 0.048089185 | 0.948457186 | hypothetical protein |
| CNXL_049820 | CNL06420 | -0.052559085 | 0.939400455 | syntaxin-binding protein 1 |
| CNXL_049830 | CNL06410 | -0.591229095 | 0.1518292 | hypothetical protein |
| CNXL_049840 | CNL06400 | -0.043664632 | 0.963190895 | Unknown |
| CNXL_049850 | | 0.876708066 | 0.078941525 | hypothetical protein |
| CNXL_049860 | CNL06390 | -0.464932046 | 0.240269875 | hypothetical protein |

| | | | | |
|---|---|---|---|---|
| CNXL_049870 | CNL06380 | -0.17149659 | 0.75817335 | zuotin |
| CNXL_049880 | CNL06370 | 0.588402875 | 0.059791942 | pre-mRNA branch site protein p14 |
| CNXL_049890 | | -0.223859453 | 0.654548423 | phytanoyl-CoA dioxygenase |
| CNXL_049900 | CNL06350 | 0.338713007 | 0.39417374 | hypothetical protein |
| CNXL_049910 | CNL06340 | 1.345454392 | 5.94099E-06 | U6 snRNA-associated Sm-like protein LSm1 |
| CNXL_049920 | :NI04210\|CNL0633 | -0.22234412 | 0.771822468 | catalase 1 similar to spore-specific catalases |
| CNXL_049930 | | -0.713243978 | 0.078954247 | putative cytosine-purine permease |
| CNXL_049940 | CNL06310 | -0.366818172 | 0.443697582 | hypothetical protein |
| CNXL_049950 | CNL06300 | -0.354944381 | 0.370462381 | phosphatidylinositol transfer protein |
| CNXL_049960 | | -0.17583157 | 0.803866606 | nascent polypeptide-associated complex subunit alpha |
| CNXL_049970 | CNL06290 | 0.186470668 | 0.743437511 | prefoldin subunit 1 |
| CNXL_049980 | CNL06270 | -0.463572355 | 0.273564602 | hypothetical protein |
| CNXL_049990 | CNL06260 | 0.321900657 | 0.843689865 | Gly-Xaa carboxypeptidase |
| CNXL_050000 | CNL06250 | 0.50230689 | 0.188305292 | Unknown |
| CNXL_050010 | CNL06240 | -0.136158543 | 0.825693641 | protein FRG1 |
| CNXL_050020 | CNL06230 | -0.919596634 | 0.002302734 | gamma-glutamyl phosphate reductase |
| CNXL_050030 | CNL06220 | -0.268625331 | 0.522756874 | hypothetical protein |
| CNXL_050040 | CNL06210 | 0.144757201 | 0.779355929 | hypothetical protein |
| CNXL_050050 | CNL06200 | 0.255174378 | 0.638320737 | hypothetical protein |
| CNXL_050060 | CNL06190 | 0.379359052 | 0.34648341 | signal recognition particle subunit SRP14 |
| CNXL_050070 | CNL06180 | -1.028122739 | 0.002114604 | aldo-keto reductase |
| CNXL_050080 | CNL06170 | -0.310872333 | 0.449659853 | class III aminotransferase |
| CNXL_050090 | | -0.032303555 | 0.981569994 | cytoplasmic protein |
| CNXL_050100 | | 0.232227084 | 0.724256813 | Unknown |
| CNXL_050110 | | -0.09210635 | 0.923938002 | Unknown |
| CNXL_050120 | CNL06150 | 0.066021421 | 0.946391322 | hypothetical protein |
| CNXL_050130 | CNL06140 | 0.155248133 | 0.767511661 | hypothetical protein |
| CNXL_050140 | | 0.16075503 | 0.746537956 | hypothetical protein |
| CNXL_050150 | CNL06120 | -0.914685607 | 0.04589775 | hypothetical protein |
| CNXL_050160 | CNL06110 | -0.247597288 | 0.658936841 | hypothetical protein |
| CNXL_050170 | CNL06100 | 0.165161639 | 0.770719952 | GTP-binding protein LepA |
| CNXL_050180 | | -0.27628318 | 0.78519633 | ULK/ULK protein kinase |
| CNXL_050190 | CNL06090 | -0.536312198 | 0.305871123 | Unknown |
| CNXL_050200 | CNL06080 | -0.008252886 | 0.992176872 | hypothetical protein |
| CNXL_050210 | | 0.24152184 | 0.61517823 | translation initiation factor eIF-2B subunit beta |
| CNXL_050220 | CNL06070 | -2.252521226 | 3.67715E-17 | hypothetical protein |
| CNXL_050230 | CNL06060 | -0.210609791 | 0.65237946 | integral membrane protein |
| CNXL_050240 | CNL06050 | 0.102235455 | 0.899291941 | hypothetical protein |
| CNXL_050250 | CNL06040 | -0.321034975 | 0.455221545 | iron donor protein CyaY |
| CNXL_050260 | CNL06030 | 0.016889294 | 0.983011297 | hypothetical protein |
| CNXL_050270 | CNL06020 | -0.010119747 | 0.991351411 | RNP domain-containing protein |
| CNXL_050280 | CNL06010 | -0.655891043 | 0.056048793 | catalase 4 |
| CNXL_050290 | CNL06000 | -0.251861342 | 0.62045122 | hypothetical protein |
| CNXL_050300 | CNL05980 | 0.073523576 | 0.918142047 | Unknown |
| CNXL_050310 | CNL05970 | 0.425229138 | 0.295034424 | hypothetical protein |
| CNXL_050320 | CNL05960 | 1.532295225 | 0.001204856 | NAD-binding Rossmann fold oxidoreductase |
| CNXL_050330 | CNL05950 | 0.486874049 | 0.253992342 | hypothetical protein |
| CNXL_050340 | CNL05940 | -0.036521605 | 0.965101267 | hypothetical protein |
| CNXL_050350 | CNL05930 | 0.173989633 | 0.765840893 | putative maltose o-acetyltransferase |
| CNXL_050360 | CNL05920 | -0.003161578 | 0.996472611 | L-xylulose reductase |
| CNXL_050370 | CNL05910 | -0.253799087 | 0.652840051 | Unknown |
| CNXL_050380 | CNL05900 | 0.14178225 | 0.800850342 | amine oxidase |
| CNXL_050390 | CNL05890 | 0.435791422 | 0.264189715 | mango esterase |
| CNXL_050400 | CNL05880 | -0.139361671 | 0.825693641 | monoamine oxidase |
| CNXL_050410 | CNL05870 | -0.003924652 | 0.996158838 | cysteine synthase |
| CNXL_050420 | | 0.295543201 | 0.629861961 | hypothetical protein |
| CNXL_050430 | CNL05860 | 0.063303051 | 0.93760092 | zinc finger protein 830 |
| CNXL_050440 | CNL05850 | 0.140037911 | 0.81510329 | 3-oxoacid CoA-transferase |

| | | | | |
|---|---|---|---|---|
| CNXL_050450 | | 0.44639133 | 0.382775862 | hypothetical protein |
| CNXL_050460 | CNL05840 | 0.478499576 | 0.125443586 | Unknown |
| CNXL_050470 | CNL05830 | 0.074036745 | 0.914338536 | hypothetical protein |
| CNXL_050480 | CNL05820 | 0.197889912 | 0.819835748 | ubiquitin-conjugating enzyme family protein |
| CNXL_050490 | CNL05810 | -0.247595254 | 0.671905355 | ferredoxin-NADP+ reductase |
| CNXL_050500 | CNL05800 | -0.77672903 | NA | hypothetical protein |
| CNXL_050510 | CNL05790 | -0.582034096 | 0.331381363 | hypothetical protein |
| CNXL_050520 | CNL05780 | -0.402616893 | 0.678724355 | hypothetical protein |
| CNXL_050530 | | 1.709963089 | 0.51244137 | hypothetical protein |
| CNXL_050540 | CNL05770 | -0.450808559 | 0.193854728 | hypothetical protein |
| CNXL_050550 | CNL05760 | -0.497617484 | 0.271524617 | NADH-ubiquinone oxidoreductase subunit 8 |
| CNXL_050560 | CNL05750 | -0.069683634 | 0.920674786 | carnitine acetyltransferase |
| CNXL_050570 | | 0.041384204 | 0.962706186 | hypothetical protein |
| CNXL_050580 | | 0.133705235 | 0.85924574 | hypothetical protein |
| CNXL_050590 | | -1.359547313 | 0.697146261 | hypothetical protein |
| CNXL_050600 | CNL05720 | 0.23749003 | 0.594358319 | hypothetical protein |
| CNXL_050610 | CNL05710 | -0.460943499 | 0.166033664 | protein mgr2 |
| CNXL_050620 | CNL05700 | -0.102609517 | 0.879531297 | insulysin |
| CNXL_050630 | CNL05690 | 0.062786586 | 0.935785373 | hypothetical protein |
| CNXL_050640 | CNL05680 | -0.532145734 | 0.314745548 | ADP-ribosylation factor GTPase-activating protein |
| CNXL_050650 | NC01610\|CNL056[illegible] | 0.478111342 | 0.099275787 | hypothetical protein |
| CNXL_050660 | | -0.449605636 | 0.428799713 | Unknown |
| CNXL_050670 | CNL05630 | 0.417473661 | 0.287017678 | hypothetical protein |
| CNXL_050680 | CNL05620 | 0.079833709 | 0.915837202 | hypothetical protein |
| CNXL_050690 | CNL05610 | 0.283643061 | 0.576230883 | hypothetical protein |
| CNXL_050700 | CNL05600 | -0.673675705 | 0.054894119 | GTPase |
| CNXL_050710 | CNL05590 | 0.980356338 | 0.002260756 | pyruvate dehydrogenase E1 component subunit beta |
| CNXL_050720 | CNL05580 | 0.31860863 | 0.756349942 | hypothetical protein |
| CNXL_050730 | CNL05570 | 0.293907327 | 0.519431801 | hypothetical protein |
| CNXL_050740 | CNL05560 | -0.651030517 | 0.103122512 | delta-pyrroline-5-carboxylate |
| CNXL_050750 | CNL05550 | -0.897876418 | 0.290745908 | ste/ste11/ssk protein kinase |
| CNXL_050760 | | 0.3624688 | 0.802313068 | hypothetical protein |
| CNXL_050770 | CNL05540 | 0.146475037 | 0.82996137 | hypothetical protein |
| CNXL_050780 | CNL05530 | -1.183123138 | 0.001547769 | hypothetical protein |
| CNXL_050790 | CNL05520 | 0.221425124 | 0.595655121 | hypothetical protein |
| CNXL_050800 | CNL05510 | -0.542172672 | 0.169598851 | GTP-binding protein ypt1 |
| CNXL_050810 | CNL05500 | -0.112105344 | 0.847718093 | ubiquinol-cytochrome c reductase subunit 10 |
| CNXL_050820 | | -0.272707406 | 0.844850118 | sulfite reductase |
| CNXL_050830 | CNL05490 | 0.379880385 | 0.41607574 | sulfite reductase |
| CNXL_050840 | CNL05470 | 0.200567876 | 0.656993267 | endopeptidase |
| CNXL_050850 | CNL05460 | 0.015898051 | 0.985595171 | alanine-glyoxylate transaminase |
| CNXL_050860 | CNL05450 | -0.30427859 | 0.61372863 | hypothetical protein |
| CNXL_050870 | | 0.144526536 | 0.814220979 | solute carrier family 20 |
| CNXL_050880 | CNL05440 | 1.093000819 | 0.002607525 | hypothetical protein |
| CNXL_050890 | | -0.098761316 | 0.924607253 | glycosyl hydrolase |
| CNXL_050900 | CNL05410 | 0.172175809 | 0.752406534 | Unknown |
| CNXL_050910 | | -0.961188133 | NA | tRNA '-O-ribosylphosphate transferase |
| CNXL_050920 | CNL05400 | 0.004133334 | 0.995735232 | hypothetical protein |
| CNXL_050930 | CNL05390 | 0.40352635 | 0.304500885 | calcofluor white hypersensitive protein |
| CNXL_050940 | CNL05370 | -0.257545867 | 0.638320737 | phosphodiesterase |
| CNXL_050950 | CNL05360 | 0.462649759 | 0.253506448 | translation initiation factor 2D |
| CNXL_050960 | CNL05350 | 0.549944733 | 0.068985512 | esterase/lipase |
| CNXL_050970 | CNL05340 | -0.418447208 | 0.483451258 | phospholipase A-2-activating protein |
| CNXL_050980 | | -0.077162728 | 0.914338536 | deoxyhypusine hydroxylase |
| CNXL_050990 | CNL05310 | -0.397738837 | 0.291577712 | Unknown |
| CNXL_051000 | | 0.221503283 | 0.825693641 | mitochondrial protein |
| CNXL_051010 | CNL05290 | 0.93824262 | 0.002843529 | hypothetical protein |
| CNXL_051020 | CNL05280 | 0.211653248 | 0.735323021 | quinone oxidoreductase |

| | | | | |
|---|---|---|---|---|
| CNXL_051030 | | -0.374167258 | 0.68927994 | hypothetical protein |
| CNXL_051040 | CNL05270 | 1.165214765 | 0.00030896 | hypothetical protein |
| CNXL_051050 | CNL05260 | -0.878945837 | 0.314390734 | pod-specific dehydrogenase SAC25 |
| CNXL_051060 | CNL05250 | 1.248688446 | 0.00015128 | histone deacetylase 1/2 |
| CNXL_051070 | CNL05240 | 0.159145579 | 0.816809805 | YjeF family protein |
| CNXL_051080 | CNL05220 | 1.041150336 | NA | ubiquitin-like protein |
| CNXL_051090 | CNL05210 | 0.121841622 | 0.857274599 | hypothetical protein |
| CNXL_051100 | CNL05200 | -0.3915759 | 0.422414274 | ENTH domain-containing protein c |
| CNXL_051110 | CNL05190 | -0.265082671 | 0.637722771 | U3 small nucleolar RNA-associated protein 12 |
| CNXL_051120 | CNL05180 | 0.214115657 | 0.758169261 | DNA repair protein Rad5 |
| CNXL_051130 | CNL05170 | 0.460924072 | 0.547836899 | transcription initiation factor TFIIF subunit beta |
| CNXL_051140 | CNL05160 | -0.793139914 | 0.027448551 | CAMK protein kinase |
| CNXL_051150 | | 0.325286666 | 0.436980626 | eukaryotic translation initiation factor 3 subunit l |
| CNXL_051160 | CNL05150 | -0.237307598 | 0.637722771 | hypothetical protein |
| CNXL_051170 | CNL05140 | -1.029066981 | 0.000381527 | prefoldin subunit 4 |
| CNXL_051180 | | 0.620521026 | 0.108539336 | CBS and PB1 domain-containing protein |
| CNXL_051190 | CNL05120 | -0.041336998 | 0.960936711 | Unknown |
| CNXL_051200 | CNL05110 | -0.094648884 | 0.879555308 | small subunit ribosomal protein S13 |
| CNXL_051210 | CNL05100 | 0.278135977 | 0.566713427 | hypothetical protein |
| CNXL_051220 | | -0.143082717 | 0.840398313 | Unknown |
| CNXL_051230 | CNL05080 | 0.087670276 | 0.874620297 | Unknown |
| CNXL_051240 | CNL05070 | -0.171648259 | 0.773509113 | hypothetical protein |
| CNXL_051250 | | 0.680904132 | 0.145012655 | aldehyde dehydrogenase |
| CNXL_051260 | CNL05050 | 0.109506794 | 0.867654918 | peroxisomal copper amine oxidase |
| CNXL_051270 | CNL05040 | 0.941081664 | 0.001526497 | sarcosine oxidase |
| CNXL_051280 | CNL05030 | -0.191710551 | 0.670362199 | DNA polymerase mu subunit |
| CNXL_051290 | CNL05020 | -0.180571185 | 0.806051382 | hypothetical protein |
| CNXL_051300 | CNL05010 | 0.766613492 | 0.021890324 | hypothetical protein |
| CNXL_051310 | | 0.952271837 | 0.026024968 | GabA permease |
| CNXL_051320 | CNL05000 | 0.299923915 | 0.437055965 | Unknown |
| CNXL_051330 | CNL04990 | -0.545035076 | 0.219149239 | nicotinate phosphoribosyltransferase |
| CNXL_051340 | CNL04980 | -0.069689632 | 0.915884007 | hypothetical protein |
| CNXL_051350 | CNL04970 | 0.20930247 | 0.778921493 | homoserine O-acetyltransferase |
| CNXL_051360 | CNL04960 | 0.319764929 | 0.445982809 | hypothetical protein |
| CNXL_051370 | CNL04950 | -0.386703519 | 0.485459548 | dynamin-like GTPase |
| CNXL_051380 | CNL04940 | -0.465245819 | 0.418683626 | diphosphomevalonate decarboxylase |
| CNXL_051390 | CNL04930 | -0.129428129 | 0.790239393 | hypothetical protein |
| CNXL_051400 | CNL04920 | 0.03022714 | 0.969527869 | hypothetical protein |
| CNXL_051410 | | -1.103373415 | 0.001856011 | hypothetical protein |
| CNXL_051420 | CNL04910 | -0.045773924 | 0.962926966 | hypothetical protein |
| CNXL_051430 | CNL04900 | 0.571699025 | 0.060462212 | hypothetical protein |
| CNXL_051440 | CNL04890 | -0.381503856 | 0.434283583 | hypothetical protein |
| CNXL_051450 | | -0.996922223 | 0.063292368 | cytochrome c oxidase subunit 5b |
| CNXL_051460 | CNL04880 | -0.335371115 | 0.430213834 | hypothetical protein |
| CNXL_051470 | CNL04870 | -0.553485504 | 0.236068427 | ornithine-oxo-acid transaminase |
| CNXL_051480 | CNL04860 | -0.465777979 | 0.166322519 | putative calcium-transporting ATPase |
| CNXL_051490 | CNL04850 | -0.32641652 | 0.417588807 | cation-transporting ATPase 13A1 |
| CNXL_051500 | CNL04840 | 0.472622084 | 0.207376888 | ER lumen protein retaining receptor |
| CNXL_051510 | CNL04830 | 0.26555916 | 0.623172865 | glucan 1 |
| CNXL_051520 | CNL04820 | -0.293795675 | 0.532490033 | putative UDP-galactose transporter |
| CNXL_051530 | | 0.745989128 | 0.115944756 | methylenetetrahydrofolate reductase |
| CNXL_051540 | CNL04810 | -0.220564437 | 0.719557989 | Unknown |
| CNXL_051550 | CNL04800 | 0.063311357 | 0.939255348 | hypothetical protein |
| CNXL_051560 | CNL04780 | 0.007554385 | 0.992981679 | hypothetical protein |
| CNXL_051570 | CNL04770 | 0.255460206 | 0.633677259 | beta-type carbonic anhydrase |
| CNXL_051580 | | 0.113436821 | 0.847923235 | DNA helicase |
| CNXL_051590 | CNL04760 | -0.206767352 | 0.818704891 | DNA-directed RNA polymerase II subunit RPB9 |
| CNXL_051600 | CNL04750 | -0.15874678 | 0.779355929 | hypothetical protein |

| | | | | |
|---|---|---|---|---|
| CNXL_051610 | CNL04740 | 0.002138523 | 0.996833113 | beta-1 |
| CNXL_051620 | CNL04730 | -0.018871984 | 0.982102702 | protoheme IX farnesyltransferase |
| CNXL_051630 | CNL04720 | -0.149163592 | 0.809427305 | ATP-binding cassette transporter |
| CNXL_051640 | CNL04710 | 0.159293545 | 0.753446444 | U3 small nucleolar RNA-associated protein 11 |
| CNXL_051650 | CNL04700 | -0.057229935 | 0.939255348 | phospholipid:diacylglycerol acyltransferase |
| CNXL_051660 | CNL04690 | -0.096444433 | 0.861349479 | hypothetical protein |
| CNXL_051670 | CNL04680 | 0.447817766 | 0.36733274 | membrane fraction protein |
| CNXL_051680 | CNL04670 | -0.924177993 | 0.000963619 | protein tyrosine phosphatase |
| CNXL_051690 | CNL04660 | 0.197602926 | 0.687568439 | hypothetical protein |
| CNXL_051700 | CNL04650 | 0.125034524 | 0.841905236 | hypothetical protein |
| CNXL_051710 | | -0.330449261 | 0.537650729 | Unknown |
| CNXL_051720 | | 0.70405945 | 0.614760546 | Unknown |
| CNXL_051730 | | 0.199012822 | 0.751284159 | hypothetical protein |
| CNXL_051740 | CNL04640 | 0.208653492 | 0.683642142 | Unknown |
| CNXL_051750 | CNL04630 | -0.021228725 | 0.981240834 | hypothetical protein |
| CNXL_051760 | CNL04620 | -0.399216427 | 0.323308793 | V-type proton ATPase subunit E |
| CNXL_051770 | CNL04610 | -0.03054377 | 0.969527869 | exoribonuclease II |
| CNXL_051780 | CNL04600 | 0.474103397 | 0.315144538 | splicing factor 3B subunit 4 |
| CNXL_051790 | CNL04590 | -0.266399845 | 0.553544499 | hypothetical protein |
| CNXL_051800 | CNL04580 | 1.56519524 | 0.165266145 | helicase SWR1 |
| CNXL_051810 | CNL04570 | -0.166144288 | 0.73464097 | hypothetical protein |
| CNXL_051820 | CNL04560 | -0.653594605 | 0.088452632 | L-lactate dehydrogenase |
| CNXL_051830 | CNL04550 | 0.034120229 | 0.965004754 | hypothetical protein |
| CNXL_051840 | CNL04540 | -0.091848522 | 0.890147007 | CCR4-NOT transcription complex subunit 7/8 |
| CNXL_051850 | CNL04530 | 1.079351965 | 0.000715453 | myosin-1 |
| CNXL_051860 | CNL04520 | -0.099288934 | 0.905935139 | DNA-3-methyladenine glycosylase II |
| CNXL_051870 | CNL04510 | -0.152000599 | 0.810331995 | centromeric protein E |
| CNXL_051880 | CNL04500 | 0.200903934 | 0.754214936 | hypothetical protein |
| CNXL_051890 | CNL04490 | -0.326816251 | 0.519652797 | hypothetical protein |
| CNXL_051900 | CNL04480 | 0.290364089 | 0.664511524 | DNA polymerase kappa subunit |
| CNXL_051910 | CNL04470 | -0.505778753 | 0.25401037 | hypothetical protein |
| CNXL_051920 | CNL04460 | 0.149175987 | 0.78809694 | ubiquinol-cytochrome c reductase core subunit 2 |
| CNXL_051930 | CNL04450 | -0.648857844 | 0.078923725 | hypothetical protein |
| CNXL_051940 | | -0.336292985 | 0.482842388 | white-collar transcription factor |
| CNXL_051950 | CNL04430 | 0.004019157 | 0.995994152 | hypothetical protein |
| CNXL_051960 | CNL04420 | -0.693294382 | 0.865614892 | hypothetical protein |
| CNXL_051970 | CNL04410 | 0.259826346 | 0.586181879 | glycosyl transferase family 8 protein |
| CNXL_051980 | CNL04400 | -0.203494968 | 0.675025545 | hypothetical protein |
| CNXL_051990 | CNL04390 | -0.333973265 | 0.664110764 | hypothetical protein |
| CNXL_052000 | CNL04380 | -0.446600083 | 0.417866535 | hypothetical protein |
| CNXL_052010 | CNL04370 | 0.087226107 | 0.889757615 | hypothetical protein |
| CNXL_052020 | CNL04360 | -0.601410591 | 0.272050531 | hypothetical protein |
| CNXL_052030 | | 0.180187931 | NA | hypothetical protein |
| CNXL_052040 | CNL04350 | -0.308250585 | 0.443530973 | Unknown |
| CNXL_052050 | CNL04340 | -0.256903407 | 0.675584168 | hypothetical protein |
| CNXL_052060 | | -0.231499247 | 0.846112002 | lipoyl synthase |
| CNXL_052070 | CNL04310 | 0.11479539 | 0.886480005 | Unknown |
| CNXL_052080 | CNL04300 | -0.241274468 | 0.627988525 | Unknown |
| CNXL_052090 | CNL04290 | 0.100210014 | 0.865225322 | ubiquitin-conjugation factor E4 B |
| CNXL_052100 | CNL04280 | -0.376228159 | 0.364205207 | nuclear cap-binding protein subunit 2 |
| CNXL_052110 | | -0.08015218 | 0.904506055 | NADH dehydrogenase |
| CNXL_052120 | | -0.144127079 | 0.809974479 | Unknown |
| CNXL_052130 | CNL04260 | -0.189838839 | 0.660663885 | Unknown |
| CNXL_052140 | CNL04250 | 0.126651138 | 0.830440343 | chaperone DnaK |
| CNXL_052150 | CNL04240 | 0.316206345 | 0.432073936 | pyridoxal kinase |
| CNXL_052160 | CNL04230 | -0.480603416 | 0.163482578 | DNA mismatch repair protein MSH4 |
| CNXL_052170 | | 0.463205292 | 0.798976008 | SET domain-containing protein 6 |
| CNXL_052180 | | -0.031380613 | 0.965966884 | hypothetical protein |

| | | | | |
|---|---|---|---|---|
| CNXL_052190 | CNL04220 | -0.681190029 | 0.417588807 | Unknown |
| CNXL_052200 | CNL04210 | 0 | NA | Unknown |
| CNXL_052210 | | 2.993792313 | NA | Unknown |
| CNXL_052220 | CNL04140 | 0.298494592 | 0.430945378 | hypothetical protein |
| CNXL_052230 | CNL04130 | -0.061859848 | 0.936507583 | hypothetical protein |
| CNXL_052240 | CNL04120 | 0.245116203 | 0.632998683 | CAMK protein kinase |
| CNXL_052250 | CNL04110 | 0.257642406 | 0.551291645 | hypothetical protein |
| CNXL_052260 | CNL04100 | 0.20097669 | 0.696473798 | adenylyl cyclase-associated protein |
| CNXL_052270 | | -0.48853345 | 0.597680886 | hypothetical protein |
| CNXL_052280 | | -0.23519627 | 0.822527149 | Unknown |
| CNXL_052290 | CNL04090 | 0.006567016 | 0.994453452 | Unknown |
| CNXL_052300 | CNL04080 | 0.192255982 | 0.681560436 | Tor-like phosphatidylinositol 3 kinase |
| CNXL_052310 | CNL04070 | -0.06306749 | 0.927441884 | histone H2A.Z |
| CNXL_052320 | CNL04060 | 0.094572868 | 0.896147015 | transcription factor |
| CNXL_052330 | CNL04050 | -0.150641436 | 0.852609559 | hypothetical protein |
| CNXL_052340 | CNL04040 | -0.095020055 | 0.879531297 | hypothetical protein |
| CNXL_052350 | CNL04030 | 0.089840618 | 0.899602567 | bud site selection protein 31 |
| CNXL_052360 | CNL04020 | -0.117171982 | 0.865815732 | hypothetical protein |
| CNXL_052370 | CNL04010 | -0.246241531 | 0.669639767 | hypothetical protein |
| CNXL_052380 | CNL03990 | 0.543281996 | 0.137913772 | U3 small nucleolar ribonucleoprotein LCP5 |
| CNXL_052390 | CNL03980 | 0.04012028 | 0.963420993 | stomatin family protein |
| CNXL_052400 | CNL03970 | -0.116768994 | 0.814220979 | sentrin/sumo-specific protease |
| CNXL_052410 | CNL03950 | -0.205640064 | 0.689993365 | nuclear protein |
| CNXL_052420 | CNL03940 | -0.311639169 | 0.566540169 | hypothetical protein |
| CNXL_052430 | CNL03930 | 0.137836615 | 0.78519633 | hypothetical protein |
| CNXL_052440 | CNL03920 | 0.616060501 | 0.038540808 | protein BMH2 |
| CNXL_052450 | | 0.260453883 | 0.615520877 | hypothetical protein |
| CNXL_052460 | | 0.341770884 | 0.314745548 | Unknown |
| CNXL_052470 | | 0.220834956 | 0.825693641 | hypothetical protein |
| CNXL_052480 | CNL03910 | 0.860479755 | 0.006462387 | Unknown |
| CNXL_052490 | CNL03900 | 0.007881765 | 0.992176872 | cytoplasmic protein |
| CNXL_052500 | CNL03890 | -0.026195591 | 0.972638293 | signal recognition particle subunit SRP68 |
| CNXL_052510 | CNL03880 | -0.170587872 | 0.809974479 | U6 snRNA-associated Sm-like protein LSm7 |
| CNXL_052520 | NH03870\|CNL038[illegible] | -0.46759719 | 0.232845005 | hypothetical protein |
| CNXL_052530 | NH03860\|CNL038[illegible] | 0.874752788 | 0.144013827 | xylulokinase |
| CNXL_052540 | | -0.867611412 | 0.002917755 | hypothetical protein |
| CNXL_052550 | NH03850\|CNL038[illegible] | 0.519633477 | 0.13614515 | hypothetical protein |
| CNXL_052560 | NH03840\|CNL038[illegible] | -0.049151977 | 0.946144993 | hypothetical protein |
| CNXL_052570 | CNL03830 | 0.167518383 | 0.746765994 | 1-acyl-sn-glycerol-3-phosphate acyltransferase |
| CNXL_052580 | NH03820\|CNL038[illegible] | -0.840512537 | 0.017777565 | clathrin binding protein |
| CNXL_052590 | NH03810\|CNL038[illegible] | -0.597294436 | 0.344051686 | hypothetical protein |
| CNXL_052600 | CNH03800 | 0.170116905 | 0.755040408 | FMN adenylyltransferase |
| CNXL_052610 | NH03790\|CNL037[illegible] | 0.462624994 | 0.125429289 | telomere maintenance protein |
| CNXL_052620 | NH03780\|CNL037[illegible] | -0.220728937 | 0.701325477 | hypothetical protein |
| CNXL_052630 | NH03770\|CNL037[illegible] | 0.337054904 | 0.437598632 | chaperone regulator |
| CNXL_052640 | NH03760\|CNL037[illegible] | -0.650867067 | 0.233174199 | hypothetical protein |
| CNXL_052650 | NH03750\|CNL037[illegible] | 0.099469007 | 0.866730187 | solute carrier family 35 |
| CNXL_052660 | NH03740\|CNL037[illegible] | -0.182688055 | 0.753507015 | hypothetical protein |
| CNXL_052670 | NH03730\|CNL037[illegible] | -0.844743789 | 0.27463373 | catalase 2 |
| CNXL_052680 | | 0.489956424 | 0.221624826 | glucose-methanol-choline oxidoreductase |
| CNXL_052690 | NH03710\|CNL037[illegible] | 0.655060391 | 0.085306326 | solute carrier family 35 |
| CNXL_052700 | NH03700\|CNL037[illegible] | 0.848718935 | 0.021054491 | hypothetical protein |
| CNXL_052710 | NH03690\|CNL036[illegible] | -0.077392426 | 0.903386376 | glutamate decarboxylase |
| CNXL_052720 | CNL03680 | 0.096838386 | 0.872866501 | hypothetical protein |
| CNXL_052730 | NH03670\|CNL036[illegible] | -1.054608043 | 0.001998138 | hypothetical protein |
| CNXL_052740 | NH03660\|CNL036[illegible] | -3.389548279 | 4.16607E-41 | Unknown |
| CNXL_052750 | CNH03650 | -1.184000045 | 2.42335E-06 | alpha-amylase AmyA |
| CNXL_052760 | CNH03640 | 0.017509803 | 0.982102702 | hypothetical protein |

| | | | | |
|---|---|---|---|---|
| CNXL_052770 | CNH03630 | -0.208108155 | 0.637722771 | membrane protein |
| CNXL_052780 | CNH03620 | -0.191953572 | 0.777814879 | NADH dehydrogenase |
| CNXL_052790 | CNH03610 | -0.172425193 | 0.725078077 | hypothetical protein |
| CNXL_052800 | CNH03600 | -0.228405525 | 0.62364132 | 20S proteasome subunit alpha 7 |
| CNXL_052810 | CNH03590 | 0.199484008 | 0.719248355 | tRNA pseudouridine13 synthase |
| CNXL_052820 | CNH03580 | 0.03613341 | 0.965101267 | transcription initiation factor TFIIE subunit beta |
| CNXL_052830 | CNH03570 | 0.252061212 | 0.582678739 | hypothetical protein |
| CNXL_052840 | CNH03560 | -0.915077016 | 0.042098547 | 1 |
| CNXL_052850 | | -0.296004227 | 0.742187954 | STE/STE20/YSK protein kinase |
| CNXL_052860 | CNH03550 | 0.382367473 | 0.33306974 | hypothetical protein |
| CNXL_052870 | CNH03540 | 0.156668447 | 0.810224824 | hypothetical protein |
| CNXL_052880 | NH03510\|CNH035 | -0.255022088 | 0.523236531 | Unknown |
| CNXL_052890 | CNH03500 | -0.558540316 | 0.738145185 | Unknown |
| CNXL_052900 | CNH03490 | 0.01853891 | 0.986381348 | hypothetical protein |
| CNXL_052910 | CNH03480 | -0.530490705 | 0.19334914 | hypothetical protein |
| CNXL_052920 | | -1.541008208 | 2.74145E-05 | f-box protein of scf ubiquitin-ligase complex |
| CNXL_052930 | CNH03470 | 0.111159223 | 0.865068963 | Unknown |
| CNXL_052940 | CNH03460 | 0.011455901 | 0.991792083 | aminophospholipid translocase |
| CNXL_052950 | CNH03450 | -0.114190953 | 0.920669589 | solute carrier family 25 |
| CNXL_052960 | CNH03440 | 0.002724359 | 0.996472611 | hypothetical protein |
| CNXL_052970 | | -0.175389022 | 0.809974479 | hypothetical protein |
| CNXL_052980 | | -0.214800816 | 0.779380569 | hypothetical protein |
| CNXL_052990 | CNH03430 | -0.780981051 | 0.083099375 | hypothetical protein |
| CNXL_053000 | CNH03420 | 0.059944497 | 0.942888075 | HUS1 checkpoint protein |
| CNXL_053010 | | 0.823658153 | 0.001963151 | hypothetical protein |
| CNXL_053020 | CNH03410 | 0.378582875 | 0.365333703 | hypothetical protein |
| CNXL_053030 | CNH03400 | -0.506872938 | 0.234350217 | hypothetical protein |
| CNXL_053040 | CNH03380 | 0.502932499 | 0.123247228 | hypothetical protein |
| CNXL_053050 | CNH03370 | -0.060510047 | 0.939255348 | glycogenin glucosyltransferase |
| CNXL_053060 | | -0.127750561 | 0.840322617 | hypothetical protein |
| CNXL_053070 | CNH03360 | -0.353330679 | 0.576167679 | hypothetical protein |
| CNXL_053080 | CNH03350 | 0.679118205 | 0.12453995 | metallochaperone |
| CNXL_053090 | CNH03330 | -0.675954 | 0.145962194 | hypothetical protein |
| CNXL_053100 | CNH03320 | 0.757220112 | 0.029865442 | hypothetical protein |
| CNXL_053110 | CNH03310 | 0.085042572 | 0.901676457 | oxidoreductase |
| CNXL_053120 | CNH03300 | -0.360183487 | 0.46632372 | mitochondrial carrier protein |
| CNXL_053130 | CNH03290 | -2.286784591 | 1.25011E-18 | coronin |
| CNXL_053140 | CNH03280 | -0.215541972 | 0.68904326 | amine oxidase |
| CNXL_053150 | CNH03270 | -0.401894441 | 0.469828881 | isocitrate lyase |
| CNXL_053160 | CNH03260 | -0.056770087 | 0.929378909 | hypothetical protein |
| CNXL_053170 | CNH03250 | 1.027592005 | 0.001450786 | beta-catenin-like protein 1 |
| CNXL_053180 | CNH03240 | 0.059241811 | 0.936783812 | hypothetical protein |
| CNXL_053190 | CNH03230 | -0.29876653 | 0.490315559 | nipsnap family protein |
| CNXL_053200 | CNH03220 | 0.67962768 | 0.593397075 | arsenite-resistant protein ASR2 |
| CNXL_053210 | CNH03200 | -0.421797302 | 0.421537718 | hypothetical protein |
| CNXL_053220 | CNH03190 | -0.043326129 | 0.949641665 | hypothetical protein |
| CNXL_053230 | CNH03180 | 0.406451264 | 0.522860075 | hypothetical protein |
| CNXL_053240 | CNH03170 | 0.041701126 | 0.953095191 | taurine catabolism dioxygenase TauD |
| CNXL_053250 | CNH03160 | -0.325831444 | 0.496021199 | inositol oxygenase |
| CNXL_053260 | CNH03150 | 0.067529775 | 0.920254084 | phytanoyl-CoA dioxygenase |
| CNXL_053270 | CNH03140 | -0.269215677 | 0.650629033 | L-mandelate dehydrogenase |
| CNXL_053280 | CNH03130 | -0.10930559 | 0.932765996 | hypothetical protein |
| CNXL_053290 | CNH03120 | 0.634924543 | 0.170587304 | oxidoreductase |
| CNXL_053300 | | 0.404774482 | 0.679255446 | transposase subfamily |
| CNXL_053310 | CNH03100 | 0.431234118 | 0.3535948 | Unknown |
| CNXL_053320 | CNH03090 | 0.620062643 | 0.116094051 | hypothetical protein |
| CNXL_053330 | CNH03080 | 0.824128228 | 0.134036805 | sugar transporter |
| CNXL_053340 | CNJ03420 | 2.885783419 | NA | Unknown |

| | | | | |
|---|---|---|---|---|
| CNXL_053350 | CNJ03430 | -0.522225694 | 0.303135759 | Unknown |
| CNXL_053360 | CNH03040 | 0.041173067 | 0.965966884 | Unknown |
| CNXL_053370 | | -0.64990066 | 0.301688 | hypothetical protein |
| CNXL_053380 | | -2.332113113 | 5.88045E-19 | Unknown |
| CNXL_053390 | CNJ00020 | 0.907056319 | 0.002697382 | hypothetical protein |
| CNXL_053400 | | 1.018713869 | 0.004611447 | Unknown |
| CNXL_053410 | CNJ00030 | 0.912525335 | 0.00548193 | Unknown |
| CNXL_053420 | CNJ00050 | 0.110768768 | 0.838813737 | cysteine-type peptidase |
| CNXL_053430 | CNJ00060 | 0.001688807 | 0.996833113 | hypothetical protein |
| CNXL_053440 | CNJ00070 | 0.374365562 | 0.392781441 | beta-flanking protein |
| CNXL_053450 | CNJ00080 | -0.169846443 | 0.733117842 | drug transporter |
| CNXL_053460 | CNJ00090 | -0.11832318 | 0.837479784 | ferro-O2-oxidoreductase |
| CNXL_053470 | CNJ00100 | -0.771408136 | 0.01306034 | U3 small nucleolar ribonucleoprotein IMP3 |
| CNXL_053480 | CNJ00110 | -0.061239742 | 0.924825499 | hypothetical protein |
| CNXL_053490 | CNJ00130 | -0.217598228 | 0.615520877 | dimethyladenosine transferase |
| CNXL_053500 | CNJ00140 | -0.083570313 | 0.879531297 | 26S proteasome regulatory subunit N1 |
| CNXL_053510 | CNJ00150 | -0.724606852 | 0.057484307 | fumarate reductase |
| CNXL_053520 | CNJ00160 | 0.311525215 | 0.483451258 | hypothetical protein |
| CNXL_053530 | | -0.093887985 | 0.958064567 | G2/mitotic-specific cyclin 1/2 |
| CNXL_053540 | CNJ00170 | -0.59473438 | 0.094172083 | Unknown |
| CNXL_053550 | CNJ00180 | 0.050583556 | 0.933786064 | hypothetical protein |
| CNXL_053560 | | 0.141493115 | 0.814730279 | nucleoside-diphosphate kinase |
| CNXL_053570 | CNJ00190 | -0.366197957 | 0.516492158 | hypothetical protein |
| CNXL_053580 | CNJ00200 | -0.286648657 | 0.614706438 | hypothetical protein |
| CNXL_053590 | CNJ00210 | -0.09094404 | 0.884028948 | Unknown |
| CNXL_053600 | CNJ00220 | -0.536154828 | 0.197375675 | alpha-1 |
| CNXL_053610 | CNJ00230 | -0.18810528 | 0.762506485 | RNA helicase |
| CNXL_053620 | CNJ00240 | 0.227098026 | 0.602311333 | hypothetical protein |
| CNXL_053630 | CNJ00250 | -0.157471261 | 0.777403833 | hypothetical protein |
| CNXL_053640 | CNJ00260 | -0.011522639 | 0.989601195 | hypothetical protein |
| CNXL_053650 | CNJ00270 | -0.516464966 | 0.438757509 | poly |
| CNXL_053660 | CNJ00280 | -0.461333477 | 0.280743918 | hypothetical protein |
| CNXL_053670 | CNJ00290 | 0.042254407 | 0.957169694 | LIM-homeobox protein |
| CNXL_053680 | CNJ00300 | 0.106702834 | 0.847311574 | hypothetical protein |
| CNXL_053690 | CNJ00320 | 0.45217149 | 0.487397795 | hypothetical protein |
| CNXL_053700 | CNJ00330 | -0.141637442 | 0.823576183 | Unknown |
| CNXL_053710 | CNJ00340 | 0.441316198 | 0.593883843 | hypothetical protein |
| CNXL_053720 | CNJ00350 | 0.156938048 | 0.838312727 | hypothetical protein |
| CNXL_053730 | CNJ00360 | -0.290646368 | 0.534447681 | prolyl endopeptidase |
| CNXL_053740 | CNJ00370 | -0.035525407 | 0.969527869 | hypothetical protein |
| CNXL_053750 | CNJ00390 | -0.220565245 | 0.647485945 | hypothetical protein |
| CNXL_053760 | | -0.816629502 | 0.527852637 | 3-methyl-2-oxobutanoate hydroxymethyltransferase |
| CNXL_053770 | CNJ00400 | -0.469744345 | 0.313451634 | Unknown |
| CNXL_053780 | CNJ00410 | -0.546607879 | 0.25888418 | DNA topoisomerase III |
| CNXL_053790 | | -0.602516666 | 0.408746917 | glycine hydroxymethyltransferase |
| CNXL_053800 | | -1.058604619 | 0.116300052 | hypothetical protein |
| CNXL_053810 | CNJ00430 | -0.263896522 | 0.595850448 | hypothetical protein |
| CNXL_053820 | CNJ00460 | 0.285738244 | 0.504097918 | tryptophan-tRNA ligase |
| CNXL_053830 | CNJ00470 | -0.14441422 | 0.803947851 | C-3 sterol dehydrogenase |
| CNXL_053840 | CNJ00480 | -0.039105982 | 0.96470999 | hypothetical protein |
| CNXL_053850 | CNJ00490 | -0.167824804 | 0.732375511 | signal peptidase I |
| CNXL_053860 | | 0.100601359 | 0.890150525 | argonaute protein |
| CNXL_053870 | CNJ00500 | -0.010913924 | 0.990777629 | hypothetical protein |
| CNXL_053880 | CNJ00510 | -0.24347579 | 0.72158924 | ubiquitin conjugating enzyme |
| CNXL_053890 | CNJ00520 | -0.091888433 | 0.889001755 | hypothetical protein |
| CNXL_053900 | CNJ00530 | 1.001913487 | 0.001836217 | ATP-binding cassette |
| CNXL_053910 | CNJ00540 | -0.559034704 | 0.227624637 | hypothetical protein |
| CNXL_053920 | CNJ00550 | -0.016078586 | 0.988526504 | L-aminoadipate-semialdehyde dehydrogenase |

| | | | | |
|---|---|---|---|---|
| CNXL_053930 | | -0.308163786 | 0.566457057 | hypothetical protein |
| CNXL_053940 | CNJ00560 | -0.199905865 | 0.691713682 | Unknown |
| CNXL_053950 | CNJ00570 | -0.006854531 | 0.993094074 | OPT family small oligopeptide transporter |
| CNXL_053960 | CNJ00580 | -0.30773916 | 0.647127462 | hypothetical protein |
| CNXL_053970 | | 0.055997544 | 0.935785373 | prolyl oligopeptidase |
| CNXL_053980 | CNJ00590 | 0.609313942 | 0.047122187 | hypothetical protein |
| CNXL_053990 | CNJ00600 | -0.383801751 | 0.432050869 | glycogen |
| CNXL_054000 | | 0.379295039 | 0.782518595 | endoplasmic reticulum protein |
| CNXL_054010 | CNJ00610 | -0.775086573 | 0.002579204 | hypothetical protein |
| CNXL_054020 | CNJ00620 | 0.103497157 | 0.847718093 | Unknown |
| CNXL_054030 | CNJ00630 | -0.522100286 | 0.27629814 | cerevisin |
| CNXL_054040 | CNJ00640 | -0.000325489 | 0.999153977 | hypothetical protein |
| CNXL_054050 | CNJ00650 | -0.241327832 | 0.628314018 | COP9 signalosome complex subunit 12 |
| CNXL_054060 | CNJ00660 | -0.540771734 | 0.179305892 | translation initiation factor 6 |
| CNXL_054070 | CNJ00670 | -0.531703368 | 0.095784948 | Mob1 family protein |
| CNXL_054080 | CNJ00680 | 0.142605545 | 0.814220979 | hypothetical protein |
| CNXL_054090 | CNJ00690 | 0.520786095 | 0.078954247 | ribitol kinase |
| CNXL_054100 | CNJ00700 | -0.110452879 | 0.893381182 | uracil permease |
| CNXL_054110 | CNJ00710 | -2.184492868 | 6.92767E-16 | Unknown |
| CNXL_054120 | CNJ00720 | -1.068369811 | 0.000129394 | hypothetical protein |
| CNXL_054130 | CNJ00730 | -2.358840381 | 9.27835E-18 | hypothetical protein |
| CNXL_054140 | | -2.642805281 | 0.000162376 | endopeptidase |
| CNXL_054150 | | -0.148289123 | 0.836716033 | Unknown |
| CNXL_054160 | CNJ00740 | -0.186233938 | 0.701325477 | Unknown |
| CNXL_054170 | CNJ00760 | -0.701689552 | 0.026839543 | metacaspase-1 |
| CNXL_054180 | CNJ00770 | -0.493753748 | 0.173210496 | hypothetical protein |
| CNXL_054190 | CNJ00800 | -0.553221924 | 0.067840292 | hypothetical protein |
| CNXL_054200 | CNJ00810 | -0.127506593 | 0.842339591 | ATP-citrate lyase |
| CNXL_054210 | CNJ00820 | -0.503699313 | 0.30897417 | general transcription factor 3C polypeptide 3 |
| CNXL_054220 | CNJ00830 | -0.19877106 | 0.674428586 | tetraspanin |
| CNXL_054230 | CNJ00840 | -0.215001294 | 0.71878528 | hypothetical protein |
| CNXL_054240 | CNJ00850 | -0.377561496 | 0.618219855 | hypothetical protein |
| CNXL_054250 | CNJ00860 | -0.022255799 | 0.980972278 | glutathione synthetase |
| CNXL_054260 | | -0.202229185 | 0.80500309 | sister chromatid cohesion protein PDS5 |
| CNXL_054270 | | -0.096102742 | 0.928525448 | Unknown |
| CNXL_054280 | CNJ00880 | -0.350011728 | 0.570755994 | Unknown |
| CNXL_054290 | CNJ00890 | 0.724229257 | 0.053107002 | actin-like protein ARP6 |
| CNXL_054300 | CNJ00900 | 0.543783458 | 0.083791338 | hypothetical protein |
| CNXL_054310 | | 0.047309954 | 0.947267247 | enoyl reductase |
| CNXL_054320 | CNJ00920 | -1.060777428 | 0.001481509 | hypothetical protein |
| CNXL_054330 | | -0.325692453 | 0.440656673 | rab family protein |
| CNXL_054340 | CNJ00930 | 1.434682131 | 8.77942E-05 | arginine-tRNA-protein transferase |
| CNXL_054350 | CNJ00940 | -0.913035552 | 0.000915409 | short-chain dehydrogenase |
| CNXL_054360 | CNJ00950 | -1.712017412 | 1.47811E-16 | hypothetical protein |
| CNXL_054370 | | 0.024209385 | 0.976407004 | pyruvate decarboxylase |
| CNXL_054380 | CNJ00970 | -0.96011746 | 0.011211877 | hypothetical protein |
| CNXL_054390 | CNJ00980 | -0.308885844 | 0.517631308 | chromosome transmission fidelity protein |
| CNXL_054400 | CNJ00990 | -0.016670907 | 0.986226596 | large subunit ribosomal protein L37 |
| CNXL_054410 | CNJ01000 | 0.054579308 | 0.936783812 | hypothetical protein |
| CNXL_054420 | CNJ01010 | 0.481890002 | 0.271947747 | 26S protease regulatory subunit 8 |
| CNXL_054430 | CNJ01020 | -0.019013331 | 0.983499434 | hypothetical protein |
| CNXL_054440 | CNJ01030 | -0.179695602 | 0.796855977 | ubiquitin-conjugating enzyme |
| CNXL_054450 | CNJ01040 | -0.340013393 | 0.443600568 | mitochondrial matrix protein import protein |
| CNXL_054460 | CNJ01050 | -0.003128373 | 0.996472611 | hypothetical protein |
| CNXL_054470 | | 0.033914111 | 0.965966884 | hypothetical protein |
| CNXL_054480 | | 0.911435972 | 0.00566394 | unspecified product |
| CNXL_054490 | CNJ01070 | 0.148731902 | 0.814220979 | Unknown |
| CNXL_054500 | | -0.238535623 | 0.755488326 | hypothetical protein |

| | | | | |
|---|---|---|---|---|
| CNXL_054510 | CNJ01080 | 0.464018295 | 0.189297101 | hypothetical protein |
| CNXL_054520 | CNJ01090 | -0.555022803 | 0.143982299 | 6-phosphofructokinase |
| CNXL_054530 | CNJ01100 | -0.302922578 | 0.547814913 | xylitol dehydrogenase |
| CNXL_054540 | CNJ01110 | -0.238141873 | 0.703721839 | protein kinase |
| CNXL_054550 | CNJ01120 | 0.294944007 | 0.527938546 | histone deacetylase complex subunit |
| CNXL_054560 | CNJ01130 | -0.944784652 | 0.002744802 | hypothetical protein |
| CNXL_054570 | CNJ01140 | -0.104876103 | 0.864719008 | hypothetical protein |
| CNXL_054580 | | 0.072574483 | 0.916894829 | hypothetical protein |
| CNXL_054590 | CNJ01150 | -0.253024077 | 0.628625129 | DNA replication complex GINS protein PSF3 |
| CNXL_054600 | CNJ01160 | -0.639095763 | 0.120571776 | putative GTPase-activating protein |
| CNXL_054610 | CNJ01170 | -0.383101096 | 0.666493831 | hypothetical protein |
| CNXL_054620 | CNJ01180 | -0.264780673 | 0.538509171 | hypothetical protein |
| CNXL_054630 | CNJ01190 | -0.037209928 | 0.962194201 | stearoyl-CoA desaturase |
| CNXL_054640 | | -0.820829397 | 0.035781436 | acyl-CoA dehydrogenase |
| CNXL_054650 | CNJ01210 | 0.005541766 | 0.995735232 | Unknown |
| CNXL_054660 | CNJ01220 | -2.983241418 | 1.85342E-41 | acylpyruvate hydrolase |
| CNXL_054670 | CNJ01230 | -0.196427619 | 0.770342269 | hypothetical protein |
| CNXL_054680 | CNJ01240 | -0.305167192 | 0.536746923 | thymidylate synthase |
| CNXL_054690 | CNJ01250 | -0.233698929 | 0.633741739 | target of rapamycin complex 2 subunit |
| CNXL_054700 | | -0.208699878 | 0.819853333 | periodic tryptophan protein 1 |
| CNXL_054710 | CNJ01260 | -0.211982513 | 0.717347987 | hypothetical protein |
| CNXL_054720 | CNJ01270 | -0.227825588 | 0.742187954 | DNA clamp loader |
| CNXL_054730 | CNJ01280 | 0.070271686 | 0.912929878 | zinc finger protein |
| CNXL_054740 | CNJ01290 | -0.484331912 | 0.311850588 | cytoplasmic protein |
| CNXL_054750 | CNJ01300 | -0.310484692 | 0.664771357 | microfibrillar-associated protein 1 |
| CNXL_054760 | | 0.389062835 | 0.417866535 | hypothetical protein |
| CNXL_054770 | CNJ01330 | -0.381477527 | 0.550258326 | hypothetical protein |
| CNXL_054780 | CNJ01340 | 0.203081054 | 0.677367265 | hypothetical protein |
| CNXL_054790 | | 2.103888825 | 1.14517E-05 | DNA clamp loader |
| CNXL_054800 | CNJ01360 | 0.519826887 | 0.068778813 | MFS transporter |
| CNXL_054810 | CNJ01370 | -0.371505825 | 0.417866535 | MFS transporter |
| CNXL_054820 | CNJ01380 | -0.395472218 | 0.278597085 | hypothetical protein |
| CNXL_054830 | | -0.752393797 | 0.28126755 | midasin |
| CNXL_054840 | CNJ01390 | -2.713007407 | 2.8333E-39 | Unknown |
| CNXL_054850 | CNJ01400 | -0.270587171 | 0.676573672 | hypothetical protein |
| CNXL_054860 | CNJ01410 | -0.027626257 | 0.970458444 | hypothetical protein |
| CNXL_054870 | CNJ01420 | 0.143570462 | 0.779355929 | hypothetical protein |
| CNXL_054880 | | -0.338248937 | 0.873324574 | hypothetical protein |
| CNXL_054890 | CNJ01430 | -0.41937415 | 0.337036418 | hypothetical protein |
| CNXL_054900 | CNJ01440 | 0.027645558 | 0.970404228 | solute carrier family 25 |
| CNXL_054910 | CNJ01450 | 0.296271684 | 0.451211894 | citrate lyase subunit beta-like protein |
| CNXL_054920 | CNJ01460 | -0.252573552 | 0.58721075 | MFS multidrug transporter protein |
| CNXL_054930 | CNJ01470 | 0.125963758 | 0.819555276 | oligosaccharyltransferase complex subunit alpha |
| CNXL_054940 | CNJ01480 | 0.401191604 | 0.375237211 | KH domain-containing protein |
| CNXL_054950 | CNJ01490 | 0.412730944 | 0.45163285 | hypothetical protein |
| CNXL_054960 | CNJ01500 | 0.113011565 | 0.86171371 | hypothetical protein |
| CNXL_054970 | CNJ01510 | -0.074731273 | 0.902229878 | solute carrier family 41 |
| CNXL_054980 | CNJ01520 | -0.089543753 | 0.902229878 | GTPase activating protein |
| CNXL_054990 | CNJ01530 | -0.231058227 | 0.753305317 | hypothetical protein |
| CNXL_055000 | CNJ01540 | 0.082805477 | 0.889191243 | hypothetical protein |
| CNXL_055010 | | 0.434567362 | 0.392734784 | ubiquitin-protein ligase E3 C |
| CNXL_055020 | CNJ01550 | -0.401259142 | 0.369739382 | Unknown |
| CNXL_055030 | CNJ01560 | -0.114665011 | 0.821753367 | hypothetical protein |
| CNXL_055040 | | 0.541553711 | 0.10930783 | large subunit ribosomal protein L18Ae |
| CNXL_055050 | CNJ01580 | 0.04612945 | 0.950999357 | Unknown |
| CNXL_055060 | CNJ01590 | -0.33979782 | 0.495174856 | DNA-directed RNA polymerase III subunit RPC8 |
| CNXL_055070 | CNJ01600 | -0.51398013 | 0.106221376 | ATP-dependent RNA helicase DBP7 |
| CNXL_055080 | CNJ01610 | 0.506834682 | 0.233174199 | G-protein coupled receptor |

| | | | | |
|---|---|---|---|---|
| CNXL_055090 | | 0.131668823 | 0.812703117 | hypothetical protein |
| CNXL_055100 | CNJ01630 | -0.223290853 | 0.637927859 | hypothetical protein |
| CNXL_055110 | CNJ01640 | -0.23689383 | 0.750135528 | DNA repair protein RAD5 |
| CNXL_055120 | CNJ01650 | -0.673283594 | 0.040033951 | mitochondrial ornithine carrier protein |
| CNXL_055130 | CNJ01660 | -1.213705407 | 0.001160116 | metalloproteinase |
| CNXL_055140 | CNJ01670 | 0.180509502 | 0.764330585 | hypothetical protein |
| CNXL_055150 | CNJ01680 | 0.772641411 | 0.010405914 | hypothetical protein |
| CNXL_055160 | CNJ01690 | -0.049567795 | 0.95265881 | fimbrin |
| CNXL_055170 | CNJ01700 | 0.227307002 | 0.635049881 | cytochrome c oxidase assembly protein COX19 |
| CNXL_055180 | CNJ01720 | -0.026693994 | 0.970906596 | hypothetical protein |
| CNXL_055190 | CNJ01730 | -0.236459587 | 0.764293155 | endosome protein |
| CNXL_055200 | CNJ01740 | 0.183290775 | 0.743437511 | DNA primase large subunit |
| CNXL_055210 | CNJ01750 | 1.609528993 | 1.16109E-06 | oligosaccharyltransferase complex subunit beta |
| CNXL_055220 | CNJ01760 | 0.325643103 | 0.496574337 | mannose-6-phosphate isomerase |
| CNXL_055230 | CNJ01770 | 0.940866063 | 0.000367053 | hypothetical protein |
| CNXL_055240 | | 0.148709105 | 0.777403833 | hypothetical protein |
| CNXL_055250 | CNJ01780 | -0.023259756 | 0.981240834 | hypothetical protein |
| CNXL_055260 | CNJ01790 | 0.030339997 | 0.965966884 | protein OS-9 |
| CNXL_055270 | CNJ01800 | 0.819434912 | 0.00853461 | hypothetical protein |
| CNXL_055280 | | 0.991011693 | 0.004267486 | hypothetical protein |
| CNXL_055290 | CNJ01820 | 0.500122471 | 0.210946096 | Unknown |
| CNXL_055300 | CNJ01830 | -0.744320682 | 0.031099326 | hypothetical protein |
| CNXL_055310 | CNJ01840 | 0.636020883 | 0.032932177 | hypothetical protein |
| CNXL_055320 | CNJ01850 | -0.160420685 | 0.782518595 | gluconolactonase |
| CNXL_055330 | CNJ01860 | -1.213965166 | 0.000239981 | hypothetical protein |
| CNXL_055340 | CNJ01870 | -0.91989459 | 0.000131021 | hypothetical protein |
| CNXL_055350 | CNJ01880 | 0.512881288 | 0.107354436 | mitochondrial protein |
| CNXL_055360 | CNJ01890 | -1.027298135 | 0.001004751 | ammonium transporter |
| CNXL_055370 | | -0.721883389 | 0.051496226 | hypothetical protein |
| CNXL_055380 | CNJ01900 | 0.006950523 | 0.992176872 | Unknown |
| CNXL_055390 | CNJ01910 | -0.733739695 | 0.08912928 | cytoplasmic protein |
| CNXL_055400 | CNJ01920 | -0.464951712 | 0.30555532 | G-protein signaling GTPase |
| CNXL_055410 | CNJ01930 | 0.017398467 | 0.985803294 | large subunit ribosomal protein L4e |
| CNXL_055420 | CNJ01940 | 0.059917467 | 0.938435008 | hypothetical protein |
| CNXL_055430 | CNJ01950 | 0.340654287 | 0.43911626 | dihydrofolate reductase |
| CNXL_055440 | | 1.917740062 | 0.116975288 | arp2/3 complex 21 kda subunit |
| CNXL_055450 | CNJ01960 | 0.222191271 | 0.689555844 | hypothetical protein |
| CNXL_055460 | | -0.327405939 | 0.670362199 | hypothetical protein |
| CNXL_055470 | | -0.289918434 | 0.611569335 | Unknown |
| CNXL_055480 | CNJ01980 | 0.065269757 | 0.929814702 | hypothetical protein |
| CNXL_055490 | CNJ01990 | 0.012423319 | 0.988641003 | hypothetical protein |
| CNXL_055500 | CNJ02000 | 0.371484595 | 0.37530434 | nucleolar essential protein 1 |
| CNXL_055510 | CNJ02010 | -0.163816833 | 0.782594586 | rab family protein |
| CNXL_055520 | CNJ02020 | 0.636320787 | 0.134763285 | hypothetical protein |
| CNXL_055530 | CNJ02030 | -0.662877533 | 0.059237283 | hypothetical protein |
| CNXL_055540 | | 0.20077763 | 0.737030484 | nuclear protein |
| CNXL_055550 | CNJ02040 | 0.08379188 | 0.912929878 | hypothetical protein |
| CNXL_055560 | CNJ02050 | 0.241722195 | 0.640550491 | homoserine dehydrogenase |
| CNXL_055570 | CNJ02060 | 0.057806643 | 0.927441884 | hypothetical protein |
| CNXL_055580 | | -0.864493463 | 0.648554518 | isocitrate dehydrogenase |
| CNXL_055590 | | -0.110510761 | 0.893662159 | Unknown |
| CNXL_055600 | CNJ02080 | 0.311709089 | 0.495138797 | Unknown |
| CNXL_055610 | | 0.288068482 | 0.709726462 | hypothetical protein |
| CNXL_055620 | CNJ02090 | 0.323046705 | 0.391858761 | hypothetical protein |
| CNXL_055630 | CNJ02100 | -0.386961326 | 0.265153342 | hypothetical protein |
| CNXL_055640 | CNJ02110 | -0.743227441 | 0.028167535 | monosaccharide transporter |
| CNXL_055650 | CNJ02120 | 0.471160829 | 0.168782041 | monosaccharide transporter |
| CNXL_055660 | CNJ02130 | -0.04046341 | 0.956990161 | hypothetical protein |

| | | | | |
|---|---|---|---|---|
| CNXL_055670 | CNJ02140 | -0.131525202 | 0.837479784 | hypothetical protein |
| CNXL_055680 | CNJ02150 | -0.894296617 | 0.010233234 | membrane protein |
| CNXL_055690 | CNJ02160 | 0.803311335 | 0.014003718 | hypothetical protein |
| CNXL_055700 | CNJ02170 | 0.125675989 | 0.847718093 | hypothetical protein |
| CNXL_055710 | CNJ02180 | -0.368688546 | 0.432217251 | hypothetical protein |
| CNXL_055720 | CNJ02200 | -0.38244299 | 0.434581417 | hypothetical protein |
| CNXL_055730 | CNJ02210 | 0.416265288 | 0.422414274 | phosphatidylserine decarboxylase |
| CNXL_055740 | CNJ02220 | 0.247163272 | 0.595655121 | spermine transporter |
| CNXL_055750 | CNJ02230 | -0.136228982 | 0.813601278 | adenine nucleotide transporter |
| CNXL_055760 | CNJ02240 | 0.177735479 | 0.770559628 | calcineurin a catalytic subunit |
| CNXL_055770 | CNJ02250 | 0.223544555 | 0.626371103 | signal recognition particle subunit SRP19 |
| CNXL_055780 | CNJ02260 | -0.132481761 | 0.81790448 | regulatory protein cys-3 |
| CNXL_055790 | CNJ02270 | -0.124147589 | 0.815340666 | large subunit ribosomal protein L14e |
| CNXL_055800 | CNJ02280 | -0.610672581 | 0.109223771 | phenylalanine-tRNA ligase |
| CNXL_055810 | CNJ02290 | -0.094174757 | 0.889001755 | hypothetical protein |
| CNXL_055820 | CNJ02300 | -0.001783364 | 0.996833113 | ribosomal RNA-processing protein 9 |
| CNXL_055830 | CNJ02310 | -0.091714023 | 0.899804184 | protein transporter SEC31 |
| CNXL_055840 | CNJ02320 | 0.058009572 | 0.927387051 | sterol regulatory element-binding protein |
| CNXL_055850 | CNJ02330 | 0.011482019 | 0.988662258 | peptidyl-prolyl isomerase CWC27 |
| CNXL_055860 | | -0.69892285 | 0.007904957 | golgi phosphoprotein 3 |
| CNXL_055870 | CNJ02340 | -0.006868717 | 0.992176872 | hypothetical protein |
| CNXL_055880 | CNJ02350 | 0.292545229 | 0.492433582 | XPG N-terminal domain-containing protein |
| CNXL_055890 | CNJ02360 | 0.018304936 | 0.983493091 | COP9 signalosome complex subunit 5 |
| CNXL_055900 | | 0.602988785 | 0.453650498 | hypothetical protein |
| CNXL_055910 | CNJ02370 | -0.29441457 | 0.569605611 | hypothetical protein |
| CNXL_055920 | CNJ02380 | -0.435426689 | 0.201056667 | ran-binding protein 3 |
| CNXL_055930 | CNJ02390 | -0.420536248 | 0.616642335 | V-type ATPase |
| CNXL_055940 | CNJ02400 | -0.583999531 | 0.108666212 | hypothetical protein |
| CNXL_055950 | CNJ02410 | -0.096604367 | 0.884028948 | formin |
| CNXL_055960 | CNJ02430 | -0.017888829 | 0.983499434 | hypothetical protein |
| CNXL_055970 | | -0.275818443 | 0.757283749 | hypothetical protein |
| CNXL_055980 | CNJ02440 | 0.052174084 | 0.944879244 | hypothetical protein |
| CNXL_055990 | CNJ02450 | 0.523508643 | 0.262925589 | hypothetical protein |
| CNXL_056000 | CNJ02460 | -0.084158164 | 0.912929878 | Unknown |
| CNXL_056010 | CNJ02470 | -0.30548235 | 0.632998683 | Unknown |
| CNXL_056020 | CNJ02480 | -0.336742583 | 0.577988443 | PAB-dependent poly |
| CNXL_056030 | | -0.295549402 | 0.78519633 | deoxyhypusine synthase |
| CNXL_056040 | CNJ02490 | -0.010783585 | 0.991672229 | Unknown |
| CNXL_056050 | CNJ02500 | 0.219386504 | 0.717347987 | peptidyl-prolyl cis-trans isomerase H |
| CNXL_056060 | CNJ02510 | 0.345512096 | 0.498081161 | mitotic spindle assembly checkpoint protein MAD1 |
| CNXL_056070 | CNJ02520 | 0.048802769 | 0.956622745 | hypothetical protein |
| CNXL_056080 | CNJ02530 | 0.101780144 | 0.889001755 | homocysteine S-methyltransferase |
| CNXL_056090 | CNJ02540 | 0.211416172 | 0.6409676 | hypothetical protein |
| CNXL_056100 | | 0.381660471 | 0.398860086 | histone H3 |
| CNXL_056110 | CNJ02550 | -0.128731629 | 0.840830694 | hypothetical protein |
| CNXL_056120 | | 0.301586785 | 0.619100215 | large subunit ribosomal protein L33 |
| CNXL_056130 | CNJ02560 | -0.589264655 | 0.203714539 | hypothetical protein |
| CNXL_056140 | CNJ02580 | 0.927178293 | 0.001141719 | RalA-binding protein 1 |
| CNXL_056150 | CNJ02590 | 0.391732552 | 0.290745908 | hypothetical protein |
| CNXL_056160 | CNJ02600 | -0.691862394 | 0.45163285 | MFS transporter |
| CNXL_056170 | CNJ02610 | -0.119115675 | 0.843098307 | dihydrodipicolinate synthase |
| CNXL_056180 | CNJ02620 | 0.702749698 | 0.012859811 | nuclear protein |
| CNXL_056190 | CNJ02630 | -0.327510533 | 0.519073971 | bHLH family transcription factor |
| CNXL_056200 | CNJ02640 | 0.105543674 | 0.869142839 | hypothetical protein |
| CNXL_056210 | CNJ02650 | -0.052103746 | 0.936086163 | exosome complex component MTR3 |
| CNXL_056220 | CNJ02660 | -0.345985878 | 0.451182928 | small nuclear ribonucleoprotein B and B' |
| CNXL_056230 | CNJ02670 | -0.051109788 | 0.948286148 | transcriptional regulatory protein |
| CNXL_056240 | CNJ02680 | -0.444364041 | 0.388091044 | hypothetical protein |

| | | | | |
|---|---|---|---|---|
| CNXL_056250 | CNJ02690 | 0.109058643 | 0.833396996 | Atypical/ABC1/ABC1-B protein kinase |
| CNXL_056260 | CNJ02700 | 0.414785801 | 0.281208534 | mitochondrial import receptor subunit tom22 |
| CNXL_056270 | CNJ02710 | -0.28370771 | 0.595654142 | mannose-P-dolichol utilization defect 1 |
| CNXL_056280 | | -0.035006499 | 0.976006716 | membrane protein |
| CNXL_056290 | CNJ02720 | -0.98847638 | 0.069009964 | Unknown |
| CNXL_056300 | CNJ02730 | -0.018038922 | 0.984913368 | hypothetical protein |
| CNXL_056310 | CNJ02740 | 1.19186915 | 0.000303644 | mRNA binding protein |
| CNXL_056320 | | 0.204818302 | 0.78519633 | vacuolar protein |
| CNXL_056330 | CNJ02750 | 0.25053416 | 0.602461418 | hypothetical protein |
| CNXL_056340 | CNJ02760 | 0.458066101 | 0.254273219 | transitional endoplasmic reticulum ATPase |
| CNXL_056350 | | 0.437271586 | 0.276076329 | 7 |
| CNXL_056360 | | -0.271757026 | 0.512930138 | derlin-2/3 |
| CNXL_056370 | 910\|CNA08330\|CN | -1.560720284 | NA | Unknown |
| CNXL_056380 | CNK00500 | 3.527491554 | NA | Unknown |
| CNXL_056390 | CNN02020 | 1.619795275 | NA | Unknown |
| CNXL_056400 | CNJ02790 | 0 | NA | Unknown |
| CNXL_056410 | | NA | NA | Unknown |
| CNXL_056420 | CNJ02810 | 0 | NA | Unknown |
| CNXL_056430 | | -1.693240689 | 0.660204343 | Unknown |
| CNXL_056440 | CNF03140 | 0 | NA | Unknown |
| CNXL_056450 | | -1.440226686 | NA | Unknown |
| CNXL_056460 | | 0.049270853 | 0.992176872 | Unknown |
| CNXL_056470 | | 0.973950494 | 0.023443562 | hypothetical protein |
| CNXL_056480 | | 1.067533227 | 0.145012655 | Unknown |
| CNXL_056490 | CNJ02870 | -2.244602033 | 1.67779E-17 | Unknown |
| CNXL_056500 | | -0.015816636 | 0.985173026 | hypothetical protein |
| CNXL_056510 | CNJ02880 | 0.125349323 | 0.825693641 | hypothetical protein |
| CNXL_056520 | CNJ02890 | -0.332539104 | 0.531504526 | rho family protein |
| CNXL_056530 | CNJ02900 | -2.378459944 | 1.22874E-18 | xylosylphosphotransferase |
| CNXL_056540 | CNJ02910 | -0.484060288 | 0.192778785 | transglycosylase SLT domain-containing protein |
| CNXL_056550 | | 0.112195749 | 0.879688103 | glutamate synthase |
| CNXL_056560 | CNJ02920 | -0.547127061 | 0.083435528 | escrt-II complex subunit |
| CNXL_056570 | | -0.643193988 | 0.628160086 | iron-responsive gata-type transcription factor |
| CNXL_056580 | CNJ02940 | -0.073009856 | 0.918156352 | Unknown |
| CNXL_056590 | CNJ02950 | -2.352576718 | 4.43425E-15 | NAD-dependent histone deacetylase SIR2 |
| CNXL_056600 | CNJ02960 | -0.032741193 | 0.974108291 | vacuolar protein |
| CNXL_056610 | CNJ02970 | -0.334115376 | 0.404388182 | cytoplasmic protein |
| CNXL_056620 | CNJ02980 | -0.789182058 | 0.255384529 | para-nitrobenzyl esterase |
| CNXL_056630 | | -0.108389486 | 0.891760995 | hypothetical protein |
| CNXL_056640 | CNJ02990 | -0.474616109 | 0.279637638 | hypothetical protein |
| CNXL_056650 | CNJ03000 | -1.504056224 | 9.49523E-10 | mitochondrial protein |
| CNXL_056660 | CNJ03020 | -0.480946947 | 0.374056899 | hypothetical protein |
| CNXL_056670 | CNJ03030 | -0.424408347 | 0.417823237 | hypothetical protein |
| CNXL_056680 | CNJ03040 | -0.88894061 | 0.003784105 | hypothetical protein |
| CNXL_056690 | CNJ03070 | 1.162050952 | 0.700697818 | endo-1 |
| CNXL_056700 | | 0.249113063 | 0.776535714 | hypothetical protein |
| CNXL_056710 | CNJ03080 | 0.285672219 | 0.618908161 | Unknown |
| CNXL_056720 | CNJ03090 | 0.245721345 | 0.648446577 | hypothetical protein |
| CNXL_056730 | CNJ03100 | 0.071738273 | 0.916084262 | glycogen debranching enzyme |
| CNXL_056740 | CNJ03110 | -0.195800168 | 0.656378443 | hypothetical protein |
| CNXL_056750 | | -0.2401884 | 0.607569451 | small subunit ribosomal protein S18 |
| CNXL_056760 | | -0.529311204 | NA | large subunit ribosomal protein L44 |
| CNXL_056770 | CNJ03130 | 1.093915029 | 0.001830484 | Unknown |
| CNXL_056780 | CNJ03140 | 0.606637142 | 0.107144034 | hypothetical protein |
| CNXL_056790 | CNJ03150 | 0.502254564 | 0.741662589 | hypothetical protein |
| CNXL_056800 | | 0.653157785 | 0.079525425 | Unknown |
| CNXL_056810 | | -0.005220237 | 0.995428247 | hypothetical protein |
| CNXL_056820 | CNJ03160 | -1.427817701 | 1.16109E-06 | hypothetical protein |

| | | | | |
|---|---|---|---|---|
| CNXL_056830 | CNJ03170 | -0.060163492 | 0.932840267 | hypothetical protein |
| CNXL_056840 | | 0.09966054 | 0.893873062 | hypothetical protein |
| CNXL_056850 | | -3.235976769 | 9.36837E-06 | hypothetical protein |
| CNXL_056860 | | 0.326606014 | 0.399376868 | hypothetical protein |
| CNXL_056870 | CNJ03180 | 0.197843721 | 0.671905355 | hypothetical protein |
| CNXL_056880 | | 0.574279537 | 0.374737238 | hypothetical protein |
| CNXL_056890 | CNJ03190 | 0.197479413 | 0.679200314 | hypothetical protein |
| CNXL_056900 | CNJ03200 | -0.112282848 | 0.864719008 | nuclear protein |
| CNXL_056910 | CNJ03210 | 0.078336974 | 0.919952257 | tyrosine-tRNA ligase |
| CNXL_056920 | CNJ03220 | -0.179127758 | 0.771763303 | hypothetical protein |
| CNXL_056930 | CNJ03230 | -0.154767057 | 0.785592376 | MFS transporter |
| CNXL_056940 | CNJ03240 | -0.238278294 | 0.699223686 | cytoplasmic protein |
| CNXL_056950 | | 1.237214464 | 0.456910562 | WD40 repeat protein Ciao1 |
| CNXL_056960 | CNJ03250 | 0.041131313 | 0.959294084 | hypothetical protein |
| CNXL_056970 | CNJ03260 | 2.26746438 | 0.017980001 | SEL1 protein |
| CNXL_056980 | CNJ03270 | 0.055509305 | 0.931449101 | hypothetical protein |
| CNXL_056990 | | -2.405212302 | 0.395304481 | clathrin heavy chain |
| CNXL_057000 | CNJ03280 | -0.121338213 | 0.846112002 | Unknown |
| CNXL_057010 | CNJ03290 | -0.015383224 | 0.987076518 | tRNA |
| CNXL_057020 | CNJ03300 | -0.101740163 | 0.95265881 | 26S protease regulatory subunit 10B |
| CNXL_057030 | CNJ03310 | -0.076554528 | 0.936086163 | hypothetical protein |
| CNXL_057040 | CNJ03320 | 0.025274056 | 0.981567052 | hypothetical protein |
| CNXL_057050 | CNJ03330 | 0.125422698 | 0.889001755 | serine carboxypeptidase-like 33 |
| CNXL_057060 | CNJ03340 | 0.894268133 | 0.001217161 | Unknown |
| CNXL_057070 | CNJ03350 | 0.098262759 | 0.879531297 | hypothetical protein |
| CNXL_057080 | CNJ03360 | 0.287732801 | 0.441020034 | syntaxin 5 |
| CNXL_057090 | | 0.351073588 | 0.499798006 | small nuclear ribonucleoprotein G |
| CNXL_057100 | CNJ03370 | -0.010288766 | 0.989214735 | hypothetical protein |
| CNXL_057110 | CNJ03380 | 0.564565661 | 0.10010968 | large subunit ribosomal protein L11 |
| CNXL_057120 | CNJ03390 | -1.862552079 | 0.031003853 | cytoplasmic protein |
| CNXL_057130 | CNJ03400 | 1.146949495 | 0.00053105 | hypothetical protein |
| CNXL_057140 | | 0.484066268 | 0.463658548 | galactose transporter |
| CNXL_057150 | | 0.64903143 | 0.175966187 | Unknown |
| CNXL_057160 | | -0.053913191 | 0.952560219 | Unknown |
| CNXL_057170 | CNJ03440 | -0.730868298 | 0.115488803 | Unknown |
| CNXL_057180 | CNK00010 | -0.00297827 | 0.996472611 | Unknown |
| CNXL_057190 | CNK00020 | -0.05756438 | 0.935535525 | hypothetical protein |
| CNXL_057200 | CNK00030 | 0.441947025 | 0.237871488 | peptidyl-prolyl isomerase G |
| CNXL_057210 | CNK00040 | 0.183168816 | 0.746765994 | hypothetical protein |
| CNXL_057220 | CNK00050 | 0.396062998 | 0.485738771 | arsenical-resistance protein |
| CNXL_057230 | CNK00060 | 0.817055413 | 0.001575332 | Unknown |
| CNXL_057240 | CNK00070 | -0.25201842 | 0.58721075 | beta-fructofuranosidase |
| CNXL_057250 | CNK00080 | 0.270775119 | 0.553544499 | hypothetical protein |
| CNXL_057260 | CNK00090 | -0.152524521 | 0.827553884 | anon-23da protein |
| CNXL_057270 | CNK00100 | -0.568253713 | 0.140437813 | hypothetical protein |
| CNXL_057280 | CNK00110 | -0.272144744 | 0.536866826 | ubiquitin carboxyl-terminal hydrolase 10 |
| CNXL_057290 | CNK00120 | -0.633755128 | 0.21296764 | rRNA 2'-O-methyltransferase fibrillarin |
| CNXL_057300 | CNK00130 | 0.59719077 | 0.032067141 | hypothetical protein |
| CNXL_057310 | CNK00140 | -0.516138623 | 0.246141967 | thiol-specific antioxidant protein 3 |
| CNXL_057320 | | 1.364365468 | 0.014540957 | hypothetical protein |
| CNXL_057330 | | 0.683310906 | 0.103401734 | hypothetical protein |
| CNXL_057340 | | -2.1438589 | 0.009650246 | Unknown |
| CNXL_057350 | CNK00160 | -2.294289544 | 1.62079E-07 | Unknown |
| CNXL_057360 | | -2.263004193 | 0.00030279 | Unknown |
| CNXL_057370 | | -0.641817479 | 0.612409488 | Unknown |
| CNXL_057380 | CNK00170 | -0.43844707 | 0.274753763 | Unknown |
| CNXL_057390 | CNK00180 | 0.403462514 | 0.21653299 | gamma-tubulin |
| CNXL_057400 | CNK00190 | 0.540747513 | 0.062066354 | L-serine ammonia-lyase |

| | | | | |
|---|---|---|---|---|
| CNXL_057410 | CNK00200 | 0.124139536 | 0.810224824 | hypothetical protein |
| CNXL_057420 | | 0.236369056 | 0.693186442 | pyridoxal biosynthesis lyase pdxS |
| CNXL_057430 | CNK00210 | -0.253207532 | 0.650714942 | hypothetical protein |
| CNXL_057440 | CNK00220 | 0.790501336 | 0.008086001 | translation initiation factor 4B |
| CNXL_057450 | CNK00230 | 1.01478404 | 0.002196564 | cytoplasmic protein |
| CNXL_057460 | CNK00240 | 0.078747478 | 0.916894829 | hypothetical protein |
| CNXL_057470 | CNK00250 | -0.131872564 | 0.846990996 | mRNA-decapping enzyme subunit 2 |
| CNXL_057480 | CNK00260 | -0.044067808 | 0.976006716 | hypothetical protein |
| CNXL_057490 | CNK00270 | -0.008879227 | 0.992176872 | Unknown |
| CNXL_057500 | CNK00280 | 0.241684559 | 0.586181879 | UDP-N-acetyl-glucosamine-1-p transferase |
| CNXL_057510 | CNK00290 | 0.157589618 | 0.78983385 | 2 |
| CNXL_057520 | CNK00300 | -0.101167971 | 0.857811508 | 26S proteasome regulatory subunit N7 |
| CNXL_057530 | CNK00320 | -0.298386828 | 0.433043689 | chitin synthase export chaperone |
| CNXL_057540 | CNK00330 | -0.797033974 | 0.042733719 | hypothetical protein |
| CNXL_057550 | CNK00350 | 0.036799311 | 0.963072147 | cytoplasmic protein |
| CNXL_057560 | CNK00340 | 0.161035194 | 0.813614656 | hypothetical protein |
| CNXL_057570 | CNK00360 | -0.167259456 | 0.779355929 | hypothetical protein |
| CNXL_057580 | CNK00370 | 0.341814927 | 0.365784664 | preconditioning-inducible protein |
| CNXL_057590 | CNK00380 | -0.367700644 | 0.34086224 | hypothetical protein |
| CNXL_057600 | CNK00390 | 0.319734651 | 0.39417374 | membrane transporter |
| CNXL_057610 | CNK00400 | 0.00241328 | 0.996739329 | sodium-hydrogen antiporter |
| CNXL_057620 | CNK00410 | 0.171136419 | 0.779892494 | cytoplasmic protein |
| CNXL_057630 | CNK00420 | -0.334650251 | 0.504222021 | hypothetical protein |
| CNXL_057640 | CNK00430 | 0.167205667 | 0.755590901 | CAMK protein kinase |
| CNXL_057650 | CNK00440 | 1.213619352 | 0.000252585 | cytoplasmic protein |
| CNXL_057660 | | 0.75211591 | 0.005588051 | hypothetical protein |
| CNXL_057670 | CNK00450 | 0.006274979 | 0.993955674 | Unknown |
| CNXL_057680 | CNK00460 | 0.646455307 | 0.062444654 | hypothetical protein |
| CNXL_057690 | CNK00470 | 0.760743741 | 0.012315571 | SBDS family rRNA metabolism protein |
| CNXL_057700 | | 0.560472084 | 0.067840292 | mitochondrial protein |
| CNXL_057710 | | 0.607940384 | 0.261955879 | hypothetical protein |
| CNXL_057720 | | 0.347942046 | 0.334301792 | Unknown |
| CNXL_057730 | | 0.156272184 | NA | hypothetical protein |
| CNXL_057740 | CNK00540 | 1.325339401 | 0.363186613 | Unknown |
| CNXL_057750 | CNK00550 | -0.091713019 | 0.884028948 | spermine transporter |
| CNXL_057760 | | -0.285014353 | 0.608495206 | Unknown |
| CNXL_057770 | CNK00560 | 0.218151167 | 0.713103489 | acyl-CoA dehydrogenase |
| CNXL_057780 | CNK00570 | -0.337826193 | 0.555395578 | DNA polymerase delta subunit 1 |
| CNXL_057790 | | 0.097372808 | 0.919196787 | tRNA-splicing endonuclease subunit Sen54 |
| CNXL_057800 | | -0.349109242 | 0.681547537 | Unknown |
| CNXL_057810 | CNK00580 | -0.823090484 | 0.037448032 | Unknown |
| CNXL_057820 | CNK00590 | -0.297534333 | 0.575791977 | homoaconitase |
| CNXL_057830 | CNK00600 | 0.29729217 | 0.518915642 | hepatocyte nuclear factor |
| CNXL_057840 | CNK00610 | 0.208869063 | 0.691220257 | UBA/TS-N domain-containing protein |
| CNXL_057850 | | -0.09873809 | 0.897988379 | hypothetical protein |
| CNXL_057860 | CNK00640 | -0.441498504 | 0.488583556 | Unknown |
| CNXL_057870 | CNK00650 | 0.870138902 | 0.01116274 | cofactor D |
| CNXL_057880 | CNK00660 | 0.271557318 | 0.527852637 | Unknown |
| CNXL_057890 | CNK00670 | -0.218220846 | 0.623094586 | RAB small monomeric GTPase |
| CNXL_057900 | CNK00690 | -0.812397935 | 0.105686013 | hypothetical protein |
| CNXL_057910 | CNK00710 | -0.271756433 | 0.568734545 | thyroid hormone receptor interactor 13 |
| CNXL_057920 | CNK00720 | -0.060033387 | 0.934642219 | hypothetical protein |
| CNXL_057930 | CNK00730 | -0.480914434 | 0.422414274 | hypothetical protein |
| CNXL_057940 | CNK00740 | 0.046570561 | 0.954286695 | capsular associated protein |
| CNXL_057950 | CNK00750 | 0.586247578 | 0.127361287 | tRNA |
| CNXL_057960 | CNK00760 | 0.173387703 | 0.734024208 | serine/threonine-protein phosphatase 2A activator 1 |
| CNXL_057970 | CNK00770 | 0.009805262 | 0.99124946 | hypothetical protein |
| CNXL_057980 | CNK00780 | -0.015036272 | 0.986620478 | hypothetical protein |

| | | | | |
|---|---|---|---|---|
| CNXL_057990 | | 2.223145555 | 4.28036E-12 | 3-hydroxyisobutyrate dehydrogenase |
| CNXL_058000 | CNK00790 | 0.341087099 | 0.567249149 | hypothetical protein |
| CNXL_058010 | CNK00800 | 0.124101736 | 0.847923235 | thioredoxin reductase GliT |
| CNXL_058020 | CNK00810 | -0.477156232 | 0.212829513 | hypothetical protein |
| CNXL_058030 | CNK00820 | -0.071073224 | 0.924607253 | hypothetical protein |
| CNXL_058040 | CNK00830 | -0.131503701 | 0.811240933 | hypothetical protein |
| CNXL_058050 | CNK00840 | -0.107292302 | 0.87678601 | hypothetical protein |
| CNXL_058060 | CNK00850 | 0.788862609 | 0.023885378 | endochitinase |
| CNXL_058070 | CNK00860 | 1.176282298 | 1.07152E-05 | 2 |
| CNXL_058080 | CNK00870 | 0.819309273 | 0.004431117 | tartrate transporter |
| CNXL_058090 | CNK00880 | 0.528164977 | 0.15654435 | hypothetical protein |
| CNXL_058100 | | 0.532867976 | 0.329557168 | flavonol synthase |
| CNXL_058110 | CNK00900 | 0.236965988 | 0.634343476 | Unknown |
| CNXL_058120 | CNK00910 | 0.340677592 | 0.463658548 | hypothetical protein |
| CNXL_058130 | CNK00920 | 0.2938463 | 0.591247363 | tbc1 domain family protein |
| CNXL_058140 | CNK00930 | 0.606142006 | 0.051415986 | hypothetical protein |
| CNXL_058150 | CNK00940 | -0.269250191 | 0.592750411 | DNA ligase 4 |
| CNXL_058160 | CNK00950 | -0.273769335 | 0.500364715 | 3-demethylubiquinone-9 3-O-methyltransferase |
| CNXL_058170 | CNK00960 | -0.230003661 | 0.681547537 | pol II transcription elongation factor |
| CNXL_058180 | CNK00970 | 0.075400866 | 0.915837202 | hypothetical protein |
| CNXL_058190 | CNK00980 | -0.091998477 | 0.90384925 | prenylated SNARE protein Ykt6p |
| CNXL_058200 | CNK00990 | -0.384036425 | 0.54848728 | crossover junction endonuclease EME1 |
| CNXL_058210 | CNK01000 | -1.064752086 | 0.004661011 | hypothetical protein |
| CNXL_058220 | CNK01010 | 0.280979826 | 0.540193794 | hypothetical protein |
| CNXL_058230 | CNK01020 | 0.777484473 | 0.018087902 | putative TFIID and saga complex component |
| CNXL_058240 | CNK01030 | 0.440702475 | 0.365333703 | hypothetical protein |
| CNXL_058250 | CNK01040 | -0.619036373 | 0.077441507 | glutamate-tRNA ligase |
| CNXL_058260 | CNK01050 | 0.475899048 | 0.15786325 | hypothetical protein |
| CNXL_058270 | CNK01060 | 0.387238878 | 0.497668553 | 6-phosphogluconate dehydrogenase |
| CNXL_058280 | CNK01070 | 0.451970531 | 0.233441904 | hypothetical protein |
| CNXL_058290 | CNK01080 | 0.251721451 | 0.557606572 | glucose-6-phosphate 1-epimerase |
| CNXL_058300 | CNK01090 | 0.819825476 | 0.005625223 | hypothetical protein |
| CNXL_058310 | CNK01100 | 0.315502061 | 0.525585172 | ATP-binding cassette transporter |
| CNXL_058320 | CNK01120 | 0.066392538 | 0.945606531 | hypothetical protein |
| CNXL_058330 | CNK01130 | -0.064380477 | 0.927549471 | hypothetical protein |
| CNXL_058340 | CNK01140 | -0.077760622 | 0.899749891 | tip120-family protein |
| CNXL_058350 | CNK01150 | 0.109643929 | 0.843098307 | capsule-associated protein |
| CNXL_058360 | | 0.086159337 | 0.920669589 | polycomb protein e |
| CNXL_058370 | CNK01170 | 0.117574378 | 0.830161344 | Unknown |
| CNXL_058380 | CNK01190 | 1.091584485 | 2.86208E-05 | DNA repair protein Rad8 |
| CNXL_058390 | CNK01200 | -0.094213466 | 0.884028948 | hypothetical protein |
| CNXL_058400 | CNK01210 | 0.384970476 | 0.560961964 | hypothetical protein |
| CNXL_058410 | CNK01220 | 0.202931013 | 0.761797019 | hypothetical protein |
| CNXL_058420 | CNK01230 | 0.198563521 | 0.697146261 | cytoplasmic protein |
| CNXL_058430 | CNK01240 | -0.338626529 | 0.550258326 | hypothetical protein |
| CNXL_058440 | CNK01250 | -0.022047903 | 0.982102702 | peptide chain release factor 1 |
| CNXL_058450 | CNK01260 | 0.409682679 | 0.371669506 | nuclear protein |
| CNXL_058460 | | 0.037387633 | 0.993713943 | sterol esterase |
| CNXL_058470 | CNK01280 | -0.053164951 | 0.947835086 | Unknown |
| CNXL_058480 | CNK01290 | 0.512242292 | 0.151396722 | hypothetical protein |
| CNXL_058490 | CNK01300 | 0.930631376 | 0.014540957 | proteasome assembly chaperone 2 |
| CNXL_058500 | CNK01310 | -0.040411956 | 0.965101267 | hypothetical protein |
| CNXL_058510 | CNK01320 | 0.204066791 | 0.674850767 | origin recognition complex subunit 2 |
| CNXL_058520 | CNK01330 | 0.77086269 | 0.031508937 | replication factor C subunit 1 |
| CNXL_058530 | CNK01340 | 0.000616408 | 0.99867251 | calcium/proton exchanger |
| CNXL_058540 | CNK01350 | -0.154556708 | 0.793711563 | mitochondrial GTPase 1 |
| CNXL_058550 | CNK01360 | -0.137884399 | 0.827954543 | DNA-directed RNA polymerase I subunit RPA43 |
| CNXL_058560 | CNK01370 | -0.118742136 | 0.849285663 | small subunit ribosomal protein S16 |

| | | | | |
|---|---|---|---|---|
| CNXL_058570 | CNK01380 | 0.207135959 | 0.636723305 | hypothetical protein |
| CNXL_058580 | CNK01390 | 0.159665178 | 0.798267462 | hypothetical protein |
| CNXL_058590 | CNK01400 | -0.085812379 | 0.932765996 | hypothetical protein |
| CNXL_058600 | CNK01410 | -0.51474728 | 0.280743918 | pyridoxal 5'-phosphate synthase |
| CNXL_058610 | CNK01420 | -0.47729716 | 0.320878752 | hypothetical protein |
| CNXL_058620 | CNK01430 | -0.489992283 | 0.190652363 | threonine ammonia-lyase |
| CNXL_058630 | CNK01440 | -0.19354868 | 0.749039348 | DNA polymerase epsilon catalytic subunit A |
| CNXL_058640 | CNK01450 | 0.311992931 | 0.398174584 | hypothetical protein |
| CNXL_058650 | CNK01460 | -0.36954361 | 0.298784347 | translation machinery-associated protein 20 |
| CNXL_058660 | | 0.233765769 | 0.717347987 | translation initiation factor 3 subunit G |
| CNXL_058670 | CNK01480 | -0.098372707 | 0.874670769 | Unknown |
| CNXL_058680 | | -1.077284046 | 0.001373921 | hypothetical protein |
| CNXL_058690 | CNK01500 | 0.450367677 | 0.213024131 | hypothetical protein |
| CNXL_058700 | CNK01510 | 0.152364193 | 0.764330585 | hypothetical protein |
| CNXL_058710 | CNK01520 | 0.053774383 | 0.936507583 | hypothetical protein |
| CNXL_058720 | CNK01530 | 0.429844413 | 0.312160498 | cysteine protease ATG4 |
| CNXL_058730 | CNK01540 | -0.097501524 | 0.874846546 | ribonucleoside-diphosphate reductase subunit M2 |
| CNXL_058740 | | -0.30383736 | 0.535192809 | D-lactate dehydrogenase |
| CNXL_058750 | CNK01570 | 0.614678566 | 0.128619972 | Unknown |
| CNXL_058760 | CNK01580 | -0.093804714 | 0.87678601 | hypothetical protein |
| CNXL_058770 | CNK01590 | -0.107246079 | 0.842339591 | bud emergence protein 1 |
| CNXL_058780 | CNK01600 | -0.130680395 | 0.794331499 | pre-mRNA-splicing factor CEF1 |
| CNXL_058790 | CNK01610 | 1.145202961 | 0.000762557 | nonsense-mediated mRNA decay protein 3 |
| CNXL_058800 | CNK01620 | -0.685123308 | 0.040033951 | NAD dependent epimerase/dehydratase |
| CNXL_058810 | CNK01630 | -0.188806518 | 0.787613279 | hypothetical protein |
| CNXL_058820 | CNK01640 | -0.367079965 | 0.453722961 | CAMK/CAMKL/GIN4 protein kinase |
| CNXL_058830 | CNK01650 | 0.119630232 | 0.817522625 | hypothetical protein |
| CNXL_058840 | CNK01660 | -1.696677576 | 0.000213456 | cytoplasmic protein |
| CNXL_058850 | CNK01670 | -0.35363782 | 0.5737945 | hypothetical protein |
| CNXL_058860 | CNK01680 | 0.13857152 | 0.787613279 | mitochondrial distribution and morphology protein 31 |
| CNXL_058870 | CNK01700 | -0.066941121 | 0.91726998 | VPS15 protein kinase |
| CNXL_058880 | CNK01710 | 0.266129576 | 0.512844952 | hypothetical protein |
| CNXL_058890 | CNK01720 | -0.231609238 | 0.6186691 | hypothetical protein |
| CNXL_058900 | CNK01730 | 1.280363263 | 0.374018226 | hypothetical protein |
| CNXL_058910 | CNK01740 | -0.063524367 | 0.918783065 | hypothetical protein |
| CNXL_058920 | CNK01750 | -0.070221561 | 0.93451582 | cystathionine beta-lyase |
| CNXL_058930 | CNK01760 | 0.401817091 | 0.535369833 | hypothetical protein |
| CNXL_058940 | CNK01770 | -0.491668665 | 0.135403173 | hypothetical protein |
| CNXL_058950 | CNK01780 | 0.645176959 | 0.109223771 | hypothetical protein |
| CNXL_058960 | | 0.9161941 | 0.04517704 | pirin |
| CNXL_058970 | CNK01790 | -0.154924193 | 0.869733568 | Unknown |
| CNXL_058980 | CNK01800 | 0.923514467 | 0.015173028 | hypothetical protein |
| CNXL_058990 | CNK01810 | 0.227298687 | 0.662151848 | nitroreductase |
| CNXL_059000 | CNK01820 | 0.788040125 | 0.005374785 | solute carrier family 25 |
| CNXL_059010 | | 0.808394482 | 0.297349189 | hypothetical protein |
| CNXL_059020 | CNK01830 | 0.094747446 | 0.879408311 | hypothetical protein |
| CNXL_059030 | CNK01840 | 0.251472187 | 0.577988443 | hypothetical protein |
| CNXL_059040 | CNK01850 | 0.667465696 | 0.055129994 | exocyst protein |
| CNXL_059050 | CNK01860 | 0.293168729 | 0.525959757 | hypothetical protein |
| CNXL_059060 | CNK01870 | -0.176494058 | 0.803833694 | hypothetical protein |
| CNXL_059070 | CNK01880 | -0.328843774 | 0.481606389 | hypothetical protein |
| CNXL_059080 | CNK01890 | -1.075528949 | 0.023709223 | hypothetical protein |
| CNXL_059090 | CNK01900 | 0.232618784 | 0.62092322 | hypothetical protein |
| CNXL_059100 | CNK01910 | 0.004244499 | 0.995735232 | putative voltage-gated chloride channel |
| CNXL_059110 | CNK01920 | 0.038250882 | 0.964414864 | hypothetical protein |
| CNXL_059120 | | 0.095724881 | 0.962706186 | hypothetical protein |
| CNXL_059130 | CNK01940 | -0.098810018 | 0.878054651 | Unknown |
| CNXL_059140 | CNK01950 | -0.114460281 | 0.862241311 | prenylcysteine oxidase/farnesylcysteine lyase |

| | | | | |
|---|---|---|---|---|
| CNXL_059150 | CNK01960 | -0.061397461 | 0.921934941 | small subunit ribosomal protein S19 |
| CNXL_059160 | CNK01970 | -0.254777663 | 0.634728039 | T-complex protein 1 subunit gamma |
| CNXL_059170 | CNK01980 | 0.213575219 | 0.645032535 | Bud32 protein kinase |
| CNXL_059180 | CNK01990 | -0.249509883 | 0.567249149 | hypothetical protein |
| CNXL_059190 | CNK02000 | 0.141144829 | 0.813525402 | hypothetical protein |
| CNXL_059200 | CNK02010 | 0.728365287 | 0.023193034 | ornithine cyclodeaminase |
| CNXL_059210 | CNK02020 | 0.207140485 | 0.786945286 | 26S proteasome non-ATPase regulatory subunit 10 |
| CNXL_059220 | CNK02030 | -0.242480882 | 0.615722567 | hexose transporter |
| CNXL_059230 | CNK02040 | 0.156688502 | 0.807491917 | chromatin remodeling complex ATPase |
| CNXL_059240 | CNK02050 | -0.167000287 | 0.720207618 | hypothetical protein |
| CNXL_059250 | CNK02060 | -0.172627146 | 0.722427823 | hypothetical protein |
| CNXL_059260 | CNK02080 | 0.203977953 | 0.724256813 | hypothetical protein |
| CNXL_059270 | CNK02090 | -0.084040199 | 0.883841589 | protein KTI12 |
| CNXL_059280 | CNK02110 | 0.348703289 | 0.383307552 | hypothetical protein |
| CNXL_059290 | CNK02120 | -0.488618335 | 0.173210496 | prolactin regulatory element-binding protein |
| CNXL_059300 | CNK02130 | 1.169429659 | 0.09569924 | solute carrier family 31 |
| CNXL_059310 | CNK02140 | 0.030037881 | 0.969333741 | hypothetical protein |
| CNXL_059320 | CNK02150 | 0.273724459 | 0.538910254 | glutathione S-transferase |
| CNXL_059330 | CNK02160 | -0.060183773 | 0.943273857 | WD-repeat protein |
| CNXL_059340 | CNK02170 | -0.191842745 | 0.773704603 | TBC1 domain family member 5 |
| CNXL_059350 | CNK02180 | -0.576520901 | 0.198990236 | Unknown |
| CNXL_059360 | CNK02190 | -0.069799304 | 0.922230958 | GMP synthase [glutamine-hydrolyzing] |
| CNXL_059370 | CNK02200 | 0.19604066 | 0.657628563 | myo-inositol 2-dehydrogenase |
| CNXL_059380 | CNK02210 | 0.356785426 | 0.408738409 | cathepsin A |
| CNXL_059390 | CNK02220 | -0.2171477 | 0.685425894 | hypothetical protein |
| CNXL_059400 | CNK02230 | -0.408929847 | 0.4640116 | molecular chaperone GrpE |
| CNXL_059410 | | -0.384101382 | 0.528467452 | histone-lysine N-methyltransferase |
| CNXL_059420 | CNK02240 | -0.157099686 | 0.746537956 | Unknown |
| CNXL_059430 | CNK02250 | -0.149694998 | 0.813525402 | large subunit ribosomal protein L3 |
| CNXL_059440 | CNK02260 | 0.269769324 | 0.599348746 | TRAMP complex associated poly |
| CNXL_059450 | CNK02290 | 0.354204396 | 0.491722624 | hypothetical protein |
| CNXL_059460 | | 0.232803768 | 0.858055094 | hypothetical protein |
| CNXL_059470 | CNK02300 | 1.200397208 | 2.92983E-05 | Unknown |
| CNXL_059480 | CNK02310 | -0.588572806 | 0.120932459 | glutathione S-transferase |
| CNXL_059490 | CNK02320 | -0.153604784 | 0.851659386 | methionine synthase |
| CNXL_059500 | CNK02330 | 0.82536453 | 0.023966976 | RAD57 protein |
| CNXL_059510 | CNK02340 | 1.45746024 | 3.22609E-05 | hypothetical protein |
| CNXL_059520 | CNK02350 | -0.172721193 | 0.771781029 | glutathione S-transferase Gst3 |
| CNXL_059530 | CNK02360 | -0.64523474 | 0.173210496 | aspartate-tRNA ligase |
| CNXL_059540 | CNK02370 | 0.813561536 | 0.008144752 | hypothetical protein |
| CNXL_059550 | CNK02380 | 0.027221454 | 0.967512149 | alcohol dehydrogenase |
| CNXL_059560 | CNK02390 | -0.081851359 | 0.915039216 | bromodomain transcription factor |
| CNXL_059570 | CNK02400 | -0.017016189 | 0.987076518 | prefoldin alpha subunit |
| CNXL_059580 | CNK02410 | 0.087587495 | 0.879770554 | hypothetical protein |
| CNXL_059590 | | -0.919584265 | 0.830440343 | general transcriptional repressor |
| CNXL_059600 | CNK02430 | 0.109970902 | 0.876987041 | Unknown |
| CNXL_059610 | CNK02450 | 0.065954041 | 0.930990489 | Unknown |
| CNXL_059620 | CNK02460 | -0.236940146 | 0.643890318 | hypothetical protein |
| CNXL_059630 | CNK02470 | -0.92332869 | 0.004007963 | vacuolar amino acid transporter 5 |
| CNXL_059640 | NK02480\|CNK024 | 0.370317054 | 0.334783364 | serine/threonine protein kinase |
| CNXL_059650 | CNK02500 | -0.95520521 | 0.123590227 | mitochondrial distribution and morphology protein 12 |
| CNXL_059660 | CNK02510 | 0.301037638 | 0.56683643 | PLK/PLK1 protein kinase |
| CNXL_059670 | CNK02520 | -0.386041132 | 0.69908436 | uroporphyrinogen-III synthase |
| CNXL_059680 | CNK02530 | 0.441245399 | 0.309788097 | signal transducer |
| CNXL_059690 | CNK02540 | 0.564942459 | 0.113351667 | hypothetical protein |
| CNXL_059700 | CNK02550 | 0.792174989 | 0.013797215 | exocyst complex component |
| CNXL_059710 | CNK02560 | 1.147689451 | 0.002051794 | NAD+ synthetase |
| CNXL_059720 | CNK02570 | 0.080531259 | 0.91942407 | hypothetical protein |

| | | | | |
|---|---|---|---|---|
| CNXL_059730 | CNK02580 | -0.621561834 | 0.063932376 | mitochondrial protein |
| CNXL_059740 | CNK02590 | -0.605968852 | 0.176093948 | ribonucleoside-diphosphate reductase subunit M2 |
| CNXL_059750 | | 0.014926567 | 0.990703091 | DNA mismatch repair protein MSH6 |
| CNXL_059760 | CNK02600 | -0.630867171 | 0.168452319 | ribonuclease P protein subunit POP4 |
| CNXL_059770 | CNK02610 | 0.682082692 | 0.015173028 | cytoskeletal regulatory protein binding protein |
| CNXL_059780 | CNK02620 | 0.219159856 | 0.675584168 | hypothetical protein |
| CNXL_059790 | | 0.495829092 | 0.199401878 | ubiquitin precursor |
| CNXL_059800 | CNK02640 | 0.29712699 | 0.451211894 | alternative cyclin Pho80 |
| CNXL_059810 | CNK02650 | 1.30572166 | 0.220340117 | ATPase GET3 |
| CNXL_059820 | CNK02660 | 0.433599633 | 0.300219136 | hypothetical protein |
| CNXL_059830 | CNK02670 | 0.148147045 | 0.787481637 | hypothetical protein |
| CNXL_059840 | | 0.295967157 | 0.673070543 | peroxisomal targeting signal |
| CNXL_059850 | CNK02680 | 0.250172129 | 0.570755994 | hypothetical protein |
| CNXL_059860 | CNK02690 | -0.330623985 | 0.633439709 | xaa-Pro aminopeptidase |
| CNXL_059870 | CNK02700 | -0.207218282 | 0.6864136 | hypothetical protein |
| CNXL_059880 | | 0.949878658 | 0.249945191 | endopeptidase |
| CNXL_059890 | | 0.27401032 | 0.602550329 | hypothetical protein |
| CNXL_059900 | | 0.263922859 | 0.587146169 | Unknown |
| CNXL_059910 | CNK02720 | -0.308783365 | 0.49772658 | hypothetical protein |
| CNXL_059920 | CNK02730 | 0.203345758 | 0.678124378 | hypothetical protein |
| CNXL_059930 | | -1.023411821 | 0.253865879 | sugar transporter |
| CNXL_059940 | CNK02740 | -0.390666251 | 0.256825843 | hypothetical protein |
| CNXL_059950 | CNK02750 | -1.653520075 | 0.000258781 | serine/threonine protein kinase |
| CNXL_059960 | CNK02760 | 0.040855327 | 0.963190895 | hypothetical protein |
| CNXL_059970 | CNK02770 | 0.187419606 | 0.779355929 | alpha-amylase |
| CNXL_059980 | | 1.216785048 | 0.325451913 | glucan synthesis regulatory protein |
| CNXL_059990 | CNK02780 | -0.216735757 | 0.675025545 | hypothetical protein |
| CNXL_060000 | CNK02800 | 1.023103492 | 0.000455937 | hypothetical protein |
| CNXL_060010 | CNK02810 | 0.310940854 | 0.637722771 | Unknown |
| CNXL_060020 | CNK02820 | 0.101393223 | 0.885831771 | Unknown |
| CNXL_060030 | | 0.628991167 | 0.105729517 | Unknown |
| CNXL_060040 | CNK02830 | 0.697606707 | 0.023966976 | Unknown |
| CNXL_060050 | CNK02840 | -0.304929082 | 0.804701377 | Unknown |
| CNXL_060060 | CNK02850 | -0.430835823 | 0.337036418 | Unknown |
| CNXL_060070 | CNK02860 | 0.177215433 | 0.804701377 | allantoate permease |
| CNXL_060080 | CNK02870 | -0.388424458 | 0.364513417 | 2 |
| CNXL_060090 | CNK02880 | -0.009410832 | 0.992176872 | nuclear protein |
| CNXL_060100 | CNK02890 | 0.09302775 | 0.913786654 | chlorophyll synthesis pathway protein BchC |
| CNXL_060110 | | -0.236820071 | 0.576514302 | hypothetical protein |
| CNXL_060120 | CNK02910 | -0.069963998 | 0.923938002 | Unknown |
| CNXL_060130 | | 0.375796069 | 0.678724355 | aryl-alcohol dehydrogenase |
| CNXL_060140 | CNK02930 | 0.087555276 | 0.954346886 | hypothetical protein |
| CNXL_060150 | CNK02940 | 0.581554388 | 0.475068322 | aldo-keto reductase |
| CNXL_060160 | | -0.084764264 | 0.934642219 | alcohol dehydrogenase |
| CNXL_060170 | CNK02950 | 0.091665244 | 0.885449489 | hypothetical protein |
| CNXL_060180 | CNK02960 | -0.034230664 | 0.96275474 | 20S proteasome subunit beta 7 |
| CNXL_060190 | CNK02970 | -0.027647002 | 0.973737268 | het-c2 protein |
| CNXL_060200 | CNK02980 | 0.488783012 | 0.229819467 | condensin complex subunit 1 |
| CNXL_060210 | CNK02990 | 0.390441037 | 0.329557168 | efflux protein EncT |
| CNXL_060220 | CNK03000 | -0.252654052 | 0.746191268 | MSP domain-containing protein |
| CNXL_060230 | CNK03010 | -0.211078131 | 0.678724355 | ubiquinone biosynthesis protein COQ4 |
| CNXL_060240 | CNK03020 | -0.354662726 | 0.452146814 | CMP/dCMP deaminase zinc-binding protein |
| CNXL_060250 | CNK03030 | -2.114479888 | 2.55546E-07 | OPT family small oligopeptide transporter |
| CNXL_060260 | | 0.944713594 | 0.029865442 | hypothetical protein |
| CNXL_060270 | | 1.291111892 | 0.021077225 | Unknown |
| CNXL_060280 | CNK03050 | 0.266843462 | 0.557382058 | Unknown |
| CNXL_060290 | | -0.150023803 | 0.830440343 | hypothetical protein |
| CNXL_060300 | CNK03060 | -0.061737121 | 0.931882065 | Unknown |

| | | | | |
|---|---|---|---|---|
| CNXL_060310 | CNK03070 | -0.28909633 | 0.51244137 | C2H2 zinc finger protein |
| CNXL_060320 | CNK03080 | -0.19017476 | 0.787846251 | C2H2 zinc finger protein Zas1A |
| CNXL_060330 | CNK03090 | -0.059673999 | 0.929814702 | large subunit ribosomal protein L36 |
| CNXL_060340 | CNK03100 | -0.022396216 | 0.976006716 | helicase |
| CNXL_060350 | CNK03110 | -1.007252682 | 0.001699856 | large subunit ribosomal protein L23 |
| CNXL_060360 | CNK03120 | 0.016981385 | 0.986620478 | prenyl protein peptidase |
| CNXL_060370 | CNK03130 | 0.163476501 | 0.838712696 | transcription initiation factor TFIID subunit 10 |
| CNXL_060380 | CNK03140 | -0.488687119 | 0.329732144 | hypothetical protein |
| CNXL_060390 | CNK03150 | -1.656243957 | 4.13807E-06 | sulfide:quinone oxidoreductase |
| CNXL_060400 | | 0.058484323 | 0.939627791 | hypothetical protein |
| CNXL_060410 | CNK03160 | 0.143814242 | 0.795546534 | Unknown |
| CNXL_060420 | CNK03170 | 0.386207515 | 0.299063218 | Unknown |
| CNXL_060430 | CNK03180 | -0.497178639 | 0.224280017 | transaldolase |
| CNXL_060440 | | 0.964907522 | 0.003502057 | hypothetical protein |
| CNXL_060450 | CNK03190 | 0.423962381 | 0.337023834 | hypothetical protein |
| CNXL_060460 | CNK03200 | -0.225345521 | 0.650959369 | carbohydrate binding protein |
| CNXL_060470 | CNK03220 | 0.181769628 | 0.831707807 | hypothetical protein |
| CNXL_060480 | CNK03230 | -0.017633368 | 0.981851244 | hypothetical protein |
| CNXL_060490 | CNK03240 | -0.431202231 | 0.276390331 | small subunit ribosomal protein S5 |
| CNXL_060500 | NK03260\|CNK032 | 0.682998728 | 0.186490236 | cytochrome c oxidase subunit 4 |
| CNXL_060510 | CNK03270 | 0.651906227 | 0.020338592 | hypothetical protein |
| CNXL_060520 | CNK03290 | -0.011937248 | 0.991508881 | hypothetical protein |
| CNXL_060530 | CNK03310 | -0.599610086 | 0.034407486 | hypothetical protein |
| CNXL_060540 | CNK03320 | 0.061986152 | 0.927441884 | hypothetical protein |
| CNXL_060550 | CNK03330 | -0.195161861 | 0.75185116 | Unknown |
| CNXL_060560 | CNK03340 | -0.354828341 | 0.35231862 | prephenate dehydrogenase |
| CNXL_060570 | | -0.017371381 | 0.986881191 | V-type proton ATPase proteolipid subunit |
| CNXL_060580 | CNK03350 | 1.11054365 | 0.000518028 | hypothetical protein |
| CNXL_060590 | CNK03360 | 0.02086648 | 0.980972278 | short-chain dehydrogenase |
| CNXL_060600 | CNK03370 | 0.12299104 | 0.837479784 | inositol-polyphosphate 5-phosphatase |
| CNXL_060610 | CNK03380 | -0.269350662 | 0.690272467 | hypothetical protein |
| CNXL_060620 | | -0.237794031 | 0.626273098 | hypothetical protein |
| CNXL_060630 | CNK03400 | -0.048757979 | 0.97076745 | OTU domain-containing protein 6B |
| CNXL_060640 | CNK03410 | 0.906989398 | 0.007038182 | hypothetical protein |
| CNXL_060650 | CNK03420 | -0.375674363 | 0.419557544 | protein N-terminal amidase |
| CNXL_060660 | CNK03440 | -1.314573622 | 7.65019E-08 | adenylate kinase 1 |
| CNXL_060670 | CNK03450 | 0.091640757 | 0.918701468 | extensin |
| CNXL_060680 | CNK03460 | 0.556758564 | 0.120635533 | tartrate transporter |
| CNXL_060690 | CNK03470 | 0.135186403 | 0.941187478 | PQQ enzyme repeat protein |
| CNXL_060700 | | 0.043880867 | 0.981240834 | TPR repeat-containing protein |
| CNXL_060710 | | 0.640776567 | 0.083273575 | Unknown |
| CNXL_060720 | | 0.76376419 | 0.007186946 | Unknown |
| CNXL_060730 | CNH03010 | -0.431720085 | 0.521085092 | hypothetical protein |
| CNXL_060740 | CNH03000 | -0.20650919 | 0.667369865 | branched-chain-amino-acid transaminase |
| CNXL_060750 | CNH02990 | -0.086471643 | 0.881190377 | cell wall integrity protein scw1 |
| CNXL_060760 | CNH02980 | -0.274025476 | 0.519652797 | myo-inositol transporter |
| CNXL_060770 | CNH02970 | -0.977033516 | 0.010733247 | FACT complex subunit POB3 |
| CNXL_060780 | | -0.039041812 | NA | hypothetical protein |
| CNXL_060790 | CNH02960 | -0.083211576 | 0.902229878 | hypothetical protein |
| CNXL_060800 | CNH02950 | -0.269849554 | 0.581242232 | L-iditol 2-dehydrogenase |
| CNXL_060810 | CNH02940 | 0.575858533 | 0.395242285 | cytoplasmic protein |
| CNXL_060820 | CNH02930 | -0.274463284 | 0.74409679 | hypothetical protein |
| CNXL_060830 | CNH02920 | 0.528696275 | 0.754263305 | EF-hand calcium-binding protein |
| CNXL_060840 | CNH02910 | -0.06348308 | 0.912772876 | hypothetical protein |
| CNXL_060850 | CNH02900 | 0.611566105 | 0.098542885 | malate synthase |
| CNXL_060860 | | 0.041603689 | 0.961099251 | cytoplasmic protein |
| CNXL_060870 | CNH02890 | 0.159260181 | 0.770559628 | Unknown |
| CNXL_060880 | CNH02880 | 0.552734439 | 0.140677125 | ubiquitin carboxyl-terminal hydrolase |

| | | | | |
|---|---|---|---|---|
| CNXL_060890 | CNH02870 | 0.034793964 | 0.965966884 | hypothetical protein |
| CNXL_060900 | CNH02860 | 0.238886643 | 0.626703863 | glutaminyl cyclase |
| CNXL_060910 | CNH02850 | -0.577647157 | 0.199119063 | hypothetical protein |
| CNXL_060920 | CNH02840 | 0.779494903 | 0.006907933 | hypothetical protein |
| CNXL_060930 | CNH02830 | 0.048989101 | 0.939255348 | COP9 signalosome complex subunit 3 |
| CNXL_060940 | | 0.873494798 | 0.005301186 | 2-nitropropane dioxygenase |
| CNXL_060950 | CNH02820 | -0.002613123 | 0.996472611 | DNA polymerase delta subunit 4 |
| CNXL_060960 | | 0.439299429 | 0.629469502 | nuclear protein |
| CNXL_060970 | CNH02810 | 0.25584305 | 0.616861062 | hypothetical protein |
| CNXL_060980 | | 0.014363336 | 0.986620478 | metal iron transporter |
| CNXL_060990 | CNH02800 | 0.04021523 | 0.964266166 | Unknown |
| CNXL_061000 | CNH02790 | -0.121831715 | 0.830440343 | hypothetical protein |
| CNXL_061010 | CNH02780 | -0.574417879 | 0.13592695 | hypothetical protein |
| CNXL_061020 | CNH02770 | -0.265061765 | 0.575997802 | hypothetical protein |
| CNXL_061030 | CNH02760 | -0.274165696 | 0.732889168 | hypothetical protein |
| CNXL_061040 | CNH02750 | -0.528593212 | 0.243026936 | large subunit ribosomal protein L19 |
| CNXL_061050 | CNH02740 | -0.088410754 | 0.879531297 | protein phosphatase 2 |
| CNXL_061060 | | 1.454239724 | 3.05672E-05 | ubiquinol-cytochrome c reductase subunit 9 |
| CNXL_061070 | CNH02730 | -0.143460006 | 0.770005211 | hypothetical protein |
| CNXL_061080 | CNH02720 | -0.033139941 | 0.967295199 | NADH-ubiquinone oxidoreductase 49 kDa subunit |
| CNXL_061090 | CNH02710 | -0.160039522 | 0.809974479 | hypothetical protein |
| CNXL_061100 | CNH02700 | -0.271612465 | 0.555516318 | endoribonuclease YSH1 |
| CNXL_061110 | CNH02690 | -0.275082427 | 0.553008373 | hypothetical protein |
| CNXL_061120 | CNH02680 | 0.080757119 | 0.919822815 | ATP-dependent rRNA helicase SPB4 |
| CNXL_061130 | CNH02670 | -0.561232711 | 0.334301792 | hypothetical protein |
| CNXL_061140 | CNH02660 | -0.247922918 | 0.690261873 | hypothetical protein |
| CNXL_061150 | CNH02650 | -0.220329298 | 0.626204081 | hypothetical protein |
| CNXL_061160 | CNH02640 | -0.451125432 | 0.465890462 | chorismate synthase |
| CNXL_061170 | | 0.126185427 | 0.926942736 | histone demethylase |
| CNXL_061180 | CNH02630 | -0.300917984 | 0.55555809 | Unknown |
| CNXL_061190 | CNH02620 | 0.099821065 | 0.879531297 | hypothetical protein |
| CNXL_061200 | CNH02610 | 0.068426141 | 0.939400455 | folylpolyglutamate synthase |
| CNXL_061210 | CNH02600 | -0.265389386 | 0.570755994 | hypothetical protein |
| CNXL_061220 | CNH02590 | -0.289826546 | 0.516492158 | TPR repeat-containing protein |
| CNXL_061230 | CNH02580 | -0.599362539 | 0.249418771 | cleft lip and palate associated transmembrane protein |
| CNXL_061240 | CNH02570 | -0.010292074 | 0.990777629 | phosphatidylinositol glycan |
| CNXL_061250 | CNH02560 | -0.075200039 | 0.893955723 | ABC transporter |
| CNXL_061260 | CNH02550 | 0.273242961 | 0.594692631 | syntaxin 1B/2/3 |
| CNXL_061270 | CNH02540 | -0.046331287 | 0.945090468 | YbgI/family dinuclear metal center protein |
| CNXL_061280 | CNH02530 | 0.071546895 | 0.934642219 | hypothetical protein |
| CNXL_061290 | | 0.004412815 | 0.995735232 | hypothetical protein |
| CNXL_061300 | CNH02520 | 0.421072644 | 0.262705869 | hypothetical protein |
| CNXL_061310 | | -0.205769985 | 0.764293155 | cysteine synthase A |
| CNXL_061320 | CNH02510 | -0.373107191 | 0.356885743 | hypothetical protein |
| CNXL_061330 | | -0.812671233 | 0.000707933 | hypothetical protein |
| CNXL_061340 | CNH02500 | -0.15882622 | 0.827849849 | Unknown |
| CNXL_061350 | CNH02490 | 0.652810502 | 0.029044252 | hypothetical protein |
| CNXL_061360 | CNH02480 | 0.20946186 | 0.742187954 | cytoplasmic protein |
| CNXL_061370 | CNH02470 | 0.122283686 | 0.869733568 | ATP-dependent DNA helicase PIF1 |
| CNXL_061380 | | 0.328816026 | 0.567249149 | DEAD box family helicase |
| CNXL_061390 | CNH02460 | 0.206608517 | 0.684345861 | hypothetical protein |
| CNXL_061400 | CNH02450 | -0.140913963 | 0.803947851 | hypothetical protein |
| CNXL_061410 | CNH02440 | 0.258743094 | 0.638378602 | 1-pyrroline-5-carboxylate dehydrogenase |
| CNXL_061420 | CNH02430 | 0.155639419 | 0.762405378 | calpain-like protease palB/RIM13 |
| CNXL_061430 | CNH02420 | -0.375233914 | 0.247775422 | indigoidine synthase A family protein |
| CNXL_061440 | CNH02410 | 0.085794249 | 0.893673169 | hypothetical protein |
| CNXL_061450 | CNH02400 | -0.19184442 | 0.723317464 | hypothetical protein |
| CNXL_061460 | CNH02390 | 0.02434824 | 0.972638293 | hypothetical protein |

| | | | | |
|---|---|---|---|---|
| CNXL_061470 | CNH02380 | -2.371626069 | 2.47759E-20 | nuclear transport factor 2 |
| CNXL_061480 | | 0.213032189 | 0.647175553 | hypothetical protein |
| CNXL_061490 | CNH02370 | -0.098191944 | 0.868403117 | hypothetical protein |
| CNXL_061500 | CNH02360 | -0.309343577 | 0.602461418 | sarcoplasmic/endoplasmic reticulum Ca2+-ATPase |
| CNXL_061510 | | -0.367867182 | 0.609326629 | hypothetical protein |
| CNXL_061520 | CNH02350 | -0.371534409 | 0.472541632 | Unknown |
| CNXL_061530 | | -0.280939155 | 0.507813116 | two-component-like sensor kinase |
| CNXL_061540 | CNH02340 | -0.210336514 | 0.832551879 | hypothetical protein |
| CNXL_061550 | CNH02330 | -0.518983154 | 0.179759391 | hypothetical protein |
| CNXL_061560 | CNH02320 | -0.717736807 | 0.034915782 | hypothetical protein |
| CNXL_061570 | CNH02310 | -1.023933608 | 0.000176807 | hypothetical protein |
| CNXL_061580 | CNH02300 | 0.098983215 | 0.856366423 | hypothetical protein |
| CNXL_061590 | CNH02290 | -0.266160653 | 0.647175553 | arf/Sar family protein |
| CNXL_061600 | CNH02280 | -0.309197016 | 0.643890318 | methylthioribulose-1-phosphate dehydratase |
| CNXL_061610 | | 0.149612387 | 0.956685629 | ceramide glucosyltransferase |
| CNXL_061620 | CNH02270 | -0.193653621 | 0.685932518 | hypothetical protein |
| CNXL_061630 | | -0.441498698 | 0.437056582 | putative chitin synthase |
| CNXL_061640 | CNH02260 | -0.105753203 | 0.88268871 | hypothetical protein |
| CNXL_061650 | CNH02250 | 0.067787133 | 0.914338536 | hypothetical protein |
| CNXL_061660 | CNH02240 | 0.110981784 | 0.879811806 | hypothetical protein |
| CNXL_061670 | CNH02230 | -0.946473269 | 0.057123552 | hypothetical protein |
| CNXL_061680 | CNH02220 | 0.55104993 | 0.205950327 | hypothetical protein |
| CNXL_061690 | CNH02210 | 0.261862087 | 0.650959369 | hypothetical protein |
| CNXL_061700 | CNH02200 | -0.167950552 | 0.789040329 | phosphatidylinositol glycan |
| CNXL_061710 | | 0.897181558 | 0.003578481 | cytochrome c oxidase assembly protein subunit 17 |
| CNXL_061720 | CNH02180 | -0.574983024 | 0.110523004 | hypothetical protein |
| CNXL_061730 | CNH02170 | 0.057210197 | 0.936783812 | U3 small nucleolar RNA-associated protein 20 |
| CNXL_061740 | | 0.666819989 | 0.061154988 | hypothetical protein |
| CNXL_061750 | CNH02160 | 0.678058457 | 0.029821523 | hypothetical protein |
| CNXL_061760 | CNH02150 | -0.050058104 | 0.942099307 | septum formation protein Maf |
| CNXL_061770 | | 0.257695079 | 0.634469107 | serine-threonine protein phosphatase |
| CNXL_061780 | CNH02130 | -0.455209009 | 0.602461418 | hypothetical protein |
| CNXL_061790 | CNH02120 | -0.066731413 | 0.933786064 | hypothetical protein |
| CNXL_061800 | CNH02110 | 0.29550045 | 0.543220631 | peroxin-10 |
| CNXL_061810 | CNH02100 | -0.013565291 | 0.987964663 | class II histone deacetylase |
| CNXL_061820 | CNH02090 | -0.579290075 | 0.131030207 | parallel beta-helix repeat protein |
| CNXL_061830 | CNH02080 | 0.165312207 | 0.777833256 | hypothetical protein |
| CNXL_061840 | CNH02070 | -2.180646168 | 5.68863E-12 | hypothetical protein |
| CNXL_061850 | CNH02060 | 0.187601272 | 0.756597645 | serine/threonine protein kinase |
| CNXL_061860 | | -0.156838666 | 0.771763303 | mitochondrial outer membrane protein |
| CNXL_061870 | CNH02040 | -0.066092224 | 0.912929878 | hypothetical protein |
| CNXL_061880 | CNH02030 | -0.422990289 | 0.489809679 | large subunit ribosomal protein L7Ae |
| CNXL_061890 | CNH02020 | -0.557018876 | 0.273564602 | cytochrome b5 |
| CNXL_061900 | CNH02010 | -0.219564009 | 0.63198337 | hypothetical protein |
| CNXL_061910 | | -0.00744587 | 0.993713943 | transcription activator snf2l1 |
| CNXL_061920 | CNH01980 | -0.661380972 | 0.024610412 | hypothetical protein |
| CNXL_061930 | CNH01970 | -0.245215708 | 0.571422188 | hypothetical protein |
| CNXL_061940 | CNH01950 | -0.110043038 | 0.848823288 | CMGC/CDK protein kinase |
| CNXL_061950 | CNH01940 | -0.282974519 | 0.517950511 | hypothetical protein |
| CNXL_061960 | CNH01930 | 0.182004513 | 0.746537956 | hypothetical protein |
| CNXL_061970 | CNH01920 | 0.731760197 | 0.017843104 | bis |
| CNXL_061980 | CNH01910 | -0.913342299 | 0.050123903 | hypothetical protein |
| CNXL_061990 | | 0.570358266 | 0.210328192 | kinase regulator |
| CNXL_062000 | CNH01900 | 0.570779717 | 0.044950679 | Unknown |
| CNXL_062010 | CNH01890 | 0.043752103 | 0.949641665 | urease |
| CNXL_062020 | CNH01880 | -0.185198566 | 0.747814538 | hypothetical protein |
| CNXL_062030 | | 0.190260553 | 0.761797019 | DNAj-like cochaperone |
| CNXL_062040 | CNH01870 | -0.158418862 | 0.819555276 | DNA repair protein Rad18 |

| | | | | |
|---|---|---|---|---|
| CNXL_062050 | CNH01860 | -0.615756758 | 0.234630832 | hypothetical protein |
| CNXL_062060 | CNH01850 | 0.282427689 | 0.46380108 | hypothetical protein |
| CNXL_062070 | CNH01840 | -0.184242994 | 0.778158678 | putative pyridoxal reductase or aldo-keto reductase |
| CNXL_062080 | CNH01830 | 0.040316042 | 0.965966884 | hypothetical protein |
| CNXL_062090 | CNH01820 | 0.682139713 | 0.027361087 | arylformamidase |
| CNXL_062100 | CNH01800 | -0.30702171 | 0.553332333 | DNA excision repair protein ERCC-4 |
| CNXL_062110 | CNH01810 | -0.105753148 | 0.899291941 | hypothetical protein |
| CNXL_062120 | CNH01790 | -0.2400293 | 0.765354566 | hypothetical protein |
| CNXL_062130 | CNH01780 | -0.439061469 | 0.380612963 | hypothetical protein |
| CNXL_062140 | | 0.168142413 | 0.782048054 | senataxin |
| CNXL_062150 | CNH01770 | -0.218340892 | 0.659118995 | ino eighty subunit 2 |
| CNXL_062160 | CNH01760 | 0.665143992 | 0.059942113 | small subunit ribosomal protein S26 |
| CNXL_062170 | CNH01750 | -2.851037077 | 3.74008E-32 | glycogen storage control protein |
| CNXL_062180 | CNH01740 | -3.075380315 | 4.40517E-43 | hypothetical protein |
| CNXL_062190 | CNH01730 | 0.456019508 | 0.172797548 | hypothetical protein |
| CNXL_062200 | CNH01720 | 0.005118756 | 0.995735232 | aldose reductase |
| CNXL_062210 | CNH01710 | 0.38857589 | 0.403037151 | hypothetical protein |
| CNXL_062220 | CNH01700 | -0.811593664 | 0.044908745 | hypothetical protein |
| CNXL_062230 | CNH01690 | -0.141568722 | 0.781361786 | exocyst complex component 4 |
| CNXL_062240 | CNH01680 | 0.419014367 | 0.39931559 | solute carrier family 45 |
| CNXL_062250 | CNH01670 | -0.498012674 | 0.273415774 | hypothetical protein |
| CNXL_062260 | | -0.36494257 | 0.576514302 | hypothetical protein |
| CNXL_062270 | CNH01660 | -0.532301216 | 0.126147616 | hypothetical protein |
| CNXL_062280 | CNH01650 | -0.009126225 | 0.992176872 | septum-promoting GTP-binding protein 1 |
| CNXL_062290 | CNH01640 | -0.412954187 | 0.346301684 | hypothetical protein |
| CNXL_062300 | CNH01630 | -0.299349223 | 0.551291645 | ATPase |
| CNXL_062310 | CNH01620 | -0.451047339 | 0.298784347 | hypothetical protein |
| CNXL_062320 | CNH01610 | -0.504430933 | 0.461869386 | imidazoleglycerol-phosphate dehydratase |
| CNXL_062330 | | -0.091600838 | 0.886308648 | hypothetical protein |
| CNXL_062340 | | -0.029521501 | 0.965101267 | small subunit ribosomal protein S8 |
| CNXL_062350 | CNH01600 | 0.003706561 | 0.996158838 | hypothetical protein |
| CNXL_062360 | CNH01590 | 0.06914881 | 0.927636535 | hypothetical protein |
| CNXL_062370 | | -0.622515499 | 0.07093838 | serine/threonine-protein phosphatase 2A activator 2 |
| CNXL_062380 | | -1.01596798 | 0.267155276 | GDSL Lipase/Acylhydrolase |
| CNXL_062390 | | -0.411433777 | 0.634728039 | Unknown |
| CNXL_062400 | CNH01570 | -0.5258621 | 0.188739467 | Unknown |
| CNXL_062410 | CNH01560 | -0.074152067 | 0.913770009 | hypothetical protein |
| CNXL_062420 | | 0.32823637 | 0.415949769 | hypothetical protein |
| CNXL_062430 | | 0.195978683 | 0.719557989 | hypothetical protein |
| CNXL_062440 | | -0.169722292 | 0.837479784 | Unknown |
| CNXL_062450 | | -0.16577081 | 0.77300142 | Unknown |
| CNXL_062460 | CNH01540 | 0.32417165 | 0.557606572 | Unknown |
| CNXL_062470 | CNH01530 | 0.048482298 | 0.935544021 | hypothetical protein |
| CNXL_062480 | CNH01520 | 0.383957599 | 0.298784347 | dihydroxy-acid dehydratase |
| CNXL_062490 | | NA | NA | 2-isopropylmalate synthase |
| CNXL_062500 | | 1.200213589 | 0.425960037 | Unknown |
| CNXL_062510 | CNH01500 | 3.148731978 | NA | Unknown |
| CNXL_062520 | | -1.488160182 | NA | hypothetical protein |
| CNXL_062530 | | -1.440226686 | NA | Unknown |
| CNXL_062540 | | NA | NA | Unknown |
| CNXL_062550 | | NA | NA | hypothetical protein |
| CNXL_062560 | | NA | NA | hypothetical protein |
| CNXL_062570 | CNF03060 | -1.440226686 | NA | Unknown |
| CNXL_062580 | | -2.215719945 | NA | Unknown |
| CNXL_062590 | | NA | NA | Unknown |
| CNXL_062600 | | -2.453604166 | NA | Unknown |
| CNXL_062610 | | NA | NA | Unknown |
| CNXL_062620 | | -0.410724667 | 0.922015054 | Unknown |

| | | | | |
|---|---|---|---|---|
| CNXL_062630 | CNH01440 | -0.165688607 | 0.767511661 | Unknown |
| CNXL_062640 | | -0.25031029 | 0.710014173 | hypothetical protein |
| CNXL_062650 | CNH01430 | 0.054375402 | 0.935785373 | Unknown |
| CNXL_062660 | CNH01420 | 0.037245826 | 0.963420993 | tRNA |
| CNXL_062670 | CNH01410 | 0.11888787 | 0.86171371 | CAMKK/CAMKK-META protein kinase |
| CNXL_062680 | | 0.627980359 | 0.112183936 | prefoldin beta subunit |
| CNXL_062690 | CNH01400 | 0.14512942 | 0.779355929 | hypothetical protein |
| CNXL_062700 | CNH01390 | -0.111047822 | 0.863513463 | hexokinase |
| CNXL_062710 | CNH01380 | 0.576686238 | 0.09512427 | Fungal specific transcription factor |
| CNXL_062720 | CNH01370 | 0.128079221 | 0.848021859 | charged multivesicular body protein 2A |
| CNXL_062730 | | -0.416001561 | 0.521768339 | protein disulfide-isomerase |
| CNXL_062740 | CNH01360 | -0.130519737 | 0.822421189 | hypothetical protein |
| CNXL_062750 | | -0.626776666 | 0.056638444 | proteasome subunit alpha type-5 |
| CNXL_062760 | | 0.182203245 | 0.807491917 | TRIAD3 |
| CNXL_062770 | CNH01340 | -0.052288885 | 0.947835086 | Unknown |
| CNXL_062780 | CNH01330 | 0.161574154 | 0.807290237 | hypothetical protein |
| CNXL_062790 | | 0.44113658 | 0.424504154 | meiotic recombination protein SPO11 |
| CNXL_062800 | CNH01320 | -0.104022432 | 0.865614892 | Unknown |
| CNXL_062810 | | 1.072424789 | 0.540193794 | nucleolar complex protein 2 |
| CNXL_062820 | CNH01310 | 0.850695774 | 0.004892746 | Unknown |
| CNXL_062830 | CNH01300 | 0.433423193 | 0.165493103 | alpha-glucosidase |
| CNXL_062840 | CNH01290 | -0.907642843 | 0.008775795 | ABC transporter family protein |
| CNXL_062850 | CNH01280 | -0.113811021 | 0.871700589 | e3 ubiquitin-protein ligase |
| CNXL_062860 | | 0.156865808 | 0.927504878 | AP endonuclease 1 |
| CNXL_062870 | | 0.070640396 | 0.964414864 | hypothetical protein |
| CNXL_062880 | | -1.898914353 | 3.53286E-15 | hypothetical protein |
| CNXL_062890 | CNH01270 | -0.258297231 | 0.512707988 | hypothetical protein |
| CNXL_062900 | CNH01260 | 0.066785795 | 0.920669589 | G-protein beta-like rack1 protein homolog |
| CNXL_062910 | CNH01250 | 0.189896061 | 0.72764662 | hypothetical protein |
| CNXL_062920 | CNH01240 | 0.410097087 | 0.243168728 | hypothetical protein |
| CNXL_062930 | CNH01230 | -0.173961175 | 0.857954608 | cytochrome b5 reductase |
| CNXL_062940 | CNH01220 | -0.159180274 | 0.764293155 | L-lactate dehydrogenase |
| CNXL_062950 | CNH01210 | -0.467114616 | 0.309241115 | 2-dehydropantoate 2-reductase |
| CNXL_062960 | CNH01200 | 0.185959393 | 0.800552138 | Pi-transporter A-1 |
| CNXL_062970 | CNH01190 | -0.491103081 | 0.161712657 | endo-1 |
| CNXL_062980 | CNH01180 | 0.120584812 | 0.865614892 | pentatricopeptide repeat protein |
| CNXL_062990 | CNH01170 | -0.023113981 | 0.973737413 | hypothetical protein |
| CNXL_063000 | CNH01160 | -0.534461997 | 0.193111431 | Unknown |
| CNXL_063010 | | 1.200191116 | 2.21694E-05 | hypothetical protein |
| CNXL_063020 | CNH01140 | 1.932266896 | 1.12508E-09 | hypothetical protein |
| CNXL_063030 | | 1.416579146 | 0.004950976 | copper metallothionein 1 |
| CNXL_063040 | | -0.385948816 | 0.61372863 | Unknown |
| CNXL_063050 | CNH01130 | 0.386271984 | 0.405021185 | Unknown |
| CNXL_063060 | CNH01120 | -0.097616671 | 0.874803693 | elongin-A |
| CNXL_063070 | CNH01110 | -0.44704326 | 0.317388446 | kexin |
| CNXL_063080 | CNH01100 | -0.227877054 | 0.779355929 | smooth muscle cell associated protein-1 isoform 2 |
| CNXL_063090 | | -0.362865681 | 0.927441884 | NADPH dehydrogenase |
| CNXL_063100 | CNH01090 | 0.103722378 | 0.854840132 | Unknown |
| CNXL_063110 | CNH01080 | 0.019209115 | 0.983499434 | TATA-box-binding protein |
| CNXL_063120 | CNH01070 | 0.279632951 | 0.566540169 | lipid particle protein |
| CNXL_063130 | CNH01060 | 0.689245667 | 0.031370426 | pyridoxamine 5'-phosphate oxidase |
| CNXL_063140 | CNH01050 | 0.052314581 | 0.955072549 | CAMK/CAMK1/CAMK1-CMK protein kinase |
| CNXL_063150 | CNH01040 | 0.014207507 | 0.985595171 | hypothetical protein |
| CNXL_063160 | | -0.049574752 | 0.95072778 | nascent polypeptide-associated complex subunit beta |
| CNXL_063170 | CNH01030 | -0.24585571 | 0.626374497 | hypothetical protein |
| CNXL_063180 | CNH01020 | -0.330422532 | 0.732149774 | NADH dehydrogenase |
| CNXL_063190 | | 0.942148093 | 0.008086001 | hypothetical protein |
| CNXL_063200 | CNH00990 | 0.740269805 | 0.07127953 | Unknown |

| | | | | |
|---|---|---|---|---|
| CNXL_063210 | CNH00980 | -0.71737973 | 0.106221376 | myo-inositol transporter |
| CNXL_063220 | CNH00970 | 0.594754462 | 0.086895363 | Unknown |
| CNXL_063230 | | 0.352890111 | 0.417866535 | C2H2 zinc finger transcription factor |
| CNXL_063240 | CNH00950 | -0.143708406 | 0.842044068 | Unknown |
| CNXL_063250 | CNH00940 | -0.225878952 | 0.656993267 | hypothetical protein |
| CNXL_063260 | CNH00935 | -3.125385204 | 2.34998E-20 | putative TFIID and saga complex component |
| CNXL_063270 | CNH00920 | -0.412957511 | 0.286496105 | Unknown |
| CNXL_063280 | CNH00910 | -0.256551405 | 0.497362585 | asparagine synthase |
| CNXL_063290 | CNH00880 | 0.107887704 | 0.843283188 | hypothetical protein |
| CNXL_063300 | | 0.053670319 | 0.95265881 | virulence related protein of unknown function |
| CNXL_063310 | CNH00870 | -0.657847339 | 0.047437309 | hypothetical protein |
| CNXL_063320 | CNH00860 | 0.732047816 | 0.019085498 | RNA polymerase II transcription factor |
| CNXL_063330 | | -0.81534688 | 0.190326365 | hypothetical protein |
| CNXL_063340 | CNH00830 | -0.112650136 | 0.848021859 | hypothetical protein |
| CNXL_063350 | CNH00820 | -0.431912814 | 0.305084524 | pre-mRNA-processing protein 45 |
| CNXL_063360 | CNH00810 | -0.05202777 | 0.953095191 | hypothetical protein |
| CNXL_063370 | CNH00800 | -1.229775712 | 3.53533E-06 | hypothetical protein |
| CNXL_063380 | | -4.528217272 | 2.78808E-92 | phosphatidylinositol glycan |
| CNXL_063390 | CNH00790 | -0.157393206 | 0.746537956 | hypothetical protein |
| CNXL_063400 | CNH00780 | 0.334570598 | 0.372306093 | endoglucanase |
| CNXL_063410 | CNH00770 | -0.460535214 | 0.272050531 | hypothetical protein |
| CNXL_063420 | CNH00760 | -1.051935921 | 0.000300169 | replication factor C subunit 2/4 |
| CNXL_063430 | CNH00750 | -0.132666928 | 0.844766798 | hypothetical protein |
| CNXL_063440 | CNH00740 | 0.002501628 | 0.996833113 | hypothetical protein |
| CNXL_063450 | | -0.035330313 | 0.969333741 | cell cycle arrest protein BUB2 |
| CNXL_063460 | CNH00720 | -0.404342886 | 0.302177561 | hypothetical protein |
| CNXL_063470 | | -0.729570758 | 0.172380125 | histone-lysine N-methyltransferase SUV39H |
| CNXL_063480 | CNH00710 | -0.289109383 | 0.520208668 | ribonuclease P protein subunit RPR2 |
| CNXL_063490 | CNH00700 | -0.421951246 | 0.28631663 | protein CMS1 |
| CNXL_063500 | CNH00690 | -0.03819714 | 0.964355617 | hypothetical protein |
| CNXL_063510 | CNH00670 | -0.222984885 | 0.723024939 | minor histocompatibility antigen H13 |
| CNXL_063520 | CNH00660 | 0.418007835 | 0.370528273 | phosphoserine transaminase |
| CNXL_063530 | CNH00650 | 0.175445052 | 0.78773562 | hypothetical protein |
| CNXL_063540 | | -0.461222698 | 0.196920936 | LRP16 family protein |
| CNXL_063550 | CNH00630 | -0.620134332 | 0.160833876 | Unknown |
| CNXL_063560 | CNH00620 | -0.773501351 | 0.160213488 | rab guanyl-nucleotide exchange factor |
| CNXL_063570 | CNH00610 | -0.049425991 | 0.953145249 | cation:cation antiporter |
| CNXL_063580 | CNH00600 | -0.584851386 | 0.26023668 | ATPase |
| CNXL_063590 | | 0.342851174 | 0.623549135 | putative zinc finger transcription factor |
| CNXL_063600 | CNH00580 | 0.222287577 | 0.661428948 | Unknown |
| CNXL_063610 | CNH00570 | 0.35963188 | 0.417588807 | centromere protein C |
| CNXL_063620 | CNH00560 | 0.464389336 | 0.202621105 | hypothetical protein |
| CNXL_063630 | CNH00550 | 1.406370742 | 2.04358E-05 | sphinganine-1-phosphate aldolase |
| CNXL_063640 | CNH00540 | -0.240018351 | 0.638319466 | formamidopyrimidine-DNA glycosylase |
| CNXL_063650 | CNH00530 | 0.522733771 | 0.21653299 | galactose transporter |
| CNXL_063660 | CNH00520 | 0.106780108 | 0.870037788 | glutamate 5-kinase |
| CNXL_063670 | CNH00510 | 0.376066673 | 0.344162205 | hypothetical protein |
| CNXL_063680 | CNH00500 | 0.860328832 | 0.002690396 | CDP-diacylglycerol-inositol 3-phosphatidyltransferase |
| CNXL_063690 | CNH00490 | 1.423831198 | 0.000192889 | hypothetical protein |
| CNXL_063700 | CNH00480 | 0.336682761 | 0.432123344 | sugar transporter |
| CNXL_063710 | CNH00470 | 0.147011025 | 0.825693641 | hypothetical protein |
| CNXL_063720 | CNH00460 | 0.368518905 | 0.45163285 | regucalcin |
| CNXL_063730 | CNH00450 | 0.487377851 | 0.381984565 | 3-hydroxyacyl-CoA dehydrogenase |
| CNXL_063740 | CNH00440 | -0.384382521 | 0.633920399 | myo-inositol transporter |
| CNXL_063750 | CNH00430 | 0.087641521 | 0.884028948 | 3-dehydroshikimate dehydratase |
| CNXL_063760 | CNH00420 | -0.260193288 | 0.671115959 | hypothetical protein |
| CNXL_063770 | CNH00410 | 0.203028495 | 0.698369977 | chromosome transmission fidelity protein 18 |
| CNXL_063780 | CNH00400 | -1.748349561 | 2.11738E-09 | elongator complex protein 4 |

| | | | | |
|---|---|---|---|---|
| CNXL_063790 | CNH00390 | -0.383043318 | 0.385920716 | pr4/barwin domain protein |
| CNXL_063800 | | 0.193504185 | 0.746537956 | hypothetical protein |
| CNXL_063810 | CNH00370 | 0.246681769 | 0.700697818 | Unknown |
| CNXL_063820 | CNH00360 | 0.530616241 | 0.077611959 | beta-glucosidase |
| CNXL_063830 | | -0.950886025 | 0.549749504 | MIPC synthase |
| CNXL_063840 | CNH00350 | -0.14127151 | 0.837479784 | Unknown |
| CNXL_063850 | CNH00340 | -0.073995039 | 0.904401612 | hypothetical protein |
| CNXL_063860 | CNH00330 | 0.08272161 | 0.893955723 | translation initiation factor 2A |
| CNXL_063870 | CNH00320 | -0.15558639 | 0.753998183 | ribose-phosphate pyrophosphokinase |
| CNXL_063880 | CNH00300 | 0.37821085 | 0.414496592 | hypothetical protein |
| CNXL_063890 | CNH00290 | 0.124450652 | 0.81790448 | pre-mRNA-splicing factor SYF2 |
| CNXL_063900 | | 0.279822477 | 0.605616298 | CMGC/CDK/CDK5 protein kinase |
| CNXL_063910 | CNH00280 | -0.163501991 | 0.76831241 | hypothetical protein |
| CNXL_063920 | CNH00270 | 0.129609475 | 0.850005462 | ATP-dependent RNA helicase MAK5 |
| CNXL_063930 | | 0.041518414 | 0.952369888 | hypothetical protein |
| CNXL_063940 | NC00740\|CNI0132 | 1.484927388 | NA | Unknown |
| CNXL_063950 | | 0.577316263 | 0.125443586 | Unknown |
| CNXL_063960 | | 1.37449672 | 0.113349233 | hypothetical protein |
| CNXL_063970 | CNH00230 | 0.334297491 | 0.500319755 | hypothetical protein |
| CNXL_063980 | CNH00220 | -0.057921649 | 0.923639918 | cytoplasmic protein |
| CNXL_063990 | CNH00210 | 0.078262301 | 0.920669589 | e3 ubiquitin-protein ligase |
| CNXL_064000 | CNH00190 | -0.207111872 | 0.689555844 | U4/U6.U5 tri-snRNP component SNU23 |
| CNXL_064010 | CNH00180 | -0.478255163 | 0.493812577 | nuclear pore complex protein Nup133 |
| CNXL_064020 | CNH00170 | -0.056519372 | 0.927441884 | acetyl-CoA transporter |
| CNXL_064030 | CNH00160 | -0.748338471 | 0.105214931 | putative phosphomannomutase |
| CNXL_064040 | CNH00150 | -0.071301495 | 0.916084262 | hypothetical protein |
| CNXL_064050 | CNH00140 | -0.051571196 | 0.934642219 | hypothetical protein |
| CNXL_064060 | CNH00130 | 0.496772819 | 0.19928113 | rho-like GTPase |
| CNXL_064070 | CNH00120 | 0.467527201 | 0.226132264 | hypothetical protein |
| CNXL_064080 | CNH00110 | 0.198726403 | 0.781580785 | peroxiredoxin 5 |
| CNXL_064090 | CNH00100 | -0.159087508 | 0.768292466 | hypothetical protein |
| CNXL_064100 | CNH00090 | -0.432720251 | 0.365784664 | hemolysin |
| CNXL_064110 | CNH00080 | 0.09414019 | 0.892724552 | hypothetical protein |
| CNXL_064120 | | -0.165105692 | 0.76831241 | hypothetical protein |
| CNXL_064130 | CNH00070 | -0.169058282 | 0.758650495 | hypothetical protein |
| CNXL_064140 | CNH00060 | 0.044176981 | 0.954307827 | monosaccharide transporter |
| CNXL_064150 | CNH00040 | 0.15185111 | 0.813598552 | Unknown |
| CNXL_064160 | | -0.614206588 | 0.534447681 | hypothetical protein |
| CNXL_064170 | CNH00030 | -0.978285038 | 0.004028003 | hypothetical protein |
| CNXL_064180 | CNH00020 | -0.135335576 | 0.829008241 | alpha-N-arabinofuranosidase |
| CNXL_064190 | | 0.542602801 | 0.102318129 | glucose transporter |
| CNXL_064200 | | -0.0661758 | 0.970775493 | hypothetical protein |
| CNXL_064210 | CNM00010 | 0.231549012 | 0.626170102 | Unknown |
| CNXL_064220 | | 1.063477131 | 0.000334848 | transmembrane transporter Liz1p |
| CNXL_064230 | CNM00030 | 1.088014463 | 0.001973863 | Unknown |
| CNXL_064240 | CNM00040 | -0.082346049 | 0.893955723 | multidrug transporter |
| CNXL_064250 | CNM00050 | -0.520486433 | 0.434265036 | hypothetical protein |
| CNXL_064260 | | 0.027018426 | 0.969527869 | solute carrier family 32 |
| CNXL_064270 | CNM00070 | 0.237224867 | 0.696473798 | hypothetical protein |
| CNXL_064280 | CNM00080 | -0.412287706 | 0.389045049 | rho-GTPase |
| CNXL_064290 | CNM00090 | -0.294451297 | 0.544657787 | peptide alpha-N-acetyltransferase |
| CNXL_064300 | CNM00100 | -0.449536575 | 0.430519244 | glycoprotein |
| CNXL_064310 | CNM00110 | -0.516869282 | 0.428821207 | phosphomevalonate kinase |
| CNXL_064320 | CNM00120 | 0.251091526 | 0.93451582 | tRNA |
| CNXL_064330 | CNM00130 | 0.285518456 | 0.619100215 | hypothetical protein |
| CNXL_064340 | CNM00140 | -0.700852513 | 0.137302949 | transcription initiation factor TFIIH subunit 3 |
| CNXL_064350 | CNM00150 | -0.188569947 | 0.735695735 | U3 small nucleolar RNA-associated protein 4 |
| CNXL_064360 | CNM00160 | -0.0876237 | 0.903834486 | hypothetical protein |

| | | | | |
|---|---|---|---|---|
| CNXL_064370 | NM00170\|CNM001 | -0.295317923 | 0.437062442 | U3 small nucleolar RNA-associated protein 23 |
| CNXL_064380 | | 0.16226187 | 0.915837202 | Unknown |
| CNXL_064390 | | -0.102642861 | 0.96580389 | Unknown |
| CNXL_064400 | CNM00200 | -0.39615635 | 0.725078077 | Unknown |
| CNXL_064410 | CNA04900 | -0.116760388 | 0.845033033 | hypothetical protein |
| CNXL_064420 | CNM00220 | 0.411049282 | 0.2231022 | hypothetical protein |
| CNXL_064430 | CNM00230 | -0.34144031 | 0.393551864 | aldehyde dehydrogenase |
| CNXL_064440 | CNM00240 | -0.016344559 | 0.984786918 | hypothetical protein |
| CNXL_064450 | CNM00250 | -0.312583829 | 0.58959601 | NADH-cytochrome b5 reductase 1 |
| CNXL_064460 | | 0.080179098 | 0.946722381 | vacuolar protein |
| CNXL_064470 | CNM00260 | -0.688960364 | 0.050491567 | hypothetical protein |
| CNXL_064480 | CNM00270 | -0.310944018 | 0.434588143 | alpha-1 |
| CNXL_064490 | | -0.751357903 | 0.157787273 | Unknown |
| CNXL_064500 | CNM00290 | 0.591016132 | 0.072939214 | Unknown |
| CNXL_064510 | CNM00300 | -0.42098014 | 0.309241115 | aldehyde dehydrogenase |
| CNXL_064520 | CNM00310 | 1.651945838 | 6.92707E-07 | hypothetical protein |
| CNXL_064530 | CNM00320 | 0.646684495 | 0.052938787 | hypothetical protein |
| CNXL_064540 | CNM00330 | 0.097531311 | 0.890150525 | rab GTPase activator |
| CNXL_064550 | CNM00340 | 0.30030673 | 0.617084227 | trans-aconitate 3-methyltransferase |
| CNXL_064560 | CNM00350 | -0.077258042 | 0.914338536 | hypothetical protein |
| CNXL_064570 | CNM00360 | -0.125453751 | 0.810102246 | peroxin-14 |
| CNXL_064580 | CNM00370 | 0.502711067 | 0.276547748 | aspartate aminotransferase |
| CNXL_064590 | CNM00380 | -1.132862792 | 0.008286585 | aryl-alcohol dehydrogenase |
| CNXL_064600 | CNM00390 | -0.428669377 | 0.403618844 | hypothetical protein |
| CNXL_064610 | CNM00400 | 0.719119329 | 0.009547259 | peptidyl-tRNA hydrolase ICT1 |
| CNXL_064620 | CNM00410 | -0.701402231 | 0.095150076 | hypothetical protein |
| CNXL_064630 | CNM00440 | -0.864187926 | 0.085416549 | Unknown |
| CNXL_064640 | | -0.255267708 | 0.595126631 | ADP-ribosylation factor-like 2 |
| CNXL_064650 | | -0.181777689 | 0.78913258 | pfkB family carbohydrate kinase superfamily |
| CNXL_064660 | CNM00460 | -0.449897155 | 0.140371229 | Unknown |
| CNXL_064670 | CNM00470 | -0.00871661 | 0.991508881 | allantoin permease |
| CNXL_064680 | | 0.04360923 | 0.962640129 | alcohol dehydrogenase |
| CNXL_064690 | CNM00480 | 0.10314694 | 0.848823288 | Unknown |
| CNXL_064700 | | NA | NA | hypothetical protein |
| CNXL_064710 | | NA | NA | Unknown |
| CNXL_064720 | | 0.470847034 | 0.248506283 | Unknown |
| CNXL_064730 | | 0.569492845 | NA | hypothetical protein |
| CNXL_064740 | | 0.536905408 | 0.193620149 | Unknown |
| CNXL_064750 | CNM00580 | 0.212346391 | 0.686488755 | Unknown |
| CNXL_064760 | CNM00590 | 0.060774472 | 0.931173948 | hypothetical protein |
| CNXL_064770 | CNM00600 | 0.854561383 | 0.081559791 | GTP-binding protein ryh1 |
| CNXL_064780 | CNM00610 | 0.534613628 | 0.071669676 | UDP-glucose 4-epimerase |
| CNXL_064790 | CNM00620 | 0.209216067 | 0.679255446 | galactokinase |
| CNXL_064800 | CNM00630 | 0.542940038 | 0.616004825 | galactose-1-phosphate uridyl transferase |
| CNXL_064810 | CNM00640 | -0.15698188 | 0.863559696 | MFS transporter |
| CNXL_064820 | CNM00650 | -0.163628741 | 0.828543224 | hypothetical protein |
| CNXL_064830 | CNM00660 | -0.083589335 | 0.931292368 | hypothetical protein |
| CNXL_064840 | CNM00670 | -0.099401873 | 0.887005373 | hypothetical protein |
| CNXL_064850 | | -0.014063547 | 0.991133859 | protein DGCR14 |
| CNXL_064860 | CNM00690 | -1.467987983 | 3.03959E-09 | Unknown |
| CNXL_064870 | CNM00700 | -0.485019001 | 0.13866828 | hypothetical protein |
| CNXL_064880 | | -0.068553978 | 0.936783812 | translation initiation factor 3 subunit E |
| CNXL_064890 | CNM00720 | -0.85652197 | 0.123590227 | hypothetical protein |
| CNXL_064900 | CNM00730 | 1.228586621 | 0.000176618 | cytochrome c oxidase assembly protein subunit 15 |
| CNXL_064910 | CNM00740 | 0.672645828 | 0.039699682 | protein tyrosine phosphatase |
| CNXL_064920 | CNM00760 | 0.607704106 | 0.267783676 | inositol polyphosphate-5-phosphatase F |
| CNXL_064930 | CNM00770 | 0.176970626 | 0.814220979 | nucleoside-diphosphate-sugar epimerase |
| CNXL_064940 | CNM00780 | -0.170449988 | 0.771763303 | hypothetical protein |

| | | | | |
|---|---|---|---|---|
| CNXL_064950 | CNM00790 | 0.24720277 | 0.632998683 | hypothetical protein |
| CNXL_064960 | | 0.167969438 | 0.798976008 | hypothetical protein |
| CNXL_064970 | CNM00800 | 0.241262326 | 0.570755994 | Unknown |
| CNXL_064980 | CNM00810 | -0.331462803 | 0.556511753 | AAT family amino acid transporter |
| CNXL_064990 | CNM00820 | -0.057523108 | 0.927441884 | hypothetical protein |
| CNXL_065000 | CNM00830 | 1.420429452 | 3.22074E-05 | chorismate mutase |
| CNXL_065010 | CNM00840 | 0.92132502 | 0.004007189 | cytoplasmic protein |
| CNXL_065020 | CNM00850 | 0.241132047 | 0.635378645 | hypothetical protein |
| CNXL_065030 | CNM00860 | 0.148654655 | 0.776955714 | cytoplasmic protein |
| CNXL_065040 | CNM00870 | 0.271536387 | 0.613436396 | mRNA export factor |
| CNXL_065050 | CNM00880 | -0.188508141 | 0.703818084 | hypothetical protein |
| CNXL_065060 | CNM00890 | 0.030408969 | 0.972638293 | proliferating cell nuclear antigen |
| CNXL_065070 | | -0.122491458 | 0.858583021 | inositol/phosphatidylinositol phosphatase |
| CNXL_065080 | CNM00900 | -0.337257591 | 0.412831796 | Unknown |
| CNXL_065090 | CNF04550 | 0.002623856 | 0.997686984 | glucose oxidase |
| CNXL_065100 | CNM00910 | -0.285835383 | 0.510006134 | Unknown |
| CNXL_065110 | CNM00920 | -0.362631373 | 0.411990901 | delayed-type hypersensitivity antigen |
| CNXL_065120 | CNM00930 | 0.242419864 | 0.664165041 | phospholipase B |
| CNXL_065130 | CNM00940 | -0.095367118 | 0.902229878 | CMGC/CDK/CDK8 protein kinase |
| CNXL_065140 | CNM00950 | -0.010997278 | 0.988641003 | kinetochore protein Spc7/SPC105 |
| CNXL_065150 | CNM00970 | -0.204836265 | 0.708500047 | aspartate aminotransferase |
| CNXL_065160 | CNM00990 | -0.283761147 | 0.46380108 | hypothetical protein |
| CNXL_065170 | CNM01000 | -0.331530725 | 0.437056582 | cyclin 1 |
| CNXL_065180 | | -0.168249235 | 0.814514438 | copII vesicle coat protein |
| CNXL_065190 | CNM01020 | -0.221072788 | 0.612359199 | hypothetical protein |
| CNXL_065200 | CNM01030 | 0.295298026 | 0.453868281 | large subunit ribosomal protein L13e |
| CNXL_065210 | CNM01040 | -0.41269568 | 0.36733274 | tricarboxylate carrier |
| CNXL_065220 | CNM01050 | 0.674757728 | 0.059237283 | hypothetical protein |
| CNXL_065230 | CNM01060 | 0.582917931 | 0.562038835 | glucosamine-6-phosphate deaminase |
| CNXL_065240 | CNM01070 | 0.113083783 | 0.842339591 | hypothetical protein |
| CNXL_065250 | CNM01080 | 0.794244286 | 0.00232079 | hypothetical protein |
| CNXL_065260 | CNM01090 | 1.120810878 | 0.000297907 | ADP |
| CNXL_065270 | CNM01100 | -0.131925636 | 0.805915487 | ADP |
| CNXL_065280 | CNM01110 | -1.093193332 | 0.007760959 | RNA binding protein |
| CNXL_065290 | | 0.472485525 | 0.593397075 | hypothetical protein |
| CNXL_065300 | CNM01120 | 0.209125496 | 0.64118547 | hypothetical protein |
| CNXL_065310 | CNM01130 | 0.301439812 | 0.434147477 | chaperone regulator |
| CNXL_065320 | CNM01150 | 1.846888518 | 0.068693686 | G protein beta subunit-like |
| CNXL_065330 | CNM01160 | 0.173445441 | 0.771763303 | hypothetical protein |
| CNXL_065340 | CNM01170 | -0.474789511 | 0.279637638 | hypothetical protein |
| CNXL_065350 | CNM01180 | -0.159329019 | 0.789076022 | hypothetical protein |
| CNXL_065360 | CNM01190 | 0.387978868 | 0.271362906 | hypothetical protein |
| CNXL_065370 | CNM01200 | -0.257624635 | 0.814220979 | rab family protein |
| CNXL_065380 | CNM01210 | -0.552719536 | 0.219945567 | acid phosphatase |
| CNXL_065390 | CNM01220 | -0.727988037 | 0.005522962 | hypothetical protein |
| CNXL_065400 | CNM01230 | -0.080564106 | 0.916084262 | hypothetical protein |
| CNXL_065410 | CNM01240 | -0.075589439 | 0.915791262 | hypothetical protein |
| CNXL_065420 | CNM01250 | 0.150374751 | 0.829025438 | hypothetical protein |
| CNXL_065430 | CNM01260 | 0.451005102 | 0.366302025 | spermine transporter |
| CNXL_065440 | CNM01270 | 1.355727735 | 7.62605E-05 | cardiolipin synthase |
| CNXL_065450 | CNM01280 | -0.023857133 | 0.981240834 | hypothetical protein |
| CNXL_065460 | CNM01290 | -0.48278696 | 0.306457146 | glycerol-1-phosphatase |
| CNXL_065470 | CNM01300 | -0.499779899 | 0.227067905 | leucine-tRNA ligase |
| CNXL_065480 | CNM01310 | 0.005379465 | 0.995735232 | elongation factor 1-alpha |
| CNXL_065490 | CNM01320 | 0.062063692 | 0.92070335 | Pin2-interacting protein X1 |
| CNXL_065500 | CNM01330 | -0.006261751 | 0.995735232 | ribosome biogenesis protein NSA2 |
| CNXL_065510 | CNM01340 | 0.066696974 | 0.934677332 | hypothetical protein |
| CNXL_065520 | CNM01350 | -0.300724742 | 0.636752734 | cytoplasmic protein |

| | | | | |
|---|---|---|---|---|
| CNXL_065530 | CNM01360 | -0.042672092 | 0.954207791 | hypothetical protein |
| CNXL_065540 | CNM01370 | -3.433227388 | 9.55069E-20 | hypothetical protein |
| CNXL_065550 | CNM01380 | -0.650289951 | 0.023968696 | glycerol-1-phosphatase |
| CNXL_065560 | CNM01390 | -0.13405875 | 0.830652518 | bZip domain protein |
| CNXL_065570 | CNM01400 | -0.187811498 | 0.781470235 | hypothetical protein |
| CNXL_065580 | CNM01410 | 0.197368368 | 0.806051382 | NADH dehydrogenase |
| CNXL_065590 | CNM01420 | 0.056968173 | 0.920669589 | hypothetical protein |
| CNXL_065600 | CNM01430 | -1.203875143 | 0.061154988 | long-chain fatty acid transporter |
| CNXL_065610 | CNM01440 | -0.71934685 | 0.237021921 | dUTP pyrophosphatase |
| CNXL_065620 | CNM01450 | -0.15196682 | 0.837479784 | DNA polymerase alpha subunit B |
| CNXL_065630 | CNM01460 | -0.032782653 | 0.960513489 | alkylated DNA repair protein AlkB |
| CNXL_065640 | CNM01470 | -0.297719881 | 0.612271528 | cytoplasmic protein |
| CNXL_065650 | CNM01480 | -0.299123598 | 0.473663995 | hypothetical protein |
| CNXL_065660 | CNM01490 | -0.383486589 | 0.521085092 | hypothetical protein |
| CNXL_065670 | CNM01500 | -0.059043315 | 0.948564415 | hypothetical protein |
| CNXL_065680 | CNM01510 | -0.238802567 | 0.717496849 | hypothetical protein |
| CNXL_065690 | CNM01520 | -1.354277553 | 7.60145E-08 | hypothetical protein |
| CNXL_065700 | CNM01530 | 0.184569147 | 0.732889168 | hsp90-like protein |
| CNXL_065710 | CNM01540 | 0.638090642 | 0.099838318 | histidine kinase two-component system |
| CNXL_065720 | CNM01550 | -0.088909089 | 0.890150525 | hypothetical protein |
| CNXL_065730 | CNM01560 | 0.745078462 | 0.024996607 | 26S protease regulatory subunit 6A-B |
| CNXL_065740 | CNM01570 | -0.091279077 | 0.889001755 | cytoplasmic protein |
| CNXL_065750 | CNM01580 | 0.049206329 | 0.947986968 | hypothetical protein |
| CNXL_065760 | CNM01590 | -0.573116817 | 0.107354436 | hypothetical protein |
| CNXL_065770 | CNM01600 | -0.12333496 | 0.867654918 | hypothetical protein |
| CNXL_065780 | CNM01610 | -3.651622013 | 3.75079E-41 | hypothetical protein |
| CNXL_065790 | CNM01620 | 0.117488224 | 0.868398587 | hypothetical protein |
| CNXL_065800 | CNM01630 | -0.022722315 | 0.978794024 | guanyl nucleotide exchange factor Sql2 |
| CNXL_065810 | CNM01640 | -0.303159569 | 0.700697818 | hypothetical protein |
| CNXL_065820 | | 0.022810149 | 0.982102702 | hypothetical protein |
| CNXL_065830 | CNM01650 | 0.441074276 | 0.275889973 | hypothetical protein |
| CNXL_065840 | CNM01660 | 0.040824845 | 0.954566056 | hypothetical protein |
| CNXL_065850 | | -0.116675753 | 0.948286148 | sterility protein Ste20 |
| CNXL_065860 | CNM01670 | -0.300374497 | 0.57446957 | Unknown |
| CNXL_065870 | | 0.321133812 | 0.58959601 | ATP-dependent DNA helicase MPH1 |
| CNXL_065880 | | 0.9307306 | 0.090086163 | Unknown |
| CNXL_065890 | CNM01680 | -0.260822212 | 0.499683579 | Unknown |
| CNXL_065900 | CNM01690 | 0.356478328 | 0.326055975 | metal homeostatis protein bsd2 |
| CNXL_065910 | CNM01700 | -2.365215059 | 0.000207187 | alcohol dehydrogenase |
| CNXL_065920 | CNM01710 | 1.985065814 | 8.49702E-09 | hypothetical protein |
| CNXL_065930 | CNM01720 | 1.177310239 | 9.30635E-05 | hypothetical protein |
| CNXL_065940 | NM01730\|CNM017 | 0.136839395 | 0.793711563 | S-formylglutathione hydrolase |
| CNXL_065950 | CNM01750 | 0.036457673 | 0.962706186 | Unknown |
| CNXL_065960 | CNM01760 | -0.017743732 | 0.982210446 | PEK/GCN2 protein kinase |
| CNXL_065970 | CNM01770 | -0.257091183 | 0.582678739 | 26S proteasome regulatory subunit N2 |
| CNXL_065980 | CNM01780 | -3.086725469 | 1.15099E-16 | aconitate hydratase |
| CNXL_065990 | CNM01790 | -0.348081819 | 0.498081161 | meiotic recombinase Dmc1 |
| CNXL_066000 | CNM01800 | -0.159176606 | 0.806051382 | hypothetical protein |
| CNXL_066010 | CNM01810 | -0.091534962 | 0.881169328 | cytoplasmic protein |
| CNXL_066020 | | 1.405571548 | 0.623549135 | NADH dehydrogenase |
| CNXL_066030 | CNM01820 | -1.469905528 | 2.90029E-06 | hypothetical protein |
| CNXL_066040 | CNM01830 | -0.188364735 | 0.798267462 | minichromosome maintenance protein 4 |
| CNXL_066050 | CNM01840 | -0.125335299 | 0.899804184 | origin recognition complex subunit 4 |
| CNXL_066060 | CNM01850 | 0.682595686 | 0.042438082 | hypothetical protein |
| CNXL_066070 | CNM01860 | -0.139565569 | 0.777403833 | hypothetical protein |
| CNXL_066080 | CNM01870 | 0.009577755 | 0.991351411 | hypothetical protein |
| CNXL_066090 | CNM01880 | 0.283263156 | 0.474540273 | streptomycin biosynthesis protein StrI |
| CNXL_066100 | CNM01890 | -0.044748684 | 0.962926966 | hypothetical protein |

| | | | | |
|---|---|---|---|---|
| CNXL_066110 | CNM01900 | -1.200007382 | 1.98946E-05 | N-acetylglucosamine-6-phosphate deacetylase |
| CNXL_066120 | CNM01910 | -0.291317201 | 0.535382959 | hypothetical protein |
| CNXL_066130 | CNM01920 | -0.843053321 | 0.007897375 | hypothetical protein |
| CNXL_066140 | CNM01930 | -0.736426224 | 0.560239377 | protein kinase |
| CNXL_066150 | CNM01940 | -0.345779263 | 0.510006134 | hypothetical protein |
| CNXL_066160 | CNM01950 | -0.002620938 | 0.996472611 | hypothetical protein |
| CNXL_066170 | CNM01960 | -0.290405003 | 0.613272076 | synaptosomal-associated protein |
| CNXL_066180 | CNM01980 | -0.475499934 | 0.227130642 | hypothetical protein |
| CNXL_066190 | CNM01990 | 0.154340067 | 0.781361786 | nuclear DNA helicase II |
| CNXL_066200 | CNM02000 | 0.827931582 | 0.014903874 | hypothetical protein |
| CNXL_066210 | | -0.493174745 | 0.515753955 | hypothetical protein |
| CNXL_066220 | CNM02020 | -1.675761528 | 1.50849E-11 | Unknown |
| CNXL_066230 | CNM02030 | -0.286406719 | 0.591192608 | hmg |
| CNXL_066240 | CNM02040 | 0.338864692 | 0.325592399 | high-affinity nicotinic acid transporter |
| CNXL_066250 | CNM02050 | 0.012853267 | 0.988641003 | pr4/barwin domain protein |
| CNXL_066260 | CNM02070 | -0.395055352 | 0.314609964 | ATP-dependent RNA helicase DBP9 |
| CNXL_066270 | CNM02100 | -0.039086071 | 0.951685063 | heat shock 70kDa protein 4 |
| CNXL_066280 | | -1.343136464 | 7.45229E-05 | hypothetical protein |
| CNXL_066290 | CNM02110 | 0.114378 | 0.838837582 | hypothetical protein |
| CNXL_066300 | CNM02120 | -0.083117067 | 0.918756814 | protein phosphatase 2 |
| CNXL_066310 | | 0.808014855 | 0.005986128 | hypothetical protein |
| CNXL_066320 | | 0.741338775 | 0.064226952 | hypothetical protein |
| CNXL_066330 | | -0.65321509 | 0.862938503 | Unknown |
| CNXL_066340 | CNM02150 | -1.256675232 | 9.55461E-05 | hypothetical protein |
| CNXL_066350 | CNM02160 | 0.396629273 | 0.332332744 | hypothetical protein |
| CNXL_066360 | CNM02170 | 0.738314973 | 0.08079843 | hypothetical protein |
| CNXL_066370 | CNM02180 | 0.375985775 | 0.239372972 | hypothetical protein |
| CNXL_066380 | CNM02190 | 0.441680128 | 0.213849586 | amidase |
| CNXL_066390 | CNM02200 | 1.680099752 | 0.136935637 | amyloid beta protein binding protein 1 |
| CNXL_066400 | CNM02230 | 0.26294442 | 0.539585872 | allergen |
| CNXL_066410 | CNM02240 | -0.0081214 | 0.991437728 | diphthamide biosynthesis protein 2 |
| CNXL_066420 | CNM02250 | -0.126186884 | 0.83412415 | large subunit ribosomal protein L32e |
| CNXL_066430 | CNM02260 | 0.074920122 | 0.917446543 | E3 SUMO-protein ligase PIAS1 |
| CNXL_066440 | | 0.747689795 | 0.830440343 | nuclear distribution protein NudC homolog |
| CNXL_066450 | CNM02270 | -0.133229086 | 0.802877744 | hypothetical protein |
| CNXL_066460 | CNM02280 | 0.264161028 | 0.744893445 | NADH dehydrogenase |
| CNXL_066470 | | 0.310117519 | 0.648673823 | fanconi-associated nuclease 1 |
| CNXL_066480 | CNM02290 | -0.170795527 | 0.766858347 | Unknown |
| CNXL_066490 | CNM02300 | 0.242815948 | 0.619100215 | MIF4G/MA4 domain-containing protein |
| CNXL_066500 | CNM02310 | -0.960341006 | 0.009881674 | hypothetical protein |
| CNXL_066510 | CNM02320 | -0.250150649 | 0.527018595 | hypothetical protein |
| CNXL_066520 | CNM02330 | 0.968793816 | 0.00199355 | large subunit ribosomal protein L13 |
| CNXL_066530 | CNM02340 | 0.067481269 | 0.964266166 | transcription factor C subunit 7 |
| CNXL_066540 | | 0.019246134 | 0.982102702 | Unknown |
| CNXL_066550 | CNM02360 | 0.588079867 | 0.191629695 | hypothetical protein |
| CNXL_066560 | CNM02370 | 0.48238716 | 0.173210496 | RNA exonuclease NGL2 |
| CNXL_066570 | CNM02380 | 1.756377501 | 5.23669E-05 | YeeE/YedE family protein |
| CNXL_066580 | | 0.406348236 | 0.361880264 | glutathione S-transferase |
| CNXL_066590 | CNM02400 | -0.46791933 | 0.261498496 | Unknown |
| CNXL_066600 | CNM02410 | -0.639507677 | 0.074787003 | hypothetical protein |
| CNXL_066610 | CNM02420 | 0.82463596 | 0.020570133 | protein disulfide-isomerase |
| CNXL_066620 | CNM02430 | 1.047169432 | 0.00080214 | ferroxidase/laccase |
| CNXL_066630 | | 0.573823593 | 0.131030207 | major iron permease |
| CNXL_066640 | CNM02440 | 0.186714067 | 0.805361201 | Unknown |
| CNXL_066650 | CNM02450 | 0.453908114 | 0.205950327 | Unknown |
| CNXL_066660 | CNM02460 | 0.512421963 | 0.08995737 | hypothetical protein |
| CNXL_066670 | CNM02470 | -0.329834986 | 0.353691088 | hypothetical protein |
| CNXL_066680 | CNM02480 | 0.269152284 | 0.633439709 | hypothetical protein |

| | | | | |
|---|---|---|---|---|
| CNXL_066690 | CNM02490 | -0.550244561 | 0.12453995 | hypothetical protein |
| CNXL_066700 | CNM02500 | 0.784160478 | 0.009648212 | protein TIF31 |
| CNXL_066710 | CNM02510 | -0.559493903 | 0.17290643 | taurine catabolism dioxygenase TauD |
| CNXL_066720 | CNM02520 | -0.147022616 | 0.830440343 | hypothetical protein |
| CNXL_066730 | CNM02530 | -0.05787663 | 0.947835086 | ser/Thr protein phosphatase |
| CNXL_066740 | CNM02540 | -0.219950078 | 0.623472961 | hypothetical protein |
| CNXL_066750 | CNM02550 | -0.021838159 | 0.974961329 | sugar transporter |
| CNXL_066760 | CNM02560 | 0.266613884 | 0.491722624 | uracil transporter FurD |
| CNXL_066770 | | 0.170008728 | 0.746347427 | hypothetical protein |
| CNXL_066780 | CNM02570 | 0.077549238 | 0.913786654 | hypothetical protein |
| CNXL_066790 | CNM02580 | -0.21231333 | 0.865815732 | putative hexose transporter |
| CNXL_066800 | CNM02590 | 0.747264741 | 0.052172941 | NAD binding dehydrogenase |
| CNXL_066810 | CNM02600 | 0.612334576 | 0.053835811 | hypothetical protein |
| CNXL_066820 | | NA | NA | hypothetical protein |
| CNXL_066830 | CNN00020 | 0.154516285 | 0.791147058 | nuclear protein |
| CNXL_066840 | CNN00030 | 0.292613302 | 0.534266327 | transcription factor |
| CNXL_066850 | CNN00040 | 0.21016857 | 0.638319466 | high-affinity nicotinic acid transporter |
| CNXL_066860 | CNN00050 | 0.404722852 | 0.375774554 | hypothetical protein |
| CNXL_066870 | | -0.122037552 | 0.934072032 | hypothetical protein |
| CNXL_066880 | | 0.529531206 | 0.490183286 | Unknown |
| CNXL_066890 | CNN00060 | 0.087047493 | 0.894317988 | Unknown |
| CNXL_066900 | CNN00070 | -0.197379223 | 0.699951255 | RNA polymerase II transcription factor |
| CNXL_066910 | CNN00080 | -0.07522972 | 0.90492405 | nucleoprotein TPR |
| CNXL_066920 | | -0.117199985 | 0.914338536 | glycylpeptide N-tetradecanoyltransferase |
| CNXL_066930 | | -1.900840889 | 0.384549545 | hypothetical protein |
| CNXL_066940 | CNN00100 | -0.140490794 | 0.79895415 | Unknown |
| CNXL_066950 | | -0.121097022 | 0.865225322 | hypothetical protein |
| CNXL_066960 | CNN00120 | -0.6808868 | 0.056242298 | Unknown |
| CNXL_066970 | CNN00130 | -0.42118464 | 0.380734973 | ribosomal RNA-processing protein 12 |
| CNXL_066980 | CNN00140 | 0.011973148 | 0.988662258 | hypothetical protein |
| CNXL_066990 | CNN00150 | -0.040902574 | 0.969527869 | alpha-glucosidase |
| CNXL_067000 | | 0.047342135 | 0.95265881 | MFS transporter |
| CNXL_067010 | | 0.201677044 | 0.930994697 | Unknown |
| CNXL_067020 | | 0.243561773 | 0.804701377 | Unknown |
| CNXL_067030 | CNN00160 | -0.139516369 | 0.795111334 | Unknown |
| CNXL_067040 | | -0.658209132 | 0.196906673 | two-component-like sensor kinase |
| CNXL_067050 | CNN00170 | -0.304467385 | 0.503000429 | Unknown |
| CNXL_067060 | | 0.425732688 | 0.232925502 | transportin-3 |
| CNXL_067070 | CNN00180 | 0.277254641 | 0.485459548 | hypothetical protein |
| CNXL_067080 | CNN00190 | 0.479588978 | 0.160684182 | nuclear protein |
| CNXL_067090 | CNN00200 | 0.003955469 | 0.996158838 | WD-repeat protein 48 |
| CNXL_067100 | CNN00210 | -0.167975676 | 0.844943819 | hypothetical protein |
| CNXL_067110 | | 0.621061458 | 0.063742019 | hypothetical protein |
| CNXL_067120 | | 0.606734632 | 0.256808702 | hypothetical protein |
| CNXL_067130 | CNN00220 | 0.292692285 | 0.434256079 | hypothetical protein |
| CNXL_067140 | CNN00230 | 0.145456604 | 0.787846251 | glutathione peroxidase |
| CNXL_067150 | CNN00240 | 0.330010161 | 0.358272717 | RNA binding protein |
| CNXL_067160 | CNN00260 | -0.218117048 | 0.609326629 | hypothetical protein |
| CNXL_067170 | CNN00270 | 0.156646617 | 0.813601278 | high-affinity glucose transporter SNF3 |
| CNXL_067180 | | -0.14349418 | 0.791528992 | polysaccharide deacetylase |
| CNXL_067190 | CNN00290 | 0.245096387 | 0.664771357 | sugar transporter |
| CNXL_067200 | CNN00300 | 0.877463412 | 0.017464684 | hypothetical protein |
| CNXL_067210 | CNN00310 | 0.175221275 | 0.772638959 | hypothetical protein |
| CNXL_067220 | CNN00320 | 0.200430091 | 0.884028948 | cytoplasmic protein |
| CNXL_067230 | CNN00330 | -4.778980815 | 5.701E-73 | hypothetical protein |
| CNXL_067240 | | -4.09622123 | 3.51575E-08 | hypothetical protein |
| CNXL_067250 | CNN00350 | 0.009358742 | 0.991508881 | hypothetical protein |
| CNXL_067260 | CNN00360 | 0.334059129 | 0.40951997 | glutamate-cysteine ligase catalytic subunit |

| | | | | |
|---|---|---|---|---|
| CNXL_067270 | CNN00370 | 1.345603454 | 8.55658E-06 | agc family protein kinase |
| CNXL_067280 | | 0.407765094 | 0.498121256 | hypothetical protein |
| CNXL_067290 | CNN00380 | 0.326613406 | 0.738137683 | Unknown |
| CNXL_067300 | CNN00390 | 0.456961353 | 0.372306093 | Unknown |
| CNXL_067310 | | -2.059195751 | 0.151674873 | WD-repeat protein |
| CNXL_067320 | | -3.3679543 | 0.102152715 | Unknown |
| CNXL_067330 | CNN00400 | -0.735774235 | 0.023995581 | hypothetical protein |
| CNXL_067340 | CNN00410 | -0.379994963 | 0.363294884 | CAMK/CAMKL protein kinase |
| CNXL_067350 | CNN00420 | 0.443546412 | 0.755488326 | cytoplasmic protein |
| CNXL_067360 | CNN00430 | 0.226912266 | 0.575997802 | hypothetical protein |
| CNXL_067370 | CNN00440 | -0.331823371 | 0.565678139 | phosphoglucomutase |
| CNXL_067380 | CNN00450 | 0.076781212 | 0.918783065 | phosphoribosylamine-glycine ligase |
| CNXL_067390 | CNN00460 | 0.047710517 | 0.943193944 | protein CWC15 |
| CNXL_067400 | CNN00470 | 0.480461244 | 0.236004557 | glycine cleavage system H protein |
| CNXL_067410 | | 0.17497554 | 0.912929878 | hypothetical protein |
| CNXL_067420 | CNN00480 | -0.142290105 | 0.825812427 | Unknown |
| CNXL_067430 | CNN00490 | -0.743249572 | 0.010733247 | ribosome biogenesis protein YTM1 |
| CNXL_067440 | | 0.489593642 | 0.420919628 | hypothetical protein |
| CNXL_067450 | CNN00500 | 0.237575794 | 0.678207179 | hypothetical protein |
| CNXL_067460 | CNN00510 | 1.355756218 | 2.31591E-07 | histone acetyltransferase |
| CNXL_067470 | | 0.416461276 | 0.437062442 | L-fucose permease |
| CNXL_067480 | CNN00520 | -1.383643656 | 0.023780306 | Unknown |
| CNXL_067490 | CNN00530 | -0.263488278 | 0.5737945 | zinc finger protein |
| CNXL_067500 | | 0.003412215 | 0.996374193 | GTPase activator |
| CNXL_067510 | | -0.205057667 | 0.688446029 | Unknown |
| CNXL_067520 | | -0.16514557 | 0.874337207 | Unknown |
| CNXL_067530 | CNN00550 | -0.130165954 | 0.81842519 | Unknown |
| CNXL_067540 | CNN00560 | 0.186654104 | 0.796875929 | zinc finger transcription factor |
| CNXL_067550 | CNN00570 | 0.376656957 | 0.495942837 | hypothetical protein |
| CNXL_067560 | CNN00580 | 0.331274587 | 0.55049819 | high-affinity nicotinic acid transporter |
| CNXL_067570 | | 0.368886767 | 0.509453385 | hypothetical protein |
| CNXL_067580 | CNN00610 | -0.196901748 | 0.701325477 | hypothetical protein |
| CNXL_067590 | CNN00620 | -0.584743421 | 0.683642142 | hypothetical protein |
| CNXL_067600 | CNN00630 | 0.246627246 | 0.645032535 | Unknown |
| CNXL_067610 | CNN00640 | 0.906190774 | 0.00111032 | replication factor C subunit 3/5 |
| CNXL_067620 | CNN00650 | -0.794046564 | 0.2430565 | hypothetical protein |
| CNXL_067630 | CNN00660 | 0.070986381 | 0.927441884 | centromeric protein E |
| CNXL_067640 | CNN00670 | -2.317342776 | 1.05491E-20 | glucan 1 |
| CNXL_067650 | CNN00680 | 0.010166734 | 0.991141666 | hypothetical protein |
| CNXL_067660 | CNN00690 | 0.408378394 | 0.25037723 | ABC transporter PMR5 |
| CNXL_067670 | CNN00700 | -0.067814679 | 0.918375149 | hypothetical protein |
| CNXL_067680 | CNN00710 | -0.137310491 | 0.856046614 | pre-rRNA-processing protein TSR4 |
| CNXL_067690 | CNN00720 | 0.060017448 | 0.94759673 | hypothetical protein |
| CNXL_067700 | CNN00730 | -0.22950863 | 0.691786101 | SUMO activating enzyme |
| CNXL_067710 | | 0.463968759 | 0.249362477 | hypothetical protein |
| CNXL_067720 | CNN00750 | -0.039881391 | 0.957559333 | hypothetical protein |
| CNXL_067730 | | -1.242100674 | 0.582702964 | mitochondrial distribution and morphology protein 10 |
| CNXL_067740 | CNN00760 | 0.77034794 | 0.007271666 | barwin-like protein domain |
| CNXL_067750 | | -0.43671108 | 0.39417374 | pr4/barwin domain protein |
| CNXL_067760 | CNN00770 | -0.582895574 | 0.293624485 | Unknown |
| CNXL_067770 | CNN00780 | -1.020937891 | 0.255634549 | ATP-binding cassette transporter |
| CNXL_067780 | CNN00790 | -0.138157958 | 0.857201424 | hypothetical protein |
| CNXL_067790 | CNN00800 | -0.062739749 | 0.92070335 | hypothetical protein |
| CNXL_067800 | CNN00810 | -0.08311394 | 0.910897541 | ER-protein sec61-beta ortholog |
| CNXL_067810 | CNN00820 | -0.020841521 | 0.980610712 | hypothetical protein |
| CNXL_067820 | | -1.098062685 | 0.056873107 | tRNA pseudouridine |
| CNXL_067830 | | 0.574248432 | 0.039924281 | hypothetical protein |
| CNXL_067840 | CNN00830 | 0.269625672 | 0.53200052 | hypothetical protein |

| | | | | |
|---|---|---|---|---|
| CNXL_067850 | CNN00840 | 0.172713619 | 0.781361786 | mitochondrial protein |
| CNXL_067860 | | 0.346684818 | 0.534018645 | hypothetical protein |
| CNXL_067870 | CNN00850 | 0.15456123 | 0.811758113 | hypothetical protein |
| CNXL_067880 | CNN00860 | 0.2023438 | 0.653437792 | scavenger mRNA-decapping enzyme DcpS |
| CNXL_067890 | CNN00870 | 0.048307005 | 0.948286148 | alcohol dehydrogenase |
| CNXL_067900 | CNN00890 | 0.065342698 | 0.925523772 | 26S proteasome regulatory subunit N6 |
| CNXL_067910 | CNN00910 | -0.325298151 | 0.435082889 | hypothetical protein |
| CNXL_067920 | CNN00920 | 0.148059922 | 0.770005211 | nucleolar protein |
| CNXL_067930 | CNN00930 | 0.006613957 | 0.994325965 | hypothetical protein |
| CNXL_067940 | CNN00940 | -1.213796808 | 0.000422279 | derlin-2/3 |
| CNXL_067950 | CNN00950 | -0.367782361 | 0.415823204 | protein kinase |
| CNXL_067960 | CNN00960 | 0.204210938 | 0.725038374 | U3 small nucleolar RNA-associated protein 3 |
| CNXL_067970 | CNN00970 | 0.460036461 | 0.255491756 | hypothetical protein |
| CNXL_067980 | CNN00980 | -0.10001952 | 0.892724552 | geranylgeranyl transferase type-2 subunit alpha |
| CNXL_067990 | CNN00990 | -0.938623145 | 0.008540515 | branched-chain-amino-acid aminotransferase |
| CNXL_068000 | CNN01000 | 1.027575865 | 0.000286013 | guanine deaminase |
| CNXL_068010 | | -0.216341267 | 0.706017002 | Unknown |
| CNXL_068020 | CNN01010 | -1.131199648 | 1.85504E-05 | mitotic spindle assembly checkpoint protein MAD2B |
| CNXL_068030 | | 0.030287195 | 0.974961329 | malate dehydrogenase |
| CNXL_068040 | CNN01020 | 0.086967001 | 0.897988379 | hypothetical protein |
| CNXL_068050 | CNN01030 | 0.176182602 | 0.706539593 | vacuolar membrane protein |
| CNXL_068060 | CNN01040 | 0.318657025 | 0.572926427 | solute carrier family 25 |
| CNXL_068070 | CNN01050 | -0.58996399 | 0.144421956 | hypothetical protein |
| CNXL_068080 | CNN01060 | 0.185895692 | 0.712281674 | N-acetyltransferase 10 |
| CNXL_068090 | CNN01070 | 0.368516266 | 0.510006134 | transcription initiation factor TFIIA large subunit |
| CNXL_068100 | CNN01080 | -0.001749352 | 0.997459233 | hypothetical protein |
| CNXL_068110 | CNN01090 | -0.331125939 | 0.519207377 | beta-transducin repeat containing protein |
| CNXL_068120 | CNN01100 | 0.374697924 | 0.345494206 | cytoplasmic protein |
| CNXL_068130 | CNN01120 | 0.102136381 | 0.866119159 | DNA repair protein RAD50 |
| CNXL_068140 | CNN01130 | -0.273775313 | 0.634625988 | Unknown |
| CNXL_068150 | CNN01140 | 0.029242204 | 0.969321276 | hypothetical protein |
| CNXL_068160 | CNN01150 | -4.772615779 | 1.1494E-104 | Cullin 1 |
| CNXL_068170 | CNN01170 | -0.727670657 | 0.026795356 | hypothetical protein |
| CNXL_068180 | | -0.153437433 | 0.869733568 | hypothetical protein |
| CNXL_068190 | | -0.869545504 | 0.560022419 | Unknown |
| CNXL_068200 | CNN01180 | 0.73582945 | 0.033774242 | hypothetical protein |
| CNXL_068210 | CNN01190 | 0.580014835 | 0.183989153 | hypothetical protein |
| CNXL_068220 | | 0.18997377 | 0.809612358 | transcription initiation factor TFIIH subunit 4 |
| CNXL_068230 | CNN01210 | 1.955192682 | 0.019055949 | hypothetical protein |
| CNXL_068240 | CNN01220 | 0.360340119 | 0.313087832 | hypothetical protein |
| CNXL_068250 | CNN01250 | 0.783290384 | 0.00566394 | hypothetical protein |
| CNXL_068260 | CNN01260 | -0.680442765 | 0.040188422 | SH3 domain-containing protein |
| CNXL_068270 | CNN01270 | -2.04897272 | 1.06262E-09 | plasma membrane H |
| CNXL_068280 | | -1.053642193 | 0.003448728 | hypothetical protein |
| CNXL_068290 | CNN01280 | 0.111037005 | 0.855058004 | Unknown |
| CNXL_068300 | | 0.340220421 | 0.663156438 | 26S proteasome complex subunit DSS1 |
| CNXL_068310 | CNN01290 | -0.127001697 | 0.833643586 | Unknown |
| CNXL_068320 | CNN01300 | -0.530416213 | 0.063800281 | hypothetical protein |
| CNXL_068330 | CNN01310 | -0.062862035 | 0.942821233 | hypothetical protein |
| CNXL_068340 | CNN01320 | -0.065348357 | 0.944879244 | hypothetical protein |
| CNXL_068350 | CNN01330 | -0.393847702 | 0.38937973 | hypothetical protein |
| CNXL_068360 | | 0.104490698 | 0.920669589 | cytochrome c oxidase subunit 7c |
| CNXL_068370 | | 0.309166168 | 0.779355929 | Unknown |
| CNXL_068380 | | 0.67504106 | 0.040706658 | Unknown |
| CNXL_068390 | CNN01360 | -1.787722612 | 1.82809E-16 | hypothetical protein |
| CNXL_068400 | CNN01370 | 0.113261941 | 0.881190377 | mutanase |
| CNXL_068410 | CNN01390 | -0.324229497 | 0.521243755 | rRNA-processing protein EFG1 |
| CNXL_068420 | CNN01400 | 0.200510368 | 0.678124378 | cytochrome c oxidase-assembly factor COX23 |

| | | | | |
|---|---|---|---|---|
| CNXL_068430 | CNN01410 | -0.214572176 | 0.710014173 | Cu |
| CNXL_068440 | CNN01420 | 0.029597951 | 0.970404228 | putative dipeptidyl aminopeptidase |
| CNXL_068450 | CNN01430 | 0.020340576 | 0.985173026 | AP-2 complex subunit sigma |
| CNXL_068460 | CNN01440 | 0.346731662 | 0.425693559 | PP2Cc protein phosphatase |
| CNXL_068470 | CNN01450 | 0.071257124 | 0.919952257 | hypothetical protein |
| CNXL_068480 | CNN01460 | -0.077231847 | 0.927504878 | solute carrier family 25 |
| CNXL_068490 | CNN01470 | -0.949080069 | 0.001380777 | acetolactate synthase |
| CNXL_068500 | CNN01480 | -0.092554012 | 0.919822815 | hypothetical protein |
| CNXL_068510 | | -0.583565204 | 0.094990157 | hypothetical protein |
| CNXL_068520 | CNN01490 | 0.100644601 | 0.903834486 | claudin family protein |
| CNXL_068530 | CNN01500 | 0.039324153 | 0.965004754 | fungal specific transcription factor |
| CNXL_068540 | CNN01510 | 0.298142724 | 0.510006134 | U6 snRNA-associated Sm-like protein LSm6 |
| CNXL_068550 | CNN01520 | 0.665934018 | 0.057822411 | hypothetical protein |
| CNXL_068560 | CNN01530 | -0.359049231 | 0.402210452 | ubiquitin-specific protease |
| CNXL_068570 | CNN01540 | -0.116374115 | 0.878054651 | O-acetyltransferase |
| CNXL_068580 | CNN01550 | 0.4251721 | 0.203097519 | hypothetical protein |
| CNXL_068590 | CNN01560 | 0.617207184 | 0.063742019 | acyl-CoA oxidase |
| CNXL_068600 | CNN01570 | -0.102331627 | 0.861625385 | acetate kinase |
| CNXL_068610 | | 2.266055238 | 0.061660269 | AMP-binding protein |
| CNXL_068620 | CNN01600 | 0.256525998 | 0.626087429 | hypothetical protein |
| CNXL_068630 | CNN01610 | -0.179677014 | 0.771343811 | regulatory subunit for Cdc7 protein kinase |
| CNXL_068640 | CNN01620 | 0.458458081 | 0.434256079 | hypothetical protein |
| CNXL_068650 | CNN01630 | 0.266428064 | 0.560961964 | hypothetical protein |
| CNXL_068660 | | -0.325338234 | 0.616555404 | hypothetical protein |
| CNXL_068670 | CNN01640 | -0.071577658 | 0.920854816 | hypothetical protein |
| CNXL_068680 | CNN01650 | -0.027427689 | 0.974020242 | d-arabinono-1 |
| CNXL_068690 | CNN01660 | 0.455313043 | 0.249559817 | DNA dependent ATPase |
| CNXL_068700 | CNN01680 | -0.685432119 | 0.011831672 | isoleucine-tRNA ligase |
| CNXL_068710 | NN01690\|CNN017( | 0.050238467 | 0.936086163 | hypothetical protein |
| CNXL_068720 | CNN01710 | -0.215298468 | 0.746537956 | Unknown |
| CNXL_068730 | CNN01720 | -0.267278493 | 0.49772658 | mitochondrial splicing suppressor |
| CNXL_068740 | CNN01730 | 0.155875975 | 0.767040572 | large subunit ribosomal protein L22 |
| CNXL_068750 | CNN01740 | -1.747242074 | 1.0055E-11 | cystathionine gamma-lyase |
| CNXL_068760 | CNN01750 | -0.603735518 | 0.03771244 | hypothetical protein |
| CNXL_068770 | | 0.287470207 | 0.527938546 | hypothetical protein |
| CNXL_068780 | CNN01760 | 0.519845844 | 0.196372554 | hypothetical protein |
| CNXL_068790 | CNN01770 | 1.24873194 | 0.000112224 | integral membrane protein |
| CNXL_068800 | CNN01780 | 0.15892726 | 0.802313068 | benzodiazapine receptor |
| CNXL_068810 | CNN01790 | -0.758152781 | 0.068127935 | Atypical/RIO/RIO1 protein kinase |
| CNXL_068820 | CNN01810 | -0.069336089 | 0.912929878 | hypothetical protein |
| CNXL_068830 | CNN01820 | 0.364338103 | 0.325688377 | hypothetical protein |
| CNXL_068840 | CNN01830 | 0.003902517 | 0.995928861 | vacuolar membrane protein |
| CNXL_068850 | CNN01840 | -0.840057101 | 0.010233234 | hypothetical protein |
| CNXL_068860 | CNN01850 | 0.059729926 | 0.942099307 | cell division control protein 25 |
| CNXL_068870 | CNN01860 | 0.132687945 | 0.827557138 | Golgi factor and virulence related protein |
| CNXL_068880 | CNN01870 | 0.038140369 | 0.970515325 | transcription regulator |
| CNXL_068890 | | -0.228676107 | 0.64162027 | jumonji domain containing 5 |
| CNXL_068900 | CNN01880 | -0.167155699 | 0.77767171 | hypothetical protein |
| CNXL_068910 | CNN01890 | -0.468755518 | 0.210709251 | nucleolar GTP-binding protein |
| CNXL_068920 | CNN01900 | -0.104554711 | 0.903247343 | aminophospholipid translocase |
| CNXL_068930 | CNN01910 | -0.100310284 | 0.87678962 | hypothetical protein |
| CNXL_068940 | CNN01920 | -0.513658017 | 0.239372972 | small subunit ribosomal protein S12 |
| CNXL_068950 | CNN01930 | -0.707197415 | 0.039924281 | nucleolar protein 4 |
| CNXL_068960 | CNN01940 | 0.024824224 | 0.970876289 | hypothetical protein |
| CNXL_068970 | CNN01950 | -0.335735893 | 0.488456653 | RNA-binding protein Musashi |
| CNXL_068980 | CNN01960 | 0.159681859 | 0.794943165 | hypothetical protein |
| CNXL_068990 | | -0.243243 | 0.9282276 | Unknown |
| CNXL_069000 | | -0.866396614 | 0.753324699 | Unknown |

| | | | | |
|---|---|---|---|---|
| CNXL_069010 | | 3.471121089 | NA | Unknown |
| CNXL_069020 | | -1.000405275 | 0.667378962 | hypothetical protein |
| CNXL_069030 | | -0.011201196 | NA | Unknown |
| CNXL_069040 | | NA | NA | Unknown |
| CNXL_069050 | | NA | NA | Unknown |
| CNXL_069060 | | NA | NA | Unknown |
| CNXL_069070 | | NA | NA | Unknown |
| CNXL_069080 | | NA | NA | Unknown |
| CNXL_069090 | | NA | NA | Unknown |
| CNXL_069100 | | NA | NA | Unknown |
| CNXL_069110 | CNE03000 | 0 | NA | Unknown |
| CNXL_069120 | | NA | NA | Unknown |
| CNXL_069130 | | NA | NA | Unknown |
| CNXL_069140 | | 0.382016958 | 0.940682674 | Unknown |
| CNXL_069150 | CNN02040 | 0 | NA | Unknown |
| CNXL_069160 | | NA | NA | Unknown |
| CNXL_069170 | CNN02060 | -0.088553222 | 0.910379167 | Unknown |
| CNXL_069180 | CNN02070 | -0.012807888 | 0.990777629 | amidase |
| CNXL_069190 | CNN02080 | -0.214519046 | 0.770559628 | hypothetical protein |
| CNXL_069200 | CNN02090 | 0.230847424 | 0.701325477 | hypothetical protein |
| CNXL_069210 | CNN02100 | 0.060987469 | 0.926035744 | hexose transporter protein |
| CNXL_069220 | CNN02110 | -0.195035739 | 0.779355929 | hypothetical protein |
| CNXL_069230 | CNN02120 | -0.155282157 | 0.852156463 | putative chitin synthase |
| CNXL_069240 | CNN02130 | -0.580543874 | 0.167895669 | hypothetical protein |
| CNXL_069250 | CNN02140 | -0.466647846 | 0.299740017 | adenosine kinase |
| CNXL_069260 | CNN02150 | -0.375602353 | 0.66424166 | CAMK/CAMKL protein kinase |
| CNXL_069270 | CNN02160 | -0.189692096 | 0.785592376 | hypothetical protein |
| CNXL_069280 | CNN02170 | -0.503395329 | 0.175271548 | cytoplasmic protein |
| CNXL_069290 | CNN02180 | 0.720410915 | 0.098879563 | Unknown |
| CNXL_069300 | CNN02190 | 0.779500434 | 0.015173028 | hypothetical protein |
| CNXL_069310 | | -0.230454681 | 0.782518595 | cold-induced thioredoxin domain-containing protein |
| CNXL_069320 | CNN02200 | -0.05622192 | 0.93471488 | Unknown |
| CNXL_069330 | CNN02210 | -0.339653879 | 0.534266327 | RNA exonuclease 1 |
| CNXL_069340 | CNN02220 | 1.031632646 | 0.005077052 | Unknown |
| CNXL_069350 | CNN02230 | -0.03842283 | 0.954346886 | microsomal epoxide hydrolase |
| CNXL_069360 | CNN02240 | -0.299902718 | 0.643890318 | hypothetical protein |
| CNXL_069370 | | -0.53236871 | 0.43363696 | hypothetical protein |
| CNXL_069380 | CNN02250 | -0.200426607 | 0.791528992 | Unknown |
| CNXL_069390 | CNN02260 | -0.188091566 | 0.75342527 | arginine metabolism transcriptional control protein |
| CNXL_069400 | CNN02270 | 0.02164591 | 0.980972278 | 1 |
| CNXL_069410 | CNN02280 | 0.329859174 | 0.393252659 | solute carrier family 25 |
| CNXL_069420 | CNN02290 | -0.048894347 | 0.948286148 | uridine permease |
| CNXL_069430 | CNN02300 | -0.097184382 | 0.87678601 | hypothetical protein |
| CNXL_069440 | CNN02310 | 0.320831774 | 0.42639861 | hypothetical protein |
| CNXL_069450 | | 1.106067518 | 0.048447072 | hypothetical protein |
| CNXL_069460 | CNN02320 | 0.100059005 | 0.865225322 | Unknown |
| CNXL_069470 | CNN02330 | -1.246062662 | 0.057424997 | 1 |
| CNXL_069480 | CNN02340 | -0.113024672 | 0.889001755 | hypothetical protein |
| CNXL_069490 | | 1.086540084 | 0.000594297 | anaphase-promoting complex subunit 2 |
| CNXL_069500 | CNN02370 | 0.585576889 | 0.071401102 | hypothetical protein |
| CNXL_069510 | | 1.791013689 | 3.03543E-09 | pirin |
| CNXL_069520 | | 1.788955423 | 6.47153E-08 | Unknown |
| CNXL_069530 | CNN02390 | -0.706792891 | 0.018967878 | Unknown |
| CNXL_069540 | CNN02400 | 0.538443394 | 0.069712502 | hypothetical protein |
| CNXL_069550 | CNN02410 | -0.358586856 | 0.434588143 | cytoplasmic protein |
| CNXL_069560 | | -0.514638769 | 0.406556386 | autophagy protein 5 |
| CNXL_069570 | | -0.241571405 | 0.806051382 | hypothetical protein |
| CNXL_069580 | | 0.336086717 | 0.53679353 | Unknown |

| | | | | |
|---|---|---|---|---|
| CNXL_069590 | CNN02420 | 0.46341876 | 0.24509521 | Unknown |
| CNXL_069600 | | -0.175218052 | 0.906286943 | hypothetical protein |
| CNXL_069610 | | 0.844181767 | 0.067063478 | Unknown |
| CNXL_069620 | | 0.235790911 | 0.749315941 | Unknown |
| CNXL_069630 | | 0.450392543 | 0.208087633 | hypothetical protein |
| CNXL_069640 | NE00100\|CNN024( | -0.552684865 | 0.802348605 | Unknown |
| CNXL_100010 | | -0.595453786 | 0.66424166 | Unknown |
| CNXL_100020 | | 0.446836922 | 0.529627838 | Unknown |
| CNXL_100030 | | -0.065108245 | 0.947835086 | Unknown |
| CNXL_100040 | | -1.901477791 | 0.471465563 | Unknown |
| CNXL_100050 | | -1.114962269 | 0.222448802 | Unknown |
| CNXL_100060 | | -1.896202063 | 0.010405914 | Unknown |
| CNXL_100070 | | 0.907926789 | 0.190652363 | Unknown |
| CNXL_100080 | | 0.870963277 | 0.157787273 | Unknown |
| CNXL_100090 | | 0.380048331 | 0.520532844 | Unknown |
| CNXL_100100 | | -0.191746839 | 0.934642219 | Unknown |
| CNXL_100110 | | -0.093016085 | 0.953095191 | Unknown |
| CNXL_100120 | | -0.235838142 | 0.837479784 | Unknown |
| CNXL_100130 | | 0.752925258 | 0.794943165 | Unknown |
| CNXL_100140 | | 1.57751656 | 0.451973326 | Unknown |
| CNXL_100150 | | -0.633305392 | 0.614941803 | Unknown |
| CNXL_100160 | | 1.045327819 | NA | Unknown |
| CNXL_100170 | | 0.888324448 | 0.609523424 | Unknown |
| CNXL_100180 | | 2.063195709 | 0.021162673 | Unknown |
| CNXL_100190 | | 0.541171438 | 0.61372863 | Unknown |
| CNXL_100200 | | -0.713517533 | 0.825693641 | Unknown |
| CNXL_100210 | | -0.372350334 | 0.81842519 | Unknown |
| CNXL_100220 | | -0.905432631 | 0.634012464 | Unknown |
| CNXL_100230 | | -2.355814862 | 9.84115E-12 | Unknown |
| CNXL_100240 | | 0.673727653 | 0.064066517 | Unknown |
| CNXL_100250 | | -1.142148989 | 0.714943724 | Unknown |
| CNXL_100260 | | -0.382946152 | 0.661510467 | Unknown |
| CNXL_100270 | | -0.433920215 | 0.703985737 | Unknown |
| CNXL_100280 | | 0.046918936 | 0.956370213 | Unknown |
| CNXL_100290 | | 0.902794684 | 0.069009964 | Unknown |
| CNXL_100300 | | 0.320440499 | 0.880063236 | Unknown |
| CNXL_100310 | | 0.070945169 | 0.953095191 | Unknown |
| CNXL_100320 | | 0.459220632 | 0.610094871 | Unknown |
| CNXL_100330 | | 0.448306897 | 0.825693641 | Unknown |
| CNXL_100340 | | 0.222836019 | 0.746537956 | Unknown |
| CNXL_100350 | | -0.540973608 | 0.879688103 | Unknown |
| CNXL_100360 | | -0.577259995 | 0.217998625 | Unknown |
| CNXL_100370 | | -1.053556474 | 0.490579054 | Unknown |
| CNXL_100380 | | -0.124187322 | 0.920704559 | Unknown |
| CNXL_100390 | | 0.59075207 | 0.523358959 | Unknown |
| CNXL_100400 | | 0.057904921 | 0.953095191 | Unknown |
| CNXL_100410 | | 1.27190264 | 0.444166175 | Unknown |
| CNXL_100420 | | -0.004894533 | 0.996472611 | Unknown |
| CNXL_100430 | | -0.175055664 | 0.850005462 | Unknown |
| CNXL_100440 | | -0.343228548 | 0.903834486 | Unknown |
| CNXL_100450 | | 1.41635458 | 0.191477648 | Unknown |
| CNXL_100460 | | 0.423798568 | 0.835748414 | Unknown |
| CNXL_100470 | | -0.194889945 | 0.911130835 | Unknown |
| CNXL_100480 | | 0.09832209 | 0.939400455 | Unknown |
| CNXL_100490 | | 0.520386779 | 0.462504391 | Unknown |
| CNXL_100500 | | 0.170870985 | 0.948286148 | Unknown |
| CNXL_100510 | | 0.251091526 | 0.93451582 | Unknown |
| CNXL_100520 | | 0.749192788 | 0.043973756 | Unknown |

| | | | |
|---|---|---|---|
| CNXL_100530 | 0.588217104 | 0.095579265 | Unknown |
| CNXL_100540 | 0.591966145 | 0.437056582 | Unknown |
| CNXL_100550 | -1.059504336 | 0.194911786 | Unknown |
| CNXL_100560 | -0.129954482 | 0.828216915 | Unknown |
| CNXL_100570 | NA | NA | Unknown |
| CNXL_100580 | 0.385239769 | 0.911130835 | Unknown |
| CNXL_100590 | 0.314026349 | 0.747332879 | Unknown |
| CNXL_100600 | 0.744152609 | 0.317388446 | Unknown |
| CNXL_100610 | -0.754189107 | 0.575360741 | Unknown |
| CNXL_100620 | 0.322029066 | 0.703818084 | Unknown |
| CNXL_100630 | -0.216387021 | 0.940348465 | Unknown |
| CNXL_100640 | 0.367264538 | 0.799344206 | Unknown |
| CNXL_100650 | 0.047113983 | 0.9653396 | Unknown |
| CNXL_100660 | 0.435739578 | 0.709598105 | Unknown |
| CNXL_100670 | 0.433459028 | 0.320923413 | Unknown |
| CNXL_100680 | -0.0151642 | NA | Unknown |
| CNXL_100690 | 0.840433894 | 0.015000562 | Unknown |
| CNXL_100700 | 1.490924927 | 1.9828E-05 | Unknown |
| CNXL_100710 | 0.518631194 | 0.730212038 | Unknown |
| CNXL_100720 | 0.095671528 | 0.985173026 | Unknown |
| CNXL_100730 | 0.235946167 | 0.933786064 | Unknown |
| CNXL_100740 | 0.753776156 | 0.191587554 | Unknown |
| CNXL_100750 | -1.83420033 | 0.415823204 | Unknown |
| CNXL_100760 | -0.048356281 | 0.976006716 | Unknown |
| CNXL_100770 | -0.674962848 | 0.496574337 | Unknown |
| CNXL_100780 | -0.575334597 | 0.776681674 | Unknown |
| CNXL_100790 | 0.036970199 | 0.97242592 | Unknown |
| CNXL_100800 | 0.204331937 | 0.838712696 | Unknown |
| CNXL_100810 | -0.523852723 | 0.884028948 | Unknown |
| CNXL_100820 | 0.066724353 | 0.987336046 | Unknown |
| CNXL_100830 | -0.134430566 | 0.967119205 | Unknown |
| CNXL_100840 | -0.032410163 | 0.964414864 | Unknown |
| CNXL_100850 | -0.031997695 | 0.995735232 | Unknown |
| CNXL_100860 | 8.228638982 | 1.14373E-46 | Unknown |
| CNXL_100870 | 0.820460865 | 0.227624637 | Unknown |
| CNXL_100880 | 2.090650363 | 1.41696E-05 | Unknown |
| CNXL_100890 | -0.618825246 | NA | Unknown |
| CNXL_100900 | -0.38196985 | 0.806051382 | Unknown |
| CNXL_100910 | 0.647079122 | 0.036770326 | Unknown |
| CNXL_100920 | 0.013214198 | 0.991133859 | Unknown |
| CNXL_100930 | 0.244411716 | 0.944879244 | Unknown |
| CNXL_100940 | 0.341138641 | 0.907031057 | Unknown |
| CNXL_100950 | 0.733474123 | 0.249969817 | Unknown |
| CNXL_100960 | -0.132096426 | 0.929435738 | Unknown |
| CNXL_100970 | -0.283509952 | 0.753324699 | Unknown |
| CNXL_100980 | -1.644945403 | 0.526368704 | Unknown |
| CNXL_100990 | -0.411274679 | 0.856469734 | Unknown |
| CNXL_101000 | 0.034665737 | 0.992176872 | Unknown |
| CNXL_101010 | 0.508645899 | 0.38882813 | Unknown |
| CNXL_101020 | 0.800164565 | 0.103195469 | Unknown |
| CNXL_101030 | -0.273716024 | 0.799344206 | Unknown |
| CNXL_101040 | -0.451312645 | 0.814220979 | Unknown |
| CNXL_101050 | -0.03944738 | 0.9653396 | Unknown |
| CNXL_101060 | -0.906585577 | 0.616439959 | Unknown |
| CNXL_101070 | -0.673501634 | 0.513365432 | Unknown |
| CNXL_101080 | 1.153585399 | 4.6492E-05 | Unknown |
| CNXL_101090 | -0.180652851 | 0.938291734 | Unknown |
| CNXL_101100 | 0.70864049 | 0.04517704 | Unknown |

| | | | |
|---|---|---|---|
| CNXL_101110 | -0.02144247 | 0.991542997 | Unknown |
| CNXL_101120 | -0.296383133 | 0.761025301 | Unknown |
| CNXL_101130 | 0.125674084 | 0.940348465 | Unknown |
| CNXL_101140 | 0.74504444 | 0.836995157 | Unknown |
| CNXL_101150 | -1.512926715 | 0.638356877 | Unknown |
| CNXL_101160 | 1.265836412 | 0.313918019 | Unknown |
| CNXL_101170 | -1.410275457 | NA | Unknown |
| CNXL_101180 | 0.160753709 | 0.919196787 | Unknown |
| CNXL_101190 | -0.705249756 | 0.209649074 | Unknown |
| CNXL_101200 | -0.830061685 | 0.405051915 | Unknown |
| CNXL_101210 | 0.209845581 | 0.886997541 | Unknown |
| CNXL_101220 | -0.130420034 | 0.890150525 | Unknown |
| CNXL_101230 | 1.36945441 | 0.13126328 | Unknown |
| CNXL_101240 | 0.556464206 | 0.452146814 | Unknown |
| CNXL_101250 | 0.779597836 | 0.259923342 | Unknown |
| CNXL_101260 | -1.403264241 | 0.098322722 | Unknown |
| CNXL_101270 | -1.233672907 | 0.764330585 | Unknown |
| CNXL_101280 | 3.87132284 | NA | Unknown |
| CNXL_101290 | 0.285896125 | 0.653396602 | Unknown |
| CNXL_101300 | -0.573789545 | 0.432824693 | Unknown |
| CNXL_101310 | -0.011201196 | NA | Unknown |
| CNXL_101320 | -0.596303222 | 0.823319274 | Unknown |
| CNXL_101330 | 0.054884839 | 0.972638293 | Unknown |
| CNXL_101340 | -4.2784827 | 0.000571402 | Unknown |
| CNXL_101350 | 0.377458243 | 0.536144787 | Unknown |
| CNXL_101360 | -2.192157905 | NA | Unknown |
| CNXL_101370 | -0.315248572 | 0.647485945 | Unknown |
| CNXL_101380 | -0.035279575 | 0.987082749 | Unknown |
| CNXL_101390 | -0.33654072 | 0.791311641 | Unknown |
| CNXL_101400 | 0.408724718 | 0.566713427 | Unknown |
| CNXL_101410 | -0.792135462 | 0.305504958 | Unknown |
| CNXL_101420 | 0.027537053 | 0.988662258 | Unknown |
| CNXL_101430 | -3.349742098 | 6.83541E-08 | Unknown |
| CNXL_101440 | -1.618959325 | NA | Unknown |
| CNXL_101450 | -0.163282867 | 0.949084374 | Unknown |
| CNXL_101460 | 0.765191784 | 0.030011523 | Unknown |
| CNXL_101470 | -0.275813219 | 0.842469317 | Unknown |
| CNXL_101480 | 1.003479637 | 0.636752734 | Unknown |
| CNXL_101490 | 0.446168981 | 0.317665528 | Unknown |
| CNXL_101500 | 0.321389164 | 0.865614892 | Unknown |
| CNXL_101510 | -0.023274474 | 0.992176872 | Unknown |
| CNXL_101520 | 0.674859674 | 0.724414078 | Unknown |
| CNXL_101530 | 1.658188062 | 5.06508E-11 | Unknown |
| CNXL_101540 | 1.049491252 | 0.396088679 | Unknown |
| CNXL_101550 | -3.27869864 | 0.321737211 | Unknown |
| CNXL_101560 | 0.470853096 | 0.55313399 | Unknown |
| CNXL_101570 | 0.335254478 | 0.811758113 | Unknown |
| CNXL_101580 | -2.400013879 | 5.94112E-09 | Unknown |
| CNXL_101590 | -0.829532145 | 0.651830672 | Unknown |
| CNXL_101600 | -0.139306368 | 0.923938002 | Unknown |
| CNXL_101610 | -0.073180217 | 0.960915029 | Unknown |
| CNXL_101620 | 1.003086096 | 0.017968291 | Unknown |
| CNXL_101630 | -3.653017084 | 5.06892E-18 | Unknown |
| CNXL_101640 | 0.038234709 | 0.981733632 | Unknown |
| CNXL_101650 | 0.640922859 | 0.681547537 | Unknown |
| CNXL_101660 | -0.118471249 | 0.953095191 | Unknown |
| CNXL_101670 | -0.807583573 | 0.495174856 | Unknown |
| CNXL_101680 | -2.364417376 | NA | Unknown |

| | | | |
|---|---|---|---|
| CNXL_101690 | 0.481933344 | 0.59620949 | Unknown |
| CNXL_101700 | 0.273600383 | 0.77424887 | Unknown |
| CNXL_101710 | 0.935848355 | 0.021073663 | Unknown |
| CNXL_101720 | 0.826044529 | 0.366888453 | Unknown |
| CNXL_101730 | 0.071149525 | 0.932840267 | Unknown |
| CNXL_101740 | -0.263109127 | 0.847923235 | Unknown |
| CNXL_101750 | -0.222479965 | 0.885370637 | Unknown |
| CNXL_101760 | -3.771168552 | 0.1830813 | Unknown |
| CNXL_101770 | 0.795254971 | 0.43363696 | Unknown |
| CNXL_101780 | -1.24516604 | NA | Unknown |
| CNXL_101790 | 0.523140056 | 0.651485008 | Unknown |
| CNXL_101800 | -0.5136189 | NA | Unknown |
| CNXL_101810 | -0.280956698 | 0.659118995 | Unknown |
| CNXL_101820 | 0.094521046 | 0.948286148 | Unknown |
| CNXL_101830 | 0.045919193 | 0.955679401 | Unknown |
| CNXL_101840 | 0.668484228 | 0.762431138 | Unknown |
| CNXL_101850 | 0.272692358 | 0.658531157 | Unknown |
| CNXL_101860 | 0.406862947 | 0.547814913 | Unknown |
| CNXL_101870 | 1.255026934 | NA | Unknown |
| CNXL_101880 | -0.356384381 | 0.911754274 | Unknown |
| CNXL_101890 | 0.117417938 | 0.902229878 | Unknown |
| CNXL_101900 | 0.377642753 | 0.857811508 | Unknown |
| CNXL_101910 | -1.330752087 | NA | Unknown |
| CNXL_101920 | 0.823424628 | 0.154758808 | Unknown |
| CNXL_101930 | 0.800267464 | 0.734647431 | Unknown |
| CNXL_101940 | -0.25939791 | 0.789136869 | Unknown |
| CNXL_101950 | 0.234924304 | 0.828543224 | Unknown |
| CNXL_101960 | 0.10343769 | 0.901676457 | Unknown |
| CNXL_101970 | -0.20335688 | 0.704832822 | Unknown |
| CNXL_101980 | 1.070584197 | 0.003509125 | Unknown |
| CNXL_101990 | 0.394092004 | 0.7013463 | Unknown |
| CNXL_102000 | 1.999829853 | NA | Unknown |
| CNXL_102010 | 1.844566739 | 0.519073971 | Unknown |
| CNXL_102020 | 0.132508871 | 0.894629654 | Unknown |
| CNXL_102030 | -0.633456487 | 0.734939207 | Unknown |
| CNXL_102040 | -1.625673497 | 0.277600586 | Unknown |
| CNXL_102050 | 0.404075411 | 0.648500229 | Unknown |
| CNXL_102060 | -0.529887231 | 0.312160498 | Unknown |
| CNXL_102070 | 0.205495669 | 0.913786654 | Unknown |
| CNXL_102080 | 0.439802601 | 0.250694211 | Unknown |
| CNXL_102090 | -0.083084235 | 0.981240834 | Unknown |
| CNXL_102100 | -0.519152127 | 0.901596087 | Unknown |
| CNXL_102110 | -0.648244356 | 0.176771646 | Unknown |
| CNXL_102120 | 0.182653446 | 0.863513463 | Unknown |
| CNXL_102130 | 0.868930071 | 0.033635478 | Unknown |
| CNXL_102140 | 0.03622475 | NA | Unknown |
| CNXL_102150 | -0.039449714 | 0.981851244 | Unknown |
| CNXL_102160 | 0.739220578 | 0.437062442 | Unknown |
| CNXL_102170 | 0.191790896 | 0.957169694 | Unknown |
| CNXL_102180 | -1.238796914 | 0.747701692 | Unknown |
| CNXL_102190 | -0.052011235 | 0.954207791 | Unknown |
| CNXL_102200 | -0.624589254 | 0.751284159 | Unknown |
| CNXL_102210 | -2.471008059 | NA | Unknown |
| CNXL_102220 | -0.251413023 | 0.885685491 | Unknown |
| CNXL_102230 | -0.076662606 | 0.917281026 | Unknown |
| CNXL_102240 | 1.106843139 | 0.002171182 | Unknown |
| CNXL_102250 | -0.630289496 | 0.447871101 | Unknown |
| CNXL_102260 | 0.278130607 | 0.920669589 | Unknown |

| | | | |
|---|---|---|---|
| CNXL_102270 | 1.186139721 | 0.510006134 | Unknown |
| CNXL_102280 | -0.239430255 | 0.920669589 | Unknown |
| CNXL_102290 | 0.796407088 | 0.253430843 | Unknown |
| CNXL_102300 | -0.236473197 | 0.843283188 | Unknown |
| CNXL_102310 | -0.079884214 | 0.918701468 | Unknown |
| CNXL_102320 | -0.552584609 | 0.315529753 | Unknown |
| CNXL_102330 | 1.494211837 | 0.366888453 | Unknown |
| CNXL_102340 | 0.146564916 | 0.809612358 | Unknown |
| CNXL_102350 | -1.243227255 | 0.353576993 | Unknown |
| CNXL_102360 | 1.667060307 | NA | Unknown |
| CNXL_102370 | 0.171442402 | 0.810132107 | Unknown |
| CNXL_102380 | -2.340869333 | 2.58654E-14 | Unknown |
| CNXL_102390 | -0.755480039 | 0.089107701 | Unknown |
| CNXL_102400 | -0.561697299 | 0.884028948 | Unknown |
| CNXL_102410 | 3.117340962 | 1.47471E-27 | Unknown |
| CNXL_102420 | 2.248601138 | 0.061884046 | Unknown |
| CNXL_102430 | 0.742241478 | 0.621719666 | Unknown |
| CNXL_102440 | 0.937936211 | 0.819551095 | Unknown |
| CNXL_102450 | -1.411650781 | 0.701325477 | Unknown |
| CNXL_102460 | 0.636917788 | NA | Unknown |
| CNXL_102470 | -0.897390815 | 0.031370426 | Unknown |
| CNXL_102480 | 1.074082644 | 0.000415933 | Unknown |
| CNXL_102490 | -0.129048253 | 0.982102702 | Unknown |
| CNXL_102500 | 0.280693704 | 0.787481637 | Unknown |
| CNXL_102510 | -0.087735818 | 0.981569994 | Unknown |
| CNXL_102520 | 0.372890456 | 0.465784729 | Unknown |
| CNXL_102530 | 0.841859751 | 0.007859817 | Unknown |
| CNXL_102540 | 0.693661827 | 0.073679745 | Unknown |
| CNXL_102550 | -0.260917091 | 0.958555985 | Unknown |
| CNXL_102560 | -0.421436725 | 0.709122618 | Unknown |
| CNXL_102570 | -0.194220736 | 0.918756814 | Unknown |
| CNXL_102580 | 0.069016474 | 0.932840267 | Unknown |
| CNXL_102590 | 0.233977987 | 0.781361786 | Unknown |
| CNXL_102600 | 0.580363956 | 0.615328522 | Unknown |
| CNXL_102610 | 0.55374168 | 0.681796222 | Unknown |
| CNXL_102620 | 0.181438711 | 0.915837202 | Unknown |
| CNXL_102630 | 0.769222266 | 0.081975892 | Unknown |
| CNXL_102640 | 0.714938796 | 0.383953092 | Unknown |
| CNXL_102650 | -1.599012678 | 0.564998733 | Unknown |
| CNXL_102660 | -2.556800775 | NA | Unknown |
| CNXL_102670 | 1.395163871 | 0.030676501 | Unknown |
| CNXL_102680 | 1.368098283 | 0.071565985 | Unknown |
| CNXL_102690 | 1.0034562 | NA | Unknown |
| CNXL_102700 | -2.050479304 | 0.003552024 | Unknown |
| CNXL_102710 | 0.295437019 | 0.848280332 | Unknown |
| CNXL_102720 | 0.917422523 | 0.536803217 | Unknown |
| CNXL_102730 | 0.991216442 | 0.350224298 | Unknown |
| CNXL_102740 | 0.367561326 | 0.669854065 | Unknown |
| CNXL_102750 | 0.242958157 | 0.883856201 | Unknown |
| CNXL_102760 | 2.52745236 | NA | Unknown |
| CNXL_102770 | NA | NA | Unknown |
| CNXL_102780 | -0.971386803 | 0.392272337 | Unknown |
| CNXL_102790 | -1.697985407 | 0.073966817 | Unknown |
| CNXL_102800 | -0.258930173 | 0.879531297 | Unknown |
| CNXL_102810 | -0.856981597 | 0.388923402 | Unknown |
| CNXL_102820 | 0.532007278 | 0.638378602 | Unknown |
| CNXL_102830 | -0.758999437 | NA | Unknown |
| CNXL_102840 | 0.011162178 | 0.992176872 | Unknown |

| | | | |
|---|---|---|---|
| CNXL_102850 | -1.441758881 | 0.44469198 | Unknown |
| CNXL_102860 | -0.294256326 | 0.936783812 | Unknown |
| CNXL_102870 | 0.366734017 | 0.771343811 | Unknown |
| CNXL_102880 | 3.302920532 | 0.005692302 | Unknown |
| CNXL_102890 | -0.903782173 | 0.023553235 | Unknown |
| CNXL_102900 | -0.793357585 | 0.190652363 | Unknown |
| CNXL_102910 | -0.030673375 | 0.972638293 | Unknown |
| CNXL_102920 | -0.513174865 | 0.857811508 | Unknown |
| CNXL_102930 | -1.039245093 | 0.515160661 | Unknown |
| CNXL_102940 | -0.069589767 | 0.944879244 | Unknown |
| CNXL_102950 | -0.127284623 | 0.884028948 | Unknown |
| CNXL_102960 | -0.135065745 | 0.814480545 | Unknown |
| CNXL_102970 | -0.676048836 | 0.500826403 | Unknown |
| CNXL_102980 | -0.139828214 | 0.935044789 | Unknown |
| CNXL_102990 | 2.609624997 | NA | Unknown |
| CNXL_103000 | -2.364417376 | NA | Unknown |
| CNXL_103010 | 0.34805625 | 0.847311574 | Unknown |
| CNXL_103020 | 0.337017683 | 0.743006144 | Unknown |
| CNXL_103030 | 1.564771686 | 0.000112916 | Unknown |
| CNXL_103040 | 0.21007424 | 0.78495956 | Unknown |
| CNXL_103050 | 0.840784837 | 0.256865157 | Unknown |
| CNXL_103060 | 0.059618805 | 0.946988179 | Unknown |
| CNXL_103070 | 3.075250752 | NA | Unknown |
| CNXL_103080 | 1.153454414 | 0.264480588 | Unknown |
| CNXL_103090 | -0.561736321 | 0.249615143 | Unknown |
| CNXL_103100 | -1.607658931 | 0.634323515 | Unknown |
| CNXL_103110 | 0.178694682 | 0.731482814 | Unknown |
| CNXL_103120 | -4.374985861 | 0.005522962 | Unknown |
| CNXL_103130 | -0.498512083 | 0.399675058 | Unknown |
| CNXL_103140 | 0.697095706 | 0.560249948 | Unknown |
| CNXL_103150 | 0.378411999 | 0.606726524 | Unknown |
| CNXL_103160 | -0.031979549 | 0.970515325 | Unknown |
| CNXL_103170 | 1.677989917 | 0.003050422 | Unknown |
| CNXL_103180 | -0.01405208 | 0.990008547 | Unknown |
| CNXL_103190 | -3.442961076 | 0.271524617 | Unknown |
| CNXL_103200 | 0.361405 | 0.902044607 | Unknown |
| CNXL_103210 | 0.038876611 | NA | Unknown |
| CNXL_103220 | -0.211363004 | 0.826046951 | Unknown |
| CNXL_103230 | -0.86587093 | 0.520208668 | Unknown |
| CNXL_103240 | 0.94648127 | 0.779355929 | Unknown |
| CNXL_103250 | 0.772847706 | 0.864719008 | Unknown |
| CNXL_103260 | 0.063519164 | 0.989960346 | Unknown |
| CNXL_103270 | 2.224426159 | 0.35484081 | Unknown |
| CNXL_103280 | -0.091261907 | 0.943586417 | Unknown |
| CNXL_103290 | -2.453604166 | NA | Unknown |
| CNXL_103300 | 0.2829665 | 0.838178569 | Unknown |
| CNXL_103310 | 1.293534426 | 0.003196301 | Unknown |
| CNXL_103320 | -3.670779171 | 1.3676E-15 | Unknown |
| CNXL_103330 | -1.317491818 | 9.38807E-07 | Unknown |
| CNXL_103340 | -0.849397726 | 0.688533126 | Unknown |
| CNXL_103350 | 0.984428701 | 0.453868281 | Unknown |
| CNXL_103360 | 0.245626585 | 0.746537956 | Unknown |
| CNXL_103370 | 0.210407957 | 0.918171803 | Unknown |
| CNXL_103380 | 0.737143848 | 0.430945378 | Unknown |
| CNXL_103390 | -0.303722727 | 0.670439863 | Unknown |
| CNXL_103400 | -0.977716751 | 0.048491421 | Unknown |
| CNXL_103410 | -1.012805576 | 0.034278503 | Unknown |
| CNXL_103420 | -0.008949876 | 0.992176872 | Unknown |

| | | | |
|---|---|---|---|
| CNXL_103430 | -0.296619396 | 0.902044607 | Unknown |
| CNXL_103440 | -1.254301523 | 0.368365274 | Unknown |
| CNXL_103450 | -1.704724775 | 7.77617E-07 | Unknown |
| CNXL_103460 | 0.530836268 | 0.669668055 | Unknown |
| CNXL_103470 | 3.781244399 | NA | Unknown |
| CNXL_103480 | -2.498680965 | 0.003784105 | Unknown |
| CNXL_103490 | 0.042053553 | 0.96871262 | Unknown |
| CNXL_103500 | -0.716956458 | 0.031508937 | Unknown |
| CNXL_103510 | -0.074259598 | 0.928525448 | Unknown |
| CNXL_103520 | -1.539197468 | NA | Unknown |
| CNXL_103530 | 0.636006738 | NA | Unknown |
| CNXL_103540 | -1.02747087 | 0.072256421 | Unknown |
| CNXL_103550 | 2.781199224 | 0.076801883 | Unknown |
| CNXL_103560 | 0.100262355 | NA | Unknown |
| CNXL_103570 | 2.510226638 | 0.244955281 | Unknown |
| CNXL_103580 | 1.171496571 | 0.431497115 | Unknown |
| CNXL_103590 | 0.194481693 | 0.925141393 | Unknown |
| CNXL_103600 | 0.414707387 | 0.504222021 | Unknown |
| CNXL_103610 | 0.341096754 | 0.746347427 | Unknown |
| CNXL_103620 | -0.494780647 | 0.779687006 | Unknown |
| CNXL_103630 | 0.757048782 | 0.39417374 | Unknown |
| CNXL_103640 | -0.516404071 | 0.83524976 | Unknown |
| CNXL_103650 | 1.590175167 | 0.336746765 | Unknown |
| CNXL_103660 | -0.115261664 | 0.957169694 | Unknown |
| CNXL_103670 | 2.409318079 | NA | Unknown |
| CNXL_103680 | 0.195267273 | 0.782518595 | Unknown |
| CNXL_103690 | -0.4318969 | 0.521375775 | Unknown |
| CNXL_103700 | 0.433995234 | 0.805915487 | Unknown |
| CNXL_103710 | -0.870982363 | 0.649391055 | Unknown |
| CNXL_103720 | -1.11311568 | 0.151765096 | Unknown |
| CNXL_103730 | 0.293345536 | 0.667378962 | Unknown |
| CNXL_103740 | 1.581545135 | 0.197482276 | Unknown |
| CNXL_103750 | 0.697042167 | 0.570636005 | Unknown |
| CNXL_103760 | 0.708909499 | 0.617732365 | Unknown |
| CNXL_103770 | 1.180762621 | 0.000234613 | Unknown |
| CNXL_103780 | -0.061046201 | 0.975403222 | Unknown |
| CNXL_103790 | 1.484927388 | NA | Unknown |
| CNXL_103800 | -2.168700152 | 0.492706776 | Unknown |
| CNXL_103810 | -1.031577643 | 0.00017141 | Unknown |
| CNXL_103820 | -0.444966102 | 0.417866535 | Unknown |
| CNXL_103830 | 0.644014784 | 0.685717343 | Unknown |
| CNXL_103840 | 0.689233427 | 0.295911559 | Unknown |
| CNXL_103850 | 0.221633375 | 0.879531297 | Unknown |
| CNXL_103860 | 0.784475622 | 0.038049615 | Unknown |
| CNXL_103870 | 1.505529639 | 1.15416E-06 | Unknown |
| CNXL_103880 | 0.151066514 | 0.879214963 | Unknown |
| CNXL_103890 | 0.534064644 | 0.317098094 | Unknown |
| CNXL_103900 | 0.265108033 | 0.837479784 | Unknown |
| CNXL_103910 | 0.485529758 | 0.670072271 | Unknown |
| CNXL_103920 | 0.425575178 | 0.703985737 | Unknown |
| CNXL_103930 | -0.183357087 | 0.962706186 | Unknown |
| CNXL_103940 | 0.17264749 | 0.907858192 | Unknown |
| CNXL_103950 | -1.568161329 | 0.540636068 | Unknown |
| CNXL_103960 | -0.650087618 | 0.55949398 | Unknown |
| CNXL_103970 | 0.403240929 | 0.743116933 | Unknown |
| CNXL_103980 | -1.269937432 | NA | Unknown |
| CNXL_103990 | -0.639334983 | 0.806051382 | Unknown |
| CNXL_104000 | 0.517996172 | 0.334783364 | Unknown |

| | | | |
|---|---|---|---|
| CNXL_104010 | 0.457913497 | 0.190906707 | Unknown |
| CNXL_104020 | -0.15523882 | 0.968757042 | Unknown |
| CNXL_104030 | -0.437060965 | 0.857954608 | Unknown |
| CNXL_104040 | 0.449894018 | 0.636723305 | Unknown |
| CNXL_104050 | 0.527533784 | 0.272050531 | Unknown |
| CNXL_104060 | 0.566516711 | NA | Unknown |
| CNXL_104070 | -0.189491318 | 0.884028948 | Unknown |
| CNXL_104080 | 0.231414139 | 0.864293242 | Unknown |
| CNXL_104090 | 0.566515686 | 0.634469107 | Unknown |
| CNXL_104100 | -0.216173953 | 0.883841589 | Unknown |
| CNXL_104110 | -1.936608603 | 0.189297101 | Unknown |
| CNXL_104120 | 0.362575254 | 0.810132107 | Unknown |
| CNXL_104130 | -0.157804034 | 0.954307827 | Unknown |
| CNXL_104140 | -0.138010988 | 0.902969744 | Unknown |
| CNXL_104150 | 0.129355861 | 0.926471551 | Unknown |
| CNXL_104160 | -0.178747085 | 0.879531297 | Unknown |
| CNXL_104170 | 0.142889525 | 0.908877669 | Unknown |
| CNXL_104180 | 0.290255084 | 0.918701468 | Unknown |
| CNXL_104190 | -0.062597548 | 0.981240834 | Unknown |
| CNXL_104200 | 0.282823585 | 0.519652797 | Unknown |
| CNXL_104210 | 1.136687187 | 0.000207353 | Unknown |
| CNXL_104220 | 0.002888388 | 0.997686984 | Unknown |
| CNXL_104230 | 0.441845132 | 0.911128506 | Unknown |
| CNXL_104240 | 1.418416536 | 0.127401224 | Unknown |
| CNXL_104250 | 0.26042482 | 0.724615229 | Unknown |
| CNXL_104260 | 0.716246999 | 0.270995541 | Unknown |
| CNXL_104270 | -0.37344981 | 0.770958815 | Unknown |
| CNXL_104280 | 0.205202982 | 0.689555844 | Unknown |
| CNXL_104290 | 0.507328898 | 0.52947806 | Unknown |
| CNXL_104300 | -0.210307139 | 0.837168643 | Unknown |
| CNXL_104310 | -0.672478193 | 0.385742493 | Unknown |
| CNXL_104320 | -0.779667918 | 0.859615039 | Unknown |
| CNXL_104330 | 0.774659329 | 0.532795674 | Unknown |
| CNXL_104340 | 0.542073243 | 0.265564198 | Unknown |
| CNXL_104350 | 0.409152301 | 0.571561379 | Unknown |
| CNXL_104360 | 0.842322609 | 0.523679914 | Unknown |
| CNXL_104370 | -0.128148726 | 0.908809603 | Unknown |
| CNXL_104380 | 0.416835674 | 0.812703117 | Unknown |
| CNXL_104390 | -0.179327413 | 0.809974479 | Unknown |
| CNXL_104400 | -4.235369743 | NA | Unknown |
| CNXL_104410 | -1.209579985 | 0.311813532 | Unknown |
| CNXL_104420 | 0.299971302 | 0.949084374 | Unknown |
| CNXL_104430 | 0.846407543 | 0.055459171 | Unknown |
| CNXL_104440 | 0.615203272 | 0.519652797 | Unknown |
| CNXL_104450 | 0.934395386 | 0.081569132 | Unknown |
| CNXL_104460 | 0.299330184 | 0.644698534 | Unknown |
| CNXL_104470 | -0.88894789 | 0.417944835 | Unknown |
| CNXL_104480 | 0.173476952 | 0.814220979 | Unknown |
| CNXL_104490 | -0.181250187 | 0.883856201 | Unknown |
| CNXL_104500 | 0.482286225 | 0.445982809 | Unknown |
| CNXL_104510 | 0.749704744 | 0.831980875 | Unknown |
| CNXL_104520 | 1.069609363 | NA | Unknown |
| CNXL_104530 | 0.520640734 | 0.714106807 | Unknown |
| CNXL_104540 | -0.146703855 | 0.963072147 | Unknown |
| CNXL_104550 | 0.448391106 | 0.208201961 | Unknown |
| CNXL_104560 | 0.813371304 | 0.249418771 | Unknown |
| CNXL_104570 | -0.526010969 | 0.595655121 | Unknown |
| CNXL_104580 | -0.240308569 | 0.934642219 | Unknown |

| | | | |
|---|---|---|---|
| CNXL_104590 | 0.914823222 | 0.010542889 | Unknown |
| CNXL_104600 | 0.182976575 | 0.887307365 | Unknown |
| CNXL_104610 | -0.441187304 | 0.614293431 | Unknown |
| CNXL_104620 | 0.664589536 | 0.105214931 | Unknown |
| CNXL_104630 | -1.604930575 | 0.190652363 | Unknown |
| CNXL_104640 | 1.3325926 | 0.064226952 | Unknown |
| CNXL_104650 | 0.377018767 | 0.472392242 | Unknown |
| CNXL_104660 | -0.510534386 | 0.814220979 | Unknown |
| CNXL_104670 | 0.073541098 | 0.918756814 | Unknown |
| CNXL_104680 | -3.368605652 | 9.19114E-06 | Unknown |
| CNXL_104690 | -2.165193575 | 6.71921E-06 | Unknown |
| CNXL_104700 | -0.113157078 | 0.937011645 | Unknown |
| CNXL_104710 | 0.354599963 | 0.699872164 | Unknown |
| CNXL_104720 | -0.470947791 | 0.627258056 | Unknown |
| CNXL_104730 | 0.17154363 | 0.85816747 | Unknown |
| CNXL_104740 | -0.65180709 | 0.417588807 | Unknown |
| CNXL_104750 | -1.946615384 | NA | Unknown |
| CNXL_104760 | 0.389071737 | 0.488583556 | Unknown |
| CNXL_104770 | -4.19605458 | 0.095784948 | Unknown |
| CNXL_104780 | -0.525434123 | 0.754895936 | Unknown |
| CNXL_104790 | -1.64977894 | 0.280284344 | Unknown |
| CNXL_104800 | 0.169460558 | 0.821171149 | Unknown |
| CNXL_104810 | 0.106713248 | 0.965101267 | Unknown |
| CNXL_104820 | 0.461345184 | 0.759678591 | Unknown |
| CNXL_104830 | 0.122246827 | 0.900623322 | Unknown |
| CNXL_104840 | 0.214982059 | 0.874872772 | Unknown |
| CNXL_104850 | 0.38378619 | 0.837479784 | Unknown |
| CNXL_104860 | 1.21417879 | 0.007874443 | Unknown |
| CNXL_104870 | 0.923466728 | 0.007129245 | Unknown |
| CNXL_104880 | 0.023827548 | 0.987423727 | Unknown |
| CNXL_104890 | -0.802172383 | 0.00349153 | Unknown |
| CNXL_104900 | 3.973006798 | 0.139913506 | Unknown |
| CNXL_104910 | -0.695512901 | 0.752406534 | Unknown |
| CNXL_104920 | -0.248484658 | 0.776535714 | Unknown |
| CNXL_104930 | -0.024292836 | 0.989960346 | Unknown |
| CNXL_104940 | -1.533404416 | 0.561580907 | Unknown |
| CNXL_104950 | -0.117263669 | 0.880747649 | Unknown |
| CNXL_104960 | 0.715398359 | 0.540193794 | Unknown |
| CNXL_104970 | 1.028695752 | 0.004917361 | Unknown |
| CNXL_104980 | 1.053632609 | 0.315522035 | Unknown |
| CNXL_104990 | -0.238803952 | 0.935785373 | Unknown |
| CNXL_105000 | -0.10335303 | 0.884028948 | Unknown |
| CNXL_105010 | -0.998336767 | 0.224254322 | Unknown |
| CNXL_105020 | 0.069146416 | 0.987967132 | Unknown |
| CNXL_105030 | 0.84035959 | NA | Unknown |
| CNXL_105040 | 0.127815817 | 0.837479784 | Unknown |
| CNXL_105050 | 0.823437077 | 0.051851032 | Unknown |
| CNXL_105060 | -0.271633104 | 0.843913486 | Unknown |
| CNXL_105070 | -0.023591243 | 0.990777629 | Unknown |
| CNXL_105080 | -0.863062218 | 0.804701377 | Unknown |
| CNXL_105090 | -0.778660948 | 0.683247689 | Unknown |
| CNXL_105100 | -1.440226686 | NA | Unknown |
| CNXL_105110 | 0.550906847 | 0.753324699 | Unknown |
| CNXL_105120 | -0.153073909 | 0.969527869 | Unknown |
| CNXL_105130 | 0.238523611 | 0.869120128 | Unknown |
| CNXL_105140 | -1.24712292 | 0.691220257 | Unknown |
| CNXL_105150 | 0.802408242 | 0.717347987 | Unknown |
| CNXL_105160 | 0.703180902 | 0.66458684 | Unknown |

| | | | |
|---|---|---|---|
| CNXL_105170 | 0.225180028 | 0.746765994 | Unknown |
| CNXL_105180 | 1.189020969 | 0.716771972 | Unknown |
| CNXL_105190 | 1.349595844 | 0.240269875 | Unknown |
| CNXL_105200 | 0.838117479 | 0.589402722 | Unknown |
| CNXL_105210 | -1.105908442 | 0.656993267 | Unknown |
| CNXL_105220 | 0.925621407 | 0.602461418 | Unknown |
| CNXL_105230 | 0.606524826 | 0.414496592 | Unknown |
| CNXL_105240 | 1.125736094 | 0.010417262 | Unknown |
| CNXL_105250 | 0.048003507 | 0.982102702 | Unknown |
| CNXL_105260 | 1.068851234 | 0.199119063 | Unknown |
| CNXL_105270 | 2.533732291 | 0.012189024 | Unknown |
| CNXL_105280 | 2.535927402 | 2.03595E-10 | Unknown |
| CNXL_105290 | 1.073070837 | 0.602461418 | Unknown |
| CNXL_105300 | 0.167042172 | 0.944589864 | Unknown |
| CNXL_105310 | 1.539298834 | 0.249844609 | Unknown |
| CNXL_105320 | -0.695629568 | NA | Unknown |
| CNXL_105330 | -1.838793263 | NA | Unknown |
| CNXL_105340 | -0.060825947 | 0.96843443 | Unknown |
| CNXL_105350 | 0.40619507 | 0.626477949 | Unknown |
| CNXL_105360 | 0.419453297 | 0.926407741 | Unknown |
| CNXL_105370 | -0.769416933 | 0.654601802 | Unknown |
| CNXL_105380 | -0.048482292 | 0.976612116 | Unknown |
| CNXL_105390 | 1.018700766 | 0.814026402 | Unknown |
| CNXL_105400 | 0.343898277 | 0.810030837 | Unknown |
| CNXL_105410 | 0.42161116 | 0.293624485 | Unknown |
| CNXL_105420 | 0.716144638 | 0.710014173 | Unknown |
| CNXL_105430 | 0.415456253 | 0.419095392 | Unknown |
| CNXL_105440 | 0.388613625 | 0.779355929 | Unknown |
| CNXL_105450 | 1.424034604 | 0.112256274 | Unknown |
| CNXL_105460 | 0.198822041 | NA | Unknown |
| CNXL_105470 | -0.959368934 | 0.78976708 | Unknown |
| CNXL_105480 | -0.68037183 | 0.638319466 | Unknown |
| CNXL_105490 | 0.121227295 | 0.82996137 | Unknown |
| CNXL_105500 | 3.148731978 | NA | Unknown |
| CNXL_105510 | -0.812676181 | 0.510243991 | Unknown |
| CNXL_105520 | 0.243197653 | 0.806051382 | Unknown |
| CNXL_105530 | -0.343111819 | 0.918701468 | Unknown |
| CNXL_105540 | 0.360246985 | 0.787481637 | Unknown |
| CNXL_105550 | 0.492164119 | 0.291577712 | Unknown |
| CNXL_105560 | -0.828376446 | 0.192778785 | Unknown |
| CNXL_105570 | 0.04683168 | 0.970515325 | Unknown |
| CNXL_105580 | 0.952955665 | 0.6409676 | Unknown |
| CNXL_105590 | -0.144818802 | 0.933786064 | Unknown |
| CNXL_105600 | -0.628258809 | 0.523048159 | Unknown |
| CNXL_105610 | -0.991534031 | 0.028676041 | Unknown |
| CNXL_105620 | -0.304946361 | 0.820266179 | Unknown |
| CNXL_105630 | 0.614106264 | 0.39417374 | Unknown |
| CNXL_105640 | -0.138955236 | 0.837479784 | Unknown |
| CNXL_105650 | 0.184424361 | 0.902356252 | Unknown |
| CNXL_105660 | 0.108933746 | 0.914338536 | Unknown |
| CNXL_105670 | -0.961291658 | 0.292795277 | Unknown |
| CNXL_105680 | -0.224398659 | 0.884028948 | Unknown |
| CNXL_105690 | 0.311480477 | 0.860561823 | Unknown |
| CNXL_105700 | -2.854301581 | NA | Unknown |
| CNXL_105710 | NA | NA | Unknown |
| CNXL_105720 | 0.702500931 | 0.365784664 | Unknown |
| CNXL_105730 | -0.121516995 | 0.875457166 | Unknown |
| CNXL_105740 | 0.079881937 | 0.957559333 | Unknown |

| | | | |
|---|---|---|---|
| CNXL_105750 | 0.005730581 | 0.996472611 | Unknown |
| CNXL_105760 | 1.051952208 | 0.388647334 | Unknown |
| CNXL_105770 | -0.860823295 | 0.755572574 | Unknown |
| CNXL_105780 | 0.311865317 | 0.720369609 | Unknown |
| CNXL_105790 | -0.57975549 | 0.789040329 | Unknown |
| CNXL_105800 | -1.226379265 | 0.757814488 | Unknown |
| CNXL_105810 | 0.013288468 | 0.988662258 | Unknown |
| CNXL_105820 | 1.484927388 | NA | Unknown |
| CNXL_105830 | -0.36363807 | 0.879531297 | Unknown |
| CNXL_105840 | 0.434152962 | 0.391884891 | Unknown |
| CNXL_105850 | -0.202668717 | 0.936783812 | Unknown |
| CNXL_105860 | 1.177615058 | 0.735571605 | Unknown |
| CNXL_105870 | -0.3758787 | 0.753998183 | Unknown |
| CNXL_105880 | 0.720350099 | 0.172207658 | Unknown |
| CNXL_105890 | 0.126500402 | 0.936086163 | Unknown |
| CNXL_105900 | -0.312547314 | 0.642361001 | Unknown |
| CNXL_105910 | 0.271807672 | 0.560961964 | Unknown |
| CNXL_105920 | -0.653290393 | 0.881830184 | Unknown |
| CNXL_105930 | -4.18449276 | NA | Unknown |
| CNXL_105940 | 1.443365657 | 0.001515528 | Unknown |
| CNXL_105950 | -0.039354266 | 0.974590999 | Unknown |
| CNXL_105960 | -0.604905815 | 0.488456653 | Unknown |
| CNXL_105970 | -0.40687772 | 0.648808974 | Unknown |
| CNXL_105980 | 0.258139538 | 0.771763303 | Unknown |
| CNXL_105990 | -0.269658925 | 0.839816365 | Unknown |
| CNXL_106000 | -0.254483127 | 0.900584045 | Unknown |
| CNXL_106010 | 1.517504309 | 0.644227445 | Unknown |
| CNXL_106020 | 0.259865225 | 0.545597137 | Unknown |
| CNXL_106030 | 0.016738257 | 0.991225434 | Unknown |
| CNXL_106040 | 0.876153523 | 0.006520316 | Unknown |
| CNXL_106050 | 1.249887201 | 0.525585172 | Unknown |
| CNXL_106060 | 0.294059728 | 0.930994697 | Unknown |
| CNXL_106070 | 0.540340694 | 0.367879589 | Unknown |
| CNXL_106080 | -0.038679185 | 0.964076726 | Unknown |
| CNXL_106090 | -0.071842035 | 0.951498478 | Unknown |
| CNXL_106100 | -0.175032318 | 0.891088304 | Unknown |
| CNXL_106110 | -0.365598862 | 0.935317161 | Unknown |
| CNXL_106120 | -0.404588386 | 0.885831771 | Unknown |
| CNXL_106130 | -2.023694566 | 0.561684603 | Unknown |
| CNXL_106140 | -0.094120676 | 0.981569994 | Unknown |
| CNXL_106150 | 0.752171486 | 0.260596728 | Unknown |
| CNXL_106160 | 2.409318079 | NA | Unknown |
| CNXL_106170 | 0.708697583 | 0.703818084 | Unknown |
| CNXL_106180 | 0.506920977 | 0.597680886 | Unknown |
| CNXL_106190 | 0.035351675 | 0.983931833 | Unknown |
| CNXL_106200 | 1.243017401 | 0.231753223 | Unknown |
| CNXL_106210 | 1.271595866 | 0.365148238 | Unknown |
| CNXL_106220 | 0.127261943 | 0.842339591 | Unknown |
| CNXL_106230 | 0.325163942 | 0.515355372 | Unknown |
| CNXL_106240 | 0.988051858 | 0.280743918 | Unknown |
| CNXL_106250 | -0.420849554 | 0.705782303 | Unknown |
| CNXL_106260 | -0.600282012 | 0.862310205 | Unknown |
| CNXL_106270 | -2.364417376 | NA | Unknown |
| CNXL_106280 | 0.118765965 | 0.885449489 | Unknown |
| CNXL_106290 | 0.950628114 | 0.77138893 | Unknown |
| CNXL_106300 | 0.787468843 | 0.335799997 | Unknown |
| CNXL_106310 | -2.159644313 | 0.007904957 | Unknown |
| CNXL_106320 | 0.189951649 | 0.779355929 | Unknown |

| | | | | |
|---|---|---|---|---|
| CNXL_106330 | | 1.302063604 | 2.06459E-05 | Unknown |
| CNXL_106340 | | -0.127675022 | 0.961131838 | Unknown |
| CNXL_106350 | | 0.743462484 | 0.005077052 | Unknown |
| CNXL_106360 | | 0.704113906 | NA | Unknown |
| CNXL_106370 | | -0.931366331 | 0.383250999 | Unknown |
| CNXL_106380 | | -0.364215292 | 0.819989759 | Unknown |
| CNXL_106390 | | -0.539988632 | 0.535382959 | Unknown |
| CNXL_106400 | | 1.047746976 | 0.511541134 | Unknown |
| CNXL_106410 | | 0.714402442 | 0.755572574 | Unknown |
| CNXL_106420 | | -1.212118007 | 0.439840176 | Unknown |
| CNXL_106430 | | 0.461750417 | 0.382582311 | Unknown |
| CNXL_106440 | | 0.071740393 | 0.964414864 | Unknown |
| CNXL_106450 | | 0.819124185 | 0.226848471 | Unknown |
| CNXL_106460 | | 0.10662072 | 0.945301604 | Unknown |
| CNXL_106470 | | -0.188325307 | 0.965465882 | Unknown |
| CNXL_106480 | | 0.72755658 | 0.20516536 | Unknown |
| CNXL_106490 | | 0.105288906 | 0.925523772 | Unknown |
| CNXL_106500 | | 0.024788106 | 0.981240834 | Unknown |
| CNXL_106510 | | 1.303307414 | 0.001398674 | Unknown |
| CNXL_106520 | | -0.02251199 | 0.992176872 | Unknown |
| CNXL_106530 | | 0.804529786 | 0.022368761 | Unknown |
| CNXL_106540 | | -2.110861807 | 0.288694687 | Unknown |
| CNXL_106550 | | 0.399044581 | 0.788934654 | Unknown |
| CNXL_106560 | | -0.337818428 | 0.717347987 | Unknown |
| CNXL_106570 | | 0.471744116 | 0.483451258 | Unknown |
| CNXL_106580 | | 0.252703871 | 0.611024721 | Unknown |
| CNXL_106590 | | -0.684161648 | 0.793332959 | Unknown |
| CNXL_106600 | | -1.603962949 | 4.8009E-07 | Unknown |
| CNXL_106610 | | 0.206338581 | 0.777634355 | Unknown |
| CNXL_106620 | | -0.098388509 | 0.938145045 | Unknown |
| CNXL_106630 | | -1.413980406 | 0.508841455 | Unknown |
| CNXL_106640 | | 0.475003418 | 0.770342269 | Unknown |
| CNXL_106650 | | 0.799125874 | 0.38282089 | Unknown |
| CNXL_106660 | | -0.775125044 | 0.817058875 | Unknown |
| CNXL_106670 | | -0.148593157 | 0.981240834 | Unknown |
| CNXL_106680 | | -0.49320419 | 0.657725818 | Unknown |
| CNXL_106690 | | 1.635067291 | NA | Unknown |
| CNXL_106700 | | -0.22408736 | 0.96470999 | Unknown |
| CNXL_106710 | | 0.578411811 | 0.173210496 | Unknown |
| CNXL_106720 | | 0.31210635 | 0.863513463 | Unknown |
| CNXL_106730 | | 0.740631942 | 0.133472782 | Unknown |
| CNXL_106740 | | 0.373373456 | 0.854323954 | Unknown |
| CNXL_106750 | | -0.134139604 | 0.953095191 | Unknown |
| CNXL_106760 | | 1.488072004 | 1.02397E-05 | Unknown |
| CNXL_106770 | | -0.03421666 | 0.981614017 | Unknown |
| CNXL_106780 | | -0.77672903 | NA | Unknown |
| CNXL_106790 | | -0.135356724 | 0.871884054 | Unknown |
| CNXL_106800 | | -0.280044692 | 0.891760995 | Unknown |
| CNXL_106810 | | 0.5463438 | 0.719557989 | Unknown |
| CNXL_106820 | | 0.852880257 | 0.581039883 | Unknown |
| CNXL_106830 | rze1 | -2.250493816 | 1.63519E-08 | Unknown |
| CNXL_106840 | | -3.636190969 | 1.36153E-12 | Unknown |
| CNXL_106850 | | -0.407698716 | 0.696473798 | Unknown |
| CNXL_106860 | | 0.528776806 | 0.498081161 | Unknown |
| CNXL_106870 | | 0.921349254 | 0.100839804 | Unknown |
| CNXL_106880 | | 0.797865805 | 0.816572532 | Unknown |
| CNXL_106890 | | -1.170954879 | 0.396035434 | Unknown |
| CNXL_106900 | | 0.607198427 | 0.356407473 | Unknown |

| | | | |
|---|---|---|---|
| CNXL_106910 | -0.064313353 | 0.986908239 | Unknown |
| CNXL_106920 | 0.423676336 | 0.810102246 | Unknown |
| CNXL_106930 | NA | NA | Unknown |
| CNXL_106940 | 0.116086634 | 0.917446543 | Unknown |
| CNXL_106950 | -0.287821612 | 0.912929878 | Unknown |
| CNXL_106960 | -4.159014776 | NA | Unknown |
| CNXL_106970 | 0.348685279 | 0.890150525 | Unknown |
| CNXL_106980 | 1.830018786 | 0.25037723 | Unknown |
| CNXL_106990 | -0.667494019 | 0.285705655 | Unknown |
| CNXL_107000 | -1.207532328 | 0.623471638 | Unknown |
| CNXL_107010 | 0.450620511 | 0.909451569 | Unknown |
| CNXL_107020 | 0.227833104 | 0.825693641 | Unknown |
| CNXL_107030 | 1.404404204 | NA | Unknown |
| CNXL_107040 | -0.442683072 | 0.877313027 | Unknown |
| CNXL_107050 | 0.484644819 | 0.68169505 | Unknown |
| CNXL_107060 | 0.117823457 | NA | Unknown |
| CNXL_107070 | -0.45962052 | 0.777311002 | Unknown |
| CNXL_107080 | 0.527652036 | 0.311813532 | Unknown |
| CNXL_107090 | 0.671416223 | 0.61372863 | Unknown |
| CNXL_107100 | 0.529256354 | 0.88179673 | Unknown |
| CNXL_107110 | NA | NA | Unknown |
| CNXL_107120 | 0.830654174 | 0.010703449 | Unknown |
| CNXL_107130 | 0.5008748 | 0.516492158 | Unknown |
| CNXL_107140 | 0.063157558 | 0.985595171 | Unknown |
| CNXL_107150 | 0.431152972 | 0.82996137 | Unknown |
| CNXL_107160 | 1.138193579 | 0.432217251 | Unknown |
| CNXL_107170 | 0.681285897 | 0.5305986 | Unknown |
| CNXL_107180 | 2.1622048 | 9.36837E-06 | Unknown |
| CNXL_107190 | 0.250608378 | 0.779355929 | Unknown |
| CNXL_107200 | -0.1753568 | 0.901676457 | Unknown |
| CNXL_107210 | -0.210728218 | 0.944263762 | Unknown |
| CNXL_107220 | 0.951597528 | 0.666536582 | Unknown |
| CNXL_107230 | 0.988423941 | 0.37530434 | Unknown |
| CNXL_107240 | -0.855113239 | 0.58400495 | Unknown |
| CNXL_107250 | -0.037133871 | 0.988662258 | Unknown |
| CNXL_107260 | 1.484927388 | NA | Unknown |
| CNXL_107270 | 0.244419281 | 0.932765996 | Unknown |
| CNXL_107280 | 0.714232547 | 0.484037787 | Unknown |
| CNXL_107290 | -0.713556986 | 0.623549135 | Unknown |
| CNXL_107300 | 0.470690137 | 0.697372377 | Unknown |
| CNXL_107310 | -0.130543721 | 0.908263861 | Unknown |
| CNXL_107320 | -1.225785909 | 0.358178392 | Unknown |
| CNXL_107330 | -0.967753991 | 0.597638853 | Unknown |
| CNXL_107340 | -0.18377254 | 0.930994697 | Unknown |
| CNXL_107350 | 0.147738486 | 0.881190377 | Unknown |
| CNXL_107360 | -0.039536502 | 0.990777629 | Unknown |
| CNXL_107370 | 0.448300495 | 0.188739467 | Unknown |
| CNXL_107380 | 0.62755886 | 0.619279684 | Unknown |
| CNXL_107390 | -0.17773588 | 0.955507961 | Unknown |
| CNXL_107400 | -0.600223739 | 0.810102246 | Unknown |
| CNXL_107410 | -0.352296507 | 0.926879789 | Unknown |
| CNXL_107420 | 0.19240872 | 0.701325477 | Unknown |
| CNXL_107430 | -2.453604166 | NA | Unknown |
| CNXL_107440 | -0.202055049 | 0.770358678 | Unknown |
| CNXL_107450 | -1.021672878 | 0.005221787 | Unknown |
| CNXL_107460 | -0.334774709 | 0.757194085 | Unknown |
| CNXL_107470 | 1.786750224 | 0.551515946 | Unknown |
| CNXL_107480 | 0.191196732 | 0.920669589 | Unknown |

| | | | |
|---|---|---|---|
| CNXL_107490 | -1.660739907 | 0.101007717 | Unknown |
| CNXL_107500 | 0.923307687 | 0.098349258 | Unknown |
| CNXL_107510 | 0.306822962 | 0.937834503 | Unknown |
| CNXL_107520 | 0.208467385 | 0.935317161 | Unknown |
| CNXL_107530 | -0.738981622 | 0.098458179 | Unknown |
| CNXL_107540 | 0.690127918 | 0.806302201 | Unknown |
| CNXL_107550 | 4.28665956 | NA | Unknown |
| CNXL_107560 | -0.375817664 | 0.735456246 | Unknown |
| CNXL_107570 | -0.622554003 | 0.61372863 | Unknown |
| CNXL_107580 | -0.40867558 | 0.613272076 | Unknown |
| CNXL_107590 | -0.343191458 | NA | Unknown |
| CNXL_107600 | -0.789003549 | 0.134036805 | Unknown |
| CNXL_107610 | 0.957904744 | 0.690752287 | Unknown |
| CNXL_107620 | 0.111231552 | 0.936783812 | Unknown |
| CNXL_107630 | -0.165244352 | 0.816572532 | Unknown |
| CNXL_107640 | -0.687119065 | 0.617244851 | Unknown |
| CNXL_107650 | 0.365717062 | 0.735738116 | Unknown |
| CNXL_107660 | 0.099531758 | 0.947835086 | Unknown |
| CNXL_107670 | 0.564044655 | 0.108539336 | Unknown |
| CNXL_107680 | 0.752925368 | 0.660412993 | Unknown |
| CNXL_107690 | -0.013021392 | 0.992176872 | Unknown |
| CNXL_107700 | -0.798664312 | NA | Unknown |
| CNXL_107710 | 0.301708008 | 0.810102246 | Unknown |
| CNXL_107720 | -3.500385175 | 1.38316E-10 | Unknown |
| CNXL_107730 | -0.343748411 | 0.787846251 | Unknown |
| CNXL_107740 | -0.096633102 | 0.932840267 | Unknown |
| CNXL_107750 | -0.81635954 | 0.612409488 | Unknown |
| CNXL_107760 | -1.688880939 | 0.001515528 | Unknown |
| CNXL_107770 | 0.87739995 | 0.687195736 | Unknown |
| CNXL_107780 | 0.753196988 | 0.132308784 | Unknown |
| CNXL_107790 | 1.38590768 | 0.592514661 | Unknown |
| CNXL_107800 | -0.048020893 | 0.981878601 | Unknown |
| CNXL_107810 | 0.375934127 | 0.615328522 | Unknown |
| CNXL_107820 | -0.058925487 | 0.964414864 | Unknown |
| CNXL_107830 | -0.247044797 | 0.78205771 | Unknown |
| CNXL_107840 | -0.174068016 | 0.830440343 | Unknown |
| CNXL_107850 | 0.834025744 | 0.719248355 | Unknown |
| CNXL_107860 | -0.036028427 | 0.979695435 | Unknown |
| CNXL_107870 | -0.256030197 | 0.746765994 | Unknown |
| CNXL_107880 | 0.459844389 | 0.925206273 | Unknown |
| CNXL_107890 | 0.050643284 | 0.964414864 | Unknown |
| CNXL_107900 | -0.311460807 | 0.906150194 | Unknown |
| CNXL_107910 | 0.517227486 | 0.241458451 | Unknown |
| CNXL_107920 | 0.429633092 | 0.527018595 | Unknown |
| CNXL_107930 | 0.081501417 | NA | Unknown |
| CNXL_107940 | -0.585246933 | 0.534266327 | Unknown |
| CNXL_107950 | 0.297106996 | 0.737538678 | Unknown |
| CNXL_107960 | -0.226200551 | 0.963072147 | Unknown |
| CNXL_107970 | 0.021999249 | 0.987423727 | Unknown |
| CNXL_107980 | 0.15006924 | 0.915884007 | Unknown |
| CNXL_107990 | 0.559375754 | 0.766858347 | Unknown |
| CNXL_108000 | 0.274405726 | 0.632998683 | Unknown |
| CNXL_108010 | 0.400572527 | 0.540948602 | Unknown |
| CNXL_108020 | 0.615201614 | 0.17616683 | Unknown |
| CNXL_108030 | 1.308116059 | 0.305341062 | Unknown |
| CNXL_108040 | 0.625870355 | 0.638319466 | Unknown |
| CNXL_108050 | -0.285382559 | 0.897183423 | Unknown |
| CNXL_108060 | -1.330752087 | NA | Unknown |

| | | | |
|---|---|---|---|
| CNXL_108070 | 1.844934233 | 3.70266E-05 | Unknown |
| CNXL_108080 | 0.309604226 | 0.664916835 | Unknown |
| CNXL_108090 | 0.117823457 | NA | Unknown |
| CNXL_108100 | 0.310929352 | 0.893955723 | Unknown |
| CNXL_108110 | -0.091045341 | 0.934402446 | Unknown |
| CNXL_108120 | 0.258942445 | 0.826907872 | Unknown |
| CNXL_108130 | -0.103863676 | 0.970515325 | Unknown |
| CNXL_108140 | 0.293865115 | 0.677367265 | Unknown |
| CNXL_108150 | 0.036388574 | 0.974108291 | Unknown |
| CNXL_108160 | -0.630836341 | 0.564057694 | Unknown |
| CNXL_108170 | 0.541877083 | 0.280284344 | Unknown |
| CNXL_108180 | 1.606039355 | 5.52776E-06 | Unknown |
| CNXL_108190 | -1.454990775 | 0.695259807 | Unknown |
| CNXL_108200 | -0.029139839 | 0.989960346 | Unknown |
| CNXL_108210 | 0.121990848 | 0.865015851 | Unknown |
| CNXL_108220 | 0.502653893 | 0.183546741 | Unknown |
| CNXL_108230 | 0.624476472 | 0.700912881 | Unknown |
| CNXL_108240 | 0.251970951 | 0.734629931 | Unknown |
| CNXL_108250 | 0.048860972 | 0.972638293 | Unknown |
| CNXL_108260 | 1.224220476 | 0.007280287 | Unknown |
| CNXL_108270 | 0.404426679 | 0.865015851 | Unknown |
| CNXL_108280 | 0.959954001 | 0.219993648 | Unknown |
| CNXL_108290 | 1.075298236 | 0.495174856 | Unknown |
| CNXL_108300 | -0.006342664 | 0.996374193 | Unknown |
| CNXL_108310 | 0.948543658 | 0.742187954 | Unknown |
| CNXL_108320 | 0.166853671 | 0.817410012 | Unknown |
| CNXL_108330 | 0.322851018 | 0.716869545 | Unknown |
| CNXL_108340 | 0.453782763 | 0.840322617 | Unknown |
| CNXL_108350 | -1.413980406 | 0.508841455 | Unknown |
| CNXL_108360 | -1.050333367 | 0.633915651 | Unknown |
| CNXL_108370 | 0.499108388 | 0.75185116 | Unknown |
| CNXL_108380 | -0.802462765 | 0.062177955 | Unknown |
| CNXL_108390 | -0.645770982 | 0.432050869 | Unknown |
| CNXL_108400 | 0.116253923 | 0.980730226 | Unknown |
| CNXL_108410 | -1.330752087 | NA | Unknown |
| CNXL_108420 | 0.055216406 | 0.950738066 | Unknown |
| CNXL_108430 | -1.178944225 | 0.762405378 | Unknown |
| CNXL_108440 | 0.397972664 | 0.746537956 | Unknown |
| CNXL_108450 | -0.297108092 | 0.58502688 | Unknown |
| CNXL_108460 | 0.841772328 | 0.022790785 | Unknown |
| CNXL_108470 | -0.208621528 | 0.90384925 | Unknown |
| CNXL_108480 | 0.205007257 | 0.850747333 | Unknown |
| CNXL_108490 | 0.006166066 | 0.996472611 | Unknown |
| CNXL_108500 | 0.71697069 | 0.381984565 | Unknown |
| CNXL_108510 | 0.936306843 | 0.116106378 | Unknown |
| CNXL_108520 | 1.162017411 | 0.172864329 | Unknown |
| CNXL_108530 | -0.483230591 | 0.814730279 | Unknown |
| CNXL_108540 | -0.158866285 | 0.93760092 | Unknown |
| CNXL_108550 | -0.199370703 | 0.782678074 | Unknown |
| CNXL_108560 | 0.480758714 | 0.654601802 | Unknown |
| CNXL_108570 | -2.978804168 | NA | Unknown |
| CNXL_108580 | -0.940871462 | 0.001830484 | Unknown |
| CNXL_108590 | -0.759388852 | 0.672235027 | Unknown |
| CNXL_108600 | 0.401544528 | 0.378967325 | Unknown |
| CNXL_108610 | 0.364696941 | 0.732619483 | Unknown |
| CNXL_108620 | 2.721506531 | NA | Unknown |
| CNXL_108630 | 0.519754963 | NA | Unknown |
| CNXL_108640 | 1.21868542 | 0.779687006 | Unknown |

| | | | |
|---|---|---|---|
| CNXL_108650 | 0.111604259 | 0.876987041 | Unknown |
| CNXL_108660 | -1.210194364 | 0.771244855 | Unknown |
| CNXL_108670 | 0.289336552 | 0.920669589 | Unknown |
| CNXL_108680 | 0.691260933 | 0.030574464 | Unknown |
| CNXL_108690 | 0.020123152 | 0.992176872 | Unknown |
| CNXL_108700 | -0.407827142 | 0.803866606 | Unknown |
| CNXL_108710 | 0.922021634 | 0.741662589 | Unknown |
| CNXL_108720 | -0.279053689 | 0.902229878 | Unknown |
| CNXL_108730 | 1.698929902 | 0.373482965 | Unknown |
| CNXL_108740 | 1.13088761 | 0.575791977 | Unknown |
| CNXL_108750 | -0.35126538 | 0.816572532 | Unknown |
| CNXL_108760 | 0.959789441 | 0.065641245 | Unknown |
| CNXL_108770 | 0.878072081 | 0.65448449 | Unknown |
| CNXL_108780 | 0.002769809 | 0.996833113 | Unknown |
| CNXL_108790 | -1.18667078 | 0.168034195 | Unknown |
| CNXL_108800 | 0.320345561 | 0.624278162 | Unknown |
| CNXL_108810 | 0.596750353 | 0.12934688 | Unknown |
| CNXL_108820 | -0.053025695 | 0.985595171 | Unknown |
| CNXL_108830 | -0.902749356 | 0.06204509 | Unknown |
| CNXL_108840 | -1.064900717 | 0.773509113 | Unknown |
| CNXL_108850 | 0.266683909 | 0.61372863 | Unknown |
| CNXL_108860 | 1.159518027 | 0.39417374 | Unknown |
| CNXL_108870 | 0.468703258 | 0.500319755 | Unknown |
| CNXL_108880 | -0.056230088 | 0.983980369 | Unknown |
| CNXL_108890 | -0.210381571 | 0.779355929 | Unknown |
| CNXL_108900 | 0.508287136 | 0.417588807 | Unknown |
| CNXL_108910 | 0.383657204 | 0.392543229 | Unknown |
| CNXL_108920 | 0.201002565 | 0.907858192 | Unknown |
| CNXL_108930 | 0.232895808 | 0.803833694 | Unknown |
| CNXL_108940 | 0.427149473 | NA | Unknown |
| CNXL_108950 | -0.424599993 | 0.756597645 | Unknown |
| CNXL_108960 | -0.752024769 | 0.734629931 | Unknown |
| CNXL_108970 | 1.039403881 | 0.000500206 | Unknown |
| CNXL_108980 | -0.045989582 | 0.965966884 | Unknown |
| CNXL_108990 | 0.668202353 | 0.38167154 | Unknown |
| CNXL_109000 | 0.44174134 | 0.532274673 | Unknown |
| CNXL_109010 | -0.460360599 | 0.877322829 | Unknown |
| CNXL_109020 | 0.158214299 | 0.953145249 | Unknown |
| CNXL_109030 | -0.070406237 | 0.94868362 | Unknown |
| CNXL_109040 | -1.085146994 | 0.080824233 | Unknown |
| CNXL_109050 | -0.904039927 | 0.807491917 | Unknown |
| CNXL_109060 | 0.431537372 | 0.830440343 | Unknown |
| CNXL_109070 | -0.069457455 | 0.934642219 | Unknown |
| CNXL_109080 | 0.902368622 | 0.037783243 | Unknown |
| CNXL_109090 | -0.934368845 | 0.519652797 | Unknown |
| CNXL_109100 | 0.181415347 | 0.793383633 | Unknown |
| CNXL_109110 | 1.073620011 | 0.142113019 | Unknown |
| CNXL_109120 | 0.483049031 | 0.91248949 | Unknown |
| CNXL_109130 | -1.079840547 | 0.584869453 | Unknown |
| CNXL_109140 | 2.680591757 | NA | Unknown |
| CNXL_109150 | 0.93259603 | 0.012537174 | Unknown |
| CNXL_109160 | -0.168652584 | 0.917446543 | Unknown |
| CNXL_109170 | 0.081096666 | 0.95265881 | Unknown |
| CNXL_109180 | 0.037188611 | 0.987958739 | Unknown |
| CNXL_109190 | 0.615484429 | 0.697372377 | Unknown |
| CNXL_109200 | 0.112513073 | 0.943326366 | Unknown |
| CNXL_109210 | -0.205733635 | 0.930994697 | Unknown |
| CNXL_109220 | -0.484876395 | 0.892800379 | Unknown |

| | | | |
|---|---|---|---|
| CNXL_109230 | 0.376332604 | 0.791042353 | Unknown |
| CNXL_109240 | 0.171437661 | NA | Unknown |
| CNXL_109250 | 0.447634577 | 0.337535369 | Unknown |
| CNXL_109260 | 0.489413199 | 0.185652474 | Unknown |
| CNXL_109270 | 0.300572866 | 0.83341953 | Unknown |
| CNXL_109280 | 0.797187177 | 0.603329126 | Unknown |
| CNXL_109290 | -0.819834424 | 0.012537174 | Unknown |
| CNXL_109300 | 1.054913829 | 0.633439709 | Unknown |
| CNXL_109310 | 0.549291015 | 0.791528992 | Unknown |
| CNXL_109320 | -0.179127694 | 0.850544343 | Unknown |
| CNXL_109330 | 1.726151046 | 0.000594297 | Unknown |
| CNXL_109340 | 1.208576839 | NA | Unknown |
| CNXL_109350 | 0.071680402 | 0.965966884 | Unknown |
| CNXL_109360 | 0.403488388 | 0.527018595 | Unknown |
| CNXL_109370 | 0.032887133 | 0.96580389 | Unknown |
| CNXL_109380 | 1.267680165 | 0.049925863 | Unknown |
| CNXL_109390 | 0.81080336 | 0.009833171 | Unknown |
| CNXL_109400 | 0.334577281 | 0.81842519 | Unknown |
| CNXL_109410 | 0.721057972 | 0.019855885 | Unknown |
| CNXL_109420 | -4.831979218 | 0.095432879 | Unknown |
| CNXL_109430 | -1.890376584 | 1.18005E-06 | Unknown |
| CNXL_109440 | 0.163439357 | 0.826907872 | Unknown |
| CNXL_109450 | -0.264116169 | 0.597443741 | Unknown |
| CNXL_109460 | -1.361850045 | 0.336027548 | Unknown |
| CNXL_109470 | -0.079263725 | 0.899194631 | Unknown |
| CNXL_109480 | -0.552723646 | 0.219198011 | Unknown |
| CNXL_109490 | 0.002039404 | 0.996833113 | Unknown |
| CNXL_109500 | 0.174582067 | 0.934642219 | Unknown |
| CNXL_109510 | 3.914828939 | NA | Unknown |
| CNXL_109520 | 0.558660554 | 0.19911361 | Unknown |
| CNXL_109530 | -0.231958553 | 0.73464097 | Unknown |
| CNXL_109540 | 2.915513788 | 0.449136359 | Unknown |
| CNXL_109550 | 1.12491051 | 0.158991381 | Unknown |
| CNXL_109560 | 1.788914437 | 0.289223192 | Unknown |
| CNXL_109570 | 0.31262879 | 0.797632564 | Unknown |
| CNXL_109580 | -0.13526456 | 0.939202685 | Unknown |
| CNXL_109590 | 0.255545411 | 0.78519633 | Unknown |
| CNXL_109600 | -0.391826579 | 0.868129828 | Unknown |
| CNXL_109610 | 0.342390815 | 0.645032535 | Unknown |
| CNXL_109620 | 0.221433896 | 0.886074062 | Unknown |
| CNXL_109630 | -0.158838953 | 0.955330361 | Unknown |
| CNXL_109640 | -0.585182064 | 0.55313399 | Unknown |
| CNXL_109650 | -1.155542267 | 0.732149774 | Unknown |
| CNXL_109660 | NA | NA | Unknown |
| CNXL_109670 | 0.00612975 | 0.996374193 | Unknown |
| CNXL_109680 | 0.821916657 | NA | Unknown |
| CNXL_109690 | -0.287640136 | 0.735749455 | Unknown |
| CNXL_109700 | -1.579128526 | 1.27714E-06 | Unknown |
| CNXL_109710 | 0.015550878 | 0.996472611 | Unknown |
| CNXL_109720 | -1.405551068 | 0.565517945 | Unknown |
| CNXL_109730 | 0.128749435 | 0.847878257 | Unknown |
| CNXL_109740 | -0.253259673 | 0.688637346 | Unknown |
| CNXL_109750 | -0.235588941 | 0.893955723 | Unknown |
| CNXL_109760 | -0.04508861 | 0.970876289 | Unknown |
| CNXL_109770 | 0.121551935 | 0.892800379 | Unknown |
| CNXL_109780 | 0.139081694 | 0.885370637 | Unknown |
| CNXL_109790 | 0.334225192 | 0.605167307 | Unknown |
| CNXL_109800 | 0.09267103 | 0.988427236 | Unknown |

| | | | |
|---|---|---|---|
| CNXL_109810 | -2.508063583 | 0.35231862 | Unknown |
| CNXL_109820 | -2.66870313 | 0.293982732 | Unknown |
| CNXL_109830 | 0.058879544 | 0.990703091 | Unknown |
| CNXL_109840 | -0.918391217 | 0.175322462 | Unknown |
| CNXL_109850 | 0.507053339 | 0.229558864 | Unknown |
| CNXL_109860 | 0.504443598 | 0.844850118 | Unknown |
| CNXL_109870 | 3.408400469 | NA | Unknown |
| CNXL_109880 | 0.107717351 | 0.908263861 | Unknown |
| CNXL_109890 | -0.071938915 | 0.96165252 | Unknown |
| CNXL_109900 | 2.254134154 | 0.168034195 | Unknown |
| CNXL_109910 | -0.188589401 | 0.919145053 | Unknown |
| CNXL_109920 | -0.118989816 | 0.850005462 | Unknown |
| CNXL_109930 | -1.7560863 | 0.046397979 | Unknown |
| CNXL_109940 | -0.572703829 | 0.800552138 | Unknown |
| CNXL_109950 | 0.642647416 | 0.038969375 | Unknown |
| CNXL_109960 | 1.316090895 | 0.233174199 | Unknown |
| CNXL_109970 | -0.09970639 | 0.943268444 | Unknown |
| CNXL_109980 | -1.594175921 | 0.529621712 | Unknown |
| CNXL_109990 | -0.530600055 | 0.75185116 | Unknown |
| CNXL_110000 | 0.395661419 | 0.309128838 | Unknown |
| CNXL_110010 | 0.015921132 | 0.992176872 | Unknown |
| CNXL_110020 | -3.039046962 | NA | Unknown |
| CNXL_110030 | -1.366128688 | NA | Unknown |
| CNXL_110040 | -0.045415181 | 0.981733632 | Unknown |
| CNXL_110050 | -0.778933128 | 0.843913486 | Unknown |
| CNXL_110060 | -0.686179732 | 0.884028948 | Unknown |
| CNXL_110070 | -0.647621988 | 0.46049631 | Unknown |
| CNXL_110080 | 1.945786813 | 0.520208668 | Unknown |
| CNXL_110090 | 0.271628842 | 0.934642219 | Unknown |
| CNXL_110100 | 1.492637907 | 0.357567274 | Unknown |
| CNXL_110110 | -0.543720419 | 0.697372377 | Unknown |
| CNXL_110120 | -0.89816992 | 0.44186596 | Unknown |
| CNXL_110130 | 0.239659106 | 0.790239393 | Unknown |
| CNXL_110140 | 0.832623668 | 0.014401088 | Unknown |
| CNXL_110150 | -0.283871613 | 0.884028948 | Unknown |
| CNXL_110160 | 0.040909819 | 0.994610173 | Unknown |
| CNXL_110170 | NA | NA | Unknown |
| CNXL_110180 | 0.509702422 | 0.837168643 | Unknown |
| CNXL_110190 | 0.865280969 | 0.46632372 | Unknown |
| CNXL_110200 | -0.921760179 | NA | Unknown |
| CNXL_110210 | 0.825613414 | 0.814220979 | Unknown |
| CNXL_110220 | 0.578775852 | 0.620363352 | Unknown |
| CNXL_110230 | 0.507422274 | 0.283889793 | Unknown |
| CNXL_110240 | -0.021061378 | 0.987336046 | Unknown |
| CNXL_110250 | 0.627733061 | 0.74676769 | Unknown |
| CNXL_110260 | 0.006064273 | 0.996158838 | Unknown |
| CNXL_110270 | -0.028420958 | 0.983269736 | Unknown |
| CNXL_110280 | -0.72079509 | 0.614375938 | Unknown |
| CNXL_110290 | -0.920192797 | NA | Unknown |
| CNXL_110300 | -0.214810276 | 0.879555308 | Unknown |
| CNXL_110310 | -1.552857478 | 2.74131E-06 | Unknown |
| CNXL_110320 | 0.082796679 | 0.951160419 | Unknown |
| CNXL_110330 | 0.111584461 | 0.972638293 | Unknown |
| CNXL_110340 | 0.412682844 | 0.560191961 | Unknown |
| CNXL_110350 | -1.695623498 | 0.602461418 | Unknown |
| CNXL_110360 | 0.567754959 | 0.730702424 | Unknown |
| CNXL_110370 | 1.173086278 | 0.435253961 | Unknown |
| CNXL_110380 | -0.275698657 | 0.814220979 | Unknown |

| | | | |
|---|---|---|---|
| CNXL_110390 | NA | NA | Unknown |
| CNXL_110400 | 0.281356627 | 0.535382959 | Unknown |
| CNXL_110410 | -2.737841392 | NA | Unknown |
| CNXL_110420 | 0.454996312 | 0.739671911 | Unknown |
| CNXL_110430 | -2.396289475 | 0.21296764 | Unknown |
| CNXL_110440 | 0.095244509 | 0.909119246 | Unknown |
| CNXL_110450 | 1.291428402 | 0.144402226 | Unknown |
| CNXL_110460 | 0.027485613 | 0.978794024 | Unknown |
| CNXL_110470 | 0.931927763 | 0.539339299 | Unknown |
| CNXL_110480 | -0.529678496 | 0.906757384 | Unknown |
| CNXL_110490 | 1.591874047 | 2.1299E-05 | Unknown |
| CNXL_110500 | 2.316319469 | 0.209971443 | Unknown |
| CNXL_110510 | 1.983894859 | 0.488819252 | Unknown |
| CNXL_110520 | 1.048975767 | 0.139938916 | Unknown |
| CNXL_110530 | -0.517305422 | 0.854323954 | Unknown |
| CNXL_110540 | 0.320289049 | 0.747332879 | Unknown |
| CNXL_110550 | -0.093072265 | 0.968698354 | Unknown |
| CNXL_110560 | 0.415166832 | 0.417866535 | Unknown |
| CNXL_110570 | -0.012585383 | 0.991133859 | Unknown |
| CNXL_110580 | 0.086508106 | 0.879531297 | Unknown |
| CNXL_110590 | 0.916007505 | 0.008655033 | Unknown |
| CNXL_110600 | -1.478150263 | 0.474675516 | Unknown |
| CNXL_110610 | 2.445452555 | NA | Unknown |
| CNXL_110620 | 0.730749962 | 0.479195874 | Unknown |
| CNXL_110630 | -0.331324253 | 0.742187954 | Unknown |
| CNXL_110640 | 0.379056607 | 0.410519266 | Unknown |
| CNXL_110650 | 0.480016783 | 0.714520393 | Unknown |
| CNXL_110660 | -0.76017788 | 0.190282816 | Unknown |
| CNXL_110670 | 0.269212622 | 0.877765755 | Unknown |
| CNXL_110680 | 0.133315555 | 0.949084374 | Unknown |
| CNXL_110690 | -0.552442456 | 0.445832045 | Unknown |
| CNXL_110700 | 3.827223779 | NA | Unknown |
| CNXL_110710 | -0.143833233 | 0.92070335 | Unknown |
| CNXL_110720 | -1.175553274 | 0.051851032 | Unknown |
| CNXL_110730 | -0.29612534 | 0.814220979 | Unknown |
| CNXL_110740 | 1.38584768 | 0.055776124 | Unknown |
| CNXL_110750 | 0.295872865 | 0.933324606 | Unknown |
| CNXL_110760 | 0.303270839 | 0.94759673 | Unknown |
| CNXL_110770 | -1.787236733 | 0.156343503 | Unknown |
| CNXL_110780 | 0.729786181 | 0.379616027 | Unknown |
| CNXL_110790 | 0.260356896 | 0.92906347 | Unknown |
| CNXL_110800 | -1.390672173 | 0.072794568 | Unknown |
| CNXL_110810 | -0.404588386 | 0.885831771 | Unknown |
| CNXL_110820 | 0.039512537 | 0.992176872 | Unknown |
| CNXL_110830 | 1.225573924 | 0.005748423 | Unknown |
| CNXL_110840 | -0.645241899 | NA | Unknown |
| CNXL_110850 | 2.301351333 | 0.163963208 | Unknown |
| CNXL_110860 | 0.051362416 | 0.992176872 | Unknown |
| CNXL_110870 | -0.012383421 | 0.995735232 | Unknown |
| CNXL_110880 | -0.050954558 | 0.96843443 | Unknown |
| CNXL_110890 | 0.821509829 | 0.046232519 | Unknown |
| CNXL_110900 | 0.576995061 | 0.572926427 | Unknown |
| CNXL_110910 | -1.560720284 | NA | Unknown |
| CNXL_110920 | -0.621557873 | 0.770559628 | Unknown |
| CNXL_110930 | -0.021699187 | 0.98510145 | Unknown |
| CNXL_110940 | -0.321752664 | 0.683933917 | Unknown |
| CNXL_110950 | -0.652104773 | 0.392610083 | Unknown |
| CNXL_110960 | -2.599312805 | 2.06999E-11 | Unknown |

| | | | |
|---|---|---|---|
| CNXL_110970 | -1.03380467 | 0.077226354 | Unknown |
| CNXL_110980 | -0.51265187 | 0.850544343 | Unknown |
| CNXL_110990 | 0.264825847 | 0.704681578 | Unknown |
| CNXL_111000 | 1.08312023 | NA | Unknown |
| CNXL_111010 | 0.438129299 | 0.593029492 | Unknown |
| CNXL_111020 | -0.210685977 | 0.935785373 | Unknown |
| CNXL_111030 | -5.47441217 | 5.82232E-22 | Unknown |
| CNXL_111040 | 0.537822792 | 0.336008895 | Unknown |
| CNXL_111050 | 0.021306535 | 0.984913368 | Unknown |
| CNXL_111060 | -1.58391283 | 0.110578259 | Unknown |
| CNXL_111070 | 0.644593321 | 0.174232551 | Unknown |
| CNXL_111080 | -0.988701689 | 0.290745908 | Unknown |
| CNXL_111090 | 0.143731179 | 0.813598552 | Unknown |
| CNXL_111100 | 0.745319602 | 0.106779702 | Unknown |
| CNXL_111110 | 0.505456886 | 0.766235579 | Unknown |
| CNXL_111120 | -0.35498038 | 0.879429764 | Unknown |
| CNXL_111130 | 1.579807799 | 0.334301792 | Unknown |
| CNXL_111140 | -1.00104224 | 0.27463373 | Unknown |
| CNXL_111150 | 0.63750176 | 0.06204509 | Unknown |
| CNXL_111160 | 1.421596923 | 0.358178392 | Unknown |
| CNXL_111170 | -3.31902664 | NA | Unknown |
| CNXL_111180 | 0.04590662 | 0.948286148 | Unknown |
| CNXL_111190 | 1.686732929 | 0.432123344 | Unknown |
| CNXL_111200 | 1.012792708 | 0.036002148 | Unknown |
| CNXL_111210 | 3.432784451 | 0.285709409 | Unknown |
| CNXL_111220 | -4.435865063 | 1.97471E-15 | Unknown |
| CNXL_111230 | -0.193441824 | 0.884028948 | Unknown |
| CNXL_111240 | 0.081501417 | NA | Unknown |
| CNXL_111250 | 0.210330811 | 0.920669589 | Unknown |
| CNXL_111260 | -0.619689897 | 0.870564658 | Unknown |
| CNXL_111270 | 0.492268736 | 0.344336085 | Unknown |
| CNXL_111280 | 0.70226389 | 0.365417169 | Unknown |
| CNXL_111290 | 0.797741104 | 0.154160874 | Unknown |
| CNXL_111300 | 0.116406521 | 0.879555308 | Unknown |
| CNXL_111310 | -0.276446686 | 0.893873062 | Unknown |
| CNXL_111320 | -0.06205224 | 0.976006716 | Unknown |
| CNXL_111330 | -1.484151624 | 0.417866535 | Unknown |
| CNXL_111340 | -0.468178383 | 0.650629033 | Unknown |
| CNXL_111350 | 0.620407932 | 0.271915015 | Unknown |
| CNXL_111360 | 0.52317249 | 0.762884467 | Unknown |
| CNXL_111370 | 0.930572386 | NA | Unknown |
| CNXL_111380 | 0.728486449 | 0.201056667 | Unknown |
| CNXL_111390 | -3.291979951 | 0.116094051 | Unknown |
| CNXL_111400 | 0.761920994 | 0.746548581 | Unknown |
| CNXL_111410 | -5.43663716 | 4.79482E-05 | Unknown |
| CNXL_111420 | -1.377232181 | 0.000715453 | Unknown |
| CNXL_111430 | -1.368836688 | NA | Unknown |
| CNXL_111440 | -2.940477827 | 6.92428E-29 | Unknown |
| CNXL_111450 | -0.284799521 | 0.660412993 | Unknown |
| CNXL_111460 | -0.334725803 | 0.664461114 | Unknown |
| CNXL_111470 | 0.236291067 | 0.69536267 | Unknown |
| CNXL_111480 | -0.150849155 | 0.856186476 | Unknown |
| CNXL_111490 | 0.587277988 | 0.518670403 | Unknown |
| CNXL_111500 | -0.764256906 | 0.043302391 | Unknown |
| CNXL_111510 | -0.899626473 | 0.293518315 | Unknown |
| CNXL_111520 | 0.022713006 | 0.984913368 | Unknown |
| CNXL_111530 | 1.300305893 | 0.004398805 | Unknown |
| CNXL_111540 | 0.332077002 | 0.700912881 | Unknown |

| | | | |
|---|---|---|---|
| CNXL_111550 | -0.105376123 | 0.979667462 | Unknown |
| CNXL_111560 | 0.73646993 | 0.225505685 | Unknown |
| CNXL_111570 | 0.259509574 | 0.955330361 | Unknown |
| CNXL_111580 | -0.345319552 | 0.771763303 | Unknown |
| CNXL_111590 | 0.573810466 | 0.706587253 | Unknown |
| CNXL_111600 | 0.10872507 | 0.936783812 | Unknown |
| CNXL_111610 | -0.666771841 | 0.796875929 | Unknown |
| CNXL_111620 | 0.911359676 | 0.191610234 | Unknown |
| CNXL_111630 | -0.663493586 | 0.539585872 | Unknown |
| CNXL_111640 | -0.046877206 | 0.948286148 | Unknown |
| CNXL_111650 | 0.292676042 | 0.784651013 | Unknown |
| CNXL_111660 | 1.484927388 | NA | Unknown |
| CNXL_111670 | 0.917971916 | 0.216214998 | Unknown |
| CNXL_111680 | 0.412797512 | 0.460448053 | Unknown |
| CNXL_111690 | 0.954885053 | 0.03434115 | Unknown |
| CNXL_111700 | 0.402218947 | 0.443697582 | Unknown |
| CNXL_111710 | 0.803257372 | 0.654578621 | Unknown |
| CNXL_111720 | 0.410808327 | 0.653924504 | Unknown |
| CNXL_111730 | 0.275534263 | 0.883503991 | Unknown |
| CNXL_111740 | -0.777198842 | 0.749315941 | Unknown |
| CNXL_111750 | -1.956653081 | 0.027448551 | Unknown |
| CNXL_111760 | 0.180187931 | NA | Unknown |
| CNXL_111770 | -1.556289274 | 0.627713007 | Unknown |
| CNXL_111780 | 0.061666512 | 0.986381348 | Unknown |
| CNXL_111790 | -1.998573043 | 0.15519211 | Unknown |
| CNXL_111800 | 0.625642664 | 0.849067839 | Unknown |
| CNXL_111810 | -0.412273483 | 0.477835242 | Unknown |
| CNXL_111820 | 0.569791765 | 0.16398742 | Unknown |
| CNXL_111830 | 1.354809599 | 0.037035228 | Unknown |
| CNXL_111840 | -0.76049114 | 0.298784347 | Unknown |
| CNXL_111850 | 0.199919238 | 0.901676457 | Unknown |
| CNXL_111860 | -0.196701614 | 0.927441884 | Unknown |
| CNXL_111870 | -3.334805886 | 0.108539336 | Unknown |
| CNXL_111880 | 0.73218428 | NA | Unknown |
| CNXL_111890 | -1.08085642 | 0.579830249 | Unknown |
| CNXL_111900 | 1.28636893 | 0.676573672 | Unknown |
| CNXL_111910 | -0.810457298 | 0.615328522 | Unknown |
| CNXL_111920 | -3.131104808 | 6.07425E-45 | Unknown |
| CNXL_111930 | -3.175796882 | 5.06892E-18 | Unknown |
| CNXL_111940 | -0.680535662 | 0.6409676 | Unknown |
| CNXL_111950 | 1.144301275 | 0.103114946 | Unknown |
| CNXL_111960 | -0.062881176 | 0.988662258 | Unknown |
| CNXL_111970 | 0.126123158 | 0.936086163 | Unknown |
| CNXL_111980 | 1.037398908 | 0.413773209 | Unknown |
| CNXL_111990 | 2.053803957 | NA | Unknown |
| CNXL_112000 | 0.809605425 | 0.015522905 | Unknown |
| CNXL_112010 | 0.112255049 | 0.951498478 | Unknown |
| CNXL_112020 | 0.367056785 | 0.638378602 | Unknown |
| CNXL_112030 | -1.08028857 | 0.708742909 | Unknown |
| CNXL_112040 | -0.015580194 | 0.996472611 | Unknown |
| CNXL_112050 | -0.043373443 | 0.975873016 | Unknown |
| CNXL_112060 | -0.107219136 | 0.885370637 | Unknown |
| CNXL_112070 | 0.299449769 | 0.580839421 | Unknown |
| CNXL_112080 | 0.028298508 | 0.980972278 | Unknown |
| CNXL_112090 | -0.087039812 | 0.918375149 | Unknown |
| CNXL_112100 | -0.306213702 | NA | Unknown |
| CNXL_112110 | 1.265894742 | 0.211421812 | Unknown |
| CNXL_112120 | -0.645241899 | NA | Unknown |

| | | | |
|---|---|---|---|
| CNXL_112130 | -0.543810422 | 0.843098307 | Unknown |

## Table S2: Strains used in this study

| | | |
|---|---|---|
| W5 | XL280α | |
| W380 | XL280**a** | |
| PH136 | XL280α, *NEO* | |
| ZJ8 | XL280α, *NAT* | |
| PH137 | XL280**a**, *HYG* | |
| YXX37 | XL280α, *NAT* | |
| YXX60 | XL280**a**, *NEO* | |
| MS110 | XL280α, *CVA1::NAT* | |
| MS111 | XL280α, *CVA1::NAT* | |
| LHM374 | XL280α, *CVA1::NAT*, $P_{native}$*-CVA1::NEO* | |
| MS83 | H99α, *NEO* | |
| MS101 | H99**a**, *NAT* | |
| Gene deletion set | H99α, CNAG_00795*::NAT* | Madhani HD |
| Gene deletion set | H99α, CNAG_04707::*NAT* | Madhani HD |
| Gene deletion set | H99α, CNAG_01512::*NAT* | Madhani HD |
| YXX40 | XL280α, *CFS1::NAT* | |
| YXX63 | XL280α, *CFS1::G418* | |
| LHM377 | XL280α, *CVA1::NAT* , $P_{native}$ *-CFS1-mCherry::NEO* | |
| LHM378 | XL280α, *CFS1::NAT* , $P_{native}$ - *CFS1-mCherry::NEO* | |

## Table S3: Primers used in this study

| Comments | Primer name | Sequence |
|---|---|---|
| *CVA1* deletion (for XL280) | Wanglab9060 | GGTCGCACCTTGTCATTACG |
| | Wanglab9061 | CTGGCCGTCGTTTTACATGCCATATCGGGCATGTCG |
| | Wanglab9062 | GTCATAGCTGTTTCCTGTCAGCTTCTTACGGCCTCCT |
| | Wanglab9063 | AACTATGGAAGCGCCACCTC |
| | Wanglab9045 | CAGCGGTGTCTGAACAGCCCAACAGTATACCCTGCCGGTG |
| | Wanglab9046 | GGGCTGTTCAGACACCGCTGGTTTTAGAGCTAGAAATAGCAAGTT |
| *CFS1* deletion (for XL280) | wanglab8364 | GCCAATTATTGCGAGTTGCA |
| | wanglab8366 | GGAGTCCGTAGGATGAATGTATG |
| | wanglab8367 | GTCATAGCTGTTTCCTGCACTGATATGGATGGGAGCACA |
| | wanglab8369 | ACCACACGGAAATAGAAATGGA |
| | wanglab8706 | CTGGCCGTCGTTTTACTGAATGGCGGAGGGAATACT |
| | wanglab8362 | GATGAGGTGGTGTTTGTCAGGTTTTAGAGCTAGAAATAGCAAGTT |
| | wanglab8363 | CTGACAAACACCACCTCATCAACAGTATACCCTGCCGGTG |
| *STE3* α qPCR-RT | Wanglab861 | TAGCGGAGCGGACTGGAAAGA |
| | Wanglab862 | CTCGACCGAGACGGCAATCATTA |
| *MF* α qPCR-RT | Wanglab865 | ATCTTCACCACCTTCACTTCT |
| | Wanglab866 | CTAGGCGATGACACAAAGG |
| *TEF1* qPCR-RT | Wanglab857 | CGTCACCACTGAAGTCAAGT |
| | Wanglab858 | AGAAGCAGCCTCCATAGG |
| *MAT2* qPCR-RT | Wanglab859 | GCTCCTCGCTACATCTCCTCA |
| | Wanglab860 | TGTTTCGGTCTACGATACCAGTT |
| *CFS1* qPCR-RT | Wanglab8360 | TCTTCACCCGTACCAAAGACA |
| | Wanglab8361 | CCATAGTCCGCATTAGCACC |
| *CVA1* complementary (for XL280) | Wanglab9518 | CATCACACTGGCGGCCGCGCCTTTGTGAGCAGTTTC |
| | Wanglab9519 | ACTGTAACCCTTAATTAATTTCTTCAGTGAGCGCATG |
| *CFS1* complementary (for XL280) | Wanglab8999 | ATATCCATCACACTGGCGGCCGCAGATAAGCGTCACGGAGCAGC |
| | Wanglab9000 | CCCTTGCTCACCATTGCGATCGCTATCGCGTGATGGTCATCCTT |
